# Design of *C*_1_-symmetric tridentate ligands for enantioselective dearomative [3 + 2] annulation of indoles with aminocyclopropanes

**DOI:** 10.1038/s41467-023-38059-7

**Published:** 2023-04-20

**Authors:** Hai-Xia Wang, Chun Yang, Bai-Yu Xue, Ming-Sheng Xie, Yin Tian, Cheng Peng, Hai-Ming Guo

**Affiliations:** 1 State Key Laboratory of Antiviral Drugs, Pingyuan Laboratory, Key Laboratory of Green Chemical Media and Reactions, Ministry of Education, Collaborative Innovation Center of Henan Province for Green Manufacturing of Fine Chemicals, School of Chemistry and Chemical Engineering, Henan Normal University, Xinxiang, Henan 453007 China; 2 State Key Laboratory of Southwestern Chinese Medicine Resources, School of Pharmacy, Chengdu University of Traditional Chinese Medicine, Chengdu, 611137 China

**Keywords:** Synthetic chemistry methodology, Asymmetric catalysis

## Abstract

Chiral polycyclic indolines are widely present in natural products and have become the focus of extensive synthetic efforts. Here, we show the catalytic asymmetric dearomative [3 + 2] annulation of indoles with donor-acceptor aminocyclopropanes to construct tricyclic indolines. Key to the success of the reaction is the rational design of *C*_1_-symmetric bifunctional tridentate imidazoline-pyrroloimidazolone pyridine ligand. Under 5 mol% of Ni(OTf)_2_-ligand complex, diverse tricyclic indolines containing cyclopentamine moieties are obtained in good chemoselectivities, high diastereoselectivities, and excellent enantioselectivities. An unusual *cis*-configuration ligand is superior to the *trans*-configuration ligand and the corresponding *C*_2_-symmetric tridentate nitrogen ligands in the annulation reaction. Mechanistic studies by control experiments and density functional theory calculations reveal a dual activation manner, where Ni(II) complex activates the aminocyclopropane via coordination with the geminal diester, and imidazolidine NH forms a H-bond with the succinimide moiety.

## Abstract (web summary)

Polycyclic indolines are valuable skeletons in drug discovery. Here, the authors report an asymmetric dearomative [3 + 2] annulation of indoles with aminocyclopropanes to construct tricyclic indolines.

## Introduction

**Fig. 1:**
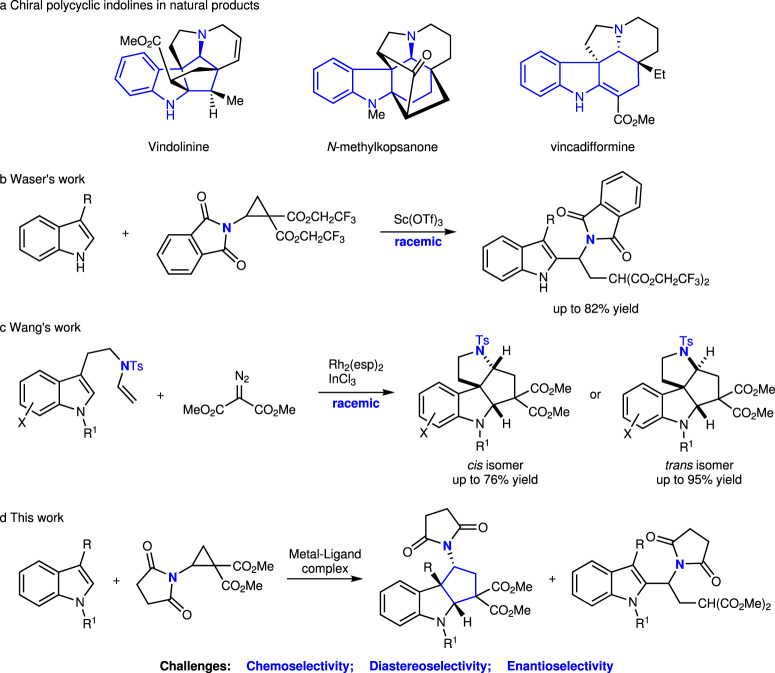
Significances and reactions of D-A cyclopropanes with substituted indoles. **a** Chiral polycyclic indolines containing cyclic amine fragments in natural products. **b** Previous work: the reaction of D-A aminocyclopropanes with C3-substituted indoles produces C2-alkylated product from Waser et al. **c** Previous work: Rh(II) carbene triggered cyclopentannulation reaction of in situ formed D-A aminoacyclpropanes from Wang et al. **d** This work: catalytic asymmetric dearomative [3 + 2] annulation reaction of D-A aminocyclopropanes with C3-substituted indoles. D-A donor–acceptor.

Chiral polycyclic indolines exist widely in natural products, which often contain cyclic amine fragments, such as vindolinine^1^, *N*-methylkopsanone^2^, and vincadifformine^3^ (Fig. 1a). For their construction, catalytic asymmetric dearomatization (CADA) reaction of indoles is a powerful strategy^4–11^. In 2013, Tang and co-workers pioneered the highly enantioselective cyclopentannulation of indoles with donor–acceptor (D-A) aryl cyclopropanes catalyzed by a chiral side armed bisoxazoline (SaBox)-Cu(OTf)_2_ complex^12–17^. Later, Waser and co-workers reported the catalyzed reaction of D-A aminocyclopropanes with C3-substituted indoles, producing C2-alkylated products instead of [3 + 2] annulation products (Fig. 1b)^18^. In 2019, Wang and co-workers developed Rh(II) carbene triggered cyclopentannulation reaction to form diverse polycyclic indolines with high yields, in which D-A aminocyclopropanes were formed in situ (Fig. 1c)^19^. In 2021, Waser and co-workers described the catalytic [3 + 2] annulation of D-A aminocyclopropane monoesters and indoles in high yields and diastereoselectivities catalyzed by triethylsilyl triflimide^20^. In order to construct chiral tricyclic indolines containing cyclopentamine moieties, we envisaged the utilization of the catalytic asymmetric dearomative [3 + 2] annulation reaction of D-A aminocyclopropanes with C3-substituted indoles. However, this approach possesses some challenges related to chemo-, diastereo-, and enantioselectivity (Fig. 1d).

**Fig. 2:**
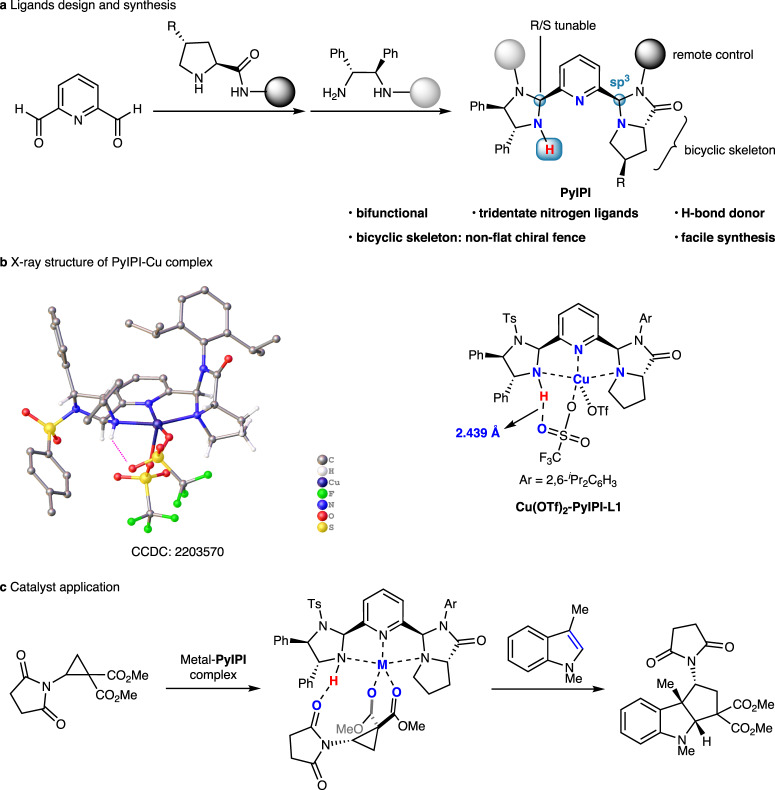
Design, synthesis, and application of PyIPI ligands. **a** Design and synthesis of PyIPI ligands. **b** X-ray structure of Cu(OTf)_2_-PyIPI **L1** complex. **c** Metal-PyIPI complex catalyzed asymmetric dearomative [3 + 2] annulation of indoles with donor–acceptor aminocyclopropane. PyIPI imidazoline-pyrroloimidazolone pyridine.

The design and development efficient chiral tridentate nitrogen ligands bearing a pyridine ring is an attractive goal in asymmetric catalysis^21–28^. *C*_1_-Symmetric tridentate nitrogen ligands have the ability to connect two different chiral moieties, hence, exhibit a greater capacity for structural tuning. Notable *C*_1_-symmetric tridentate nitrogen ligands include pyridyl-based benzimidazolyl-oxazolyl ligand^29^, bis(imino)pyridine^30^, iminopyridine-oxazoline (IPO)^31,32^, oxazoline aminoisopropylpyridine (OAP), imidazoline iminopyridine (IIP) and thiazoline iminopyridine (TIP)^33^. In 2010, Arai and co-workers developed bis(imidazolidine)pyridine (PyBidine) as a highly efficient bifunctional tridentate nitrogen ligand with imidazolidine NH as H-bond donor, and proved that imidazolidine was a privileged chiral moiety^34,35^. Recently, in 2022, our group reported bis(pyrroloimidazolone)pyridine (PyBPI) as a bifunctional tridentate ligand, in which pyrroloimidazolone framework^36,37^ formed a non-flat chiral fence^38^. By incorporating imidazoine and pyrroloimidazolone into the pyridine skeleton, we designed a *C*_1_-symmetric tridentate imidazoline-pyrroloimidazolone pyridine (PyIPI) ligands (Fig. 2a).

PyIPI ligand was facilely synthesized from pyridine-2,6-dicarbaldehyde in twice condensation reactions with L-prolinamide and (*R*,*R*)-diphenylethylenediamine, respectively. PyIPI ligand readily formed a metal complex with Cu(OTf)_2_, which was determined by X-ray diffraction analysis of a single crystal (Fig. 2b). The central Cu metal displayed a pentacoordinated square pyramidal geometry, where three nitrogen atoms and two triflate ligands were bonded to the central metal. Furthermore, H-bonding existed between the imidazolidine NH atom and one oxygen atom of triflate anion, with a distance of 2.439 Å. We hypothesized that PyIPI may exhibit the following features: (1) the tridentate nitrogen ligand could coordinate with different Lewis acids, and imidazolidine NH could act as H-bond donor, thus playing a bifunctional role; (2) the bicyclic pyrroloimidazolone moiety formed a non-flat chiral fence, and exerted an enhanced steric hindrance effect; (3) the configuration of newly formed sp^3^ carbons was tunable, resulting in greater catalyst variability; (4) the steric hindrance of two amine moieties could remotely control the chiral environment created by a tridentate nitrogen ligand; (5) the two different chiral sources were cheap and readily available.

In this work, we envisage that PyIPI coordinates with a metal to activate the aminocyclopropane via bidentate coordination with two carbonyl oxygen atoms, and imidazolidine NH forms a H-bond with the oxygen atom in succinimide moiety, thus resulting in a dual activation and creating a good stereocontrol in [3 + 2] annulation with indoles (Fig. 2c).

## Results

### Optimization study

Initially, the reaction of indole **1a** with racemic succinimido-substituted dimethyl ester cyclopropane **2a** was selected as the model reaction under PyIPI **L1** as the chiral ligand in CH_2_Cl_2_ at 25 °C (Table 1). When Cu(OTf)_2_ was used as the central metal salt, the reaction proceeded in low yield, affording a mixture of tricyclic indoline **3a** and C2 alkylation product **4a** in a ratio of 83:17, in which major product **3a** had 7:1 diastereoselectivity and 81% ee (entry 1). Hence, several metal salts were evaluated (entries 2-8), where Ni(OTf)_2_ showed the best results (91% total yield, **3a**/**4a** = 80:20, 8:1 dr, and 83% ee, entry 7). In the case of PyIPI **L2**, derived from *trans*-4-hydroxyl-L-prolinamide, low ee of **3a** was observed (entry 9). Chiral PyIPI **L3**‒**L6** that contained different sterically hindered amide substituents were tested (entries 10–13). PyIPI **L4**, bearing a phenyl group on aniline, exhibited higher catalytic activity and enhanced stereocontrol (entry 11). Hence, PyIPI **L4** was used as the ligand to examine different nickel salts such as Ni(BF_4_)_2_∙6H_2_O, Ni(ClO_4_)_2_∙6H_2_O, and Ni(OAc)_2_. However, the obtained results did not show any enhancement (Supplementary Table 1). (*S*,*S*)-Diphenylethylenediamine derived PyIPI **L7** with a common *trans*-configuration was employed, resulting in a dramatic decrease in ee value of product **3a** (entry 14), indicating the importance of the unusual *cis*-configuration in **L4**. Utilization of *C*_2_-symmetric tridentate nitrogen ligands **L8** and **L9** showed low catalytic activity and poor ee values (entries 15 and 16), which illustrated the significance of *C*_1_-symmetry in **L4**. Increasing the ratio of aminocyclopropane **2a** to 2.2 equiv., further improved ee value of **3a** with 98% ee (entry 17). Altering the catalytic loading demonstrated that 5 mol% of Ni(OTf)_2_-**L4** complex was optimal; however, even 2.5 mol% of catalyst still afforded product **3a** in 95% ee (entries 18 and 19). In comparison, other commonly used chiral ligands, such as chiral bisoxazoline (Box) and pyridine-bisoxazoline (Pybox) were also tested. However, with ^*t*^Bu-Box or ^*t*^Bu-Pybox as ligands and Ni(OTf)_2_ as the central metal, the reaction did not happen under the same conditions (entries 20 and 21).

**Table 1:** Optimization of the reaction conditions^a^


Entry	Metal	L	x	Yield^b^ (%)	3a/4a^c^	dr^c^	ee^d^ (%)
1	Cu(OTf)_2_	**L1**	10	23	83:17	7:1	81
2	Sc(OTf)_3_	**L1**	10	82	68:32	5:1	2
3	Yb(OTf)_3_	**L1**	10	Trace			
4	Mg(OTf)_2_	**L1**	10	0			
5	Fe(OTf)_3_	**L1**	10	75	70:30	7:1	53
6	Co(OTf)_2_	**L1**	10	79	79:21	8:1	69
7	Ni(OTf)_2_	**L1**	10	91	80:20	8:1	83
8	Zn(OTf)_2_	**L1**	10	65	80:20	8:1	73
9	Ni(OTf)_2_	**L2**	10	95	78:22	6:1	36
10	Ni(OTf)_2_	**L3**	10	93	80:20	9:1	89
11	Ni(OTf)_2_	**L4**	10	95	82:18	10:1	95
12	Ni(OTf)_2_	**L5**	10	92	81:19	10:1	92
13	Ni(OTf)_2_	**L6**	10	95	77:23	10:1	92
14	Ni(OTf)_2_	**L7**	10	90	76:24	6:1	−11
15	Ni(OTf)_2_	**L8**	10	38	75:25	8:1	15
16	Ni(OTf)_2_	**L9**	10	25	80:20	9:1	−2
17^e^	Ni(OTf)_2_	**L4**	10	98	82:18	10:1	98
18^e^	Ni(OTf)_2_	**L4**	5	98	82:18	10:1	98
19^e^	Ni(OTf)_2_	**L4**	2.5	74	80:20	10:1	95
20	Ni(OTf)_2_	^*t*^Bu-Box	5	0			
21	Ni(OTf)_2_	^*t*^Bu-Pybox	5	0			

^a^Reaction conditions: Metal/**L** (1:1.2, x mol%), **1a** (0.1 mmol), **2a** (0.2 mmol) in CH_2_Cl_2_ (2.0 mL) at 25 °C under N_2_ for 24 h.

^b^The total yield (**3a** + **4a**) was determined by ^1^H NMR spectra of the crude product.

^c^The ratio of **3a**/**4a** and dr value of **3a** was determined by ^1^H NMR spectra of the crude product.

^d^The ee value of **3a** was determined by chiral HPLC analysis.

^e^**2a** (0.22 mmol).

### Scope of the reaction

**Fig. 3:**
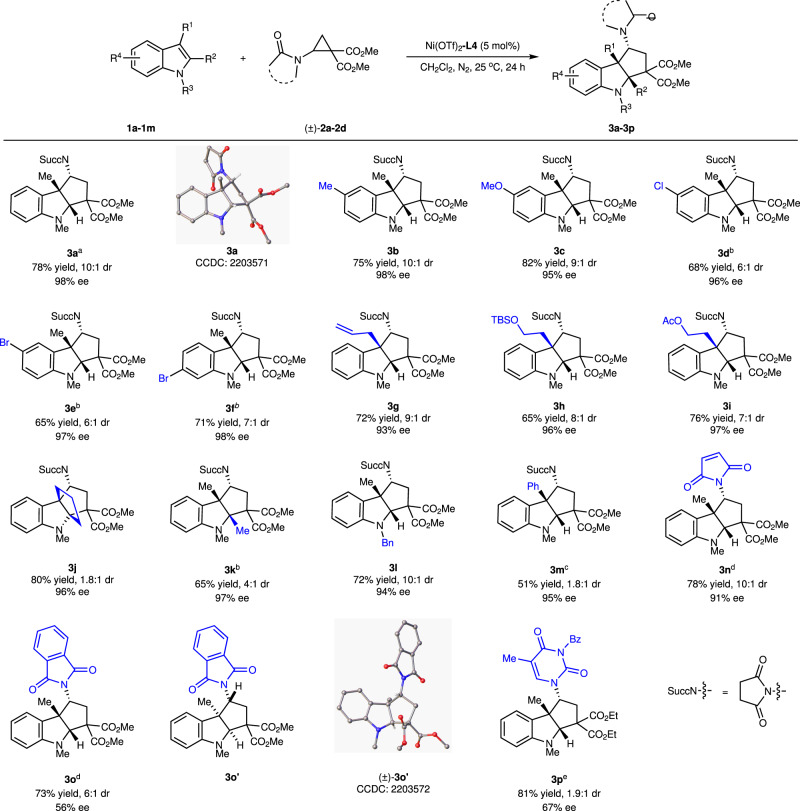
Substrate scope of the reaction. Unless otherwise noted, reaction conditions are as follows: Ni(OTf)_2_/**L4** (1:1.2, 5 mol%), **1** (0.2 mmol), **2** (0.44 mmol) in CH_2_Cl_2_ (4.0 mL) at 25 °C under N_2_ for 24 h. Isolated total yields for product **3** (both diastereoisomers) were reported. The dr value was determined by ^1^H NMR spectra of the crude product. The ee value was determined by chiral HPLC analysis. **a** Reaction conditions: **1a** (0.3 mmol), **2a** (0.66 mmol) in CH_2_Cl_2_ (6.0 mL). **b** Reaction time: 36 h. **c** Reaction conditions: Cu(OTf)_2_/**L4** (1:1.2, 10 mol%) at 50 °C for 64 h. **d** Reaction temperature: 0 °C. **e** Reaction conditions: Cu(OTf)_2_/**L4** (1:1.2, 10 mol%) at 35 °C.

Under optimal reaction conditions (Table 1, entry 18), the substrate scope of the asymmetric dearomative [3 + 2] annulation was explored (Fig. 3). Compared with 5-methyl- and 5-methoxy-substituted indoles **1b** and **1c**, the reaction of 5-chloro- and 5-bromo-substituted indoles **1d** and **1e** were slower, while the influence of electronic effect on enantioselectivity was negligible (**3b**–**3e**). 6-Bromo-substituted indole **1f** afforded annulation product **3f** in satisfactory results. C3-Allyl-substituted indole **1g** and C3-alkoxy-substituted indoles **1h**-**i** also proceeded smoothly, delivering tricyclic indoline **3g-i** in good results with 93–97% ee. In the case of penta-fused indole **1j**, [3,3,3,0]-tetracyclic indoline **3j** was obtained in 96% ee, albeit with low diastereoselectivity. Meanwhile, the reaction of 1,2,3-trimethylindole **1k** also proceeded well, delivering indoline **3k** in 65% yield, 4:1 dr, and 97% ee. It should be pointed out that indoles without a C3 substituent usually afforded only the products of Friedel-Crafts at C3 position, rather than the expected [3 + 2] annulation products (Supplementary Fig. 5). *N*-Benzyl indole **1l** gave [3 + 2] annulation product **3l** in good results. With 3-phenylindole **1m** as the reactant, the [3 + 2] annulation reaction could be occurred with Cu(OTf)_2_-**L4** as the catalyst, affording the tricyclic product **3m** in 51% yield, 1.8:1 dr, and 95% ee. Maleimido-substituted dimethyl ester cyclopropane **2b** was successfully reacted, affording annulation adduct **3n** in good results. Replacing the succinimide group with phthalimide increased reactivity but decreased enantioselectivity (**3a** vs **3o**). With thyminyl-substituted D-A aminocyclopropane **2d** as the reactant, the [3 + 2] annulation could proceed under Cu(OTf)_2_-**L4** as the catalyst, affording chiral thymine carbocyclic nucleoside analog **3p** in 81% yield, 1.9:1 dr, and 67% ee. The absolute configuration of **3a** was assigned by X-ray diffraction analysis. The relative configuration of diastereomer **3o′** was also assigned by X-ray crystallography.

### Kinetic resolution experiment and scale-up reaction

**Fig. 4:**
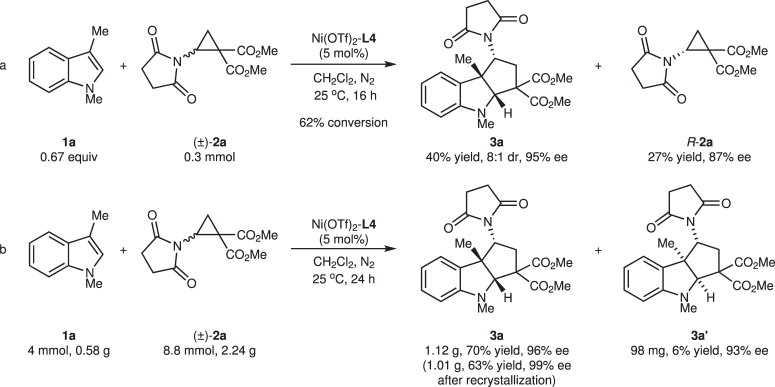
Kinetic resolution experiment and scale-up reaction. **a** Kinetic resolution experiment of 3-methylindole **1a** with aminocyclopropane **2a**. **b** Gram-scale synthesis of tricyclic indoline **3a**.

In the above [3 + 2] annulation reaction, excess aminocyclopropane (2.2 equiv.) was used, which influenced ee value of **3a**. Thus, the kinetic resolution of aminocyclopropane **2a** was performed (Fig. 4a). At 62% conversion, recovered aminocyclopropane **2a** was obtained in 87% ee, and product **3a** was obtained in 40% yield, 8:1 dr, and 95% ee. Furthermore, a gram-scale synthesis of tricyclic indoline **3a** was carried out (Fig. 4b). Using 5 mol% of Ni(OTf)_2_-PyIPI **L4**, 4 mmol of indole **1a** reacted smoothly with aminocyclopropane **2a**, affording 1.12 g (70% yield) of product **3a** with 11:1 dr and 96% ee. After recrystallization, indoline **3a** was obtained as a pure enantiomer (63% yield, 99% ee).

### Mechanistic studies

**Fig. 5:**
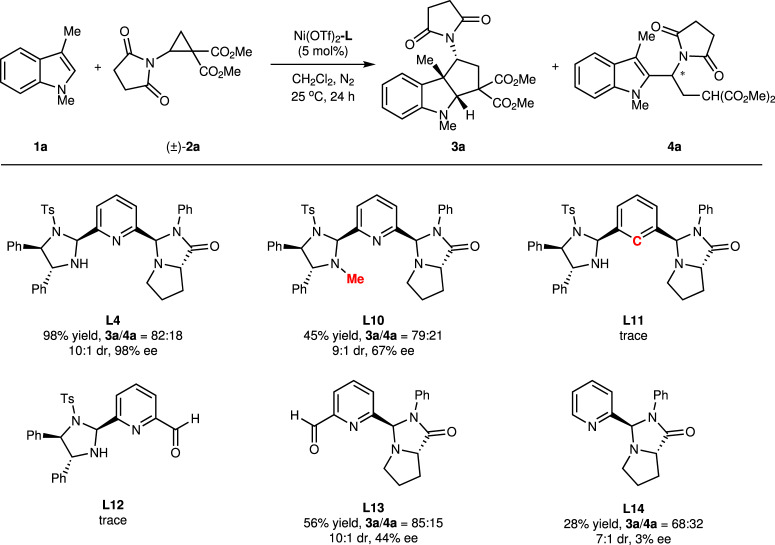
Control experiments. Reaction conditions: Ni(OTf)_2_/**L** (1:1.2, 5 mol%), **1a** (0.1 mmol), **2a** (0.22 mmol) in CH_2_Cl_2_ (2.0 mL) at 25 °C under N_2_ for 24 h; The total yield (**3a** + **4a**) and dr value of **3a** were determined by ^1^H NMR spectra of the crude product; The ee value of **3a** was determined by chiral HPLC analysis.

To understand the mechanism of the reaction catalyzed by Ni(OTf)_2_-PyIPI **L4**, a series of control experiments were performed (Fig. 5). Firstly, ligand **L10**, the *N*-Me derivative of **L4**, was evaluated, generating product **3a** in low yield (45%) and reduced enantioselectivity (67% ee). This indicated that N‒H proton on the imidazolidine moiety was crucial for enhanced reactivity and enantioselectivity. In the case of ligand **L11**, bearing a phenyl ring instead of pyridine ring, only trace amount of product **3a** was obtained. By eliminating the pyrroloimidazolone moiety (ligand **L12**), once again only trace amount of product **3a** was obtained. When ligands **L13** and **L14**, lacking imidazolidine moieties, were examined, the yields and enantioselectivities were poor. Therefore, according to the obtained data the tridentate nitrogen coordinated to Ni(II) ion was essential for reactivity and stereocontrol.

**Fig. 6:**
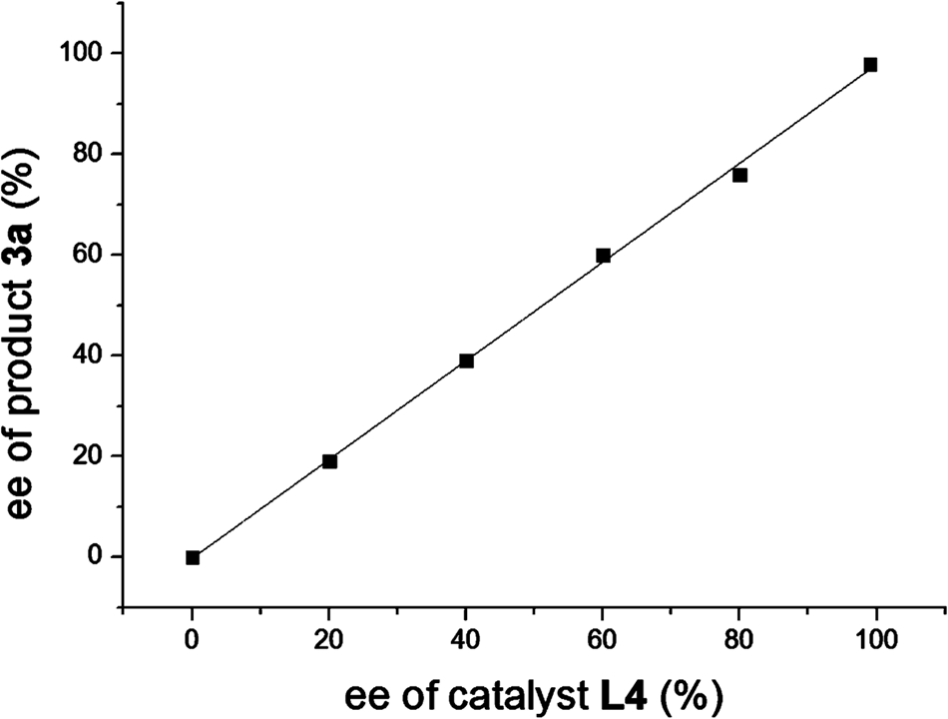
Mechanism investigation. The relationship between ee values of ligand **L4** and product **3a**.

Examination of the relationship between the enantioselectivity of ligand **L4** and product **3a** (Fig. 6) revealed a linear correlation in the catalytic reaction, which indicated that the species, 1:1 ratio of **L4** to Ni(II), may be the active catalytic species for the reaction.

**Fig. 7:**
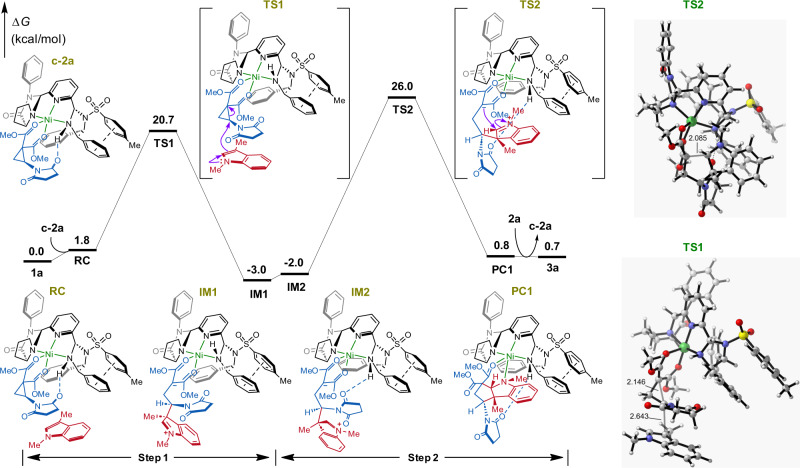
Catalytic reaction pathway. Relative energy profiles (in kcal/mol) of catalyzed reaction pathway and optimized structures of important transition state (bond lengths, Å) at the M06-2X-D3/Def2-TZVP/SMD(DCM)//M06-2X-D3/Def2-SVP level.

The Ni(OTf)_2_-PyIPI **L4**-catalyzed reaction mechanism and stereoselectivity were extensively examined by using density functional theory (DFT). Firstly, the structure of complex **c-2a** was studied in detail by theoretical calculations and X-ray crystallography, which were displayed in Supplementary Figs. 131–132. As described in Fig. 7, the mechanism consisted of a two-step process: ring-opening and ring-closing. In step 1, Ni(OTf)_2_-PyIPI **L4** and (*S*)-aminocyclopropane **2a** generated complex **c**-**2a**, in which pentacoordinated Ni(II) formed square pyramidal geometry due to the steric repulsion of a phenyl group in the imidazolidine moiety of **L4**. PyIPI **L4** acted as a tridentate ligand with three nitrogen atoms coordinated to Ni(II) and (*S*)-**2a** as a bidentate substrate with two carbonyl oxygen atoms coordinated to Ni(II). Meanwhile, the imidazolidine NH of **L4** formed H-bond with the oxygen atom in succinimide moiety, and π–π stacking interaction was observed between two aryl groups in the imidazolidine moiety. Then, the reactant complex (**RC**) was formed from indole **1a** and complex **c**-**2a**, followed by ring-opening of indole **1a** to (*S*)-**2a** from the indole’s *Si* face via transition state **TS1** with the relative free energy of 20.7 kcal/mol. Intermediate **IM1** formed the C3-alkylation indole zwitterion.

In step 2, intermediate **IM2** was generated via bond rotation from **IM1**, which allowed restoration of H-bond between the imidazolidine NH of **L4** and the oxygen atom in succinimide moiety. The ring-closing at C2 position of the indole proceeded via transition state **TS2** with the relative free energy of 26.0 kcal/mol, affording complex **PC1**. Addition of substrate **2a** released tricyclic indoline **3a** and complex **c**-**2a** was regenerated. Based on the energetic span model^39^ of catalytic cycles, the reaction rate was determined by **TS2** (26.0 kcal/mol) and **IM1** (−3.0 kcal/mol).

Furthermore, as shown in Supplementary Fig. 133, there was a bifurcating transition state^40^ **TS3** between **TS1** and **IM1** in the reaction pathways. Meanwhile, the other pathway would lead to intermediate **IM3** which corresponded to product **4a**. This phenomenon corresponeded to 1,2-alkyl group migration^41–43^ in the experimental study. Interestingly, from the viewpoint of the activation energy barrier, the side-pathway was energetically more favored than the main-pathway. However, from another perspective, in the dynamic competition between **IM1** and **IM3**, the nucleophilic attack of N lone pair at the nearby C2 atom in indole **1a** to form a double bond would lead to the dominant **IM1** intermediate. Importantly, the relative free energy of **IM1** was 6.4 kcal/mol lower than that of **IM3**, and was even 3.0 kcal/mol lower than reactant, which indicated that **IM1** was the high fraction stable intermediate. Thus, C3-alkylation product **3a** was the main product and was consistent with the above experiments (Table 1, entry 18).

**Fig. 8:**
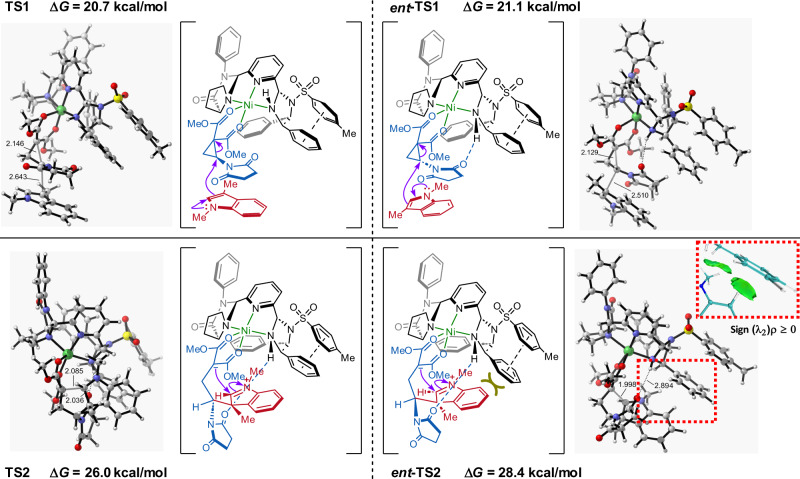
Stereocontrol mechanism. DFT-optimized structures (bond lengths, Å) and relative free energies (∆*G*, kcal/mol) of enantio-determining transition states at the M06-2X-D3/Def2-TZVP/SMD(DCM)//M06-2X-D3/Def2-SVP level of theory.

The stereoselectivity of the [3 + 2] annulation reaction was further explored by comparing the geometry information and free energy of the enantio-determining transition states (Fig. 8). As mentioned above, the ring-closing step was the rate-determining step. The relative free energy of **TS2** was 2.4 kcal/mol (ΔΔ*G*^‡^) lower than that of ***ent*-TS2**, which suggested that (1 *R*,3a*R*,8b*R*)-**3a** was the dominant adduct, and was in good agreement with the above experiments (Table 1, entry 18). The visual analysis of non-covalent interactions (NCI)^44^ for ***ent*-TS2** was performed by independent gradient model based on Hirshfeld partition (IGMH)^45,46^. As shown in the lower right corner of Fig. 8, it indicated that the observed increase of the energy barrier was mainly caused by the obvious steric repulsion between the indoline ring and phenyl group of the imidazoline moiety. In addition, H-bond interaction between imidazolidine NH and oxygen atom in succinimide moiety in **TS2** (2.036 Å) was stronger than that of ***ent*-TS2** (2.894 Å), which was also favorable for lowering the energy barrier in Fig. 8.

## Discussion

The catalytic asymmetric dearomative [3 + 2] annulation of 3-substituted indoles with D-A aminocyclopropanes is realized. *C*_1_-Symmetric bifunctional tridentate PyIPI ligand was rationally designed and facilely synthesized with structurally tunable. With 5 mol% of Ni(OTf)_2_-PyIPI **L4** complex as the catalyst, diverse tricyclic indolines were obtained in moderate to good yields, good chemoselectivities, high diastereoselectivities (up to 10:1 dr), and excellent enantioselectivities (up to 98% ee). To our surprise, PyIPI **L4** with an unusual *cis*-configuration was the optimal ligand, which was found to be better than *C*_2_-symmetric tridentate nitrogen ligands **L8** and **L9** in the annulation reaction. A series of control experiments, as well as single crystal structure of PyIPI **L1**-Cu(OTf)_2_ complex, linear correlation experiment, and DFT calculations, revealed a dual activation mechanism, where the tridentate nitrogen atoms coordinated with Ni(II) to activate aminocyclopropane via bidentate coordination with the geminal diester, followed by imidazolidine NH formed a H-bond with the oxygen atom in succinimide moiety. In addition, high enantioselectivity of the reaction was governed by steric factors. Further applications of chiral PyIPI in other metal-catalyzed asymmetric reactions are currently underway in our research group.

## Methods

### Typical procedure for the synthesis of PyIPI ligand

Pyridine-2,6-dicarbaldehyde (20.0 mmol) and *L*-prolinamides (10.0 mmol) were added to a pressure-resistant test tube with anhydrous ethanol (30.0 mL). Then, the reaction was heated and stirred at 60 °C for 3 h. After that, the reaction mixture was concentrated in vacuo to remove ethanol. The residue was purified by flash column chromatography (Pet/EtOAc, 10/1-1/1, v/v) to give the one-sided condensation product. Then, in a round-bottomed flask containing a stir bar, the above one-sided condensation product (1.0 mmol), (*R*,*R*)-diphenylethylenediamine (1.0 mmol), CH_3_CO_2_H (1.5 mmol, 74.0 μL), and dichloromethane (6.0 mL) were added. Then, the reaction was stirred at 30 °C under N_2_ for 6–8 h. After that, the reaction mixture was quenched by aqueous NaHCO_3_. The organic layer was extracted with dichloromethane for 3 times, and the collected organic layer was dried over Na_2_SO_4_. After removing the solvent under reduced pressure, the resulting residue was purified by silica gel column chromatography to give the corresponding PyIPI ligand.

### Typical procedure for the catalytic asymmetric dearomative [3 + 2] annulation

In a dry reaction tube, a mixture of Ni(OTf)_2_ (3.6 mg, 0.01 mmol, 5 mol%), ligand **L4** (7.9 mg, 0.012 mmol, 6 mol%), and aminocyclopropane **2** (0.44 mmol) in DCM (3.0 mL) were stirred at room temperature for 30 minutes under the atmosphere of nitrogen. Then indole substrate **1** (0.2 mmol) in DCM (1.0 mL) was added to the mixture of catalyst via a syringe. After 24 h, the reaction was complete (monitored by TLC). The reaction was filtered through a glass funnel within layer of silica gel (100–200 mesh) and purified by flash column chromatography (Pet/EtOAc, v/v, 10:1-2:1) to give the product **3**.

## Supplementary information

Supplementary Information

Description of Additional Supplementary Files

Supplementary Data 1

## Data Availability

The data that support the findings of this study is available within the main text and its Supplementary Information file. Source data is provided as Source data file. Data is also available from the corresponding author upon request. The X-ray crystallographic coordinates for structures reported in this study have been deposited at the Cambridge Crystallographic Data Centre (CCDC), under deposition numbers 2203570 (**L1**-Cu(OTf)_2_ complex), 2203566 (**L3**), 2203567 (**L4**), 2203568 (**L8**), 2203569 (**L13**), 2203571 (**3a**), and 2203572 (±**3o′**). These data can be obtained free of charge from The Cambridge Crystallographic Data Centre via www.ccdc.cam.ac.uk/data_request/cif.
