# Supplementary material for: Design of C1-symmetric tridentate ligands for enantioselective dearomative [3 + 2] annulation of indoles with aminocyclopropanes
Source: Nat Commun. 2023 Apr 20;14:2270. doi: 10.1038/s41467-023-38059-7 (PMC10119320; doi:10.1038/s41467-023-38059-7)
Supplement: Supplementary file 1 — Supplementary Information [file 41467_2023_38059_MOESM1_ESM.pdf]

# Supplementary Information

## Design of $C_1$-Symmetric Tridentate Ligands for Enantioselective Dearomative [3+2] Annulation of Indoles with Aminocyclopropanes

Hai-Xia Wang[1,⊥], Chun Yang[1,⊥], Bai-Yu Xue[1], Ming-Sheng Xie[1]*, Yin Tian[2]*, Cheng Peng[2], and Hai-Ming Guo[1]*

[1]State Key Laboratory of Antiviral Drugs, Pingyuan Laboratory, Key Laboratory of Green Chemical Media and Reactions, Ministry of Education, Collaborative Innovation Center of Henan Province for Green Manufacturing of Fine Chemicals, School of Chemistry and Chemical Engineering, Henan Normal University, Xinxiang, Henan 453007, China.

[2]State Key Laboratory of Southwestern Chinese Medicine Resources, School of Pharmacy, Chengdu University of Traditional Chinese Medicine, Chengdu 611137, China

[⊥]These authors contributed equally: Hai-Xia Wang, Chun Yang

Email: xiemingsheng@htu.edu.cn; ytian227@outlook.com; ghm@htu.edu.cn

# Table of Contents


| | |
|---|---|
| Supplementary note 1 | S3-S3 |
| Supplementary method A : synthesis of chiral ligands | S4-S8 |
| Supplementary method B: synthesis of chiral tridentate ligands | S9-S24 |
| General procedure for optimization study | S25-S29 |
| Supplementary method C: enantioselective [3+2] annulation of indoles with cyclopropanes | S30-S50 |
| Supplementary method D: the reaction of 2-substituted indoles with aminocyclopropane | S51-S53 |
| X-ray data of **L3**, **L4**, **L8**, **L13**, **PyIPI L1-Cu complex**, **3a**, and ±**3o'** | S54-S67 |
| HSQC of **L4** | S68-S68 |
| Scale-up reaction and recrystallization | S69-S71 |
| Kinetic resolution experiment of aminocyclopropane **2a** | S72-S76 |
| Nonlinear effect experiment | S77-S81 |
| Transformations of products **3a** | S82-S83 |
| NMR Spectra of all new compounds | S84-S129 |
| HPLC Spectra of all new compounds | S130-S147 |
| Quantum chemical calculations | S148-S149 |
| Supplementary references | S150-S150 |

## Supplementary note 1

$^1$H NMR spectra were recorded on Bruker Avance III HD 600 or Avance 400 MHz spectrometer. Chemical shifts are recorded in ppm relative to tetramethylsilane and with the solvent resonance as the internal standard. Data are reported as follows: chemical shift, multiplicity (s = singlet, d = doublet; t = triplet; q = quartet; quint = quintet; m = multiplet; br = broad), coupling constants (Hz), integration. $^{13}$C NMR data were collected on Bruker Avance III HD 150 or Avance 100 MHz spectrometer. $^1$H NMR spectra was recorded with tetramethylsilane (0.00 ppm) or solvent residual peak ($CDCl_3$: 7.26 ppm; $CD_2Cl_2$: 5.32 ppm) as internal reference. Enantiomer excesses were determined by chiral HPLC analysis on Chiralcel OD-H/IA/IE in comparison with the authentic racemates. Chiral HPLC analysis recorded on Thermo scientific Dionex Ultimate 3000 and Agilent Technologies 1260 Infinity. Optical rotations were reported as follows: $[\alpha]_D^T$ (c: g/100 mL, in solvent). Optical rotations recorded on Autopol Automatic Polarimeter. HRMS was recorded on an ABI/Sciex QStar Mass Spectrometer (ESI). Dichloromethane ($CH_2Cl_2$) and tetrahydrofuran (THF) were purchased extra dry solvents form Innochem Chemical Company. $Ni(OTf)_2$, $Cu(OTf)_2$, $Ni(ClO_4)_2{\cdot}6H_2O$ and $Sc(OTf)_3$ were purchased from Energy Chemical Company. Other solvents used for work-up and purification purposes were purchased in technical grade quality and distilled by rotary evaporator before use. Indole substrates **1a-1o** were prepared according to literature precedents[1-4]. Aminocyclopropanes **2a-d** were prepared according to literature precedents[5-8].

## Supplementary method A: synthesis of chiral ligands

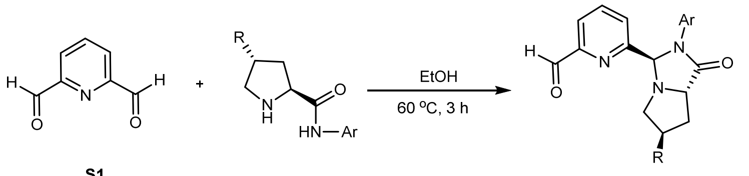


S1
2.0 equiv

S2

S3

**S3a:** R = H; Ar = 2,6-$^{i}Pr_2C_6H_3$
**S3b:** R = OH; Ar = 2,6-$^{i}Pr_2C_6H_3$
**S3c:** R = H; Ar = 2,6-$Et_2C_6H_3$
**S3d:** R = H; Ar = Ph
**S3e:** R = H; Ar = 1-naphthyl
**S3f :** R = H; Ar = Bn

Pyridine-2,6-dicarbaldehyde **S1** (20.0 mmol) and *L*-prolinamides **S2** (10.0 mmol) were added to a pressure-resistant test tube with anhydrous ethanol (30.0 mL). Then, the reaction was heated and stirred at 60 °C (oil bath as the heat source) for 3 h. After that, the reaction mixture was concentrated in vacuo to remove ethanol. The residue was purified by flash column chromatography (Pet/EtOAc, 10/1-1/1, v/v) to give compounds **S3**.

### 6-((3*R*,7*aS*)-2-(2,6-Diisopropylphenyl)-1-oxohexahydro-1*H*-pyrrolo[1,2-*c*] imidazol-3-yl)picolinaldehyde (S3a)

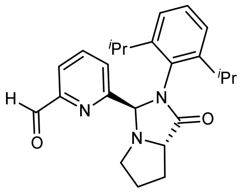

Prepared according to supplementary method A - Pyridine-2,6-dicarbaldehyde **S1a** (2.7 g, 20.0 mmol) and *L*-prolinamide **S2a** (2.74 g, 10.0 mmol) were added to a pressure-resistant test tube with anhydrous ethanol (30.0 mL). Then, the reaction was heated and stirred at 60 °C (oil bath as the heat source) for 3 h. After that, the reaction mixture was concentrated in vacuo to remove ethanol. The residue was purified by flash column chromatography (Pet/EtOAc, 20/1-5:1, v/v) to give **S3a** as a white solid (2.8 g, 72% yield); $R_f$ = 0.51 (Pet/EtOAc, 2/1, v/v), m.p.: 125.6-128.7 °C. $[\alpha]_D^{25.5}$ = -63.2 (*c* = 0.2, in $CHCl_3$). $^1$H NMR (400 MHz, $CDCl_3$) δ 9.86 (s, 1H), 7.84 (d, *J* = 7.6 Hz, 1H), 7.78 (t, *J* = 7.6 Hz, 1H), 7.50 (d, *J* = 7.2 Hz, 1H), 7.28–7.23 (m, 1H), 7.20–7.17 (d, *J* = 7.6 Hz, 1H), 6.92 (d, *J* = 7.6 Hz, 1H), 5.45 (s, 1H), 4.59 (dd, *J* = 8.8, 4.8 Hz. 1H), 3.49–3.42 (m, 1H), 3.14–3.05 (m, 2H), 2.45 (quint, 1H), 2.37–2.21 (m, 2H), 2.05–1.93 (m, 2H), 1.46 (d, *J* = 6.8 Hz, 3H), 1.22 (d, *J* = 6.4 Hz, 3H), 1.03 (d, *J* = 6.8 Hz, 3H), 0.06 (d, *J* = 6.8 Hz, 3H); $^{13}$C NMR (100 MHz, $CDCl_3$) δ 193.4, 174.9, 159.5, 152.9, 147.9, 146.5, 137.9, 129.6, 129.3, 126.7, 124.5, 124.0, 121.0, 87.2, 65.4, 57.1, 29.0, 28.7, 25.5, 25.1,

24.9, 23.6, 23.1; HRMS (m/z): $(M+H)^+$ calcd. for $C_{24}H_{30}N_3O_2^+$, 392.2333; found, 392.2324.

**6-((3*R*,6*R*,7*aS*)-2-(2,6-Diisopropylphenyl)-6-hydroxy-1-oxohexahydro-1*H*-pyrrolo[1,2-*c*]imidazol-3-yl)picolinaldehyde (S3b)**

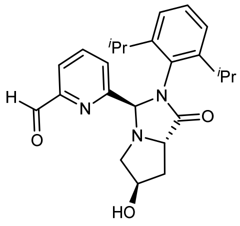


Prepared according to supplementary method A - Pyridine-2,6-dicarbaldehyde **S1a** (540.0 mg, 4.0 mmol) and *trans*-4-hydroxyl-*L*-prolinamide **S2b** (584.0 mg, 2.0 mmol) were added to a pressure-resistant test tube with anhydrous ethanol (8.0 mL). Then, the reaction was heated and stirred at 60 °C (oil bath as the heat source) for 3 h. After that, the reaction mixture was concentrated in vacuo to remove ethanol. The residue was purified by flash column chromatography (Pet/EtOAc, 20/1-2:1, v/v) to give **S3b** as a white solid (448.8 mg, 55% yield); $R_f$ = 0.35 (Pet/EtOAc, 1/1, v/v), m.p.: 183.6-187.1 °C. $[\alpha]_D^{25.5}$ = -53.3 (*c* = 0.2, in $CHCl_3$). $^1H$ NMR (400 MHz, $CDCl_3$) δ 9.85 (s, 1H), 7.86–7.76 (m, 2H), 7.50 (d, *J* = 7.6 Hz, 1H), 7.30–7.23 (m, 1H), 7.19 (d, *J* = 7.6 Hz, 1H), 6.93 (d, *J* = 8.0 Hz, 1H), 5.45 (s, 1H), 4.80 (t, *J* = 7.6, 1H), 4.64 (br, 1H), 3.46 (dd, *J* = 9.6 Hz, 1H), 3.22 (dd, *J* = 10.8, 4.4 Hz, 1H), 3.05 (quint, *J* = 6.8 Hz, 1H), 2.48–2.37 (m, 3 H), 1.45 (d, *J* = 6.8 Hz, 3H), 1.21 (d, *J* = 6.8 Hz, 3H), 1.02 (d, *J* = 6.4 Hz, 3H), 0.05 (d, *J* = 6.8 Hz, 3H); $^{13}C$ NMR (100 MHz, $CDCl_3$) δ 193.3, 174.7, 159.1, 152.8, 147.9, 146.4, 138.0, 129.5, 129.4, 126.7, 124.6, 124.0, 121.2, 86.4, 72.1, 63.8, 63.3, 37.6, 29.0, 28.8, 25.1, 24.8, 23.5, 23.1; HRMS (m/z): $(M+H)^+$ calcd. for $C_{24}H_{30}N_3O_3^+$, 408.2282; found, 408.2274.

**6-((3*R*,7a*S*)-2-(2,6-Diethylphenyl)-1-oxohexahydro-1*H*-pyrrolo[1,2-*c*]imidazol-3-yl)picolinaldehyde (S3c)**

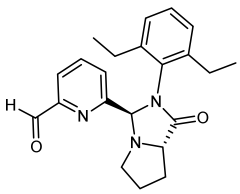

Prepared according to supplementary method A - Pyridine-2,6-dicarbaldehyde **S1a** (540.0 mg, 4.0 mmol) and *L*-prolinamide **S2c** (492.0 mg, 2.0 mmol) were added to a pressure-resistant test tube with anhydrous ethanol (8.0 mL). Then, the reaction was heated and stirred at 60 $^{o}$C (oil bath as the heat source) for 3 h. After that, the reaction mixture was concentrated in vacuo to remove ethanol. The residue was purified by flash column chromatography (Pet/EtOAc, 20/1-4:1, v/v) to give **S3c** as a white solid (399.3 mg, 55% yield); $R_f$ = 0.52 (Pet/EtOAc, 2/1, v/v), m.p.: 132.1.1-135.9 $^{o}$C. $[\alpha]_D^{25.5}$ = -63.2 (*c* = 0.2, in $CHCl_3$). $^1$H NMR (600 MHz, $CDCl_3$) δ 9.78 (s, 1H), 7.81 (d, *J* = 7.8 Hz, 1H), 7.75 (t, *J* = 7.8 Hz, 1H), 7.48 (d, *J* = 7.2 Hz, 1H), 7.17 (t, *J* = 7.2 Hz, 1H), 7.12 (d, *J* = 7.2 Hz, 1H), 6.89 (d, *J* = 7.8 Hz, 1H), 5.50 (s, 1H), 4.52 (q, *J* = 4.8 Hz, 1H), 3.48–3.41 (m, 1H), 3.16–3.08 (m, 1H), 2.76–2.64 (m, 2H), 2.37–2.30 (m, 1H), 2.28–2.20 (m, 1H), 2.03–1.92 (m, 3H), 1.48–1.39 (m, 1H), 1.29 (t, *J* = 7.2 Hz, 3H), 0.64 (t, *J* = 7.2 Hz, 3H); $^{13}$C NMR (100 MHz, $CDCl_3$) δ 193.3, 174.4, 159.2, 152.5, 143.0, 141.6, 137.8, 131.3, 128.8, 126.7, 126.32, 126.28, 120.9, 85.7, 65.3, 56.8, 28.8, 25.4, 25.0, 23.0, 15.1, 13.8; HRMS (m/z): $(M+H)^+$ calcd. for $C_{22}H_{26}N_3O_2^+$, 364.2020; found, 364.2020.

**6-((3*R*,7a*S*)-1-Oxo-2-phenylhexahydro-1*H*-pyrrolo[1,2-*c*]imidazol-3-yl) picolinaldehyde (S3d)**

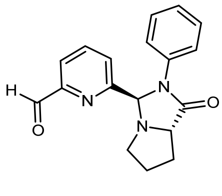

Prepared according to supplementary method A - Pyridine-2,6-dicarbaldehyde **S1a** (1.35 g, 10.0 mmol) and *L*-prolinamide **S2d** (950.0 mg, 5.0 mmol) were added to a pressure-resistant test tube with anhydrous ethanol (15.0 mL). Then, the reaction was heated and stirred at 60 $^{o}$C

(oil bath as the heat source) for 3 h. After that, the reaction mixture was concentrated in vacuo to remove ethanol. The residue was purified by flash column chromatography (Pet/EtOAc, 20/1-4:1, v/v) to give **S3d** as a white solid (844.1 mg, 55% yield). Further purification could be achieved by recrystallization (recrystallization solvent: Pet/ EtOAc) to give **S3d** as a white solid (706.1mg, 46% yield); $R_f$ = 0.48 (Pet/ EtOAc/ $Et_3N$, 2/1/0.001, v/v/v); m.p.: 135.1-137.9 °C. $[\alpha]_D^{25.5}$ = -99.0 (*c* = 0.2, in $CHCl_3$). $^1H$ NMR (400 MHz, $CDCl_3$) δ 10.00 (s, 1H), 7.85–7.78 (m, 2H), 7.45–7.42 (m, 3H), 7.25–7.22 (m, 2H), 7.06 (t, *J* = 7.2 Hz, 1H), 5.83 (s, 1H), 4.15 (t, *J* = 6.8 Hz, 1H), 3.49–3.46 (m, 1H), 3.01–2.95 (m, 1H), 2.21 (q, *J* = 6.8 Hz, 2H), 1.92–1.84 (m, 2H); $^{13}C$ NMR (100 MHz, $CDCl_3$) δ 193.2, 175.0, 159.3, 153.0, 138.5, 137.2, 129.2, 125.5, 124.6, 121.3, 84.3, 64.9, 56.6, 27.9, 24.9; HRMS (m/z): $(M+Na)^+$ calcd. for $C_{18}H_{17}NaN_3O_2^+$, 330.1213; found, 330.1210.

**6-((3*R*,7*aS*)-2-(Naphthalen-1-yl)-1-oxohexahydro-1*H*-pyrrolo[1,2-*c*]imidazol-3-yl) picolinaldehyde (S3e)**

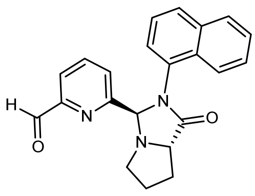

Prepared according to supplementary method A - Pyridine-2,6-dicarbaldehyde **S1a** (135.0 g, 1.0 mmol) and *L*-prolinamide **S2e** (95.0 mg, 0.5 mmol) were added to a pressure-resistant test tube with anhydrous ethanol (4.0 mL). Then, the reaction was heated and stirred at 60 °C (oil bath as the heat source) for 3 h. After that, the reaction mixture was concentrated in vacuo to remove ethanol. The residue was purified by flash column chromatography (Pet/EtOAc, 20/1-1/1, v/v) to give **S3e** as a white solid (103.5 mg, 53% yield); $R_f$ = 0.31 (Pet/EtOAc, 1/1, v/v), m.p.: 100.1-103.1 °C. $[\alpha]_D^{25.5}$ = -51.2 (*c* = 0.2, in $CHCl_3$). $^1H$ NMR (400 MHz, $CDCl_3$) δ 9.96 (s, 1H), 7.86–7.80 (m, 3H), 7.76–7.69 (m, 2H), 7.53–7.45 (m, 2H), 7.36 (d, *J* = 7.6 Hz, 1H), 7.29 (t, *J* = 8.0 Hz, 1H), 7.02 (br, 1H), 5.74 (s, 1H), 4.61 (dd, *J* = 8.4 , 5.2 Hz, 1H), 3.61–3.54 (m, 1H), 3.34–3.25 (m, 1H), 2.42–2.30 (m, 2H), 2.17–1.98 (m, 2H); $^{13}C$ NMR (100 MHz, $CDCl_3$) δ 193.3, 175.7, 159.4, 152.8, 138.1, 134.6, 132.2, 130.1, 128.9, 128.8, 126.9, 126.4, 126.0,

125.5, 122.3, 121.2, 86.4, 65.1, 57.0, 28.3, 25.4; HRMS (m/z): $(M+Na)^+$ calcd. for $C_{22}H_{19}N_3NaO_3^+$, 380.1369; found, 380.1369.

**6-((3*R*,7*aS*)-2-benzyl-1-oxohexahydro-1*H*-pyrrolo[1,2-*c*]imidazol-3-yl)picolinaldehyde (S3f)**

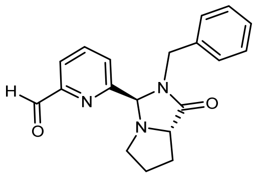

Prepared according to supplementary method A - Pyridine-2,6-dicarbaldehyde **S1a** (405.0 mg, 3.0 mmol) and *L*-prolinamide **S2f** (285.0 mg, 1.5 mmol) were added to a pressure-resistant test tube with anhydrous ethanol (10.0 mL). Then, the reaction was heated and stirred at 60 °C (oil bath as the heat source) for 3 h. After that, the reaction mixture was concentrated in vacuo to remove ethanol.The residue was purified by flash column chromatography (Pet/EtOAc, 20/1-1/1, v/v) to give **S3f** as a yellow oil (313.0 mg, 65% yield); $R_f$ = 0.28 (Pet/EtOAc, 1/1, v/v), $[\alpha]_D^{25.5}$ = -43.6 (*c* = 0.2, in $CHCl_3$). $^1H$ NMR (600 MHz, $CDCl_3$) δ 10.0 (s, 1H), 7.96 (d, *J* = 7.2 Hz, 1H), 7.88 (t, *J* = 7.2 Hz, 1H), 7.41 (d, *J* = 7.8 Hz, 1H), 7.23–7.16 (m, 3H), 6.94 (d, *J* = 4.8 Hz, 2H), 5.59 (s, 1H), 5.02 (d, *J* = 14.4 Hz, 1H), 4.04 (d, *J* = 15.0 Hz, 1H), 3.96–3.90 (m, 1H), 2.56–2.47 (m, 1H), 2.26–2.10 (m, 3H), 1.74–1.61 (m, 2H); $^{13}C$ NMR (100 MHz, $CDCl_3$) δ 193.2, 177.1, 155.5, 152.8, 138.0, 135.8, 128.7, 128.4, 128.0, 127.8, 121.4, 78.4, 65.7, 48.9, 45.6, 27.1, 25.4; HRMS (m/z): $(M+H)^+$ calcd. for $C_{19}H_{20}N_3O_3^+$, 322.1550; found, 322.1549.

# Supplementary method B: synthesis of chiral tridentate ligands

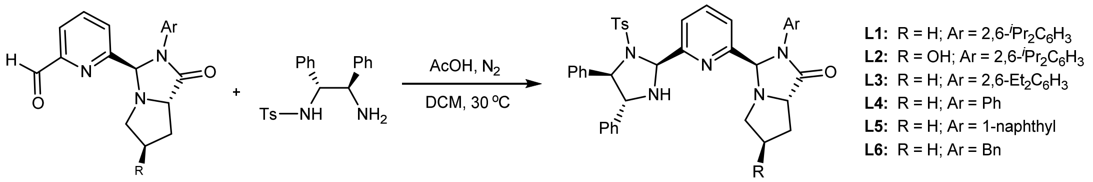


**S3** **S4a** **L1-6**

In a round-bottomed flask containing a stir bar, compound **S3** (1.0 mmol), (*R*,*R*)-TsDPEN **S4a** (1.0 mmol), AcOH (1.5 mmol, 74.0 μL), and dichloromethane (6.0 mL) were added. Then, the reaction was stirred at 30 °C under $N_2$ for 6-8 h. After that, the reaction mixture was quenched by aqueous $NaHCO_3$. The organic layer was extracted with dichloromethane for 3 times, and the collected organic layer was dried over $Na_2SO_4$. After removing the solvent under reduced pressure, the resulting residue was purified by silica gel column chromatography to give ligands **L1-6**.

**(3*R*,7a*S*)-2-(2,6-Diisopropylphenyl)-3-(6-((2*S*,4*R*,5*R*)-4,5-diphenyl-1-tosylimidazolidin-2-yl)pyridin-2-yl)hexahydro-1*H*-pyrrolo[1,2-*c*]imidazol-1-one (L1)**

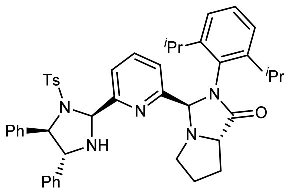

Prepared according to supplementary method B - In a round-bottomed flask containing a stir bar, compound **S3a** (391.0 mg, 1.0 mmol), (*R*,*R*)-TsDPEN **S4a** (366.0 mg, 1.0 mmol), AcOH (1.5 mmol, 74.0 μL), and dichloromethane (10.0 mL) were added. Then, the reaction was stirred at 30 °C under $N_2$ for 6 h. After that, the reaction mixture was quenched by aqueous $NaHCO_3$. The organic layer was extracted with dichloromethane for 3 times, and the collected organic layer was dried over $Na_2SO_4$. After removing the solvent under reduced pressure, the resulting residue was purified by silica gel column chromatography to give **L1** as a white solid (591.2 mg, 80% yield). Further purification can be achieved by recrystallization (recrystallization solvent: Pet/EtOAc) to obtain the chiral tridentate ligand **L1** as a

white solid (546.9 mg, 74% yield); $R_f$ = 0.65 ($CH_2Cl_2$/MeOH, 30/1, v/v); m.p.: 196.5-198.3 °C. $[\alpha]_D^{25.5}$ = -113.0 (*c* = 0.2, in $CHCl_3$). $^1$H NMR (400 MHz, $CDCl_3$) δ 7.87 (t, *J* = 7.6 Hz, 1H), 7.80 (d, *J* = 7.2 Hz, 1H), 7.72 (d, *J* = 7.6 Hz, 1H), 7.52 (d, *J* = 8.0 Hz, 2H), 7.21–7.10 (m, 10H), 7.06 (t, *J* = 8.0 Hz 1H), 6.94–6.86 (m, 4H), 5.62 (s, 1H), 5.32 (s, 1H), 4.47 (d, *J* = 6.0 Hz, 1H), 4.36–4.32 (m, 1H), 4.15 (d, *J* = 6.4 Hz, 1H), 3.44–3.38 (m, 1H), 3.08–3.02 (m, 1H), 2.92 (quint, *J* = 6.8 Hz, 1H), 2.82 (br, 1H), 2.41 (s,3H), 2.35–2.27 (m, 1H), 2.23–2.15 (m, 2H), 2.06–1.89 (m, 2H), 1.12 (d, *J* = 6.8 Hz, 3H), 1.06 (d, *J* = 6.8 Hz, 3H), 0.98 (d, *J* = 6.8 Hz, 3H), 0.22 (d, *J* = 6.8, 3H); $^{13}$C NMR (100 MHz, $CDCl_3$) δ174.2, 158.4, 158.0, 147.8, 146.2, 143.8, 139.9, 139.8, 138.1, 134.1, 130.2, 129.6, 129.1, 128.3, 128.2, 128.0, 127.6, 127.4, 127.3, 126.9, 124.1, 123.9, 123.7, 121.9, 87.3, 77.9, 71.9, 69.7, 65.3, 57.0, 29.5, 29.1, 29.0, 25.6, 25.2, 24.9, 23.3, 22.8, 21.7; HRMS (m/z): $(M+H)^+$ calcd. for $C_{45}H_{50}N_5O_3S^+$, 740.3629; found, 740.3629.

**(3*R*,7a*S*)-2-(2,6-Diisopropylphenyl)-3-(6-((2*S*,4*R*,5*R*)-4,5-diphenyl-1-tosylimidazolidin-2-yl)pyridin-2-yl)-6-hydroxyhexahydro-1*H*-pyrrolo[1,2-*c*]imidazol-1-one (L2)**

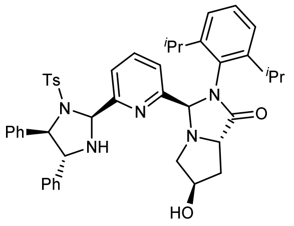


Prepared according to supplementary method B - In a round-bottomed flask containing a stir bar, compound **S3b** (391.0 mg, 1.0 mmol), (*R*,*R*)-TsDPEN **S4a** (366.0 mg, 1.0 mmol), AcOH (1.5 mmol, 74.0 μL), and dichloromethane (10.0 mL) were added. Then, the reaction was stirred at 30 °C under $N_2$ for 6 h. After that, the reaction mixture was quenched by aqueous $NaHCO_3$. The organic layer was extracted with dichloromethane for 3 times, and the collected organic layer was dried over $Na_2SO_4$. After removing the solvent under reduced pressure, the resulting residue was purified by silica gel column chromatography to give **L2** as a white solid (535.3 mg, 71% yield). Further purification can be achieved by recrystallization

(recrystallization solvent: Pet/$CH_2Cl_2$) to obtain the chiral tridentate ligand **L2** as a white solid (490.1 mg, 65% yield); $R_f$ = 0.24 ($CH_2Cl_2$/MeOH, 25/1, v/v); m.p.: 136.5-138.7 °C. $[\alpha]_D^{25.5}$ = -127.0 (*c* = 0.2, in $CHCl_3$). $^1H$ NMR (600 MHz, $CDCl_3$) δ 7.86 – 7.81 (m, 2H), 7.65 (d, *J* = 7.2 Hz, 1H), 7.54 (dd, *J* = 6.4, 1.6 Hz, 2H), 7.21 – 7.05 (m, 11H), 6.95 – 6.90 (m, 2H), 6.86 (dd, *J* = 7.2, 2.0 Hz, 2H), 5.63 (s, 1H), 5.33 (s, 1H), 4.66 (br, 1H), 4.58 (t, *J* = 7.2 Hz, 1H), 4.47 (d, *J* = 6.4 Hz, 1H), 4.16 (d, *J* = 6.6 Hz,1H), 3.47 (m, 1H), 3.18 (dd, *J* = 11.2, 4.4Hz, 1H), 2.90 (quint, *J* = 6.8 Hz, 1H), 2.82 (br, 1H), 2.42 (s, 3H), 2.40–2.29 (m, 3H), 2.16 (quint, *J* = 6.8 Hz, 1H), 1.12 (d, *J* = 7.2 Hz, 3H), 1.08 (d, *J* = 7.2 Hz, 3H), 0.98 (d, *J* = 6.6 Hz, 3H), 0.22 (d, *J* = 6.6 Hz, 3H); $^{13}C$ NMR (150 MHz, $CDCl_3$) δ 174.0, 158.2, 157.7, 147.9, 146.1, 143.9, 139.8, 139.6, 138.2, 134.0, 129.8, 129.7, 129.2, 128.34, 128.26, 128.0, 127.6, 127.4, 127.3, 126.8, 124.2, 123.7, 121.9, 86.7, 78.0, 72.3, 71.9, 69.7, 63.7, 63.6, 37.8, 29.2, 28.9, 25.1, 25.0, 23.3, 22.8, 21.7; HRMS (m/z): $(M+H)^+$ calcd. for $C_{45}H_{50}N_5O_4S^+$, 756.3578; found, 756.3580.

**(3*R*,7a*S*)-2-(2,6-Diethylphenyl)-3-(6-((2*S*,4*R*,5*R*)-4,5-diphenyl-1-tosylimidazolidin-2-yl)pyridin-2-yl)hexahydro-1*H*-pyrrolo[1,2-*c*]imidazol-1-one (L3)**

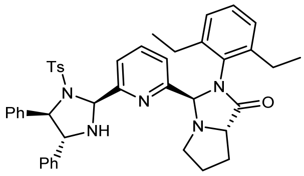


Prepared according to supplementary method B - In a round-bottomed flask containing a stir bar, compound **S3c** (363.2 mg, 1.0 mmol), (*R*,*R*)-TsDPEN **S4a** (366.0 mg, 1.0 mmol), AcOH (1.5 mmol, 74.0 μL), and dichloromethane (10.0 mL) were added. Then, the reaction was stirred at 30 °C under $N_2$ for 6 h. After that, the reaction mixture was quenched by aqueous $NaHCO_3$. The organic layer was extracted with dichloromethane for 3 times, and the collected organic layer was dried over $Na_2SO_4$. After removing the solvent under reduced pressure, the resulting residue was purified by silica gel column chromatography to give **L3** as a white solid (583.0 mg, 82% yield). Further purification can be achieved by recrystallization

(recrystallization solvent: Pet/EtOAc) to obtain the chiral tridentate ligand **L3** as a white solid (533.2 mg, 75% yield); $R_f$ = 0.56 ($CH_2Cl_2$/MeOH, 30/1, v/v); m.p.: 118.2-121.7 °C. $[\alpha]_D^{25.5}$ = -76.0 (*c* = 0.2, in $CHCl_3$). $^1H$ NMR (400 MHz, $CDCl_3$) δ 7.85 (t, *J* = 7.6 Hz, 1H), 7.71 (t, *J* = 8.4 Hz, 2H), 7.41–7.35 (m, 2H), 7.25–7.20 (m, 5H), 7.19–7.13 (m, 3H), 7.06 (d, *J* = 8.0 Hz, 2H), 6.98 (dd, *J* = 7.6, 1.6 Hz, 2H), 6.95 (d, *J* = 7.6 Hz, 1H), 6.88 (dd, *J* = 8.0, 1.6 Hz, 1H), 6.76 (dd, *J* = 7.6, 1.6 Hz, 1H), 5.53 (s, 1H), 5.40 (s, 1H), 4.63 (d, *J* = 4.8 Hz, 1H), 4.28 (dd, *J* = 8.8, 5.2 Hz, 1H), 4.11 (d, *J* = 4.4 Hz,1H), 3.51–3.46 (m, 1H), 3.13–3.07 (m, 1H), 2.66–2.57 (m, 2H), 2.54–2.45 (m, 1H), 2.37 (s, 3H), 2.34–2.18 (m, 2H), 2.04–1.97 (m, 2H), 1.85–1.76 (m,1H), 1.25 (q, *J* = 7.6 Hz, 1H), 1.14 (t, *J* = 7.6 Hz, 3H), 0.74 (t, *J* = 7.2 Hz, 3H); $^{13}C$ NMR (100 MHz, $CDCl_3$) δ 173.9, 158.2, 157.4, 143.5, 142.9, 141.3, 140.7, 140.2, 138.0, 134.3, 132.1, 129.4 128.4, 128.3, 128.2, 127.7, 127.4, 127.3, 127.2, 126.6, 126.4, 126.2, 123.9, 121.4, 85.5, 78.4, 71.3, 69.5, 65.1, 57.0, 29.1, 25.6, 25.0, 22.9, 21.6, 14.8, 13.6; HRMS (m/z): $(M+H)^+$ calcd. for $C_{43}H_{46}N_5O_3S^+$, 712.3316; found, 712.3315.

**(3*R*,7a*S*)-3-(6-((2*S*,4*R*,5*R*)-4,5-Diphenyl-1-tosylimidazolidin-2-yl)pyridin-2-yl) -2-phenylhexahydro-1*H*-pyrrolo[1,2-*c*]imidazol-1-one (L4)**

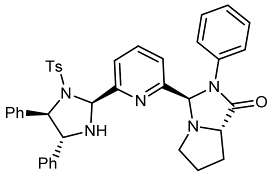


Prepared according to supplementary method B - In a round-bottomed flask containing a stir bar, compound **S3d** (307.1 mg, 1.0 mmol), (*R*,*R*)-TsDPEN **S4a** (366.0 mg, 1.0 mmol), AcOH (1.5 mmol, 74.0 μL), and dichloromethane (10.0 mL) were added. Then, the reaction was stirred at 30 °C under $N_2$ for 6 h. After that, the reaction mixture was quenched by aqueous $NaHCO_3$. The organic layer was extracted with dichloromethane for 3 times, and the collected organic layer was dried over $Na_2SO_4$. After removing the solvent under reduced pressure, the resulting residue was purified by silica gel column chromatography to give **L4** as a white solid

(563.3 mg, 86% yield). Further purification can be achieved by recrystallization (recrystallization solvent: $Et_2O$) to obtain the chiral tridentate ligand **L4** as a white solid (537.1 mg, 82% yield); $R_f$ = 0.46 ($CH_2Cl_2$/MeOH, 25/1, v/v); m.p.: 85.7-87.8 °C. $[\alpha]_D^{25.5}$ = -102.0 (*c* = 0.2, in $CHCl_3$); HPLC CHIRALPAK OD-H, n-hexane/2-propanol=75/25, flow rate = 0.8 mL /min, λ = 256 nm, retention time: 13.841 min (major). $^1H$ NMR (600 MHz, $CDCl_3$) δ 7.95 (d, *J* = 7.2 Hz, 1H), 7.85 (t, *J* = 7.2 Hz, 1H), 7.64–7.60 (m, 2H), 7.39 (d, *J* = 7.8 Hz, 1H), 7.36–7.33 (m, 2H), 7.23–7.12 (m, 8H), 7.11–7.07 (m, 2H), 7.05–7.01 (m, 2H), 6.96–6.92 (m, 1H), 6.89–6.86 (m, 2H), 5.88 (s, 1H), 5.76 (s, 1H), 4.54 (d, *J* = 6.6 Hz, 1H), 4.15 (dd, *J* = 8.4, 4.8 Hz,1H), 4.10 (d, *J* = 6.6 Hz, 1H), 3.50 (br, 1H), 3.45–3.43 (m, 1H), 2.96–2.92 (m, 1H), 2.44 (s, 3H), 2.25–2.15 (m, 2H), 1.93–1.85 (m, 2H); $^{13}C$ NMR (150 MHz, $CDCl_3$) δ 174.8, 159.1, 157.8, 143.9, 139.4, 139.1, 138.5, 137.5, 134.2, 129.7, 129.0, 128.4, 128.3, 128.1, 127.3, 127.1, 125.1, 12) 3.7, 121.4, 120.6, 84.3, 77.7, 72.1, 69.6, 65.0, 56.5, 28.0, 25.0, 21.7; HRMS (m/z): $(M+Na)^+$ calcd. for $C_{39}H_{37}N_5NaO_3S^+$, 678.2509; found, 678.2509.

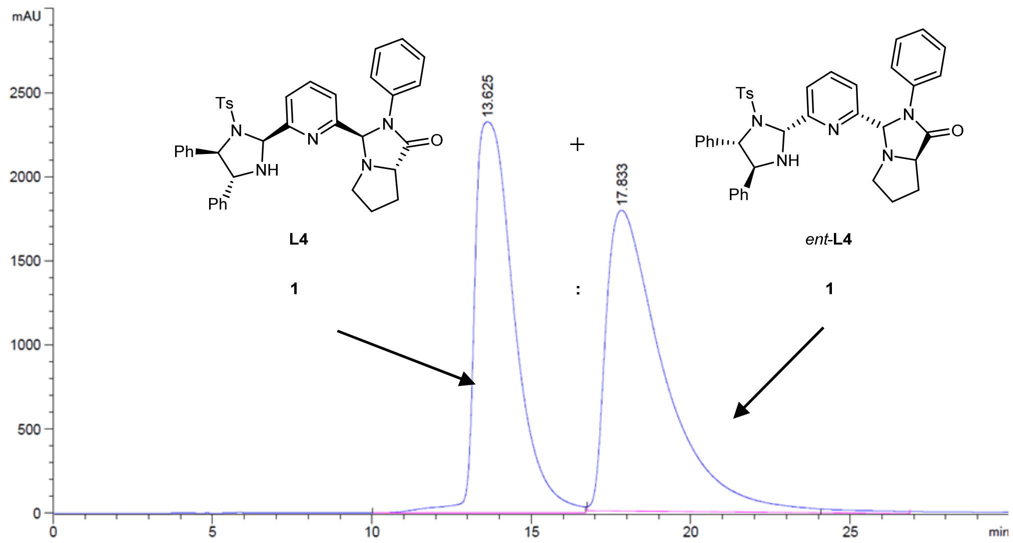


| Peak # | RetTime [min] | Type | Width [min] | Area [mAU*s] | Height [mAU] | Area % |
|---|---|---|---|---|---|---|
| 1 | 13.625 | BV | 1.0185 | 2.00002e5 | 2324.15918 | 48.2294 |
| 2 | 17.833 | MM | 2.0868 | 2.23468e5 | 1784.75830 | 51.7706 |

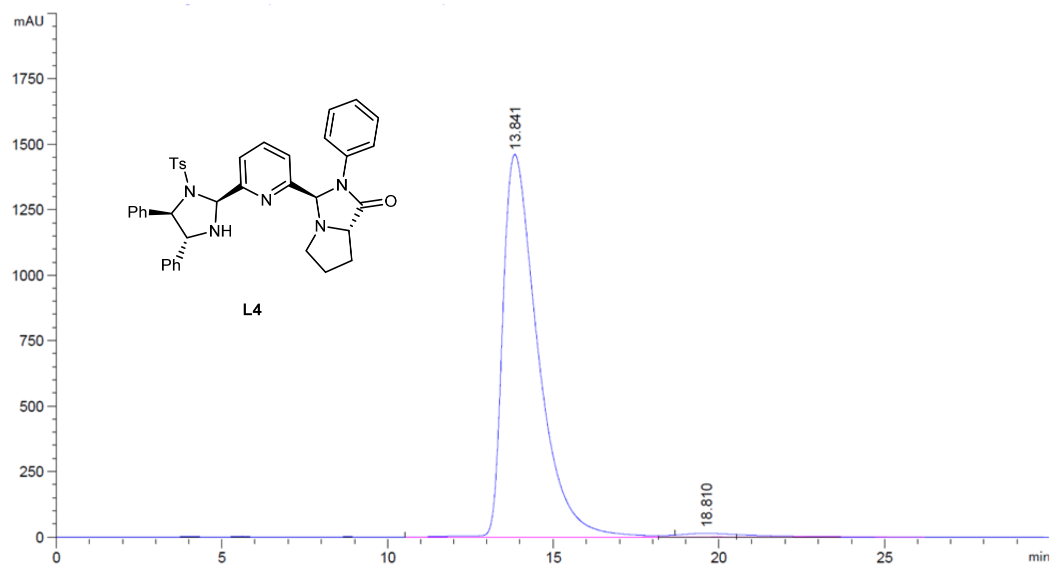


| Peak # | RetTime [min] | Type | Width [min] | Area [mAU*s] | Height [mAU] | Area % |
|---|---|---|---|---|---|---|
| 1 | 13.841 | BV | 1.0841 | 1.07125e5 | 1461.41638 | 99.4240 |
| 2 | 18.810 | MM | 0.9525 | 620.60492 | 7.70442 | 0.5760 |

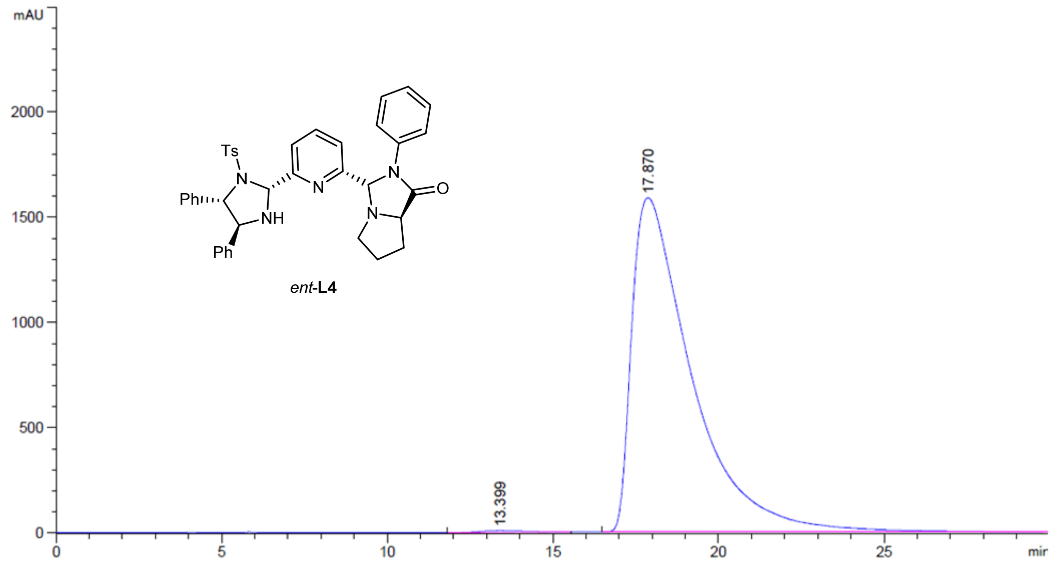


| Peak # | RetTime [min] | Type | Width [min] | Area [mAU*s] | Height [mAU] | Area % |
|---|---|---|---|---|---|---|
| 1 | 13.399 | BB | 1.1550 | 814.47473 | 8.30001 | 0.4048 |
| 2 | 17.870 | BB | 1.7562 | 2.00412e5 | 1588.48999 | 99.5952 |

**Supplementary Fig. 1** HPLC spectra of **L4**

**(3*R*,7a*S*)-3-(6-((2*S*,4*R*,5*R*)-4,5-Diphenyl-1-tosylimidazolidin-2-yl)pyridin-2-yl)-2-(naphthalen-1-yl)hexahydro-1*H*-pyrrolo[1,2-*c*]imidazol-1-one (L5)**

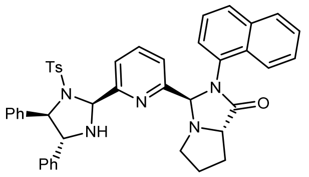


Prepared according to supplementary method B - In a round-bottomed flask containing a stir bar, compound **S3e** (357.1 mg, 1.0 mmol), (*R*,*R*)-TsDPEN **S4a** (366.0 mg, 1.0 mmol), AcOH (1.5 mmol, 74.0 μL), and dichloromethane (10.0 mL) were added. Then, the reaction was stirred at 30 $^oC$ under $N_2$ for 6 h. After that, the reaction mixture was quenched by aqueous $NaHCO_3$. The organic layer was extracted with dichloromethane for 3 times, and the collected organic layer was dried over $Na_2SO_4$. After removing the solvent under reduced pressure, the resulting residue was purified by silica gel column chromatography to give **L5** as a white solid (493.5 mg, 70% yield). Further purification can be achieved by recrystallization (recrystallization solvent: $Et_2O$) to obtain the chiral tridentate ligand **L5** as a white solid (430.1 mg, 61% yield); $R_f$ = 0.34 ($CH_2Cl_2$/MeOH, 30/1, v/v); m.p.: 1737-176.7 $^oC$. $[\alpha]_D^{25.5}$ = -78.0 (*c* = 0.2, in $CHCl_3$). $^1H$ NMR (400 MHz, $CDCl_3$) δ 7.88 (d, *J* = 7.2 Hz, 1H), 7.79 (t, *J* = 8.0 Hz, 1H), 7.75–7.72 (m, 1H), 7.68–7.55 (m, 4H), 7.43–7.34 (m, 3H), 7.23–7.14 (m, 8H), 7.10–7.06 (m, 3H), 6.90–6.78 (m, 3H), 5.70 (br, 1H), 5.64 (s, 1H), 4.55 (d, *J* = 6.8 Hz, 1H), 4.45 (dd, *J* = 8.8, 5.6 Hz, 1H), 4.06 (t, *J* = 6.4 Hz, 1H), 3.59–3.53 (m, 1H), 3.29–3.19 (m,1H), 3.08 (br, 1H), 2.44 (s, 3H), 2.35–2.28 (m, 2H), 2.10–2.02 (m, 2H); $^{13}C$ NMR (100 MHz, $CDCl_3$) δ 175.3, 158.9, 158.1, 143.9, 139.8, 139.2, 138.2, 134.5, 134.1, 132.5, 130.2, 129.7, 128.7, 128.6, 128.5, 128.3, 128.1, 127.7, 127.6, 127.2, 127.0, 126.9, 126.3, 125.5, 123.8, 122.1, 121.3, 86.8, 77.9, 71.9, 69.6, 65.2, 57.0, 28.6, 25.5, 21.7; HRMS (m/z): $(M+H)^+$ calcd. for $C_{43}H_{40}N_5O_3S^+$, 706.2846; found, 706.2839.

**(3*R*,7a*S*)-2-benzyl-3-(6-((2*S*,4*R*,5*R*)-4,5-Diphenyl-1-tosylimidazolidin-2-yl) pyridin-2-yl)hexahydro-1*H*-pyrrolo[1,2-*c*]imidazol-1-one (L6)**

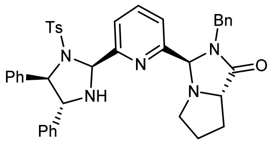


Prepared according to supplementary method B - In a round-bottomed flask containing a stir bar, compound **S3f** (192.6 mg, 0.6 mmol), (*R*,*R*)-TsDPEN **S4a** (219.6 mg, 0.6 mmol), AcOH (1.0 mmol, 60.0 μL), and dichloromethane (8.0 mL) were added. Then, the reaction was stirred at 30 °C under $N_2$ for 6 h. After that, the reaction mixture was quenched by aqueous $NaHCO_3$. The organic layer was extracted with dichloromethane for 3 times, and the collected organic layer was dried over $Na_2SO_4$. After removing the solvent under reduced pressure, the resulting residue was purified by silica gel column chromatography to give **L6** as a yellow oil (563.3 mg, 86% yield). Further purification can be achieved by recrystallization (recrystallization solvent: Pet/EtOAc) to obtain the chiral tridentate ligand **L6** as a yellow solid (197.0 mg, 49% yield), $R_f$ = 0.52 ($CH_2Cl_2$/MeOH, 25/1, v/v); m.p.: 159.6-163.3 °C. $[\alpha]_D^{25.5}$ = -102.0. $^1H$ NMR (400 MHz, $CDCl_3$) δ 7.97 (d, *J* = 7.6 Hz, 1H), 7.88 (t, *J* = 7.6 Hz, 1H), 7.62 (dd, *J* = 6.4, 1.6 Hz, 2H), 7.29 (d, *J* = 7.6 Hz, 1H), 7.24–7.18 (m, 11H), 7.14–7.10 (m, 2H), 7.07–7.05 (m, 2H), 7.01–6.98 (m, 2H), 5.97 (s, 1H), 4.91 (d, *J* = 15.2 Hz, 1H), 4.86 (s, 1H), 4.66 (d, *J* = 6.4 Hz, 1H), 4.39 (d, *J* = 6.0 Hz, 1H), 4.08 (dd, *J* = 9.2, 5.2 Hz, 1H), 3.65 (br, 1H), 3.39 (d, *J* = 14.8 Hz, 1H), 3.20–3.15 (m, 1H), 2.66–2.60 (m, 1H), 2.43 (s, 3H), 2.21–2.06 (m, 2H), 1.84–1.73 (m, 2H); $^{13}C$ NMR (100 MHz, $CDCl_3$) δ 174.9, 158.9, 158.0, 144.0, 139.8, 139.4, 138.5, 136.1, 134.5, 129.7, 128.8, 128.6, 128.4, 128.2, 128.1, 127.81, 127.78, 127.7, 127.2, 127.1, 124.0, 121.4, 82.8, 78.0, 71.8, 70.0, 64.6, 58.5, 56.5, 44.4, 28.3, 25.1, 21.7, 18.5; HRMS (m/z): $(M+H)^+$ calcd. for $C_{40}H_{40}N_5O_3S^+$, 670.2846; found, 670.2845.

**(3*R*,7a*S*)-3-(6-((2*R*,4*S*,5*S*)-4,5-Diphenyl-1-tosylimidazolidin-2-yl)pyridin-2-yl)-2-phenylhexahydro-1*H*-pyrrolo[1,2-*c*]imidazol-1-one (L7)**

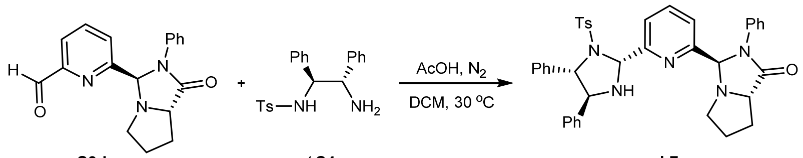


Prepared according to supplementary method B -In a round-bottomed flask containing a stir bar, compound **S3d** (183.0 mg, 0.6 mmol), (*S*,*S*)-TsDPEN *ent*-**S4a** (219.6 mg, 0.6 mmol), AcOH (1.0 mmol, 60.0 μL), and dichloromethane (10.0 mL) were added. Then, the reaction was stirred at 30 °C under $N_2$ for 6 h. After that, the reaction mixture was quenched by aqueous $NaHCO_3$. The organic layer was extracted with dichloromethane for 3 times, and the collected organic layer was dried over $Na_2SO_4$. After removing the solvent under reduced pressure, the resulting residue was purified by silica gel column chromatography to give **L7** as a white solid (314.4 mg, 80% yield). Further purification can be achieved by recrystallization (recrystallization solvent: $Et_2O$) to obtain the chiral tridentate ligand **L7** as a white solid (286.9 mg, 73% yield); $R_f$ = 0.46 ($CH_2Cl_2$/MeOH, 25/1, v/v); m.p.: 184.4-187.0 °C. $[\alpha]_D^{25.5}$ = -166.0 (*c* = 0.2, in $CHCl_3$). $^1H$ NMR (600 MHz, $CDCl_3$) δ 7.96 (d, *J* = 7.8 Hz, 1H), 7.86 (t, *J* = 7.8 Hz, 1H), 7.64 (d, *J* = 7.8 Hz, 2H), 7.42 (d, *J* = 7.8 Hz, 2H), 7.38 (d, *J* = 7.8 Hz, 1H), 7.24–7.18 (m, 8H), 7.17–7.14 (t, *J* = 7.8 Hz, 2H), 7.09 (d, *J* = 6.6 Hz, 2H), 7.00 (t, *J* = 7.8 Hz, 1H), 6.95 (d, *J* = 7.2 Hz, 2H), 5.90 (s, 1H), 5.74 (s, 1H), 4.59 (d, *J* = 6.6 Hz, 1H), 4.24 (d, *J* = 6.6 Hz,1H), 3.99 (t, *J* = 6.6 Hz, 1H), 3.53 (br, 1H), 3.44–3.41 (m, 1H), 2.93–2.89 (m, 1H), 2.44 (s, 3H), 2.17–2.14 (m, 2H), 1.89–1.86 (m, 2H); $^{13}C$ NMR (150 MHz, $CDCl_3$) δ 174.9, 159.2, 157.8, 144.0, 139.5, 139.3, 138.5, 137.6, 134.2, 129.7, 129.1, 128.5, 128.4, 128.1, 127.8, 127.6, 127.2, 127.1, 125.2, 123.7, 121.2, 120.5, 84.4, 77.8, 72.1, 69.7, 64.8, 56.5, 27.9, 25.0, 21.7; HRMS (m/z): $(M+Na)^+$ calcd. for $C_{39}H_{37}N_5NaO_3S^+$, 678.2509; found, 678.2502.

**2,6-Bis((2*S*,4*R*,5*R*)-4,5-Diphenyl-1-tosylimidazolidin-2-yl)pyridine (L8)**

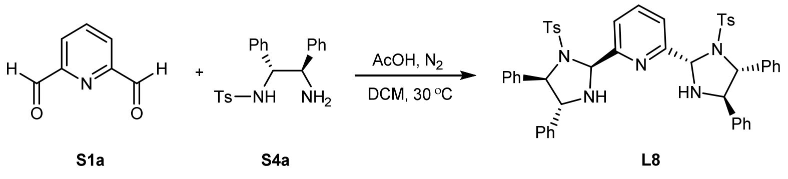


Prepared according to supplementary method B - In a round-bottomed flask containing a stir bar, pyridine-2,6-dicarbaldehyde **S1a** (0.5 mmol, 67.5 mg) and **S4a** (366.0 mg, 1.0 mmol), AcOH (1.0 mmol, 60.0 μL), and dichloromethane (8.0 mL) were added. Then, the reaction was stirred at 30 °C under $N_2$ for 6 h. After that, the reaction mixture was quenched by aqueous $NaHCO_3$. The organic layer was extracted with dichloromethane for 3 times, and the collected organic layer was dried over $Na_2SO_4$. After removing the solvent under reduced pressure, the resulting residue was purified by silica gel column chromatography (Pet/EtOAc, 2:1, v/v) to give the **L8** as a white solid (257.0 mg, 62% yield); $R_f$ = 0.41 (Pet/EtOAc, 2:1, v/v); m.p.: 185.1-186.9 °C. $[\alpha]_D^{25.5}$ = -75.2 (*c* = 0.2, in $CHCl_3$). $^1$H NMR (400 MHz, $CDCl_3$) δ 8.04–7.96 (m, 3H), 7.53 (dd, *J* = 8.0 Hz, 4H), 7.21–7.10 (m, 20H), 7.00 (d, *J* = 6.8 Hz, 4H), 5.84 (s, 2H), 4.72 (d, *J* = 5.2 Hz, 2H), 4.27 (d, *J* = 5.2 Hz, 2H), 3.55 (s, 2H), 2.40 (s, 6H); $^{13}$C NMR (100 MHz, $CDCl_3$) δ 158.1, 143.8, 139.8, 139.3, 138.2, 134.4, 129.6, 128.6, 128.4, 127.9, 127.54, 127.51, 127.1, 126.8, 124.2, 78.5, 71.6, 69.4, 21.7; HRMS (m/z): $(M+Na)^+$ calcd. for $C_{49}H_{45}N_5NaO_4S_2^+$, 854.2805; found, 854.2803.

**(3*R*,7a*S*)-3-(6-((1*S*,3a*R*)-3-Oxo-2-phenyloctahydrocyclopenta[*c*]pyrrol-1-yl) pyridin-2-yl)-2-phenylhexahydro-1*H*-pyrrolo[1,2-*c*]imidazol-1-one (L9)**

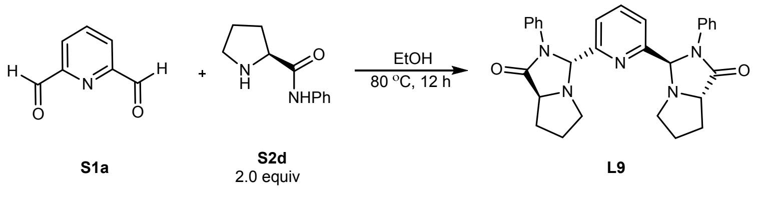


Pyridine-2,6-dicarbaldehyde **S1a** (2.0 mmol) and *L*-prolinamide **S2d** (4.2 mmol) were added into a pressure-resistant test tube. Then, anhydrous ethanol (8.0 mL) was added and the reaction was heated with stirring at 80 °C (oil bath as the heat

source) for 12 h. After that, the reaction mixture was concentrated in vacuo to remove ethanol. The residue was purified by flash column chromatography (Pet/EtOAc, 10/1-1:2, v/v) to give the ligand **L9**. Further purification was achieved by recrystallization (recrystallization solvent: Pet/EtOAc) to obtain the chiral tridentate ligand **L9** as a white solid (710.4 mg, 74% yield), $R_f$ = 0.21 (EtOAc); m.p.: 185.1-186.9 °C. $[\alpha]_D^{25.5}$ = -108.0 (*c* = 0.2, in $CHCl_3$). $^1H$ NMR (600 MHz, $CDCl_3$) δ 7.65 (t, *J* = 7.8 Hz, 1H), 7.39–7.36 (m, 4H), 7.22 (s, 1H), 7.21–7.16 (m, 5H), 7.06–7.02 (m, *J* = 7.2 Hz, 2H), 5.68 (s, 2H), 4.03 (dd, *J* = 7.8, 5.4 Hz, 2H), 3.40–3.35 (m, 2H), 2.94–2.89 (m, 2H), 2.19–2.15 (m, 4H), 1.91–1.81 (m, 4H); $^{13}C$ NMR (100 MHz, $CDCl_3$) δ 175.1, 158.4, 138.7, 137.5, 129.0, 125.1, 121.3, 120.7, 84.4, 64.9, 56.5, 27.9, 24.9; HRMS (m/z): $(M+H)^+$ calcd. for $C_{29}H_{30}N_5O_2^+$, 480.2394; found, 480.2399.

**(3*R*,7a*S*)-3-(6-((2*S*,4*R*,5*R*)-1-Methyl-4,5-diphenyl-3-tosylimidazolidin-2-yl) pyridin-2-yl)-2-phenylhexahydro-1*H*-pyrrolo[1,2-*c*]imidazol-1-one (L10)**

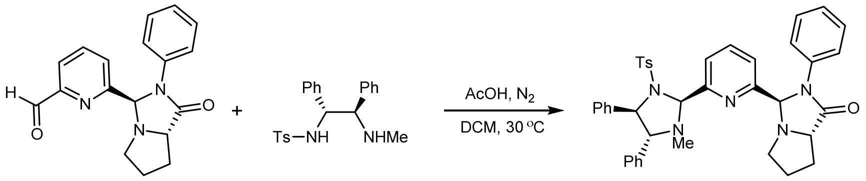


**S3d** **S4b** **L10**

In a round-bottomed flask containing a stir bar, compound **S3a** (183.0 mg, 0.6 mmol), **S4b** (230.0 mg, 0.6 mmol), AcOH (1.0 mmol, 60.0 μL), and dichloromethane (8.0 mL) were added. Then, the reaction was stirred at 30 °C under $N_2$ for 6 h. After that, the reaction mixture was quenched by aqueous $NaHCO_3$. The organic layer was extracted with dichloromethane for 3 times, and the collected organic layer was dried over $Na_2SO_4$. After removing the solvent under reduced pressure, the resulting residue was purified by silica gel column chromatography (Pet/EtOAc, 10/1-1:2, v/v) to give the ligand **L10** as a white solid. Further purification can be achieved by recrystallization (recrystallization solvent: $Et_2O$) to obtain the chiral tridentate ligand **L10** as a white solid (156.5 mg, 39% yield); $R_f$ = 0.52 (Pet/EtOAc/$Et_3N$, 1/1/0.001, v/v); m.p.: 145.1-147.9 °C. $[\alpha]_D^{25.5}$ = -113.6 (*c* = 0.2, in

$CHCl_3$). $^1H$ NMR (400 MHz, $CDCl_3$) δ 7.91 (d, *J* = 8.0 Hz, 1H), 7.84 (t, *J* = 7.6 Hz, 1H), 7.45 (d, *J* = 8.0 Hz, 2H), 7.40 (d, *J* = 7.6 Hz, 1H), 7.27–7.24 (m, 3H), 7.20–7.14 (m, 5H), 7.01 (t, *J* = 7.6 Hz, 2H), 6.96 (t, *J* = 7.6 Hz, 1H), 6.90–6.82 (m, 6H), 5.73 (s, 1H), 5.08 (s, 1H), 4.87 (d, *J* = 8.0 Hz, 1H), 4.34 (dd, *J* = 8.4, 4.8 Hz, 1H), 3.72 (d, *J* = 8.0 Hz, 1H), 3.51–3.48 (m, 1H), 3.02–2.98 (m, 1H), 2.31–2.57 (m, 5H), 2.00 (s, 3H), 1.98–1.89 (m, 2H); $^{13}C$ NMR (100 MHz, $CDCl_3$) δ 175.9, 159.9, 156.6, 142.0, 138.1, 137.9, 137.8, 137.1, 129.5, 128.92, 128.87, 128.7, 128.3, 128.0, 127.8, 127.7, 126.8, 125.3, 123.8, 121.9, 121.3, 86.5, 84.6, 78.2, 72.5, 64.9, 56.2, 36.4, 27.8, 25.0, 21.5; HRMS (m/z): $(M+H)^+$ calcd. for $C_{40}H_{40}N_5O_3S^+$, 670.2846; found, 670.2845.

**(3*R*,7*aS*)-3-(3-((2*S*,4*R*,5*R*)-4,5-Diphenyl-1-tosylimidazolidin-2-yl)phenyl)-2-phenylhexahydro-1*H*-pyrrolo[1,2-*c*]imidazol-1-one (L11)**

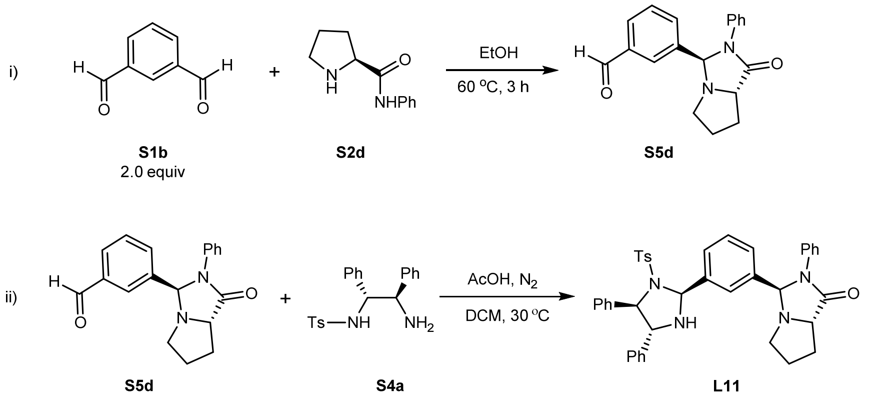


Isophthalaldehyde **S1b** (670.0 mg, 5.0 mmol) and *L*-prolinamide **S2d** (475.0 mg, 2.5 mmol) were added to a pressure-resistant test tube with anhydrous ethanol (10.0 mL). Then, the reaction was heated and stirred at 60 °C (oil bath as the heat source) for 3 h. After that, the reaction mixture was concentrated in vacuo to remove ethanol. The residue was purified by flash column chromatography (Pet/EtOAc, 10/1-2:1, v/v) to give **S5d** as a white solid (419.4 mg, 55% yield). Further purification could be achieved by recrystallization (recrystallization solvent: Pet/ EtOAc) to give **S5d** as a white solid (350.8mg, 46% yield).Then, in a round-bottomed flask containing a stir bar, compound **S5d** (91.2 mg, 0.3 mmol), **S4a** (110.0 mg, 0.3 mmol), AcOH (0.5 mmol, 30.0 μL), and dichloromethane (5.0 mL) were added. Then, the reaction was stirred at 30 °C under $N_2$

for 6 h. After that, the reaction mixture was quenched by aqueous $NaHCO_3$. The organic layer was extracted with dichloromethane for 3 times, and the collected organic layer was dried over $Na_2SO_4$. After removing the solvent under reduced pressure, the resulting residue was purified by silica gel column chromatography (Pet/EtOAc, 10/1-1:2, v/v) to give the ligand **L11** as a white solid. Further purification can be achieved by recrystallization (recrystallization solvent: Pet/EtOAc) to obtain the chiral tridentate ligand **L11** as a white solid (84.5 mg, 43% yield); $R_f$ = 0.39 ($CH_2Cl_2$/MeOH, 25/1, v/v); m.p.: 135.7-137.9 °C. $[\alpha]_D^{25.5}$ = -151.2 (*c* = 0.2, in $CHCl_3$). $^1$H NMR (400 MHz, $CDCl_3$) δ 7.78 (m, 2H), 7.71 (dd, *J* = 6.8, 2.0 Hz, 2H), 7.44 (m, 3H), 7.33–7.27 (m, 3H), 7.25–7.03 (m, 10H), 6.83 (dd, *J* = 7.2, 2.0 Hz, 2H), 6.76 (dd, *J* = 8.0, 1.2 Hz, 2H), 6.18 (s, 1H), 5.71 (s, 1H), 4.54 (d, *J* = 8.0 Hz, 1H), 4.06 (t, *J* = 6.8 Hz, 1H), 3.99 (d, *J* = 8.0 Hz, 1H), 3.45 (quint, *J* = 4.8 Hz, 1H), 2.91 (q, *J* = 8.4 Hz, 1H), 2.44 (s, 3H), 2.21 (q, *J* = 7.2 Hz, 2H), 1.96–1.87 (m, 2H); $^{13}$C NMR (100 MHz, $CDCl_3$) δ175.0, 144.4, 141.2, 140.0, 139.2, 137.7, 137.4, 134.7, 130.0, 129.5, 129.3, 129.1, 128.9, 128.4, 128.3, 128.2, 128.0, 127.5, 127.2, 126.9, 125.9, 125.33, 125.31, 121.6, 84.0, 78.7, 71.9, 71.0, 64.6, 56.3, 27.8, 25.0, 21.7; HRMS (m/z): $(M+H)^+$ calcd. for $C_{40}H_{39}N_4O_3S^+$, 655.2737; found, 655.2737.

**6-((4*R*,5*R*)-4,5-Diphenyl-1-tosylimidazolidin-2-yl)picolinaldehyde (L12)**

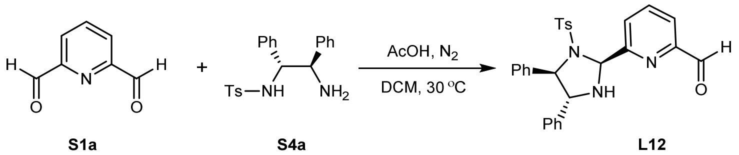


**S1a** **S4a** **L12**

Prepared according to supplementary method B –In a round-bottomed flask containing a stir bar, pyridine-2,6-dicarbaldehyde **S1a** (270.0 mg, 2.0 mmol), **S4a** (366.0 mg, 1.0 mmol), AcOH (1.5 mmol, 74.0 μL), and dichloromethane (10.0 mL) were added. Then, the reaction was stirred at 30 °C under $N_2$ for 6 h. After that, the reaction mixture was quenched by aqueous $NaHCO_3$. The organic layer was extracted with dichloromethane for 3 times, and the collected organic layer was dried over $Na_2SO_4$. After removing the solvent under reduced pressure, the resulting residue

was purified by silica gel column chromatography to obtain the chiral tridentate ligand **L12** as a white solid (227.7 mg, 45% yield); $R_f$ = 0.53 (Pet/EtOAc, 5/1, v/v ); m.p.: 155.1-156.9 °C. $[\alpha]_D^{25.5}$ = -104.8 (*c* = 0.2, in $CHCl_3$). $^1H$ NMR (400 MHz, $CDCl_3$) δ 10.02 (d, *J* = 0.8 Hz, 1H), 8.22 (dd, *J* = 7.6, 1.2 Hz, 1H), 8.06–7.99 (m, 2H), 7.60–7.58 (m, 2H), 7.24–7.19 (m, 10H), 7.04 (dd, *J* = 7.6, 1.6 Hz, 2H), 6.04 (s, 1H), 4.72 (d, *J* = 6.4 Hz, 1H), 4.39 (d, *J* = 6.8 Hz,1H), 3.78 (br, 1H), 2.43 (s, 3H); $^{13}C$ NMR (100 MHz, $CDCl_3$) δ 193.2, 159.8, 152.3, 144.1, 139.4, 138.7, 138.3, 129.7, 128.7, 128.4, 128.3, 128.1, 128.0, 127.8, 127.3, 127.0, 121.4, 78.0, 71.9, 70.1, 21.7; HRMS (m/z): $(M+Na)^+$ calcd. for $C_{28}H_{25}N_3NaO_3S^+$, 506.1509; found, 506.1508.

**6-((3*R*,7*aS*)-1-Oxo-2-phenylhexahydro-1*H*-pyrrolo[1,2-*c*]imidazol-3-yl)picolinaldehyde (L13)**

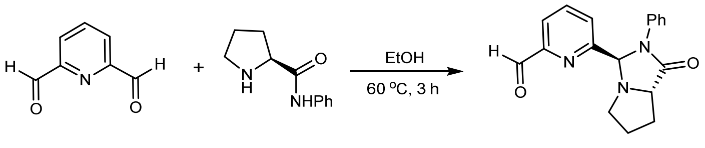


**S1a** 2.0 equiv **S2d** **L13**

Pyridine-2,6-dicarbaldehyde **S1a** (1.35 g, 10.0 mmol) and *L*-prolinamide **S2d** (950.0 mg, 5.0 mmol) were added to a pressure-resistant test tube with anhydrous ethanol (15.0 mL). Then, the reaction was heated and stirred at 60 °C (oil bath as the heat source) for 3 h. After that, the reaction mixture was concentrated in vacuo to remove ethanol. The residue was purified by flash column chromatography (Pet/EtOAc, 20/1-4:1, v/v) to give **S3d** as a white solid (844.1 mg, 55% yield). Further purification could be achieved by recrystallization (recrystallization solvent: Pet/ EtOAc) to give **S3d** as a white solid (706.1mg, 46% yield); $R_f$ = 0.48 (Pet/EtOAc/$Et_3N$, 2/1/0.001, v/v/v); m.p.: 135.1-137.9 °C. $[\alpha]_D^{25.5}$ = -99.0 (*c* = 0.2, in $CHCl_3$). $^1H$ NMR (400 MHz, $CDCl_3$) δ 10.00 (s, 1H), 7.85–7.78 (m, 2H), 7.45–7.42 (m, 3H), 7.25–7.22 (m, 2H), 7.06 (t, *J* = 7.2 Hz, 1H), 5.83 (s, 1H), 4.15 (t, *J* = 6.8 Hz, 1H), 3.49–3.46 (m, 1H), 3.01–2.95 (m, 1H), 2.21 (q, *J* = 6.8 Hz, 2H), 1.92 – 1.84 (m, 2H); $^{13}C$ NMR (100 MHz, $CDCl_3$) δ 193.2, 175.0, 159.3, 153.0, 138.5, 137.2, 129.2, 125.5, 124.6, 121.3, 84.3, 64.9, 56.6, 27.9, 24.9; HRMS

(m/z): $(M+ Na)^+$ calcd. for $C_{18}H_{17}N_3NaO_2^+$, 330.1213; found, 330.1210.

**(3*R*,7*aS*)-2-Phenyl-3-(pyridin-2-yl)hexahydro-1*H*-pyrrolo[1,2-*c*]imidazol-1-one (L14)**

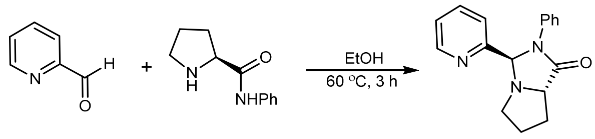


**S1c** **S2d** **L14**

Picolinaldehyde **S1c** (0.6 mmol, 62.4 mg) and *L*- prolinamide **S2d** (0.5 mmol, 95.0 mg) were added to a pressure-resistant test tube with anhydrous ethanol (15.0 mL). Then, the reaction was heated and stirred at 60 °C (oil bath as the heat source) for 3 h. After that, the reaction mixture was concentrated in vacuo to remove ethanol. The residue was purified by flash column chromatography (Pet/EtOAc, 20/1-4:1, v/v) to give **L14** as a white solid (83.7 mg, 60% yield). Further purification could be achieved by recrystallization (recrystallization solvent: Pet/ EtOAc) to obtain **L14** as a white solid (75.1 mg, 54% yield), $R_f$ = 0.46 (Pet/ EtOAc, 3/1, v/v); m.p.: 164.8-166.3 °C. $[\alpha]_D^{25.5}$ = -100.8 (*c* = 0.2, in $CHCl_3$). $^1H$ NMR (400 MHz, $CDCl_3$) δ 8.63 (dd, *J* = 5.6, 2.0 Hz, 1H), 7.65 (td, *J* = 7.6, 2.0 Hz, 1H), 7.50 (d, *J* = 8.0 Hz, 2H), 7.30–7.26 (m, 2H), 7.23–7.20 (m, 2H), 7.10 (t, *J* = 7.2 Hz, 1H), 5.78 (s, 1H), 4.15 (t, *J* = 6.8 Hz, 1H), 3.94 (quint, *J* = 5.2 Hz, 1H), 2.97 (dd, *J* = 17.2, 8.0 Hz, 1H), 2.23 (q, *J* = 7.2 Hz, 2H), 1.91 (m, 2H); $^{13}C$ NMR (100 MHz, $CDCl_3$) δ 175.3, 158.4, 150.3, 137.6, 137.5, 129.2, 125.2, 123.6, 121.0, 120.2, 84.8, 64.9, 56.6, 27.9, 25.0; HRMS (m/z): $(M+ Na)^+$ calcd. for $C_{17}H_{17}N_3NaO^+$, 302.1264; found, 302.1264.

**(3*S*,7*aR*)-3-(6-((2*R*,4*S*,5*S*)-4,5-Diphenyl-1-tosylimidazolidin-2-yl)pyridin-2-yl)-2-phenylhexahydro-1*H*-pyrrolo[1,2-*c*]imidazol-1-one (*ent*-L4)**

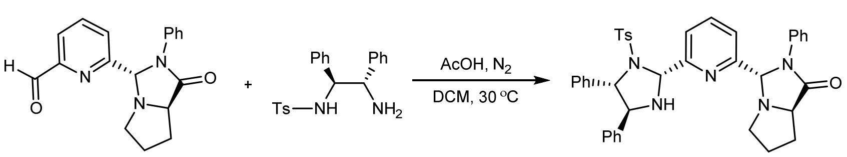


*ent*-**S3d** *ent*-**S4a** *ent*-**L4**

Prepared according to supplementary method B – In a round-bottomed flask containing a stir bar, *ent*-**S3d** (183.0 mg, 0.6 mmol), *ent*-**S4a** (219.6 mg, 0.6 mmol), AcOH (1.0 mmol, 60.0 μL), and dichloromethane (10.0 mL) were added. Then, the reaction

was stirred at 30 °C under $N_2$ for 6 h. After that, the reaction mixture was quenched by aqueous $NaHCO_3$. The organic layer was extracted with dichloromethane for 3 times, and the collected organic layer was dried over $Na_2SO_4$. After removing the solvent under reduced pressure, the resulting residue was purified by silica gel column chromatography to give *ent*-**L4** as a white solid (199.2 mg, 82% yield). Further purification can be achieved by recrystallization (recrystallization solvent: $Et_2O$) to obtain the chiral tridentate ligand *ent*-**L4** as a white solid (135.2 mg, 68% yield); $R_f$ = 0.43 ($CH_2Cl_2$/MeOH, 25/1, v/v); m.p.: 103.2-105.6 °C. $[\alpha]_D^{25.5}$ = -166.4 (*c* = 0.2, in $CHCl_3$). $^1H$ NMR (400 MHz, $CDCl_3$) δ 7.93 (dd, *J* =7.6, 0.8Hz, 1H), 7.85 (t, *J* = 7.6 Hz, 1H), 7.60 (dd, *J* = 6.4, 1.6 Hz, 2H), 7.38 (dd, *J* = 7.6, 1.2 Hz, 1H), 7.35–7.34 (m, 1H), 7.33–7.32 (m, 1H), 7.23–7.07 (m, 10H), 7.03–7.00 (m, 2H), 6.96–6.92 (m, 1H), 6.89–6.86 (m, 2H), 5.87 (s, 1H), 5.75 (s, 1H), 4.54 (d, *J* =6.8, 1H), 4.15–4.08 (m, 2H), 3.49 (br, 1H), 3.47–3.42 (m, 1H), 2.97–2.91 (m, 1H), 2.44 (s, 3H), 2.25–2.14 (m, 2H), 1.93–1.85 (m, 2H); $^{13}C$ NMR (100 MHz, $CDCl_3$) δ 174.9, 159.2, 157.8, 144.0, 139.5, 139.1, 138.5, 137.6, 134.3, 129.7, 129.0, 128.5, 128.3, 128.2, 127.8, 127.6, 127.4, 127.1, 125.2 123.7, 121.4, 120.6, 84.4, 77.8, 72.1, 69.6, 65.0, 56.6, 28.0, 25.1, 21.7; HRMS (m/z): $(M+ Na)^+$ calcd. for $C_{39}H_{37}N_5NaO_3S^+$, 678.2509; found, 678.2509.

# General procedure for optimization study

## **Supplementary Table 1**. Screening of Ni(II) salts

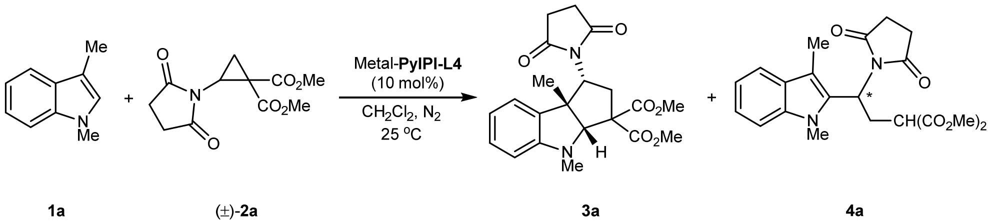


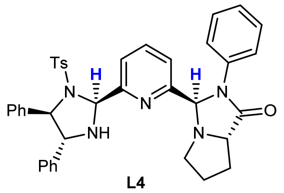


| Entry[a] | Metal | Yield[b] (%) | 3a/4a[c] | dr[c] | ee[d] (%) |
|---|---|---|---|---|---|
| 1 | $Ni(OAc)_2$ | 0 | | | |
| 2 | $Ni(BF_4)_2{\cdot}6H_2O$ | 95 | 78:22 | 8:1 | 82 |
| 3 | $NiCl_2$ | 0 | | | |
| 4 | $NiCl_2{\cdot}6H_2O$ | 0 | | | |
| 5 | $Ni(ClO_4)_2{\cdot}6H_2O$ | 95 | 79:21 | 8:1 | 89 |
| 6 | $Ni(Ts)_2{\cdot}6H_2O$ | 0 | | | |

[a]Unless otherwise noted, reaction conditions were as follows: Metal/**L4** (1:1.2), **1a** (0.1 mmol), **2a** (0.2 mmol) in $CH_2Cl_2$ (2.0 mL) at 25 °C under $N_2$ for 24 h.

[b]The total yield (**3a**+**4a**) was determined by $^1$H NMR spectra of the crude product.

[c]The ratio of **3a/4a** and dr value of **3a** was determined by $^1$H NMR spectra of the crude product.

[d]The ee value of **3a** was determined by chiral HPLC analysis.

**Supplementary Table 2**. Screening of different Metal salts

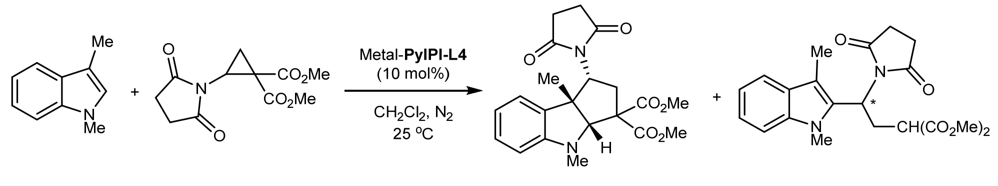


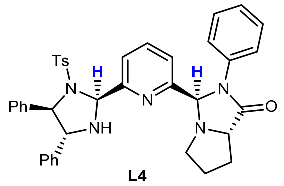


| **Entry**[a] | **Metal** | **Yield**[b] (%) | **3a/4a**[c] | **dr**[c] | **ee**[d] (%) |
|---|---|---|---|---|---|
| 1 | $Cu(OTf)_2$ | 53 | 80:20 | 7:1 | 89 |
| 2 | $Sc(OTf)_3$ | 95 | 74:26 | 5:1 | 0 |
| 3 | $Yb(OTf)_3$ | trace | | | |
| 4 | $Mg(OTf)_2$ | 0 | | | |
| 5 | $Fe(OTf)_3$ | 75 | 70:30 | 7:1 | 63 |
| 6 | $Co(OTf)_2$ | 89 | 79:21 | 8:1 | 90 |
| 7 | $Ni(OTf)_2$ | 98 | 81:19 | 10:1 | 95 |
| 8 | $Zn(OTf)_2$ | 92 | 80:20 | 8:1 | 88 |
| 9 | $MgI_2$ | 0 | | | |
| 10 | $Fe(OTf)_2$ | 90 | 83:17 | 9:1 | 78 |
| 11 | CuOTf | 25 | 86:14 | 7:1 | 95 |

[a]Unless otherwise noted, reaction conditions were as follows: Metal/**L4** (1:1.2), **1a** (0.1 mmol), **2a** (0.2 mmol) in $CH_2Cl_2$ (2.0 mL) at 25 °C under $N_2$ for 24 h.

[b]The total yield (**3a**+**4a**) was determined by $^1H$ NMR spectra of the crude product.

[c]The ratio of **3a/4a** and dr value of **3a** was determined by $^1H$ NMR spectra of the crude product.

[d]The ee value of **3a** was determined by chiral HPLC analysis.

## **Supplementary Table 3**. Screening of solvents

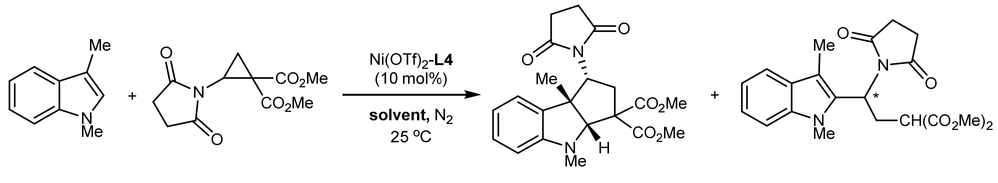


1a (±)-2a 3a 4a

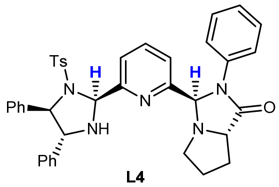

L4

| Entry[a] | solvent | Yield[b] (%) | 3a/4a[c] | dr[c] | ee[d] (%) |
|---|---|---|---|---|---|
| 1 | toluene | 54 | 80:20 | 4:1 | 27 |
| 2 | THF | 14 | 59:41 | 5:1 | 17 |
| 3 | DCE | 95 | 78:22 | 8:1 | 88 |
| 4[e] | DCE/DCM | 95 | 77:23 | 9:1 | 92 |

[a]Unless otherwise noted, reaction conditions were as follows: $Ni(OTf)_2$/**L4** (1:1.2), **1a** (0.1 mmol), **2a** (0.2 mmol) in solvent (2.0 mL) at 25 °C under $N_2$ for 24 h.

[b]The total yield (**3a**+**4a**) was determined by $^1$H NMR spectra of the crude product.

[c]The ratio of **3a/4a** and dr value of **3a** was determined by $^1$H NMR spectra of the crude product.

[d]The ee value of **3a** was determined by chiral HPLC analysis.

[e]DCE/DCM (v/v, 1:1).

**Supplementary Table 4.** Screening of substrate ratio

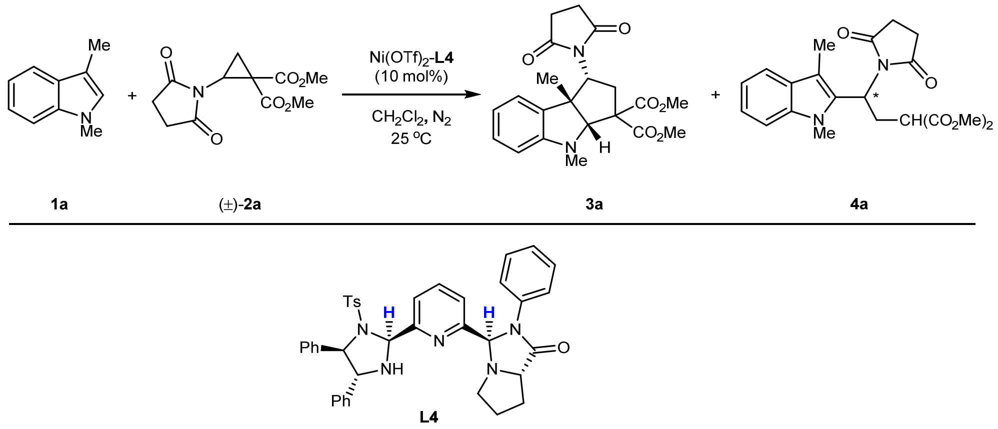


| Entry[a] | 1a:2a | Yield[b] (%) | 3a/4a[c] | dr[c] | ee[d] (%) |
|---|---|---|---|---|---|
| 1 | 1:1 | 68 | 74:26 | 7:1 | 35 |
| 2 | 1:1.5 | 85 | 76:24 | 9:1 | 65 |
| 3 | 1:1.8 | 95 | 78:22 | 9:1 | 85 |
| 4 | 1:2 | 95 | 81:19 | 10:1 | 95 |
| 5 | 1:2.2 | 98 | 82:18 | 10:1 | 98 |
| 6 | 1:2.5 | 98 | 82:18 | 10:1 | 98 |

[a]Unless otherwise noted, reaction conditions were as follows: $Ni(OTf)_2$/**L4** (1:1.2), **1a** (0.1 mmol), in $CH_2Cl_2$ (2.0 mL) at 25 °C under $N_2$ for 24 h.

[b]The total yield (**3a**+**4a**) was determined by $^1$H NMR spectra of the crude product.

[c]The ratio of **3a/4a** and dr value of **3a** was determined by $^1$H NMR spectra of the crude product.

[d]The ee value of **3a** was determined by chiral HPLC analysis.

**Supplementary Table 5.** Screening of temperatures

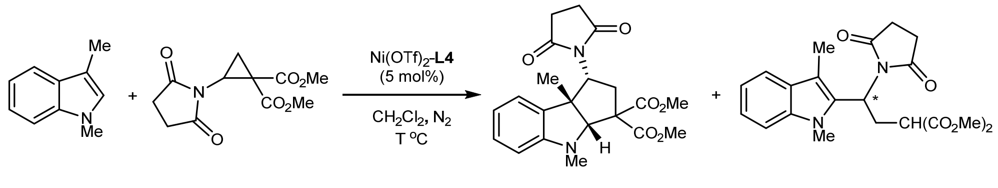


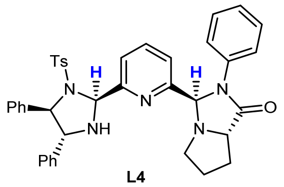


| Entry[a] | T/ °C | Yield[b] (%) | 3a/4a[c] | dr[c] | ee[d] (%) |
|---|---|---|---|---|---|
| 1 | 0 | 74 | 81:19 | 10:1 | 98 |
| 2 | 25 | 98 | 82:18 | 10:1 | 98 |
| 3 | 35 | 98 | 80:20 | 10:1 | 95 |

[a]Unless otherwise noted, reaction conditions were as follows: $Ni(OTf)_2$/**L4** (1:1.2), **1a** (0.1 mmol), **2a** (0.2 mmol) in $CH_2Cl_2$ (2.0 mL) under $N_2$ for 24 h.

[b]The total yield (**3a**+**4a**) was determined by $^1$H NMR spectra of the crude product.

[c]The ratio of **3a/4a** and dr value of **3a** was determined by $^1$H NMR spectra of the crude product.

[d]The ee value of **3a** was determined by chiral HPLC analysis.

## Supplementary method C : enantioselective [3+2] annulation of indoles with cyclopropanes

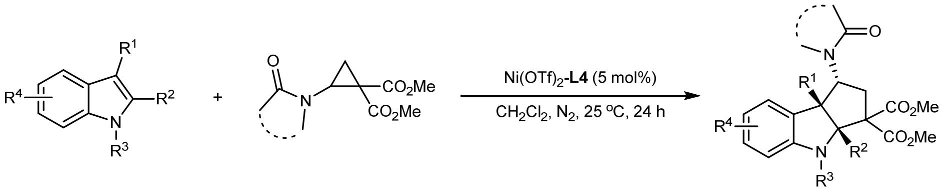


**1a-1m** (±)-**2a-2d** **3a-3p**

In a dry reaction tube, a mixture of $Ni(OTf)_2$ (3.6 mg, 0.01 mmol, 5 mol%), ligand **L4** (7.9 mg, 0.012 mmol, 6 mol%), and aminocyclopropane **2** (0.44 mmol) in DCM (3.0 mL) were stirred at room temperature for 30 minutes under the atmosphere of nitrogen. Then indole substrate **1** (0.2 mmol) in DCM (1.0 mL) was added to the mixture of catalyst via a syringe. After 24 h, the reaction was complete (monitored by TLC). Then, the reaction was filtered through a glass funnel within layer of silica gel (100-200 mesh) and purified by flash column chromatography (Pet/EtOAc, v/v, 10:1-2:1) to give the product **3**.

**(1*R*,3*aR*,8*bR*)-Dimethyl-1-(2,5-dioxopyrrolidin-1-yl)-4,8*b*-dimethyl-1,3*a*,4,8*b*-tetrahydro cyclopenta[*b*]indole-3,3(2*H*)-dicarboxylate (3a)**

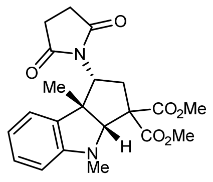


Prepared according to supplementary method C - In a dry reaction tube, a mixture of $Ni(OTf)_2$ (5.4 mg, 0.015 mmol, 5 mol%), ligand **L4** (11.8 mg, 0.018 mmol, 6 mol%), and aminocyclopropane **2a** (168.3 mg, 0.66 mmol) in DCM (4.5 mL) were stirred at room temperature for 30 minutes under the atmosphere of nitrogen. Then indole **1a** (43.5 mg, 0.3 mmol) in DCM (1.5 mL) was added to the mixture of catalyst via a syringe. After 24 h, the reaction was complete (monitored by TLC). Then, the reaction was filtered through a glass funnel within layer of silica gel (100-200 mesh) and purified by flash column chromatography (Pet/EtOAc, v/v, 10:1-2:1) to give the product **3a** as a white solid (90.1 mg, 72% yield, 98% ee); $R_f$ = 0.42 (Pet/EtOAc, 2/1, v/v); m.p.: 165.3-168.8 °C. HPLC CHIRALPAK IA, n-hexane/2-propanol = 80/20, flow rate 0.8 mL/min, λ = 254 nm, retention time: 22.992

min (minor) , 15.188 min (major) ; $[\alpha]_D^{25.5}$ = +65.6 (*c* = 0.2, in $CHCl_3$). $^1H$ NMR (400 MHz, $CDCl_3$) δ 7.12 (td, *J* = 7.6, 1.2 Hz, 1H), 6.62 (td, *J* = 7.6, 1.2 Hz, 1H), 6.55–6.51 (m, 2H), 4.39 (dd, *J* = 14.0, 6.4 Hz, 1H), 4.34 (d, *J* = 1.2 Hz, 1H), 3.99 (t, *J* = 13.6 Hz, 1H), 3.81 (s, 3H), 3.79 (s, 3H), 2.76 (s, 3H), 2.58 (br, 4H), 2.20 (qd, *J* = 6.4, 1.6 Hz, 1H), 1.55 (s, 3H); $^{13}C$ NMR (100 MHz, $CDCl_3$) δ 170.9, 168.5, 154.4, 132.5, 128.9, 122.2, 118.4, 109.6, 82.9, 63.5, 60.5, 56.2, 53.1, 52.7, 39.2, 30.9, 27.8; HRMS (m/z): $(M+ Na)^+$ calcd. for $C_{21}H_{24}N_2NaO_6^+$, 423.1527; found, 423.1527.

**Dimethyl-1-(2,5-dioxopyrrolidin-1-yl)-4,8*b*-dimethyl-1,3*a*,4,8*b*-tetrahydrocyclopenta[*b*] indole-3,3(2*H*)-dicarboxylate (3a')**

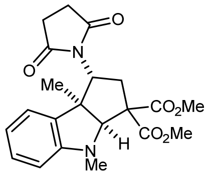


Colorless oil (8.4 mg, 7% yield, 94% ee); $R_f$ = 0.46 (Pet/EtOAc , 2/1, v/v). HPLC CHIRALPAK IA, n-hexane/2-propanol = 80/20, flow rate 0.8 mL/min, λ = 254 nm, retention time: 12.035 min (minor), 18.368 min (major); $[\alpha]_D^{25.5}$ = +198.8 (*c* = 0.15, in $CHCl_3$); $^1H$ NMR (400 MHz, $CDCl_3$) δ 7.09-7.00 (m, 2H), 6.65 (t, *J* = 7.2 Hz, 1H), 6.33 (d, *J* = 8.0 Hz, 1H), 5.21 (dd, *J* = 9.6, 8.0, 1H), 4.49 (s, 1H), 3.79 (s, 3H), 3.40 (s, 3H), 3.23 (dd, *J* = 13.6, 9.6 Hz, 1H), 2.97 (s, 3H), 2,75 (s, 4H), 2.55 (dd, *J* = 13.6, 8.4 Hz, 1H), 1.13 (s, 3H); $^{13}C$ NMR (100 MHz, $CDCl_3$) δ 177.9, 171.9, 170.2, 149.4, 135.9, 128.4, 122.9, 117.8, 106.3, 82.0, 63.9, 58.3, 56.0, 53.0, 52.4, 34.4, 33.2, 28.2, 23.9; HRMS (m/z): $(M+ Na)^+$ calcd. for $C_{21}H_{24}N_2NaO_6^+$, 423.1527; found, 423.1527.

**Dimethyl-2-(2-(1,3-dimethyl-1*H*-indol-2-yl)-2-(2,5-dioxopyrrolidin-1-yl)ethyl)-2-methylma lonate (4a')**

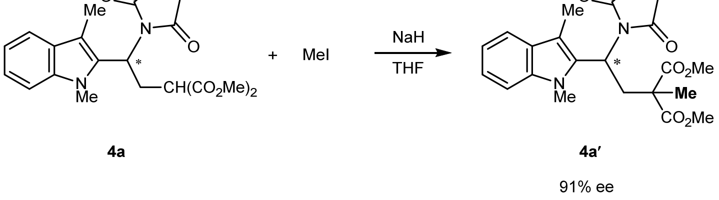


Products **3a** and **4a** were obtained by flash chromatography as a mixture (colorless oil, 121.0

mg). In order to separate products **3a** and **4a**, in a mixture of **3a** and **4a** in THF, NaH (1.5 equiv as 60% dispersion in mineral oil) was added at 0 °C and stirred for 15 min. Subsequently, $CH_3I$ (60.0 µL, 2.0 equiv) was added dropwise and the reaction was stirred at 0 °C for another 10 min. The reaction was then warmed to room temperature and stirred for 6 h. After that, the reaction mixture was quenched with distilled water. The reaction mixture was then concentrated in vacuo to remove THF. The aqueous layer was washed for 3 times with dichloromethane. The organic layer was dried over $Na_2SO_4$ then filtered, concentrated, and purified by flash column chromatography to give the product **4a'** as a colorless oil (19.2 mg, 0.046 mmol, 91% ee): $R_f$ = 0.44 (Pet/EtOAc, 2/1, v/v). HPLC CHIRALPAK IA, n-hexane/2-propanol = 80/20, flow rate 0.8 mL/min, λ = 254 nm, retention time: 12.030 min (minor), 16.227 min (major); $[\alpha]_D^{25.5}$ = +98.8 (*c* = 0.2, in $CHCl_3$). $^1$H NMR (600 MHz, $CDCl_3$) δ 7.55 (d, *J* = 7.8 Hz, 1H), 7.25-7.19 (m, 2H), 7.10-7.05 (m, 1H), 5.85 (dd, *J* = 7.8, 5.4 Hz, 1H), 3.78 (s, 3H), 3.59 (s, 6H), 3.32 (dd, *J* = 15.0, 7.8 Hz, 1H), 3.02 (dd, *J* = 15.0, 5.4 Hz, 1H), 2.64 - 2.58 (m, 4H), 2.57 (s, 3H), 1.52 (s, 3H); $^{13}$C NMR (150 MHz, $CDCl_3$) δ 176.9, 172.2, 172.1, 136.9, 131.5, 128.4, 122.6, 119.12, 119.09, 111.8, 109.3, 53.1, 52.9, 52.8, 44.4, 35.7, 30.7, 28.1, 20.3, 10.4; HRMS (m/z): $(M+ Na)^+$ calcd. for $C_{22}H_{26}N_2NaO_6^+$, 437.1683; found, 437.1683.

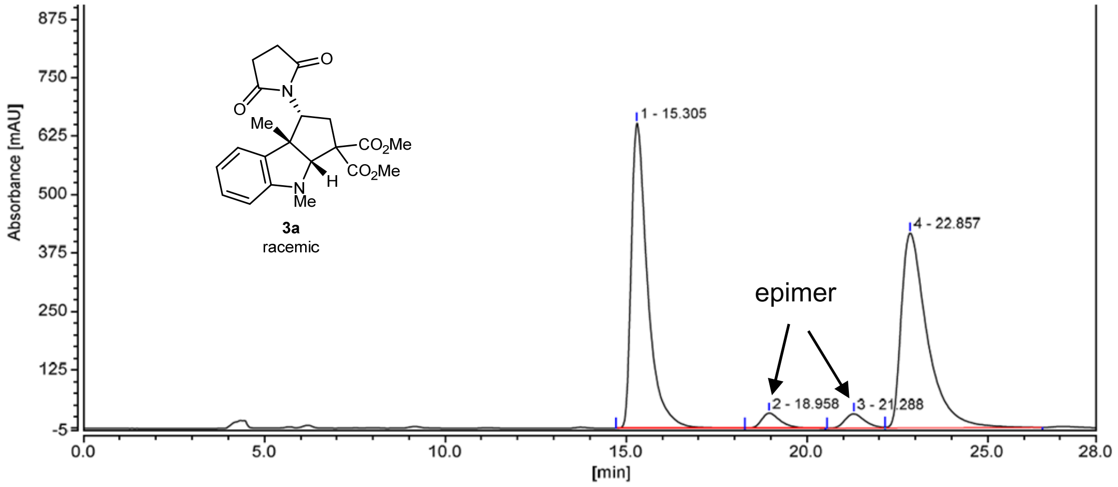


| Peak | Retention Time min | Area mAU*min | Height mAU | Area % | Height % |
|---|---|---|---|---|---|
| 1 | 15.305 | 341.327 | 652.988 | 46.91 | 57.63 |
| 2 | 18.958 | 19.633 | 32.148 | 2.70 | 2.84 |
| 3 | 21.288 | 19.320 | 30.412 | 2.65 | 2.68 |
| 4 | 22.857 | 347.417 | 417.486 | 47.74 | 36.85 |
| **Total:** | | **727.696** | **1133.033** | **100.00** | **100.00** |

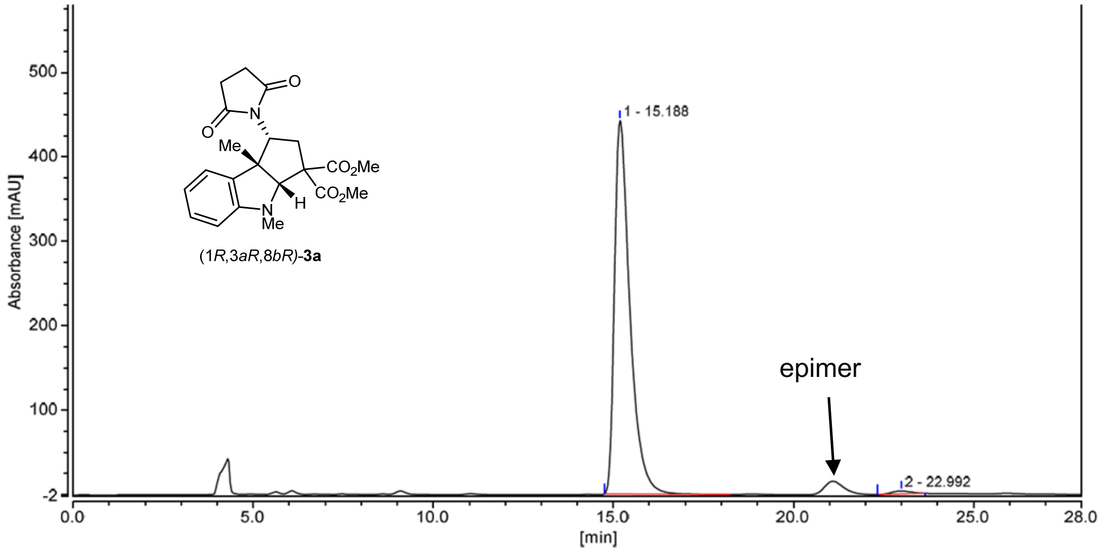


| Peak | Retention Time min | Area mAU*min | Height mAU | Area % | Height % |
|---|---|---|---|---|---|
| 1 | 15.188 | 219.510 | 441.998 | 99.03 | 99.22 |
| 2 | 22.992 | 2.157 | 3.489 | 0.97 | 0.78 |
| **Total:** | | **221.667** | **445.487** | **100.00** | **100.00** |

**Supplementary Fig. 2** HPLC spectra of product **3a** in this work

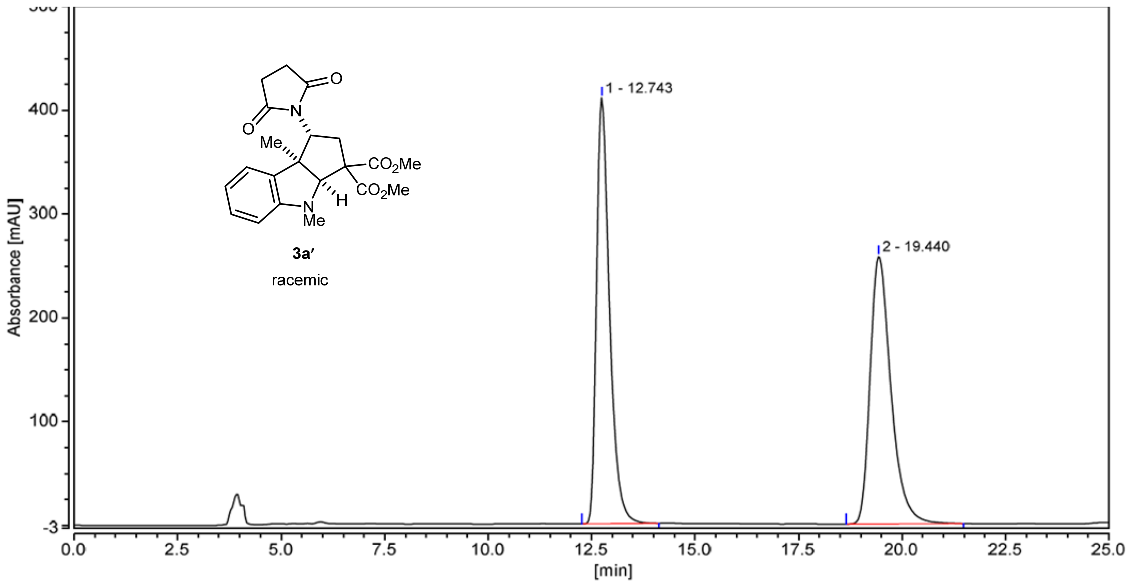


| Peak | Retention Time<br>min | Area<br>mAU*min | Height<br>mAU | Area<br>% | Height<br>% |
|---|---|---|---|---|---|
| 1 | 12.743 | 149.774 | 410.781 | 49.76 | 61.41 |
| 2 | 19.440 | 151.220 | 258.131 | 50.24 | 38.59 |
| **Total:** | | **300.994** | **668.912** | **100.00** | **100.00** |

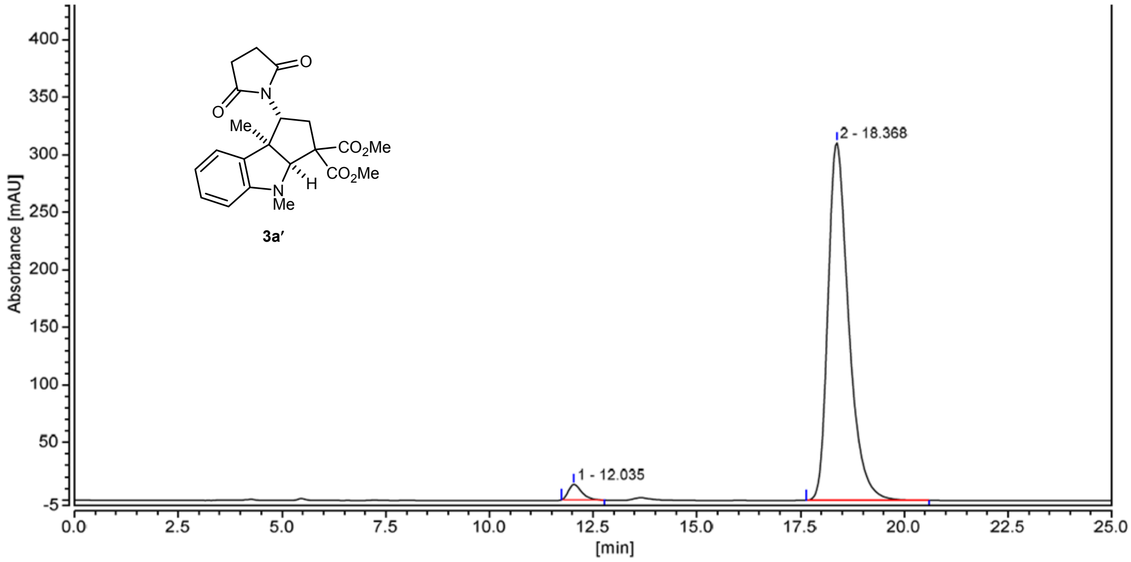


| Peak | Retention Time<br>min | Area<br>mAU*min | Height<br>mAU | Area<br>% | Height<br>% |
|---|---|---|---|---|---|
| 1 | 12.035 | 4.927 | 13.416 | 2.67 | 4.14 |
| 2 | 18.368 | 179.449 | 310.980 | 97.33 | 95.86 |
| **Total:** | | **184.376** | **324.396** | **100.00** | **100.00** |

**Supplementary Fig. 3** HPLC spectra of product **3a'** in this work

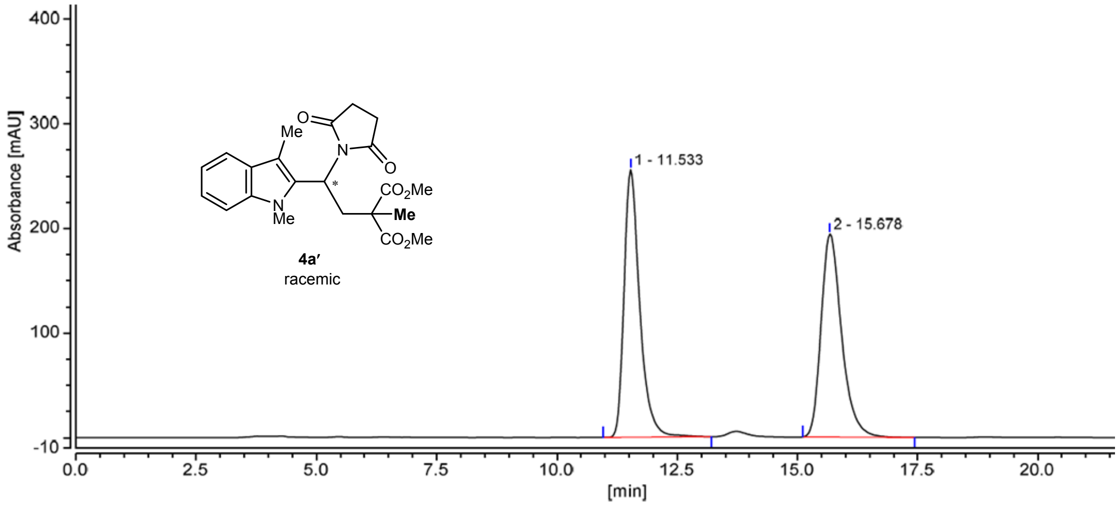


| Peak | Retention Time min | Area mAU*min | Height mAU | Area % | Height % |
|---|---|---|---|---|---|
| 1 | 11.533 | 96.703 | 255.866 | 50.19 | 56.86 |
| 2 | 15.678 | 95.990 | 194.167 | 49.81 | 43.14 |
| **Total:** | | **192.692** | **450.033** | **100.00** | **100.00** |

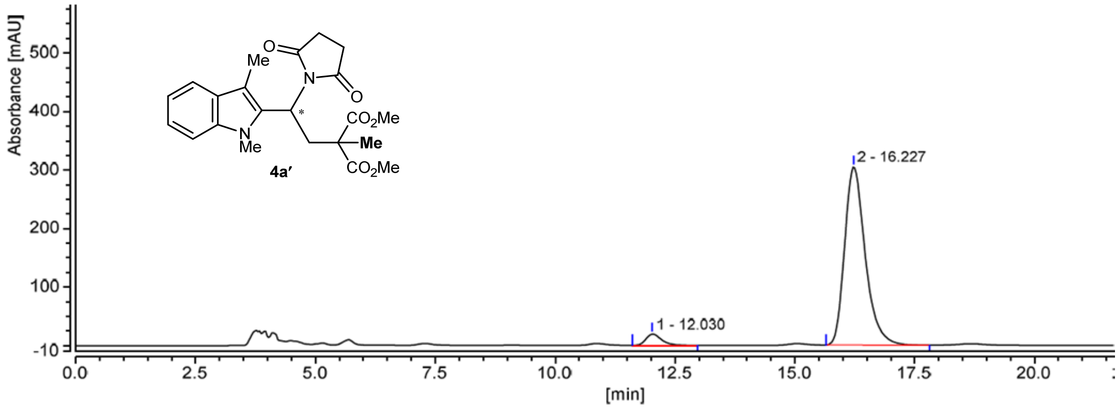


| Peak | Retention Time min | Area mAU*min | Height mAU | Area % | Height % |
|---|---|---|---|---|---|
| 1 | 12.030 | 7.458 | 19.540 | 4.68 | 6.03 |
| 2 | 16.227 | 151.931 | 304.691 | 95.32 | 93.97 |
| **Total:** | | **159.389** | **324.231** | **100.00** | **100.00** |

**Supplementary Fig. 4** HPLC spectra of byproduct **4a'** in this work.

**(1*R*,3*aR*,8*bR*)-Dimethyl-1-(2,5-dioxopyrrolidin-1-yl)-4,7,8*b*-trimethyl-1,3*a*,4,8*b*-tetrahydrocyclopenta[*b*]indole-3,3(2*H*)-dicarboxylate (3b)**

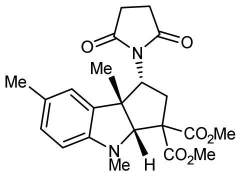


Prepared according to supplementary method C – In a dry reaction tube, a mixture of $Ni(OTf)_2$ (3.6 mg, 0.01 mmol, 5 mol%), ligand **L4** (7.9 mg, 0.012 mmol, 6 mol%), and aminocyclopropane **2a** (112.2 mg, 0.44 mmol) in DCM (3.0 mL) were stirred at room temperature for 30 minutes under the atmosphere of nitrogen. Then indole **1b** (31.8 mg, 0.2 mmol) in DCM (1.0 mL) was added to the mixture of catalyst via a syringe. After 24 h, the reaction was complete (monitored by TLC). Then, the reaction was filtered through a glass funnel within layer of silica gel (100-200 mesh) and purified by flash column chromatography (Pet/EtOAc, v/v, 10:1-1:1) to give the product **3b** as a colorless oil (62.1 mg, 75% yield, 98% ee, 10:1 dr); $R_f$ = 0.48 (Pet/EtOAc , 2/1, v/v). HPLC CHIRALPAK ID, n-hexane/2-propanol = 80/20, flow rate 0.8 mL/min, λ = 254 nm, retention time: 33.097 min (minor), 35.922 min (major); $[\alpha]_D^{25.5}$ = +12.00 (*c* = 0.2, in $CHCl_3$). $^1$H NMR (400 MHz, $CDCl_3$) δ 6.93 (d, *J* = 8.8 Hz, 1H), 6.44 (d, *J* = 8.0 Hz, 1H), 6.34 (s,1H), 4.38 (dd, *J* = 13.6, 6.0 Hz, 1H), 4.29 (s, 1H), 4.00 (t, *J* = 13.2 Hz, 1H), 3.81 (s, 3H), 3.79 (s, 3H), 2.73 (s, 3H), 2.62–2.30 (m, 4H), 2.21 (d, *J* = 7.2 Hz, 1H), 2.19 (s, 3H), 1.54 (s, 3H); $^{13}$C NMR (100 MHz, $CDCl_3$) δ 170.9, 168.5, 152.4, 132.7, 129.3, 127.6, 123.0, 109.8, 83.3, 63.6, 60.5, 56.3, 53.1, 52.7, 39.9, 30.9, 27.8, 21.0; HRMS (m/z): $(M+ Na)^+$ calcd. for $C_{22}H_{26}N_2NaO_6^+$, 437.1683; found, 437.1683.

**(1*R*,3*aR*,8*bR*)-Dimethyl-1-(2,5-dioxopyrrolidin-1-yl)-7-methoxy-4,8*b*-dimethyl-1,3*a*,4,8*b*-tetrahydrocyclopenta[*b*]indole-3,3(2*H*)-dicarboxylate (3c)**

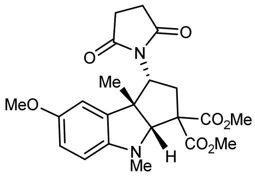


Prepared according to supplementary method C - In a dry reaction tube, a mixture of $Ni(OTf)_2$ (3.6 mg, 0.01 mmol, 5 mol%), ligand **L4** (7.9 mg, 0.012 mmol, 6 mol%), and

aminocyclopropane **2a** (112.2 mg, 0.44 mmol) in DCM (3.0 mL) were stirred at room temperature for 30 minutes under the atmosphere of nitrogen. Then indole **1c** (35.0 mg, 0.2 mmol) in DCM (1.0 mL) was added to the mixture of catalyst via a syringe. After 24 h, the reaction was complete (monitored by TLC). Then, the reaction was filtered through a glass funnel within layer of silica gel (100-200 mesh) and purified by flash column chromatography (Pet/EtOAc, v/v, 10:1-1:1) to give the product **3c** as a colorless oil (70.5 mg, 82% yield, 95% ee, 9:1 dr); $R_f$ = 0.45 (Pet/EtOAc, 2/1, v/v). HPLC CHIRALPAK IA, n-hexane/2-propanol = 70/30, flow rate 0.8 mL/min, λ = 254 nm, retention time: 24.335 (minor), 11.315min (major) ; $[\alpha]_D^{25.5}$ = +9.0 (*c* = 0.2, in $CHCl_3$). $^1$H NMR (400 MHz, $CDCl_3$) δ 6.69 (dd, *J* = 8.8, 2.8 Hz, 1H), 6.46 (d, *J* = 8.8 Hz, 1H), 6.15 (d, *J* = 2.4 Hz, 1H), 4.39 (dd, *J* = 13.6, 6.0 Hz, 1H), 4.27 (d, *J* = 1.2 Hz, 1H), 4.00 (t, *J* = 13.6, 1H), 3.81 (s, 3H), 3.79 (s, 3H), 3.68 (s, 3H), 2.72 (s, 3H), 2.56 (br, 4H), 2.19 (qd, *J* = 6.0, 1.2 Hz, 1H), 1.54 (s, 3H); $^{13}$C NMR (100 MHz, $CDCl_3$) δ 171.0, 168.5, 153.6, 148.9, 134.1, 112.8, 110.3, 110.2, 83.6, 63.7, 60.4, 56.3, 56.1, 53.1, 52.7, 40.7, 31.0, 27.8; HRMS (m/z): $(M+ Na)^+$ calcd. for $C_{22}H_{26}N_2NaO_7^+$, 453.1632; found, 453.1628.

**(1*R*,8*bR*)-Dimethyl-7-chloro-1-(2,5-dioxopyrrolidin-1-yl)-4,8*b*-dimethyl-1,3*a*,4,8*b*-tetrahydrocyclopenta[*b*]indole-3,3(2*H*)-dicarboxylate (3d)**

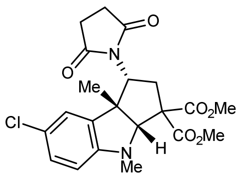


Prepared according to supplementary method C - In a dry reaction tube, a mixture of $Ni(OTf)_2$ (3.6 mg, 0.01 mmol, 5 mol%), ligand **L4** (7.9 mg, 0.012 mmol, 6 mol%), and aminocyclopropane **2a** (112.2 mg, 0.44 mmol) in DCM (3.0 mL) were stirred at room temperature for 30 minutes under the atmosphere of nitrogen. Then indole **1d** (35.8 mg, 0.2 mmol) in DCM (1.0 mL) was added to the mixture of catalyst via a syringe. After 36 h, the reaction was complete (monitored by TLC). Then, the reaction was filtered through a glass funnel within layer of silica gel (100-200 mesh) and purified by flash column chromatography (Pet/EtOAc, v/v, 10:1-3:1) to give the product **3d** as a white solid (59.2 mg, 68% yield, 96% ee, 6:1 dr) ; $R_f$ = 0.50 (Pet/EtOAc, 2/1, v/v); m.p.: 195.3-198.8 °C. HPLC CHIRALPAK IA, n-hexane/2-propanol = 70/30, flow rate 0.8 mL/min, λ = 254 nm, retention time: 18.885 min

(minor), 9.347 min (major) ; $[\alpha]_D^{25.5}$ = +37.0 (*c* = 0.2, in $CHCl_3$). $^1H$ NMR (400 MHz, $CDCl_3$) δ 7.05 (dd, *J* = 8.4, 2.0 Hz, 1H), 6.46 (d, *J* = 2.4 Hz, 1H), 6.42 (d, *J* = 8.4 Hz, 1H), 4.41–4.36 (m, 2H), 3.98 (t, *J* = 13.2 Hz, 1H), 3.81 (s, 3H), 3.79 (s, 3H), 2.74 (s, 3H), 2.60 (br, 4H), 2.25–2.17 (m, 1H), 1.53 (s, 3H); $^{13}C$ NMR (100 MHz, $CDCl_3$) δ 170.7, 168.3, 153.0, 134.4, 128.6, 123.0, 122.5, 110.4, 83.2, 63.5, 60.3, 56.2, 53.2, 52.8, 39.2, 30.8, 27.9, 27.5; HRMS (m/z): (M+ Na)$^+$ calcd. for $C_{21}H_{23}ClN_2NaO_6^+$, 457.1137; found, 457.1135.

**(1*R*,3*aR*,8*bR*)-Dimethyl-7-bromo-1-(2,5-dioxopyrrolidin-1-yl)-4,8*b*-dimethyl-1,3*a*,4,8*b*-tetrahydrocyclopenta[*b*]indole-3,3(2*H*)-dicarboxylate (3e)**

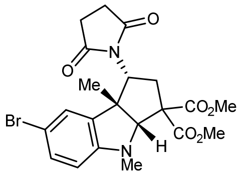


Prepared according to supplementary method C - In a dry reaction tube, a mixture of $Ni(OTf)_2$ (3.6 mg, 0.01 mmol, 5 mol%), ligand **L4** (7.9 mg, 0.012 mmol, 6 mol%), and aminocyclopropane **2a** (112.2 mg, 0.44 mmol) in DCM (3.0 mL) were stirred at room temperature for 30 minutes under the atmosphere of nitrogen. Then indole **1e** (44.4 mg, 0.2 mmol) in DCM (1.0 mL) was added to the mixture of catalyst via a syringe. After 36 h, the reaction was complete (monitored by TLC). Then, the reaction was filtered through a glass funnel within layer of silica gel (100-200 mesh) and purified by flash column chromatography (Pet/EtOAc, v/v, 10:1-1:1) to give the product **3e** as a white solid (62.4 mg, 65% yield, 97% ee, 6:1 dr), $R_f$ = 0.42 (Pet/EtOAc, 2/1, v/v); m.p.: 206.4-207.5 °C. HPLC CHIRALPAK IA, n-hexane/2-propanol = 70/30, flow rate 0.8 mL/min, λ = 254 nm, retention time: 19.377 min (minor), 9.375 min (major); $[\alpha]_D^{25.5}$ = +43.0 (*c* = 0.2, in $CHCl_3$). $^1H$ NMR (600 MHz, $CDCl_3$) δ 6.97 (t, *J* = 7.8 Hz, 1H), 6.75 (d, *J* = 7.8 Hz, 1H), 6.45 (d, *J* = 7.8 Hz, 1H), 4.45 (dd, *J* = 14.4, 6.0 Hz, 1H), 4.39 (d, *J* = 1.2 Hz, 1H), 3.93 (t, *J* = 13.2 Hz, 1H), 3.82 (s, 3H), 3.79 (s, 3H), 2.77–2.72 (m, 4H), 2.63–2.56 (m, 2H), 2.47–2.43 (m, 1H), 2.17–2.14 (m, 1H), 1.82 (s, 3H); $^{13}C$ NMR (150 MHz, $CDCl_3$) δ 178.6, 176.9, 170.5, 156.6, 130.3, 129.9, 123.6, 118.1, 108.5, 83.8, 62.9, 59.8, 57.4, 53.1, 52.8, 39.1, 31.4, 28.3, 28.0, 26.5; HRMS (m/z): (M+ Na)$^+$ calcd. for $C_{21}H_{23}BrN_2NaO_6^+$, 501.0632; found, 501.0632.

**(1*R*,3*aR*,8*bR*)-Dimethyl-6-bromo-1-(2,5-dioxopyrrolidin-1-yl)-4,8*b*-dimethyl-1,3*a*,4,8*b*-tetrahydrocyclopenta[*b*]indole-3,3(2*H*)-dicarboxylate (3f)**

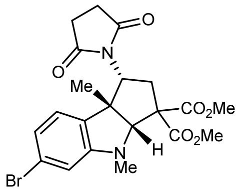


Prepared according to supplementary method C – In a dry reaction tube, a mixture of $Ni(OTf)_2$ (3.6 mg, 0.01 mmol, 5 mol%), ligand **L4** (7.9 mg, 0.012 mmol, 6 mol%), and aminocyclopropane **2a** (112.2 mg, 0.44 mmol) in DCM (3.0 mL) were stirred at room temperature for 30 minutes under the atmosphere of nitrogen. Then indole **1f** (44.4 mg, 0.2 mmol) in DCM (1.0 mL) was added to the mixture of catalyst via a syringe. After 36 h, the reaction was complete (monitored by TLC). Then, the reaction was filtered through a glass funnel within layer of silica gel (100-200 mesh) and purified by flash column chromatography (Pet/EtOAc, v/v, 10:1-1:1) to give the product **3f** as a white solid (69.8 mg, 71% yield, 98% ee, 7:1 dr); $R_f$ = 0.52 (Pet/EtOAc, 2/1, v/v); m.p.: 222.1-224.5 $^{o}$C. HPLC CHIRALPAK IA, n-hexane/2-propanol = 70/30, flow rate 0.8 mL/min, λ = 254 nm, retention time: 35.050 min (minor), 18.843 min (major); $[\alpha]_D^{25.5}$ = +181.3 (*c* = 0.2, in $CHCl_3$). $^1$H NMR (600 MHz, $CDCl_3$) δ 6.71 (dd, *J* = 8.4, 1.8 Hz, 1H), 6.61 (d, *J* = 1.2 Hz, 1H), 6.37 (d, *J* = 7.8 Hz, 1H), 4.42-4.30 (m, 2H), 3.98 (t, *J* = 13.2 Hz, 1H), 3.80 (d, *J* = 11.4 Hz, 6H), 2.74 (s, 3H), 2.57 (s, 4H), 2.23–2.17 (m, 1H), 1.51 (s, 3H); $^{13}$C NMR (150 MHz, $CDCl_3$) δ 170.7, 168.3, 155.5, 131.7, 123.3, 122.8, 121.1, 112.5, 83.0, 63.4, 60.2, 55.9, 53.2, 52.9, 38.6, 30.9, 27.9, 27.7; HRMS (m/z): (M+Na)$^+$ calcd. for $C_{21}H_{23}BrN_2NaO_6^+$, 501.0632; found, 501.0624.

**(1*R*,3a*R*,8*bR*)-Dimethyl-8*b*-allyl-1-(2,5-dioxopyrrolidin-1-yl)-4-methyl-1,3*a*,4,8*b*-tetrahydrocyclopenta[*b*]indole-3,3(2*H*)-dicarboxylate (3g)**

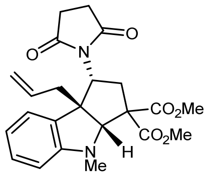

Prepared according to supplementary method C - In a dry reaction tube, a mixture of $Ni(OTf)_2$ (3.6 mg, 0.01 mmol, 5 mol%), ligand **L4** (7.9 mg, 0.012 mmol, 6 mol%), and aminocyclopropane **2a** (112.2 mg, 0.44 mmol) in DCM (3.0 mL) were stirred at room temperature for 30 minutes under the atmosphere of nitrogen. Then indole **1g** (34.2 mg, 0.2 mmol) in DCM (1.0 mL) was added to the mixture of catalyst via a syringe. After 24 h, the reaction was complete (monitored by TLC). Then, the reaction was filtered through a glass funnel within layer of silica gel (100-200 mesh) and purified by flash column chromatography (Pet/EtOAc, v/v, 10:1-3:1) to give the product **3g** as a colorless oil (61.3 mg, 72% yield, 93% ee, 9:1 dr); $R_f$ = 0.56 (Pet/EtOAc, 2/1, v/v). HPLC CHIRALPAK OD-H, n-hexane/2-propanol = 80/20, flow rate 0.8 mL/min, λ = 254 nm, retention time: 25.898 min (minor), 21.547 min (major); $[\alpha]_D^{25.5}$ = +9.0 (*c* = 0.2, in $CHCl_3$). $^1H$ NMR (400 MHz, $CDCl_3$) δ 7.11 (t, *J* = 7.6 Hz, 1H), 6.63 (t, *J* = 7.2 Hz, 1H), 6.54–6.50 (m, 2H), 5.27–5.18 (m, 1H), 5.15–5.10 (m, 1H), 4.95 (dd, *J* = 10.0, 2.4 Hz, 1H), 4.48 (s, 1H), 4.41 (dd, *J* = 14.0, 6.0 Hz, 1H), 4.00 (t, *J* = 13.2 Hz, 1H,), 3.81 (s, 3H), 3.78 (s, 3H), 2.78 (dd, *J* = 14.4, 4.8 Hz, 1H), 2.71 (s, 3H), 2.67–2.50 (m, 5H), 2.24–2.15 (m, 1H); $^{13}C$ NMR (100 MHz, $CDCl_3$) δ 171.1, 168.5, 155.4, 135.4, 129.8, 129.0, 122.5, 118.7, 118.3, 110.0, 78.5, 63.5, 60.4, 60.2, 53.1, 52.7, 42.9, 39.2, 30.7, 27.9; HRMS (m/z): $(M+ Na)^+$ calcd. for $C_{23}H_{26}N_2NaO_6^+$, 449.1683; found, 449.1683.

**(1*R*,3*aR*,8*bR*)-Dimethyl-8*b*-(2-((tert-butyldimethylsilyl)oxy)ethyl)-1-(2,5-dioxopyrrolidin-1-yl)-4-methyl-1,3*a*,4,8*b*-tetrahydrocyclopenta[*b*]indole-3,3(2*H*)-dicarboxylate (3h)**

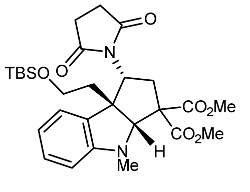


Prepared according to supplementary method C - In a dry reaction tube, a mixture of $Ni(OTf)_2$ (3.6 mg, 0.01 mmol, 5 mol%), ligand **L4** (7.9 mg, 0.012 mmol, 6 mol%), and aminocyclopropane **2a** (112.2 mg, 0.44 mmol) in DCM (3.0 mL) were stirred at room temperature for 30 minutes under the atmosphere of nitrogen. Then indole **1h** (57.8 mg, 0.2 mmol) in DCM (1.0 mL) was added to the mixture of catalyst via a syringe. After 24 h, the reaction was complete (monitored by TLC). Then, the reaction was filtered through a glass

funnel within layer of silica gel (100-200 mesh) and purified by flash column chromatography (Pet/EtOAc, v/v, 10:1-3:1) to give the product **3h** as a white solid (69.4 mg, 65% yield, 96% ee, 8:1 dr); $R_f$ = 0.36 (Pet/EtOAc, 1/1, v/v); m.p.: 173.2-175.3 $^{o}$C. HPLC CHIRALPAK OD-H, n-hexane/2-propanol = 80/20, flow rate 0.8 mL/min, λ = 254 nm, retention time: 13.299 min (minor), 26.782 min (major); $[\alpha]_D^{25.5}$ = +234.0 (*c* = 0.2, in $CHCl_3$). $^1$H NMR (600 MHz, $CDCl_3$) δ 7.11 (t, *J* = 7.8 Hz, 1H), 6.61 (t, *J* = 7.8 Hz, 1H), 6.50 (dd, *J* = 16.8, 7.8 Hz, 2H), 4.87 (d, *J* = 1.2 Hz, 1H), 4.40 (q, *J* = 6.6 Hz, 1H), 3.91 (t, *J* = 13.2 Hz, 1H), 3.81 (d, *J* = 10.8 Hz, 6H), 3.40–3.36 (m, 1H), 3.29–3.26 (m, 1H ), 2.74 (s, 3H), 2.54 (br, 4H), 2.19–2.10 (m, 3H), 0.82 (s, 9H), -0.044 (s, 3H), -0.062 (s, 3 H); $^{13}$C NMR (150 MHz, $CDCl_3$) δ 170.9, 168.6, 155.2, 129.2, 129.0, 122.7, 118.5, 110.0, 79.0, 63.9, 61.0, 60.8, 59.0, 53.1, 52.7, 40.5, 39.4, 29.8, 26.1, 18.4, -5.2, -5.3; HRMS (m/z): $(M+Na)^+$ calcd. for $C_{28}H_{40}N_2NaO_7Si^+$, 567.2497; found, 567.2498.

**(1*R*,3*aR*,8*bR*)-Dimethyl-8*b*-(2-acetoxyethyl)-1-(2,5-dioxopyrrolidin-1-yl)-4-methyl-1,3*a*, 4, 8*b*-tetrahydrocyclopenta[*b*]indole-3,3(2*H*)-dicarboxylate (3i)**

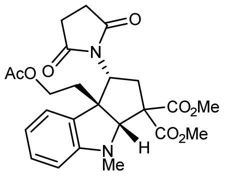


Prepared according to supplementary method C - In a dry reaction tube, a mixture of $Ni(OTf)_2$ (3.6 mg, 0.01 mmol, 5 mol%), ligand **L4** (7.9 mg, 0.012 mmol, 6 mol%), and aminocyclopropane **2a** (112.2 mg, 0.44 mmol) in DCM (3.0 mL) were stirred at room temperature for 30 minutes under the atmosphere of nitrogen. Then indole **1i** (43.4 mg, 0.2 mmol) in DCM (1.0 mL) was added to the mixture of catalyst via a syringe. After 24 h, the reaction was complete (monitored by TLC). Then, the reaction was filtered through a glass funnel within layer of silica gel (100-200 mesh) and purified by flash column chromatography (Pet/EtOAc, v/v, 10:1-3:1) to give the product **3i** as a colorless oil (71.9 mg, 76% yield, 97% ee, 7:1 dr); $R_f$ = 0.42 (Pet/EtOAc, 1/1, v/v). HPLC CHIRALPAK IA, n-hexane/2-propanol = 80/20, flow rate 0.8 mL/min, λ = 254 nm, retention time: 27.162 min (minor), 16.630 min (major); $[\alpha]_D^{25.5}$ = +15.0 (*c* = 0.2, in $CHCl_3$). $^1$H NMR (400 MHz, $CDCl_3$) δ 7.11 (td, *J* = 7.2, 1.2 Hz, 1H), 6.63 (t, *J* = 7.2 Hz, 1H), 6.51 (t, *J* = 7.6 Hz, 2H), 4.59 (d, *J* = 1.2 Hz, 1H), 4.39 (dd, *J* = 14.0, 6.4 Hz, 1H), 3.95 (t, *J* = 13.2 Hz, 1H), 3.88–3.82 (m, 2H), 3.81 (s, 3H), 3.80 (s, 3H), 2.76

(s, 3H), 2.67–2.42 (m, 4H), 2.38–2.31 (m, 1H), 2.27–2.19 (m, 1H), 2.15 (qd, *J* = 6.0, 1.2 Hz, 1H), 1.88 (s, 3H); $^{13}$C NMR (100 MHz, $CDCl_3$) δ 171.1, 170.9, 168.4, 155.0, 129.3, 128.5, 122.6, 118.7, 110.2, 78.9, 63.7, 62.2, 60.7, 58.8, 53.2, 52.7, 39.4, 36.3, 29.9, 20.9; HRMS (m/z): $(M+ Na)^+$ calcd. for $C_{24}H_{28}N_2NaO_8^+$, 495.1738; found, 495.1730.

**(1*R*,8b*R*)-Dimethyl-1-(2,5-dioxopyrrolidin-1-yl)-4-methyl-1,2-dihydro-3*H*,4*H*-3*a*,8*b*-propanocyclopenta[*b*]indole-3,3-dicarboxylate (3j)**

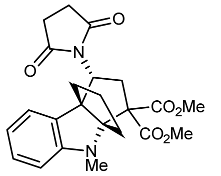


Prepared according to supplementary method C - In a dry reaction tube, a mixture of $Ni(OTf)_2$ (3.6 mg, 0.01 mmol, 5 mol%), ligand **L4** (7.9 mg, 0.012 mmol, 6 mol%), and aminocyclopropane **2a** (112.2 mg, 0.44 mmol) in DCM (3.0 mL) were stirred at room temperature for 30 minutes under the atmosphere of nitrogen. Then indole **1j** (34.6 mg, 0.2 mmol) in DCM (1.0 mL) was added to the mixture of catalyst via a syringe. After 24 h, the reaction was complete (monitored by TLC). Then, the reaction was filtered through a glass funnel within layer of silica gel (100-200 mesh) and purified by flash column chromatography (Pet/EtOAc, v/v, 10:1-4:1) to give the product **3j** as a white solid (71.5 mg, 80% yield, 96% ee, 1.8:1 dr); $R_f$ = 0.45 (Pet/EtOAc, 3/1, v/v); m.p.: 220.4-223.5 $^oC$. HPLC CHIRALPAK IA, n-hexane/2-propanol = 70/30, flow rate 0.8 mL/min, λ = 254 nm, retention time: 13.357 min (minor), 10.920 min (major) ; $[\alpha]_D^{25.5}$ = +24.0 (*c* = 0.25, in $CHCl_3$). $^1$H NMR (400 MHz, $CDCl_3$) δ 7.10 (td, *J* = 7.6, 1.2 Hz, 1H), 6.57 (td, 7.2, 0.8 Hz, 1H), 6.47 (dd, *J* = 7.2, 1.2 Hz, 1H), 6.38 (d, 7.6 Hz, 1H), 4.84 (dd, *J* = 14.0, 6.0 Hz, 1H), 4.28 (t, *J* = 13.6 Hz, 1H), 3.79 (s, 3H), 3.76 (s, 3H), 2.73 (s, 3H), 2.70–2.35 (br, 6H), 2.18 (dd, *J* = 13.2, 6.0 Hz, 1H), 2.07–2.01 (m, 1H), 1.96–1.89 (m, 2H), 1.52–1.44 (m, 1H); $^{13}$C NMR (100 MHz, $CDCl_3$) δ 171.3, 169.2, 155.2, 131.2, 129.0, 122.2, 117.8, 108.0, 92.7, 70.0, 65.9, 59.8, 52.8, 52.4, 40.6, 35.5, 33.9, 29.7, 29.2; HRMS (m/z): $(M+ Na)^+$ calcd. for $C_{23}H_{26}N_2NaO_6^+$, 449.1683; found, 449.1683.

**Dimethyl-(1*R*,3*aR*,8*bR*)-1-(2,5-dioxopyrrolidin-1-yl)-3*a*,4,8*b*-trimethyl-1,3*a*,4,8*b*-tetrahydrocyclopenta[*b*]indole-3,3(2*H*)-dicarboxylate (3k)**

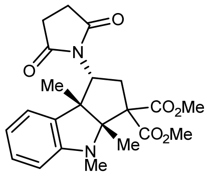


Prepared according to supplementary method C - In a dry reaction tube, a mixture of $Ni(OTf)_2$ (3.6 mg, 0.010 mmol, 5 mol%) and, **L4** (7.86 mg, 0.012 mmol, 6 mol%), the cyclopropane **2a** (112.2 mg, 0.44 mmol) in DCM (3.0 mL) was stirred at 30 °C for 30 minutes under the atmosphere of nitrogen. Then,the mixture was cooled to room temperature for 10 minutes and the indole **1k** (31.8 mg, 0.2 mmol) in 1.0 mL DCM were added to the mixture of catalyst via a syringe. After 36 hours, the reaction was complete (monitored by TLC), the reaction was filtered through a glass funnel with thin layer of silica gel (100-200 mesh) and eluted with (Pet/EtOAc, v/v, 10:1-2:1). The filtrate was concentrated under reduced pressure. After the determination of the diatereoselectivity by $^1H$ NMR, the residue was purified by flash chromatography to give the title product **3k** as a colorless oil (53.6 mg, 65% yield, 97% ee, 4:1 dr); $R_f$ = 0.41 (Pet/EtOAc, 3/1, v/v). HPLC CHIRALPAK IA, n-hexane/2-propanol = 80/20, flow rate 0.8 mL/min, λ = 254 nm, retention time: 22.330 min (minor), 18.163 min (major); $[\alpha]_D^{25.5}$ = +47.40 (*c* = 0.20, in $CHCl_3$). $^1H$ NMR (600 MHz, $CDCl_3$) δ 7.13–7.09 (m, 1H), 6.60–6.54 (m, 2H), 6.39 (d, *J* = 7.8 Hz, 1H), 4.59 (dd, *J* = 14.4, 6.6 Hz, 1H), 4.19 (t, *J* = 13.8 Hz, 1H), 3.82 (s, 3H), 3.74 (s, 3H), 2.77–2.70 (m, 1H), 2.65–2.57 (m, 4H), 2.41–2.28 (m, 2H), 1.94 (q, *J* = 6.6 Hz, 1H), 1.57 (s, 3H), 1.55 (s, 3H); $^{13}C$ NMR (100 MHz, $CDCl_3$) δ 178.4, 176.6, 170.9, 169.5, 153.2, 132.0, 129.1, 122.0, 117.4, 108.1, 82.9, 66.3, 60.7, 58.7, 52.7, 52.6, 32.2, 31.1, 28.1, 27.5, 22.8, 14.1. HRMS (m/z): $(M+Na)^+$ calcd. for $C_{22}H_{26}N_2NaO_6^+$, 437.1683; found, 437.1679.

**(1*R*,3*aR*,8*bR*)-Dimethyl-4-benzyl-1-(2,5-dioxopyrrolidin-1-yl)-8*b*-methyl-1,3*a*,4,8*b*-tetra hydrocyclopenta[*b*]indole-3,3(2*H*)-dicarboxylate (3l)**

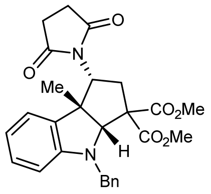


Prepared according to supplementary method C - In a dry reaction tube, a mixture of $Ni(OTf)_2$ (3.6 mg, 0.01 mmol, 5 mol%), ligand **L4** (7.9 mg, 0.012 mmol, 6 mol%), and aminocyclopropane **2a** (112.2 mg, 0.44 mmol) in DCM (3.0 mL) were stirred at room temperature for 30 minutes under the atmosphere of nitrogen. Then indole **1l** (44.2 mg, 0.2 mmol) in DCM (1.0 mL) was added to the mixture of catalyst via a syringe. After 24 h, the reaction was complete (monitored by TLC). Then, the reaction was filtered through a glass funnel within layer of silica gel (100-200 mesh) and purified by flash column chromatography (Pet/EtOAc, v/v, 10:1-2:1) to give the product **3l** as a colorless oil (68.5 mg, 72% yield, 94% ee, 10:1 dr); $R_f$ = 0.46 (Pet/EtOAc, 2/1, v/v). HPLC CHIRALPAK OD-H, n-hexane/2-propanol = 80/20, flow rate 0.7 mL/min, λ = 254 nm, retention time: 22.415 min (minor), 35.750 min (major); $[\alpha]_D^{25.5}$ = +23.4 (*c* = 0.15, in $CHCl_3$). $^1H$ NMR (400 MHz, $CDCl_3$) δ 7.32–7.29 (m, 2H), 7.25–7.21 (m, 3H), 7.03 (td, *J* = 7.6, 1.2 Hz, 1H), 6.65 (t, *J* = 7.6 Hz, 1H), 6.57 (dd, *J* = 7.2, 1.2 Hz, 1H ), 6.37 (d, *J* = 7.6 Hz, 1H), 4.63 (d, *J* = 1.2 Hz, 1H), 4.56 (d, *J* = 16.4 Hz, 1H), 4.45 (dd, *J* = 13.6, 6.4 Hz, 1H), 4.20–4.13 (m, 2H), 3.80 (s, 3H), 3.26 (s, 3H), 2.75–2.40 (m, 4H), 2.32–2.28 (qd, *J* = 6.4, 1.6 Hz, 1H), 1.51 (s, 3H); $^{13}C$ NMR (100 MHz, $CDCl_3$) δ 170.7, 168.3, 154.3, 138.9, 132.9, 129.0, 128.5, 127.0, 126.8, 122.1, 118.8, 110.3, 81.7, 64.1, 60.6, 57.0, 56.5, 53.2, 52.6, 30.8, 27.5; HRMS (m/z): $(M+ Na)^+$ calcd. for $C_{27}H_{28}N_2NaO_6^+$, 499.1840; found, 499.1839.

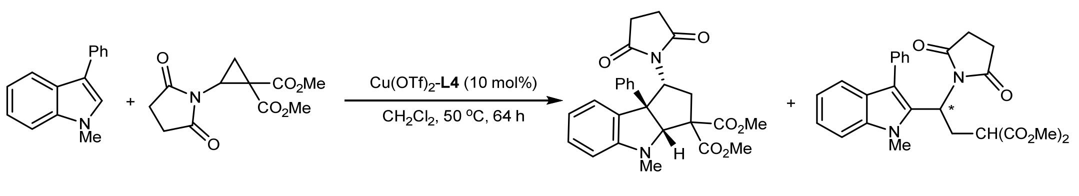


A mixture of $Cu(OTf)_2$ (7.2 mg, 0.020 mmol, 10 mol%) and, **L4** (7.86 mg, 0.024 mmol, 12 mol%), the cyclopropane **2a** (153.0 mg, 0.60 mmol) in DCM (3.0 mL) was stirred at 30 °C for 30 minutes under the atmosphere of nitrogen via a pressure-resistant test tube. Then, the mixture was heated to 50 °C for 10 minutes and the indole **1m** (41.4 mg, 0.2 mmol) in 1.0 mL DCM were added to the mixture of catalyst. After 64 hours, the reaction was complete (monitored by TLC), the reaction was filtered through a glass funnel with thin layer of silica gel (100-200 mesh) and eluted with (Pet/EtOAc, v/v, 10:1-2:1). The filtrate was concentrated under reduced pressure. After the determination of the diatereoselectivity by $^{1}H$ NMR, the residue was purified by flash chromatography to give the title product **3m** as a colorless oil (47.3 mg, 51% yield, 95% ee, 1.8:1 dr); $R_f$ = 0.31 (Pet/EtOAc, 2/1, v/v).

**Dimethyl-(1*R*,3*aR*,8*bR*)-1-(2,5-dioxopyrrolidin-1-yl)-4-methyl-8*b*-phenyl-1,3*a*,4,8*b*-tetrahydrocyclopenta[*b*]indole-3,3(2*H*)-dicarboxylate (3m)**

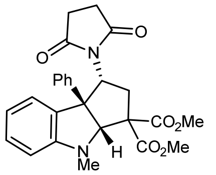


Colorless oil, HPLC CHIRALPAK IA, n-hexane/2-propanol = 80/20, flow rate 0.8 mL/min, λ = 254 nm, retention time: 22.175 min (minor), 18.645 min (major); $[\alpha]_D^{20.0}$ = +3.0 (*c* = 0.30, in $CHCl_3$). $^{1}H$ NMR (400 MHz, $CDCl_3$) δ 7.40 (d, *J* = 8.0 Hz, 2H), 7.30–7.24 (m, 2H), 7.20–7.13 (m, 2H), 6.64 (d, *J* = 8.0 Hz, 1H), 6.58 (t, *J* = 7.2 Hz, 1H), 6.29 (d, *J* = 7.2 Hz, 1H), 5.46 (q, *J* = 6.8 Hz, 1H), 4.70 (s, 1H), 4.12 (t, *J* = 8.8 Hz, 1H), 3.79 (s, 3H), 3.56 (s, 3H), 2.87 (s, 3H), 2.58–2.44 (m, 4H), 2.42–2.35 (m, 1H); $^{13}C$ NMR (100 MHz, $CDCl_3$) δ 177.1, 170.3, 168.3, 155.1, 145.1, 133.2, 129.1, 128.02, 127.95, 126.5, 124.8, 118.9, 110.0, 86.8, 64.9, 63.5, 56.1, 52.9, 52.8, 39.6, 31.0, 27.9. HRMS (m/z): $(M+Na)^+$ calcd. for $C_{26}H_{26}N_2NaO_6^+$, 485.1683; found, 485.1674.

**Dimethyl-2-(2-(2,5-dioxopyrrolidin-1-yl)-2-(1-methyl-3-phenyl-1*H*-indol-2-yl)-2λ₃-ethyl) malonate (4m)**

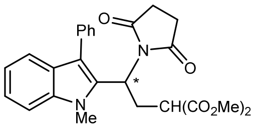


Colorless oil, $^{1}H$ NMR (400 MHz, $CDCl_3$) δ 7.45–7.30 (m, 6H), 7.27–7.21 (m, 2H), 7.05 (t, *J* = 7.2 Hz, 1H), 5.52 (q, *J* = 5.2 Hz, 1H), 3.84 (s, 3H), 3.67 (s, 6H), 3.36 (dd, *J* = 8.8, 5.6 Hz, 1H), 3.19–3.10 (m, 1H), 2.79–2.69 (m, 1H), 2.50–2.40 (m, 2H), 2.37–2.23 (m, 2H); $^{13}C$ NMR (100 MHz, $CDCl_3$) δ 177.0, 169.1, 169.0, 136.9, 134.9, 131.4, 131.1, 128.7, 128.2, 127.1, 122.8, 120.0, 119.8, 117.0, 109.2, 77.4, 52.9, 49.2, 48.7, 30.8, 30.2, 28.0. HRMS (m/z): $(M+Na)^{+}$ calcd. for $C_{26}H_{26}N_2NaO_6^{+}$, 485.1683; found, 485.1647.

**(1*R*,3*aR*,8*bR*)-Dimethyl-1-(2,5-dioxo-2,5-dihydro-1H-pyrrol-1-yl)-4,8*b*-dimethyl-1,3*a*,4,8*b*-tetrahydrocyclopenta[*b*]indole-3,3(2*H*)-dicarboxylate (3n)**

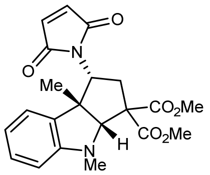


Prepared according to supplementary method C - In a dry reaction tube, a mixture of $Ni(OTf)_2$ (3.6 mg, 0.01 mmol, 5 mol%), ligand **L4** (7.9 mg, 0.012 mmol, 6 mol%), and aminocyclopropane **2c** (111.3 mg, 0.44 mmol) in DCM (3.0 mL) were stirred at room temperature for 30 minutes under the atmosphere of nitrogen. Then the mixture was cooled to 0 °C for 20 minutes and indole **1a** (29.0 mg, 0.2 mmol) in DCM (1.0 mL) was added to the mixture of catalyst via a syringe. After 36 hours, the reaction was complete (monitored by TLC). Then, the reaction was filtered through a glass funnel within layer of silica gel (100-200 mesh) and purified by flash column chromatography (Pet/EtOAc, v/v, 20:1-4:1) to give the product **3n** as a yellow solid (62.1 mg, 78% yield, 91% ee, 10:1 dr); $R_f$ = 0.56 (Pet/EtOAc 3/1, v/v); m.p.: 135.1-138.2 °C. HPLC CHIRALPAKOD-H, n-hexane/2-propanol = 80/20, flow rate 0.7 mL/min, λ = 254 nm, retention time: 16.214 min (minor), 20.535 min (major); $[\alpha]_D^{25.5}$ = +19.0 (*c* = 0.2, in $CHCl_3$). $^{1}H$ NMR (400 MHz, $CDCl_3$) δ 7.11 (td, *J* = 7.6, 1.6 Hz, 1H), 6.72–6.47 (m, 5H), 4.36–4.31 (m, 2H), 3.85–3.75 (m, 7H), 2.78 (s, 3H), 2.24 (qd, *J* = 6.0, 1.6 Hz, 1H), 1.55 (s, 3H);

$^{13}C$ NMR (150 MHz, $CDCl_3$) δ 170.8, 168.4, 154.2, 132.2, 128.7, 122.8, 118.5, 109.5, 82.5, 63.4, 59.7, 56.1, 53.0, 52.6, 39.2, 31.5, 27.5; HRMS (m/z): $(M+Na)^+$ calcd. for $C_{21}H_{22}N_2NaO_6^+$, 421.1370; found, 421.1362.

**(1*R*,3*aR,8bR*)-Dimethyl-1-(1,3-dioxoisoindolin-2-yl)-4,8*b*-dimethyl-1,3*a*,4,8*b*-tetrahydro cyclopenta[*b*]indole-3,3(2*H*)-dicarboxylate (3o)**

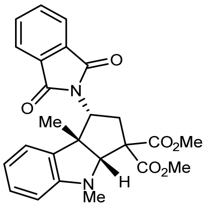


Prepared according to supplementary method C - In a dry reaction tube, a mixture of $Ni(OTf)_2$ (3.6 mg, 0.01 mmol, 5 mol%), ligand **L4** (7.9 mg, 0.012 mmol, 6 mol%), and aminocyclopropane **2b** (133.2 mg, 0.44 mmol) in DCM (3.0 mL) were stirred at room temperature for 30 minutes under the atmosphere of nitrogen. Then the mixture was cooled to 0 °C for 20 minutes and indole **1a** (29.0 mg, 0.2 mmol) in DCM (1.0 mL) was added to the mixture of catalyst via a syringe. After 24 h, the reaction was complete (monitored by TLC). Then, the reaction was filtered through a glass funnel within layer of silica gel (100-200 mesh) and purified by flash column chromatography (Pet/EtOAc, v/v, 20:1-4:1) to give the product **3o** as a yellow solid (65.4 mg, 73% yield, 56% ee, 6:1 dr); $R_f$ = 0.52 (Pet/EtOAc, 4/1, v/v); m.p.: 163.9-167.1 °C. HPLC CHIRALPAK IA, n-hexane/2-propanol = 70/30, flow rate 0.8 mL/min, λ = 254 nm, retention time: 18.613 min (minor), 10.257 min (major); $[\alpha]_D^{25.5}$ = +22.0 (*c* = 0.18, in $CHCl_3$). $^1H$ NMR (400 MHz, $CDCl_3$) δ 7.86–7.69 (m, 4H), 7.11–7.06 (m, 1H), 6.54 (d, *J* = 8.0 Hz, 1H), 6.47 (dd, *J* = 7.2, 1.2 Hz, 1H), 6.41 (td, *J* = 7.2, 0.8 Hz, 1H), 4.55 (dd, *J* = 14.4, 6.0 Hz, 1H), 4.38 (s, 1H), 4.01 (t, *J* = 13.2 Hz, 1H), 3.84 (s, 3H), 3.82 (s, 3H), 2.80 (s, 3H), 2.30 (qd, *J* = 6.4, 1.6 Hz, 1H), 1.61 (s, 3H); $^{13}C$ NMR (100 MHz, $CDCl_3$) δ 171.1, 168.6, 154.3, 134.1, 132.5, 128.8, 123.3, 122.9, 118.7, 109.4, 82.7, 63.7, 60.0, 56.4, 53.1, 52.7, 39.4, 31.8, 27.8; HRMS (m/z): $(M+Na)^+$ calcd. for $C_{25}H_{24}N_2NaO_6^+$, 471.1527; found, 471.1524.

**Dimethyl-1-(1,3-dioxoisoindolin-2-yl)-4,8*b*-dimethyl-1,3*a*,4,8*b*-tetrahydrocyclopenta[*b*]-indole-3,3(2*H*)-dicarboxylate (3o')**

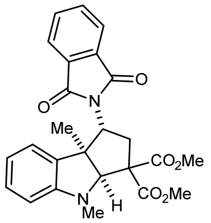


Yellow solid (10.6 mg, 10% yield); $R_f$ = 0.56 (Pet/EtOAc, 4/1, v/v); m.p.:150.9-153.6 $^oC$. $^1H$ NMR (400 MHz, $CDCl_3$) δ 7.89–7.86 (m, 2H), 7.76–7.74 (m, 2H), 7.11–7.04 (m, 2H), 6.67 (td, *J* = 7.6, 1.2 Hz, 1H), 6.37 (d, *J* = 7.6 Hz, 1H), 5.36 (dd, *J* = 10.8, 7.6 Hz, 1H), 4.54 (s, 1H), 3.82 (s, 3H), 3.47 (s, 3H), 3.39 (dd, *J* = 13.6, 10.8 Hz, 1H), 2.98 (s, 3H), 2.62 (dd, *J* = 13.6, 7.6 Hz, 1H), 1.24 (s, 3H); $^{13}C$ NMR (100 MHz, $CDCl_3$) δ 171.9, 170.2, 169.1, 149.8, 136.0, 134.3, 131.9, 128.3, 123.5, 123.0, 118.0, 106.6, 82.0, 63.5, 58.3, 56.1, 53.0, 52.5, 35.0, 33.7, 24.0; HRMS (m/z): $(M+Na)^+$ calcd. for $C_{25}H_{24}N_2NaO_6^+$, 471.1527; found, 471.1521.

**Diethyl-(1*R*,3*aR*,8*bR*)-1-(3-benzoyl-5-methyl-2,4-dioxo-3,4-dihydropyrimidin-1(2*H*)-yl)-4,8*b*-dimethyl-1,3*a*,4,8*b*-tetrahydrocyclopenta[*b*]indole-3,3(2*H*)-dicarboxylate (3p)**

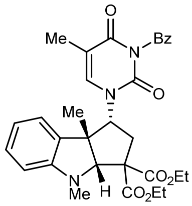


A mixture of $Cu(OTf)_2$ (7.2 mg, 0.020 mmol, 10 mol%) and, **L4** (15.8 mg, 0.024 mmol, 12 mol%), the cyclopropane **2d** (182.2 mg, 0.44 mmol) in DCM (3.0 mL) was stirred at 30 $^oC$ for 30 minutes under the atmosphere of nitrogen. Then, the mixture was cooled to room temperature for 10 minutes and the indole **1a** (29.0 mg, 0.2 mmol) in 1.0 mL DCM were added to the mixture of catalyst via a syringe. After 24 hours, the reaction was complete (monitored by TLC), the reaction was filtered through a glass funnel with thin layer of silica gel (100-200 mesh) and eluted with (Pet/EtOAc, v/v, 10:1-2:1). The filtrate was concentrated under reduced pressure. After the determination of the diatereoselectivity by $^1H$ NMR, the residue was purified by flash chromatography to give the title product **3p** as a colorless oil (90.7 mg, 81% yield, 67% ee, 1.9:1 dr); $R_f$ = 0.43 (Pet/EtOAc, 4/1, v/v). HPLC CHIRALPAK IA,

n-hexane/2-propanol = 80/20, flow rate 0.8 mL/min, λ = 254 nm, retention time: 8.223 min (minor), 13.712 min (major); $[\alpha]_D^{25.5}$ = +14.40 (*c* = 0.20, in $CHCl_3$). $^1H$ NMR (400 MHz, $CDCl_3$) δ 7.95 (d, *J* = 7.2 Hz, 2H), 7.66 (t, *J* = 7.2 Hz, 1H), 7.52 (t, *J* = 7.6 Hz, 2H), 7.16 (t, *J* = 7.6 Hz, 1H), 6.74–6.64 (m, 2H), 6.55 (d, *J* = 8.0 Hz, 1H), 6.33 (s, 1H), 5.04 (dd, *J* = 13.6, 6.4 Hz, 1H), 4.44–4.30 (m, 3H), 4.26–4.16 (m, 2H), 2.79 (s, 3H), 2.71 (t, *J* = 13.2 Hz, 1H), 2.47–2.41 (m, 1H), 1.67 (s, 3H), 1.59 (s, 3H), 1.36–1.25 (m, 6H); $^{13}C$ NMR (100 MHz, $CDCl_3$) δ 169.7, 169.1, 167.9, 162.6, 154.3, 151.0, 138.0, 135.1, 131.8, 130.5, 129.9, 129.6, 129.3, 125.3, 119.1, 109.7, 109.1, 82.3, 77.4, 62.9, 62.4, 62.0, 61.4, 56.4, 39.2, 35.1, 27.3, 14.16, 14.12, 12.3.; HRMS (m/z): $(M+Na)^+$ calcd. for $C_{31}H_{33}N_3NaO_7^+$, 582.2211; found, 582.2204.

**(1*R*,3*aR*,8*bR*)-Dimethyl-1-(4-hydrazinyl-4-oxobutanamido)-4,8*b*-dimethyl-1,3*a*,4,8*b*-tetra hydrocyclopenta[*b*]indole-3,3(2*H*)-dicarboxylate (6a)**

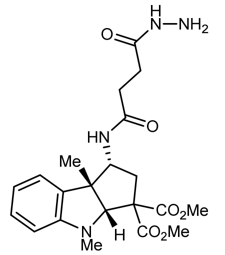


White solid, (40.2 mg, 93% yield, 99% ee); $R_f$ = 0.25 (MeOH/EtOAc, 15/1, v/v); m.p.: 188.1-189.2 °C. HPLC CHIRALPAK IA, n-hexane/2-propanol = 80/20, flow rate 0.8 mL/min, λ = 254 nm, retention time: 11.193 min (minor), 17.890 min (major); $[\alpha]_D^{25.5}$ = +59.0 (*c* = 0.15, in $CHCl_3$). $^1H$ NMR (400 MHz, $CDCl_3$) δ 7.40 (s, 1H), 7.13 (td, *J* = 7.6, 1.6 Hz, 1H), 6.92 (dd, *J* = 7.6, 1.2 Hz, 1H), 6.73 (td, *J* = 7.6, 1.2 Hz, 1H), 6.48 (d, *J* = 7.6 Hz, 1H), 6.01 (d, *J* = 9.6 Hz, 1H), 4.39–4.33 (m, 1H), 4.32–4.31 (m, 1H), 3.80 (s, 3H), 3.75 (s, 3H), 2.76 (s, 3H), 2.47–2.32 (m, 5H), 2.22–2.15 (m, 1H), 1.50 (s, 3H); $^{13}C$ NMR (150 MHz, $CDCl_3$) δ 172.9, 171.4, 171.1, 169.4, 154.1, 131.8, 128.8, 124.5, 118.7, 109.2, 82.6, 64.1, 56.82, 56.77, 53.2, 52.7, 38.9, 37.7, 31.4, 29.5, 27.5; HRMS (m/z): $(M+Na)^+$ calcd. for $C_{21}H_{28}N_4NaO_6^+$, 455.1901; found, 455.1899.

**1,3-Dimethyl-1*H*-indole (1a)**

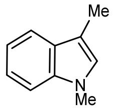


Colorless oil, $^{1}$H NMR (400 MHz, $CDCl_3$) δ 7.58 (dt, *J* = 8.0, 1.2 Hz, 1H), 7.27 (dt, *J* = 8.0, 1.2 Hz, 1H), 7.24–7.19 (m, 1H), 7.13–7.08 (m, 1H), 6.80 (d, *J* = 1.2 Hz, 1H), 3.72 (s, 3H), 2.32 (d, *J* = 1.2 Hz, 3H); $^{13}$C NMR (100 MHz, $CDCl_3$) δ 137.1, 128.8, 126.6, 121.5, 119.1, 118.6, 110.2, 109.1, 32.6, 27.1, 9.6.

# Supplementary method D : the reaction of 2-substituted indoles with aminocyclopropane

a

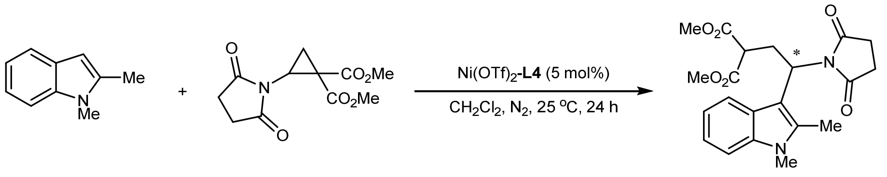


1n (±)-2a 5a
51% yield, 46% ee

b

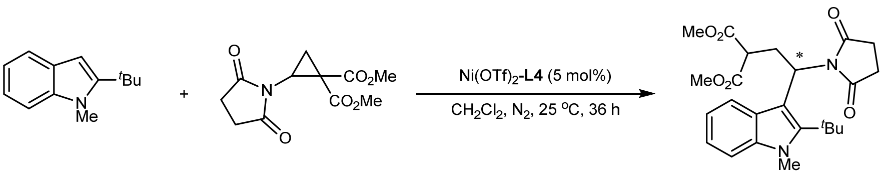


1o (±)-2a 5b
53% yield, 97% ee

**Supplementary Fig. 5 Reaction of 2-substituted indoles. a** Reaction with 2-methyl indole. **b** Reaction with 2-*tert*-butyl indole.

In a dry reaction tube, a mixture of $Ni(OTf)_2$ (3.6 mg, 0.01 mmol, 5 mol%), ligand **L4** (7.9 mg, 0.012 mmol, 6 mol%), and aminocyclopropane **2a** (0.44 mmol) in DCM (3.0 mL) were stirred at room temperature for 30 minutes under the atmosphere of nitrogen. Then indole substrate **1n-1o** (0.2 mmol) in DCM (1.0 mL) was added to the mixture of catalyst via a syringe. After 24-36 h, the reaction was complete (monitored by TLC). Then, the reaction was filtered through a glass funnel within layer of silica gel (100-200 mesh) and purified by flash column chromatography to give the product **5a**-**5b**.

### Dimethyl-2-(2-(1,2-dimethyl-1*H*-indol-3-yl)-2-(2,5-dioxopyrrolidin-1-yl)ethyl)malonate (5a)

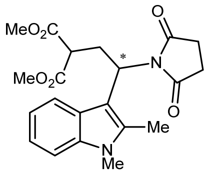

Prepared according to supplementary method D - A mixture of $Ni(OTf)_2$ (3.6 mg, 0.010 mmol, 5 mol%) and, **L4** (7.86 mg, 0.012 mmol, 6 mol%), the cyclopropane **2a** (112.2 mg, 0.44 mmol) in DCM (3.0 mL) was stirred at 30 °C for 30 minutes under the atmosphere of nitrogen. Then, the

mixture was cooled to room temperature for 10 minutes and the indole **1n** (29.0 mg, 0.2 mmol) in 1.0 mL DCM were added to the mixture of catalyst via a syringe. After 24 hours, the reaction was complete (monitored by TLC), the reaction was filtered through a glass funnel with thin layer of silica gel (100-200 mesh) and eluted with (Pet/EtOAc, v/v, 10:1-2:1). The filtrate was concentrated under reduced pressure. After the determination of the diatereoselectivity by $^1H$ NMR, the residue was purified by flash chromatography to give the title product **5a** as a white solid (40.8 mg, 51% yield, 46% ee); $R_f$ = 0.51 (Pet/EtOAc, 4/1, v/v). m.p.: 178.8-181.2 $^oC$, HPLC CHIRALPAK IE, n-hexane/2-propanol = 60/40, flow rate 0.8 mL/min, λ = 254 nm, retention time: 20.032 min (minor), 22.505 min (major); $[\alpha]_D^{25.5}$ = +6.40 (*c* = 0.20, in $CHCl_3$). $^1H$ NMR (400 MHz, $CDCl_3$) δ 8.02 (d, *J* = 7.6 Hz, 1H), 7.25–7.21 (m, 1H), 7.18–7.14 (m, 1H), 7.13–7.08 (m, 1H), 5.58–5.52 (m, 1H), 3.73 (s, 3H), 3.64 (s, 3H), 3.63 (s, 3H), 3.33–3.24 (m, 2H), 3,13–3.03 (m, 1H), 2.59 (s, 4H), 2.50 (s, 3H); $^{13}C$ NMR (100 MHz, $CDCl_3$) δ 177.5, 169.4, 169.3, 136.9, 136.8, 126.1, 121.1, 120.5, 119.7, 108.7, 107.7, 52.8, 52.7, 50.0, 47.6, 29.7, 29.6, 28.2, 11.0; HRMS (m/z): $(M+Na)^+$ calcd. for $C_{21}H_{24}N_2NaO_6^+$, 423.1527; found, 423.1519.

**Dimethyl-2-(2-(2-(*tert*-butyl)-1-methyl-1*H*-indol-3-yl)-2-(2,5-dioxopyrrolidin-1-yl)ethyl) malonate (5b)**

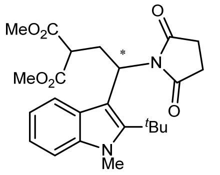


Prepared according to supplementary method D - A mixture of $Ni(OTf)_2$ (3.6 mg, 0.010 mmol, 5 mol%) and, **L4** (7.86 mg, 0.012 mmol, 6 mol%), the cyclopropane **2a** (112.2 mg, 0.44 mmol) in DCM (3.0 mL) was stirred at 30 $^oC$ for 30 minutes under the atmosphere of nitrogen. Then, the mixture was cooled to room temperature for 10 minutes and the indole **1o** (34.6 mg, 0.2 mmol) in 1.0 mL DCM were added to the mixture of catalyst via a syringe. After 36 hours, the reaction was complete (monitored by TLC), the reaction was filtered through a glass funnel with thin layer of silica gel (100-200 mesh) and eluted with (Pet/EtOAc, v/v, 10:1-2:1). The filtrate was concentrated under reduced pressure. After the determination of the diatereoselectivity by $^1H$ NMR, the residue was purified by flash chromatography to give the title product **5b** as a

colorless oil (47.0 mg, 53% yield, 97% ee,); $R_f$ = 0.45 (Pet/EtOAc, 3/1, v/v). HPLC CHIRALPAK IA, n-hexane/2-propanol = 80/20, flow rate 0.8 mL/min, λ = 254 nm, retention time: 13.293 min (minor), 11.738 min (major); $[\alpha]_D^{25.5}$ = +8.90 (*c* = 0.20, in $CHCl_3$). Colorless oil, $^1H$ NMR (400 MHz, $CDCl_3$) δ 8.01 (d, *J* = 8.0 Hz, 1H), 7.24–7.15 (m, 2H), 7.07 (t, *J* = 7.6 Hz, 1H), 6.03 (dd, *J* = 12.0, 5.2 Hz, 1H), 3.86 (s, 3H), 3.77–3.71 (m, 4H), 3.68 (s, 3H), 3.62–3.57 (m, 1H), 2.68–2.62 (m, 1H), 2.59 (s, 4H), 1.66 (s, 9H); $^{13}C$ NMR (100 MHz, $CDCl_3$) δ 178.7, 169.8, 169.2, 144.7, 138.3, 125.8, 121.6, 120.4, 119.7, 110.0, 108.8, 52.8, 52.7, 50.3, 49.9, 34.9, 34.1, 31.6, 31.0, 28.4; HRMS (m/z): $(M+Na)^+$ calcd. for $C_{24}H_{30}N_2NaO_6^+$, 465.1996; found, 465.1992.

## X-ray data of L3, L4, L8, L13, PyIPI L1-Cu, 3a, and ±3o′

**Supplementary Fig. 6** X-Ray crystal structure of **L3** (The crystal was obtained by slow evaporation of **L3**, in a mixture of ethyl acetate/petroleum ether). (CCDC: 2203566 )

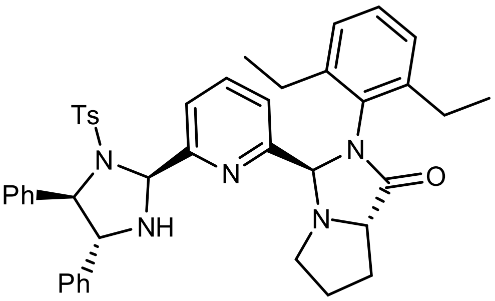


**L3**

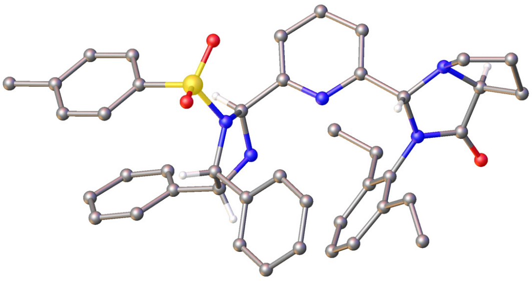

**L3**

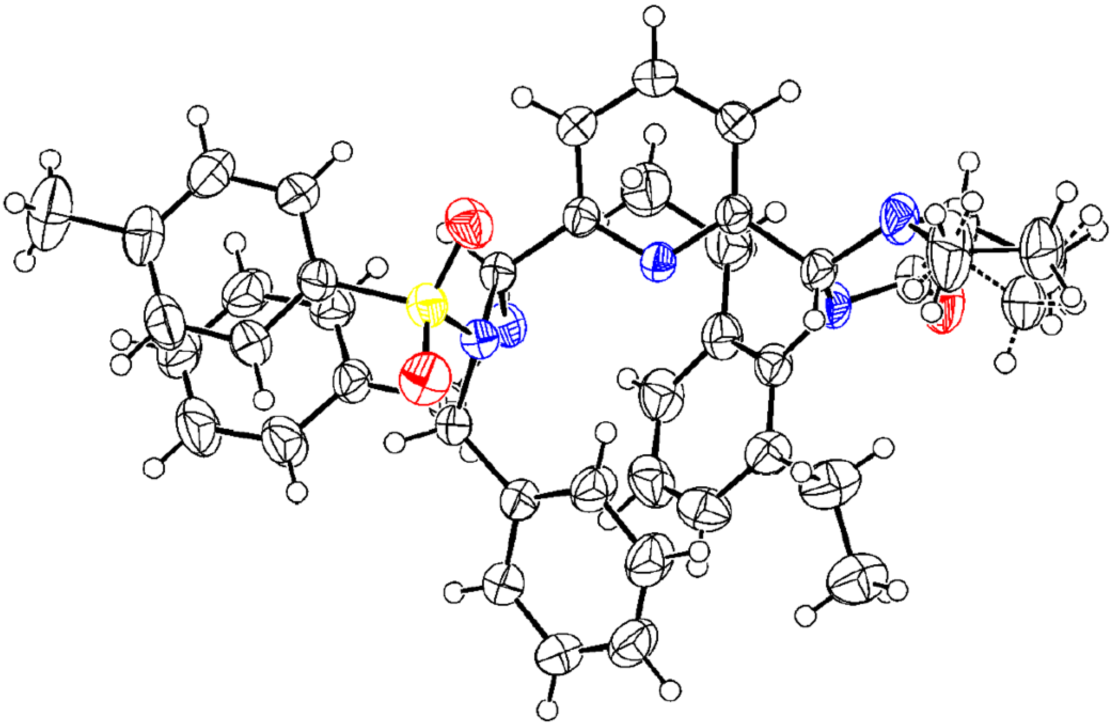

**L3**

**Supplementary Table 6. Crystal data and structure refinement for L3**

| | |
|---|---|
| Identification code | L3 |
| Empirical formula | $C_{43}H_{45}N_5O_3S$ |
| Formula weight | 711.90 |
| Temperature/K | 293(2) |
| Crystal system | orthorhombic |
| Space group | $P2_12_12_1$ |
| a/Å | 7.98020(10) |
| b/Å | 12.33920(10) |
| c/Å | 37.3080(3) |
| α/° | 90 |
| β/° | 90 |
| γ/° | 90 |
| Volume/Å$^3$ | 3673.69(6) |
| Z | 4 |
| $\rho_{calc}$g/cm$^3$ | 1.287 |
| μ/mm$^{-1}$ | 1.159 |
| F(000) | 1512.0 |
| Crystal size/mm$^3$ | 0.2 × 0.15 × 0.1 |
| Radiation | Cu Kα (λ = 1.54184) |
| 2Θ range for data collection/° | 7.546 to 143.086 |
| Index ranges | -9 ⩽ h ⩽ 9, -15 ⩽ k ⩽ 15, -45 ⩽ l ⩽ 45 |
| Reflections collected | 60643 |
| Independent reflections | 7130 [$R_{int}$ = 0.0756, $R_{sigma}$ = 0.0301] |
| Data/restraints/parameters | 7130/42/482 |
| Goodness-of-fit on F$^2$ | 1.066 |
| Final R indexes [I>=2σ (I)] | $R_1$ = 0.0483, w$R_2$ = 0.1312 |
| Final R indexes [all data] | $R_1$ = 0.0499, w$R_2$ = 0.1336 |
| Largest diff. peak/hole / e Å$^{-3}$ | 0.52/-0.36 |
| Flack parameter | 0.015(7) |

**Supplementary Fig. 7** X-Ray crystal structure of **L4** (The crystal was obtained by slow evaporation of **L4**, in diethyl ether). (CCDC: 2203567)

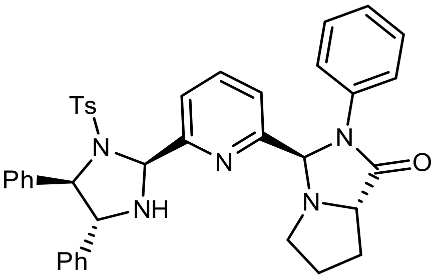


**L4**

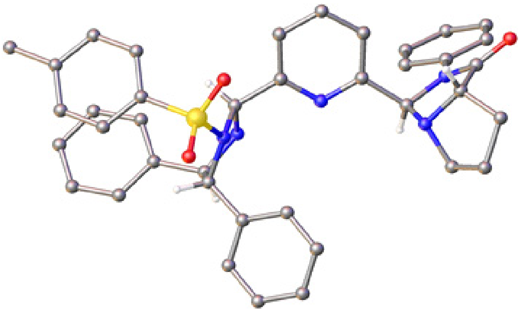

**L4**

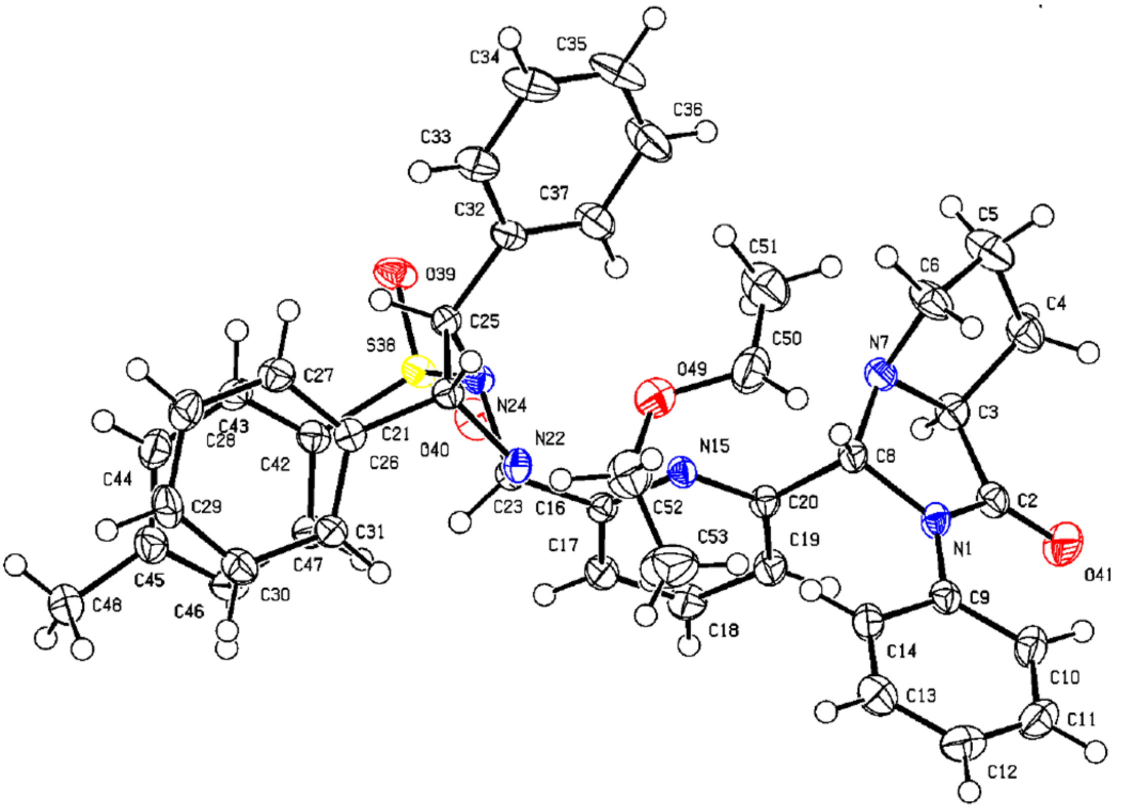


**L4**

**Supplementary Table 7. Crystal data and structure refinement for L4**

| | |
|---|---|
| Identification code | L4 |
| Empirical formula | $C_{43}H_{45}N_5O_4S$ |
| Formula weight | 727.93 |
| Temperature/K | 293 |
| Crystal system | monoclinic |
| Space group | $P2_1$ |
| a/Å | 9.22760(10) |
| b/Å | 10.04110(10) |
| c/Å | 20.5178(2) |
| α/° | 90 |
| β/° | 98.6650(10) |
| γ/° | 90 |
| Volume/Å$^3$ | 1879.38(3) |
| Z | 2 |
| $\rho_{calc}$g/cm$^3$ | 1.2862 |
| μ/mm$^{-1}$ | 1.165 |
| F(000) | 774.9 |
| Crystal size/mm$^3$ | 0.16 × 0.08 × 0.07 |
| Radiation | Cu Kα (λ = 1.54184) |
| 2Θ range for data collection/° | 8.72 to 143.16 |
| Index ranges | -11 ≤ h ≤ 11, -12 ≤ k ≤ 12, -25 ≤ l ≤ 25 |
| Reflections collected | 47639 |
| Independent reflections | 7069 [$R_{int}$ = 0.0509, $R_{sigma}$ = 0.0293] |
| Data/restraints/parameters | 7069/1/481 |
| Goodness-of-fit on F$^2$ | 1.036 |
| Final R indexes [I>=2σ (I)] | $R_1$ = 0.0447, w$R_2$ = 0.1174 |
| Final R indexes [all data] | $R_1$ = 0.0473, w$R_2$ = 0.1201 |
| Largest diff. peak/hole / e Å$^{-3}$ | 0.53/-0.34 |
| Flack parameter | 0.006(13) |

**Supplementary Fig. 8** X-Ray crystal structure of **L8** (The crystal was obtained by slow evaporation of **L8**, in a mixture of ethyl acetate/petroleum ether). (CCDC: 2203568)

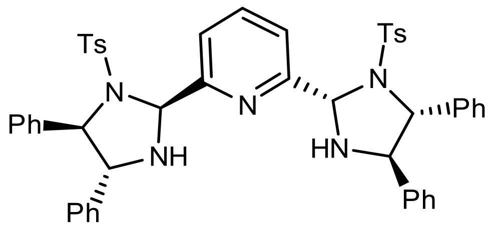


**L8**

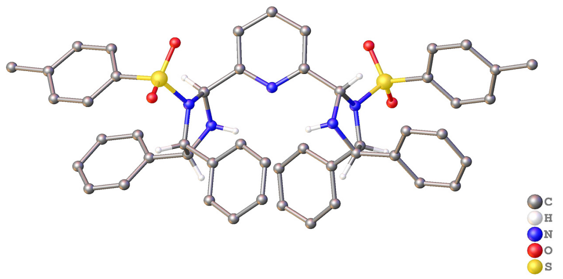


**L8**

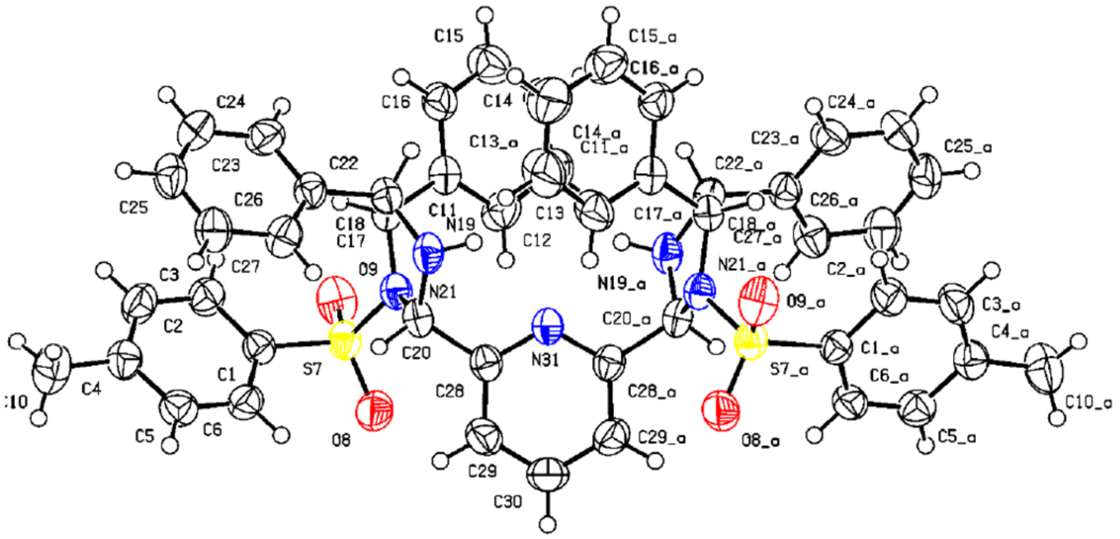

**L8**

**Supplementary Table 8. Crystal data and structure refinement for L8**

| | |
|---|---|
| Identification code | L8 |
| Empirical formula | $C_{24.5}H_{22.5}N_{2.5}O_2S$ |
| Formula weight | 416.03 |
| Temperature/K | 293 |
| Crystal system | orthorhombic |
| Space group | $P2_12_12$ |
| a/Å | 22.9404(2) |
| b/Å | 10.24030(10) |
| c/Å | 9.06350(10) |
| α/° | 90 |
| β/° | 90 |
| γ/° | 90 |
| Volume/Å$^3$ | 2129.17(4) |
| Z | 4 |
| $\rho_{calc}$g/cm$^3$ | 1.2978 |
| μ/mm$^{-1}$ | 1.546 |
| F(000) | 879.8 |
| Crystal size/mm$^3$ | 0.15 × 0.08 × 0.05 |
| Radiation | Cu Kα (λ = 1.54184) |
| 2Θ range for data collection/° | 7.7 to 142.76 |
| Index ranges | -28 ≤ h ≤ 28, -12 ≤ k ≤ 12, -11 ≤ l ≤ 11 |
| Reflections collected | 32044 |
| Independent reflections | 4144 [$R_{int}$ = 0.0384, $R_{sigma}$ = 0.0184] |
| Data/restraints/parameters | 4144/0/277 |
| Goodness-of-fit on $F^2$ | 1.074 |
| Final R indexes [I>=2σ (I)] | $R_1$ = 0.0290, $wR_2$ = 0.0764 |
| Final R indexes [all data] | $R_1$ = 0.0297, $wR_2$ = 0.0771 |
| Largest diff. peak/hole / e Å$^{-3}$ | 0.17/-0.20 |
| Flack parameter | -0.017(11) |

**Supplementary Fig. 9** X-Ray crystal structure of **L13** (The crystal was obtained by slow evaporation of **L13**, in a mixture of $CH_2Cl_2$/petroleum ether). (CCDC: 2203569)

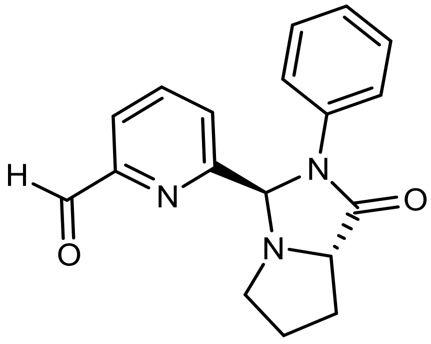


**L13**

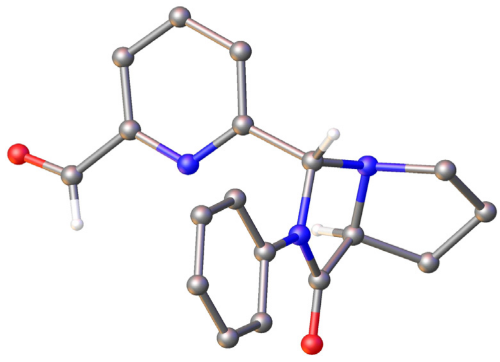

**L13**

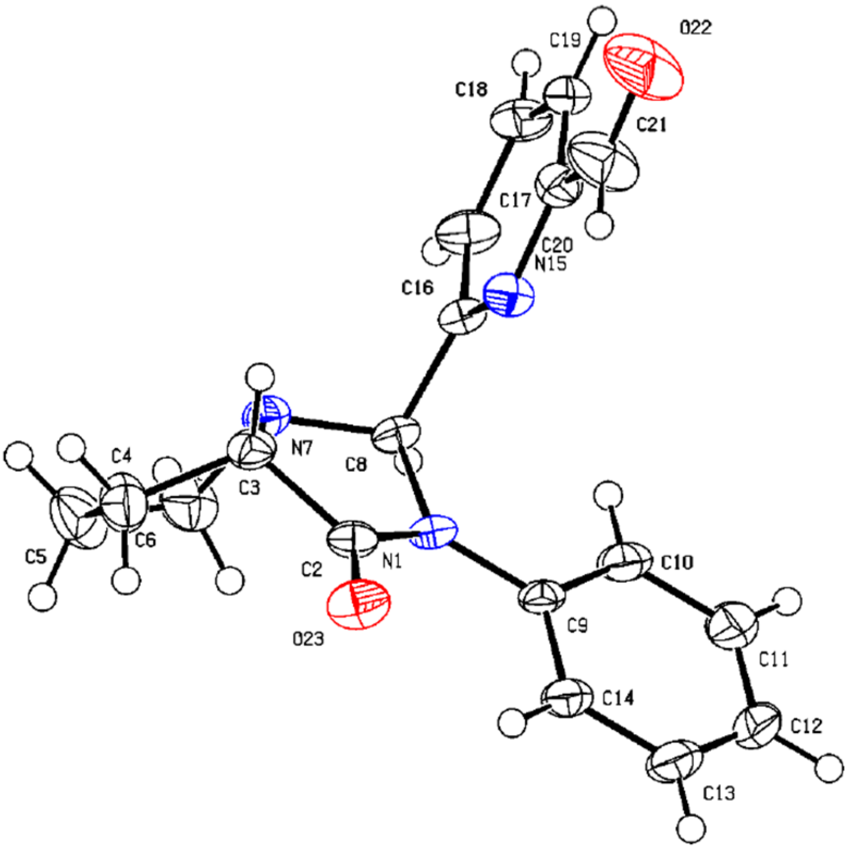


**L13**

**Supplementary Table 9. Crystal data and structure refinement for L13**

| | |
|---|---|
| Identification code | L13 |
| Empirical formula | $C_{18}H_{17}N_3O_2$ |
| Formula weight | 307.34 |
| Temperature/K | 293 |
| Crystal system | monoclinic |
| Space group | $P2_1$ |
| a/Å | 6.68630(10) |
| b/Å | 15.7601(2) |
| c/Å | 7.46670(10) |
| α/° | 90 |
| β/° | 104.3260(10) |
| γ/° | 90 |
| Volume/$Å^3$ | 762.349(18) |
| Z | 2 |
| $\rho_{calc}g/cm^3$ | 1.339 |
| $\mu/mm^{-1}$ | 0.723 |
| F(000) | 324.0 |
| Crystal size/$mm^3$ | 0.13 × 0.07 × 0.06 |
| Radiation | CuKα (λ = 1.54184) |
| 2Θ range for data collection/° | 11.228 to 142.636 |
| Index ranges | $-8 \leqslant h \leqslant 8, -19 \leqslant k \leqslant 19, -9 \leqslant l \leqslant 9$ |
| Reflections collected | 17372 |
| Independent reflections | 2942 [$R_{int}$ = 0.0718, $R_{sigma}$ = 0.0283] |
| Data/restraints/parameters | 2942/1/208 |
| Goodness-of-fit on $F^2$ | 1.045 |
| Final R indexes [I>=2σ (I)] | $R_1$ = 0.0443, $wR_2$ = 0.1145 |
| Final R indexes [all data] | $R_1$ = 0.0444, $wR_2$ = 0.1147 |
| Largest diff. peak/hole / e $Å^{-3}$ | 0.27/-0.35 |
| Flack parameter | -0.03(18) |

**Supplementary Fig. 10** X-ray crystal structure of **PyIPI L1-Cu** complex (The crystal was obtained by slow evaporation of **PyIPI L1-Cu**, in a mixture of THF/$Et_2O$). (CCDC: 2203570)

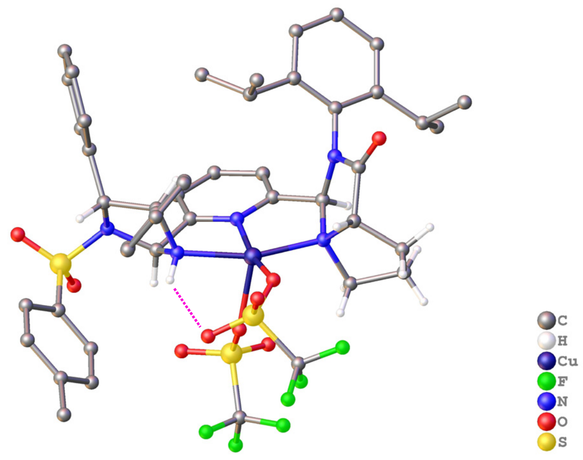


**Cu(OTf)$_2$-PyIPI-L1**

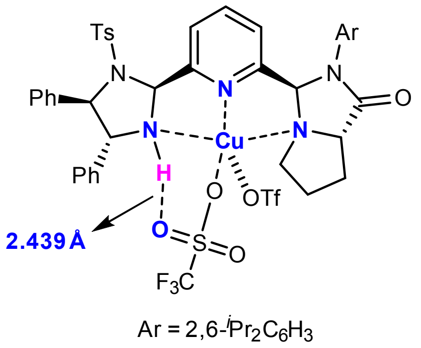


**Cu(OTf)$_2$-PyIPI-L1**

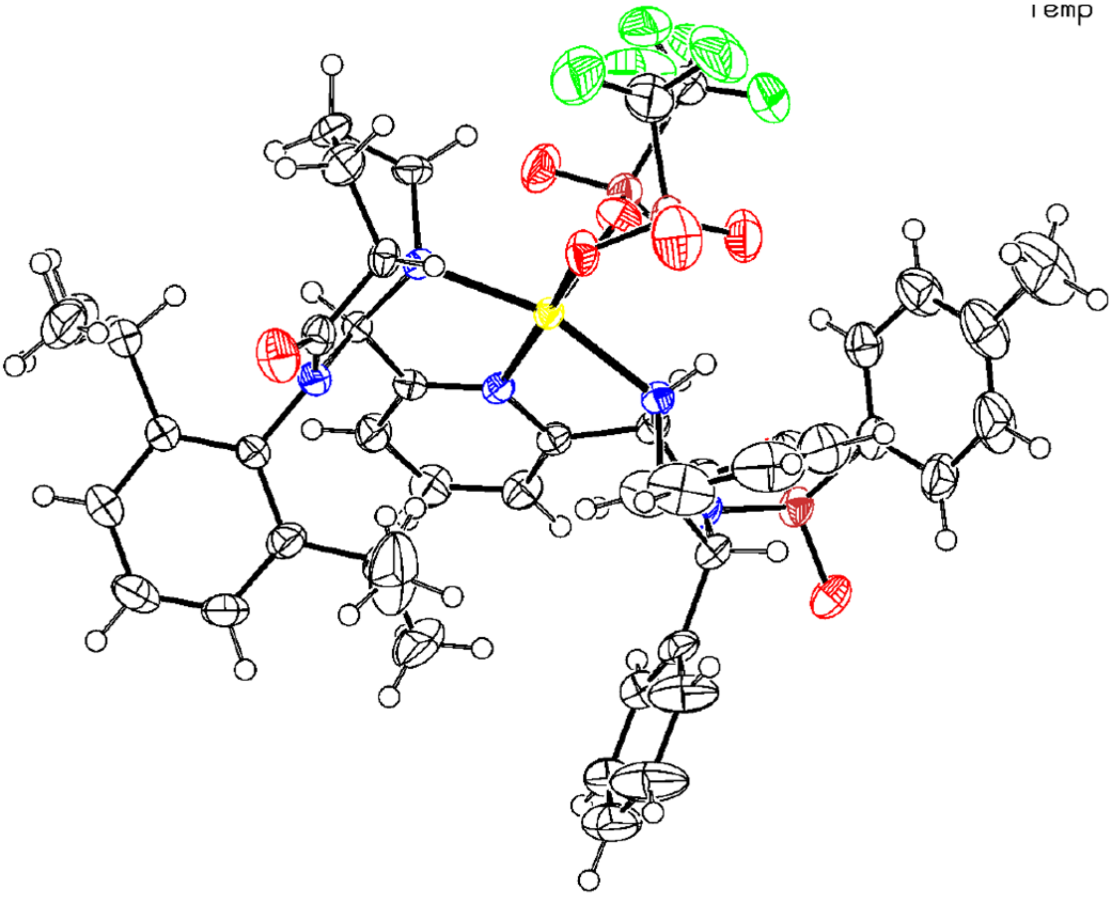

**PyIPI L1-Cu**

**Supplementary Table 10. Crystal data and structure refinement for $Cu(OTf)_2$-PyIPI-L1**

| | |
|---|---|
| Identification code | $Cu(OTf)_2$-PyIPI-L1 |
| Empirical formula | $C_{47}H_{49}CuF_6N_5O_9S_3$ |
| Formula weight | 1101.63 |
| Temperature/K | 293 |
| Crystal system | orthorhombic |
| Space group | $P2_12_12_1$ |
| a/Å | 13.09960(10) |
| b/Å | 15.45880(10) |
| c/Å | 28.3615(2) |
| α/° | 90 |
| β/° | 90 |
| γ/° | 90 |
| Volume/Å$^3$ | 5743.32(7) |
| Z | 4 |
| $\rho_{calc}$g/cm$^3$ | 1.274 |
| μ/mm$^{-1}$ | 2.175 |
| F(000) | 2276.0 |
| Crystal size/mm$^3$ | 0.2 × 0.09 × 0.08 |
| Radiation | CuKα (λ = 1.54184) |
| 2Θ range for data collection/° | 7.434 to 143.17 |
| Index ranges | -15 ⩽ h ⩽ 16, -19 ⩽ k ⩽ 19, -34 ⩽ l ⩽ 34 |
| Reflections collected | 99633 |
| Independent reflections | 11144 [$R_{int}$ = 0.0557, $R_{sigma}$ = 0.0268] |
| Data/restraints/parameters | 11144/0/652 |
| Goodness-of-fit on $F^2$ | 1.022 |
| Final R indexes [I>=2σ (I)] | $R_1$ = 0.0300, $wR_2$ = 0.0787 |
| Final R indexes [all data] | $R_1$ = 0.0311, $wR_2$ = 0.0793 |
| Largest diff. peak/hole / e Å$^{-3}$ | 0.27/-0.28 |
| Flack parameter | 0.002(5) |

**Supplementary Fig. 11** X-Ray crystal structure of **3a** (The crystal was obtained by slow evaporation of **3a**，in a mixture of ethyl acetate/petroleum ether). (CCDC: 2203571)

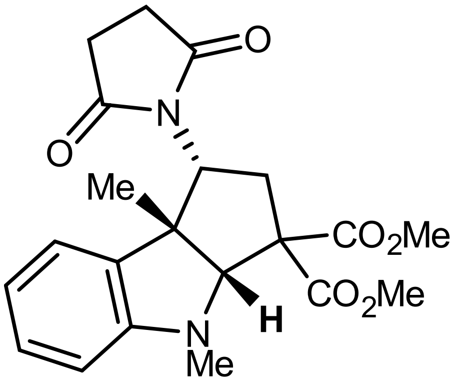


**3a**

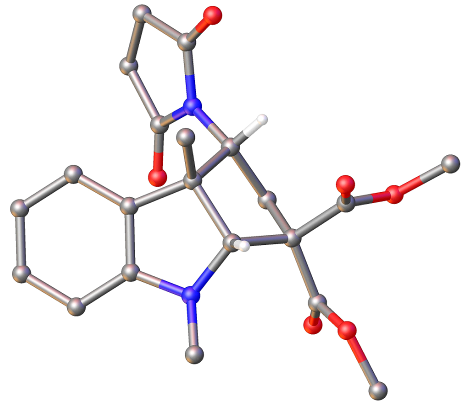

**3a**

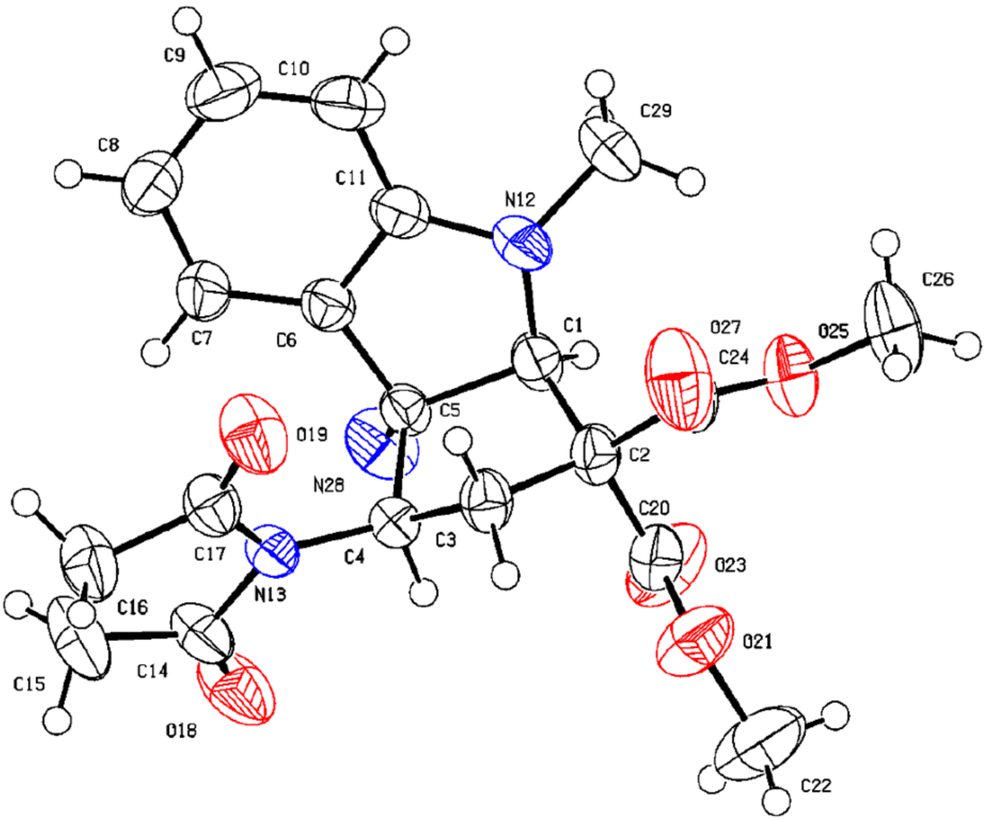


**3a**

**Supplementary Table 11. Crystal data and structure refinement for 3a.**

| | |
|---|---|
| Identification code | 3a |
| Empirical formula | $C_{20}H_{21}N_3O_6$ |
| Formula weight | 399.41 |
| Temperature/K | 293 |
| Crystal system | orthorhombic |
| Space group | $P2_12_12_1$ |
| a/Å | 10.84620(10) |
| b/Å | 12.86500(10) |
| c/Å | 14.58870(10) |
| α/° | 90 |
| β/° | 90 |
| γ/° | 90 |
| Volume/Å$^3$ | 2035.65(3) |
| Z | 4 |
| $\rho_{calc}$g/cm$^3$ | 1.3031 |
| μ/mm$^{-1}$ | 0.816 |
| F(000) | 843.0 |
| Crystal size/mm$^3$ | 0.15 × 0.08 × 0.07 |
| Radiation | Cu Kα (λ = 1.54184) |
| 2Θ range for data collection/° | 9.16 to 143.12 |
| Index ranges | -11 ≤ h ≤ 13, -15 ≤ k ≤ 15, -17 ≤ l ≤ 17 |
| Reflections collected | 26363 |
| Independent reflections | 3943 [$R_{int}$ = 0.0449, $R_{sigma}$ = 0.0222] |
| Data/restraints/parameters | 3943/0/265 |
| Goodness-of-fit on $F^2$ | 1.066 |
| Final R indexes [I>=2σ (I)] | $R_1$ = 0.0474, $wR_2$ = 0.1303 |
| Final R indexes [all data] | $R_1$ = 0.0492, $wR_2$ = 0.1328 |
| Largest diff. peak/hole / e Å$^{-3}$ | 0.43/-0.31 |
| Flack parameter | 0.0(2) |

**Supplementary Fig. 12** X-Ray crystal structure of **3o´** (The crystal was obtained by slow evaporation of **3o´** in a mixture of $CH_2Cl_2$/petroleum ether). (CCDC: 2203572)

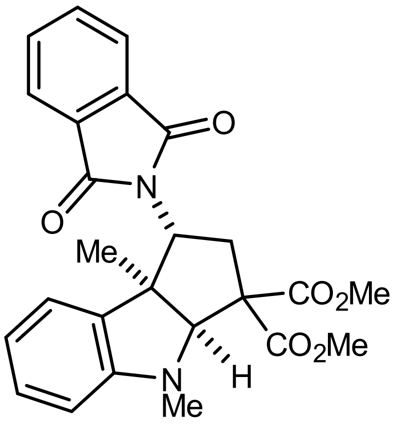


**3o′**

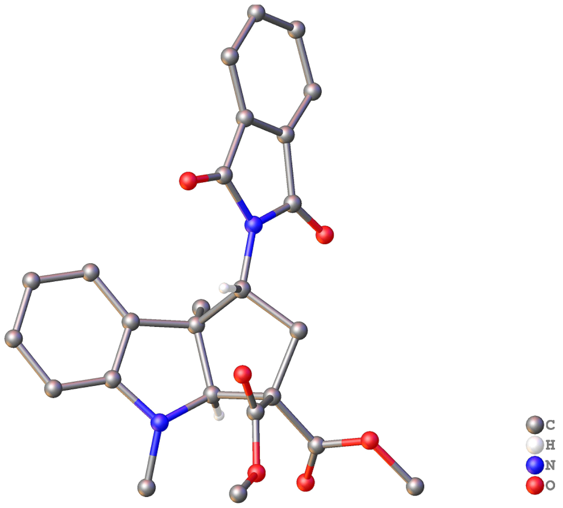


± **3o′**

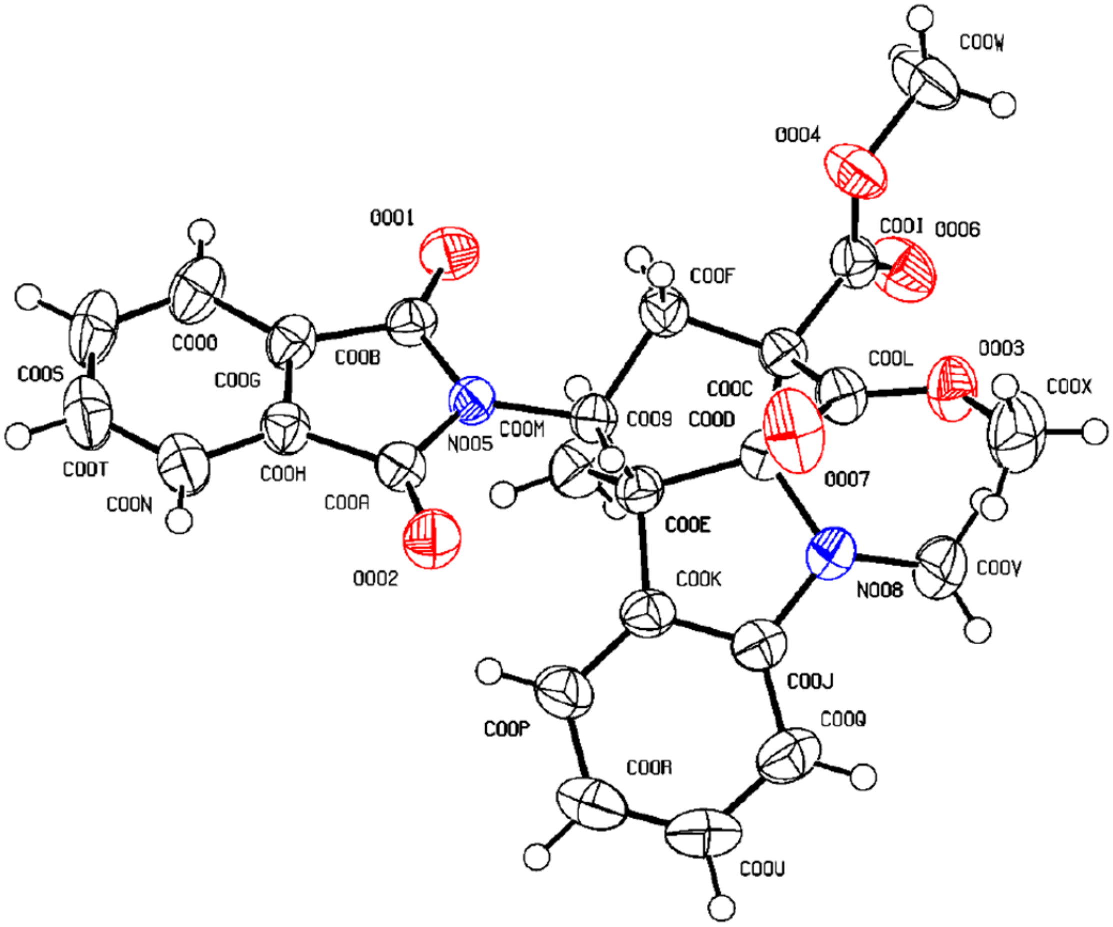


**±3o´**

**Supplementary Table 12. Crystal data and structure refinement for ±3o′.**

| | |
|---|---|
| Identification code | ±3o′ |
| Empirical formula | $C_{25}H_{24}N_2O_6$ |
| Formula weight | 448.48 |
| Temperature/K | 293 |
| Crystal system | orthorhombic |
| Space group | Pbca |
| a/Å | 8.33790(10) |
| b/Å | 15.3323(2) |
| c/Å | 35.5438(4) |
| α/° | 90 |
| β/° | 90 |
| γ/° | 90 |
| Volume/Å$^3$ | 4543.89(10) |
| Z | 8 |
| $\rho_{calc}$g/cm$^3$ | 1.3110 |
| μ/mm$^{-1}$ | 0.780 |
| F(000) | 1894.6 |
| Crystal size/mm$^3$ | 0.15 × 0.08 × 0.06 |
| Radiation | Cu Kα (λ = 1.54184) |
| 2Θ range for data collection/° | 9.96 to 142.88 |
| Index ranges | -9 ≤ h ≤ 4, -18 ≤ k ≤ 16, -43 ≤ l ≤ 42 |
| Reflections collected | 12280 |
| Independent reflections | 4301 [$R_{int}$ = 0.0256, $R_{sigma}$ = 0.0273] |
| Data/restraints/parameters | 4301/0/302 |
| Goodness-of-fit on $F^2$ | 1.055 |
| Final R indexes [I>=2σ (I)] | $R_1$ = 0.0567, $wR_2$ = 0.1514 |
| Final R indexes [all data] | $R_1$ = 0.0637, $wR_2$ = 0.1582 |
| Largest diff. peak/hole / e Å$^{-3}$ | 0.26/-0.28 |

**Supplementary Fig. 13 HSQC of L4**

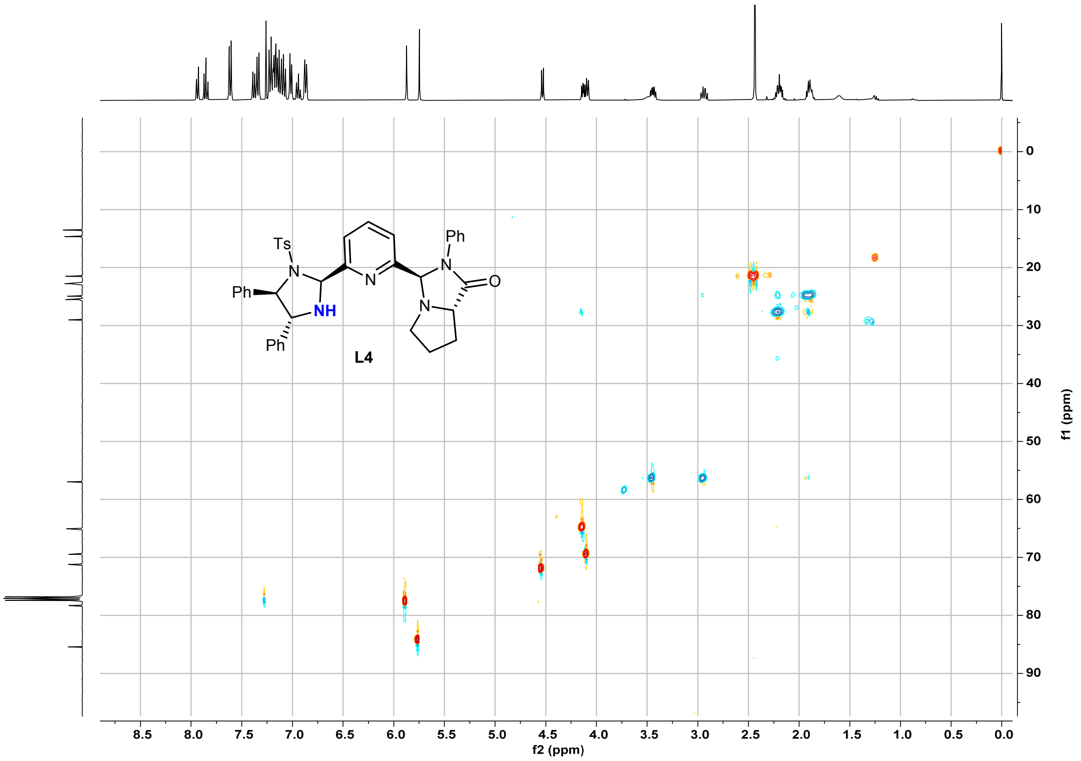


# Partial enlargement

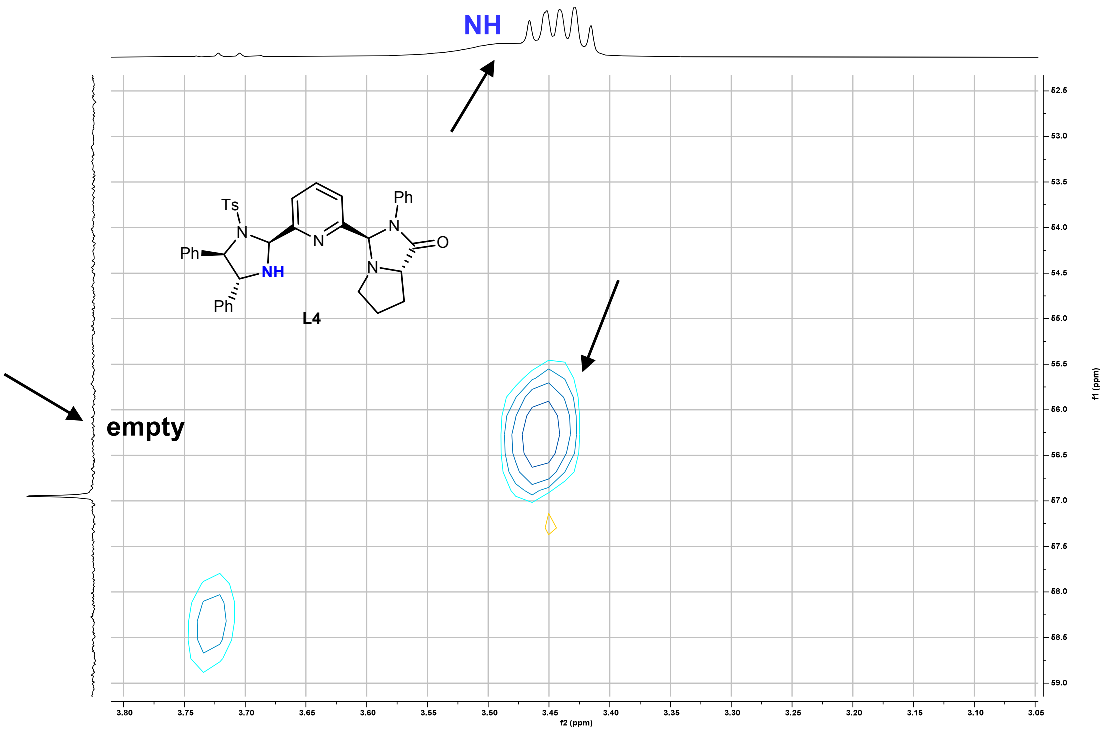

## Scale-up reaction and recrystallization

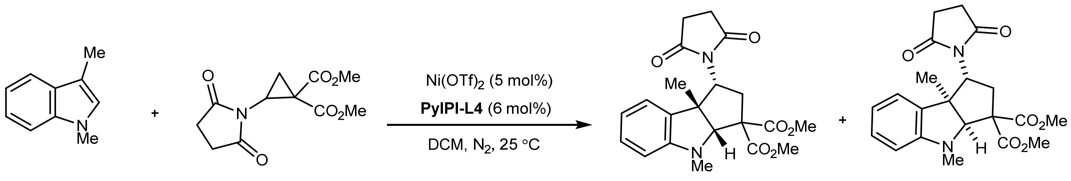


| 1a | (±) 2a | 3a | 3a′ |
|---|---|---|---|
| 4 mmol<br>0.58 g | 8.8 mmol<br>2.24 g | 1.12 g 70% yield, 96% ee,<br>(1.01 g 63% yield, 99% ee<br>after recrystallization) | 98 mg, 6.0% yield<br>94% ee |

In a 250.0 mL round bottom flask, a mixture of $Ni(OTf)_2$ (71.4 mg, 0.2 mmol, 5 mol%), ligand **L4** (157.2 mg, 0.24 mmol, 6 mol%), and aminocyclopropane **2a** (2.24 g, 8.8 mmol) in DCM (75.0 mL) were stirred at room temperature for 30 minutes under the atmosphere of nitrogen. Then indole **1a** (580.0 mg, 4.0 mmol) in DCM (25.0 mL) was added to the mixture of catalyst via a syringe. After 24 h, the reaction was complete (monitored by TLC). Then, the reaction was filtered through a glass funnel within layer of silica gel (100-200 mesh) and purified by flash chromatography to give the product **3a**′ (94% ee, 98.0 mg, 6.0% yield) firstly. After that, products **3a** and **4a** were obtained by flash chromatography as a mixture (colorless oil, 1.4 g).

In order to separate products **3a** and **4a**, in a mixture of **3a** and **4a** in THF, NaH (1.5 equiv as 60% dispersion in mineral oil) was added at 0 °C and stirred for 15 min. Subsequently, $CH_3I$ (0.7 mL, 2.0 equiv) was added dropwise and the reaction was stirred at 0 °C for another 10 min. The reaction was then warmed to room temperature and stirred for 6 h. After that, the reaction mixture was quenched with distilled water. The reaction mixture was then concentrated in vacuo to remove THF. The aqueous layer was washed for 3 times with dichloromethane. The organic layer was dried over $Na_2SO_4$ then filtered, concentrated, and purified by flash column chromatography to give the product **3a** as a white solid (1.12 g, 70% yield). After recrystallization from ethyl acetate and dichloromethane, product **3a** was obtained in 1.01 g (63% yield).

HPLC CHIRALPAK IA, n-hexane/2-propanol = 80/20, flow rate 0.8 mL/min, λ = 254 nm, retention time: 20.337 min (minor) , 13.035 min (major).

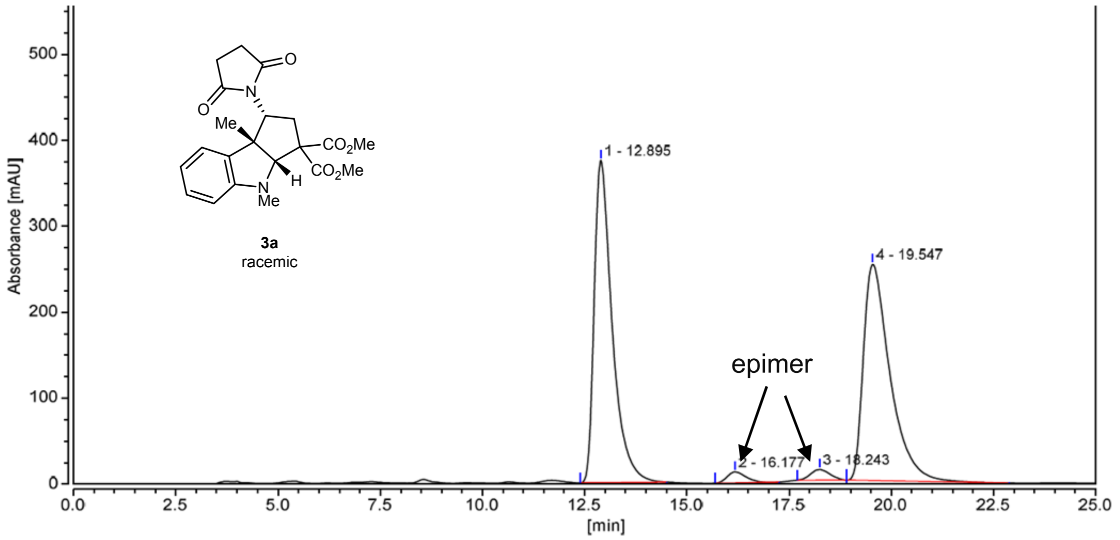


| Peak | Retention Time min | Area mAU*min | Height mAU | Area % | Height % |
|---|---|---|---|---|---|
| 1 | 12.895 | 190.941 | 375.144 | 48.41 | 57.52 |
| 2 | 16.177 | 6.615 | 12.633 | 1.68 | 1.94 |
| 3 | 18.243 | 6.906 | 12.471 | 1.75 | 1.91 |
| 4 | 19.547 | 189.987 | 251.946 | 48.17 | 38.63 |
| **Total:** | | **394.448** | **652.193** | **100.00** | **100.00** |

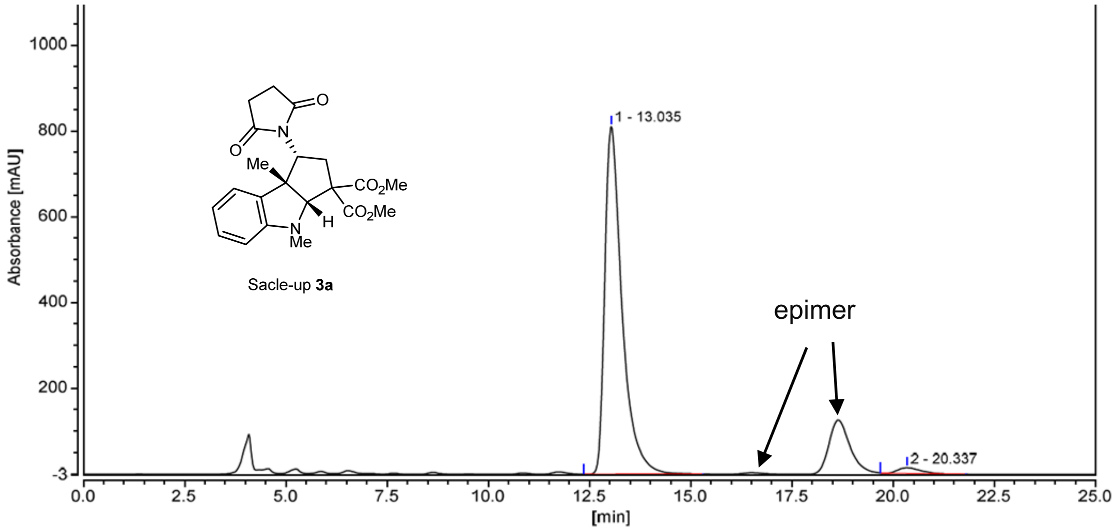


| Peak | Retention Time min | Area mAU*min | Height mAU | Area % | Height % |
|---|---|---|---|---|---|
| 1 | 13.035 | 398.702 | 810.221 | 98.03 | 98.43 |
| 2 | 20.337 | 8.021 | 12.915 | 1.97 | 1.57 |
| **Total:** | | **406.722** | **823.136** | **100.00** | **100.00** |

**Supplementary Fig. 14** HPLC spectra of scale-up **3a**

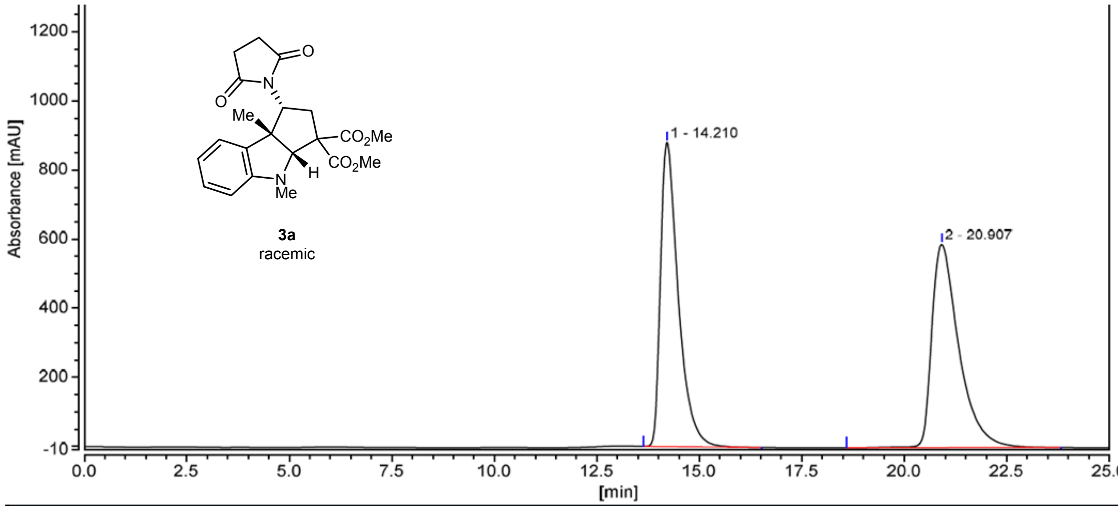


| Peak | Retention Time min | Area mAU*min | Height mAU | Area % | Height % |
|---|---|---|---|---|---|
| 1 | 14.210 | 442.522 | 880.088 | 49.60 | 59.96 |
| 2 | 20.907 | 449.675 | 587.768 | 50.40 | 40.04 |
| **Total:** | | **892.197** | **1467.856** | **100.00** | **100.00** |

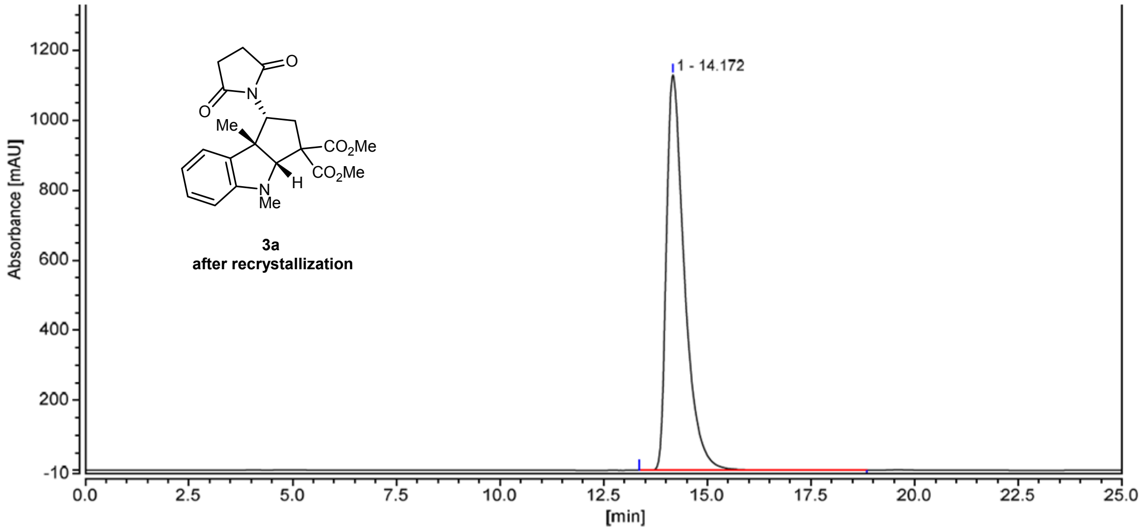


| Peak | Retention Time min | Area mAU*min | Height mAU | Area % | Height % |
|---|---|---|---|---|---|
| 1 | 14.172 | 571.639 | 1129.212 | 100.00 | 100.00 |
| **Total:** | | **571.639** | **1129.212** | **100.00** | **100.00** |

**Supplementary Fig. 15** HPLC spectra of **3a** after recrystallization

## Kinetic resolution experiment of aminocyclopropane 2a

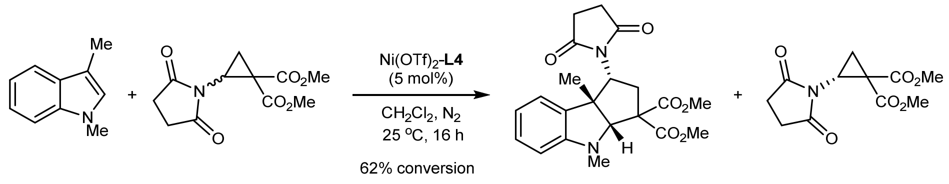

**1a**
0.67 equiv

(±)-**2a**
0.3 mmol

**3a**
40% yield, 8:1 dr, 95% ee

*R*-**2a**
27% yield, 87% ee

In a dry reaction tube, a mixture of $Ni(OTf)_2$ (5.4 mg, 0.015 mmol, 5 mol%), ligand **L4** (11.8 mg, 0.018 mmol, 6 mol%), and aminocyclopropane **2a** (76.5 mg, 0.3 mmol) in DCM (3.0 mL) were stirred at room temperature for 30 minutes under the atmosphere of nitrogen. Then indole **1a** (29.0 mg, 0.2 mmol) in DCM (1.0 mL) was added to the mixture of catalyst via a syringe. After 24 h, the reaction was complete (monitored by TLC). Then, the reaction was filtered through a glass funnel within layer of silica gel (100-200 mesh) and purified by flash column chromatography (Pet/EtOAc, v/v, 10:1-2:1) to give the product **3a** as a white solid (40% yield, 8:1 dr, 95% ee). After that, products *R*-**2a** was obtained by flash chromatography as a white solid (27% yield, 87% ee).

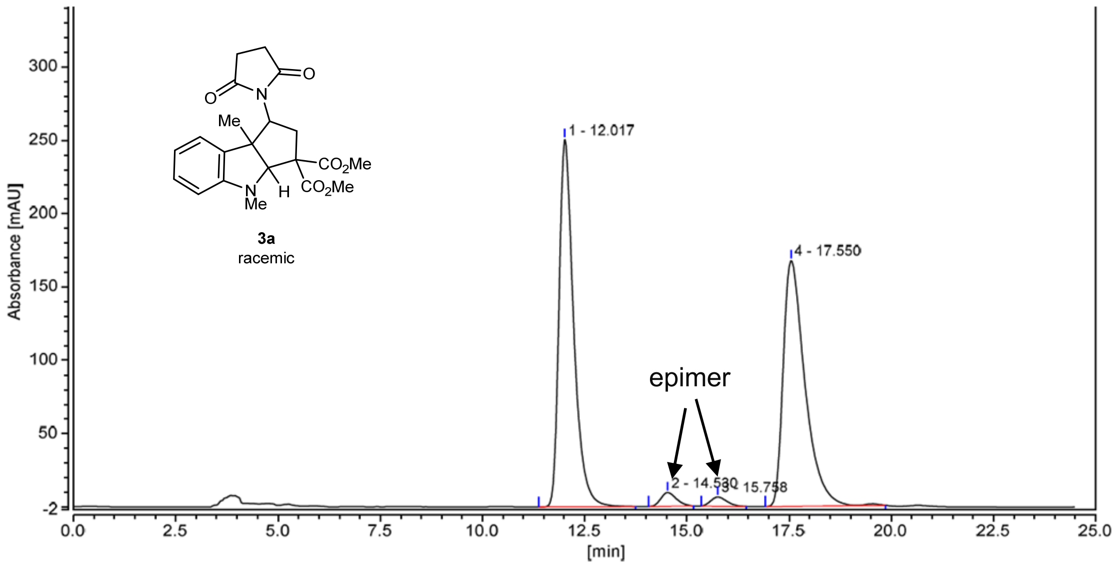


| Peak | Retention Time min | Area mAU*min | Height mAU | Area % | Height % |
|---|---|---|---|---|---|
| 1 | 12.017 | 103.631 | 250.536 | 48.37 | 57.70 |
| 2 | 14.530 | 4.168 | 9.437 | 1.95 | 2.17 |
| 3 | 15.758 | 2.880 | 6.292 | 1.34 | 1.45 |
| 4 | 17.550 | 103.555 | 167.917 | 48.34 | 38.67 |
| **Total:** | | **214.234** | **434.181** | **100.00** | **100.00** |

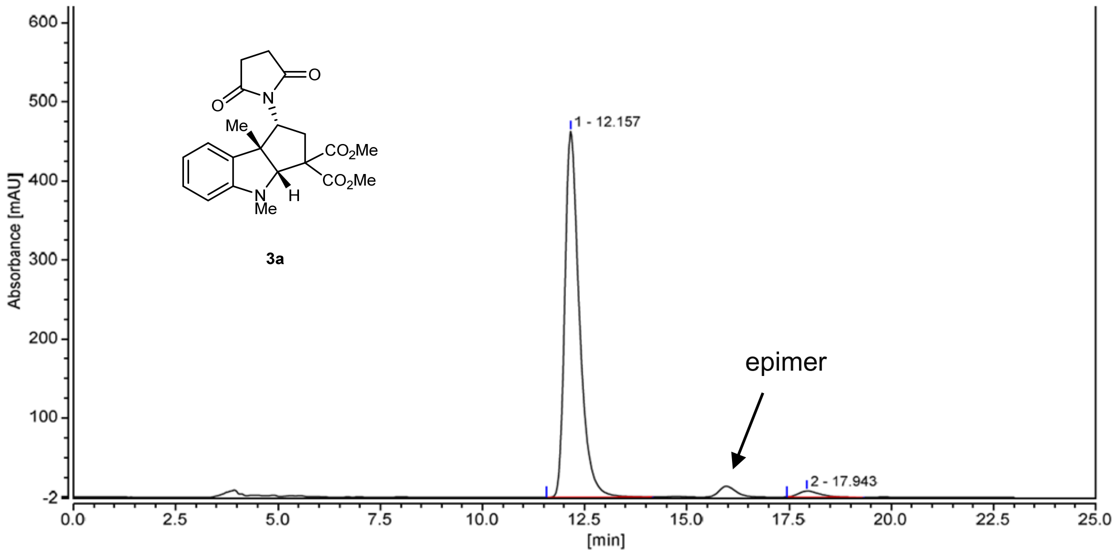


| Peak | Retention Time min | Area mAU*min | Height mAU | Area % | Height % |
|---|---|---|---|---|---|
| 1 | 12.157 | 191.357 | 463.185 | 97.69 | 98.39 |
| 2 | 17.943 | 4.520 | 7.593 | 2.31 | 1.61 |
| **Total:** | | **195.877** | **470.778** | **100.00** | **100.00** |

**Supplementary Fig. 16 HLPC** of product **3a**

**Dimethyl-(*R*)-2-(2,5-dioxopyrrolidin-1-yl)cyclopropane-1,1-dicarboxylate (*R*-2a)**

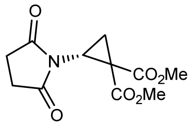


White solid, m.p.: 83.4-86.5 °C; $R_f$ = 0.25 (Pet/EtOAc, 3/2, v/v); $[\alpha]_D^{25}$ = -19.1 (c = 0.5, $CHCl_3$). HPLC CHIRALCEL ODH, *n*-hexane/2-propanol = 60/40, flow rate = 0.8 mL/min, λ = 210 nm, retention time: 13.904 min (minor), 27.336 min (major). $^1H$ NMR (400 MHz, $CDCl_3$) δ 3.79 (s, 3H), 3.69 (s, 3H), 3.46 (dd, *J* = 8.4, 6.4 Hz, 1H), 2.72–2.60 (m, 4H), 2.46 (t, *J* = 6.4 Hz, 1H), 1.94 (dd, *J* = 8.4, 6.4 Hz, 1H); $^{13}C$ NMR (100 MHz, $CDCl_3$) δ 176.9, 168.4, 167.3, 53.2, 53.1, 35.1, 32.7, 28.1, 19.7.

The absolute configuration of product *R*-**2a** was determined by comparison the specific optical rotation and HPLC spectra with the prepared reference sample.

With aminocyclopropane **2a** and benzothiazole **1a** as starting material, the reference sample of *S*-**2a** was prepared by the following route described[7,8].

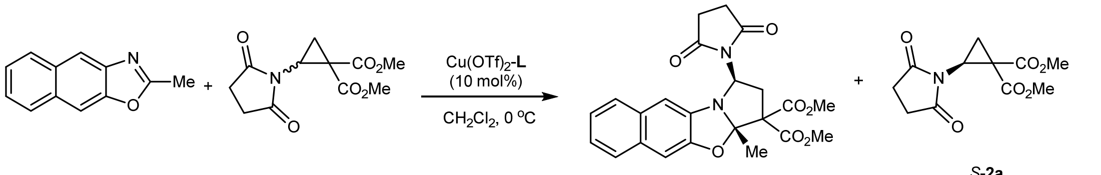


1) Specific optical rotation data

*R*-**2a** in this work, 87% ee. $[\alpha]_D^{25}$ = -19.1 (c = 0.5, $CHCl_3$);

*S*-**2a** in the prepared reference sample, 98% ee. $[\alpha]_D^{20}$ = +8.2 (c = 0.61, $CHCl_3$).

By comparison the specific optical rotation data, the absolute configuration of **2a** in this work was determined to be *R* configuration.

2) HPLC spectra

*R*-**2a** in this work, 87% ee. HPLC CHIRALCEL ODH, *n*-hexane/2-propanol = 60/40, flow rate = 0.8 mL/min, λ = 210 nm, retention time: 13.904 min (minor), 27.336 min (major).

*S*-**2a** in the prepared reference sample, 98% ee. HPLC CHIRALCEL ODH, n-hexane/2-propanol = 60/40, flow rate = 0.8 mL/min, λ = 210 nm, retention time: 12.868 min (major), 29.605 min (minor).

By comparison the HPLC spectra, the absolute configuration of **2a** in this work was confirmed to be *R* configuration.

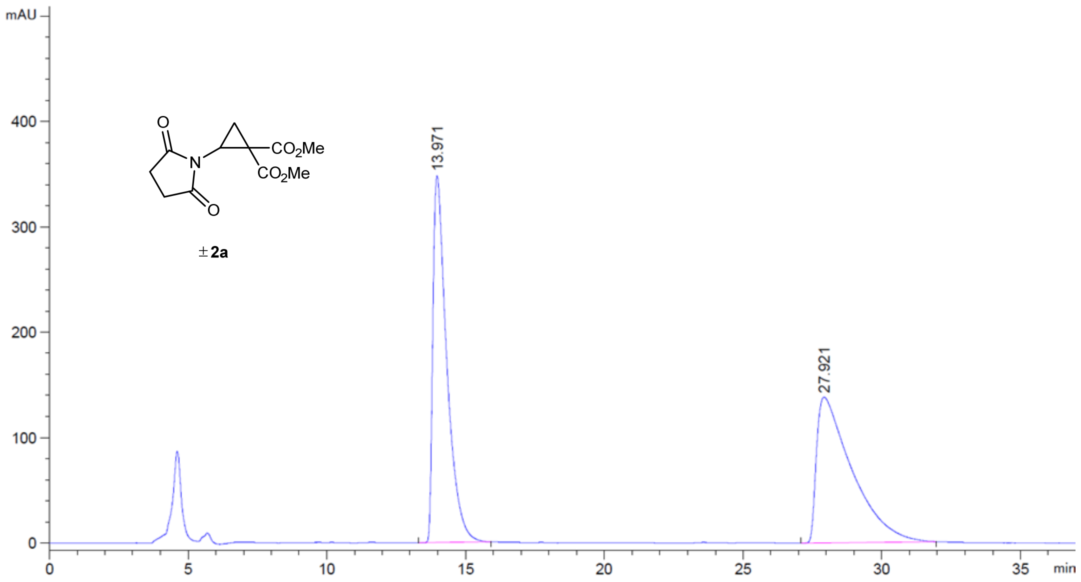


| Peak # | RetTime [min] | Type | Width [min] | Area [mAU*s] | Height [mAU] | Area % |
|---|---|---|---|---|---|---|
| 1 | 13.971 | BB | 0.5173 | 1.21357e4 | 348.18277 | 49.6699 |
| 2 | 27.921 | BB | 1.1986 | 1.22970e4 | 138.34830 | 50.3301 |

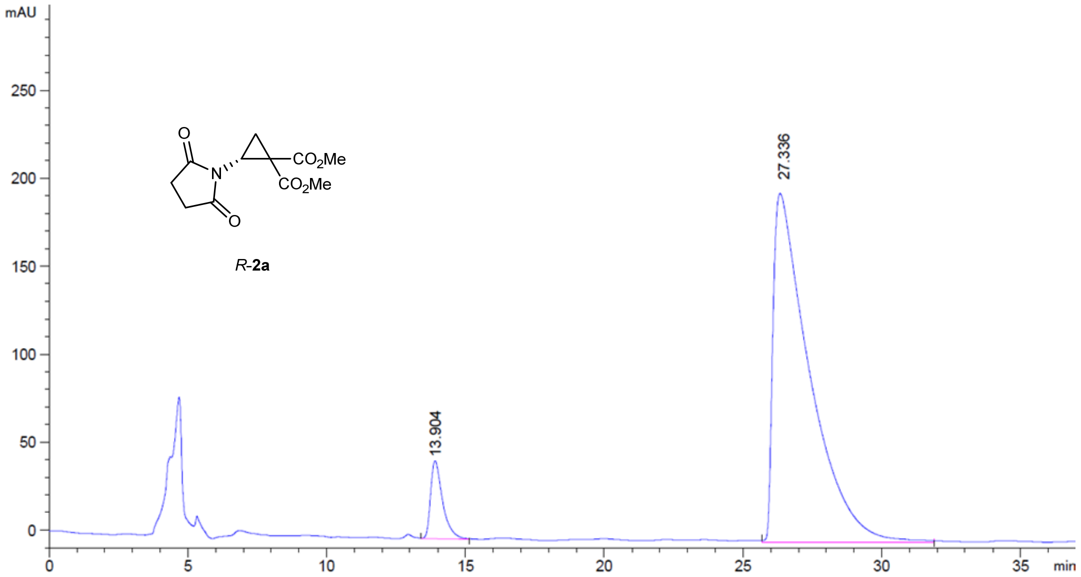


| Peak # | RetTime [min] | Type | Width [min] | Area [mAU*s] | Height [mAU] | Area % |
|---|---|---|---|---|---|---|
| 1 | 13.904 | VB | 0.4489 | 1328.57019 | 44.31153 | 6.6961 |
| 2 | 27.336 | MM | 1.5477 | 1.85124e4 | 199.35600 | 93.3039 |

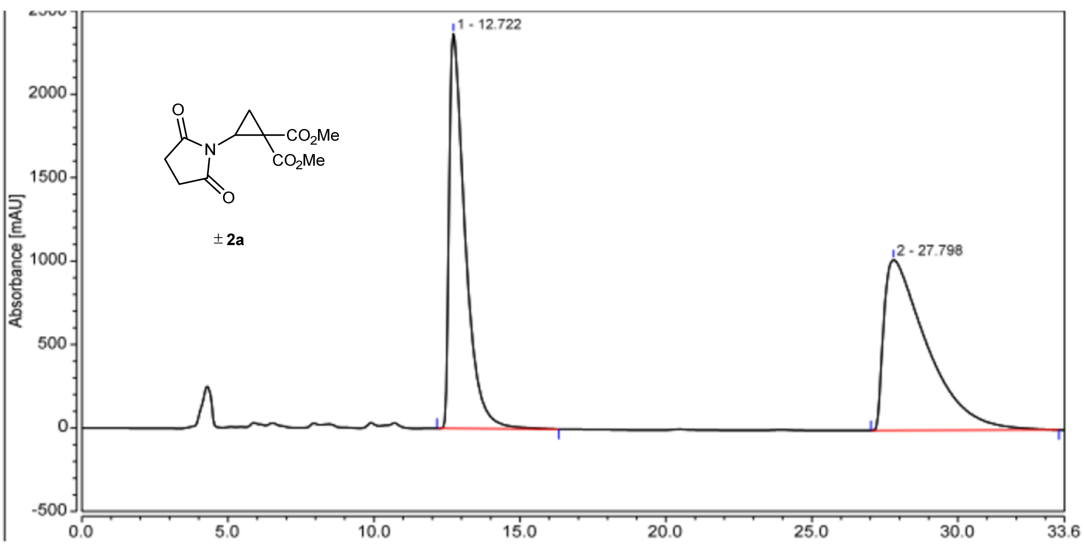


| Peak | Retention Time<br>min | Area<br>mAU*min | Height<br>mAU | Area<br>% | Height<br>% |
|---|---|---|---|---|---|
| 1 | 12.722 | 1505.498 | 2366.639 | 46.05 | 69.83 |
| 2 | 27.798 | 1763.889 | 1022.564 | 53.95 | 30.17 |
| **Total:** | | **3269.387** | **3389.203** | **100.00** | **100.00** |

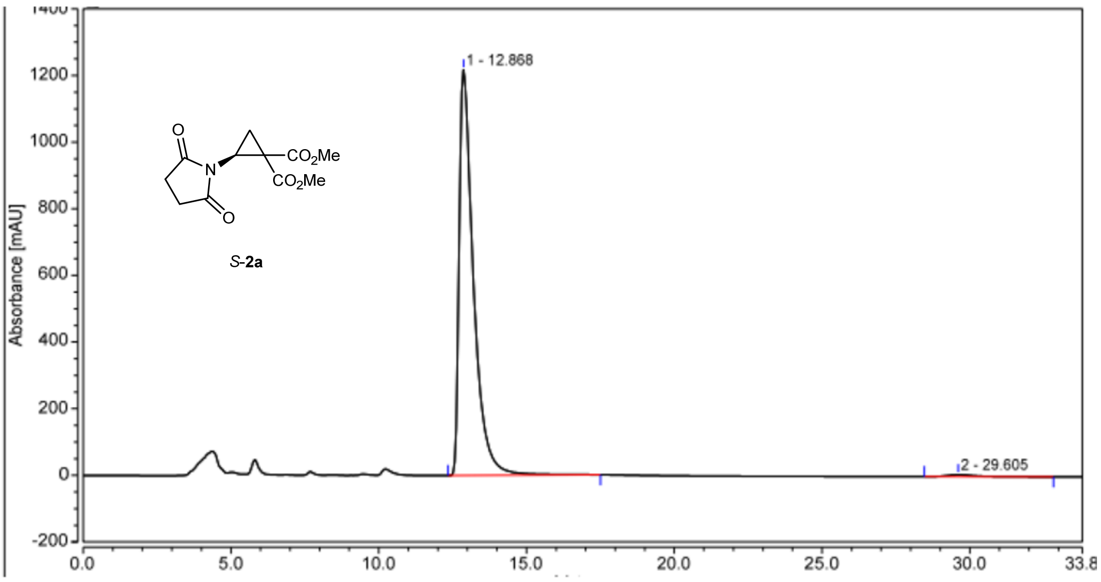


| Peak | Retention Time<br>min | Area<br>mAU*min | Height<br>mAU | Area<br>% | Height<br>% |
|---|---|---|---|---|---|
| 1 | 12.868 | 685.273 | 1216.722 | 99.11 | 99.53 |
| 2 | 29.605 | 6.171 | 5.704 | 0.89 | 0.47 |
| **Total:** | | **691.444** | **1222.426** | **100.00** | **100.00** |

**Supplementary Fig. 17 HLPC** spectra of product *R*-**2a** in this work and reference sample *S*-**2a**

## Nonlinear effect experiment

Nonlinear effect experiment between the ee value of ligand **L4** and product **3a**

a) For the preparation of the ligand **L4** solution: in a 5.0 mL of volumetric flask, **L4** (160.0 mg, 0.24 mmol) was added, then DCM (5.0 mL) was added to make the total volume up to 5.0 mL

b) For the preparation of the ligand *ent*-**L4** solution: in a 5.0 mL of volumetric flask, **L4** (160.0 mg, 0.24 mmol) was added, then DCM (5.0 mL) was added to make the total volume up to 5.0 mL

For 20% ee, **L4** : 150.0 μL **L4** was mixed with 100.0 μL *ent*-**L4**;

For 40% ee, **L4** : 175.0 μL **L4** was mixed with 75.0 μL *ent*-**L4**;

For 60% ee, **L4** : 200.0 μL **L4** was mixed with 50.0 μL *ent*-**L4**;

For 80% ee, **L4** : 225.0 μL **L4** was mixed with 25.0 μL *ent*-**L4**;

Take $Ni(OTf)_2$ (3.6 mg) and ligand **L4** (20-80% ee, 250.0 μL), the aminocyclopropanes **2a** (126.5 mg, 0.5 mmol) were added to the reaction tube containing the metal ligand. Dry $CH_2Cl_2$ (4.0 mL) was added to the mixture as a solvent. The mixture was placed at 25 ºC and stirred at room temperature in nitrogen atmosphere for 30 min. Then the 1,3-dimethylindole **1a** (29.0 mg, 0.2 mmol) was added and reacted at 25 ºC for 24 h. After the reaction, thin layer chromatography (TLC) was used to detect the reaction products, and Pet/EtOAc (10/1-2/1, v/v) was used as the eluent, and column chromatography was performed directly on silica gel to obtain the desired product **3a**.

**Supplementary Table 13.** Nonlinear effect experiment

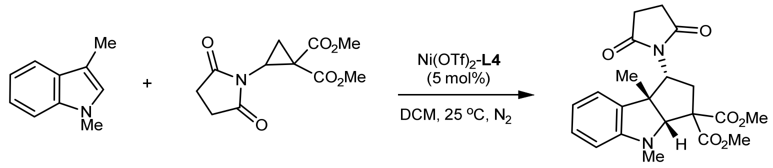


**1a** ± **2a** **3a**

| entry | ee of ligand **L4** (%) | ee of product **3a** (%) |
|---|---|---|
| 1 | 0 | 0 |
| 2 | 20 | 19 |
| 3 | 40 | 39 |
| 4 | 60 | 60 |
| 5 | 80 | 76 |
| 6 | 99 | 98 |

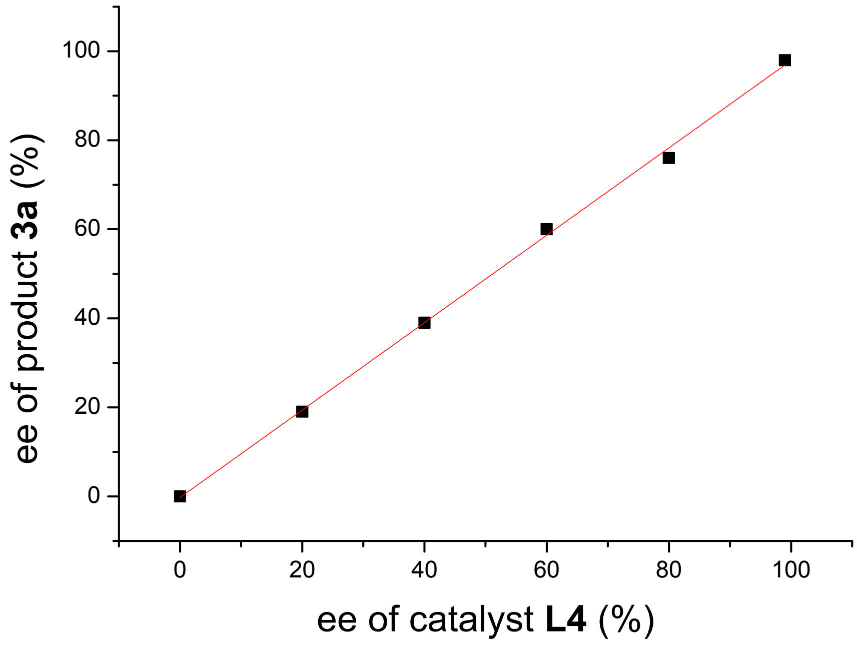


**Supplementary Fig. 18** The relationship between the ee value of ligand **L4** and product **3a**

HPLC CHIRALPAK IA, n-hexane/2-propanol = 80/20, flow rate 0.8 mL/min, λ = 254 nm, retention time: 15.117 min (minor), 22.958 min (major).

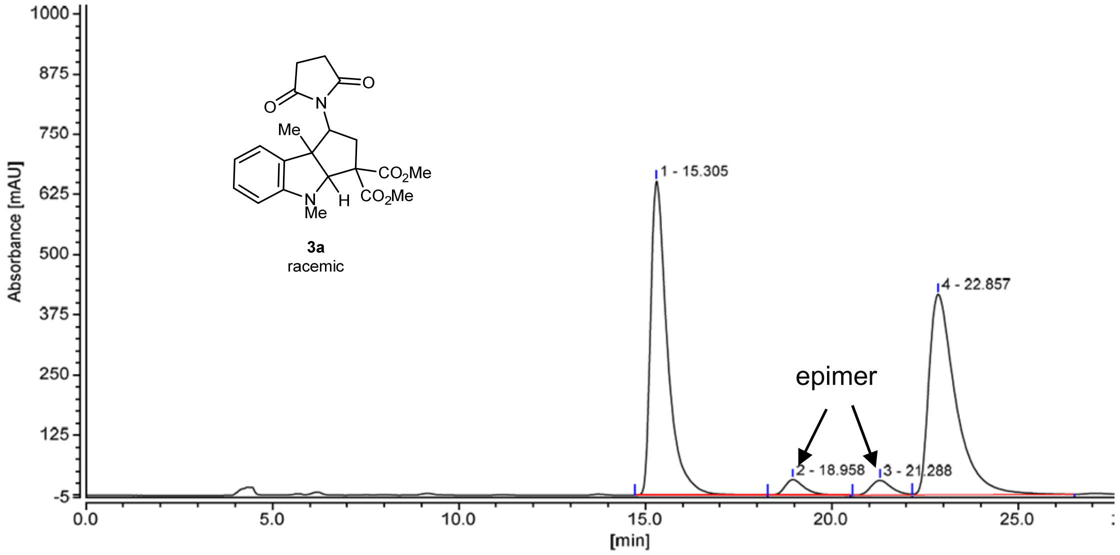


| Peak | Retention Time min | Area mAU*min | Height mAU | Area % | Height % |
|---|---|---|---|---|---|
| 1 | 15.305 | 341.327 | 652.988 | 46.91 | 57.63 |
| 2 | 18.958 | 19.633 | 32.148 | 2.70 | 2.84 |
| 3 | 21.288 | 19.320 | 30.412 | 2.65 | 2.68 |
| 4 | 22.857 | 347.417 | 417.486 | 47.74 | 36.85 |
| **Total:** | | **727.696** | **1133.033** | **100.00** | **100.00** |

In the presence of ligand **L4** with 20% ee

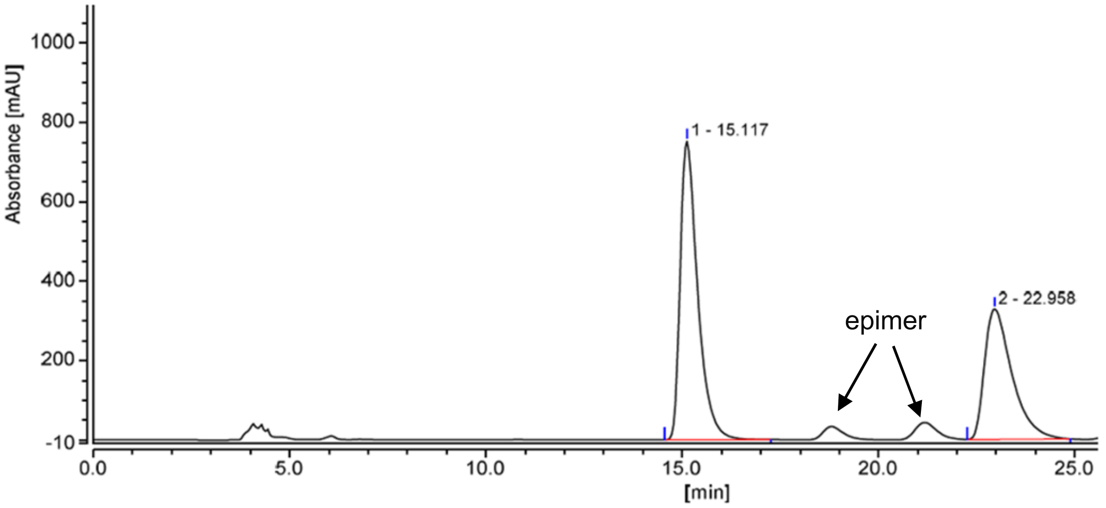


| Peak | Retention Time min | Area mAU*min | Height mAU | Area % | Height % |
|---|---|---|---|---|---|
| 1 | 15.117 | 388.908 | 752.865 | 59.40 | 69.64 |
| 2 | 22.958 | 265.771 | 328.185 | 40.60 | 30.36 |
| **Total:** | | **654.680** | **1081.050** | **100.00** | **100.00** |

In the presence of ligand **L4** with 40% ee

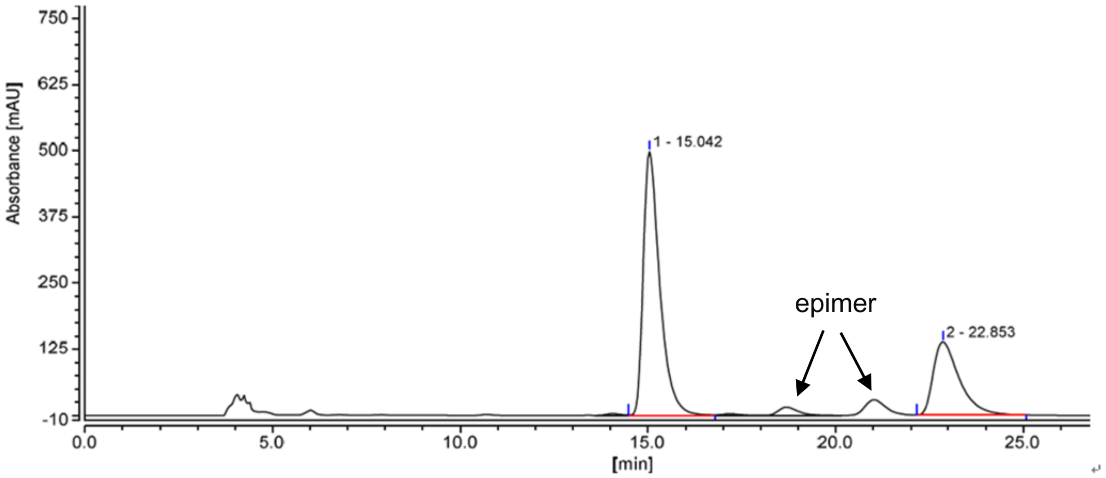


| Peak | Retention Time min | Area mAU*min | Height mAU | Area % | Height % |
|---|---|---|---|---|---|
| 1 | 15.042 | 255.220 | 498.854 | 69.57 | 78.29 |
| 2 | 22.853 | 111.655 | 138.313 | 30.43 | 21.71 |
| **Total:** | | **366.875** | **637.167** | **100.00** | **100.00** |

In the presence of ligand **L4** with 60% ee

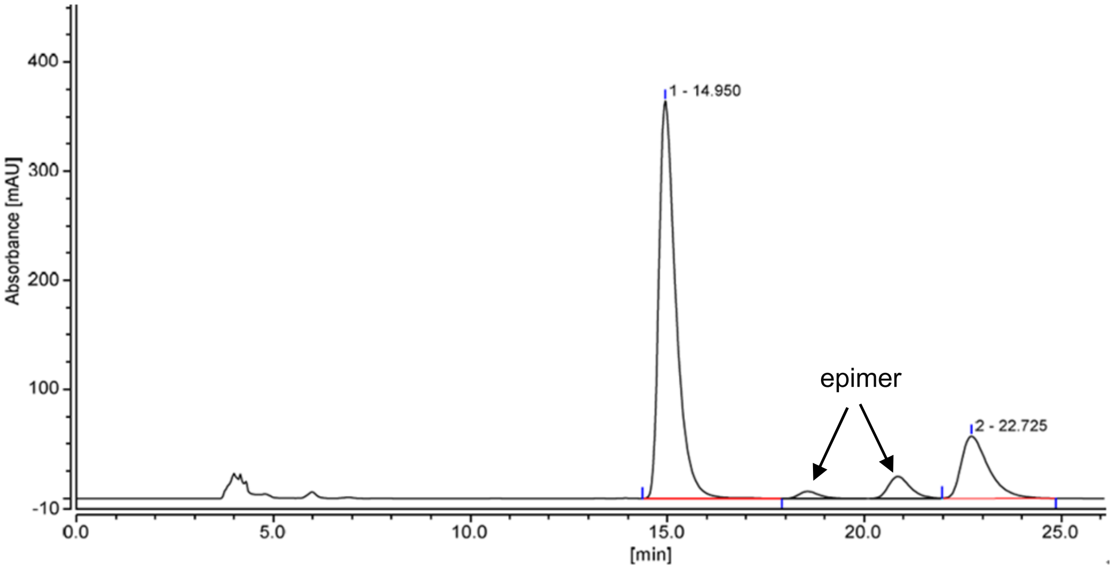


| Peak | Retention Time min | Area mAU*min | Height mAU | Area % | Height % |
|---|---|---|---|---|---|
| 1 | 14.950 | 182.997 | 364.389 | 80.10 | 86.45 |
| 2 | 22.725 | 45.468 | 57.097 | 19.90 | 13.55 |
| **Total:** | | **228.465** | **421.485** | **100.00** | **100.00** |

In the presence of ligand **L4** with 80% ee

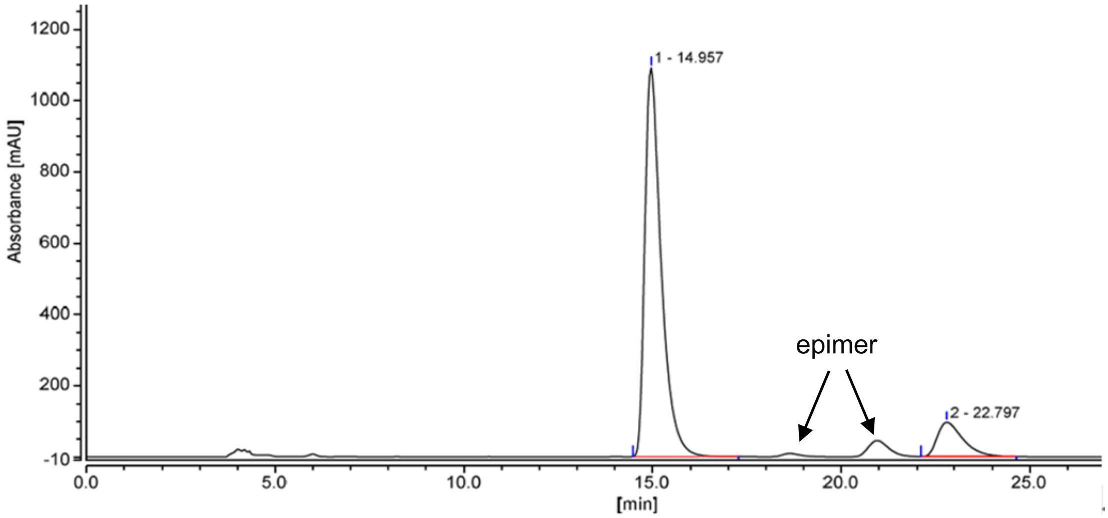


| Peak | Retention Time min | Area mAU*min | Height mAU | Area % | Height % |
|---|---|---|---|---|---|
| 1 | 14.957 | 549.767 | 1091.858 | 87.79 | 91.88 |
| 2 | 22.797 | 76.486 | 96.449 | 12.21 | 8.12 |
| **Total:** | | **626.253** | **1188.307** | **100.00** | **100.00** |

In the presence of ligand **L4** with 99% ee

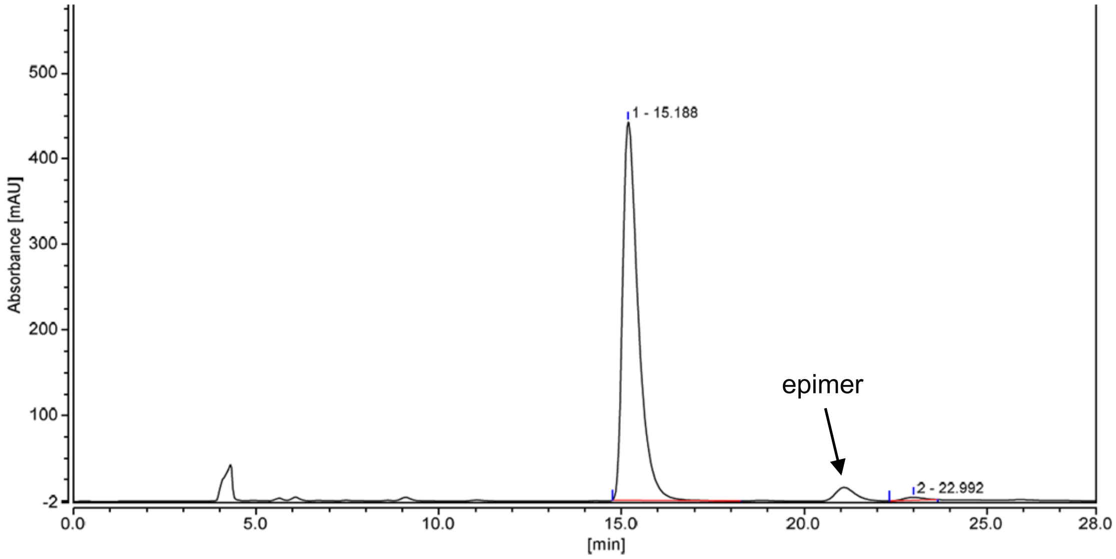


| Peak | Retention Time min | Area mAU*min | Height mAU | Area % | Height % |
|---|---|---|---|---|---|
| 1 | 15.188 | 219.510 | 441.998 | 99.03 | 99.22 |
| 2 | 22.992 | 2.157 | 3.489 | 0.97 | 0.78 |
| **Total:** | | **221.667** | **445.487** | **100.00** | **100.00** |

**Supplementary Fig. 19 HLPC** of **3a** with different ee value of ligand **L4**

# Transformations of products 3a

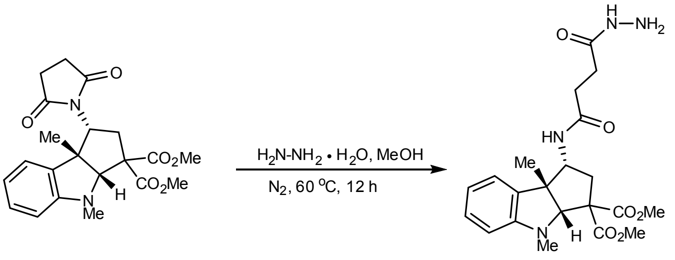


**3a**
0.1 mmol, 98% ee

**6a**
93% yield, 99% ee

In a schlenk tube, **3a** (40.0 mg, 98% ee, 0.1 mmol), hydrazine hydrate (30.0 μL, 0.6 mmol, 6.0 equiv) and MeOH (3.0 mL) were added. The mixture was stirred at 60 °C for 12 h under $N_2$ atmosphere. Finally, the reaction mixture was concentrated in vacuo and was purified by flash column chromatography (pure EtOAc) to give product **6a** as a white solid (40.2 mg, 99% ee, 93% yield).

HPLC CHIRALPAK IA, n-hexane/2-propanol = 80/20, flow rate 0.8 mL/min, λ = 254 nm, retention time: 11.193 min (minor),17.890 min (major).

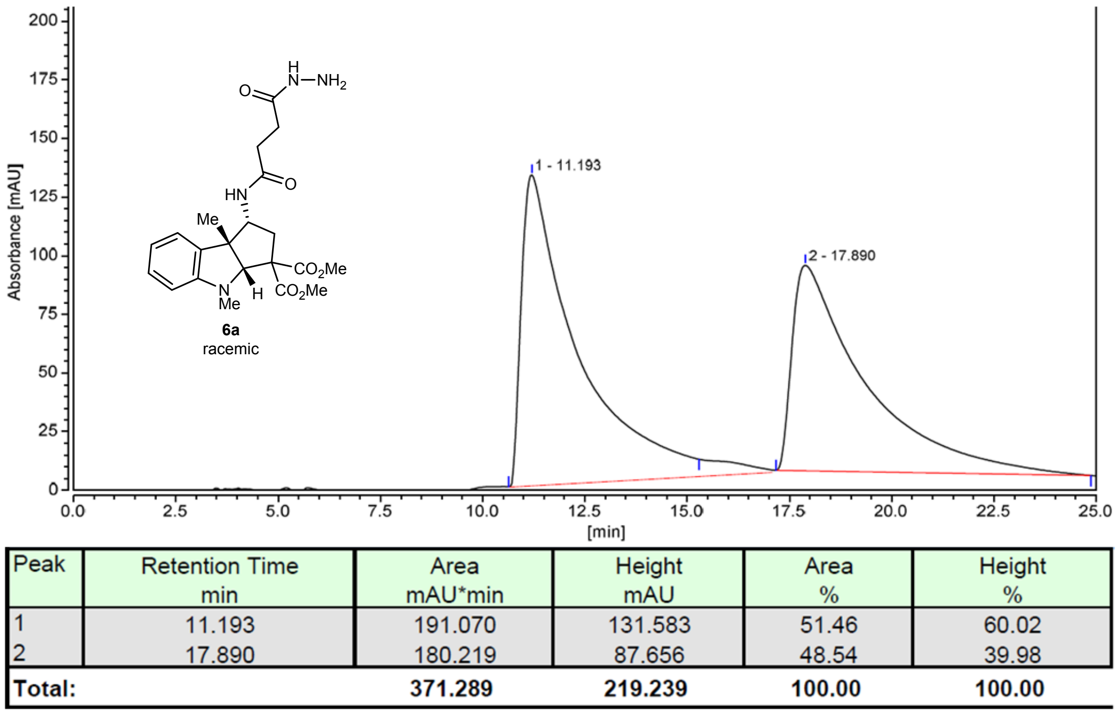


| Peak | Retention Time min | Area mAU*min | Height mAU | Area % | Height % |
|---|---|---|---|---|---|
| 1 | 11.193 | 191.070 | 131.583 | 51.46 | 60.02 |
| 2 | 17.890 | 180.219 | 87.656 | 48.54 | 39.98 |
| **Total:** | | **371.289** | **219.239** | **100.00** | **100.00** |

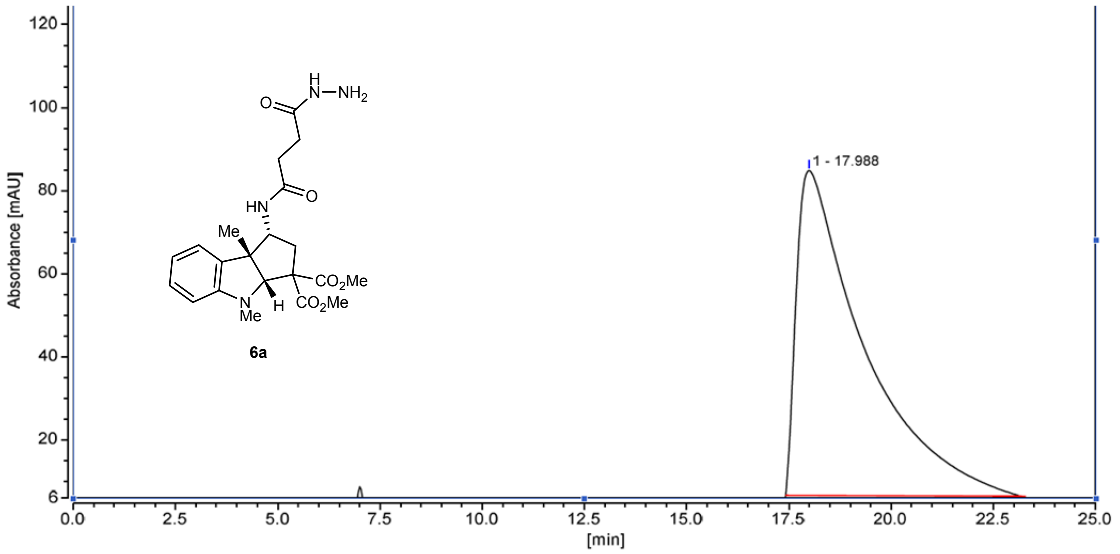


| Peak | Retention Time min | Area mAU*min | Height mAU | Area % | Height % |
|---|---|---|---|---|---|
| 1 | 17.988 | 172.584 | 81.942 | 100.00 | 100.00 |
| **Total:** | | **172.584** | **81.942** | **100.00** | **100.00** |

**Supplementary Fig. 20** HLPC of **6a**

# NMR Spectra of all new compounds

Supplementary Fig. 21 $^{1}H$ NMR(400 MHz, $CDCl_3$)

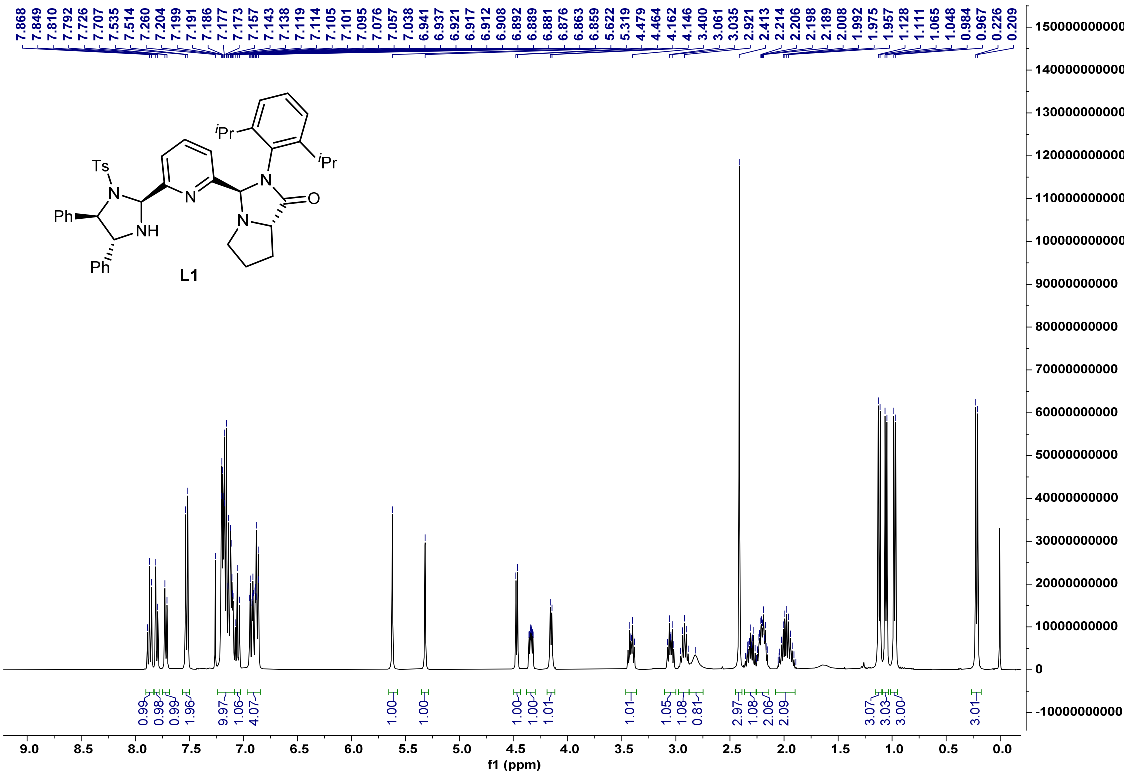


Supplementary Fig. 22 $^{13}C$ NMR(100 MHz, $CDCl_3$)

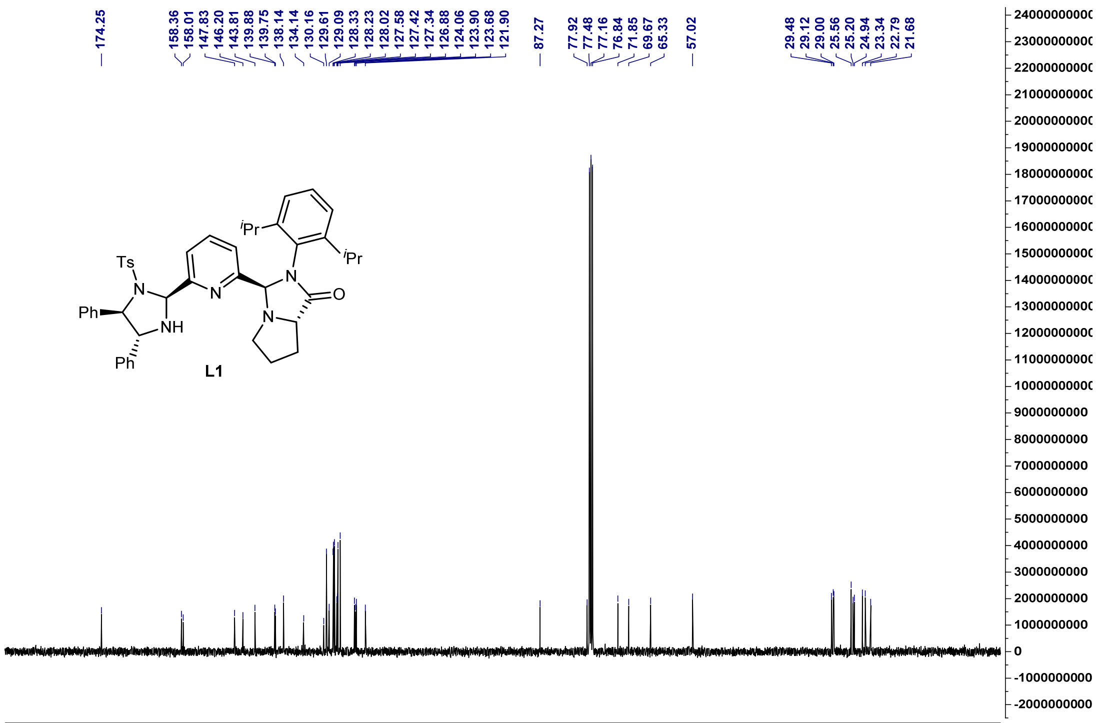

Supplementary Fig. 23 $^1H$ NMR(600 MHz, $CDCl_3$)

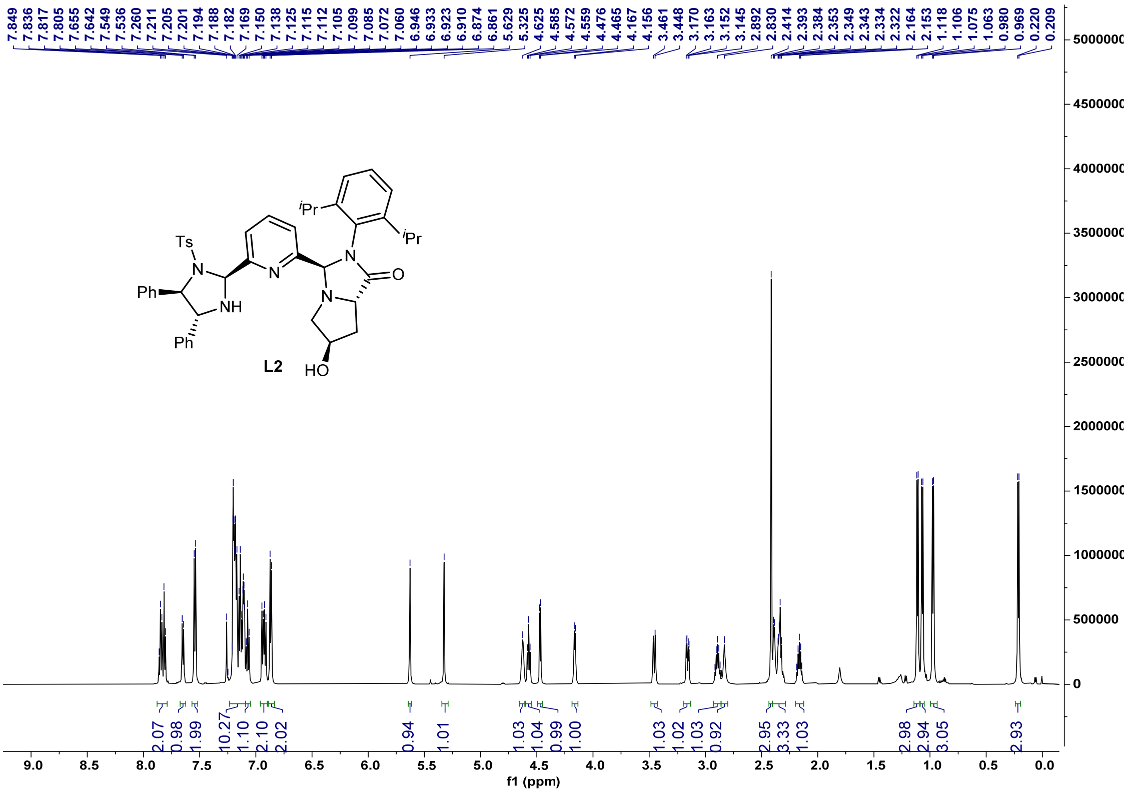

Supplementary Fig. 24 $^{13}C$ NMR(150 MHz, $CDCl_3$)

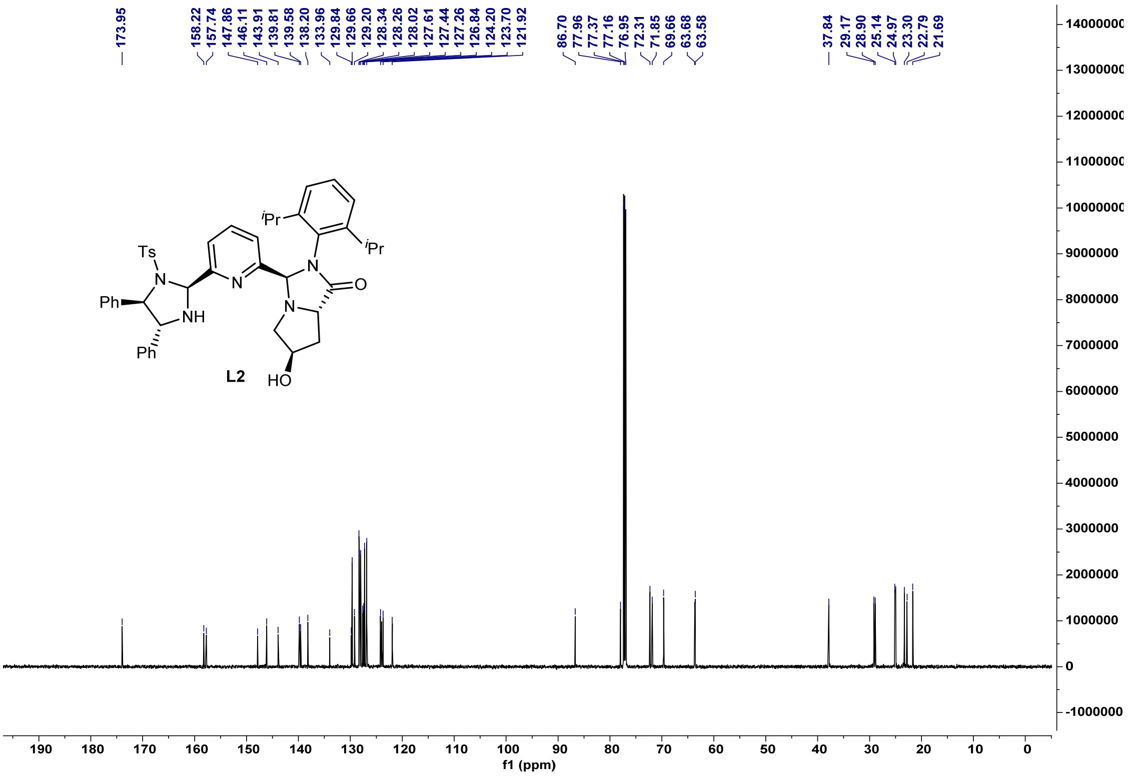

Supplementary Fig. 25 $^{1}H$ NMR(400 MHz, $CDCl_3$)

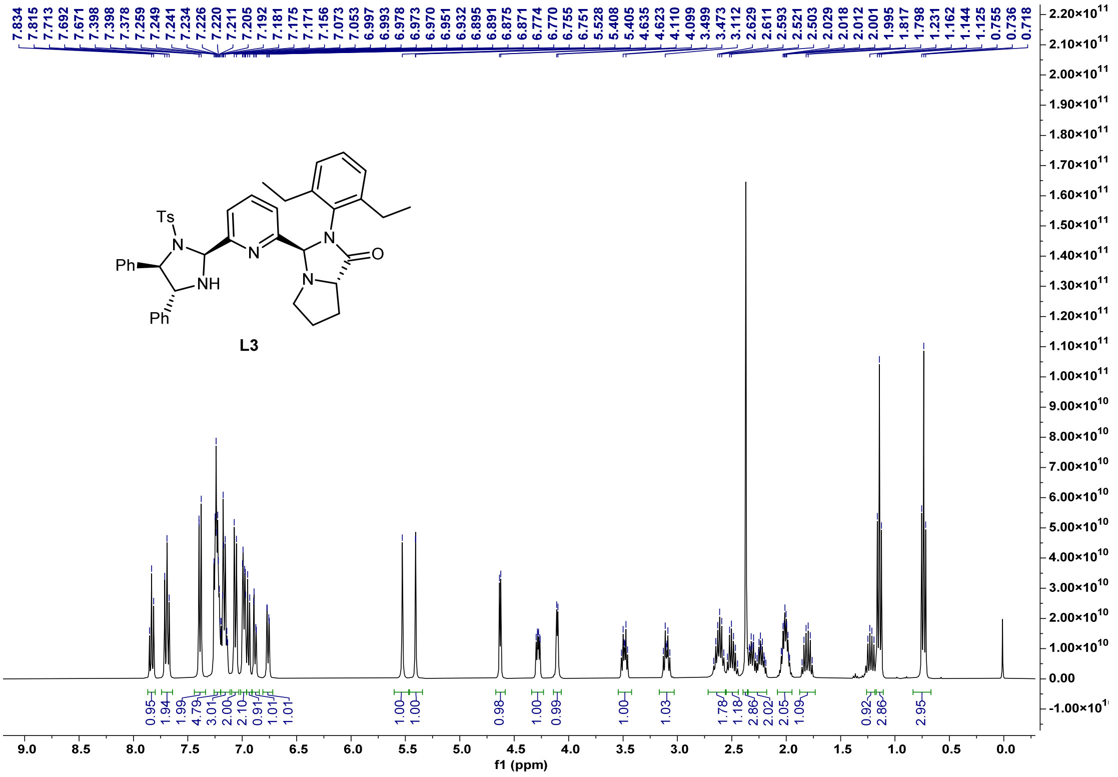


Supplementary Fig. 26 $^{13}C$ NMR(100 MHz, $CDCl_3$)

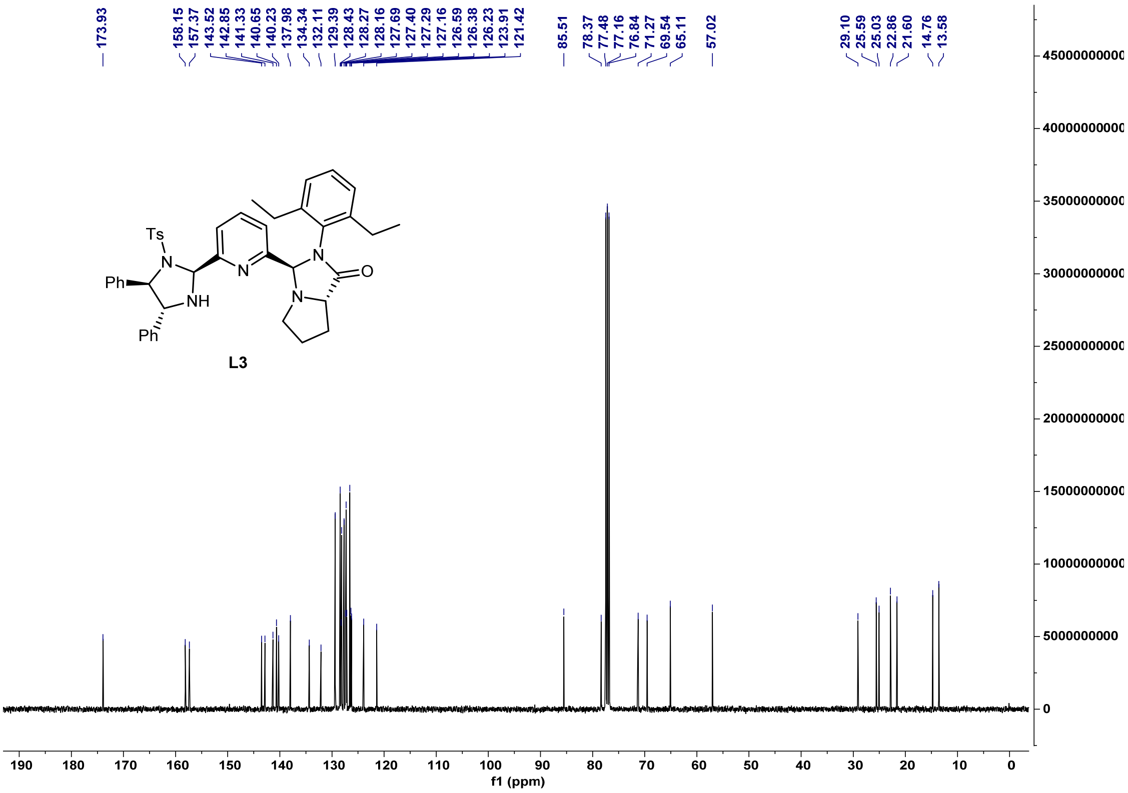

Supplementary Fig. 27 $^{1}$H NMR(600 MHz, $CDCl_3$)

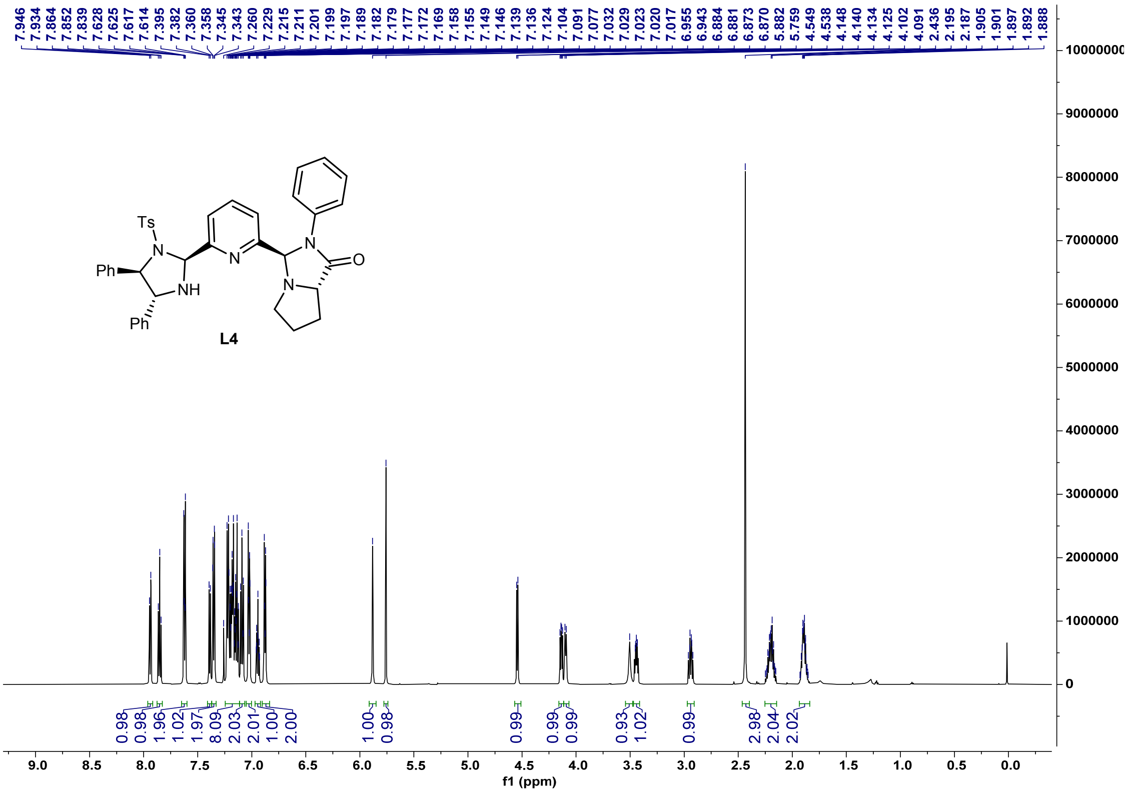

Supplementary Fig. 28 $^{13}$C NMR(150 MHz, $CDCl_3$)

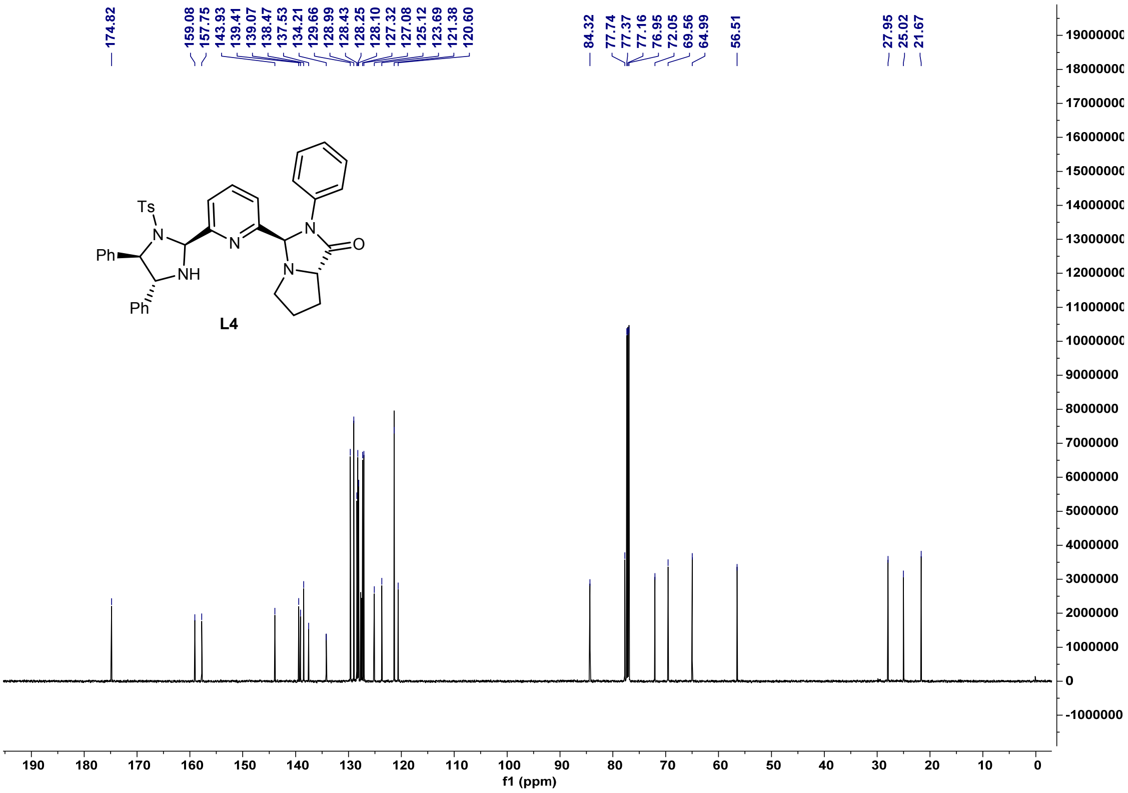

Supplementary Fig. 29 $^{1}$H NMR(400 MHz, $CDCl_3$)

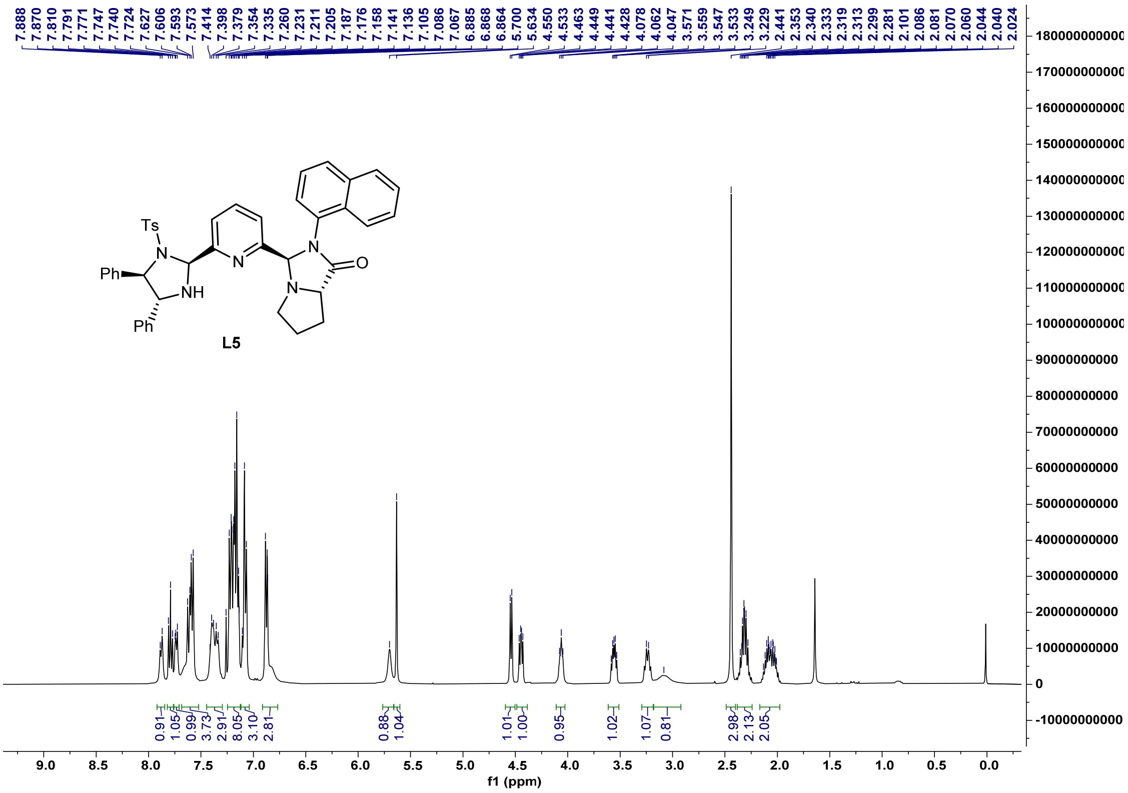


Supplementary Fig. 30 $^{13}$C NMR(100 MHz, $CDCl_3$)

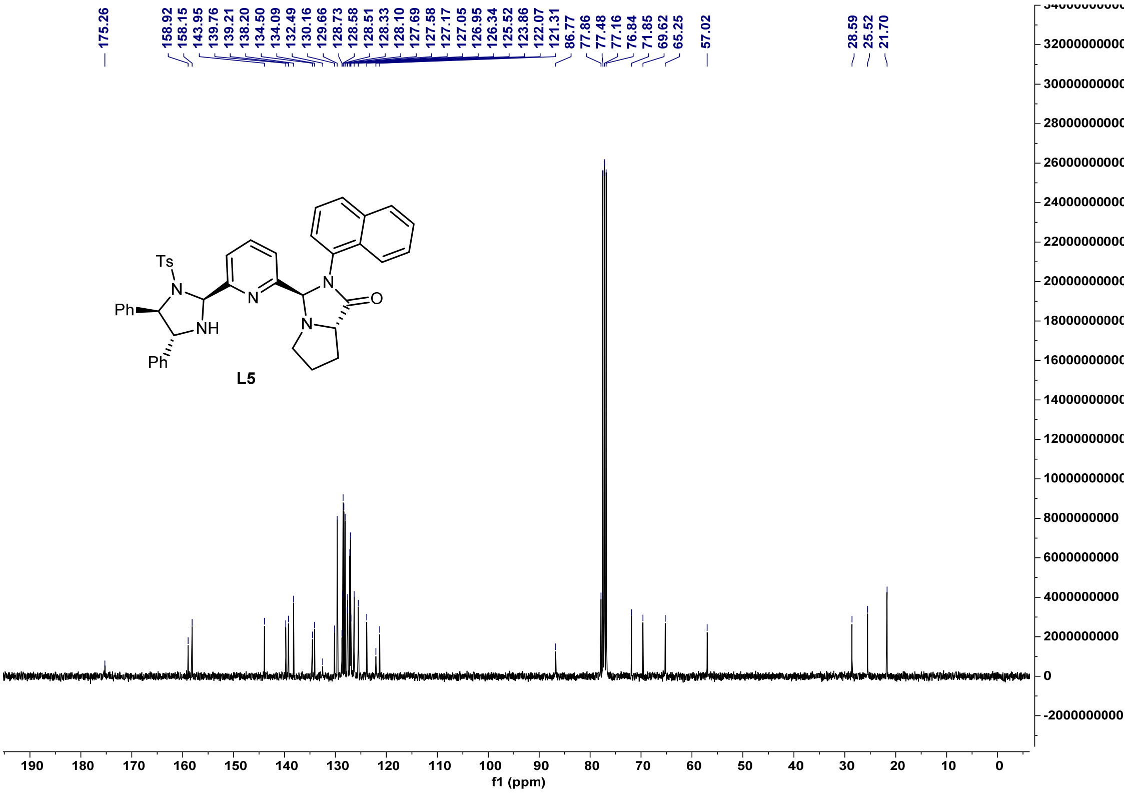

Supplementary Fig. 31 $^{1}$H NMR(400 MHz, $CDCl_3$)

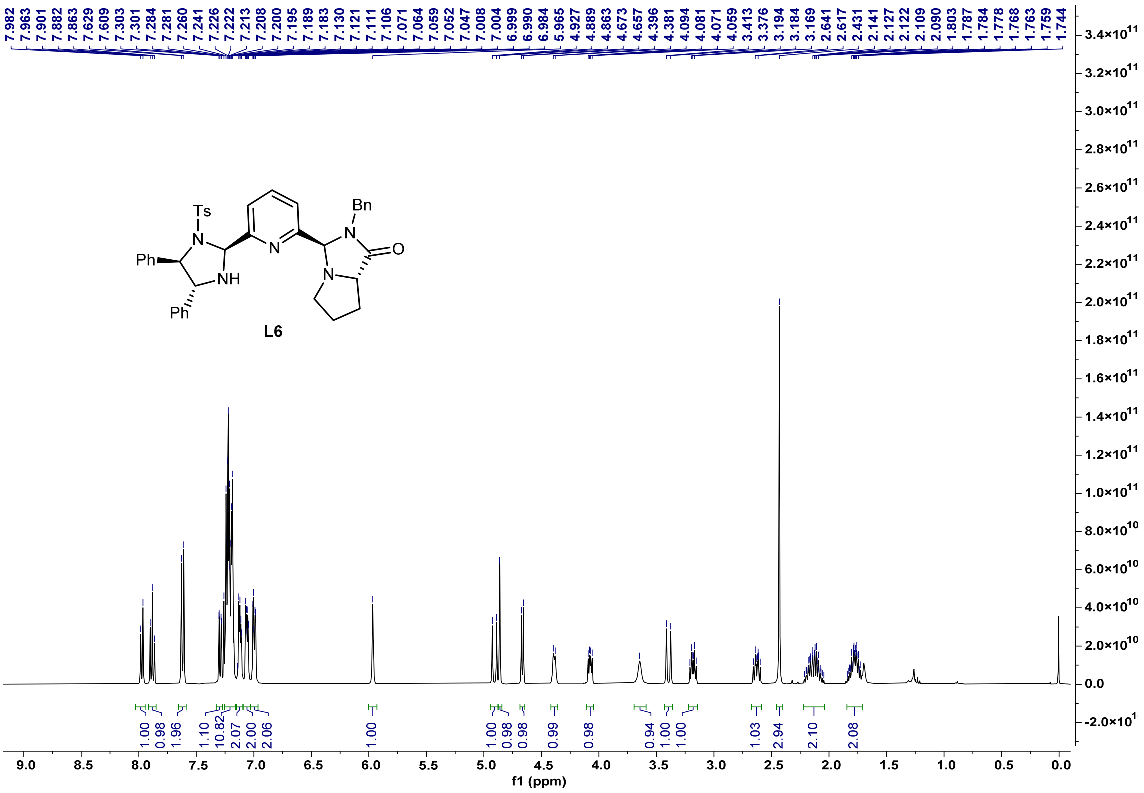


Supplementary Fig. 32 $^{13}$C NMR(100 MHz, $CDCl_3$)

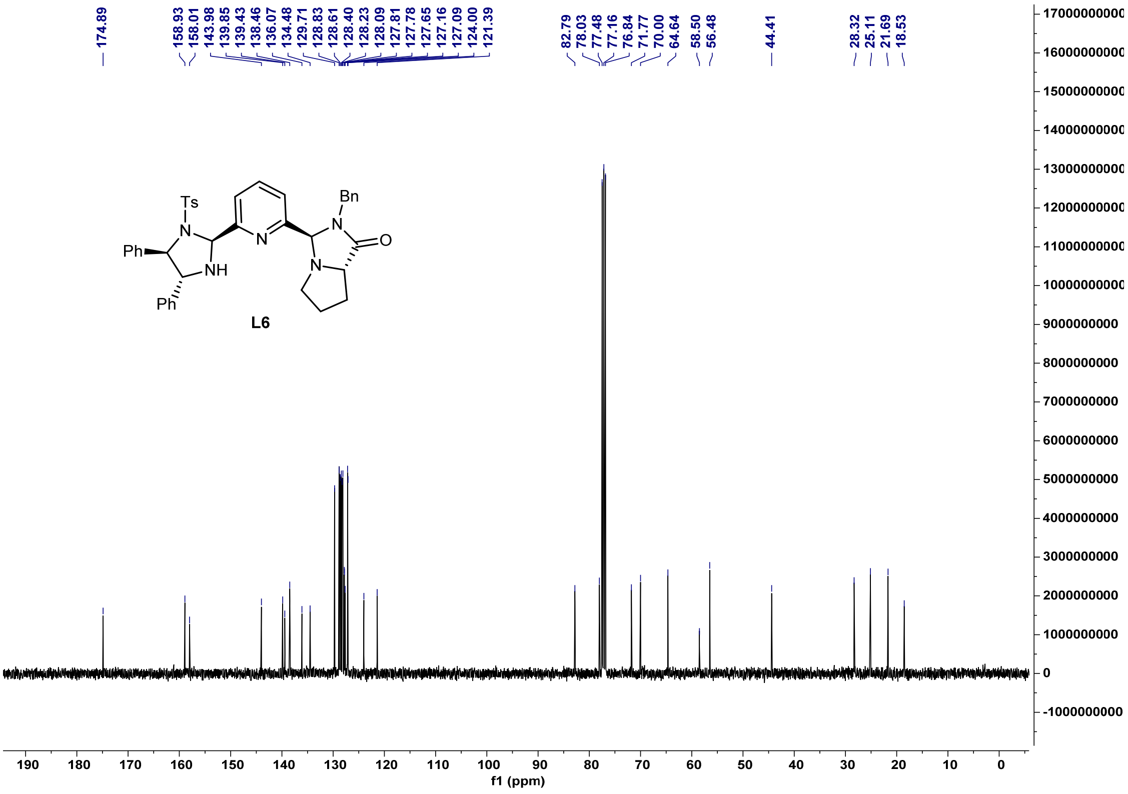

Supplementary Fig. 33 $^{1}H$ NMR(600 MHz, $CDCl_3$)

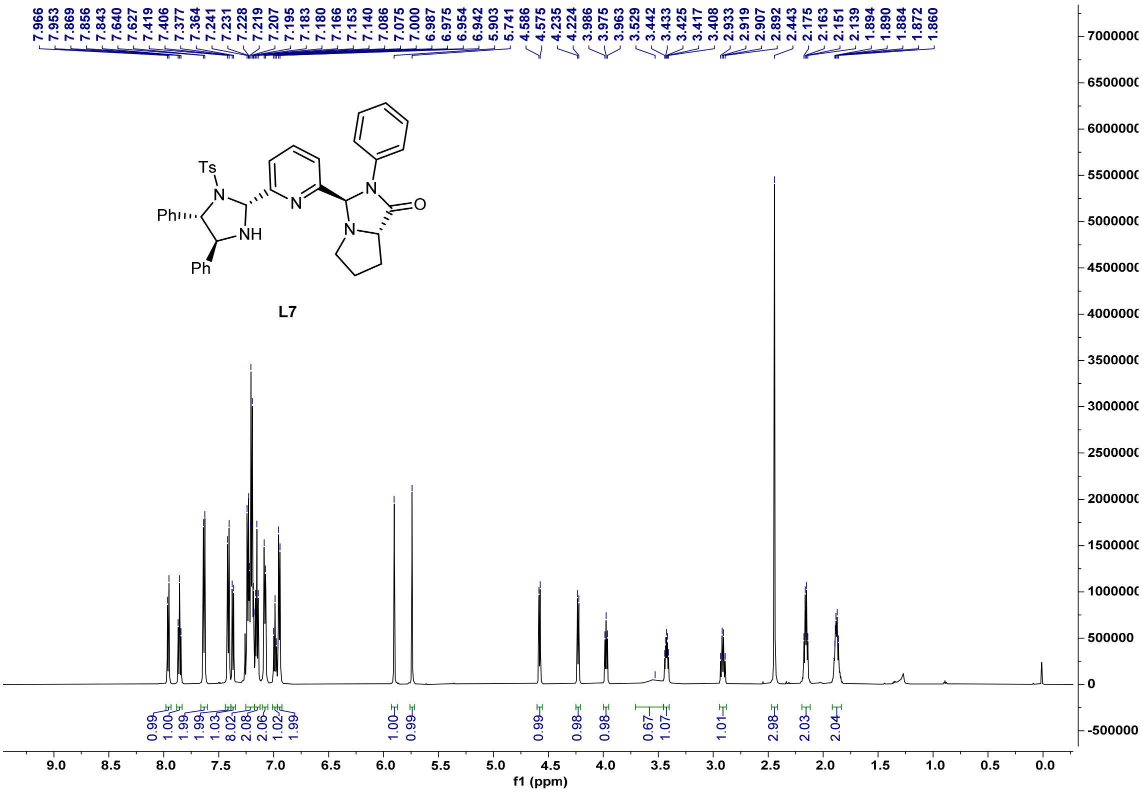


Supplementary Fig. 34 $^{13}C$ NMR(150 MHz, $CDCl_3$)

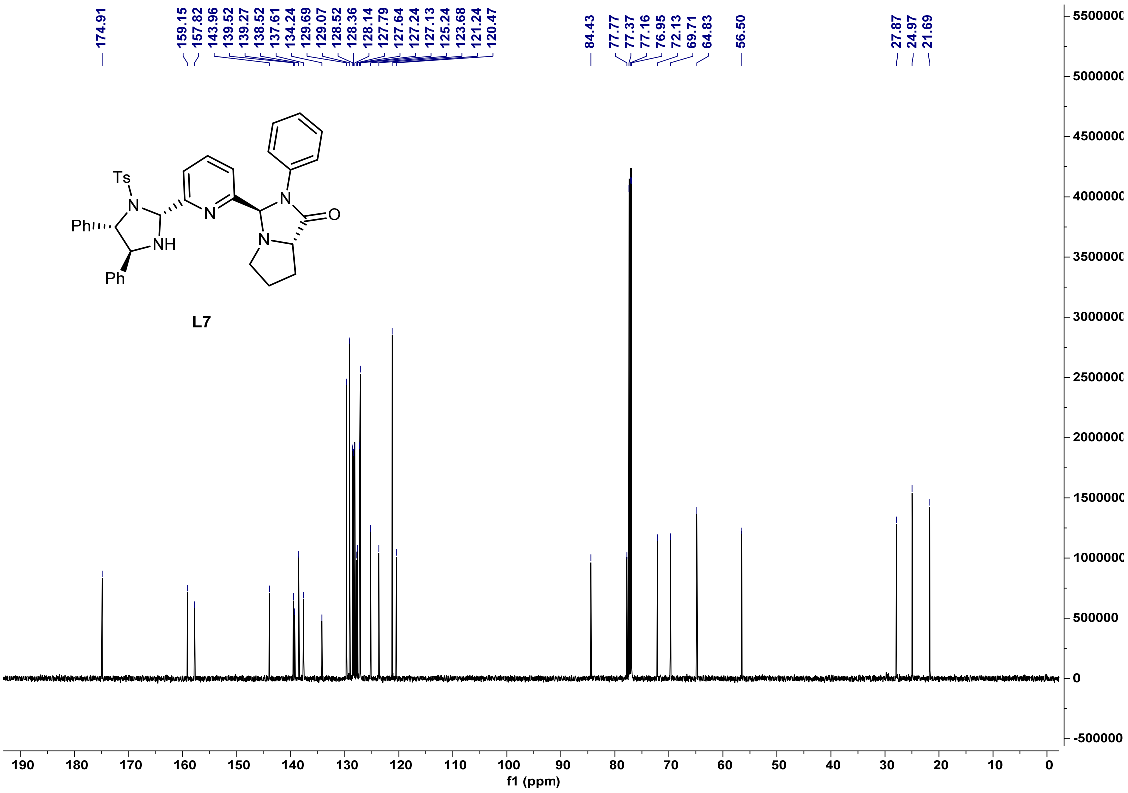

Supplementary Fig. 35 $^{1}$H NMR(400 MHz, $CDCl_3$)

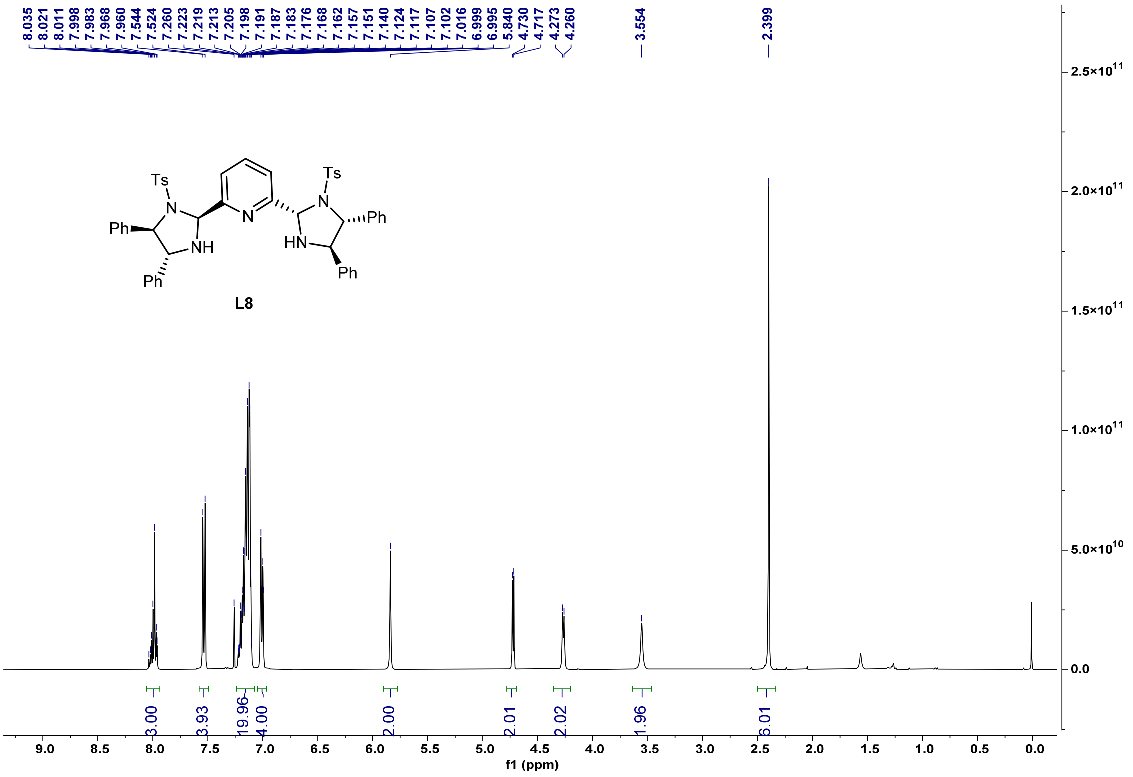

Supplementary Fig. 36 $^{13}$C NMR(100 MHz, $CDCl_3$)

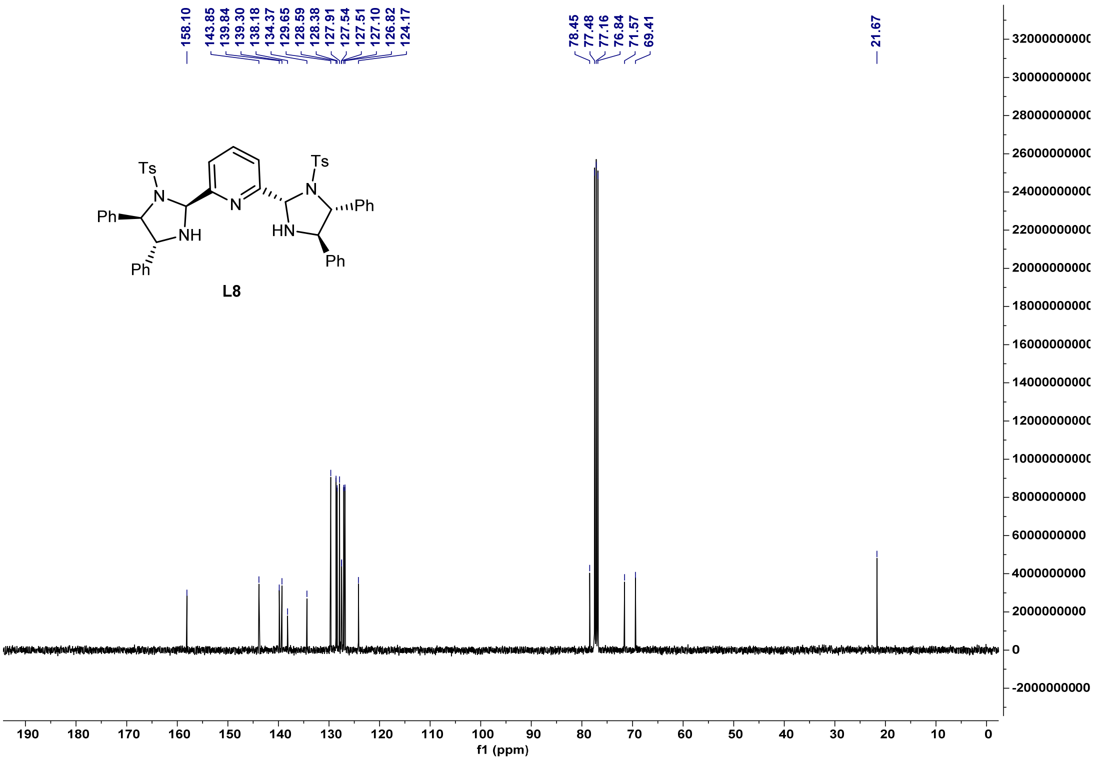

Supplementary Fig. 37 $^{1}H$ NMR(600 MHz, $CDCl_3$)

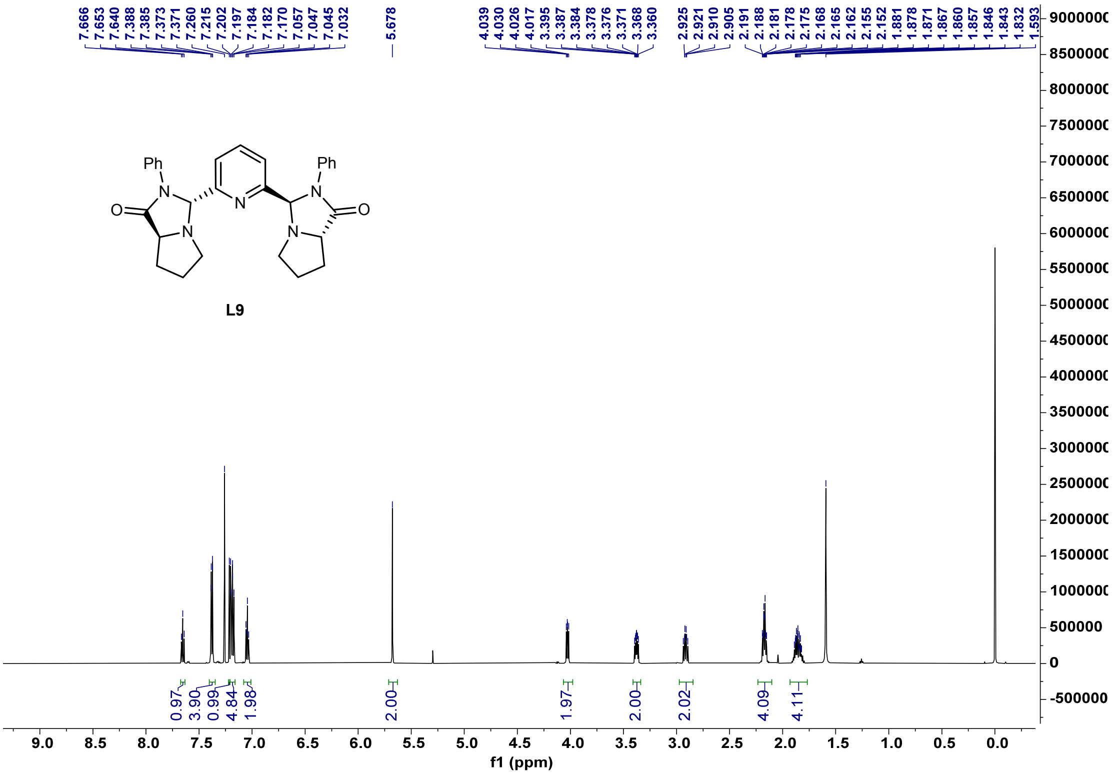


Supplementary Fig. 38 $^{13}C$ NMR(100 MHz, $CDCl_3$)

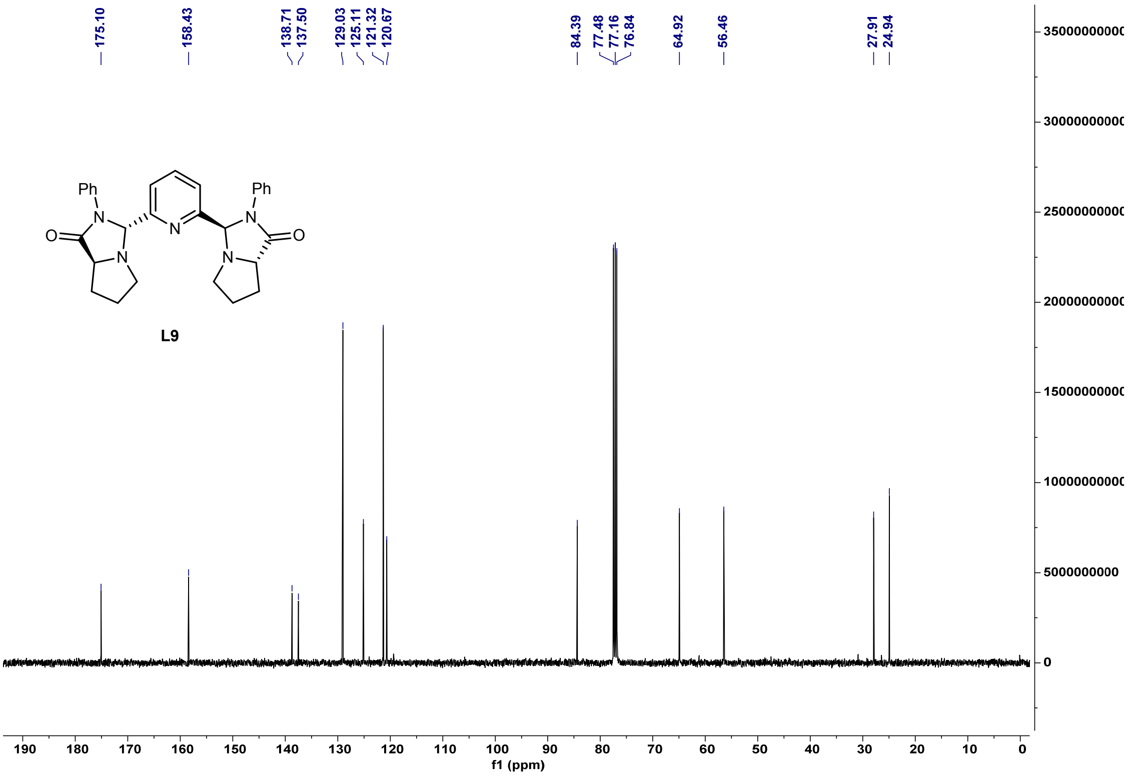

Supplementary Fig. 39 $^{1}$H NMR(400 MHz, $CDCl_3$)

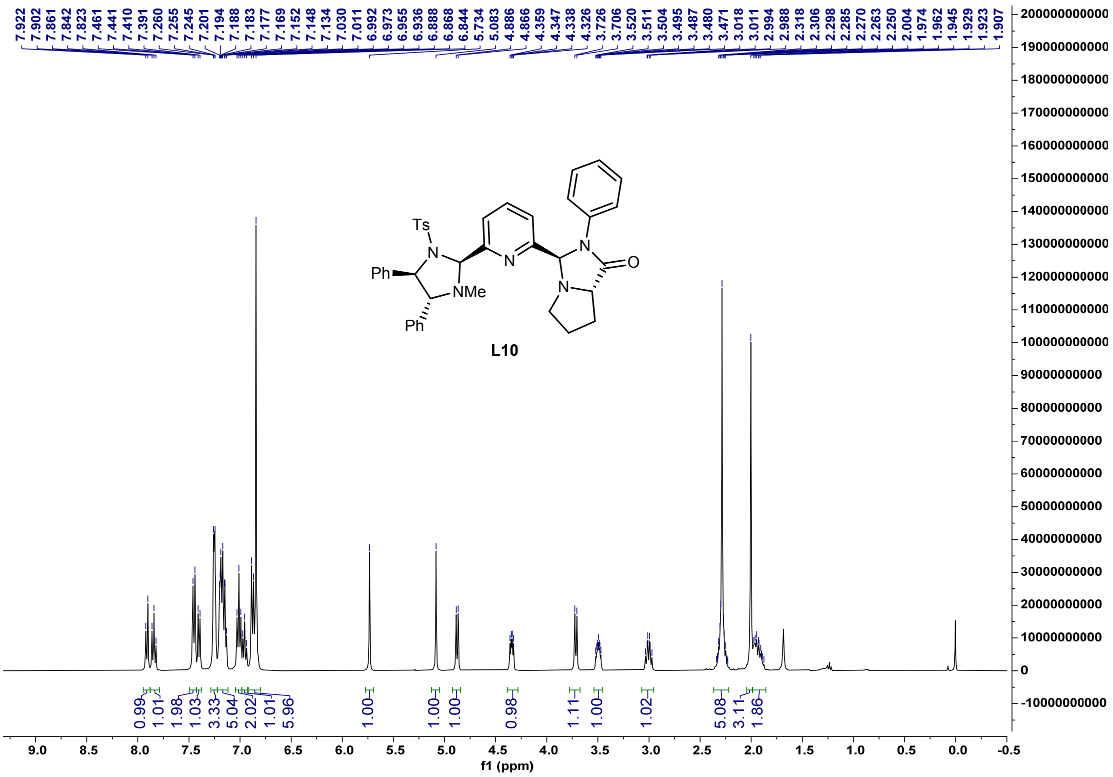


Supplementary Fig. 40 $^{13}$C NMR(100 MHz, $CDCl_3$)

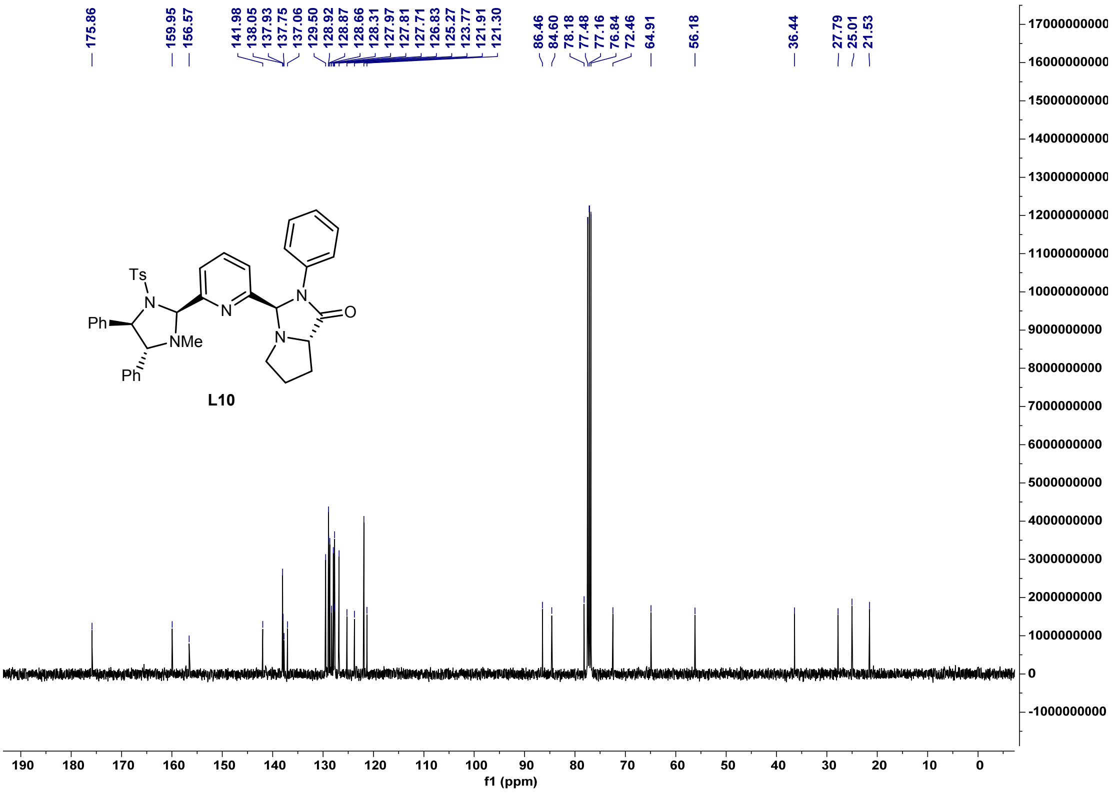

Supplementary Fig. 41 $^{1}H$ NMR(400 MHz, $CDCl_3$)

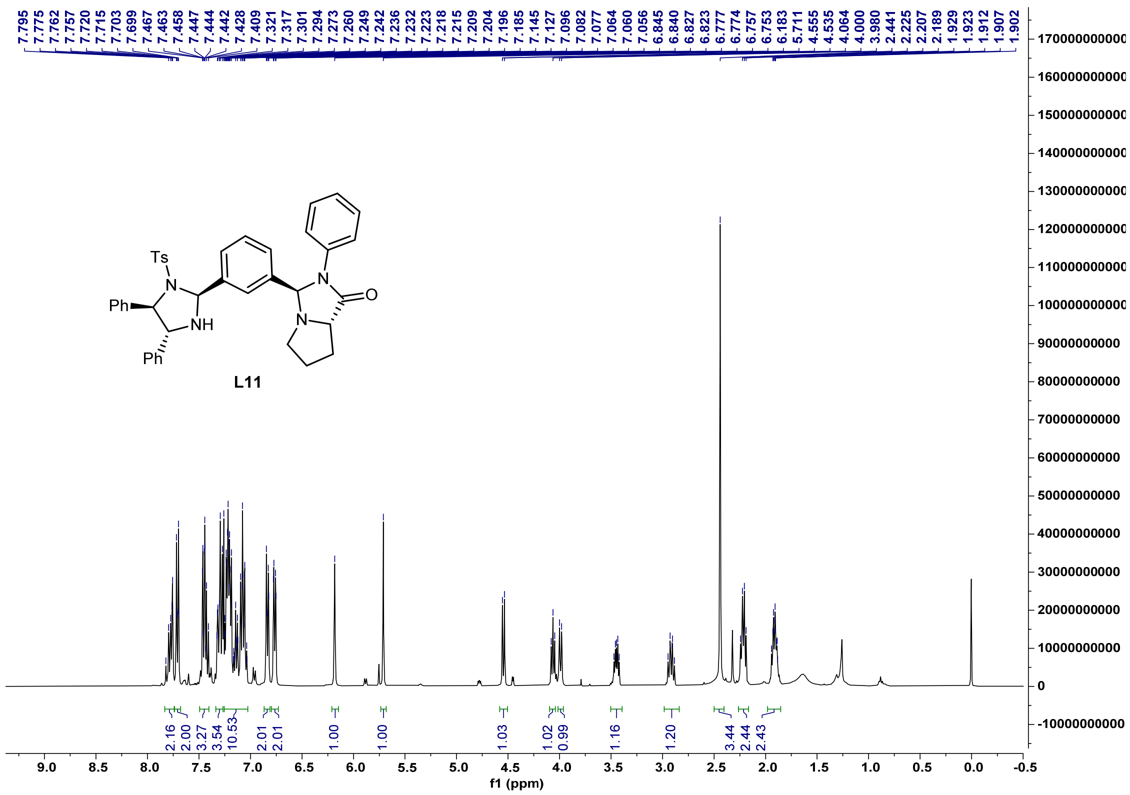


Supplementary Fig. 42 $^{13}C$ NMR(100 MHz, $CDCl_3$)

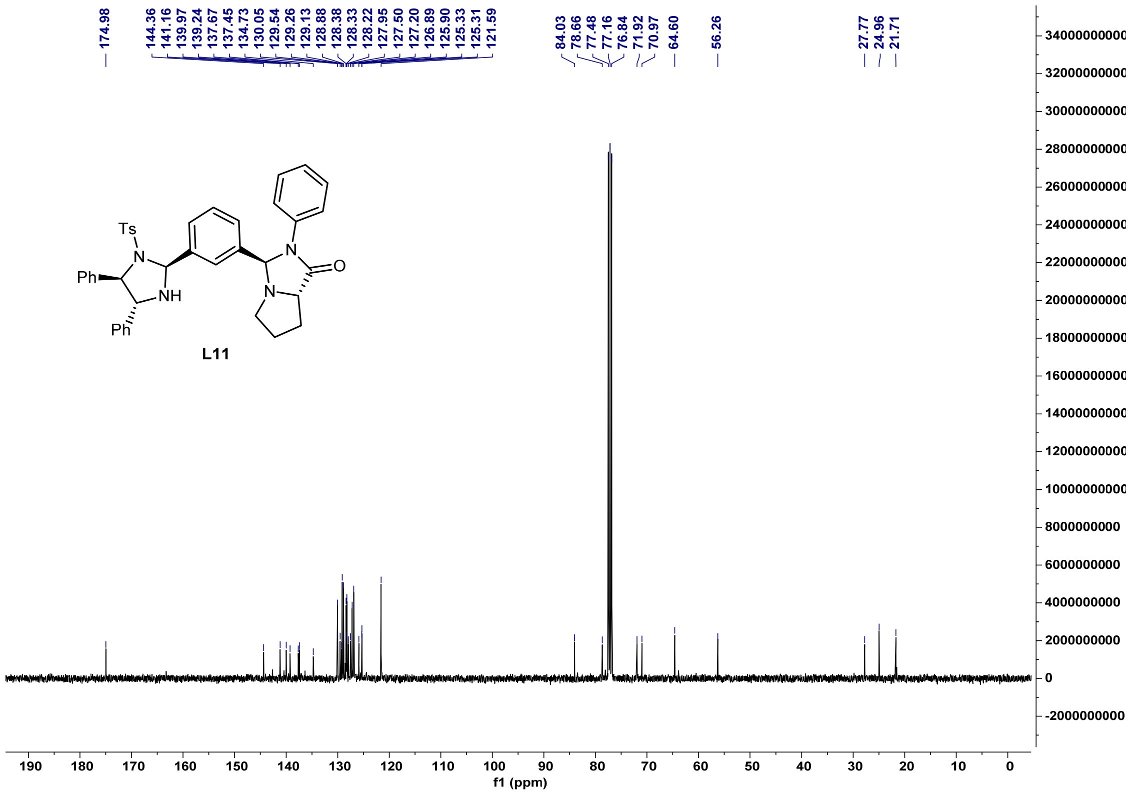

Supplementary Fig. 43 $^1$H NMR(400 MHz, $CDCl_3$)

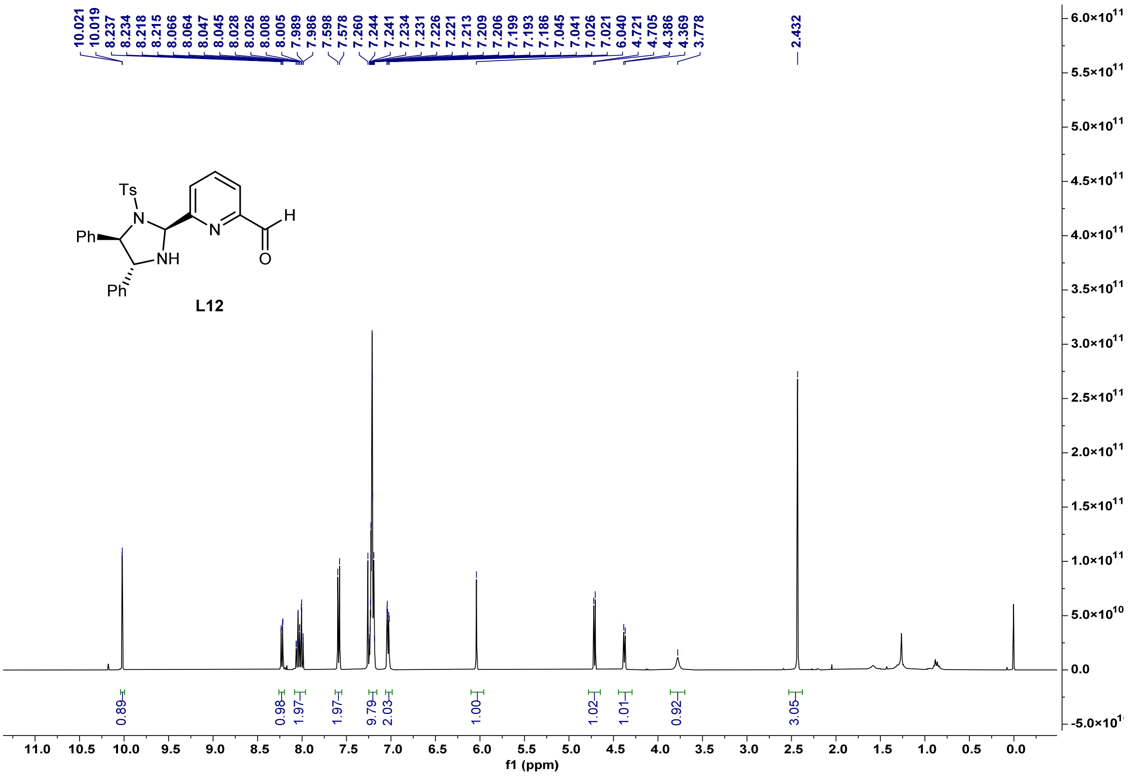


Supplementary Fig. 44 $^{13}$C NMR(100 MHz, $CDCl_3$)

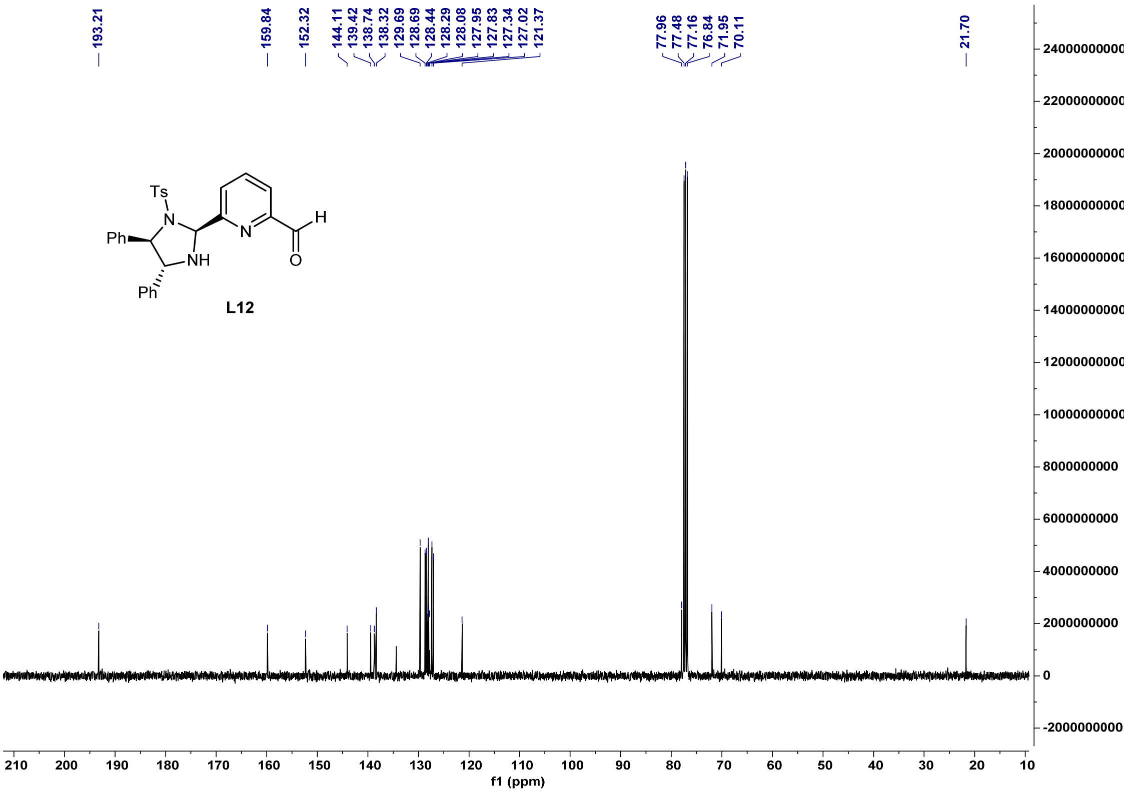

Supplementary Fig. 45 $^{1}H$ NMR(400 MHz, $CDCl_3$)

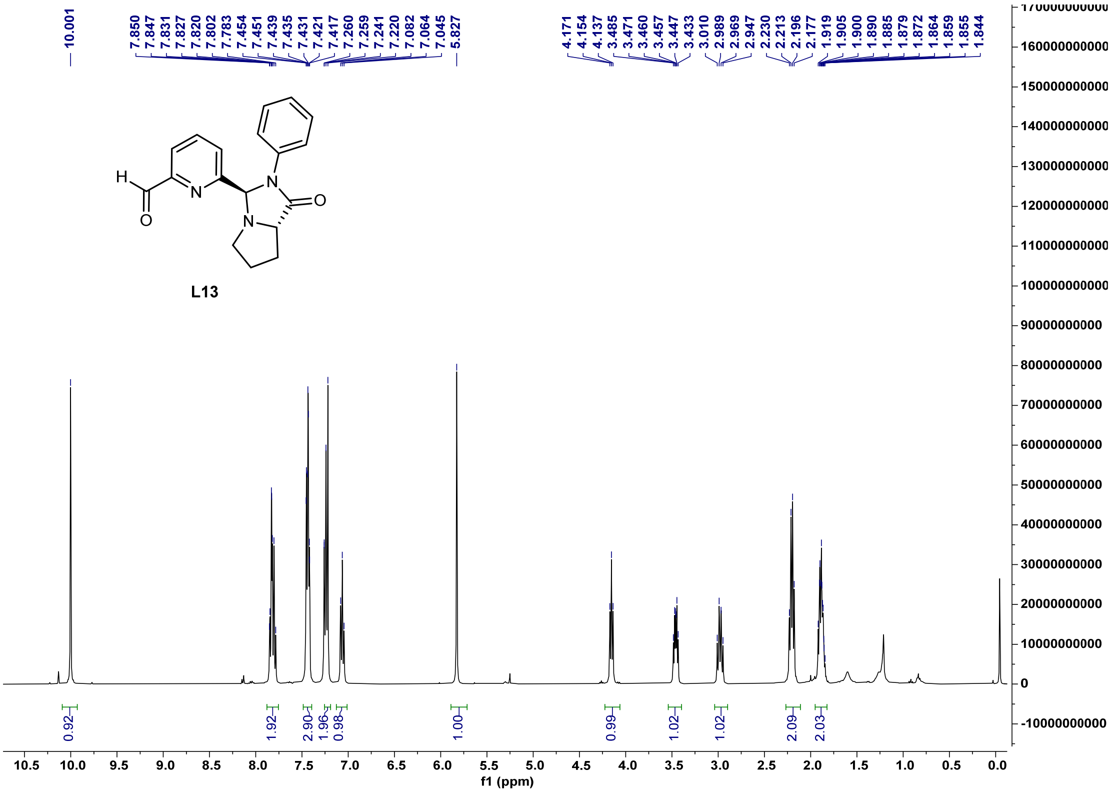


Supplementary Fig. 46 $^{13}C$ NMR(100 MHz, $CDCl_3$)

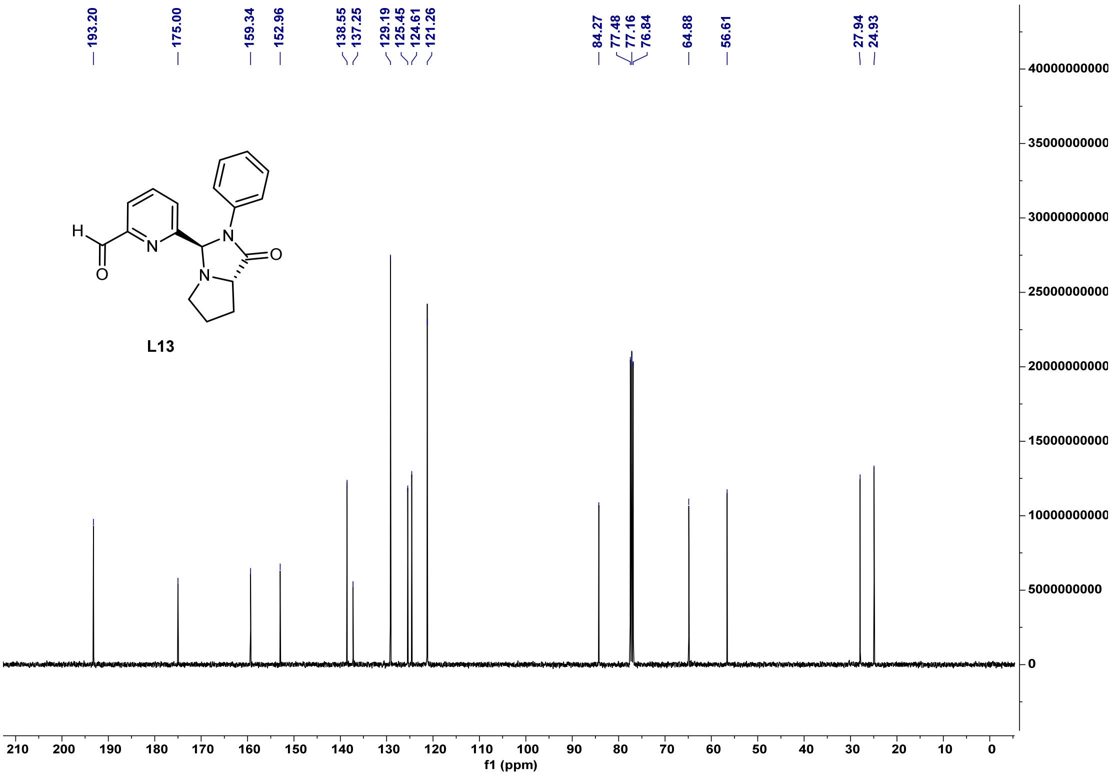

Supplementary Fig. 47 $^{1}$H NMR(400 MHz, $CDCl_3$)

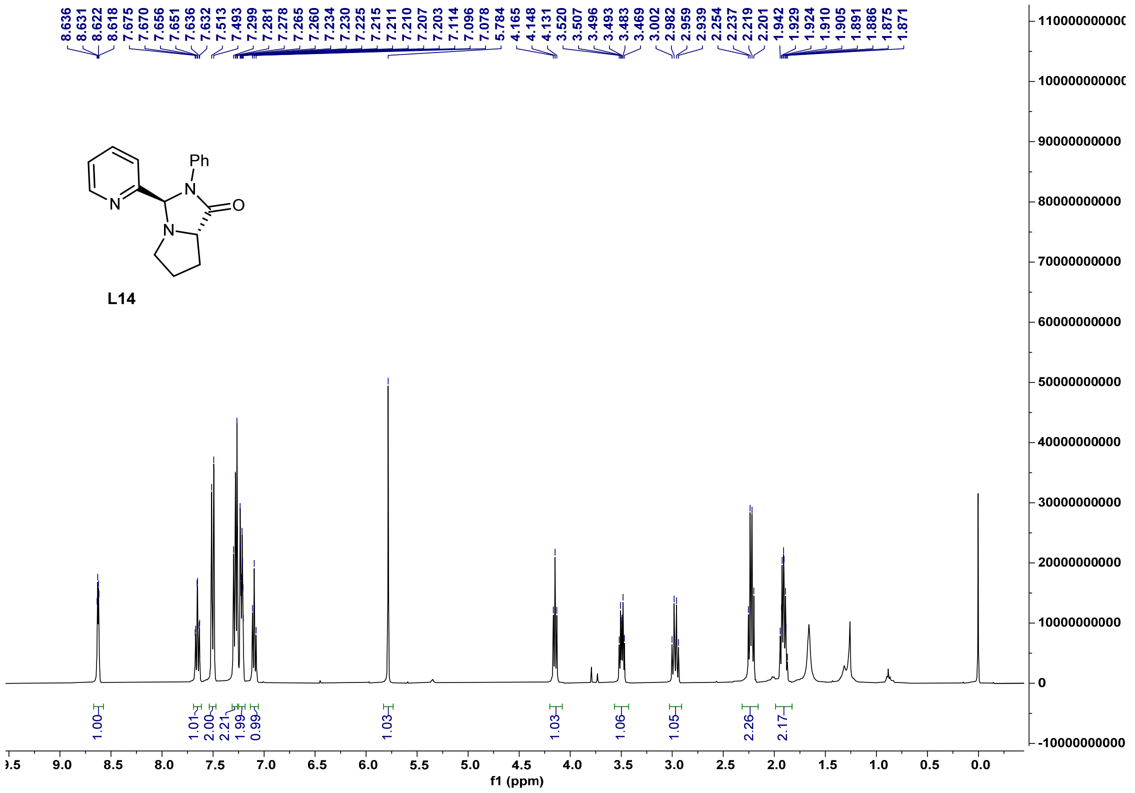


Supplementary Fig. 48 $^{13}$C NMR(100 MHz, $CDCl_3$)

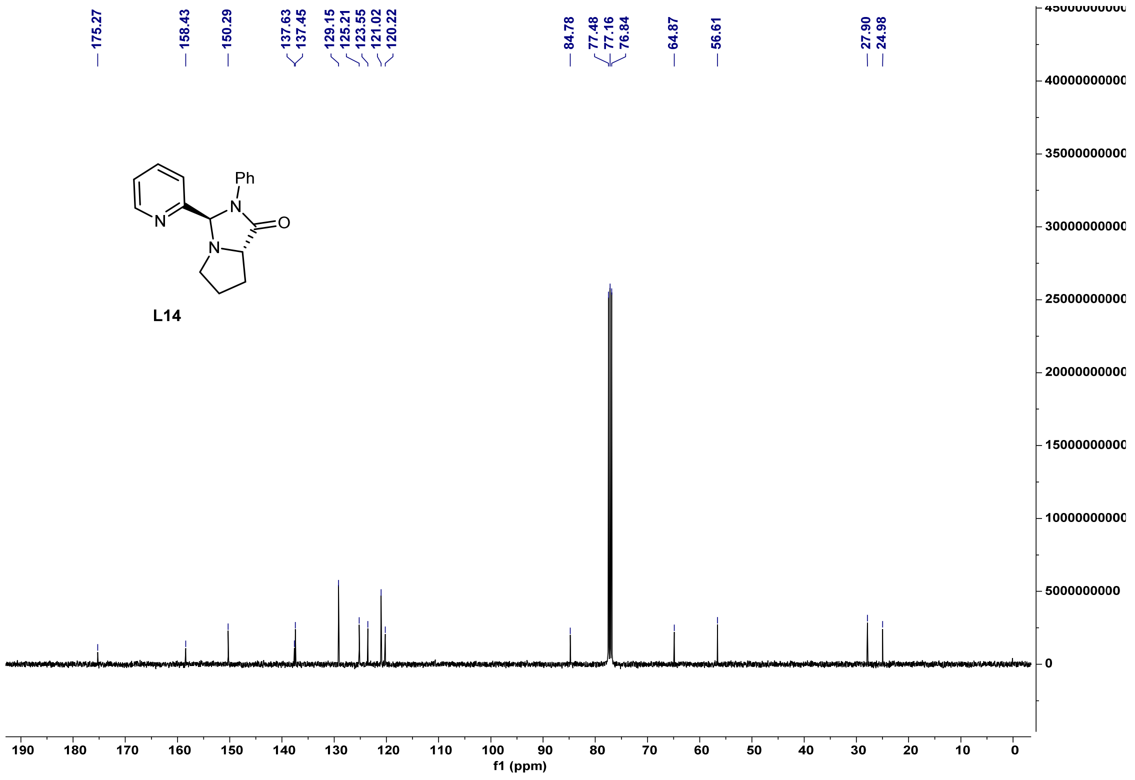

Supplementary Fig. 49 $^{1}$H NMR(400 MHz, $CDCl_3$)

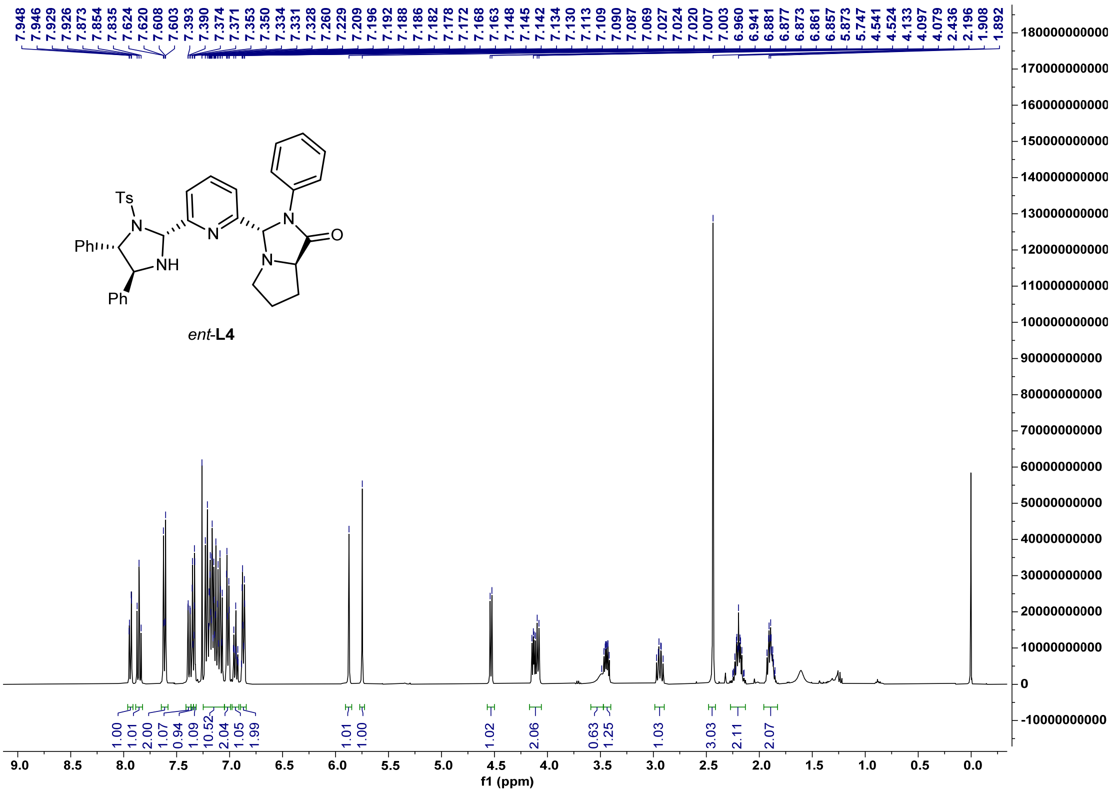

Supplementary Fig. 50 $^{13}$C NMR(100 MHz, $CDCl_3$)

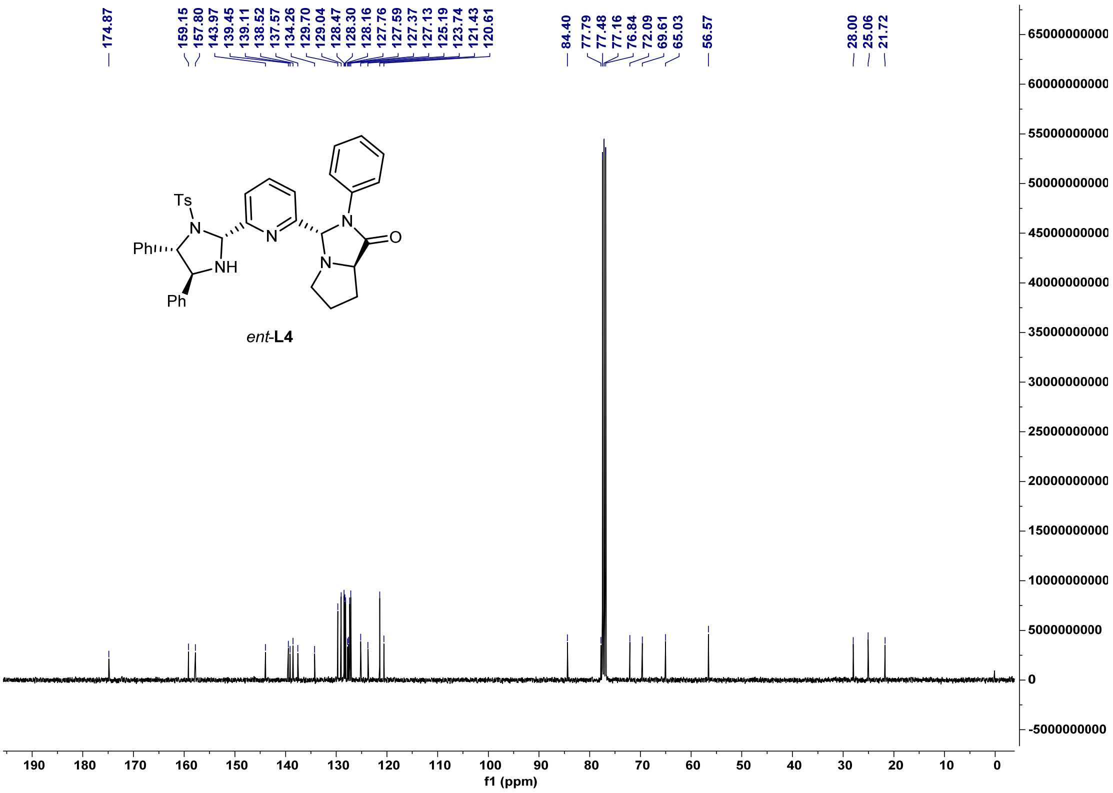

Supplementary Fig. 51 $^{1}$H NMR(400 MHz, $CDCl_3$)

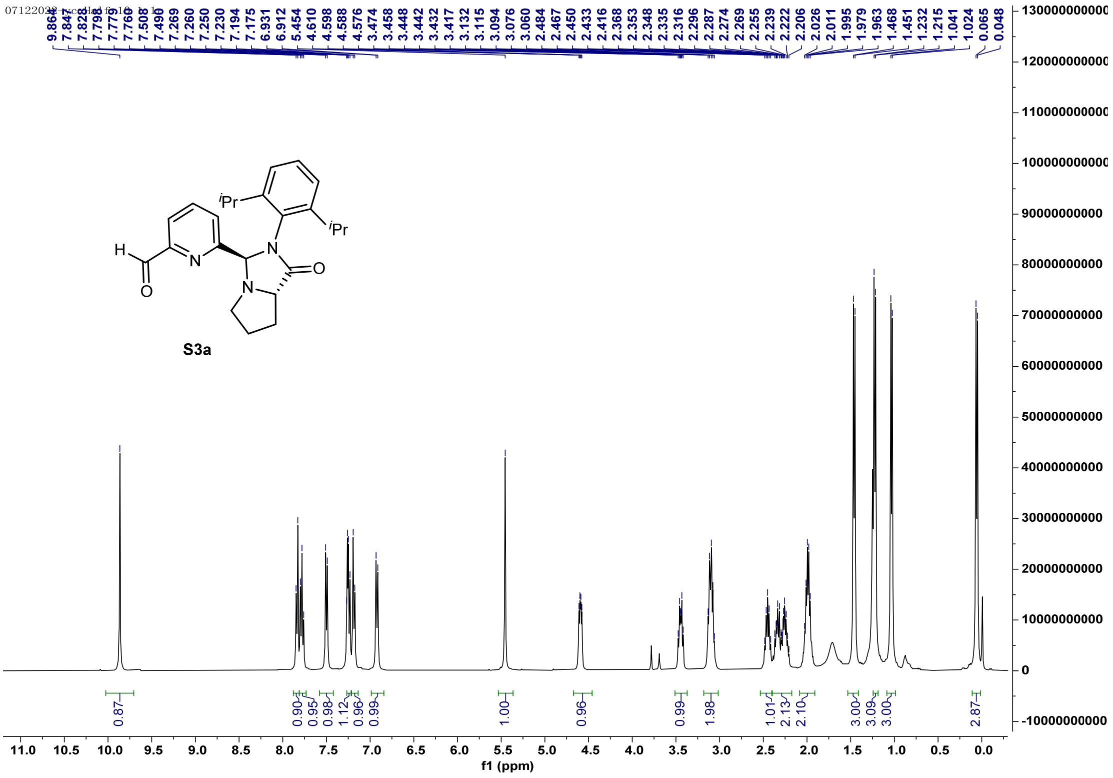


Supplementary Fig. 52 $^{13}$C NMR(100 MHz, $CDCl_3$)

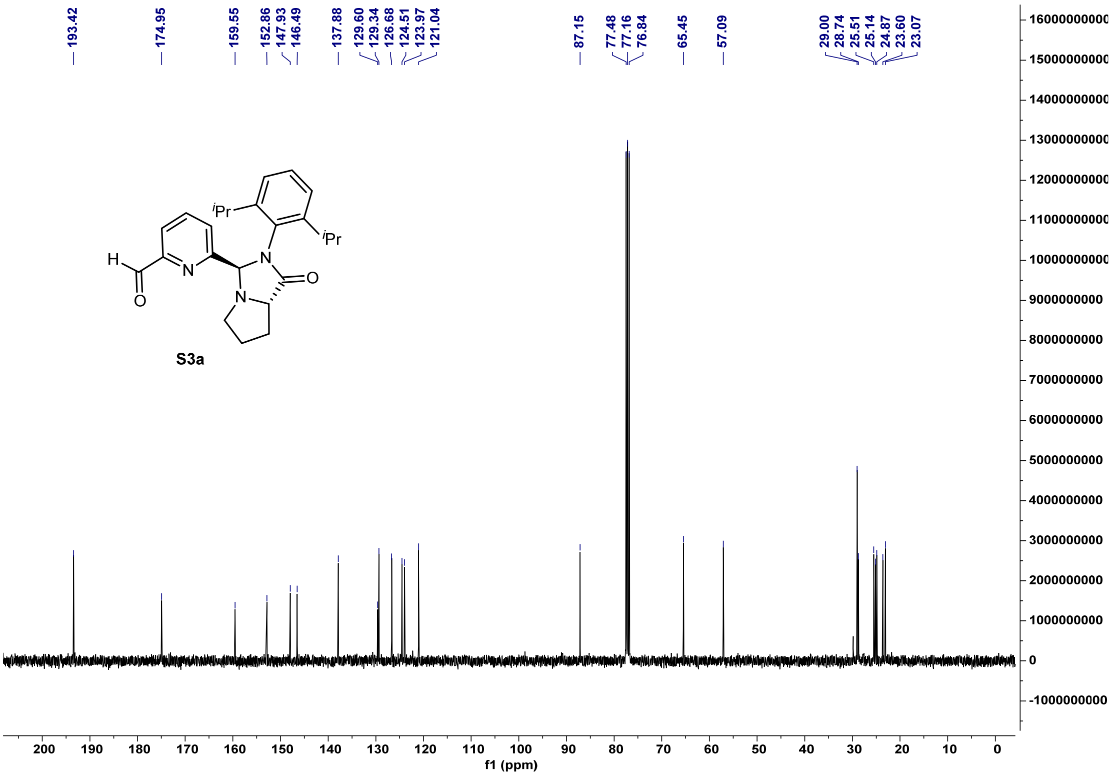

Supplementary Fig. 53 $^{1}H$ NMR(400 MHz, $CDCl_3$)

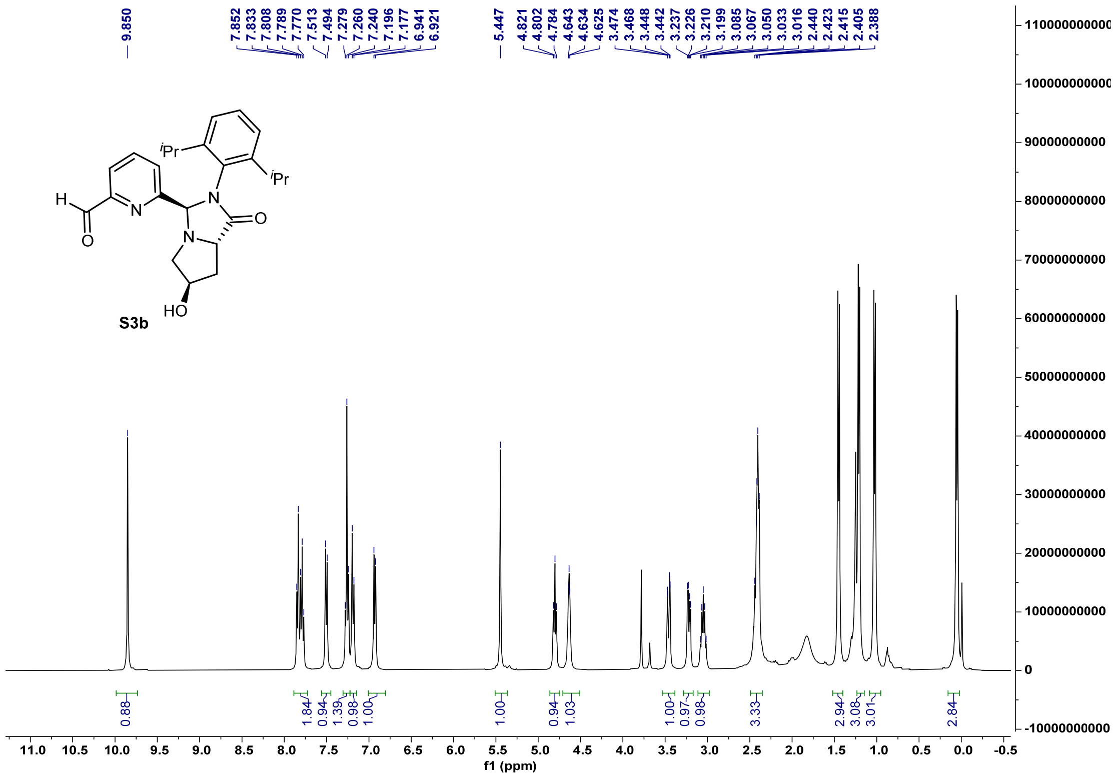


Supplementary Fig. 54 $^{13}C$ NMR(100 MHz, $CDCl_3$)

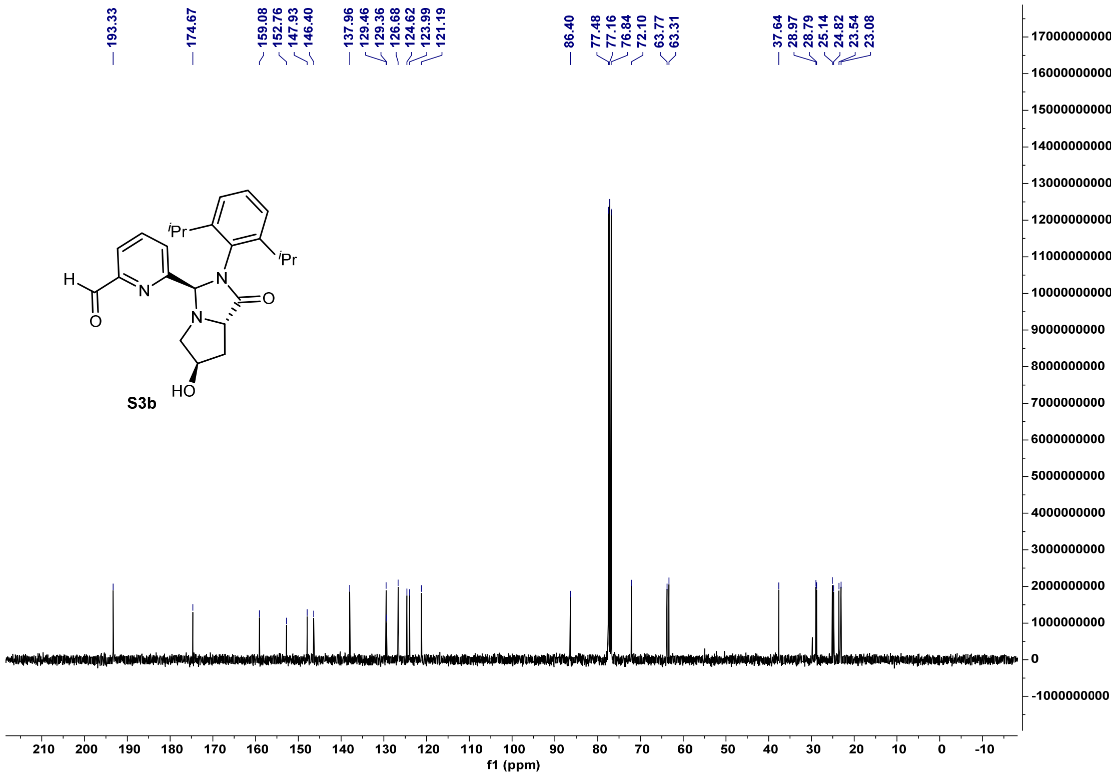

Supplementary Fig. 55 $^{1}$H NMR(600 MHz, $CDCl_3$)

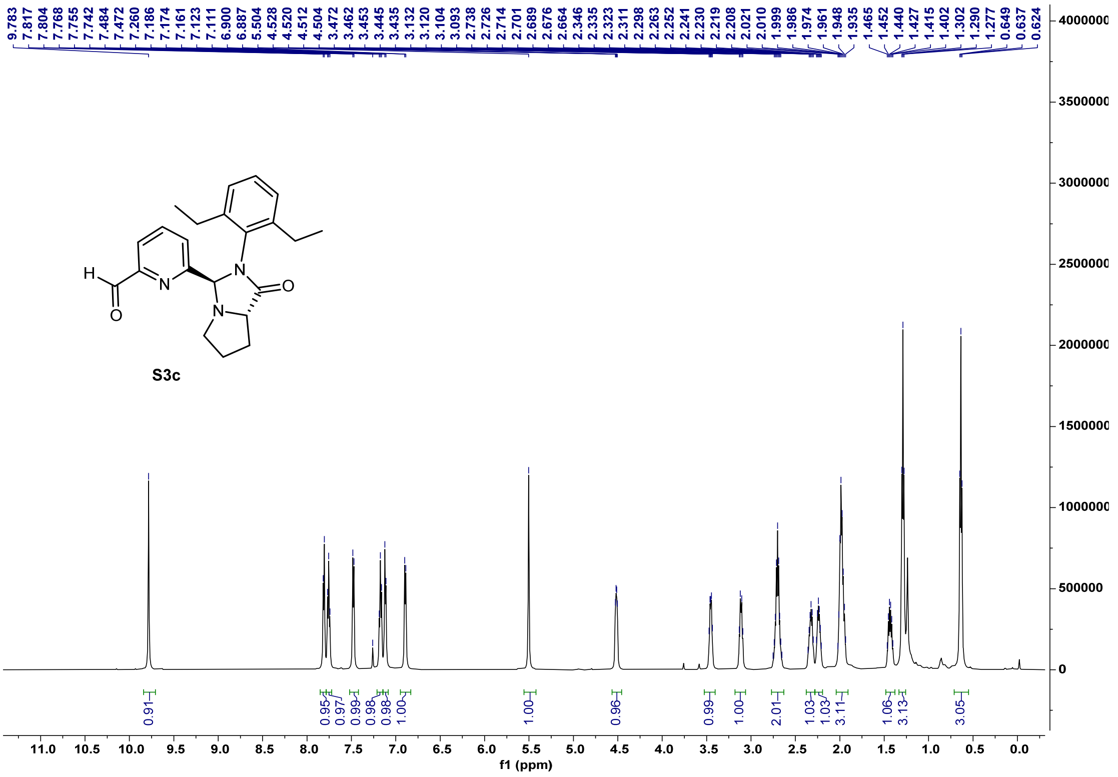


Supplementary Fig. 56 $^{13}$C NMR(100 MHz, $CDCl_3$)

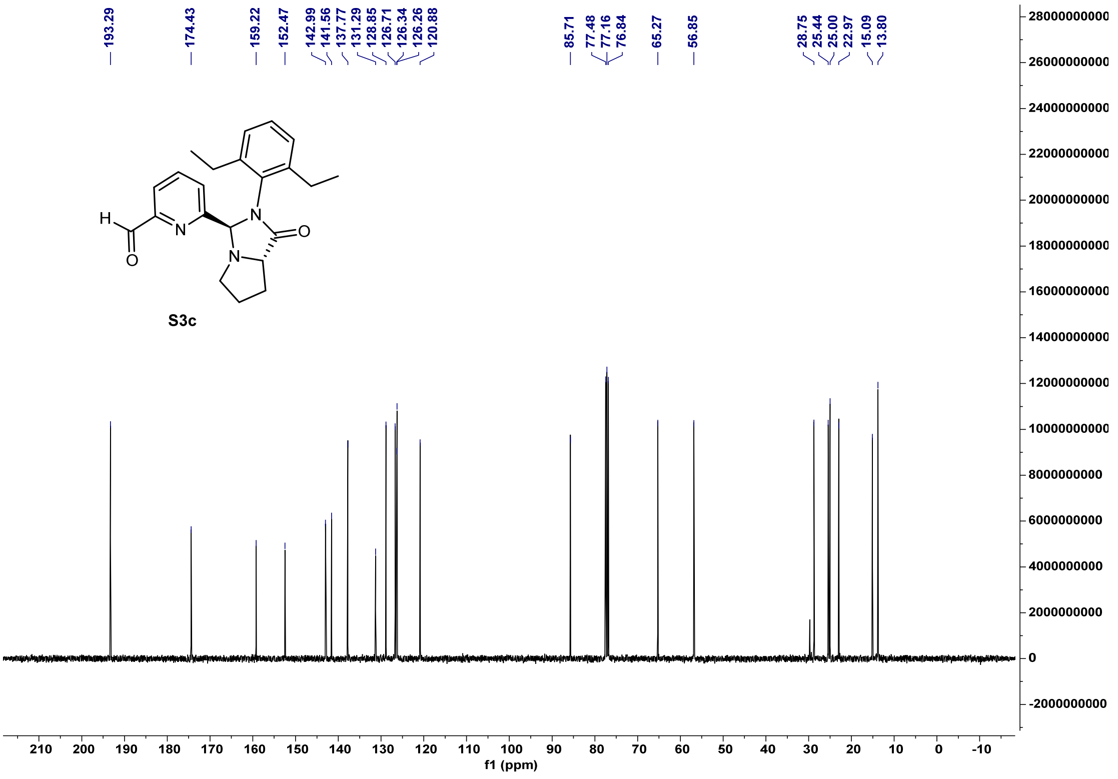

Supplementary Fig. 57 $^{1}$H NMR(400 MHz, $CDCl_3$)

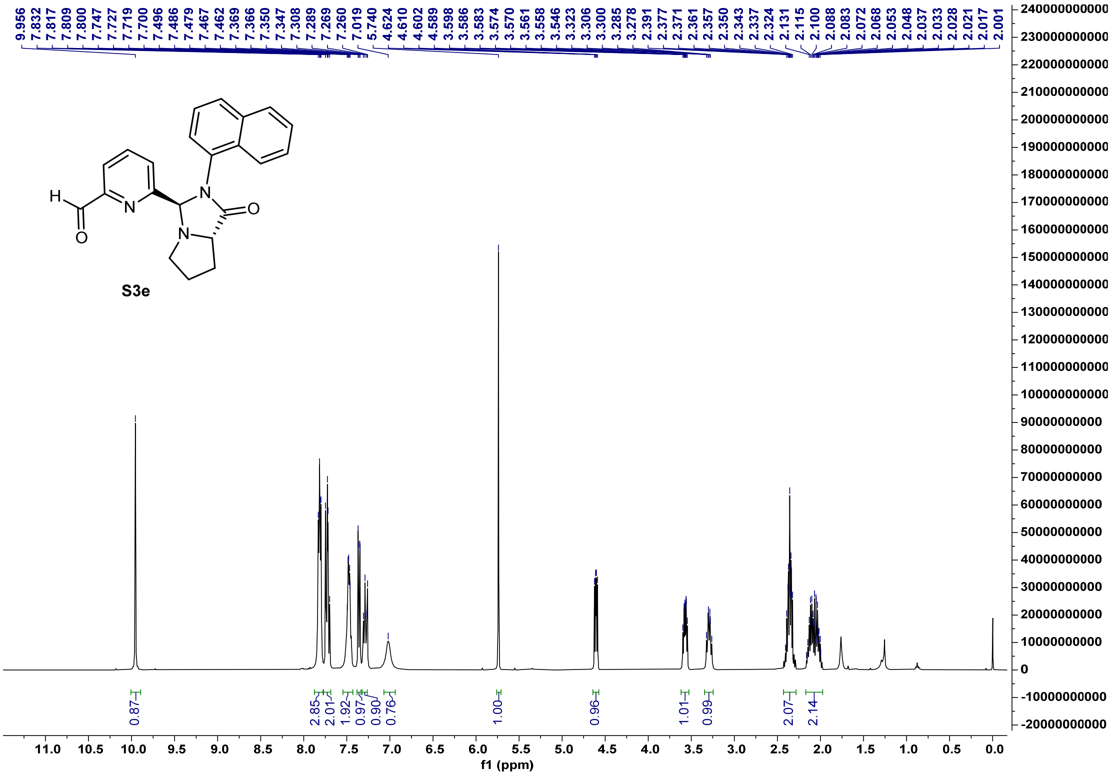


Supplementary Fig. 58 $^{13}$C NMR(100 MHz, $CDCl_3$)

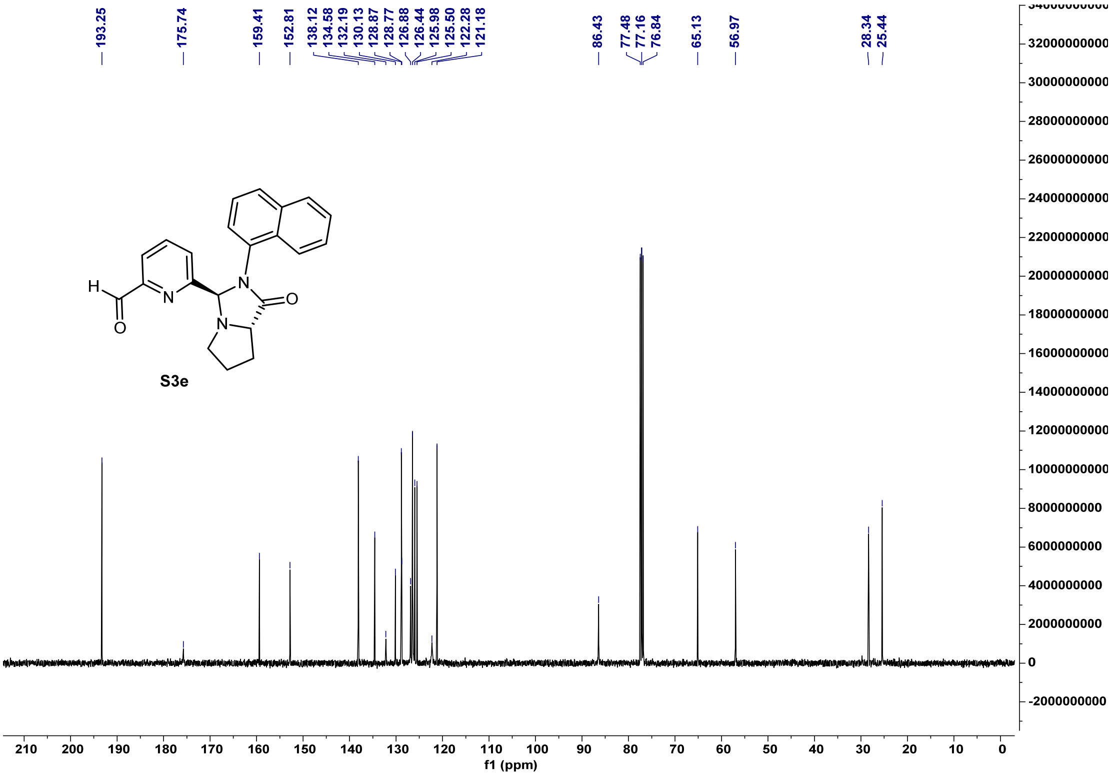

Supplementary Fig. 59 $^{1}$H NMR(600 MHz, $CDCl_3$)

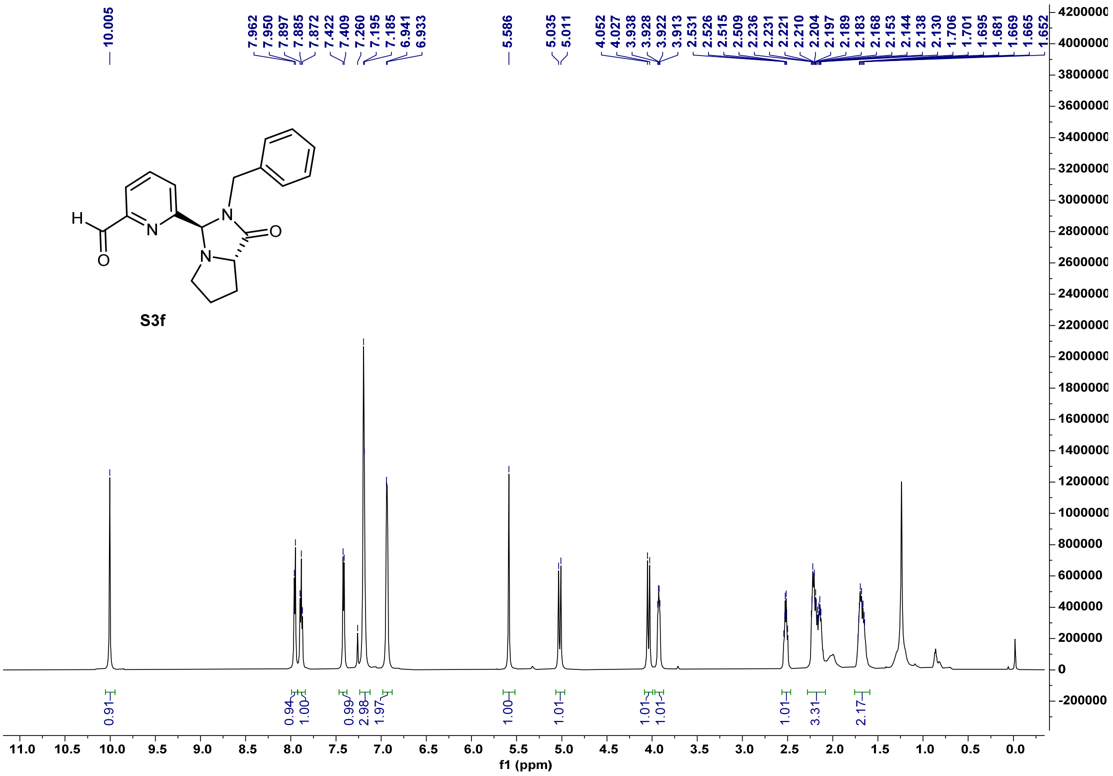


Supplementary Fig. 60 $^{13}$C NMR(100 MHz, $CDCl_3$)

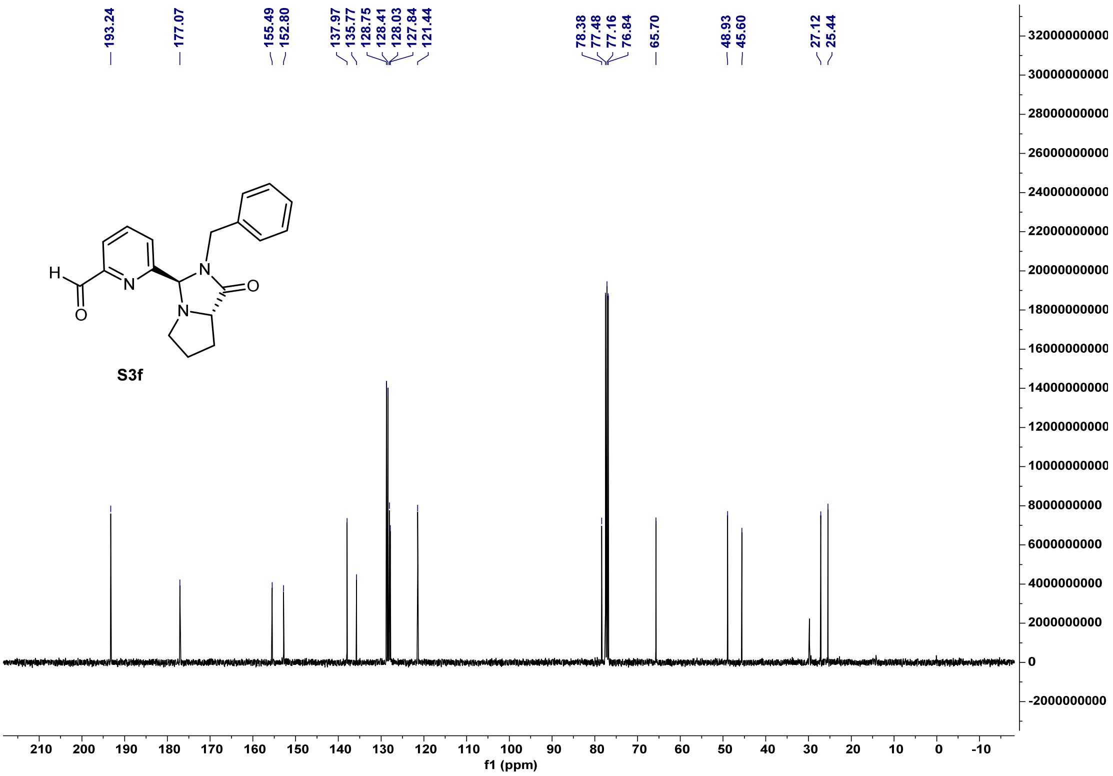

Supplementary Fig. 61 $^{1}H$ NMR(400 MHz, $CDCl_3$)

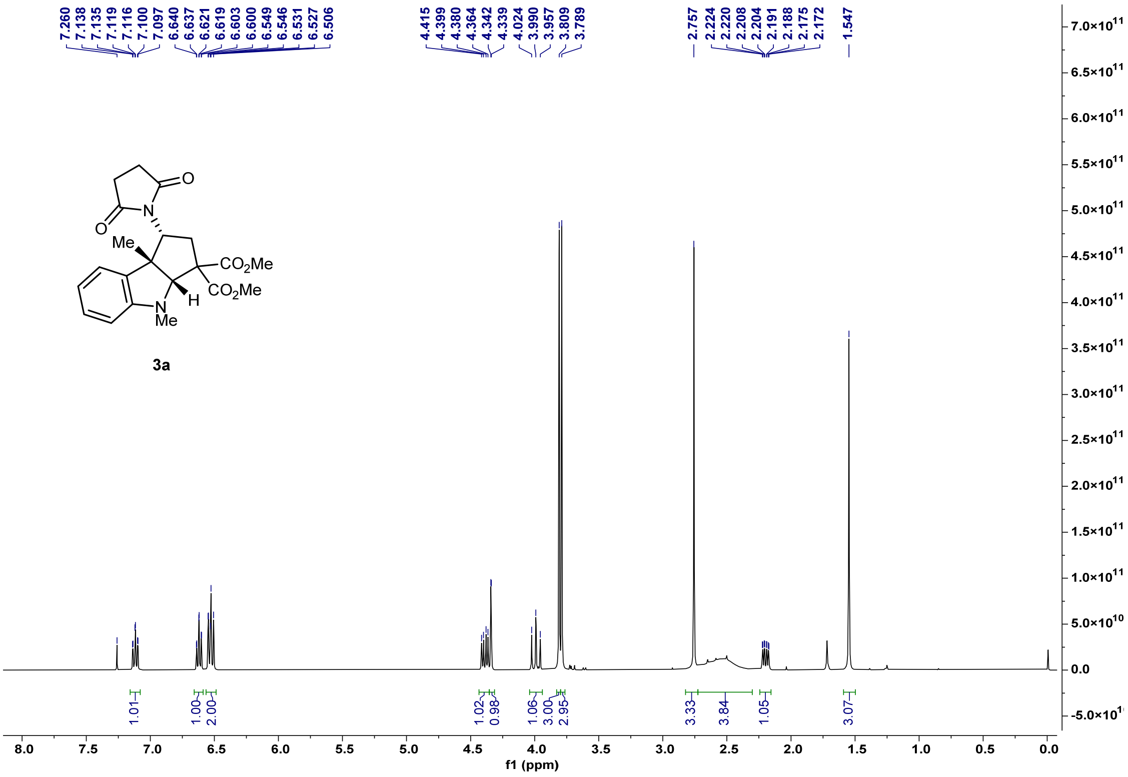

Supplementary Fig. 62 $^{13}C$ NMR(100 MHz, $CDCl_3$)

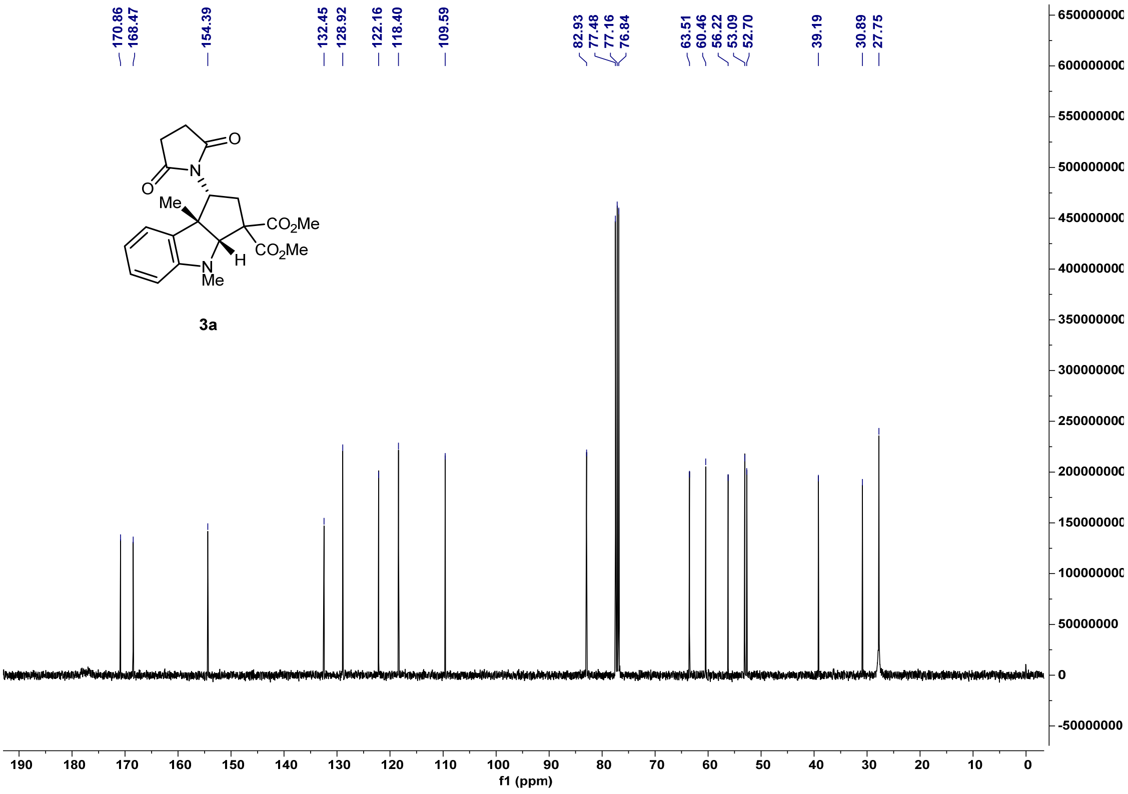

Supplementary Fig. 63 $^1$H NMR(400 MHz, $CDCl_3$)

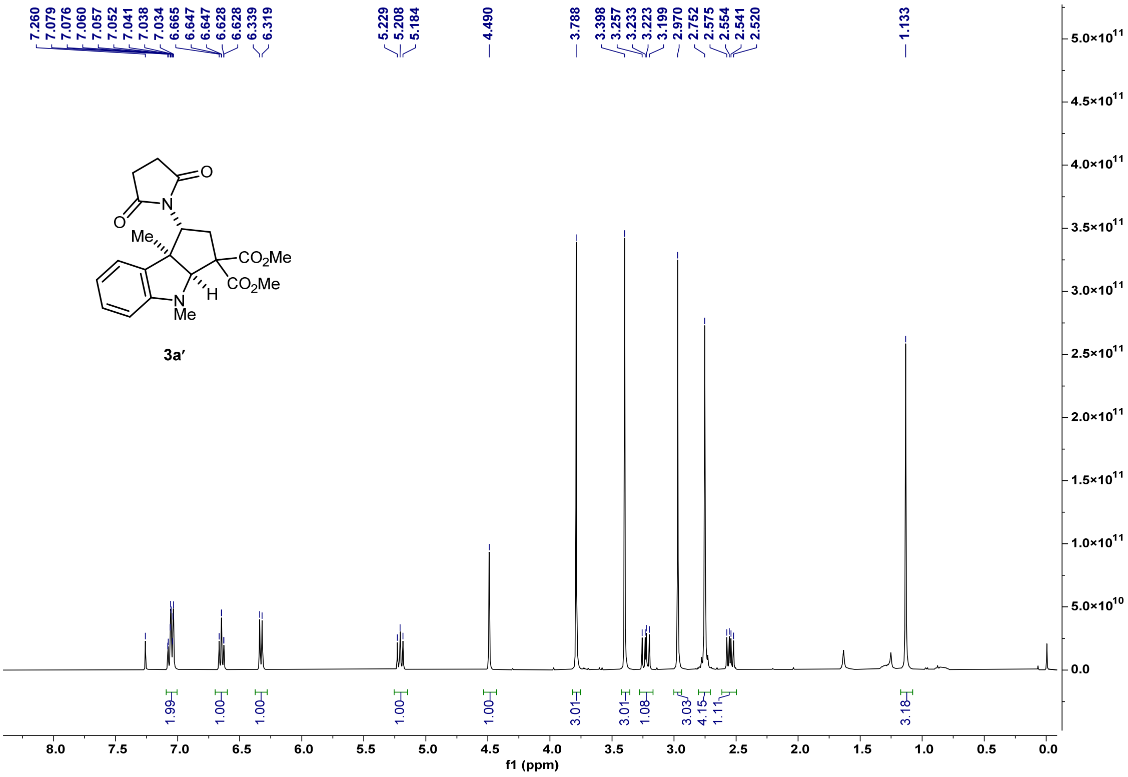


Supplementary Fig. 64 $^{13}$C NMR(100 MHz, $CDCl_3$)

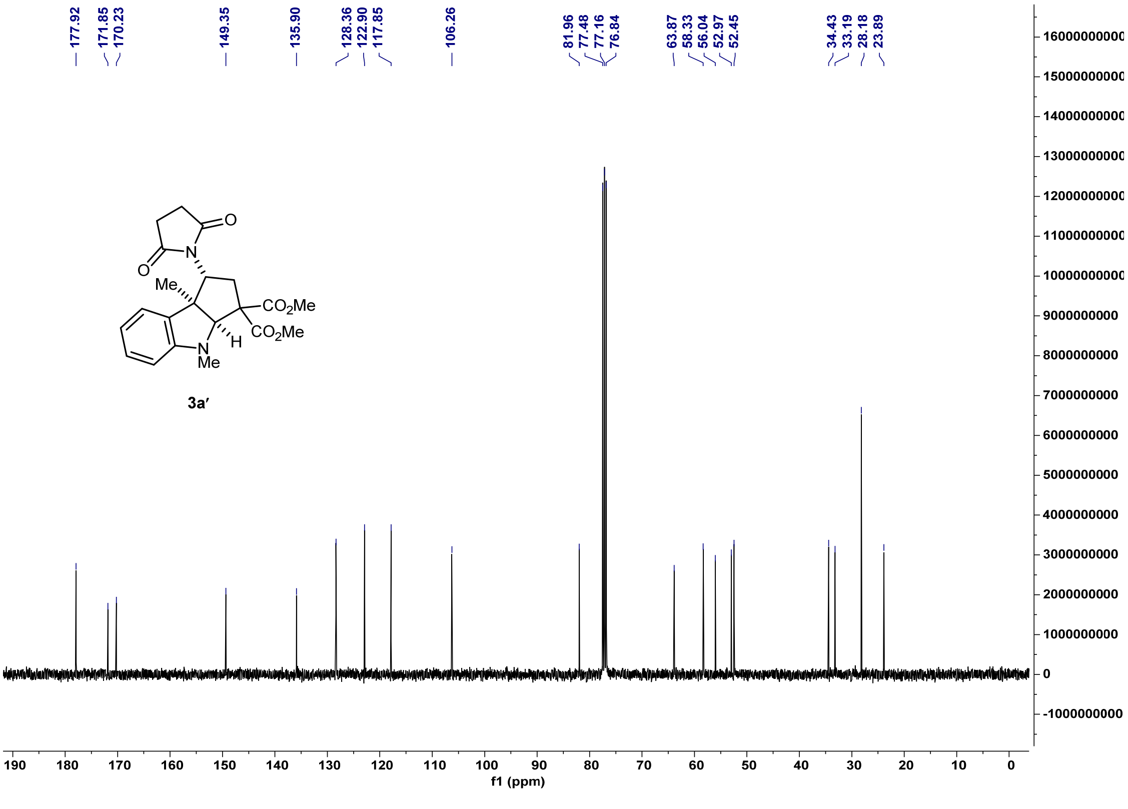

Supplementary Fig. 65 $^{1}$H NMR(600 MHz, $CDCl_3$)

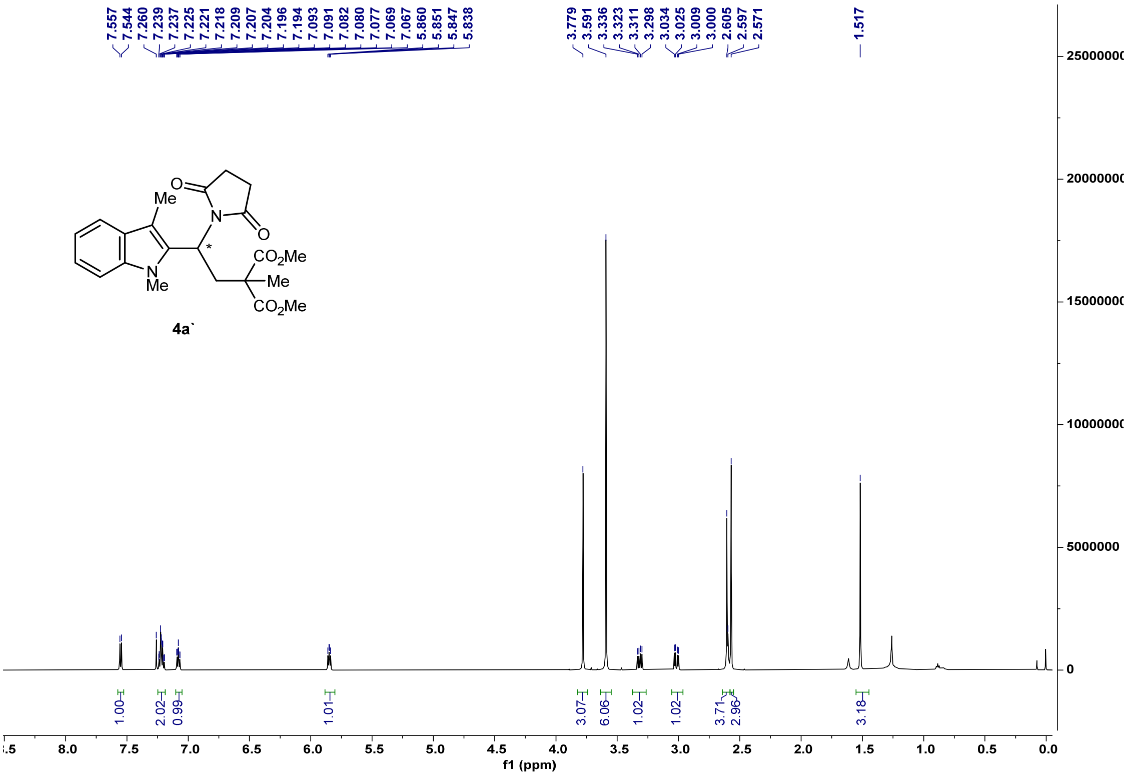

Supplementary Fig. 66 $^{13}$C NMR(150 MHz, $CDCl_3$)

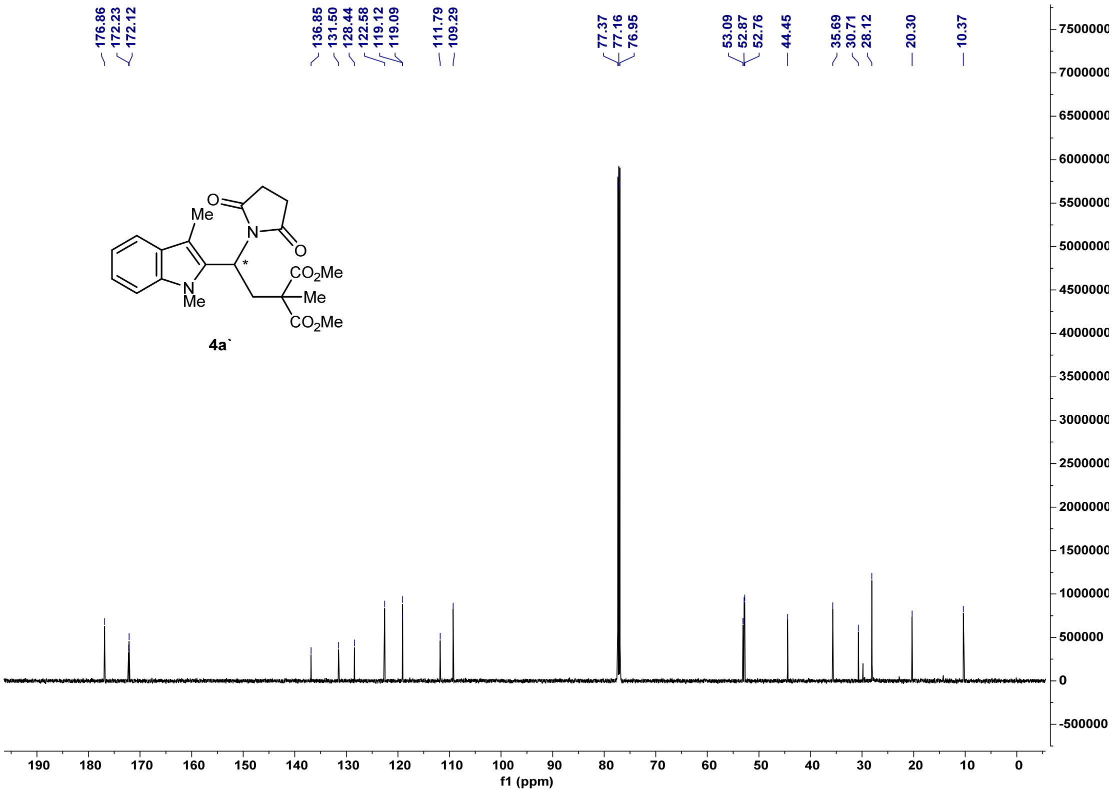

Supplementary Fig. 67 $^{1}H$ NMR(400 MHz, $CDCl_3$)

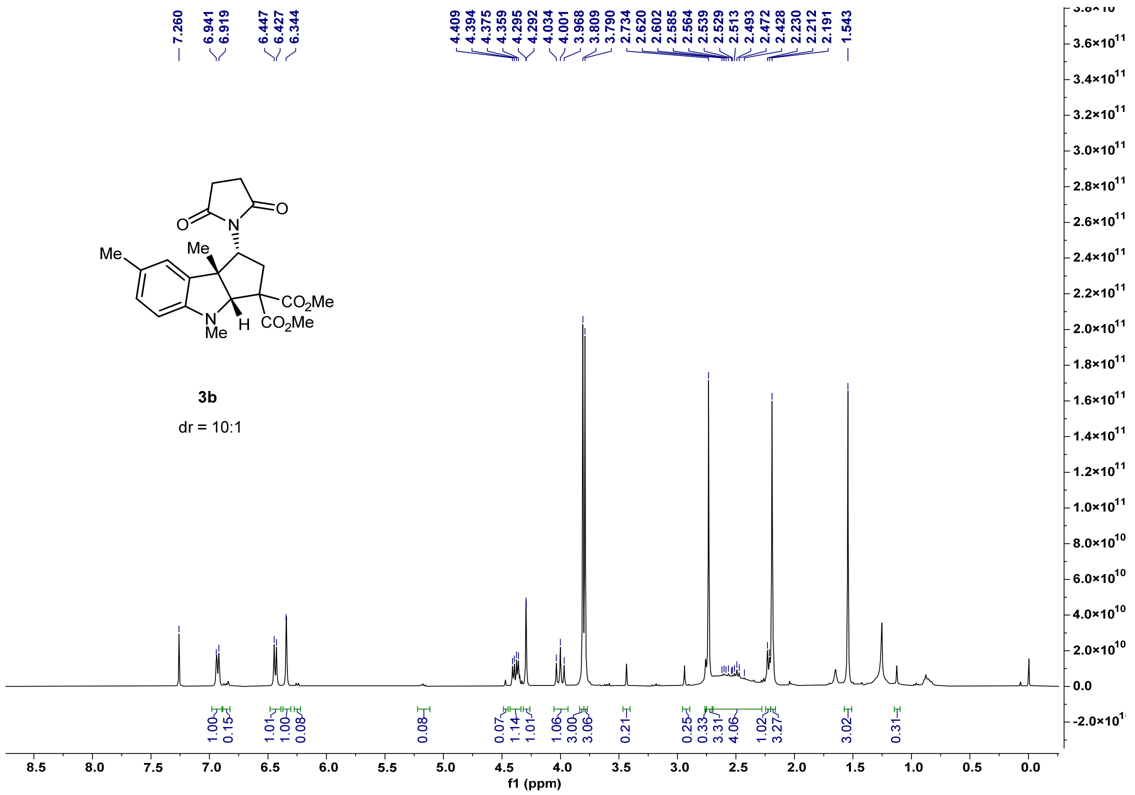


Supplementary Fig. 68 $^{13}C$ NMR(100 MHz, $CDCl_3$)

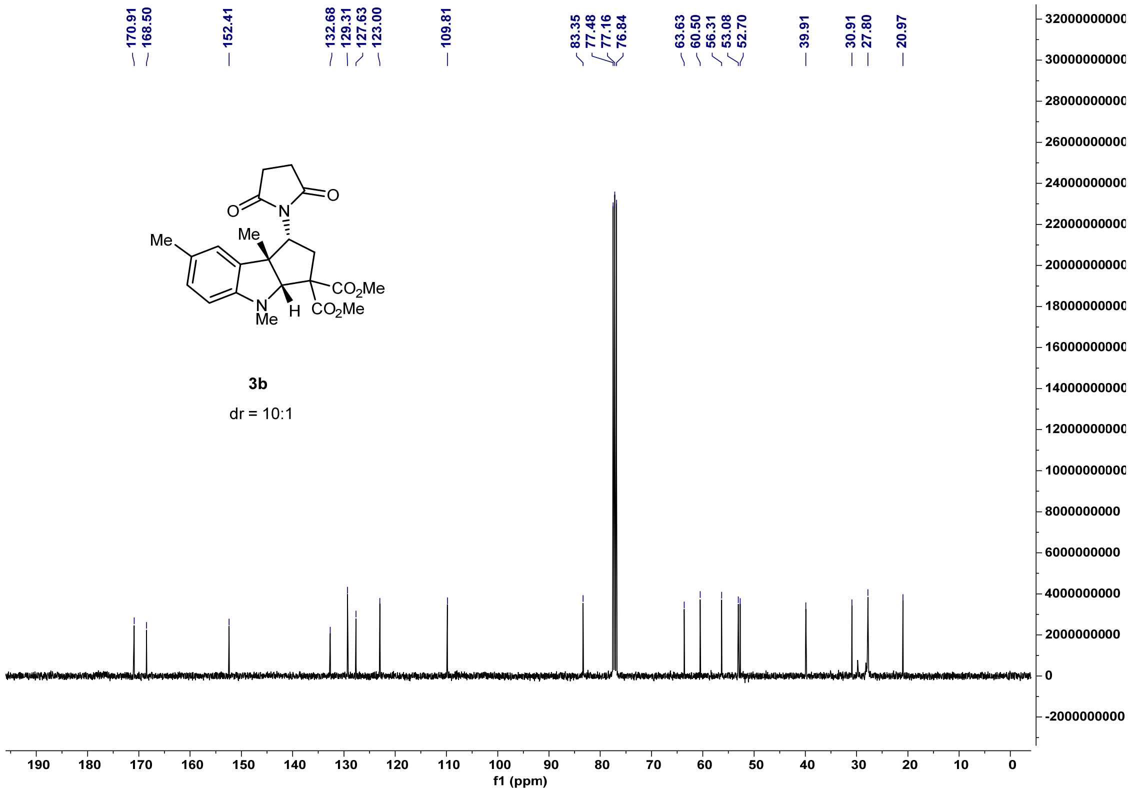

Supplementary Fig. 69 $^{1}$H NMR(400 MHz, $CDCl_3$)

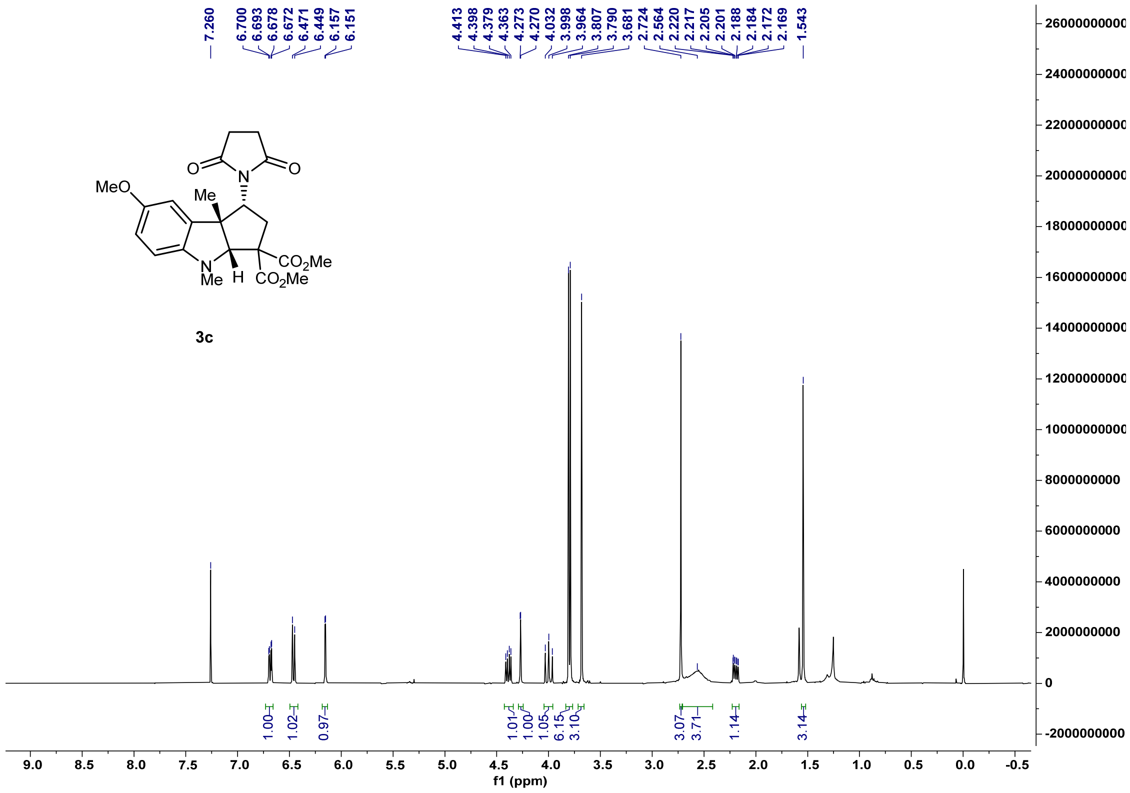

Supplementary Fig. 70 $^{13}$C NMR(100 MHz, $CDCl_3$)

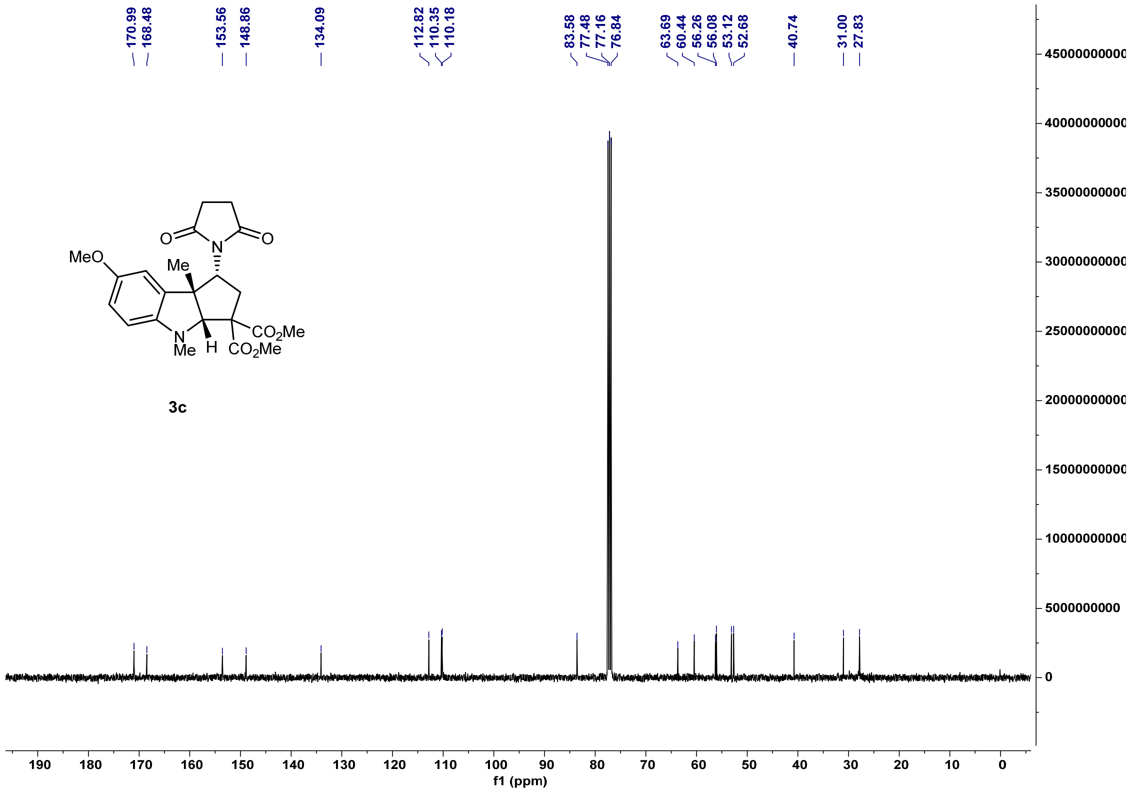

Supplementary Fig. 71 $^{1}$H NMR(400 MHz, $CDCl_3$)

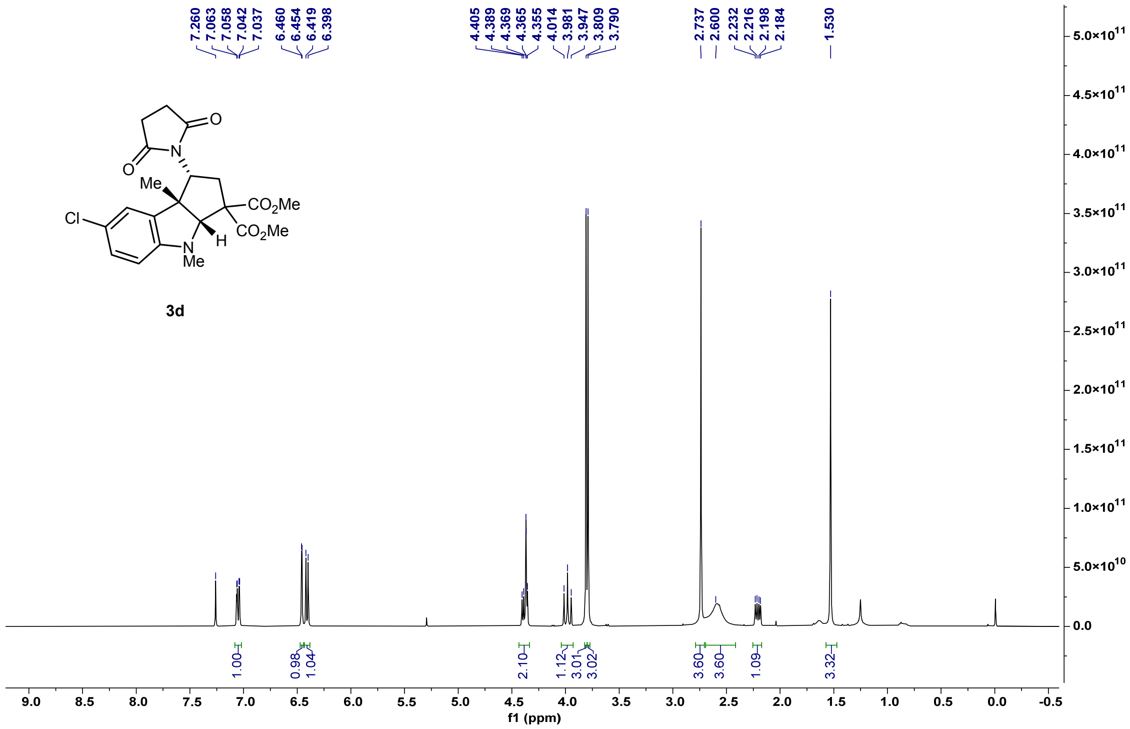


Supplementary Fig. 72 $^{13}$C NMR(100 MHz, $CDCl_3$)

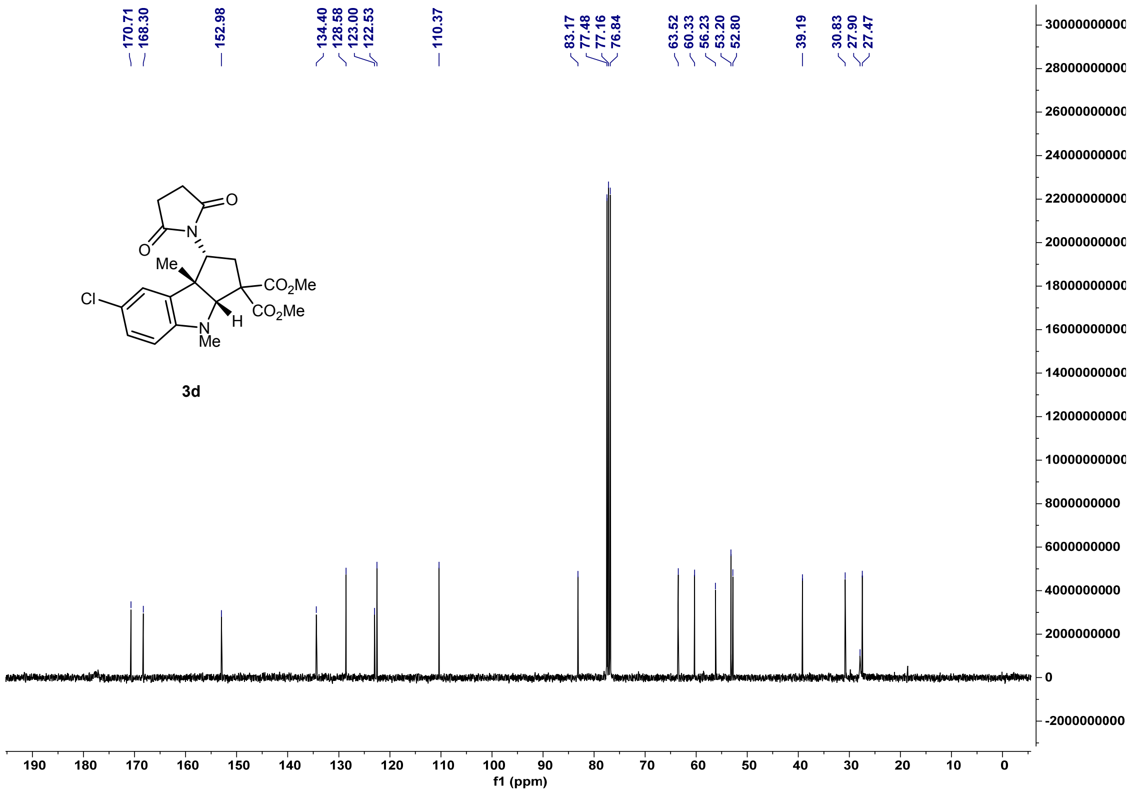

Supplementary Fig. 73 $^1$H NMR(600 MHz, $CDCl_3$)

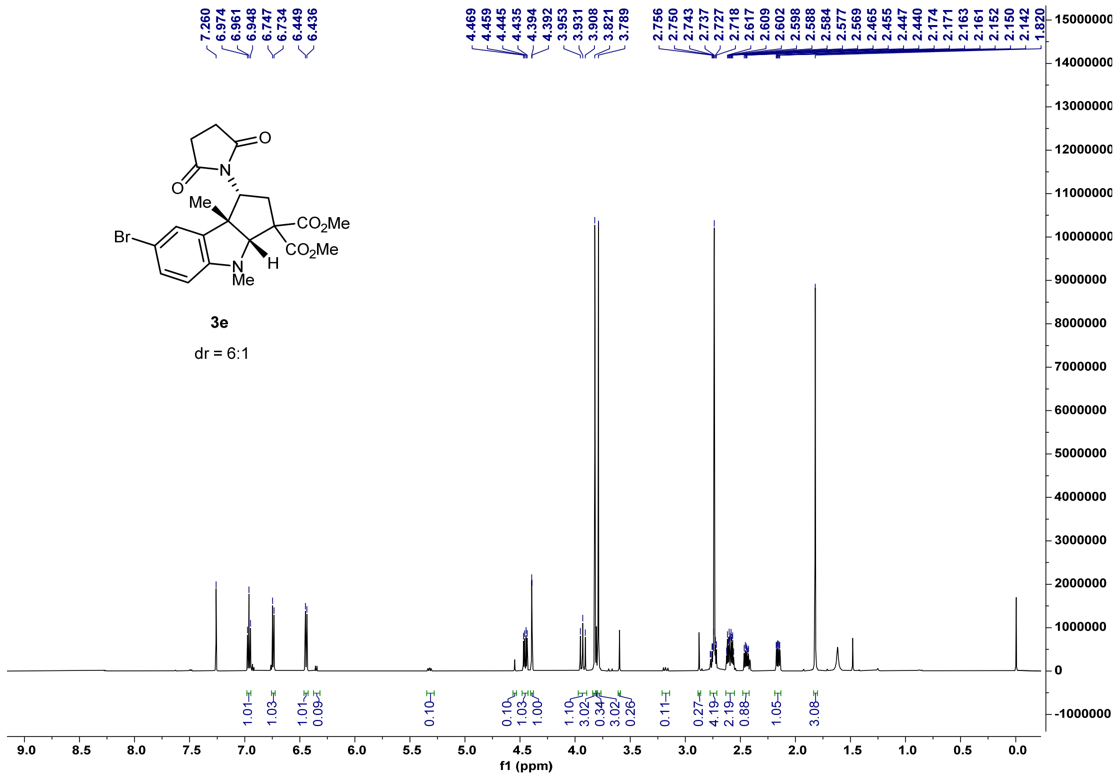

Supplementary Fig. 74 $^{13}$C NMR(150 MHz, $CDCl_3$)

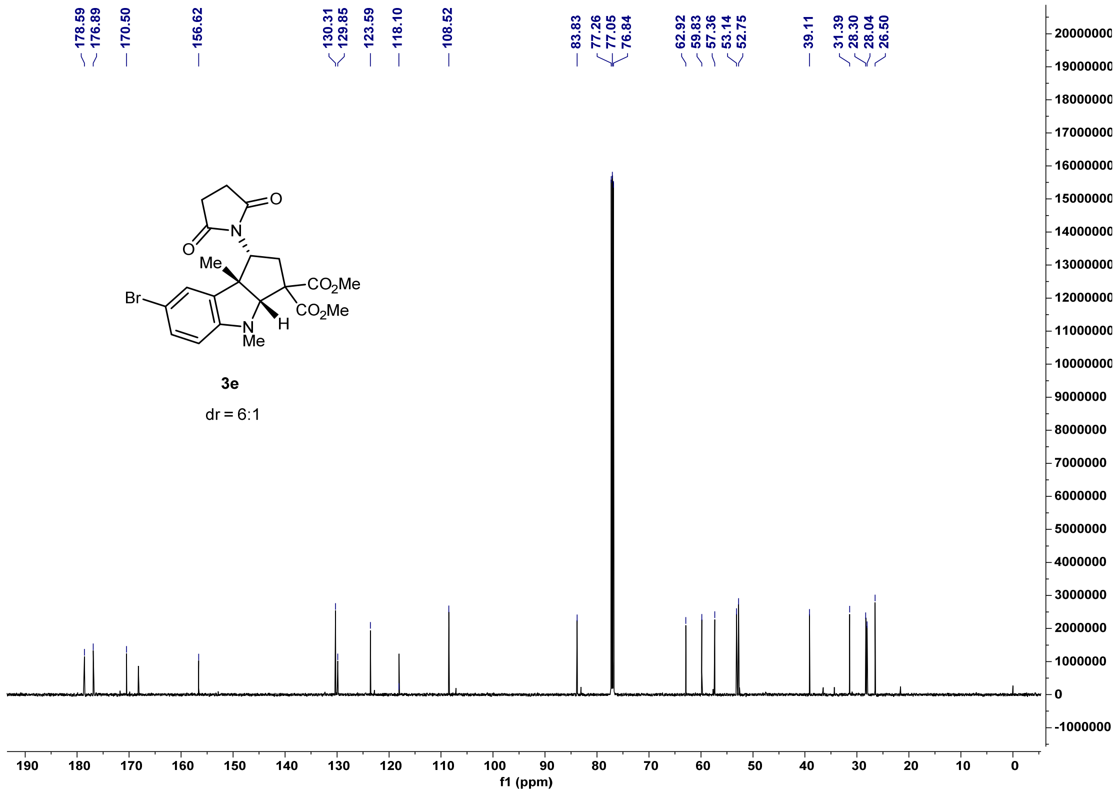

Supplementary Fig. 75 $^{1}H$ NMR(600 MHz, $CDCl_3$)

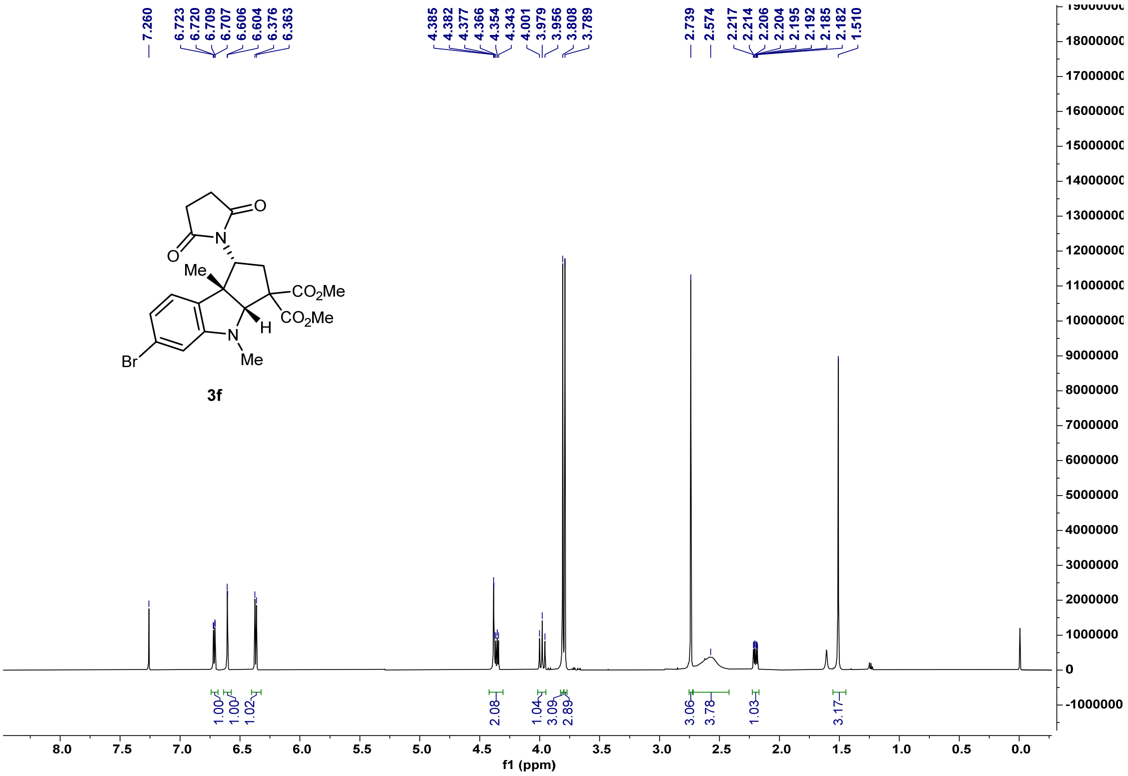

Supplementary Fig. 76 $^{13}C$ NMR(150 MHz, $CDCl_3$)

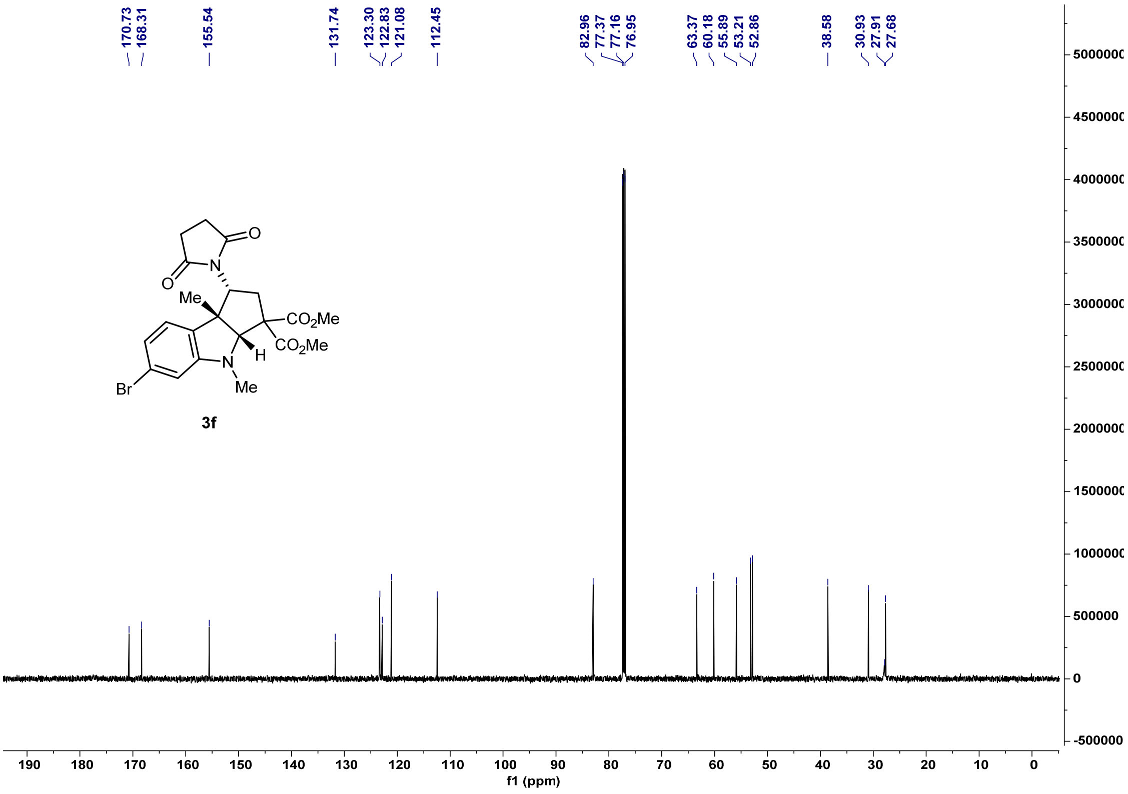

Supplementary Fig. 77 $^{1}H$ NMR(400 MHz, $CDCl_3$)

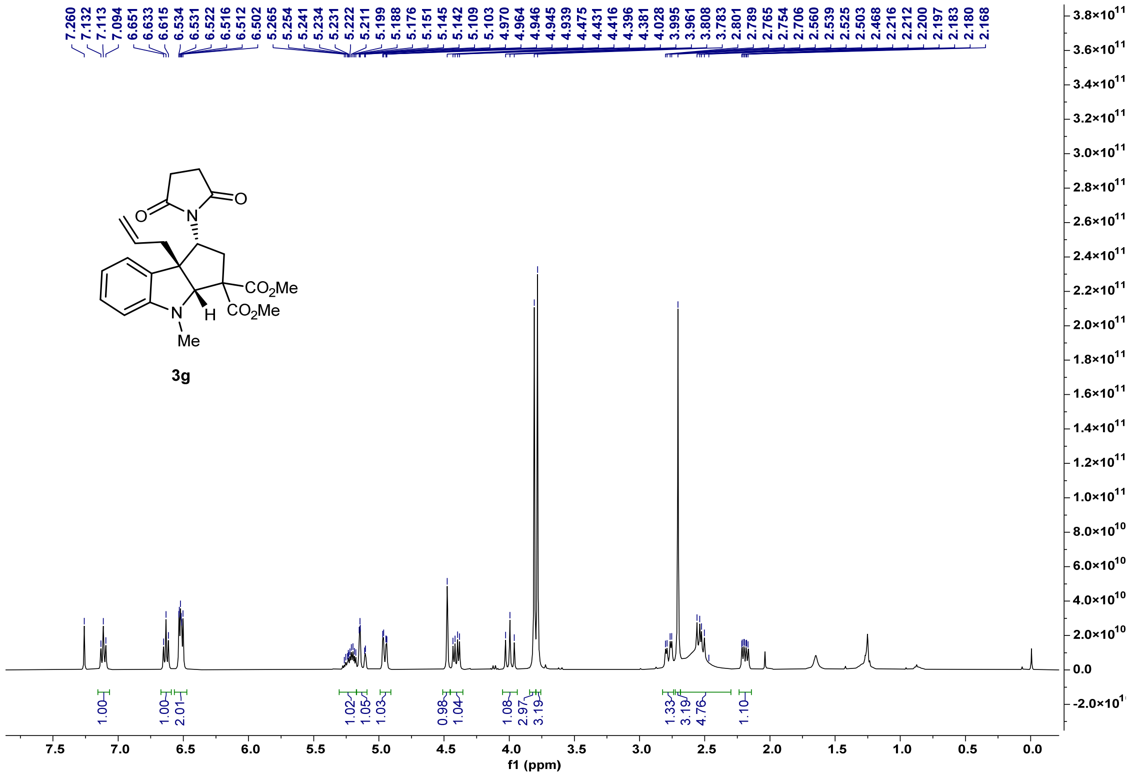


Supplementary Fig. 78 $^{13}C$ NMR(100 MHz, $CDCl_3$)

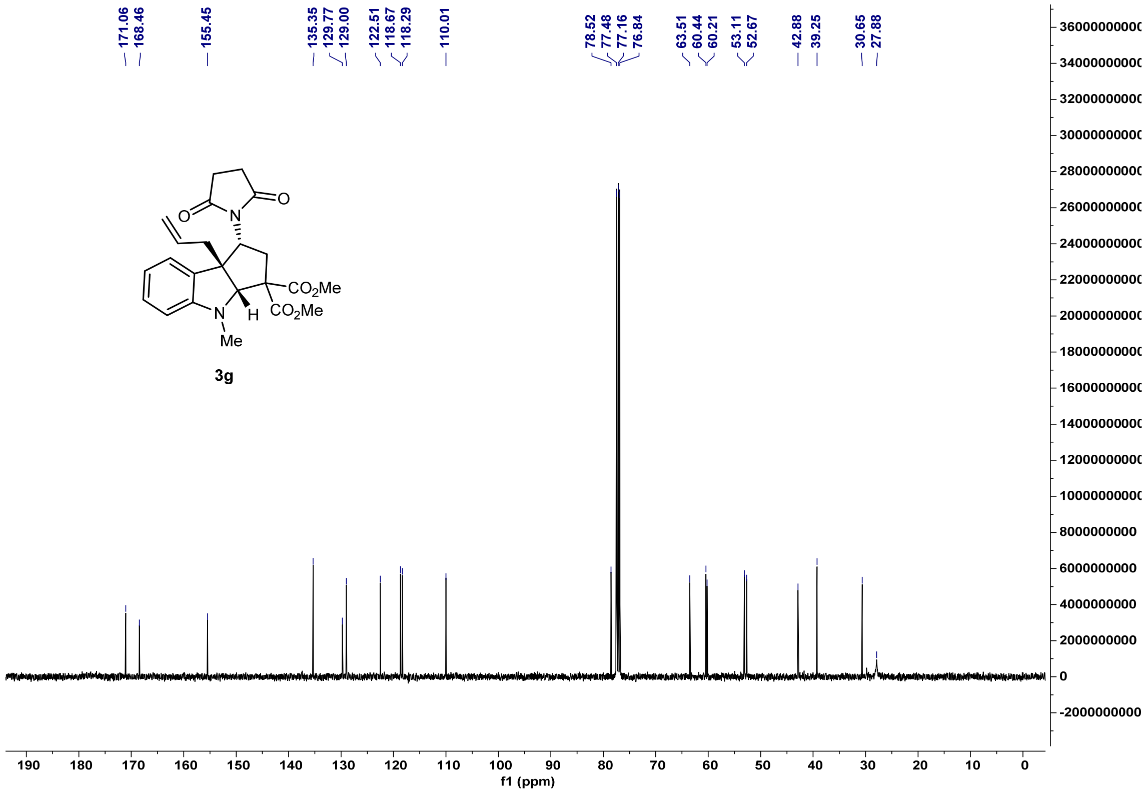

Supplementary Fig. 79 $^{1}$H NMR(600 MHz, $CDCl_3$)

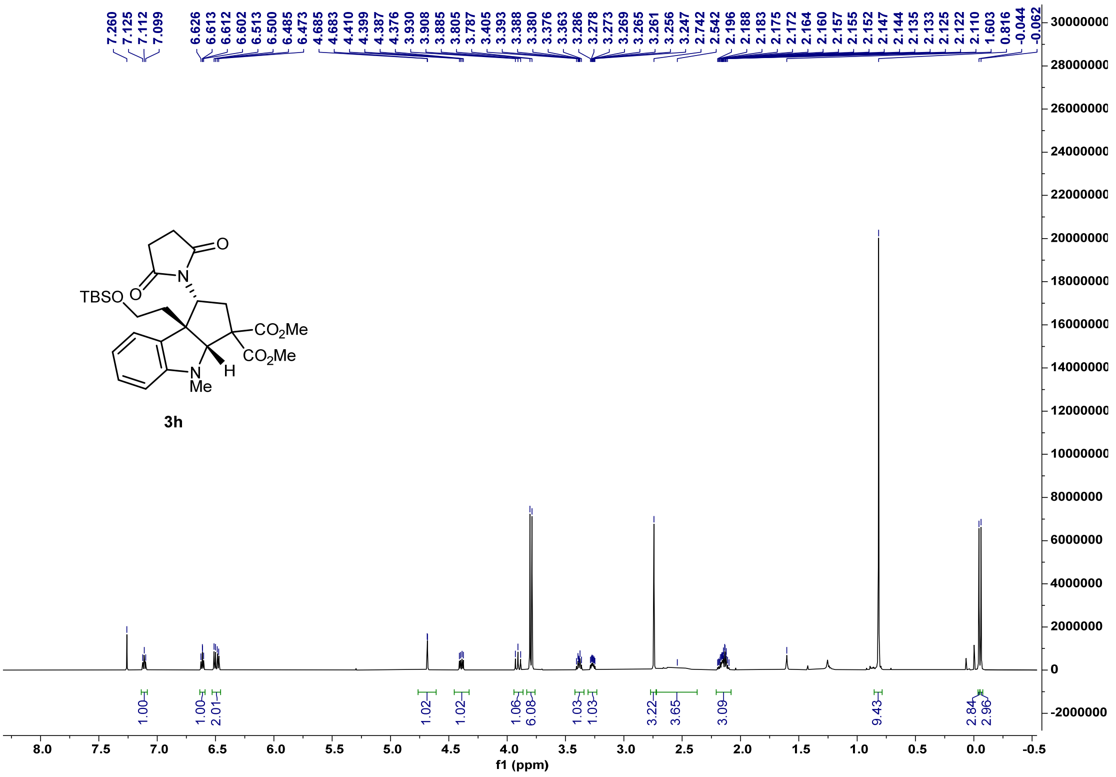


Supplementary Fig. 80 $^{13}$C NMR(150 MHz, $CDCl_3$)

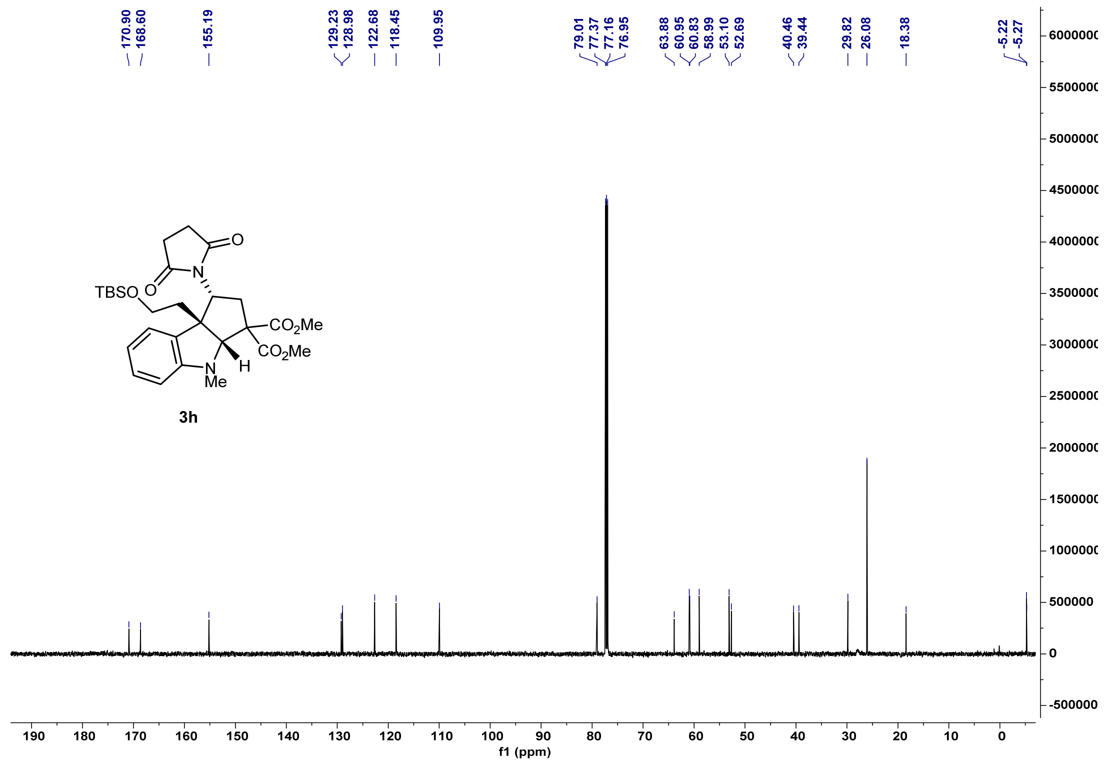

Supplementary Fig. 81 $^{1}H$ NMR(400 MHz, $CDCl_3$)

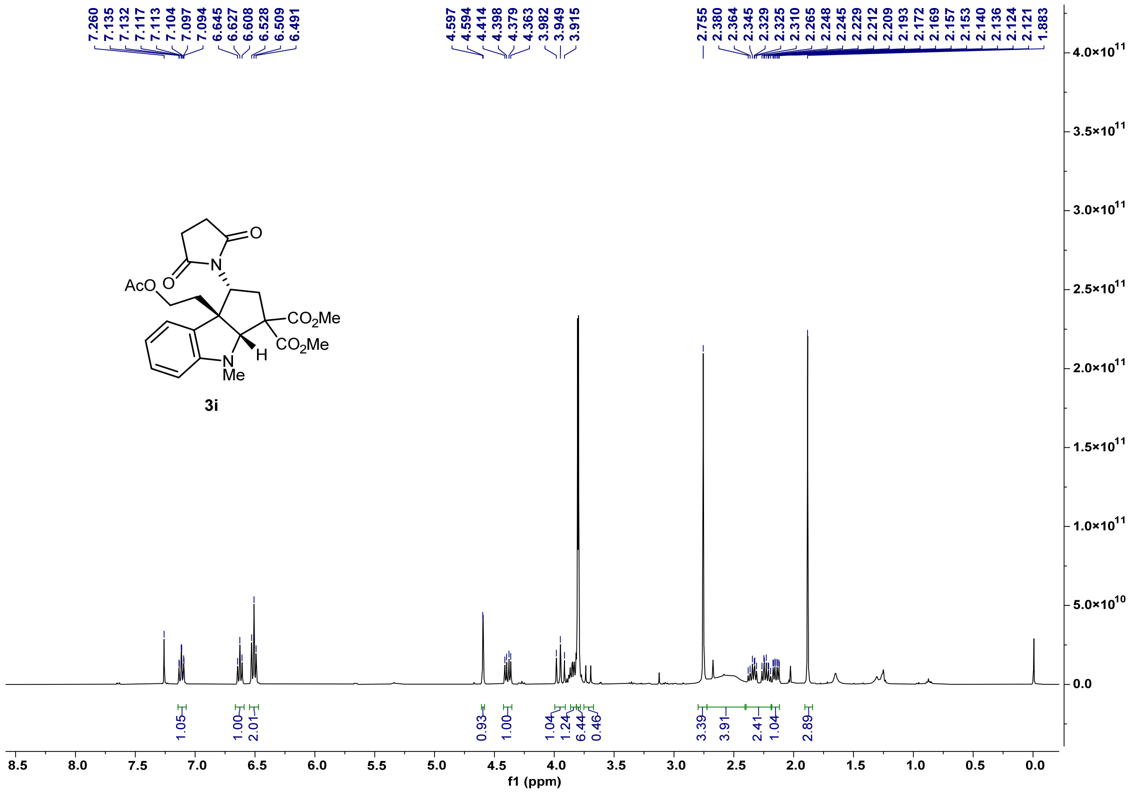


Supplementary Fig. 82 $^{13}C$ NMR(100 MHz, $CDCl_3$)

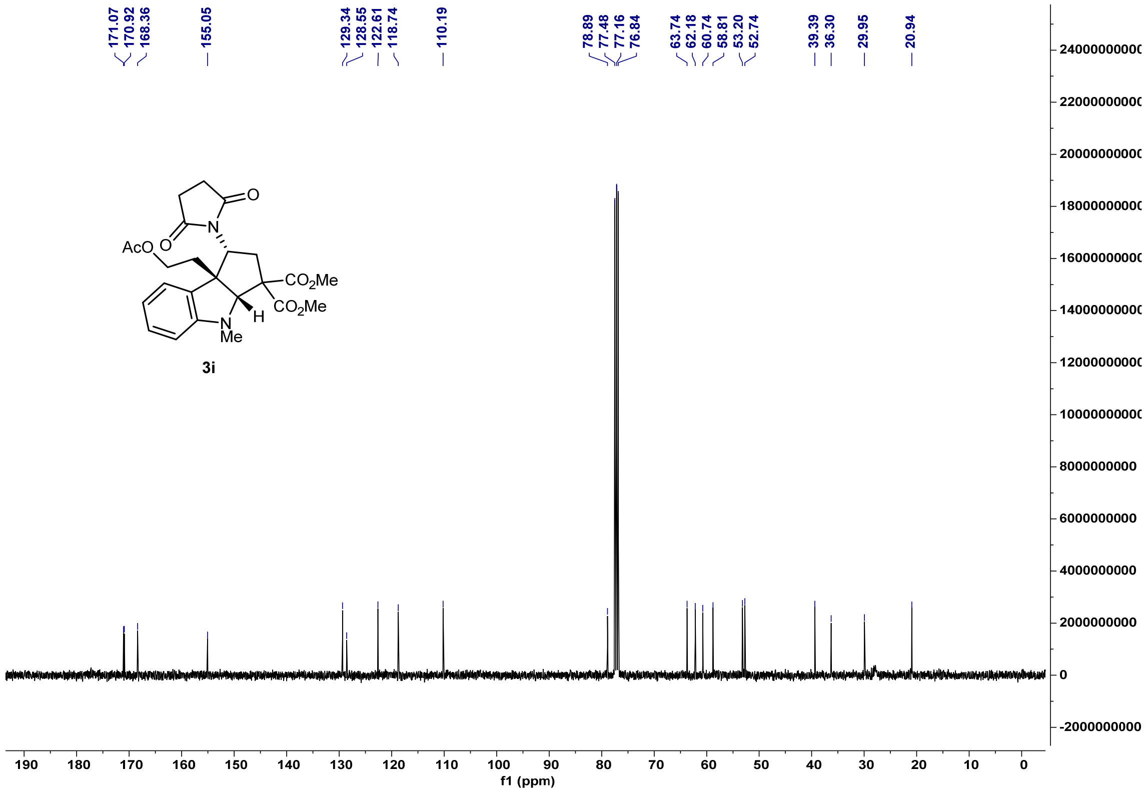

Supplementary Fig. 83 $^{1}$H NMR(400 MHz, $CDCl_3$)

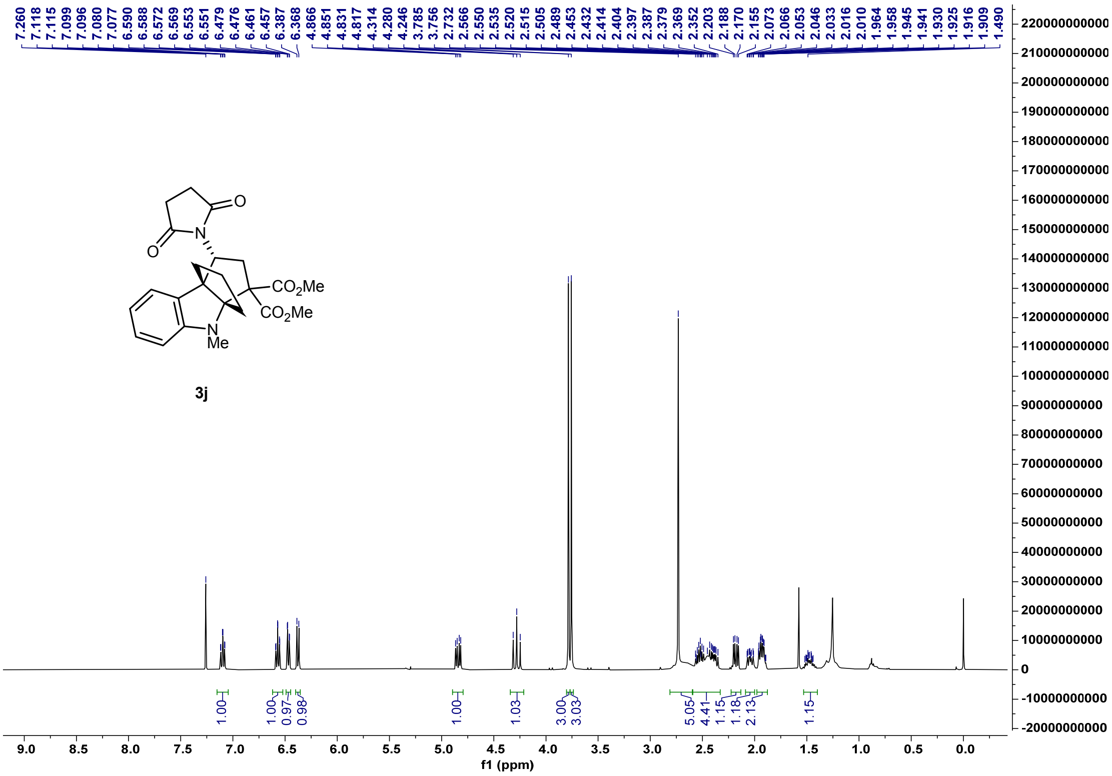

Supplementary Fig. 84 $^{13}$C NMR(100 MHz, $CDCl_3$)

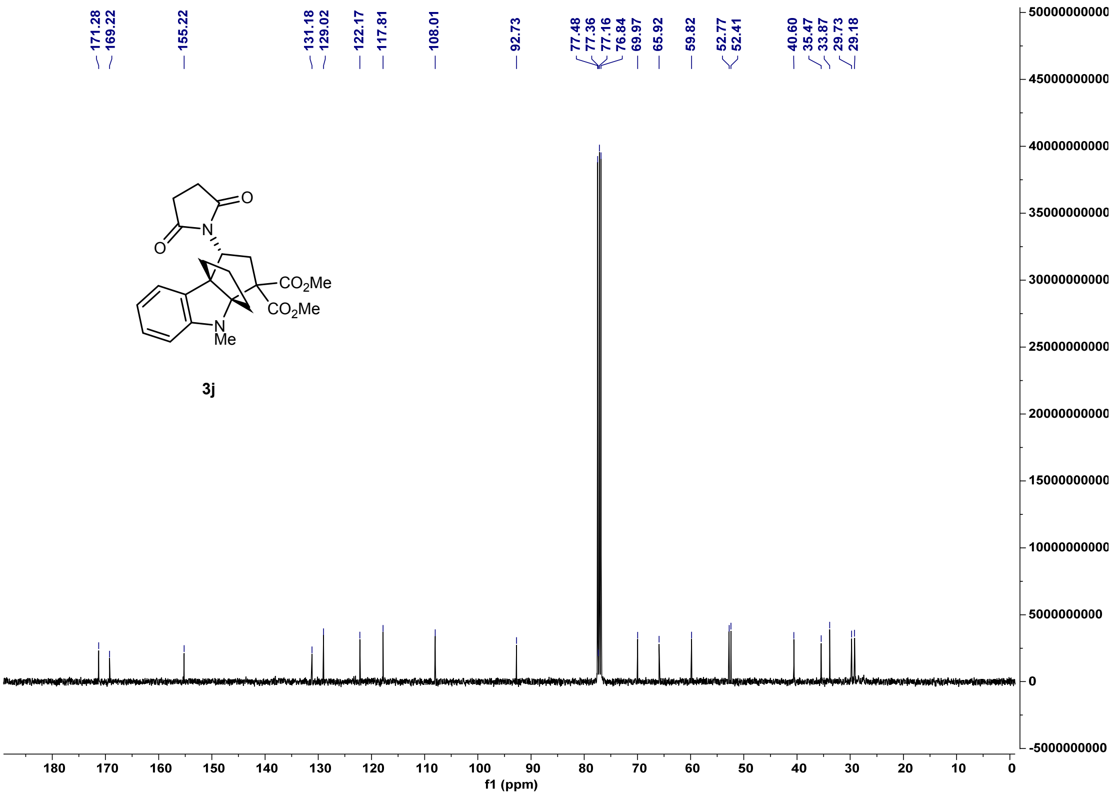

Supplementary Fig. 85 $^1$H NMR(600 MHz, $CDCl_3$)

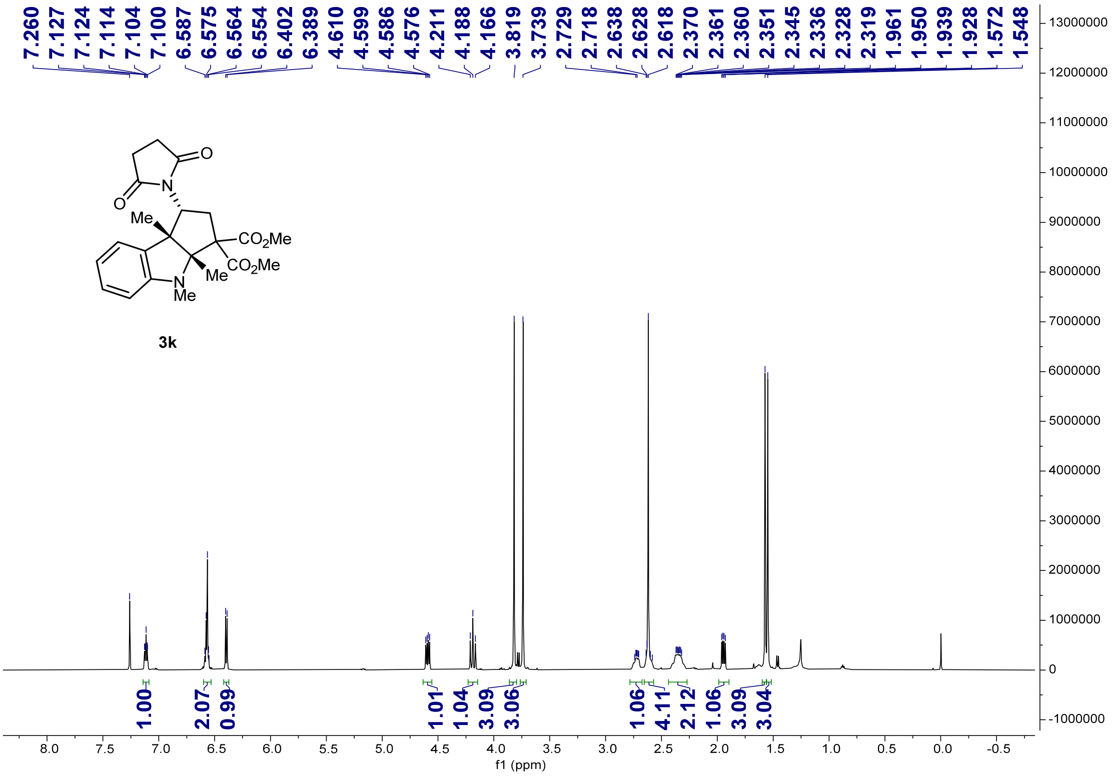


Supplementary Fig. 86 $^{13}$C NMR(100 MHz, $CDCl_3$)

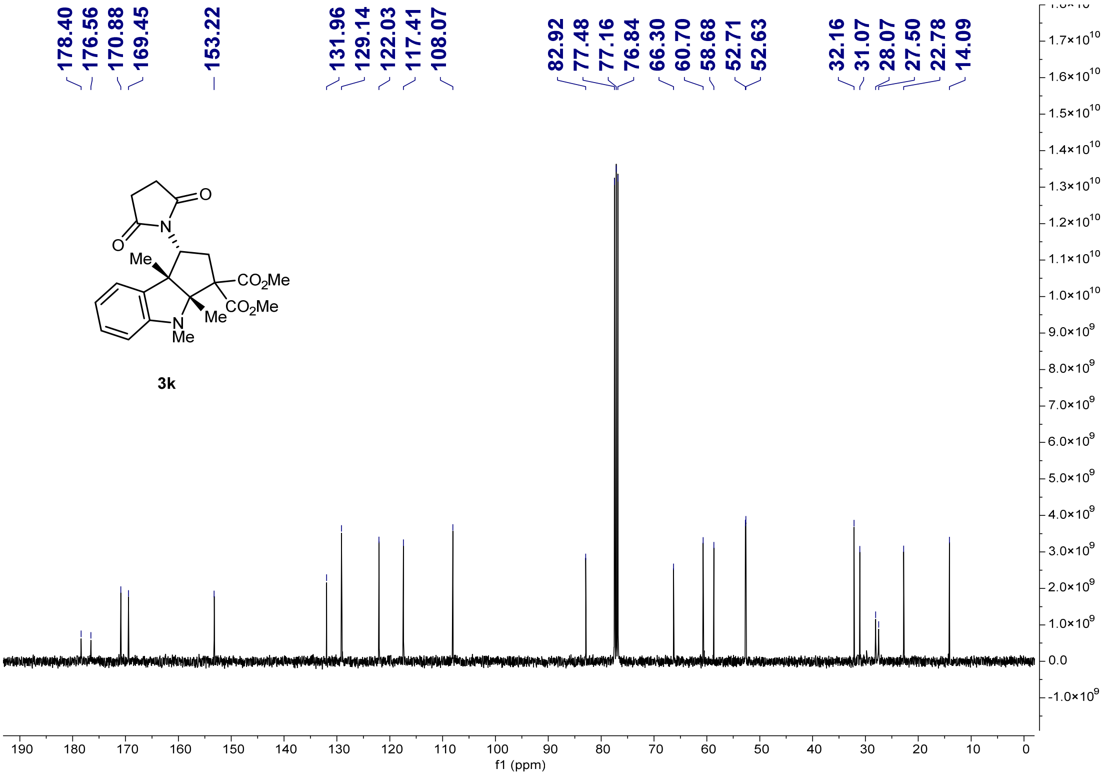

Supplementary Fig. 87 $^1$H NMR(400 MHz, $CDCl_3$)

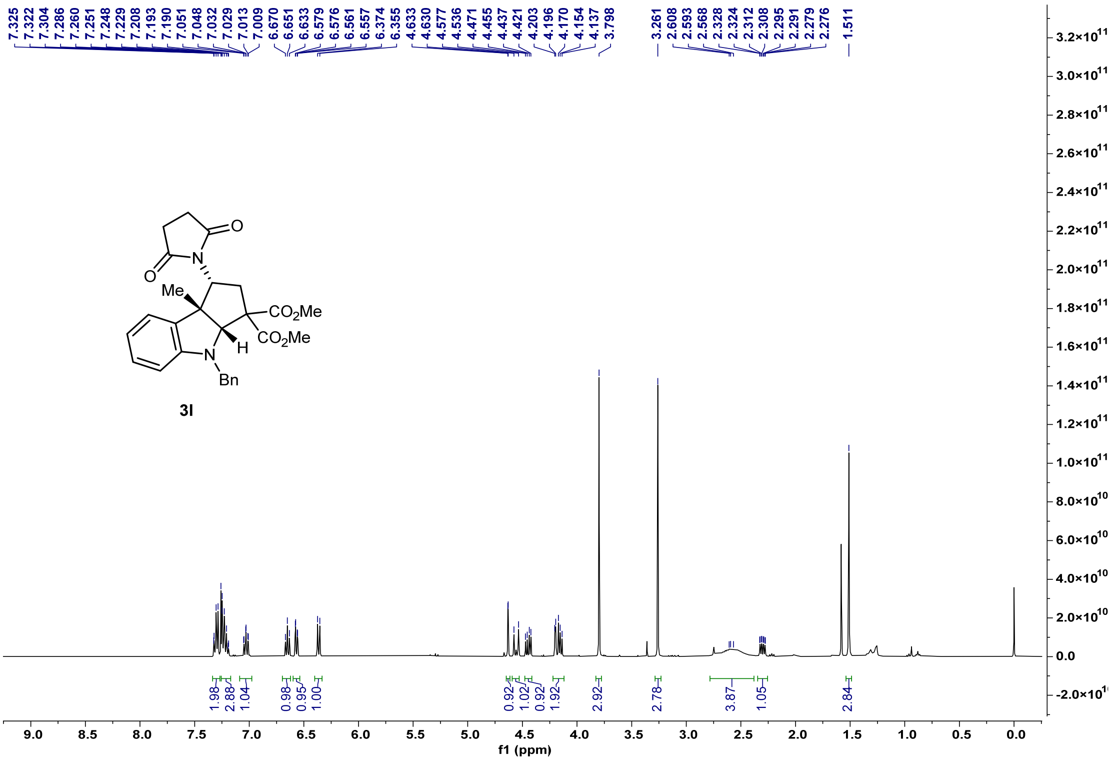


Supplementary Fig. 88 $^{13}$C NMR(100 MHz, $CDCl_3$)

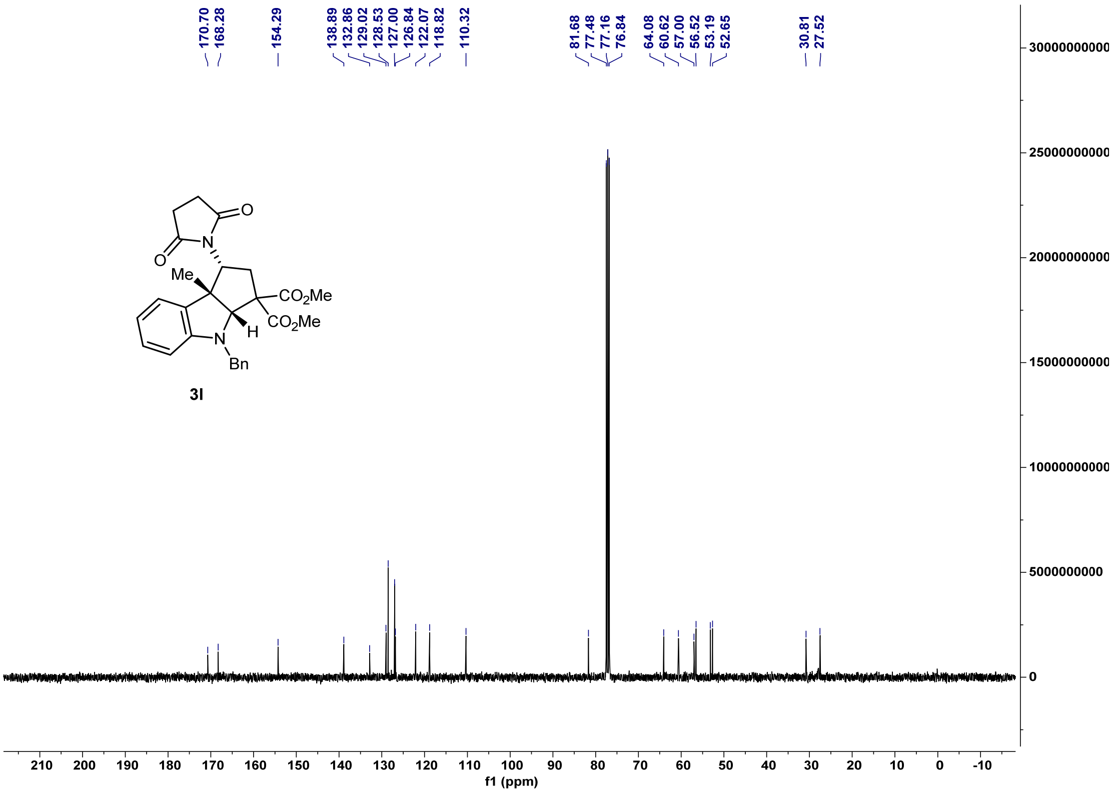

Supplementary Fig. 89 $^{1}$H NMR(400 MHz, $CDCl_3$)

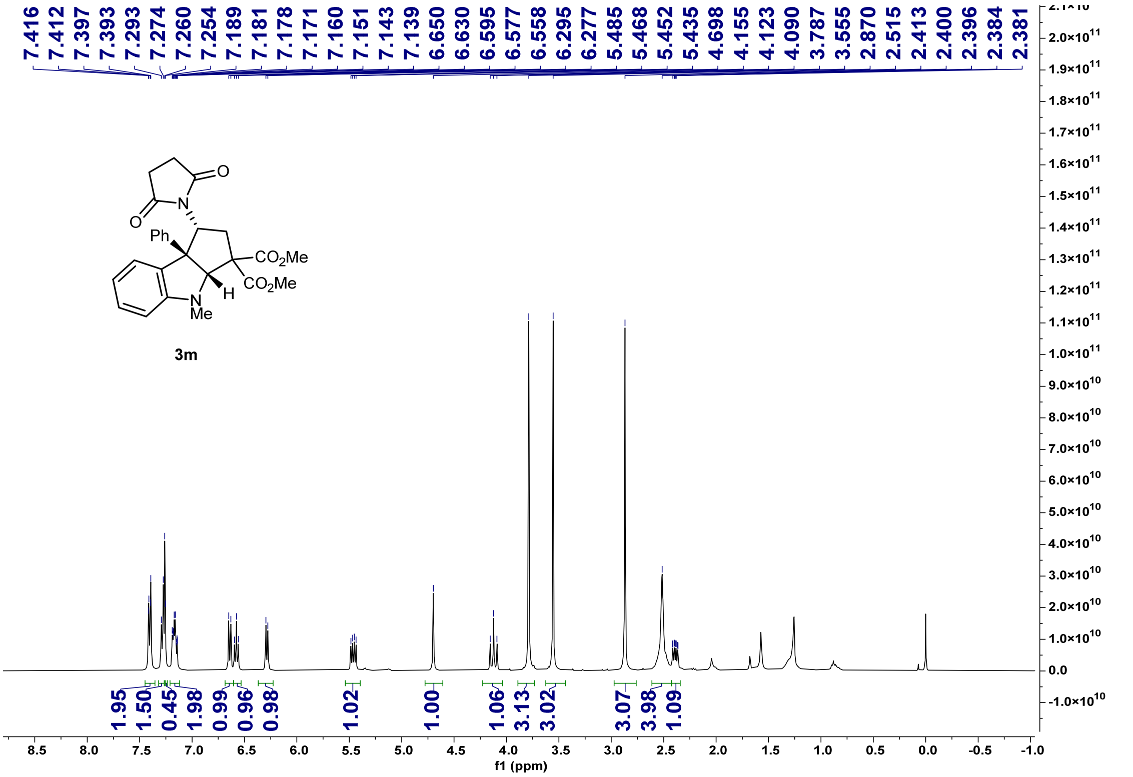


Supplementary Fig. 90 $^{13}$C NMR(100 MHz, $CDCl_3$)

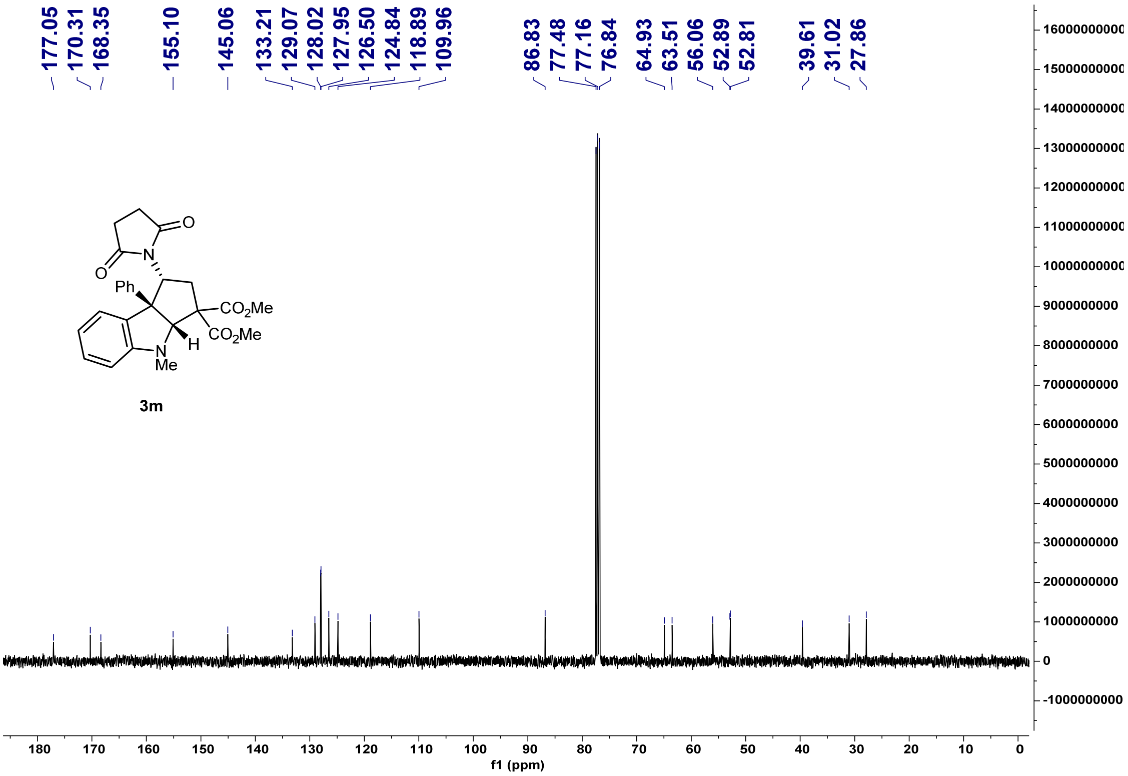

Supplementary Fig. 91 $^1H$ NMR(400 MHz, $CDCl_3$)

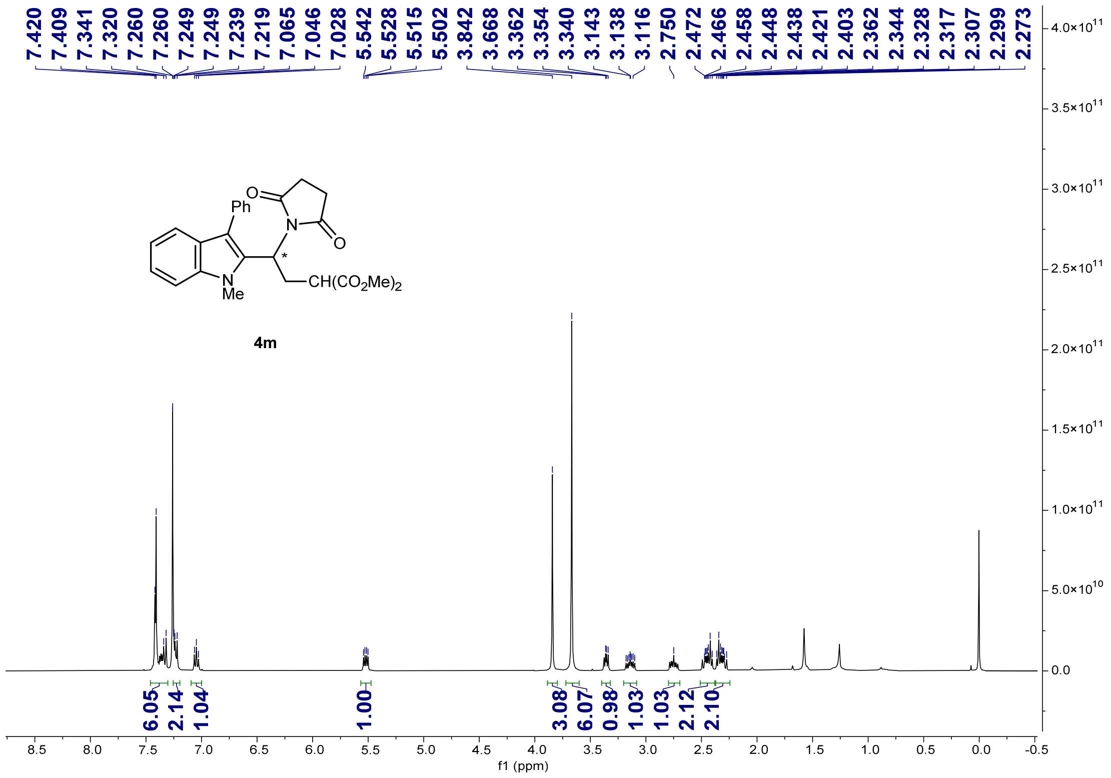


Supplementary Fig. 92 $^{13}C$ NMR(100 MHz, $CDCl_3$)

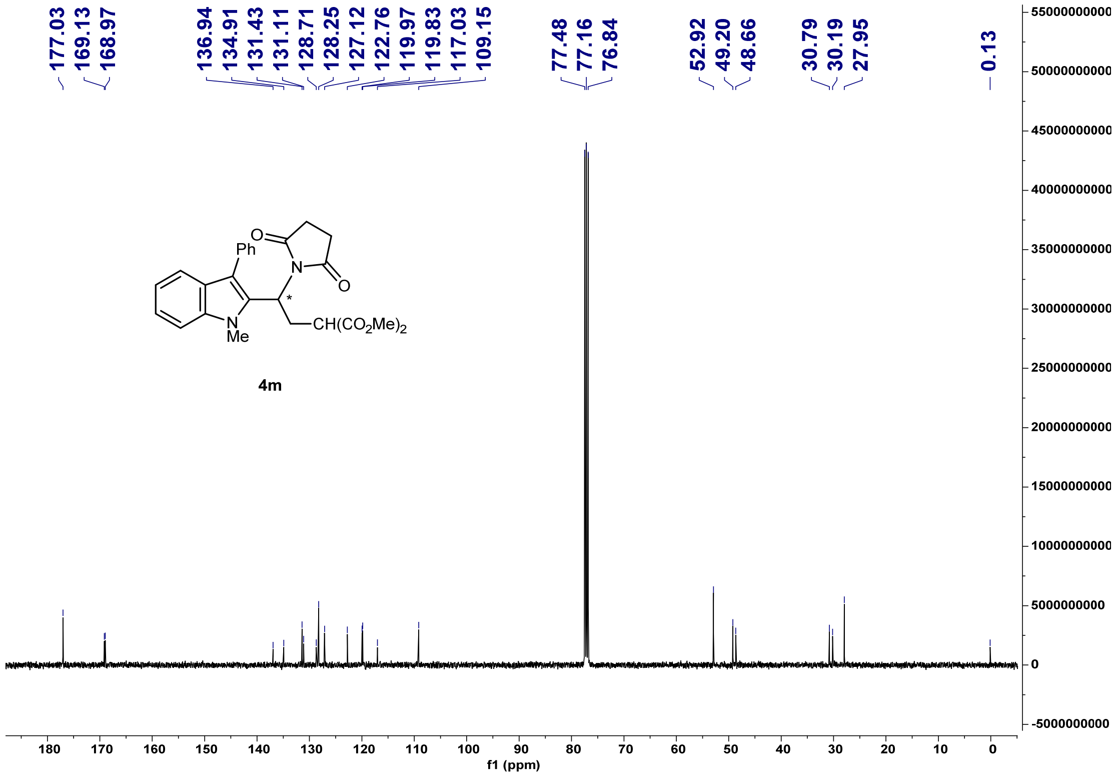

Supplementary Fig. 93 $^{1}H$ NMR(400 MHz, $CDCl_3$)

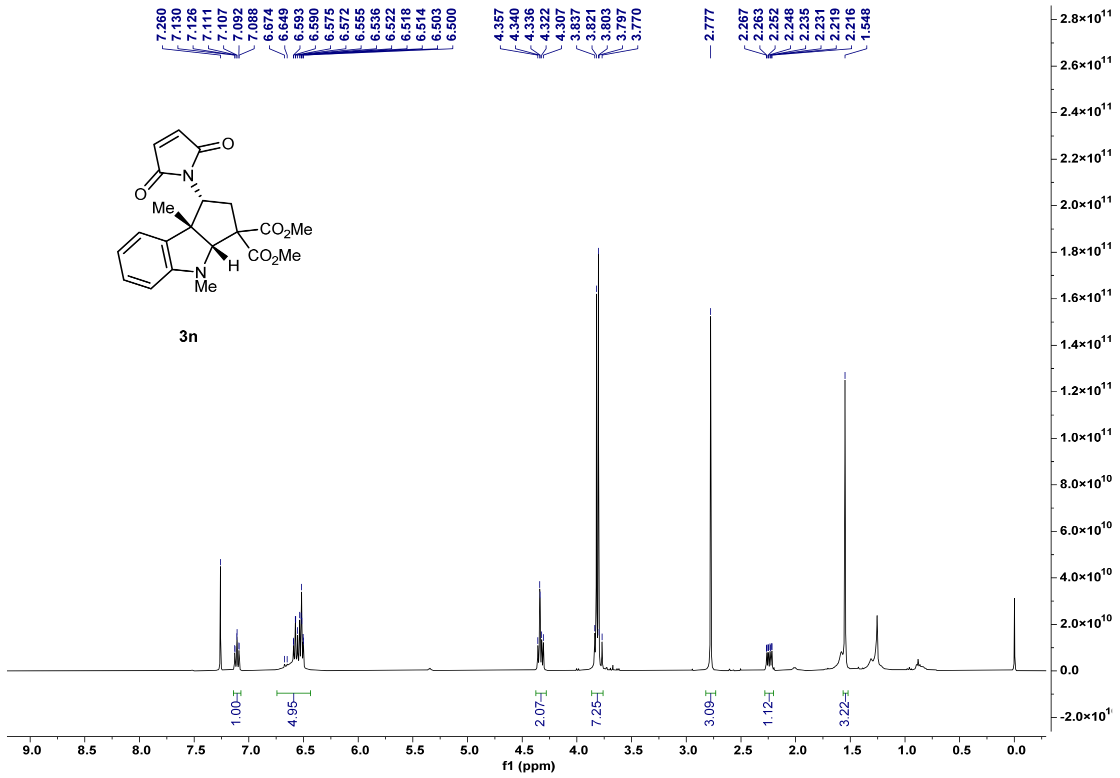


Supplementary Fig. 94 $^{13}C$ NMR(150 MHz, $CDCl_3$)

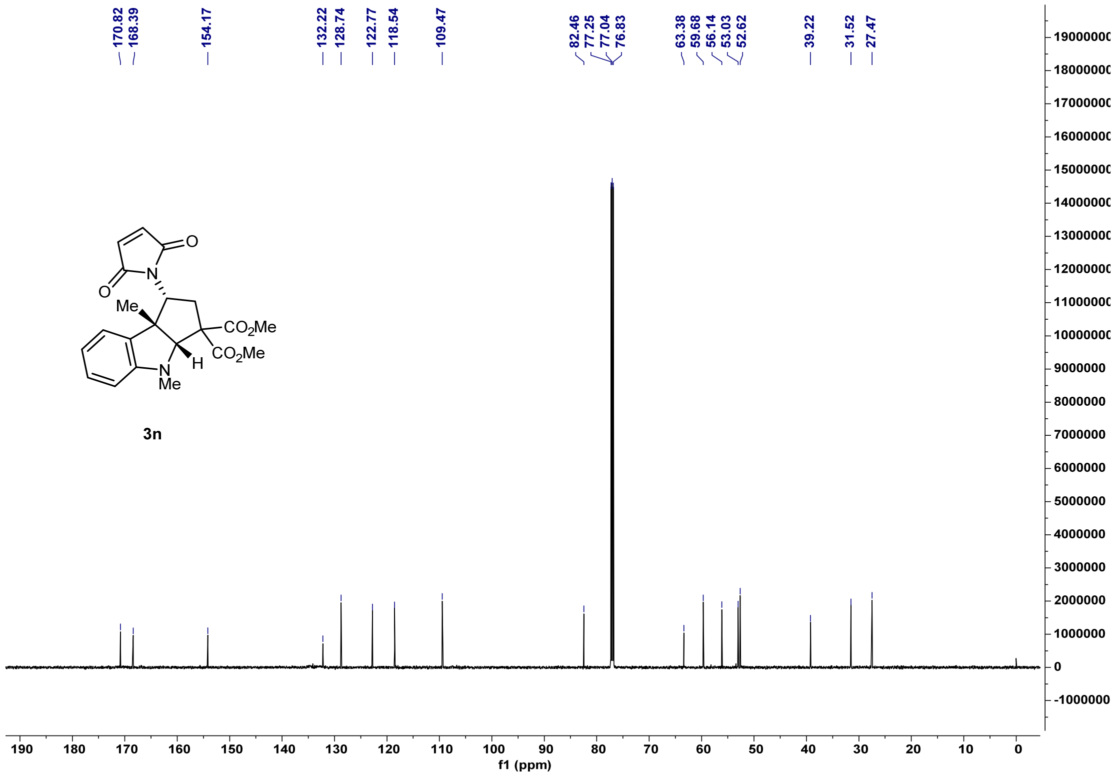

Supplementary Fig. 95 $^{1}H$ NMR(400 MHz, $CDCl_3$)

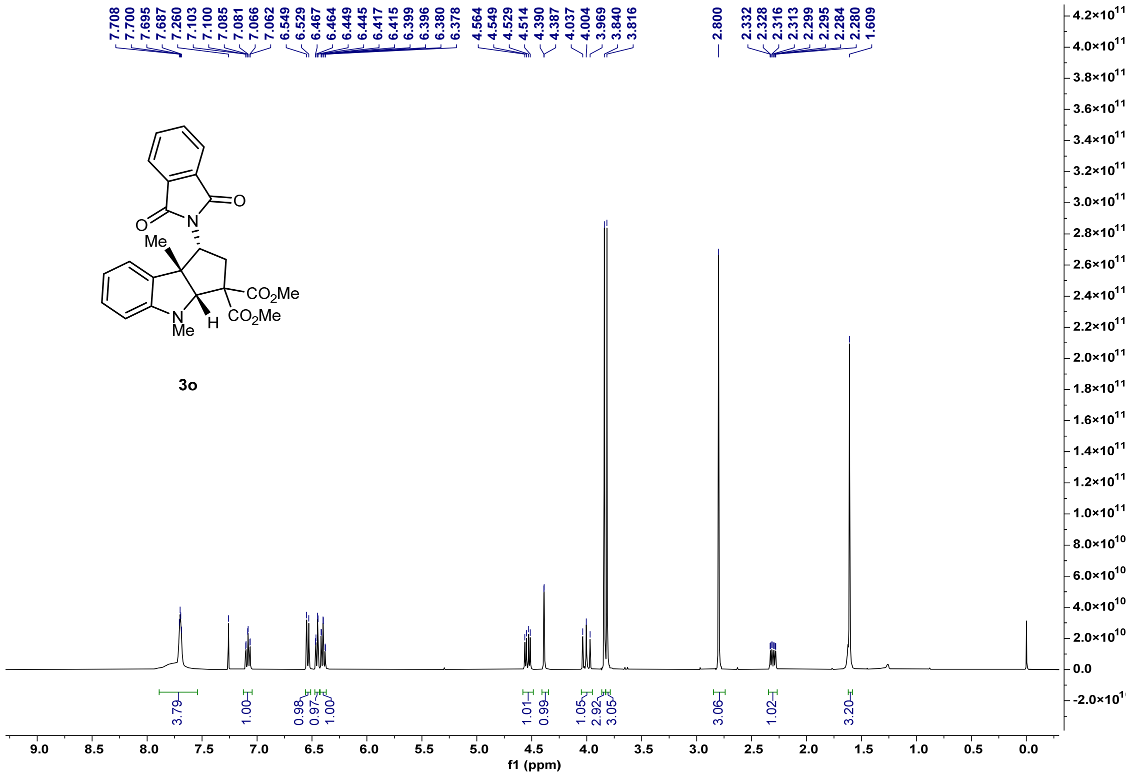


Supplementary Fig. 96 $^{13}C$ NMR(100 MHz, $CDCl_3$)

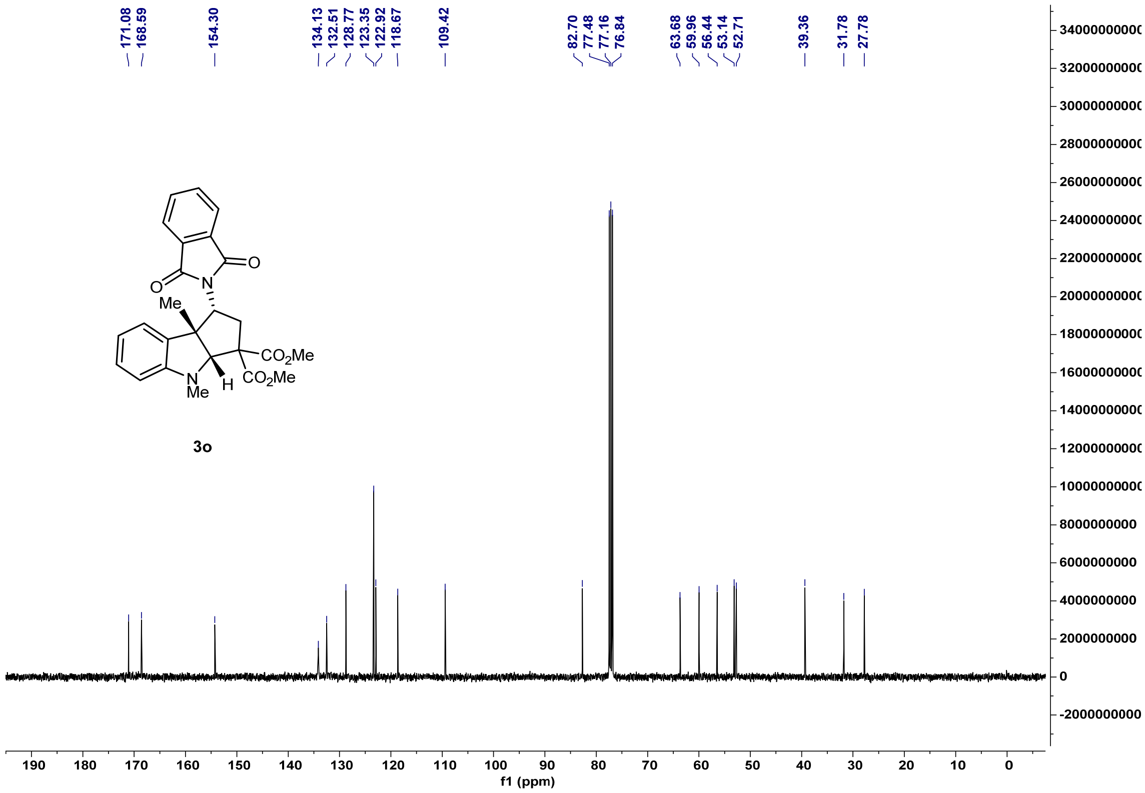

Supplementary Fig. 97 $^{1}$H NMR(400 MHz, $CDCl_3$)

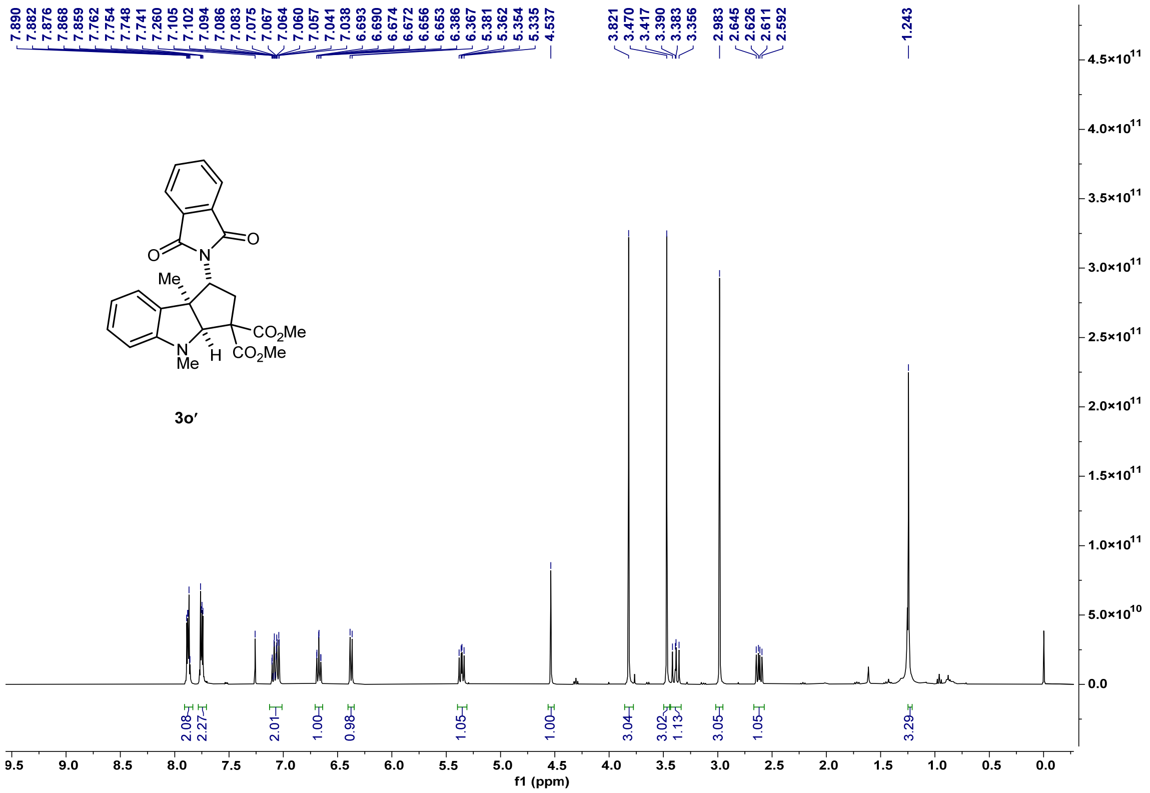

3o′

Supplementary Fig.98 $^{13}$C NMR(100 MHz, $CDCl_3$)

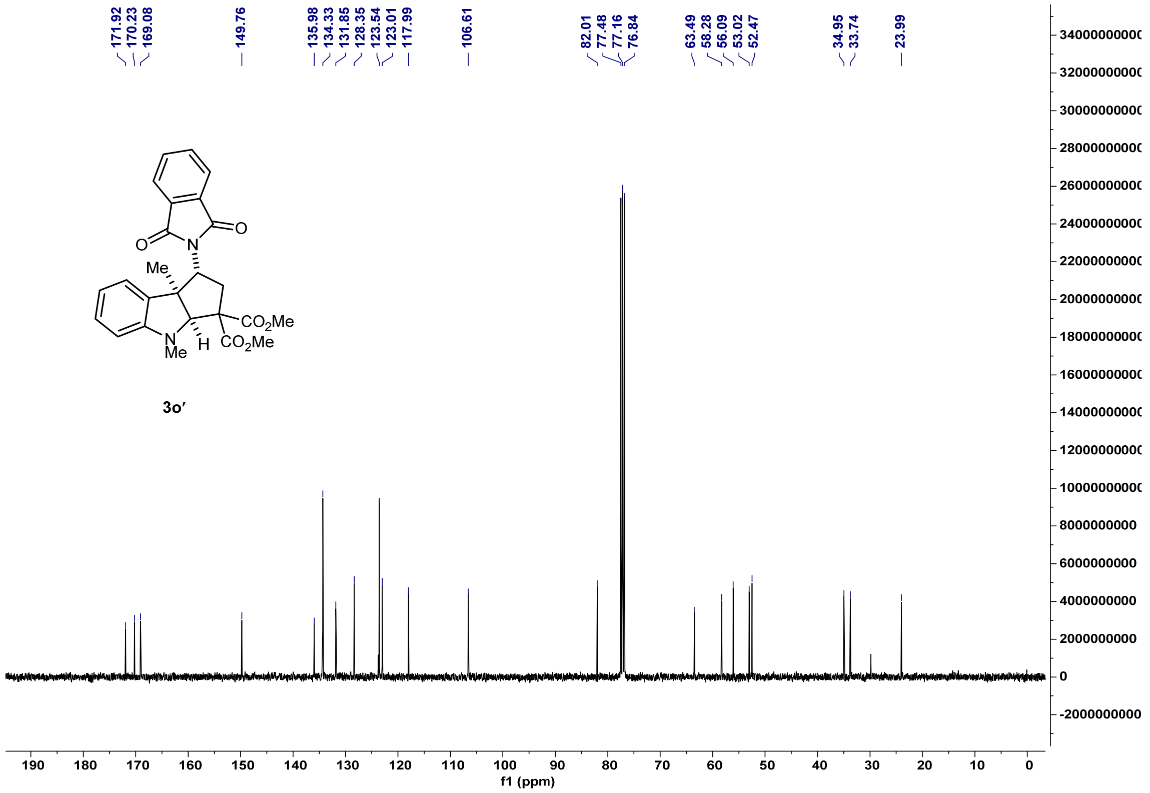

3o′

Supplementary Fig. 99 $^{1}H$ NMR(400 MHz, $CDCl_3$)

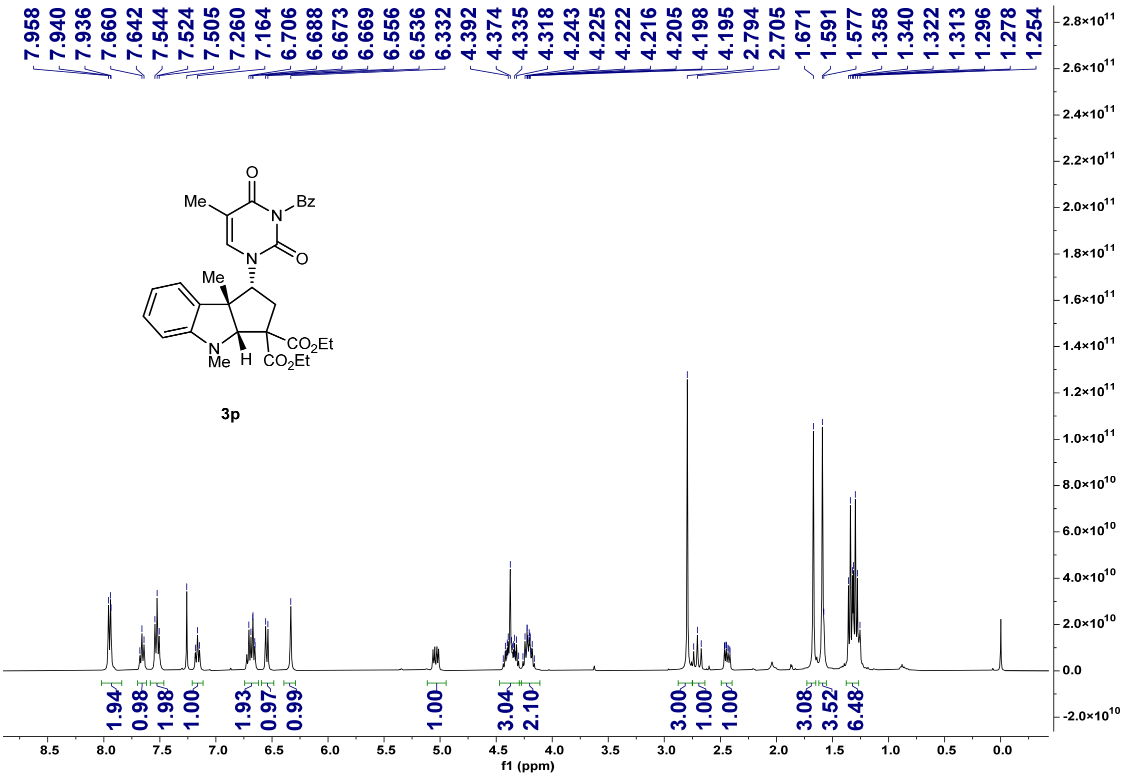


Supplementary Fig. 100 $^{13}C$ NMR(100 MHz, $CDCl_3$)

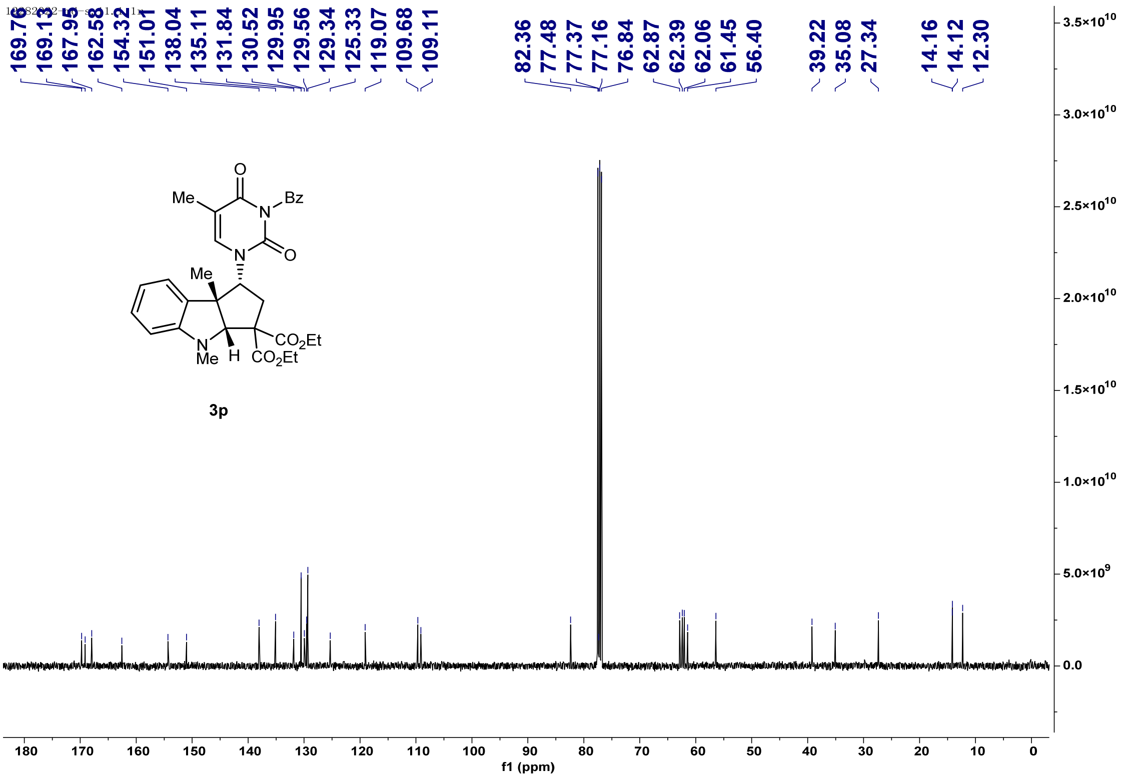

Supplementary Fig. 101 $^{1}H$ NMR(400 MHz, $CDCl_3$)

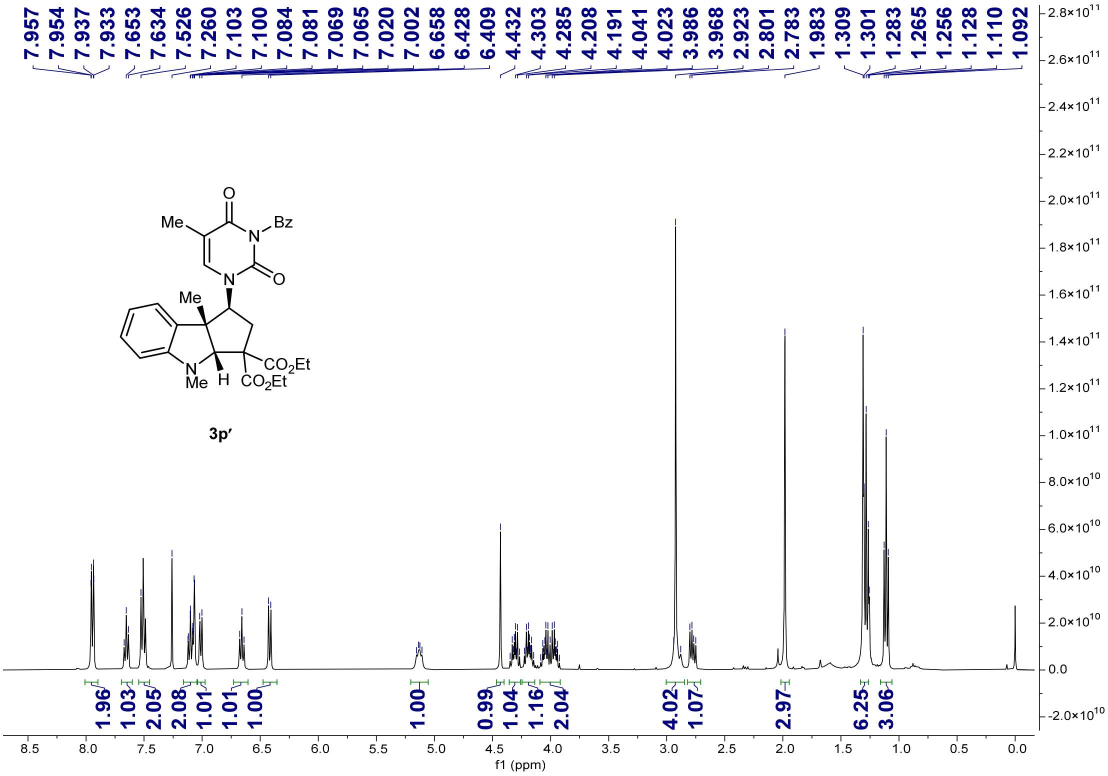


Supplementary Fig. 102 $^{13}C$ NMR(100 MHz, $CDCl_3$)

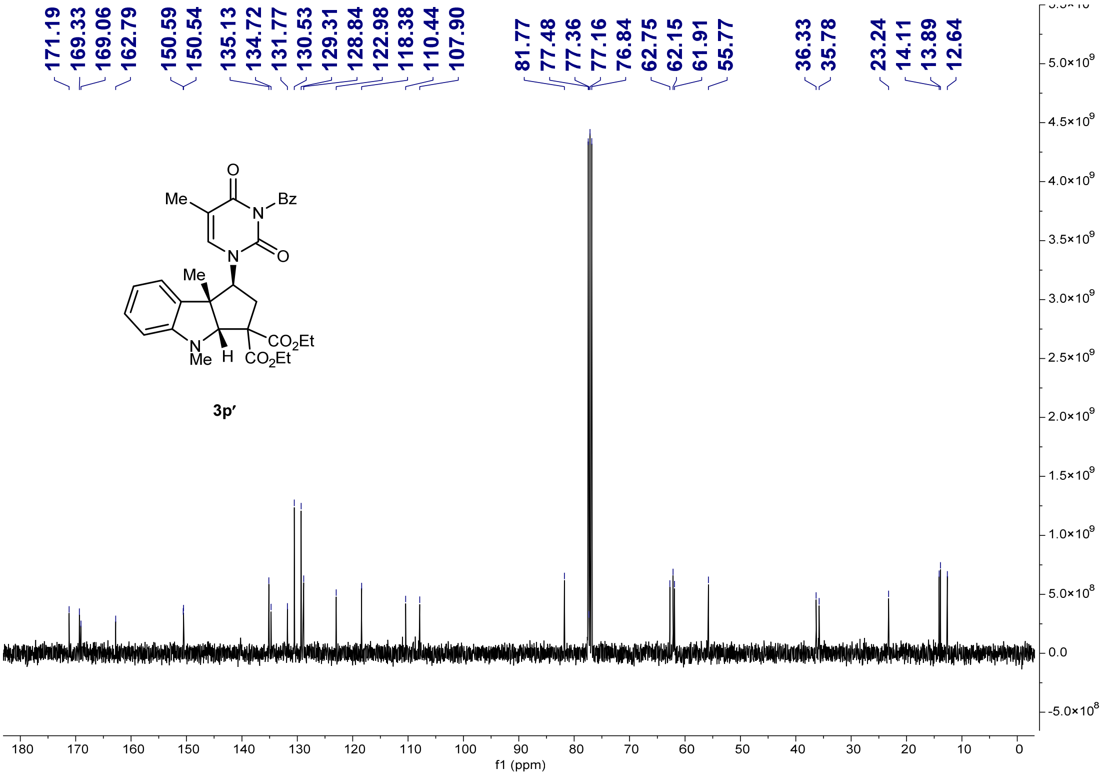

Supplementary Fig. 103 $^{1}H$ NMR(400 MHz, $CDCl_3$)

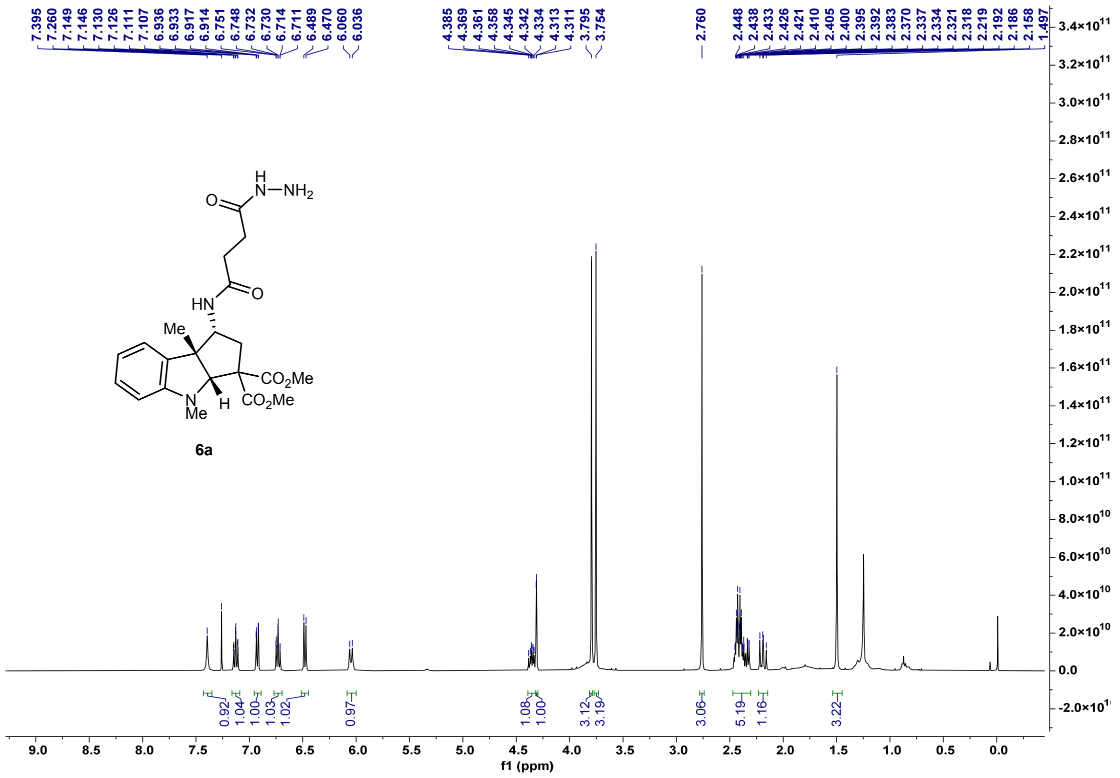


Supplementary Fig. 104 $^{13}C$ NMR(150 MHz, $CDCl_3$)

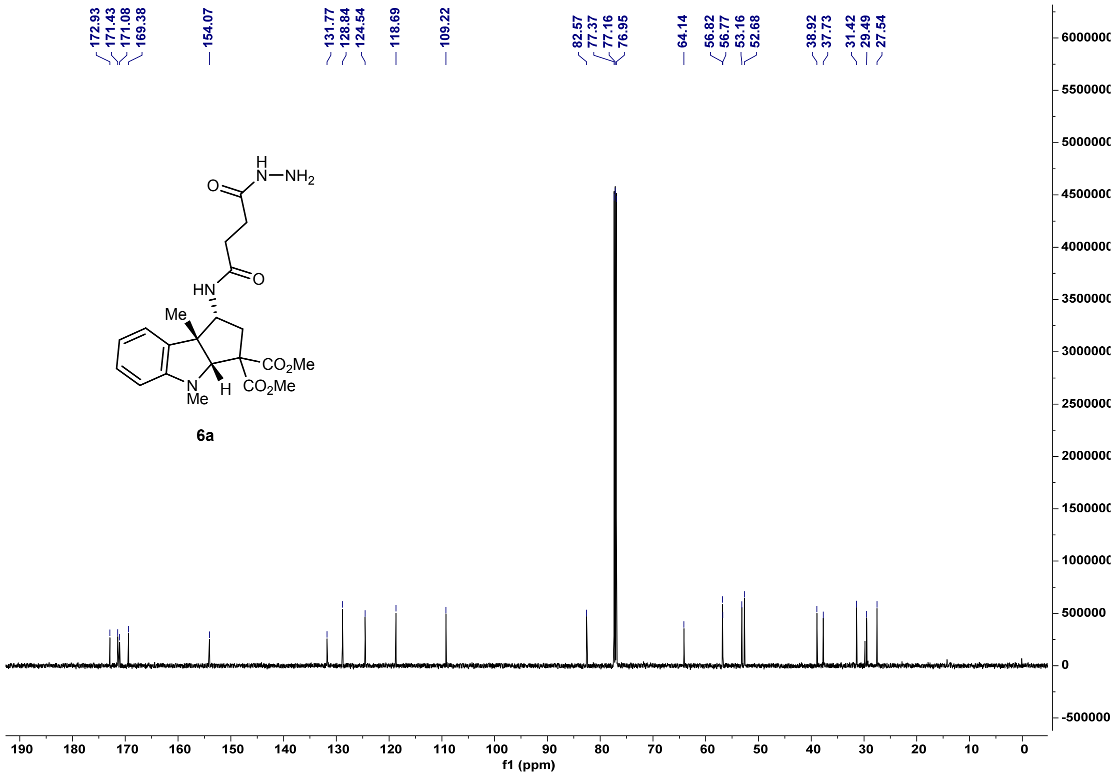

Supplementary Fig. 105 $^{1}$H NMR(400 MHz, $CDCl_3$)

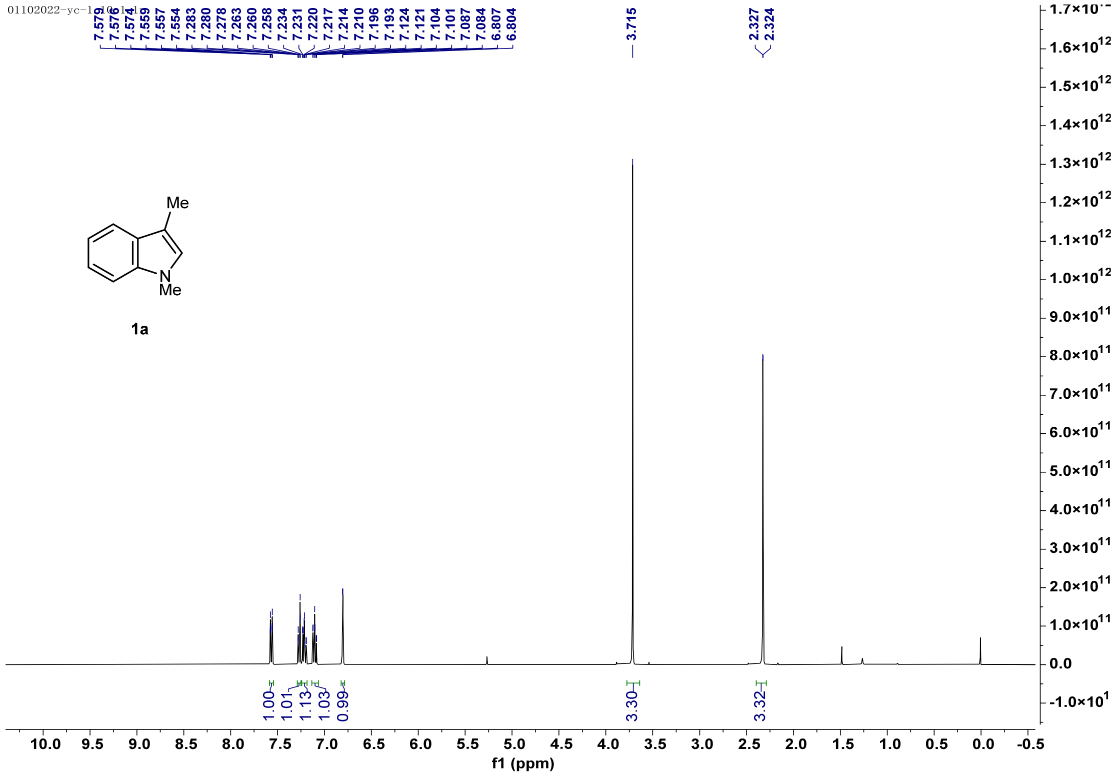


Supplementary Fig. 106 $^{13}$C NMR(100 MHz, $CDCl_3$)

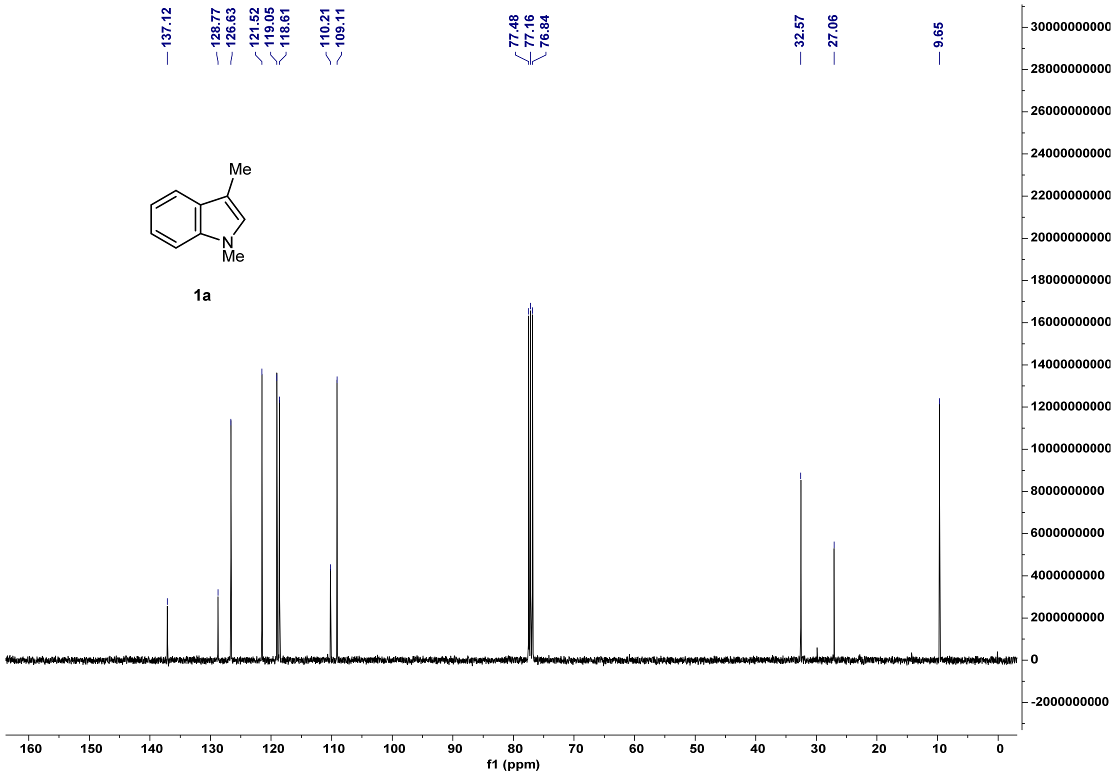

Supplementary Fig. 107 $^{1}H$ NMR(400 MHz, $CDCl_3$)

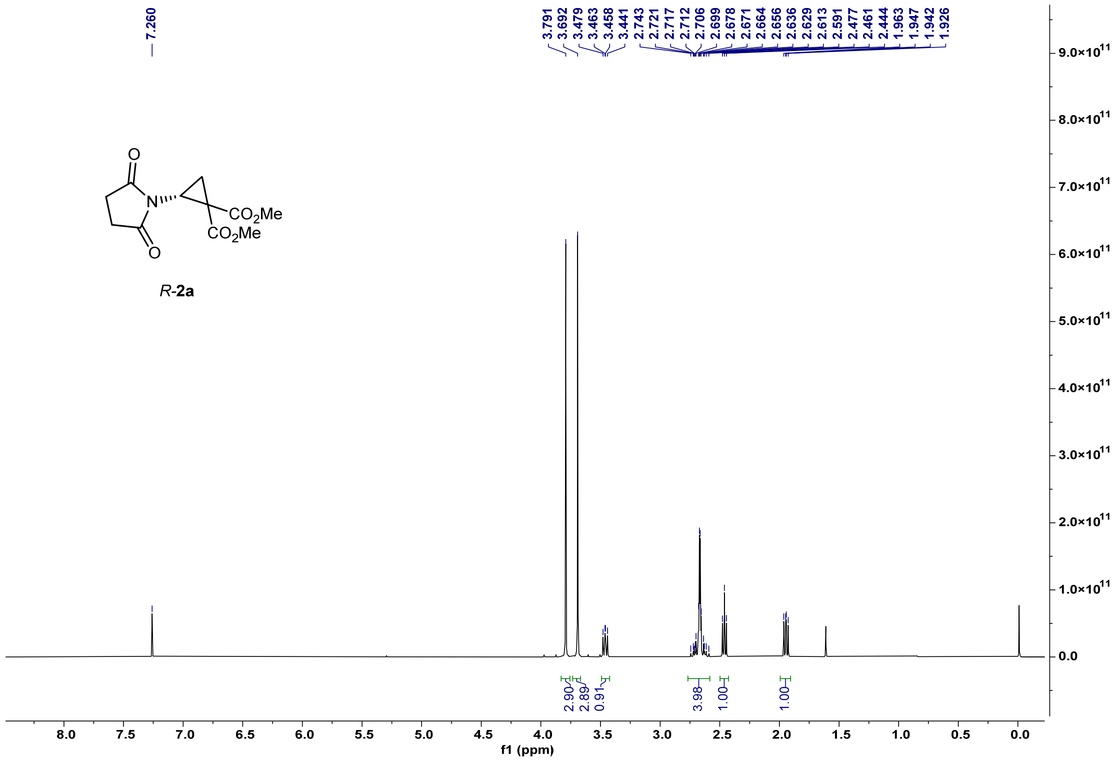


Supplementary Fig. 108 $^{13}C$ NMR(100 MHz, $CDCl_3$)

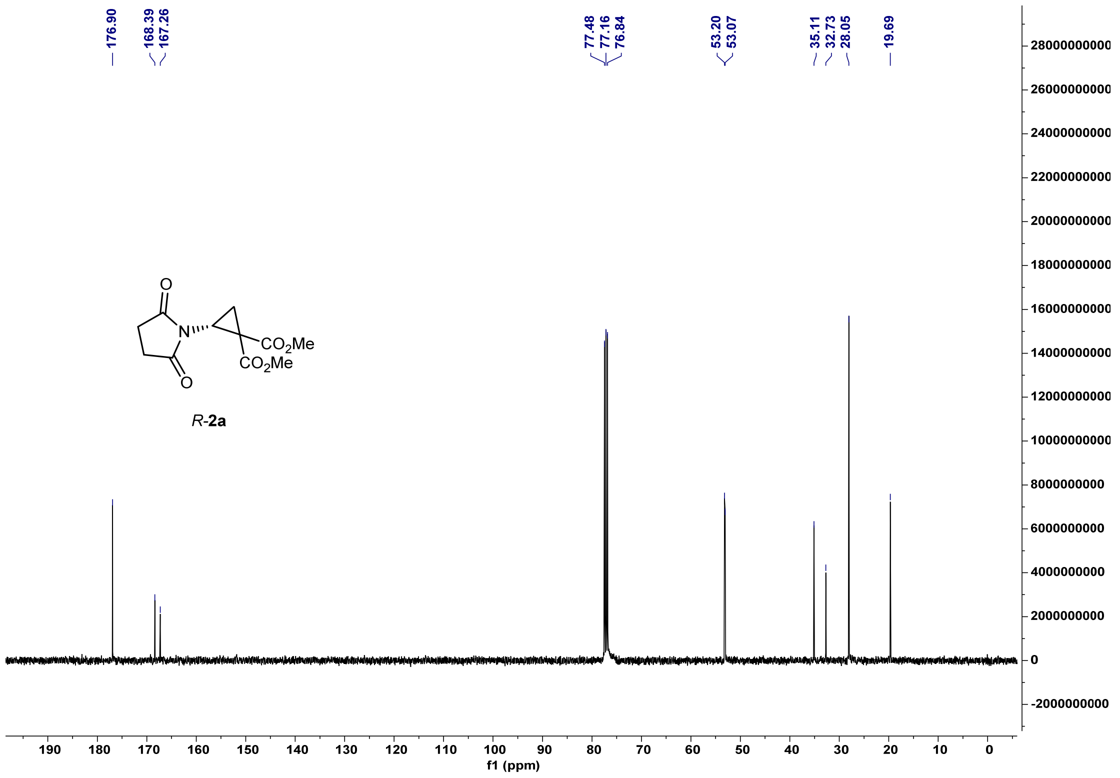

Supplementary Fig. 109 $^{1}H$ NMR(400 MHz, $CDCl_3$)

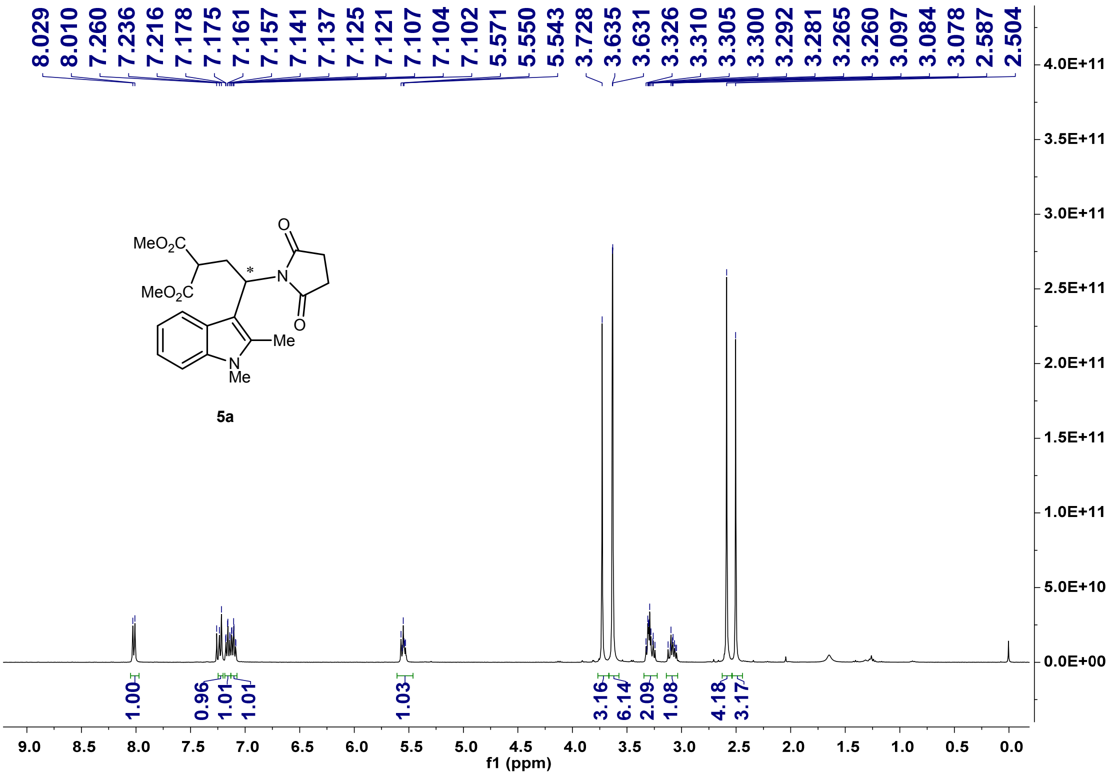


Supplementary Fig. 110 $^{13}C$ NMR(100 MHz, $CDCl_3$)

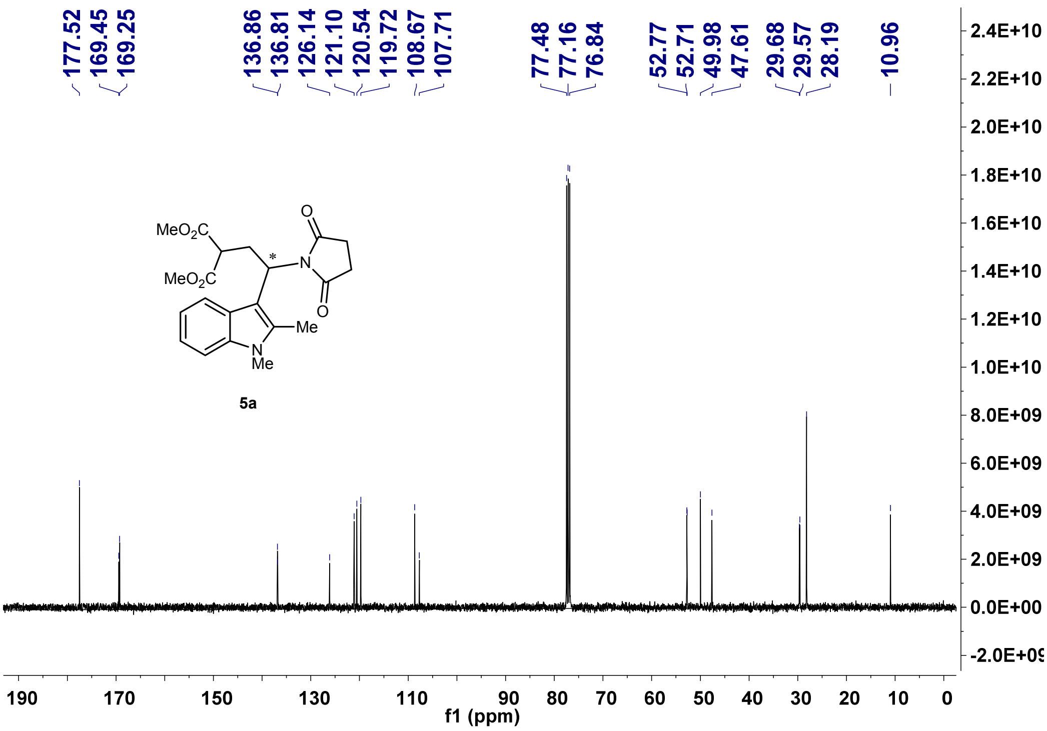

Supplementary Fig. 111 $^{1}$H NMR(400 MHz, $CDCl_3$)

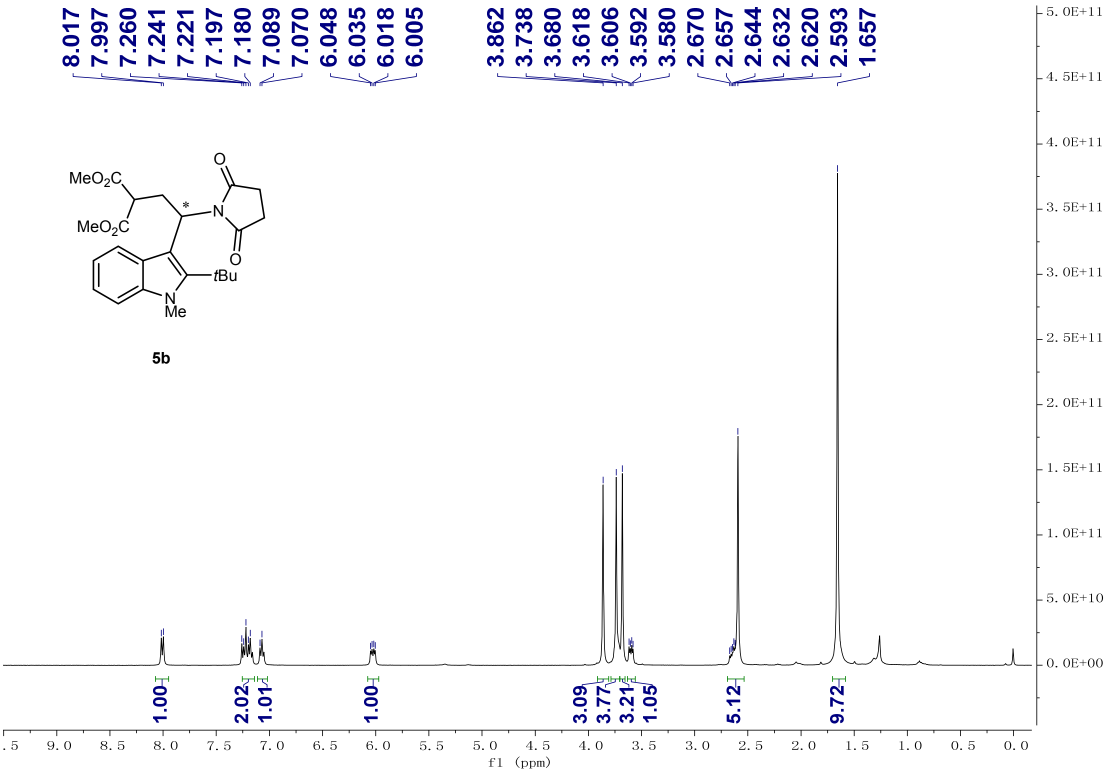


Supplementary Fig. 112 $^{13}$C NMR(100 MHz, $CDCl_3$)

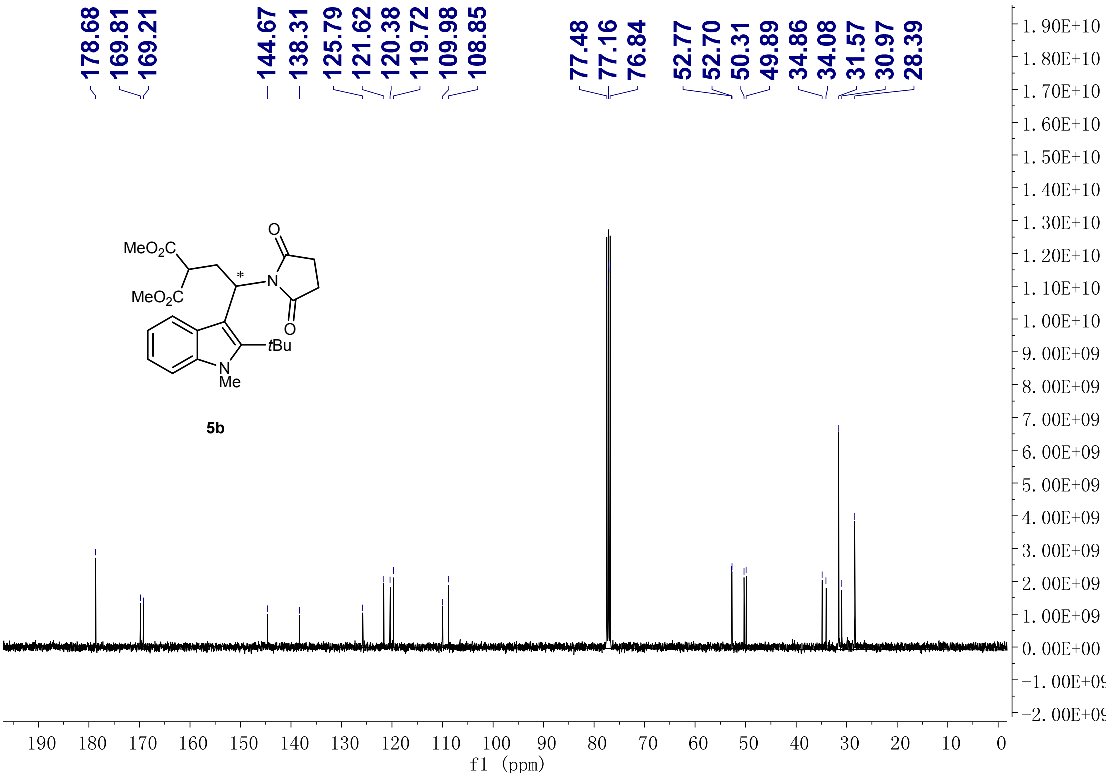

# HPLC Spectra of all new compounds

Supplementary Fig. 113 HPLC spectra of **3a**

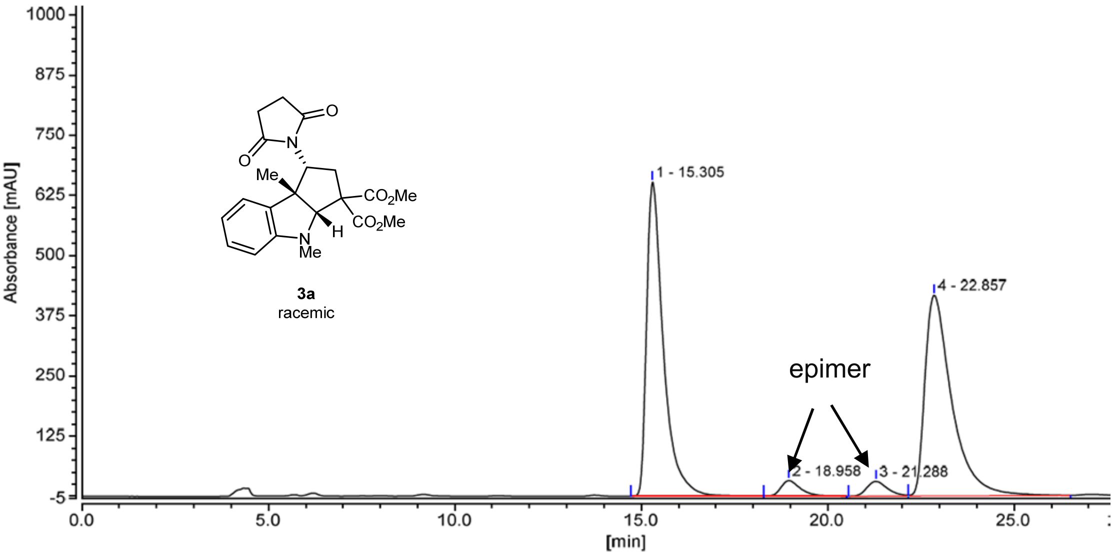


| Peak | Retention Time min | Area mAU*min | Height mAU | Area % | Height % |
|---|---|---|---|---|---|
| 1 | 15.305 | 341.327 | 652.988 | 46.91 | 57.63 |
| 2 | 18.958 | 19.633 | 32.148 | 2.70 | 2.84 |
| 3 | 21.288 | 19.320 | 30.412 | 2.65 | 2.68 |
| 4 | 22.857 | 347.417 | 417.486 | 47.74 | 36.85 |
| **Total:** | | **727.696** | **1133.033** | **100.00** | **100.00** |

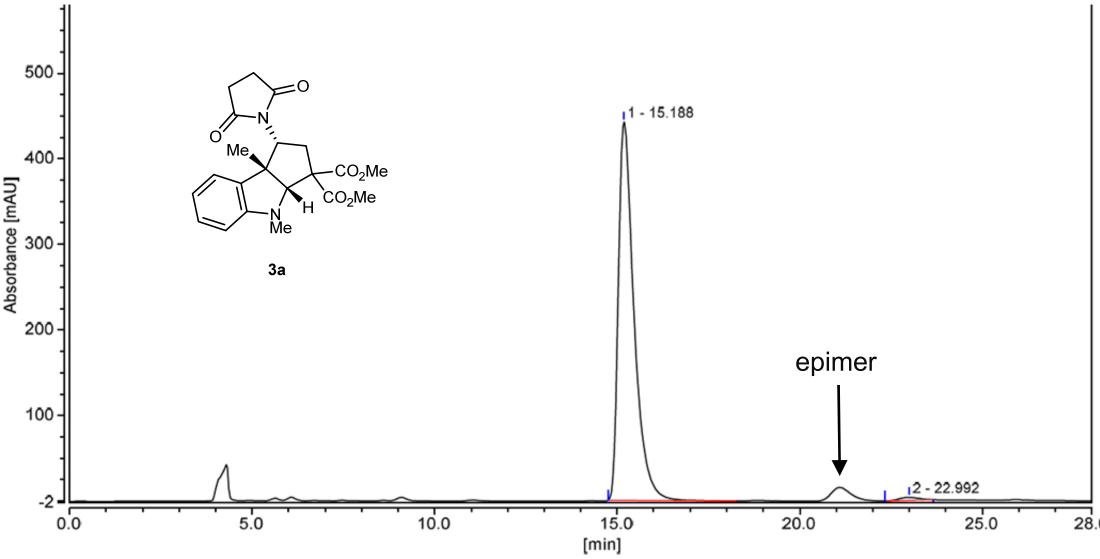


| Peak | Retention Time min | Area mAU*min | Height mAU | Area % | Height % |
|---|---|---|---|---|---|
| 1 | 15.188 | 219.510 | 441.998 | 99.03 | 99.22 |
| 2 | 22.992 | 2.157 | 3.489 | 0.97 | 0.78 |
| **Total:** | | **221.667** | **445.487** | **100.00** | **100.00** |

Supplementary Fig. 114 HPLC spectra of **3b**

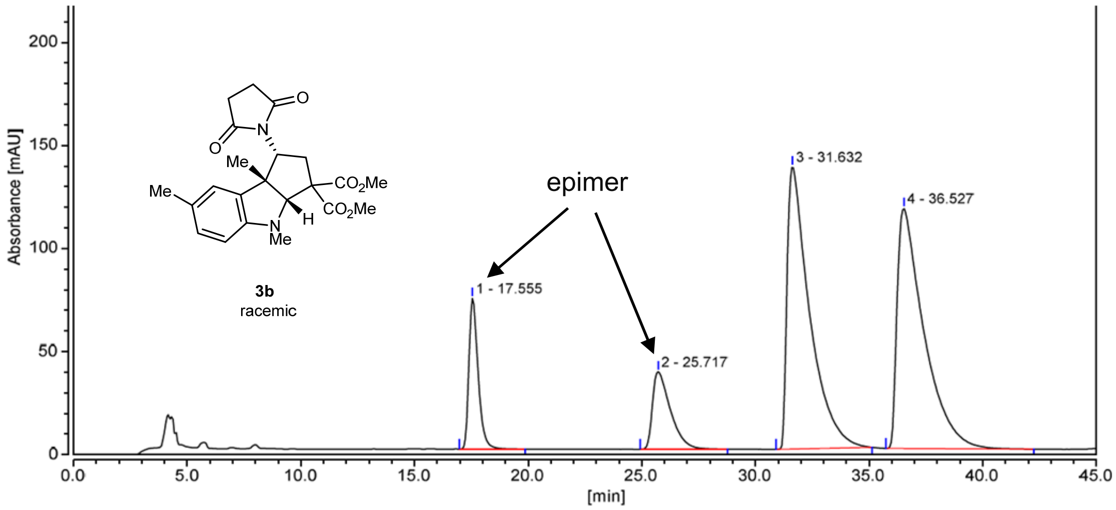


| Peak | Retention Time<br>min | Area<br>mAU*min | Height<br>mAU | Area<br>% | Height<br>% |
|---|---|---|---|---|---|
| 1 | 17.555 | 34.468 | 73.249 | 8.99 | 20.10 |
| 2 | 25.717 | 34.350 | 37.684 | 8.96 | 10.34 |
| 3 | 31.632 | 156.439 | 137.028 | 40.79 | 37.60 |
| 4 | 36.527 | 158.266 | 116.469 | 41.27 | 31.96 |
| **Total:** | | **383.523** | **364.430** | **100.00** | **100.00** |

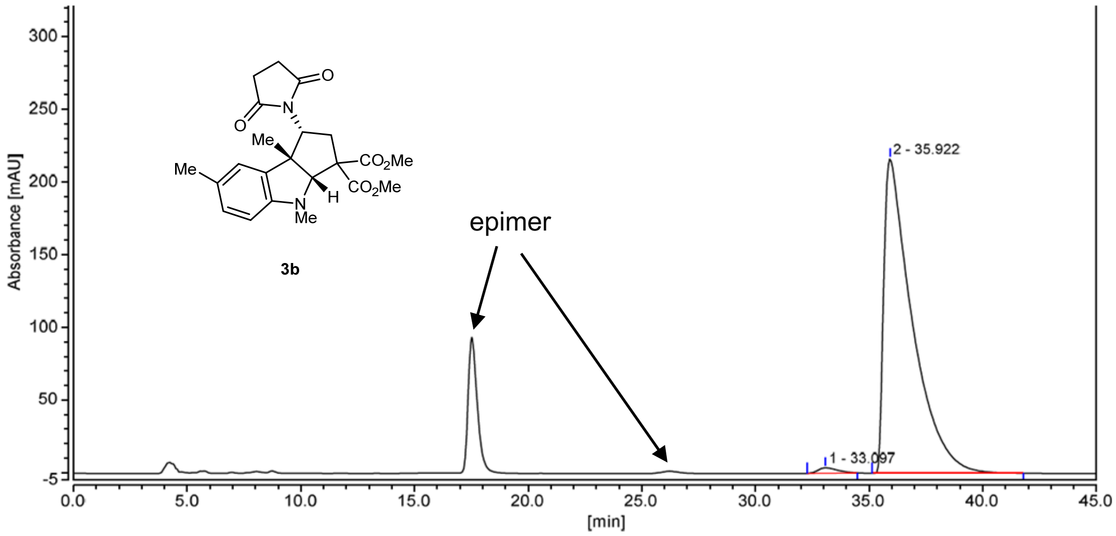


| Peak | Retention Time<br>min | Area<br>mAU*min | Height<br>mAU | Area<br>% | Height<br>% |
|---|---|---|---|---|---|
| 1 | 33.097 | 3.635 | 3.724 | 1.15 | 1.69 |
| 2 | 35.922 | 312.282 | 216.228 | 98.85 | 98.31 |
| **Total:** | | **315.918** | **219.952** | **100.00** | **100.00** |

Supplementary Fig. 115 HPLC spectra of **3c**

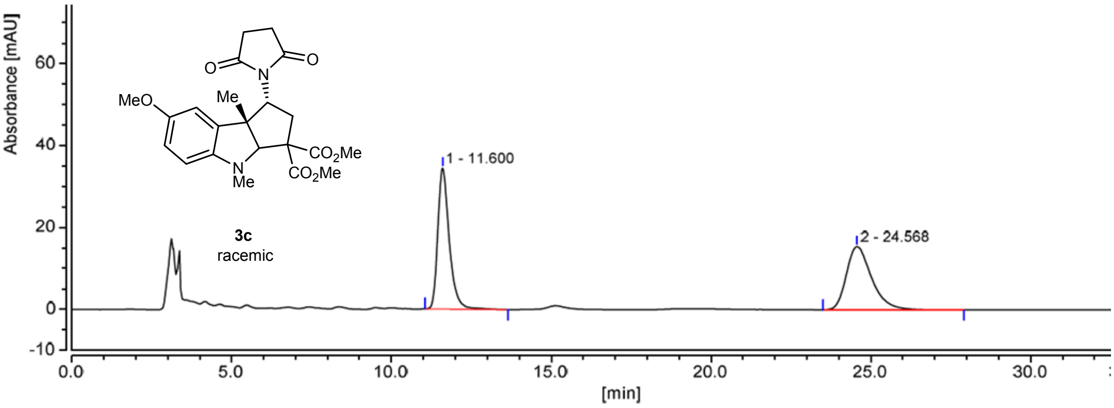


| Peak | Retention Time min | Area mAU*min | Height mAU | Area % | Height % |
|---|---|---|---|---|---|
| 1 | 11.600 | 14.267 | 34.365 | 50.20 | 68.97 |
| 2 | 24.568 | 14.153 | 15.458 | 49.80 | 31.03 |
| **Total:** | | **28.420** | **49.823** | **100.00** | **100.00** |

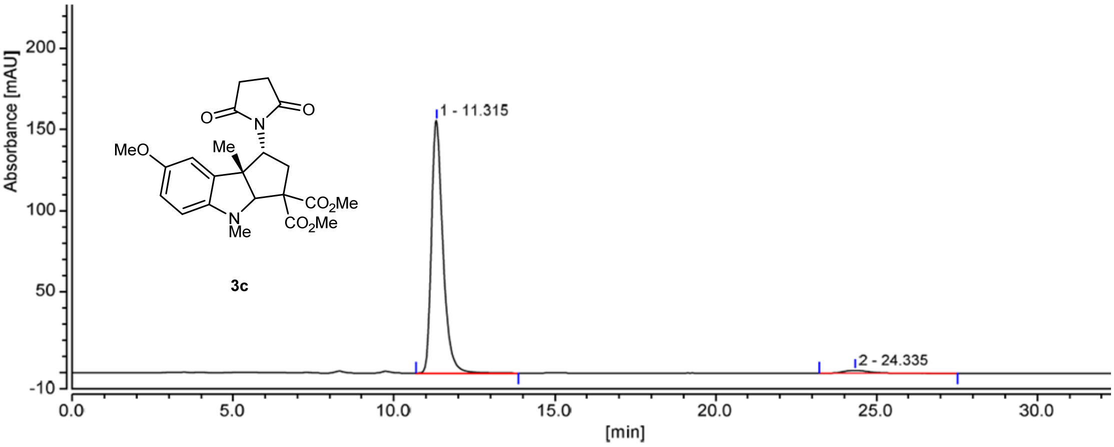


| Peak | Retention Time min | Area mAU*min | Height mAU | Area % | Height % |
|---|---|---|---|---|---|
| 1 | 11.315 | 64.337 | 155.931 | 97.45 | 98.86 |
| 2 | 24.335 | 1.682 | 1.802 | 2.55 | 1.14 |
| **Total:** | | **66.019** | **157.733** | **100.00** | **100.00** |

Supplementary Fig. 116 HPLC spectra of **3d**

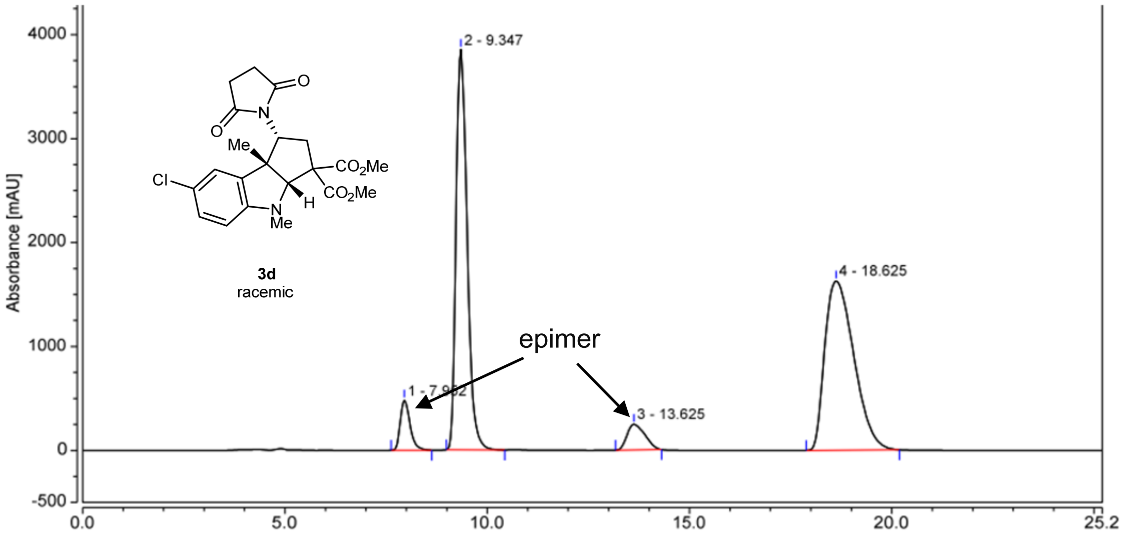


| Peak | Retention Time min | Area mAU*min | Height mAU | Area % | Height % |
|---|---|---|---|---|---|
| 1 | 7.952 | 135.097 | 482.353 | 4.75 | 7.77 |
| 2 | 9.347 | 1227.528 | 3850.391 | 43.13 | 62.02 |
| 3 | 13.625 | 126.323 | 247.065 | 4.44 | 3.98 |
| 4 | 18.625 | 1357.118 | 1628.119 | 47.68 | 26.23 |
| **Total:** | | **2846.066** | **6207.928** | **100.00** | **100.00** |

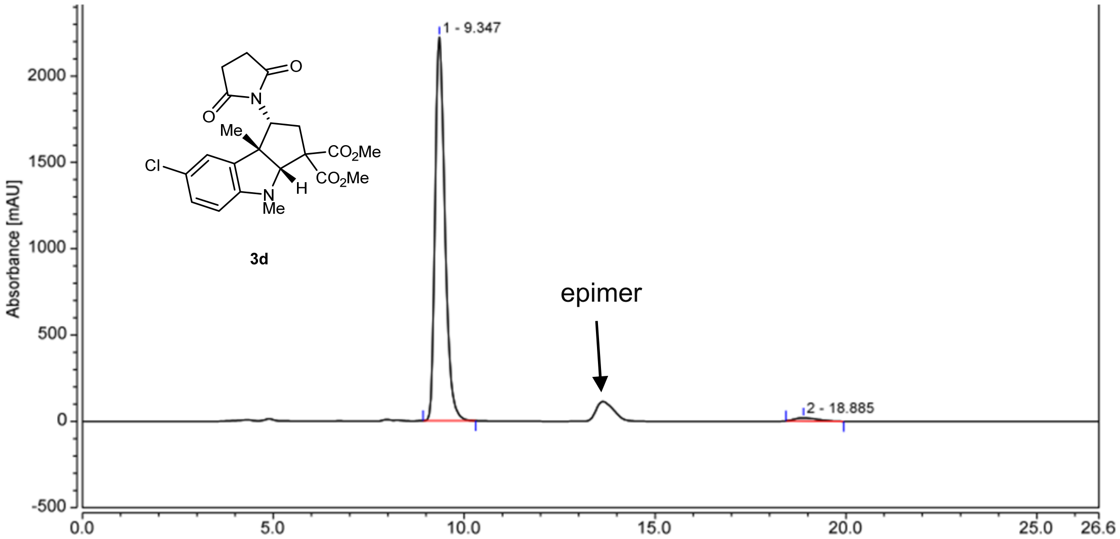


| Peak | Retention Time min | Area mAU*min | Height mAU | Area % | Height % |
|---|---|---|---|---|---|
| 1 | 9.347 | 665.966 | 2221.788 | 98.16 | 99.20 |
| 2 | 18.885 | 12.502 | 17.868 | 1.84 | 0.80 |
| **Total:** | | **678.469** | **2239.655** | **100.00** | **100.00** |

Supplementary Fig. 117 HPLC spectra of **3e**

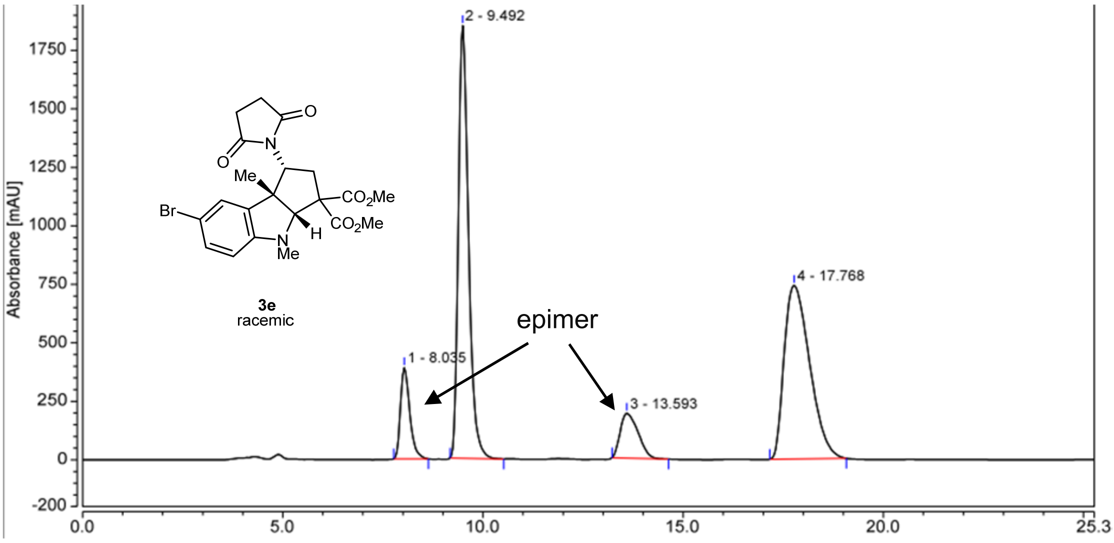


| Peak | Retention Time min | Area mAU*min | Height mAU | Area % | Height % |
|---|---|---|---|---|---|
| 1 | 8.035 | 105.241 | 390.059 | 8.11 | 12.29 |
| 2 | 9.492 | 545.576 | 1849.702 | 42.03 | 58.30 |
| 3 | 13.593 | 98.605 | 191.308 | 7.60 | 6.03 |
| 4 | 17.768 | 548.594 | 741.819 | 42.26 | 23.38 |
| **Total:** | | **1298.015** | **3172.888** | **100.00** | **100.00** |

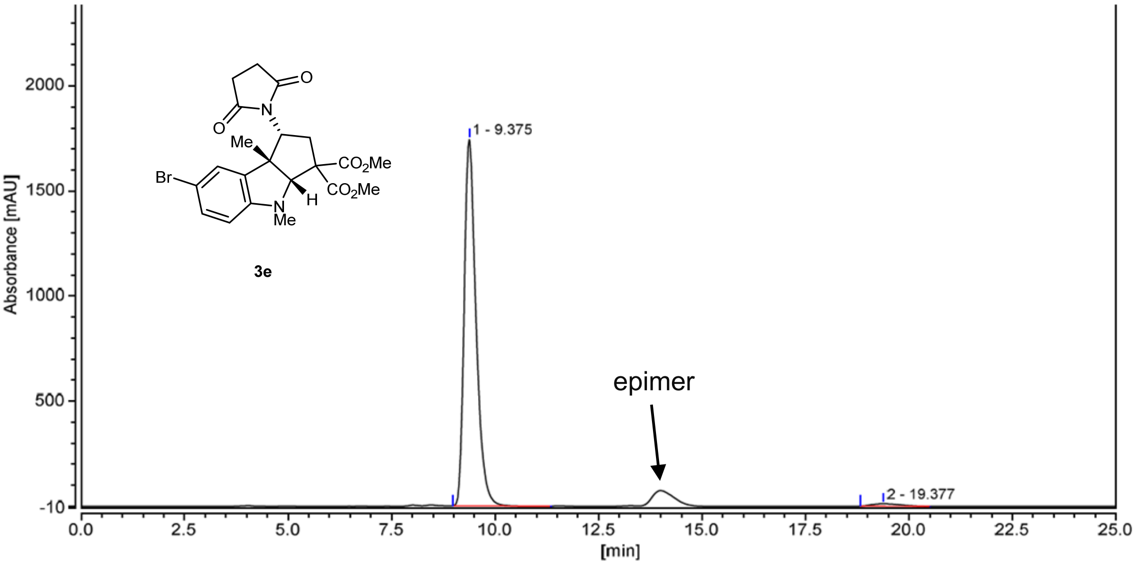


| Peak | Retention Time min | Area mAU*min | Height mAU | Area % | Height % |
|---|---|---|---|---|---|
| 1 | 9.375 | 576.094 | 1743.688 | 98.37 | 99.33 |
| 2 | 19.377 | 9.529 | 11.703 | 1.63 | 0.67 |
| **Total:** | | **585.622** | **1755.390** | **100.00** | **100.00** |

Supplementary Fig. 118 HPLC spectra of **3f**

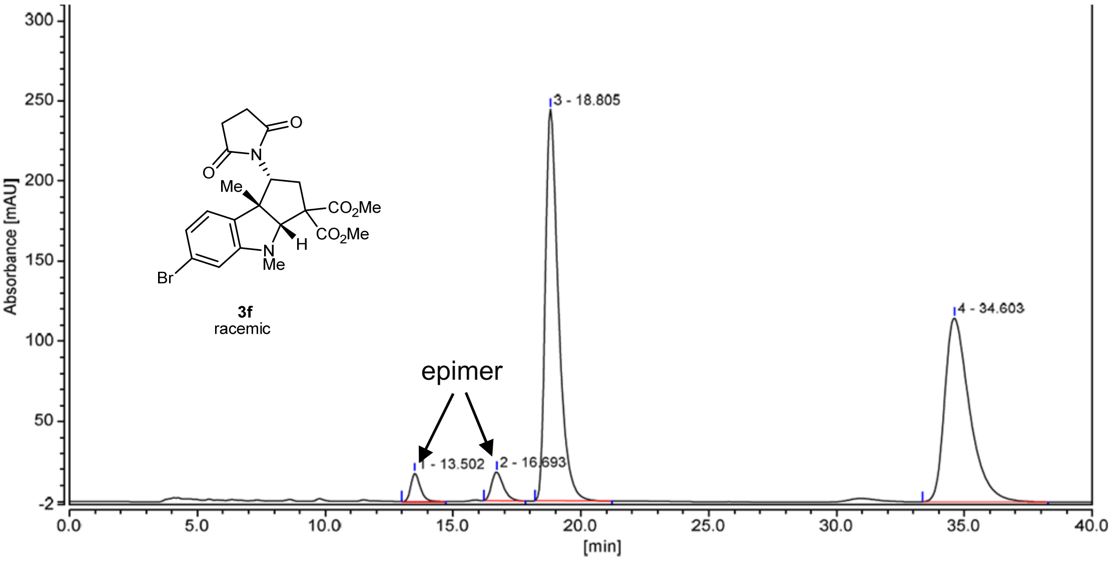


| Peak | Retention Time<br>min | Area<br>mAU*min | Height<br>mAU | Area<br>% | Height<br>% |
|---|---|---|---|---|---|
| 1 | 13.502 | 7.578 | 17.578 | 2.59 | 4.46 |
| 2 | 16.693 | 9.492 | 17.902 | 3.24 | 4.54 |
| 3 | 18.805 | 145.453 | 244.120 | 47.72 | 61.92 |
| 4 | 34.603 | 130.040 | 114.671 | 46.45 | 29.08 |
| **Total:** | | **292.562** | **394.271** | **100.00** | **100.00** |

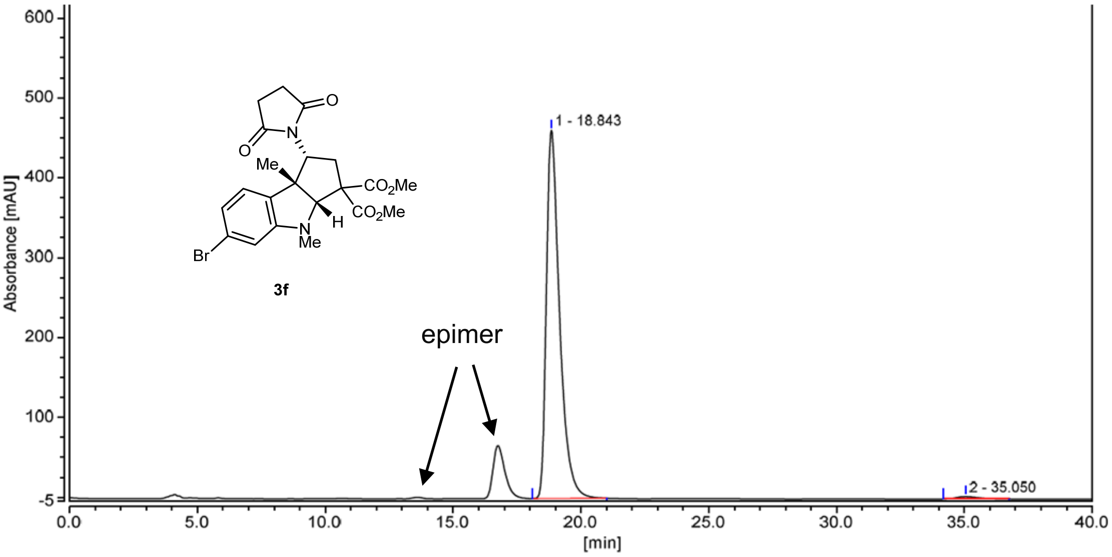


| Peak | Retention Time<br>min | Area<br>mAU*min | Height<br>mAU | Area<br>% | Height<br>% |
|---|---|---|---|---|---|
| 1 | 18.843 | 275.901 | 460.981 | 99.25 | 99.49 |
| 2 | 35.050 | 2.077 | 2.361 | 0.75 | 0.51 |
| **Total:** | | **277.978** | **463.342** | **100.00** | **100.00** |

.Supplementary Fig. 119 HPLC spectra of **3g**

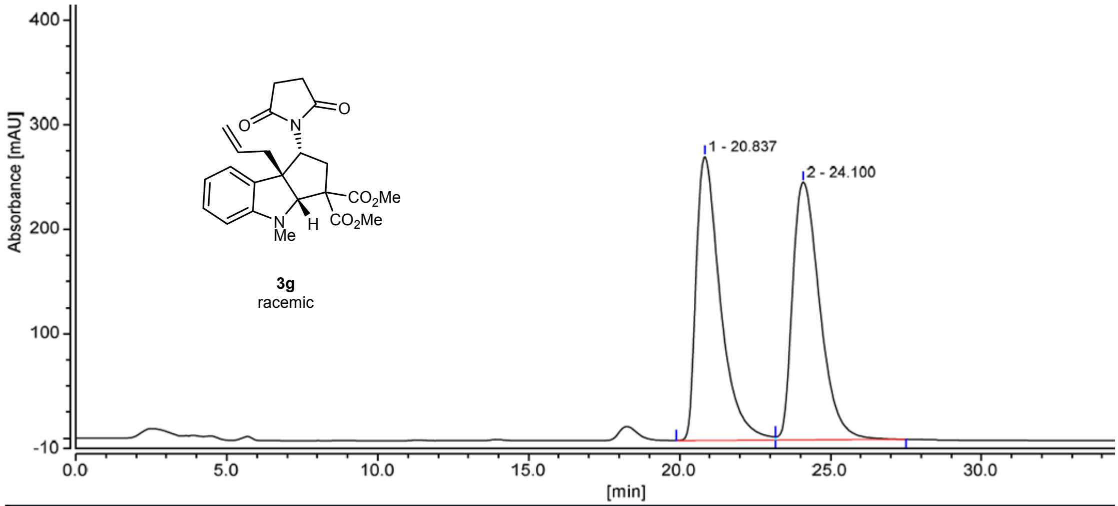


| Peak | Retention Time<br>min | Area<br>mAU*min | Height<br>mAU | Area<br>% | Height<br>% |
|---|---|---|---|---|---|
| 1 | 20.837 | 252.786 | 271.763 | 49.57 | 52.40 |
| 2 | 24.100 | 257.125 | 246.855 | 50.43 | 47.60 |
| **Total:** | | **509.911** | **518.618** | **100.00** | **100.00** |

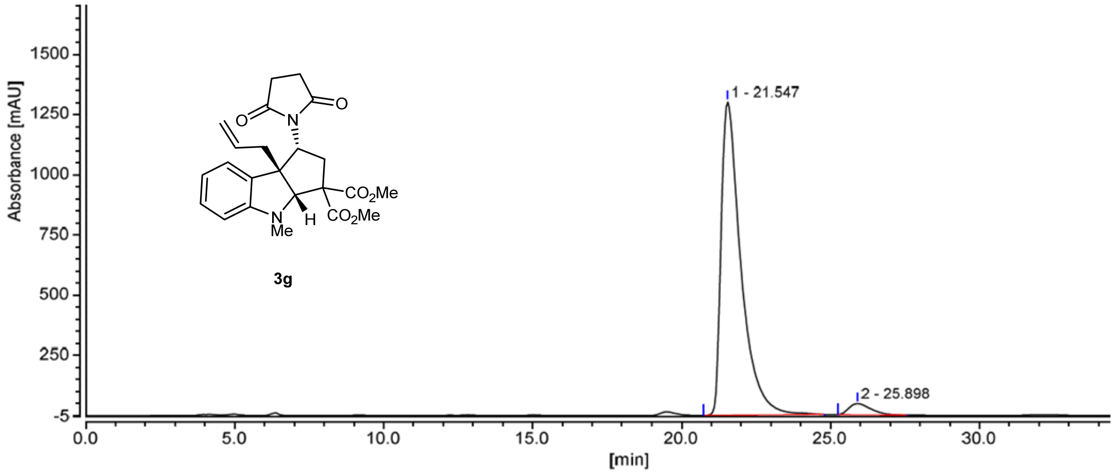


| Peak | Retention Time<br>min | Area<br>mAU*min | Height<br>mAU | Area<br>% | Height<br>% |
|---|---|---|---|---|---|
| 1 | 21.547 | 1000.668 | 1300.892 | 96.22 | 96.59 |
| 2 | 25.898 | 39.309 | 45.968 | 3.78 | 3.41 |
| **Total:** | | **1039.976** | **1346.859** | **100.00** | **100.00** |

Supplementary Fig. 120 HPLC spectra of **3h**

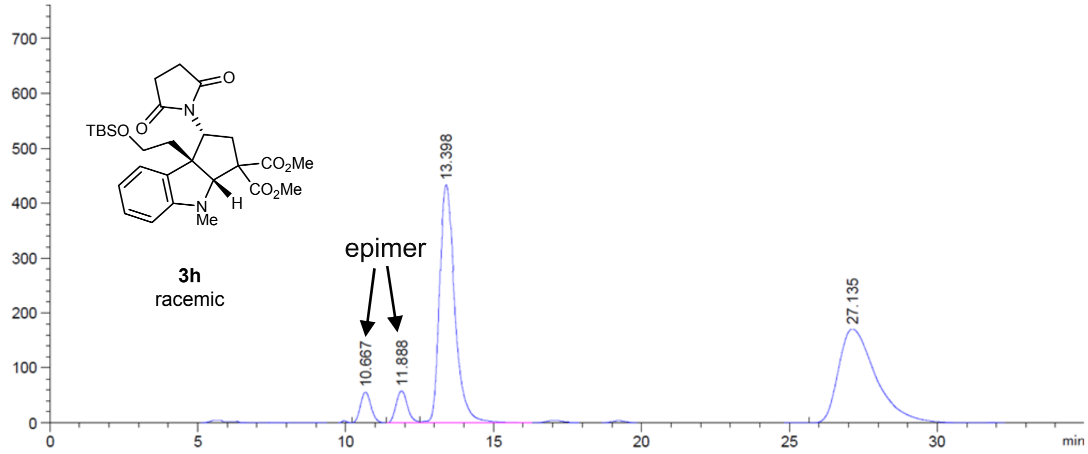


| # | [min] | | [min] | [mAU*s] | [mAU] | % |
|---|---|---|---|---|---|---|
| 1 | 10.667 | VB | 0.3917 | 1431.90552 | 56.82360 | 4.2233 |
| 2 | 11.888 | BV | 0.4188 | 1593.64465 | 58.70010 | 4.7003 |
| 3 | 13.398 | VB | 0.5369 | 1.56367e4 | 434.01111 | 46.1193 |
| 4 | 27.135 | VB | 1.2915 | 1.52426e4 | 172.07320 | 44.9570 |

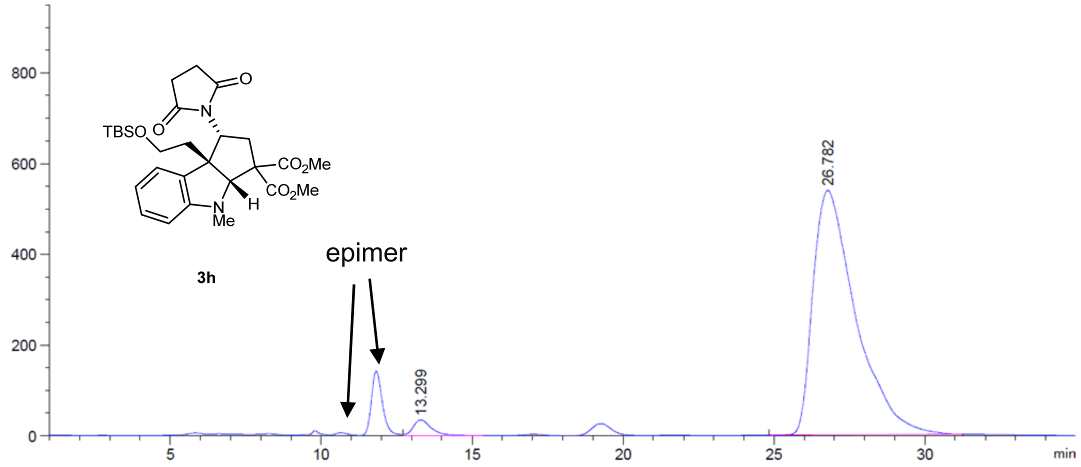


| # | [min] | | [min] | [mAU*s] | [mAU] | % |
|---|---|---|---|---|---|---|
| 1 | 13.299 | VB | 0.6076 | 1445.90393 | 34.35489 | 2.6529 |
| 2 | 26.782 | BB | 1.3211 | 5.30577e4 | 539.36639 | 97.3471 |

Supplementary Fig. 121 HPLC spectra of **3i**

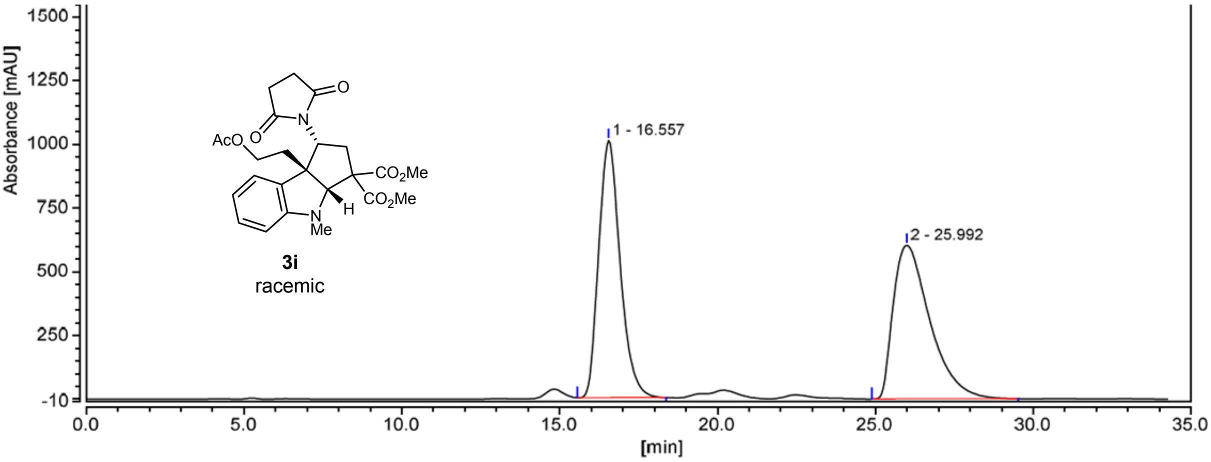


| Peak | Retention Time min | Area mAU*min | Height mAU | Area % | Height % |
|---|---|---|---|---|---|
| 1 | 16.557 | 795.481 | 1009.831 | 49.51 | 62.60 |
| 2 | 25.992 | 811.065 | 603.299 | 50.49 | 37.40 |
| **Total:** | | **1606.546** | **1613.130** | **100.00** | **100.00** |

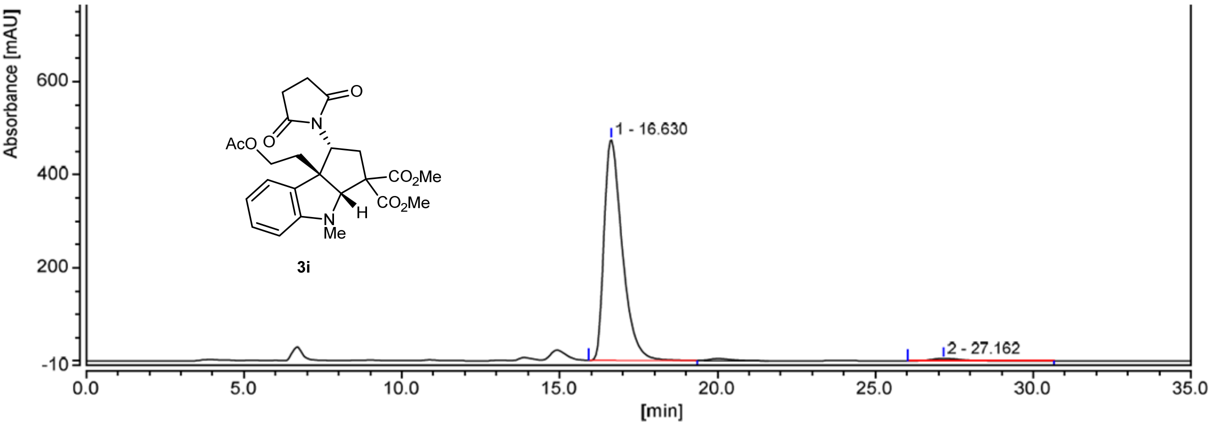


| Peak | Retention Time min | Area mAU*min | Height mAU | Area % | Height % |
|---|---|---|---|---|---|
| 1 | 16.630 | 317.191 | 474.952 | 98.20 | 98.93 |
| 2 | 27.162 | 5.809 | 5.158 | 1.80 | 1.07 |
| **Total:** | | **323.000** | **480.110** | **100.00** | **100.00** |

Supplementary Fig. 122 HPLC spectra of **3j**

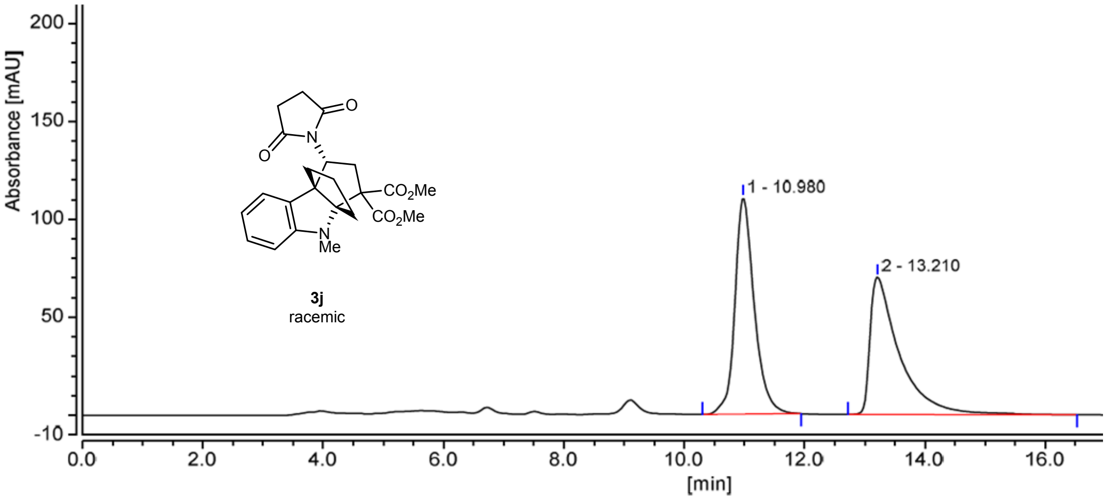


| Peak | Retention Time min | Area mAU*min | Height mAU | Area % | Height % |
|---|---|---|---|---|---|
| 1 | 10.980 | 41.103 | 110.357 | 50.92 | 61.15 |
| 2 | 13.210 | 39.622 | 70.116 | 49.08 | 38.85 |
| **Total:** | | **80.725** | **180.473** | **100.00** | **100.00** |

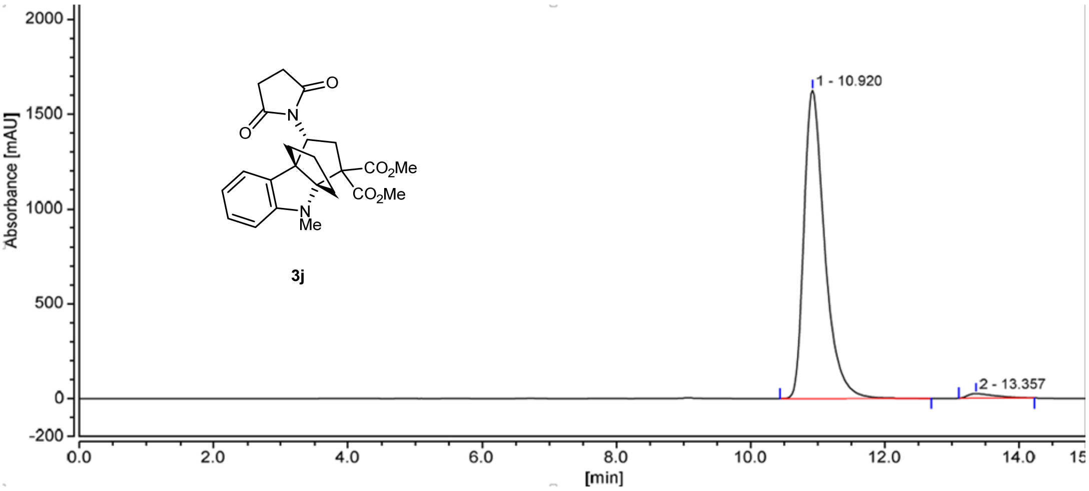


| Peak | Retention Time min | Area mAU*min | Height mAU | Area % | Height % |
|---|---|---|---|---|---|
| 1 | 10.920 | 581.761 | 1626.435 | 98.08 | 98.58 |
| 2 | 13.357 | 11.365 | 23.418 | 1.92 | 1.42 |
| **Total:** | | **593.126** | **1649.853** | **100.00** | **100.00** |

Supplementary Fig. 123 HPLC spectra of **3k**

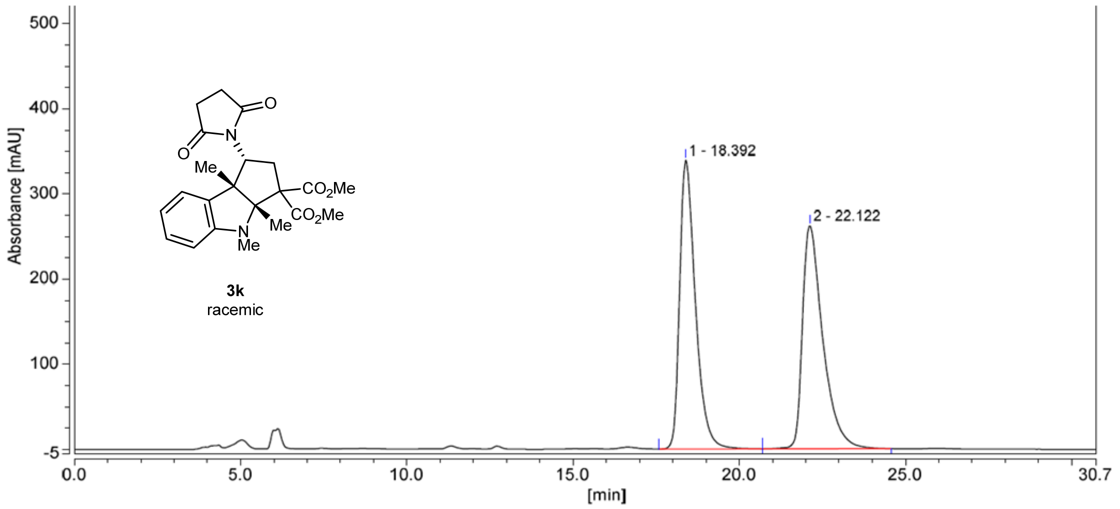


| Retention Time min | Area mAU*min | Heigh mAU | Area % | Height % |
|---|---|---|---|---|
| 18.392 | 186.202 | 339.357 | 50.00 | 56.37 |
| 22.122 | 186.222 | 262.674 | 50.00 | 43.63 |
| | **372.423** | **602.031** | **100.00** | **100.00** |

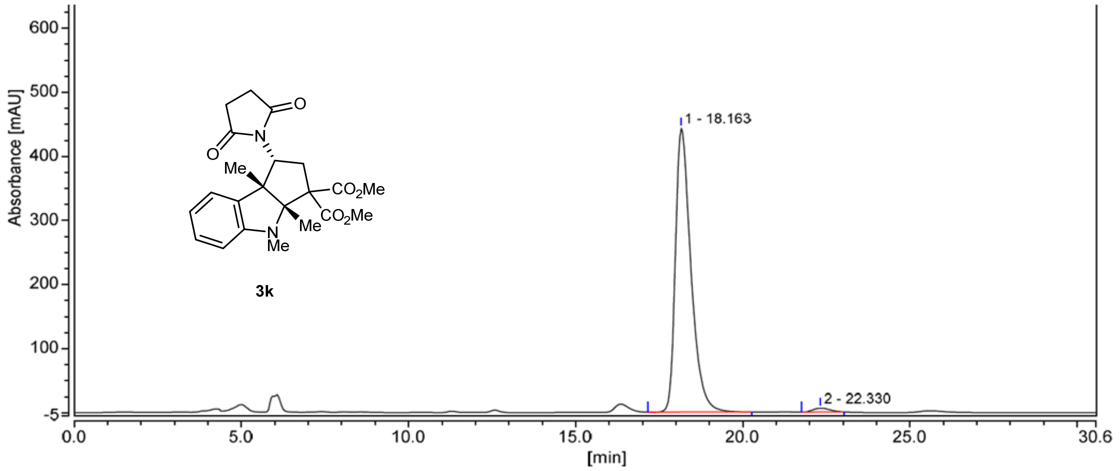


| Retention Time min | Area mAU*min | Heigh mAU | Area % | Height % |
|---|---|---|---|---|
| 18.163 | 238.605 | 443.641 | 98.50 | 98.67 |
| 22.330 | 3.628 | 5.990 | 1.50 | 1.33 |
| | **242.234** | **449.631** | **100.00** | **100.00** |

Supplementary Fig. 124 HPLC spectra of **3l**

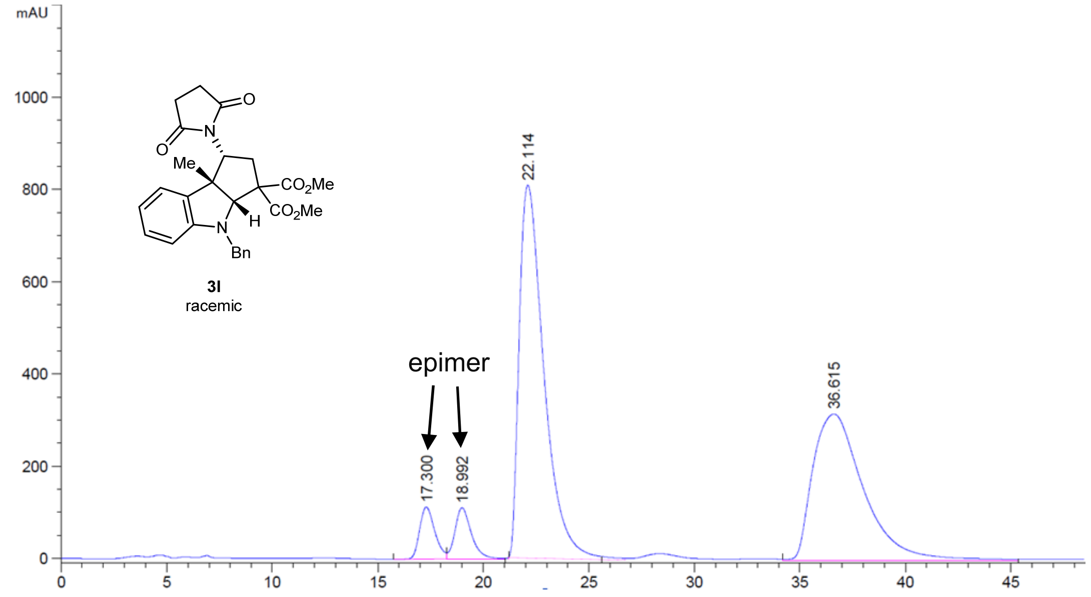


| Peak # | RetTime [min] | Type | Width [min] | Area [mAU*s] | Height [mAU] | Area % |
|---|---|---|---|---|---|---|
| 1 | 17.300 | BV | 0.7735 | 5666.07568 | 113.18438 | 4.3829 |
| 2 | 18.992 | VV | 0.8151 | 6017.70313 | 111.18372 | 4.6549 |
| 3 | 22.114 | MM | 1.3339 | 6.39044e4 | 798.49506 | 45.4326 |
| 4 | 36.615 | MM | 2.8129 | 5.36875e4 | 318.10135 | 45.5296 |

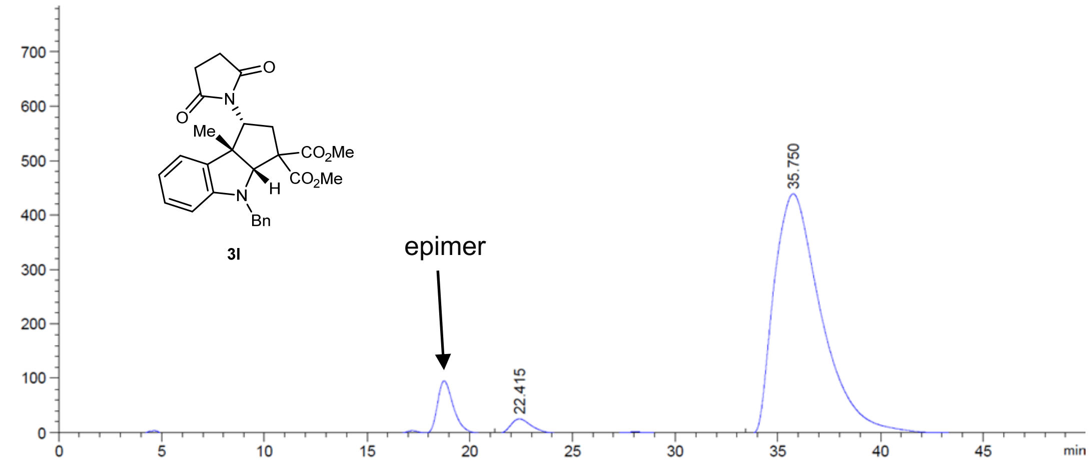


| Peak # | RetTime [min] | Type | Width [min] | Area [mAU*s] | Height [mAU] | Area % |
|---|---|---|---|---|---|---|
| 1 | 22.415 | VB | 1.1660 | 2179.29883 | 28.13662 | 2.9944 |
| 2 | 35.750 | BBA | 2.4733 | 7.05999e4 | 441.19867 | 97.0056 |

Supplementary Fig. 125 HPLC spectra of **3m**

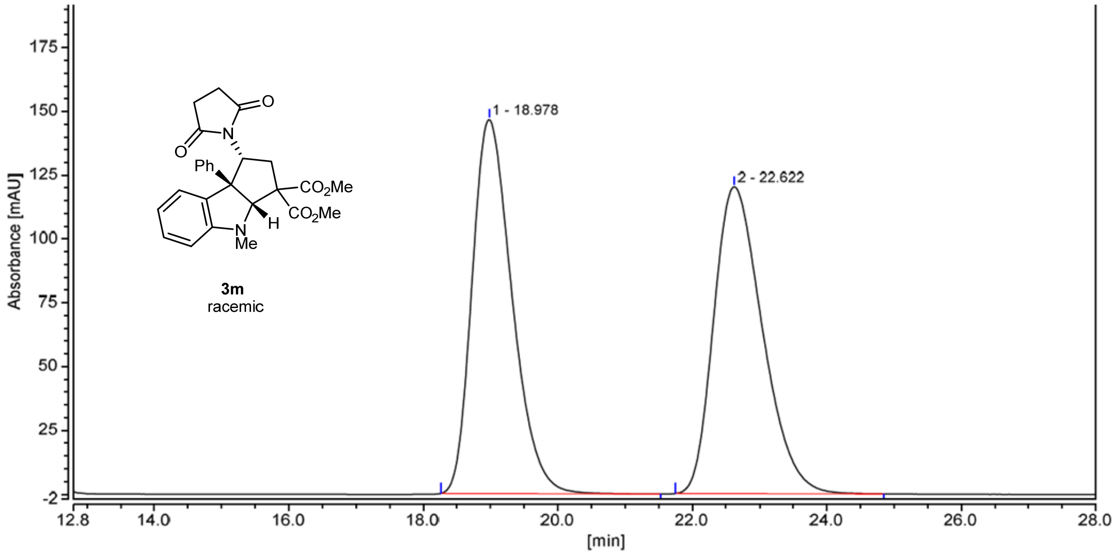


| Peak | Retention Time<br>min | Area<br>mAU*min | Height<br>mAU | Area<br>% | Height<br>% |
|---|---|---|---|---|---|
| 1 | 18.978 | 100.708 | 146.610 | 49.95 | 54.94 |
| 2 | 22.622 | 100.896 | 120.254 | 50.05 | 45.06 |
| **Total:** | | **201.604** | **266.864** | **100.00** | **100.00** |

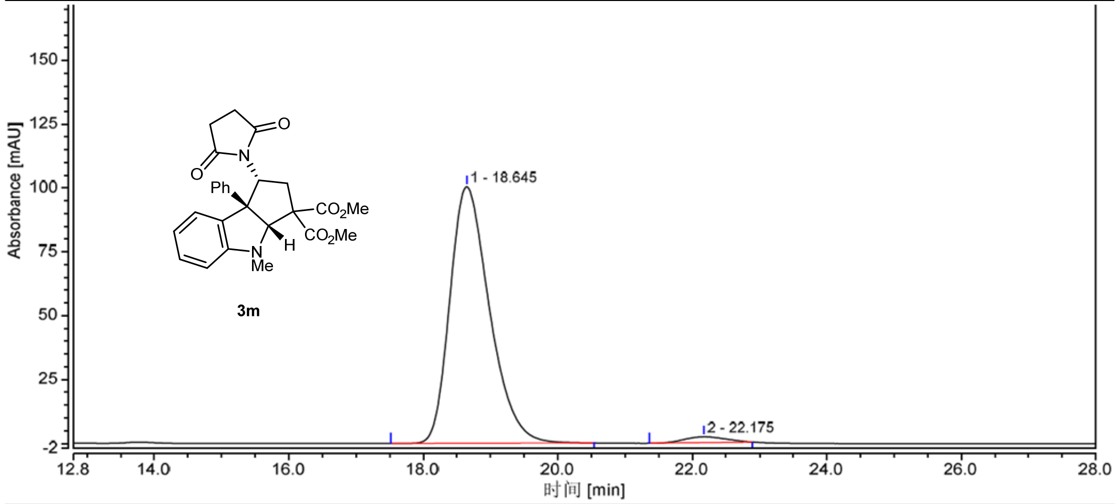


| Peak | Retention Time<br>min | Area<br>mAU*min | Height<br>mAU | Area<br>% | Height<br>% |
|---|---|---|---|---|---|
| 1 | 18.645 | 68.560 | 100.491 | 97.68 | 97.78 |
| 2 | 22.175 | 1.627 | 2.280 | 2.32 | 2.22 |
| **Total:** | | **70.187** | **102.771** | **100.00** | **100.00** |

Supplementary Fig. 126 HPLC spectra of **3n**

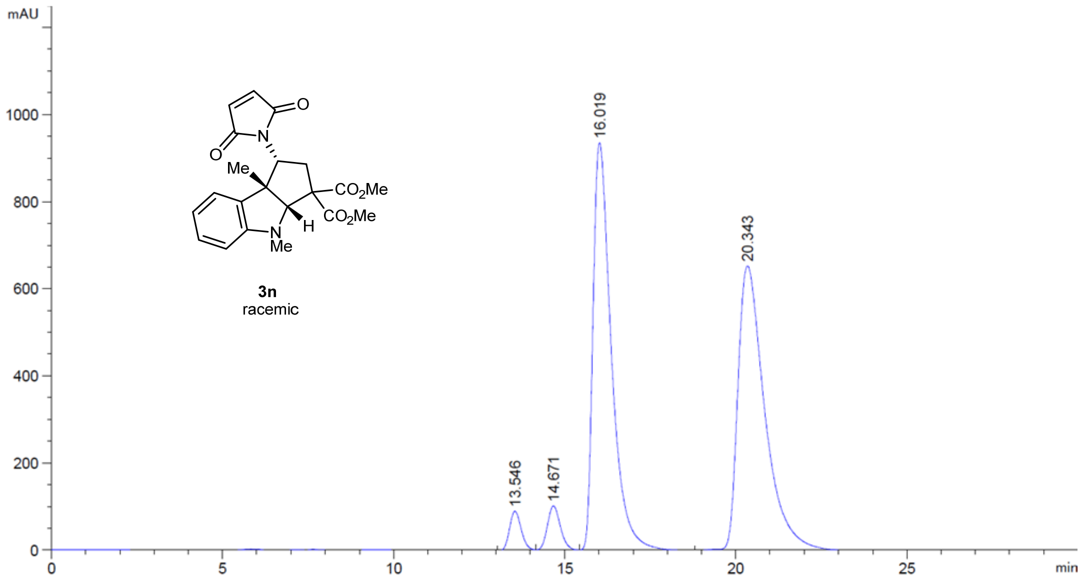


| Peak # | RetTime [min] | Type | Width [min] | Area [mAU*s] | Height [mAU] | Area % |
|---|---|---|---|---|---|---|
| 1 | 13.546 | BV | 0.3726 | 2191.82617 | 91.03405 | 2.9064 |
| 2 | 14.671 | VB | 0.4078 | 2720.20728 | 102.27887 | 3.6070 |
| 3 | 16.019 | BB | 0.5578 | 3.48478e4 | 936.72760 | 46.2082 |
| 4 | 20.343 | BBA | 0.7945 | 3.56549e4 | 654.29443 | 47.2784 |

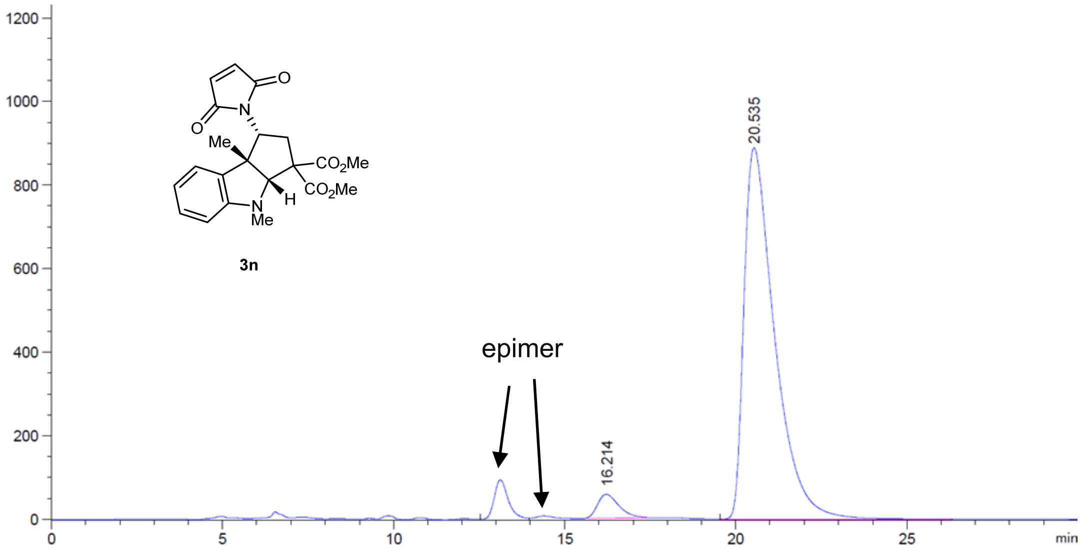


| Peak # | RetTime [min] | Type | Width [min] | Area [mAU*s] | Height [mAU] | Area % |
|---|---|---|---|---|---|---|
| 1 | 16.214 | BB | 0.6695 | 2553.23413 | 57.98306 | 4.4092 |
| 2 | 20.535 | BB | 0.9164 | 5.53534e4 | 888.11847 | 95.5908 |

Supplementary Fig. 127 HPLC spectra of **3o**

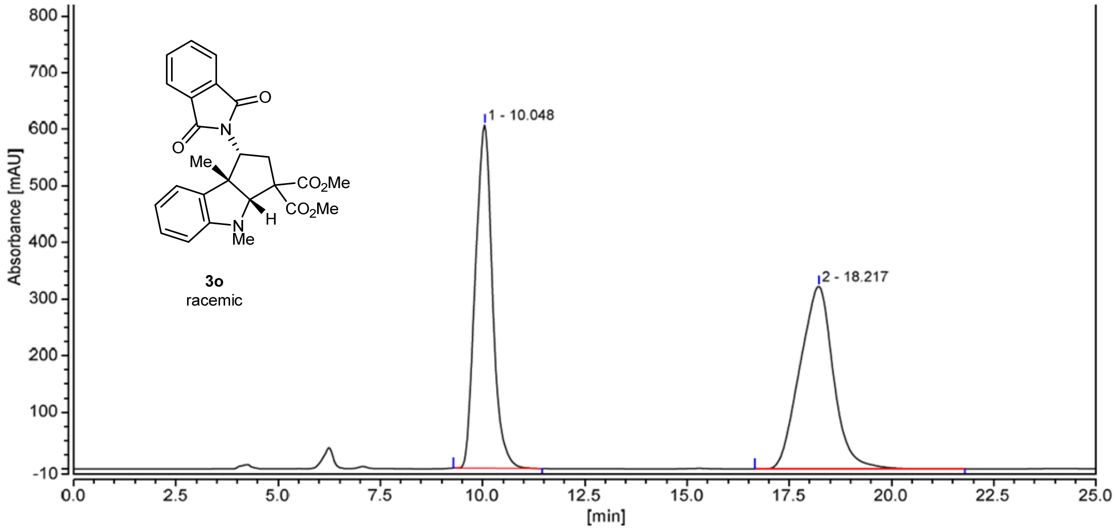


| Peak | Retention Time min | Area mAU*min | Height mAU | Area % | Height % |
|---|---|---|---|---|---|
| 1 | 10.048 | 293.781 | 606.845 | 48.90 | 65.28 |
| 2 | 18.217 | 307.023 | 322.772 | 51.10 | 34.72 |
| **Total:** | | **600.805** | **929.617** | **100.00** | **100.00** |

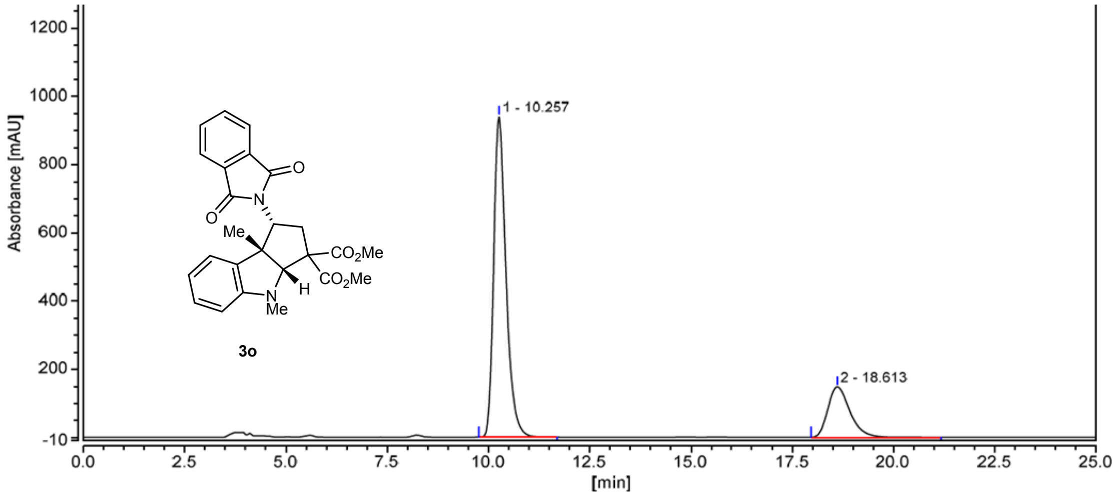


| Peak | Retention Time min | Area mAU*min | Height mAU | Area % | Height % |
|---|---|---|---|---|---|
| 1 | 10.257 | 330.751 | 938.972 | 77.86 | 86.37 |
| 2 | 18.613 | 94.042 | 148.194 | 22.14 | 13.63 |
| **Total:** | | **424.793** | **1087.166** | **100.00** | **100.00** |

Supplementary Fig. 128 HPLC spectra of **3p**

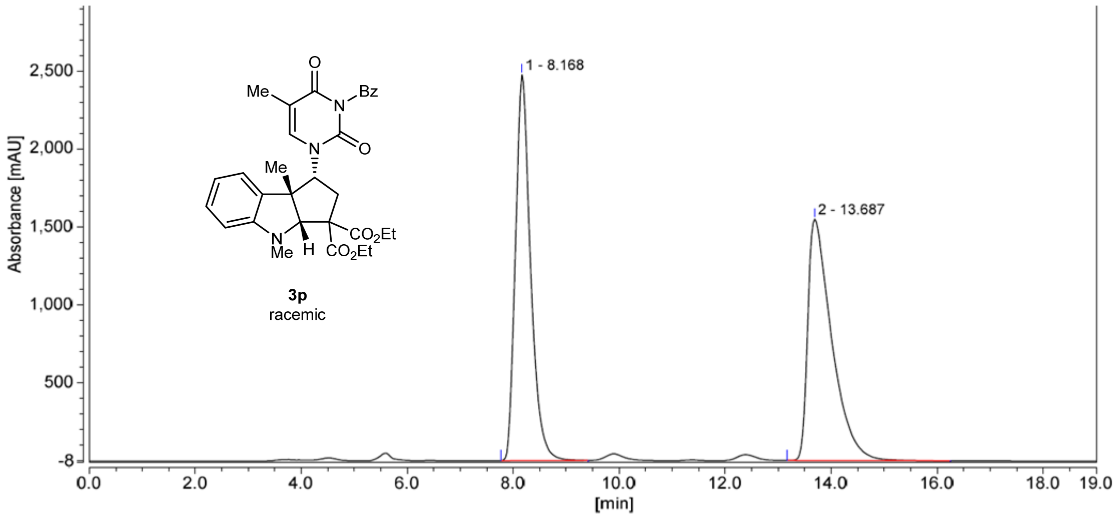


| Retention Time min | Area mAU*min | Heigh mAU | Area % | Height % |
|---|---|---|---|---|
| 8.168 | 818.411 | 2476.735 | 50.28 | 61.52 |
| 13.687 | 809.310 | 1548.859 | 49.72 | 38.48 |
| | **1627.721** | **4025.594** | **100.00** | **100.00** |

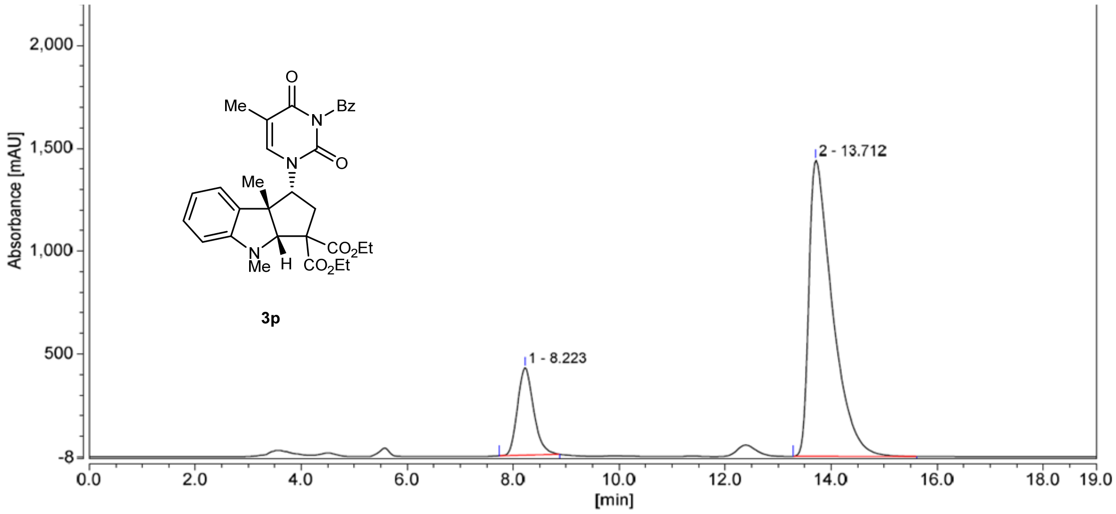


| Retention Time min | Area mAU*min | Heigh mAU | Area % | Height % |
|---|---|---|---|---|
| 8.223 | 150.050 | 425.289 | 16.85 | 22.79 |
| 13.712 | 740.671 | 1440.672 | 83.15 | 77.21 |
| | **890.722** | **1865.961** | **100.00** | **100.00** |

Supplementary Fig. 129 HPLC spectra of **5a**

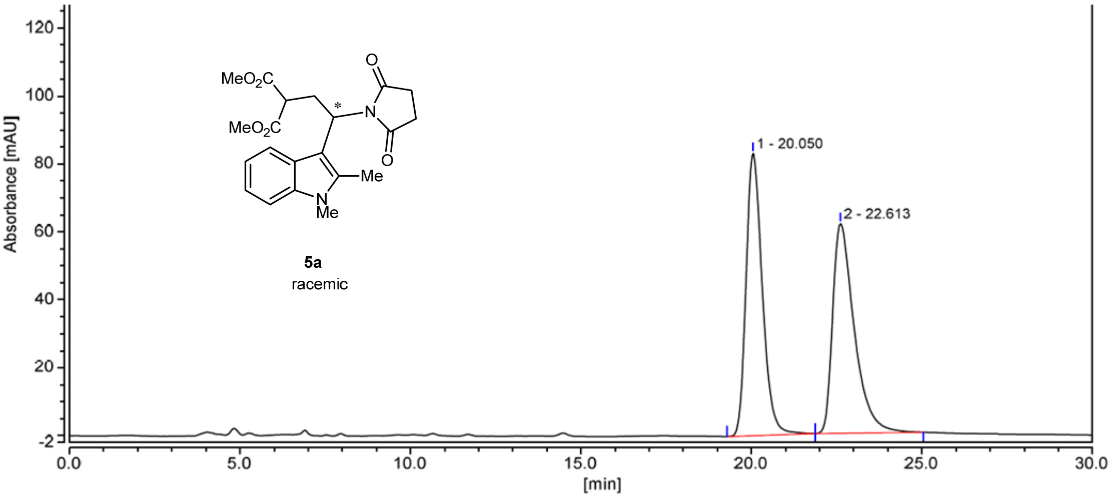


| Peak | Retention Time<br>min | Area<br>mAU*min | Height<br>mAU | Area<br>% | Height<br>% |
|---|---|---|---|---|---|
| 1 | 20.050 | 45.852 | 83.111 | 50.29 | 57.37 |
| 2 | 22.613 | 45.322 | 61.753 | 49.71 | 42.63 |
| **Total:** | | **91.173** | **144.865** | **100.00** | **100.00** |

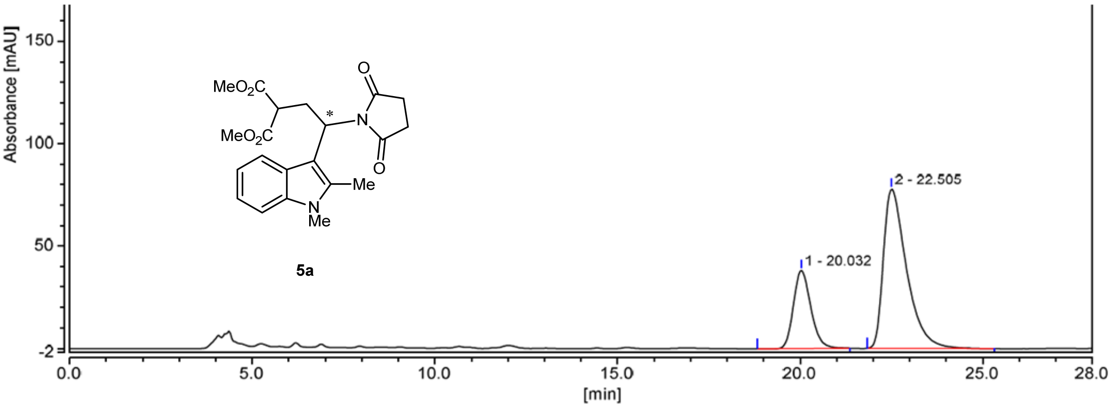


| Peak | Retention Time<br>min | Area<br>mAU*min | Height<br>mAU | Area<br>% | Height<br>% |
|---|---|---|---|---|---|
| 1 | 20.032 | 21.060 | 38.145 | 26.90 | 32.90 |
| 2 | 22.505 | 57.225 | 77.787 | 73.10 | 67.10 |
| **Total:** | | **78.285** | **115.931** | **100.00** | **100.00** |

Supplementary Fig. 130 HPLC spectra of **5b**

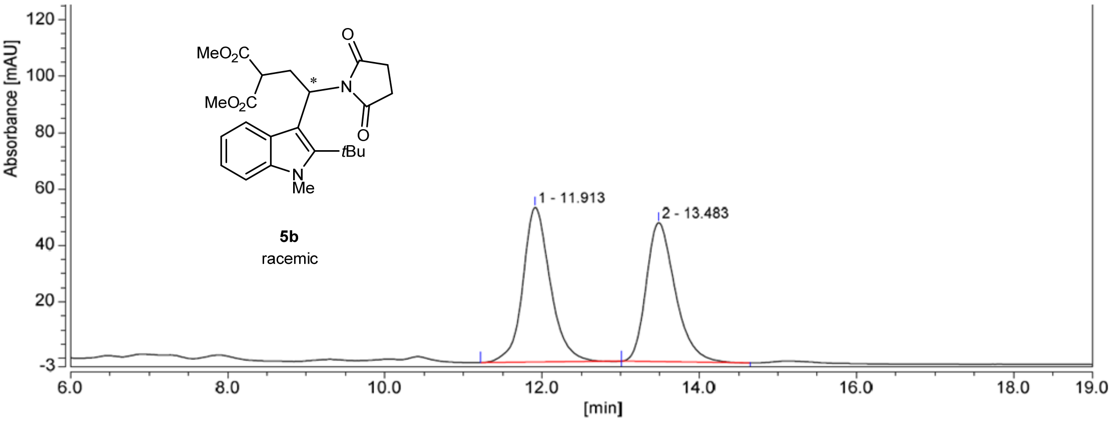


| Retention Time min | Area mAU*min | Heigh mAU | Area % | Height % |
|---|---|---|---|---|
| 11.913 | 22.164 | 54.884 | 51.21 | 52.67 |
| 13.483 | 21.118 | 49.318 | 48.79 | 47.33 |
| | **43.282** | **104.202** | **100.00** | **100.00** |

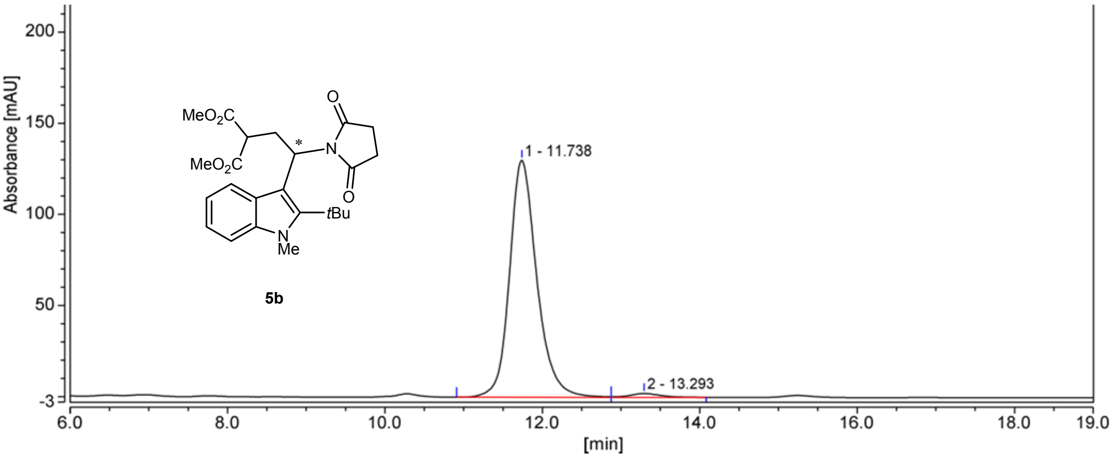


| Retention Time min | Area mAU*min | Heigh mAU | Area % | Height % |
|---|---|---|---|---|
| 11.738 | 52.283 | 130.062 | 98.22 | 98.42 |
| 13.293 | 0.949 | 2.088 | 1.78 | 1.58 |
| | **53.232** | **132.149** | **100.00** | **100.00** |

## Quantum chemical calculations

The electron correlation effects were considered by employing the density functional theory (DFT)[9,10] with Gaussian 16 program[11]. The ultrafine grid (99,590), having 99 radial shells and 590 angular points per shell, was used to evaluate the numerical integration accuracy. Geometry optimizations are performed at M06-2X-D3[12,13] level of theory with the double-zeta basis set Def2-SVP[14] in the gas phase (298.15 K, 0.1 MPa). The harmonic vibrational frequencies were analyzed after the geometry optimizations to characterize the nature of the stationary point as a minimum with all positive frequencies or as a transition state with only one imaginary frequency and to provide the zero-point energy ($E_{ZPE}$), total entropy ($S_{tot}$) and thermal correction to enthalpy ($H_{corr}$) at the same theoretical level. Then, the intrinsic reaction coordinate (IRC)[15] calculations are carried out to verify the transition state (TS) associated with the correct reactant complexes (RC), intermediate (IM) and product complexes (PC) at the same level of theory. Based on the optimized structures, the electronic energy ($E_{electron}$) and solvation free energy ($\Delta G_{solv}$) are calculated at the M06-2X-D3[12,13] level of theory with the triple-zeta basis set Def2-TZVP[14] in dichloromethane medium while using Truhlar's SMD solvation model[16]. The Gibbs free energy of free substrate 1**a** ($G_{\mathbf{1a}}$) and complex of **2a** with catalyst ($G_{\mathbf{c\text{-}2a}}$) were defined as reference-point.

$$G = E_{electron} + \Delta G_{solv} + E_{ZPE} + H_{corr} - TS_{tot} \quad (1)$$

$$\Delta G = G - (G_{\mathbf{1a}} + G_{\mathbf{c\text{-}2a}}) \quad (2)$$

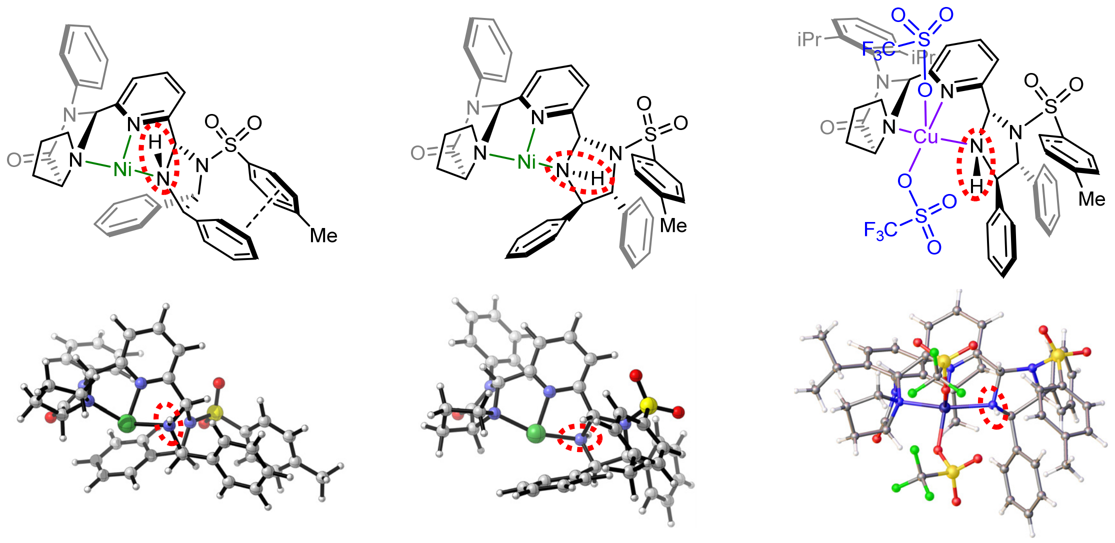


(a) c-1 ΔΔG = 0.0 (b) c-2 ΔΔG = 13.1 (c) CCDC: 2203570

**Supplementary Fig. 131** DFT-optimized structures and relative free energies (Δ*G*, kcal/mol) for the complex of Ni(II) with **L4** at the M06-2X-D3/Def2-TZVP/SMD(DCM)//M06-2X-D3/Def2-SVP level.

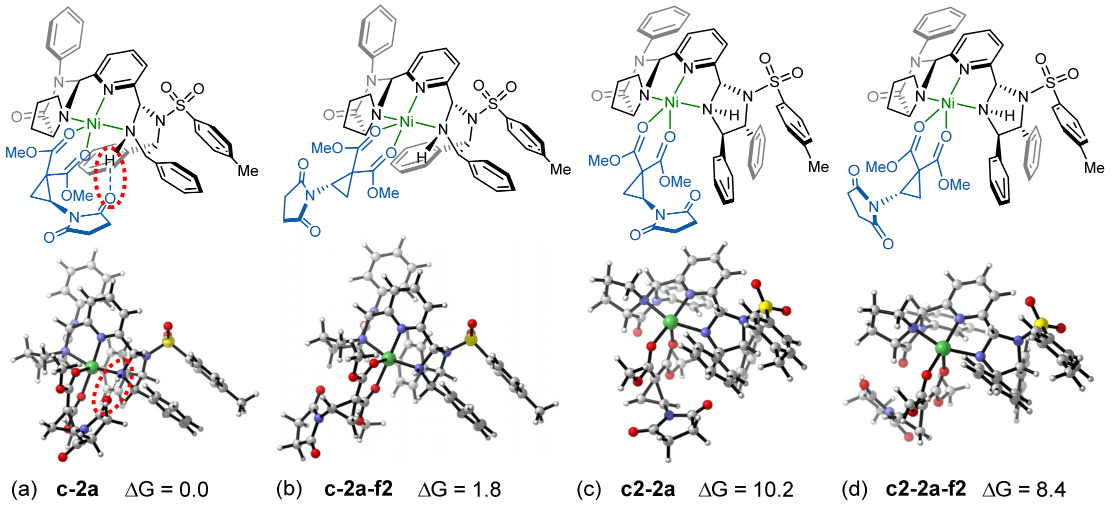


**Supplementary Fig. 132** DFT-optimized structures and relative free energies ($\Delta G$, kcal/mol) for the complex of Ni(**II**) with **L4** and **2a** at the M06-2X-D3/Def2-TZVP/SMD(DCM)//M06-2X-D3/Def2-SVP level.

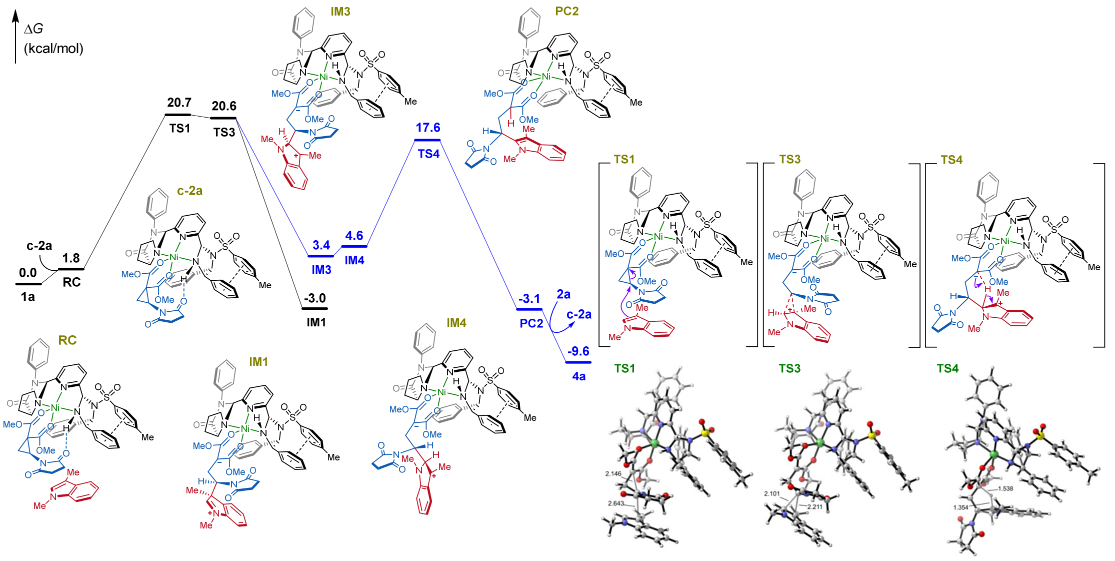


**Supplementary Fig. 133** Relative energy profiles (in kcal/mol) of bifurcating reaction pathway[17] and optimized structures of important transition state (bond lengths, Å) at the M06-2X-D3/Def2-TZVP/SMD(DCM)//M06-2X-D3/Def2-SVP level.

## Supplementary references

1. Xiong, H., Xu, H., Liao, S., Xie, Z. & Tang, Y. Copper-catalyzed highly enantioselective cyclopentannulation of indoles with donor-acceptor cyclopropanes. *J. Am. Chem. Soc*. **135**, 7851-7854 (2013).
2. Shang, M. et al. Exceedingly fast copper(II)-promoted *ortho* C-H trifluoromethylation of arenes using $TMSCF_3$. *Angew. Chem. Int. Ed.* **53**, 10439-10442 (2014).
3. DiPoto, M. C., Hughes, R. P. & Wu, J. Dearomative indole (3 + 2) reactions with azaoxyallyl cations-new method for the synthesis of pyrroloindolines. *J. Am. Chem. Soc*. **137**, 14861-14864 (2015).
4. Repka, L. M., Ni, J. & Reisman, S. E. Enantioselective synthesis of pyrroloindolines by a formal [3 + 2] cycloaddition reaction. *J. Am. Chem. Soc*. **132**, 14418-14420 (2010).
5. Preindl, J., Chakrabarty, S. & Waser, J. Dearomatization of electron poor six-membered N-heterocycles through [3 + 2] annulation with aminocyclopropanes. *Chem. Sci.* **8**, 7112-7118 (2017).
6. De Nanteuil, F., Loup, J. & Waser, J. Catalytic Friedel-Crafts reaction of aminocyclopropanes. *Org. Lett*. **15**, 3738-3741 (2013).
7. Zhang, M.-C. et al. Cu-catalyzed asymmetric dearomative [3 + 2] cycloaddition reaction of benzazoles with aminocyclopropanes. *Chem* **5**, 156-167 (2019).
8. Wang, H.-X. et al. Synthesis of chiral pyrimidine-substituted diester D-A cyclopropanes via asymmetric cyclopropanation of phenyliodonium ylides. *Chem. Commun.* **56**, 11649-11652 (2020).
9. Hohenberg, P. & Kohn, W. Inhomogeneous electron gas. *Phys. Rev*. **136**, B864-B871 (1964).
10. Kohn, W. & Sham, L. J. Self-consistent equations including exchange and correlation effects. *Phys. Rev*. **140**, A1133-A1138 (1965).
11. Frisch, M. J. et al. *Gaussian 16*, revision B.01, Gaussian, Inc.: Wallingford, CT, 2016.
12. Zhao, Y. & Truhlar, D. G. The M06 suite of density functionals for main group thermochemistry, thermochemical kinetics, noncovalent interactions, excited states, and transition elements: two new functionals and systematic testing of four M06-class functionals and 12 other functionals. *Theor. Chem. Acc.* **120**, 215-241 (2008).
13. Grimme, S., Antony, J., Ehrlich, S. & Krieg, H. A consistent and accurate *ab initio* parametrization of density functional dispersion correction (DFT-D) for the 94 elements H-Pu. *J. Chem. Phys.* **132**, 154104 (2010).
14. Weigend, F. & Ahlrichs, R. Balanced basis sets of split valence, triple zeta valence and quadruple zeta valence quality for H to Rn: Design and assessment of accuracy. *Phys. Chem. Chem. Phys.* **7**, 3297-3305 (2005).
15. Fukui, K. The path of chemical reactions-the IRC approach. *Acc. Chem. Res.* **14**, 363-368 (1981).
16. Marenich, A. V., Cramer, C. J. & Truhlar, D. G. Universal solvation model based on solute electron density and on a continuum model of the solvent defined by the bulk dielectric constant and atomic surface tensions. *J. Phys. Chem. B* **113**, 6378-6396 (2009).
17. Yang, Z. et al. Relationships between product ratios in ambimodal pericyclic reactions and bond lengths in transition structures. *J. Am. Chem. Soc.* **140**, 3061-3067 (2018).
