# Supplementary material for: Design of C1-symmetric tridentate ligands for enantioselective dearomative [3 + 2] annulation of indoles with aminocyclopropanes
Source: Nat Commun. 2023 Apr 20;14:2270. doi: 10.1038/s41467-023-38059-7 (PMC10119320; doi:10.1038/s41467-023-38059-7)
Supplement: Supplementary file 2 — Description of Additional Supplementary Files [file 41467_2023_38059_MOESM2_ESM.docx]

**Description of Additional Supplementary Files**

**File Name: Supplementary Data 1
Description:** Cartesian coordinate for theoretical calculation
