# Supplementary material for: Design of C1-symmetric tridentate ligands for enantioselective dearomative [3 + 2] annulation of indoles with aminocyclopropanes
Source: Nat Commun. 2023 Apr 20;14:2270. doi: 10.1038/s41467-023-38059-7 (PMC10119320; doi:10.1038/s41467-023-38059-7)
Supplement: Supplementary file 3 — Supplementary Data 1 [file 41467_2023_38059_MOESM3_ESM.pdf]

**Cartesian coordinate for theoretical calculation**

1. The main reaction pathway in Fig. 7

**RC**

| | | | |
|---|---|---|---|
| C | 2.12198100 | 0.84288200 | -2.01748100 |
| C | 2.80206600 | 1.28859100 | -3.14721600 |
| C | 3.89918700 | 0.54563100 | -3.58778500 |
| C | 4.30039400 | -0.59837200 | -2.89541500 |
| C | 3.57000200 | -0.97226000 | -1.77171800 |
| H | 2.49771800 | 2.20982600 | -3.64181300 |
| H | 4.45310300 | 0.86885500 | -4.47019300 |
| H | 5.17058900 | -1.17692800 | -3.20866900 |
| C | 0.96815000 | 1.57895500 | -1.34118200 |
| H | 0.44834700 | 2.21264600 | -2.07723200 |
| N | 2.51634000 | -0.25978300 | -1.38194900 |
| N | 1.45534300 | 2.36851800 | -0.21594700 |
| C | 3.94366200 | -2.13756300 | -0.86876500 |
| H | 4.35432200 | -2.94624500 | -1.50314700 |
| C | 4.66884900 | -2.35324700 | 1.35692100 |
| C | 3.22163900 | -4.44482900 | 1.51265600 |
| H | 4.07638100 | -4.77760200 | 2.11564500 |
| H | 2.30274100 | -4.73682600 | 2.04182600 |
| C | 0.82743600 | 1.98774500 | 1.04208000 |
| N | 2.77393200 | -2.64248000 | -0.08195300 |
| O | 5.41211700 | -2.40800100 | 2.29615000 |
| C | 3.25918500 | -2.92144900 | 1.30970400 |
| H | 2.65542300 | -2.38412800 | 2.05644300 |
| N | 4.91047000 | -1.75754600 | 0.14187400 |
| C | 6.16452100 | -1.15416000 | -0.19116200 |
| C | 6.23089300 | 0.22730200 | -0.38719800 |
| C | 7.30809900 | -1.94552800 | -0.30575000 |
| C | 7.44868100 | 0.81774600 | -0.71746400 |
| C | 8.52475400 | -1.34745000 | -0.62835100 |
| C | 8.59391000 | 0.03000900 | -0.83860700 |
| H | 7.50461200 | 1.89632400 | -0.86747100 |
| H | 9.42361200 | -1.95928800 | -0.70966700 |
| H | 9.54858200 | 0.49391900 | -1.08892300 |
| C | 3.19985000 | -4.98525200 | 0.08247300 |
| H | 4.21130100 | -4.98124900 | -0.35443700 |
| C | 2.31275600 | -3.95677700 | -0.60901400 |
| H | 1.26715900 | -4.10526700 | -0.29811500 |
| H | 2.34101200 | -3.95584200 | -1.70702600 |
| H | 2.80904300 | -6.00705400 | 0.00315600 |
| H | 7.24530700 | -3.01955900 | -0.12300400 |
| H | 5.32934200 | 0.83317100 | -0.27498800 |
| H | 0.61830400 | 2.89193600 | 1.63527100 |
| C | -0.47984200 | 1.36241100 | 0.52012700 |
| H | -0.89145800 | 0.62904700 | 1.22882800 |
| N | 0.05967500 | 0.63258500 | -0.64558000 |
| H | -0.69927600 | 0.29209400 | -1.24439400 |
| S | 2.17732600 | 3.86696900 | -0.46356000 |
| O | 3.26992000 | 3.94125000 | 0.48252500 |
| O | 2.38405500 | 3.89331400 | -1.90344900 |
| C | 0.97600700 | 5.07804200 | -0.02017300 |
| C | 0.99153400 | 5.61274400 | 1.26693200 |
| C | 0.04729200 | 5.49017800 | -0.97819900 |
| C | 0.03079200 | 6.56317700 | 1.60568100 |
| H | 1.76212800 | 5.30618500 | 1.97647000 |
| C | -0.89299800 | 6.44629700 | -0.62045400 |
| H | 0.08633800 | 5.08735500 | -1.99141500 |
| C | -0.92110500 | 6.99212900 | 0.67320100 |
| H | 0.03419900 | 6.99489600 | 2.60810800 |
| H | -1.62014100 | 6.78656600 | -1.36069300 |
| C | -1.94421200 | 8.03409800 | 1.02829900 |
| H | -1.96886700 | 8.22573700 | 2.10788800 |
| H | -1.71549400 | 8.98360300 | 0.52162500 |
| H | -2.94805900 | 7.72601300 | 0.70199500 |
| C | 1.67552500 | 1.03923800 | 1.88214400 |
| C | 1.11556300 | 0.37998500 | 2.98834500 |

| | | | |
|---|---|---|---|
| C | 3.06073200 | 0.96051500 | 1.67774300 |
| C | 1.92566400 | -0.33163700 | 3.87249900 |
| H | 0.04375900 | 0.46321100 | 3.19025900 |
| C | 3.87015200 | 0.25712600 | 2.56991800 |
| H | 3.50289300 | 1.52748900 | 0.85746300 |
| C | 3.30632600 | -0.39049000 | 3.66844800 |
| H | 1.48359800 | -0.81507100 | 4.74553900 |
| H | 4.95007200 | 0.22159200 | 2.41516900 |
| H | 3.94843800 | -0.93972600 | 4.35848600 |
| C | -1.53695300 | 2.40743700 | 0.19872600 |
| C | -2.10970800 | 2.54731600 | -1.06763400 |
| C | -1.96970600 | 3.25236700 | 1.23023300 |
| C | -3.08119200 | 3.52234000 | -1.30428400 |
| H | -1.82884400 | 1.88315900 | -1.88630400 |
| C | -2.94594300 | 4.21750400 | 0.99924200 |
| H | -1.54488200 | 3.15442500 | 2.23324400 |
| C | -3.50190300 | 4.35829300 | -0.27384800 |
| H | -3.51675500 | 3.61757400 | -2.30010400 |
| H | -3.27208200 | 4.86369900 | 1.81556300 |
| H | -4.26509600 | 5.11663200 | -0.45710000 |
| Ni | 1.24506400 | -1.09449500 | -0.07970000 |
| C | -4.65576800 | -0.90569100 | -2.41156900 |
| C | -5.59855100 | -2.11056800 | -2.41144500 |
| H | -5.13212900 | 0.00473300 | -2.01462300 |
| H | -4.23723300 | -0.65474400 | -3.39550500 |
| H | -5.59686800 | -2.66591300 | -3.36071200 |
| H | -6.63997700 | -1.85246800 | -2.18266600 |
| N | -3.82771800 | -2.49765700 | -0.90538900 |
| C | -5.06479800 | -3.03555900 | -1.33806100 |
| C | -3.53188900 | -1.27551100 | -1.47447000 |
| O | -2.50023300 | -0.66926400 | -1.25726700 |
| O | -5.51928200 | -4.05051400 | -0.90348800 |
| C | -3.05744200 | -3.12926400 | 0.10211700 |
| C | -2.44722300 | -4.44972600 | -0.08846300 |
| C | -1.52190800 | -3.22874200 | -0.07124500 |
| H | -3.34976800 | -2.86445800 | 1.11962900 |
| H | -2.55377900 | -4.92135000 | -1.06510500 |
| H | -2.38948300 | -5.10696700 | 0.77795600 |
| C | -0.80563900 | -2.88123400 | 1.18848500 |
| O | 0.11359400 | -2.07445900 | 1.29052600 |
| O | -1.28257900 | -3.49285800 | 2.23497700 |
| C | -0.90703900 | -2.80115900 | -1.36199700 |
| O | 0.12189700 | -2.14617000 | -1.47236100 |
| O | -1.55233900 | -3.23718800 | -2.40907400 |
| C | -0.71923000 | -3.17041800 | 3.51843800 |
| H | -0.83547000 | -2.09685600 | 3.70949800 |
| H | -1.27794400 | -3.76579000 | 4.24417300 |
| H | 0.34549200 | -3.43391600 | 3.53049700 |
| C | -1.08962600 | -2.81350200 | -3.69979900 |
| H | -0.05886700 | -3.15121900 | -3.85765200 |
| H | -1.76400300 | -3.27673200 | -4.42404300 |
| H | -1.13649700 | -1.71847400 | -3.75812600 |
| C | -5.45122100 | 0.01222200 | 0.84015000 |
| C | -6.66025500 | -0.64758600 | 0.49634600 |
| C | -7.60118700 | -0.07118900 | -0.37245500 |
| C | -7.30897000 | 1.18325700 | -0.88681800 |
| C | -6.11590800 | 1.86008800 | -0.54415800 |
| C | -5.18613900 | 1.28860300 | 0.31099200 |
| C | -4.75307400 | -0.84511400 | 1.75992700 |
| H | -8.53511100 | -0.57822000 | -0.62067100 |
| H | -8.02402300 | 1.66650100 | -1.55375600 |
| H | -5.93344500 | 2.85446400 | -0.95310600 |
| H | -4.27164900 | 1.82124200 | 0.57847600 |
| N | -6.69944700 | -1.84150600 | 1.17268700 |
| C | -5.55997800 | -1.94562500 | 1.93359100 |
| H | -5.41343400 | -2.81878400 | 2.56905400 |
| C | -3.40043100 | -0.59129400 | 2.34764600 |
| H | -2.61967500 | -0.64141300 | 1.56264900 |
| H | -3.33738200 | 0.40971800 | 2.80193300 |

| | | | |
|---|---|---|---|
| H | -3.16311500 | -1.33001200 | 3.12791700 |
| C | -7.76917600 | -2.81494900 | 1.09292000 |
| H | -7.61930500 | -3.57293300 | 1.86971000 |
| H | -8.73863300 | -2.32761400 | 1.26312700 |
| H | -7.77099900 | -3.31787500 | 0.11430700 |

**TS1**

| | | | |
|---|---|---|---|
| C | 2.71410300 | 1.03127500 | -1.86405800 |
| C | 3.62375100 | 1.59903100 | -2.75196400 |
| C | 4.73695900 | 0.84367200 | -3.12563400 |
| C | 4.92877100 | -0.43259900 | -2.59649400 |
| C | 3.97889400 | -0.92271300 | -1.70479700 |
| H | 3.47285100 | 2.61506300 | -3.11354800 |
| H | 5.46466000 | 1.25907000 | -3.82401500 |
| H | 5.80592600 | -1.02875000 | -2.85224100 |
| C | 1.48014200 | 1.75310800 | -1.32292900 |
| H | 1.11919900 | 2.46156600 | -2.08712400 |
| N | 2.90692200 | -0.19927600 | -1.38748200 |
| N | 1.72934200 | 2.44127100 | -0.06475200 |
| C | 4.13403900 | -2.24317000 | -0.96390300 |
| H | 4.57120100 | -2.98301200 | -1.66161300 |
| C | 4.51023600 | -2.81157400 | 1.29051500 |
| C | 2.93018200 | -4.79340700 | 0.90742400 |
| H | 3.68225300 | -5.29409600 | 1.53084100 |
| H | 1.93692600 | -5.07209400 | 1.28822100 |
| C | 0.87852500 | 1.95600800 | 1.01284200 |
| N | 2.83781600 | -2.74272600 | -0.42020900 |
| O | 5.09031200 | -3.01503000 | 2.32050100 |
| C | 3.09988500 | -3.26485000 | 0.96000700 |
| H | 2.42299400 | -2.80045900 | 1.69098100 |
| N | 4.97515800 | -2.09949200 | 0.20933500 |
| C | 6.29653800 | -1.55676900 | 0.14686600 |
| C | 6.47581600 | -0.17395800 | 0.22910900 |
| C | 7.39343700 | -2.40879000 | 0.01358300 |
| C | 7.76060800 | 0.36021500 | 0.16181700 |
| C | 8.67700600 | -1.86924800 | -0.04677000 |
| C | 8.85976000 | -0.48787400 | 0.02286700 |
| H | 7.90378300 | 1.43905100 | 0.22962100 |
| H | 9.53836000 | -2.53103200 | -0.14181200 |
| H | 9.86618100 | -0.07025700 | -0.02209500 |
| C | 3.02752500 | -5.11184100 | -0.58591400 |
| H | 4.07978400 | -5.15699100 | -0.90979700 |
| C | 2.32914000 | -3.90433400 | -1.20108100 |
| H | 1.24170900 | -3.96960600 | -1.04471600 |
| H | 2.50471700 | -3.74376900 | -2.27357300 |
| H | 2.55532100 | -6.06241200 | -0.86254800 |
| H | 7.23903800 | -3.48847800 | -0.02439600 |
| H | 5.60552200 | 0.47526500 | 0.34731000 |
| H | 0.55378600 | 2.80116700 | 1.63993300 |
| C | -0.30962900 | 1.39478000 | 0.19803000 |
| H | -0.84335700 | 0.60978700 | 0.75818900 |
| N | 0.44924300 | 0.77558700 | -0.90135300 |
| H | -0.13465300 | 0.49543000 | -1.68910000 |
| S | 2.47628100 | 3.94383300 | -0.01898400 |
| O | 3.35089600 | 3.91808500 | 1.13150500 |
| O | 2.96294900 | 4.11131200 | -1.37952400 |
| C | 1.19051000 | 5.11410000 | 0.28560200 |
| C | 0.93764600 | 5.52307400 | 1.59369500 |
| C | 0.46220700 | 5.61482900 | -0.79530600 |
| C | -0.09117600 | 6.43476100 | 1.82106800 |
| H | 1.55784200 | 5.15018500 | 2.41089700 |
| C | -0.55128700 | 6.53036700 | -0.54548600 |
| H | 0.71315300 | 5.31293000 | -1.81344200 |
| C | -0.84737400 | 6.94986800 | 0.76141400 |
| H | -0.29574400 | 6.76859400 | 2.83994600 |
| H | -1.12147900 | 6.94104300 | -1.38146600 |
| C | -1.93523200 | 7.95878900 | 1.00236800 |
| H | -2.82155100 | 7.73902800 | 0.39042000 |
| H | -2.23261700 | 7.98834400 | 2.05785300 |

| | | | |
|---|---|---|---|
| H | -1.58997200 | 8.96586900 | 0.72351700 |
| C | 1.56119000 | 0.91080000 | 1.88561500 |
| C | 0.84222900 | 0.26790200 | 2.90196300 |
| C | 2.93315700 | 0.65724800 | 1.75890000 |
| C | 1.48359300 | -0.61546000 | 3.76795200 |
| H | -0.22284800 | 0.48148300 | 3.03513500 |
| C | 3.57286800 | -0.23141700 | 2.62655200 |
| H | 3.50868700 | 1.22493800 | 1.02621400 |
| C | 2.84894700 | -0.87553100 | 3.62781500 |
| H | 0.92340500 | -1.08668700 | 4.57731200 |
| H | 4.64747600 | -0.40272900 | 2.54217000 |
| H | 3.35849700 | -1.56503000 | 4.30264300 |
| C | -1.30202900 | 2.46215100 | -0.24045900 |
| C | -1.59496300 | 2.71978300 | -1.58343100 |
| C | -1.97599500 | 3.19805100 | 0.74375200 |
| C | -2.52349300 | 3.70321200 | -1.93963000 |
| H | -1.09509500 | 2.17184200 | -2.38551400 |
| C | -2.90198400 | 4.17598300 | 0.39347700 |
| H | -1.78248900 | 2.99649000 | 1.80026100 |
| C | -3.17749900 | 4.43461400 | -0.95226600 |
| H | -2.72644500 | 3.89928900 | -2.99365900 |
| H | -3.41054300 | 4.74230000 | 1.17527500 |
| H | -3.89840200 | 5.20621700 | -1.22710000 |
| Ni | 1.42301100 | -1.12566400 | -0.37778500 |
| C | -4.76870800 | 1.04364900 | -0.74833500 |
| C | -5.00296100 | 0.33466200 | -2.08237200 |
| H | -5.71084700 | 1.21981300 | -0.20251800 |
| H | -4.24043500 | 2.00311600 | -0.81612800 |
| H | -4.37892800 | 0.74109800 | -2.89353200 |
| H | -6.04502600 | 0.36447800 | -2.42695900 |
| N | -3.96921100 | -1.18550500 | -0.59176900 |
| C | -4.59909700 | -1.11119600 | -1.87625600 |
| C | -3.96499100 | 0.08727100 | 0.08239400 |
| O | -3.37026200 | 0.24911100 | 1.10634600 |
| O | -4.73377000 | -2.04454300 | -2.60571400 |
| C | -3.48799100 | -2.26037800 | 0.05212400 |
| C | -2.89929600 | -3.46775800 | -0.55823000 |
| C | -1.51298100 | -2.85137800 | -0.54279900 |
| H | -3.22841600 | -2.05028700 | 1.08984200 |
| H | -3.25730300 | -3.67948200 | -1.56796400 |
| H | -2.96512500 | -4.33371500 | 0.10714200 |
| C | -0.79975700 | -2.75988100 | 0.69272100 |
| O | 0.25099200 | -2.12400000 | 0.90615600 |
| O | -1.38271700 | -3.39947400 | 1.69763600 |
| C | -0.97329800 | -2.26554900 | -1.73199800 |
| O | 0.17303200 | -1.79748000 | -1.87950000 |
| O | -1.81735500 | -2.25820000 | -2.75132900 |
| C | -0.79563800 | -3.28283000 | 2.99499700 |
| H | -0.83165200 | -2.23828900 | 3.32825200 |
| H | -1.39208900 | -3.92160800 | 3.65212100 |
| H | 0.24888100 | -3.61828500 | 2.97606100 |
| C | -1.35775600 | -1.79722200 | -4.01920200 |
| H | -0.50454600 | -2.39731200 | -4.35816800 |
| H | -2.20555700 | -1.91360000 | -4.69990400 |
| H | -1.05453700 | -0.74285400 | -3.96176700 |
| C | -6.34946200 | -1.39066400 | 1.42961900 |
| C | -7.02652100 | -1.52902400 | 0.19060200 |
| C | -7.91317900 | -0.55363500 | -0.29196700 |
| C | -8.10327200 | 0.57176400 | 0.49694500 |
| C | -7.42935400 | 0.73507700 | 1.73071900 |
| C | -6.55481100 | -0.22940000 | 2.20151700 |
| C | -5.58361700 | -2.59025900 | 1.62854400 |
| H | -8.45680600 | -0.68730100 | -1.22842500 |
| H | -8.80241300 | 1.34174600 | 0.16761600 |
| H | -7.61917500 | 1.62985300 | 2.32401800 |
| H | -6.04887600 | -0.10225700 | 3.16008600 |
| N | -6.69349000 | -2.75431600 | -0.34650900 |
| C | -5.83749700 | -3.38213900 | 0.51219800 |
| H | -5.51341900 | -4.40267900 | 0.31108000 |

| | | | |
|---|---|---|---|
| C | -4.78781300 | -2.95383500 | 2.84212500 |
| H | -4.13969100 | -2.11944900 | 3.15520500 |
| H | -5.44974700 | -3.18449900 | 3.68987500 |
| H | -4.15828200 | -3.83581900 | 2.65797300 |
| C | -7.27541200 | -3.31966800 | -1.54883900 |
| H | -6.80905600 | -4.29104100 | -1.74684200 |
| H | -8.35741500 | -3.46042400 | -1.41796300 |
| H | -7.08703800 | -2.66669100 | -2.41150700 |

**IM1**

| | | | |
|---|---|---|---|
| C | 3.00402500 | 0.55495700 | -1.90574400 |
| C | 4.05134200 | 0.83331500 | -2.78027300 |
| C | 4.91266500 | -0.20999800 | -3.12531100 |
| C | 4.73310600 | -1.47997600 | -2.57669700 |
| C | 3.67466600 | -1.66896600 | -1.69196700 |
| H | 4.19427800 | 1.84648700 | -3.15444800 |
| H | 5.73669100 | -0.02704000 | -3.81624100 |
| H | 5.41224700 | -2.30122100 | -2.80994400 |
| C | 2.01497800 | 1.60565900 | -1.39741300 |
| H | 1.85661200 | 2.35227100 | -2.19237300 |
| N | 2.83969900 | -0.67157500 | -1.40949000 |
| N | 2.46662700 | 2.25648200 | -0.17635300 |
| C | 3.45480100 | -2.96296100 | -0.91784900 |
| H | 3.64212000 | -3.81411600 | -1.60007100 |
| C | 3.70347200 | -3.51238200 | 1.36256800 |
| C | 1.56395000 | -4.89773500 | 1.16931600 |
| H | 2.11291000 | -5.54013000 | 1.87008200 |
| H | 0.52838200 | -4.80406000 | 1.52903900 |
| C | 1.57163100 | 2.01573300 | 0.94669800 |
| N | 2.08345800 | -3.05323700 | -0.34118700 |
| O | 4.22460000 | -3.84169600 | 2.39213300 |
| C | 2.21214800 | -3.50746700 | 1.08083200 |
| H | 1.74524500 | -2.78880700 | 1.77035700 |
| N | 4.33430900 | -3.04316200 | 0.23461600 |
| C | 5.75595800 | -2.94499300 | 0.12562900 |
| C | 6.36491800 | -1.68839100 | 0.16269700 |
| C | 6.52413400 | -4.10203200 | -0.00994600 |
| C | 7.74980800 | -1.58932300 | 0.04897800 |
| C | 7.90964700 | -3.99745900 | -0.11804800 |
| C | 8.52074900 | -2.74360000 | -0.09255800 |
| H | 8.22794100 | -0.60982700 | 0.08122900 |
| H | 8.51474900 | -4.89929000 | -0.21559400 |
| H | 9.60544100 | -2.66609700 | -0.17472900 |
| C | 1.57265900 | -5.37931400 | -0.28247900 |
| H | 2.56983900 | -5.75576600 | -0.56376700 |
| C | 1.25759300 | -4.08644400 | -1.02627200 |
| H | 0.19821900 | -3.82032300 | -0.89412300 |
| H | 1.47360700 | -4.08770300 | -2.10315600 |
| H | 0.84614900 | -6.17563500 | -0.48560000 |
| H | 6.03536100 | -5.07784300 | -0.01311900 |
| H | 5.74715200 | -0.79538100 | 0.27919900 |
| H | 1.48316000 | 2.93100700 | 1.55341000 |
| C | 0.24573300 | 1.74499300 | 0.19793500 |
| H | -0.42829800 | 1.10727800 | 0.79242900 |
| N | 0.76700800 | 0.96209600 | -0.93232500 |
| H | 0.09285800 | 0.84475300 | -1.68943400 |
| S | 3.55129600 | 3.53571200 | -0.21794100 |
| O | 4.50028700 | 3.30498600 | 0.84796900 |
| O | 3.94378700 | 3.59466600 | -1.61848500 |
| C | 2.59674300 | 4.96441100 | 0.18760800 |
| C | 2.56693100 | 5.41077600 | 1.50725400 |
| C | 1.87406700 | 5.60328400 | -0.82111800 |
| C | 1.76566200 | 6.50595700 | 1.82354200 |
| H | 3.17699200 | 4.91365900 | 2.26353700 |
| C | 1.08845300 | 6.69729800 | -0.48422200 |
| H | 1.94993200 | 5.25716500 | -1.85320700 |
| C | 1.01508500 | 7.15992000 | 0.83936000 |
| H | 1.73488000 | 6.86804600 | 2.85270300 |
| H | 0.52369800 | 7.21331300 | -1.26351900 |

| | | | |
|---|---|---|---|
| C | 0.16748300 | 8.35540700 | 1.17395200 |
| H | 0.01118100 | 8.44957400 | 2.25557200 |
| H | 0.65227800 | 9.27868900 | 0.82252000 |
| H | -0.81234400 | 8.29352700 | 0.67935400 |
| C | 2.01696600 | 0.86337800 | 1.83715500 |
| C | 1.18804200 | 0.43399300 | 2.88103100 |
| C | 3.28846200 | 0.29250000 | 1.69964400 |
| C | 1.61870200 | -0.55246300 | 3.76534600 |
| H | 0.20262900 | 0.88972000 | 3.01526400 |
| C | 3.71595400 | -0.70024500 | 2.58399000 |
| H | 3.96302200 | 0.68199200 | 0.93466000 |
| C | 2.88173800 | -1.12864900 | 3.61508600 |
| H | 0.97442900 | -0.86204000 | 4.58991700 |
| H | 4.71247000 | -1.13435500 | 2.48439800 |
| H | 3.22529600 | -1.90792100 | 4.29679700 |
| C | -0.49967000 | 3.00698500 | -0.19823500 |
| C | -0.67812900 | 3.40392300 | -1.52698300 |
| C | -1.08433500 | 3.77598700 | 0.81589800 |
| C | -1.43135300 | 4.53908000 | -1.83896600 |
| H | -0.24202900 | 2.83177400 | -2.34827600 |
| C | -1.83045400 | 4.91024500 | 0.50918100 |
| H | -0.96772800 | 3.47111000 | 1.85916000 |
| C | -2.01166500 | 5.29264200 | -0.82244200 |
| H | -1.55941100 | 4.83330100 | -2.88182500 |
| H | -2.27771400 | 5.49718000 | 1.31294100 |
| H | -2.59974800 | 6.17940900 | -1.06394000 |
| Ni | 1.13819100 | -1.11480400 | -0.39815500 |
| C | -3.89692400 | 1.85939300 | -0.85099200 |
| C | -4.55346300 | 1.21428600 | -2.07209300 |
| H | -4.57740000 | 2.54628300 | -0.32482300 |
| H | -2.97494300 | 2.41647100 | -1.05338500 |
| H | -3.86051600 | 1.11079400 | -2.92044400 |
| H | -5.44877800 | 1.72681500 | -2.44396200 |
| N | -4.34120000 | -0.39760100 | -0.37903800 |
| C | -4.91631600 | -0.18302500 | -1.61539900 |
| C | -3.62040700 | 0.70559900 | 0.09156300 |
| O | -2.94046800 | 0.69031300 | 1.08394300 |
| O | -5.58649100 | -1.00616200 | -2.19602900 |
| C | -4.39904300 | -1.65798000 | 0.33819500 |
| C | -3.49487000 | -2.70568000 | -0.33120700 |
| C | -2.06669300 | -2.22738600 | -0.39594300 |
| H | -3.97859200 | -1.43309800 | 1.32858900 |
| H | -3.85568600 | -2.91104500 | -1.34749000 |
| H | -3.54449200 | -3.63475800 | 0.25260400 |
| C | -1.34691900 | -2.08420500 | 0.81471200 |
| O | -0.20950000 | -1.58331400 | 0.98353500 |
| O | -1.99783300 | -2.52741200 | 1.89308000 |
| C | -1.48771900 | -1.79713000 | -1.61165800 |
| O | -0.29121700 | -1.47172100 | -1.81881000 |
| O | -2.33636400 | -1.75074400 | -2.64745100 |
| C | -1.38935600 | -2.31047900 | 3.16118600 |
| H | -1.30167400 | -1.23350800 | 3.35628700 |
| H | -2.04700200 | -2.78278400 | 3.89710100 |
| H | -0.38954400 | -2.76261500 | 3.19826600 |
| C | -1.80211000 | -1.46196600 | -3.93216800 |
| H | -1.01405100 | -2.17805200 | -4.19713100 |
| H | -2.63985000 | -1.54316800 | -4.63116900 |
| H | -1.38123900 | -0.44736200 | -3.96324200 |
| C | -6.77005400 | -0.99398800 | 1.05064100 |
| C | -7.91931000 | -1.01601700 | 0.26152000 |
| C | -8.98188300 | -0.14013300 | 0.42510800 |
| C | -8.84987500 | 0.80688800 | 1.44403100 |
| C | -7.70259800 | 0.85511000 | 2.24267600 |
| C | -6.64805800 | -0.04629400 | 2.05731600 |
| C | -5.85787400 | -2.11564500 | 0.60633100 |
| H | -9.87364400 | -0.17656000 | -0.20101800 |
| H | -9.65644600 | 1.51967400 | 1.61709300 |
| H | -7.63228300 | 1.60844400 | 3.02787600 |
| H | -5.75599300 | 0.00312000 | 2.68547500 |

| | | | |
|---|---|---|---|
| N | -7.77147700 | -2.06121000 | -0.70349300 |
| C | -6.61336400 | -2.62227500 | -0.58782800 |
| H | -6.30688800 | -3.42882100 | -1.25595000 |
| C | -5.86083100 | -3.25233400 | 1.66311400 |
| H | -5.35150800 | -2.89011400 | 2.56644000 |
| H | -6.88931400 | -3.52669900 | 1.93263400 |
| H | -5.33833400 | -4.14583200 | 1.29951300 |
| C | -8.74889500 | -2.30891600 | -1.75492600 |
| H | -8.42750700 | -3.16983900 | -2.34925800 |
| H | -9.72715700 | -2.50894600 | -1.30121000 |
| H | -8.80375900 | -1.41703700 | -2.39293200 |

**IM2**

| | | | |
|---|---|---|---|
| C | 2.10198200 | 0.79879900 | -1.92916000 |
| C | 2.89286400 | 1.22917000 | -2.99136500 |
| C | 4.04490500 | 0.49766400 | -3.28302900 |
| C | 4.38144300 | -0.62737100 | -2.52910800 |
| C | 3.53304900 | -0.99525300 | -1.48471600 |
| H | 2.62106000 | 2.12619200 | -3.54865800 |
| H | 4.68918500 | 0.80826300 | -4.10671400 |
| H | 5.27945900 | -1.20387400 | -2.75143500 |
| C | 0.88120900 | 1.58399500 | -1.43609900 |
| H | 0.29703800 | 1.92219400 | -2.30630800 |
| N | 2.43712900 | -0.28213200 | -1.22595700 |
| N | 1.30055600 | 2.73011200 | -0.64788000 |
| C | 3.77103000 | -2.21321100 | -0.58172500 |
| H | 3.68993200 | -3.12168200 | -1.19588400 |
| C | 4.95291800 | -1.72404800 | 1.38427700 |
| C | 3.10902000 | -2.55390600 | 2.89716100 |
| H | 3.85921200 | -2.44746200 | 3.69148900 |
| H | 2.12319500 | -2.26242600 | 3.28890400 |
| C | 1.01362700 | 2.55440000 | 0.76281400 |
| N | 2.78892700 | -2.27803900 | 0.52185100 |
| O | 5.84268400 | -1.46502000 | 2.14861000 |
| C | 3.46246800 | -1.67287500 | 1.70741300 |
| H | 3.17070600 | -0.62156200 | 1.84478300 |
| N | 5.06640600 | -2.14494800 | 0.07855000 |
| C | 6.29765400 | -2.32452000 | -0.61307400 |
| C | 7.34550200 | -1.41900100 | -0.42921800 |
| C | 6.44285500 | -3.40387600 | -1.49036900 |
| C | 8.53578400 | -1.59841200 | -1.13168100 |
| C | 7.63061300 | -3.56345100 | -2.20005900 |
| C | 8.67991500 | -2.66130000 | -2.02232200 |
| H | 9.35675600 | -0.89645600 | -0.98174100 |
| H | 7.74175000 | -4.40577600 | -2.88373000 |
| H | 9.61212200 | -2.79228200 | -2.57237100 |
| C | 3.06284700 | -3.94204800 | 2.25737500 |
| H | 4.08441900 | -4.31902400 | 2.09908000 |
| C | 2.37475800 | -3.66378000 | 0.92130100 |
| H | 1.28086700 | -3.67258700 | 1.03951700 |
| H | 2.63325900 | -4.37922700 | 0.13008300 |
| H | 2.52026400 | -4.68347400 | 2.85632900 |
| H | 5.63556700 | -4.12842600 | -1.61318100 |
| H | 7.23132200 | -0.59422200 | 0.27245000 |
| H | 0.77136300 | 3.53425100 | 1.20405200 |
| C | -0.26836100 | 1.70139100 | 0.66225900 |
| H | -0.41294600 | 1.08362400 | 1.56215100 |
| N | 0.07386900 | 0.80151500 | -0.45905000 |
| H | -0.77577700 | 0.40929800 | -0.87518300 |
| S | 1.70692100 | 4.19761000 | -1.35772600 |
| O | 3.00729300 | 4.57002300 | -0.84529300 |
| O | 1.47543600 | 3.95748000 | -2.77614400 |
| C | 0.52272600 | 5.35448300 | -0.74978900 |
| C | 0.83197000 | 6.11273700 | 0.37823300 |
| C | -0.69336800 | 5.49290500 | -1.41959600 |
| C | -0.12043600 | 7.00671100 | 0.86204800 |
| H | 1.81084100 | 6.01373800 | 0.85085600 |
| C | -1.62182200 | 6.39999800 | -0.92667800 |
| H | -0.88866600 | 4.91424400 | -2.32354800 |

| | | | |
|---|---|---|---|
| C | -1.35575700 | 7.16305900 | 0.22139200 |
| H | 0.10850700 | 7.60832600 | 1.74346900 |
| H | -2.57392000 | 6.52918300 | -1.44560000 |
| C | -2.36962900 | 8.15253000 | 0.72373300 |
| H | -2.16819000 | 8.44509900 | 1.76158600 |
| H | -2.34840400 | 9.06513400 | 0.10887300 |
| H | -3.38737800 | 7.74229100 | 0.66280100 |
| C | 2.14786300 | 1.93823900 | 1.57261200 |
| C | 1.90518400 | 1.47692800 | 2.87424800 |
| C | 3.46786000 | 1.98927400 | 1.10404000 |
| C | 2.96300300 | 1.09343500 | 3.70064600 |
| H | 0.88652800 | 1.45773400 | 3.27032000 |
| C | 4.52176800 | 1.59815800 | 1.93020600 |
| H | 3.66748700 | 2.39147200 | 0.10879300 |
| C | 4.27543600 | 1.15639800 | 3.23142900 |
| H | 2.76161300 | 0.75459200 | 4.71795800 |
| H | 5.54712900 | 1.65190500 | 1.56173200 |
| H | 5.10588000 | 0.84878800 | 3.86743100 |
| C | -1.49924600 | 2.56455300 | 0.43906200 |
| C | -2.27563700 | 2.50858100 | -0.71859500 |
| C | -1.89332300 | 3.42567100 | 1.47213600 |
| C | -3.43857600 | 3.27511100 | -0.83230000 |
| H | -1.99939600 | 1.85306500 | -1.54581800 |
| C | -3.05039200 | 4.19062000 | 1.36296300 |
| H | -1.29327600 | 3.48907100 | 2.38484300 |
| C | -3.83535900 | 4.10863800 | 0.20965200 |
| H | -4.03379000 | 3.22409800 | -1.74560600 |
| H | -3.34201100 | 4.85314600 | 2.17931100 |
| H | -4.74462600 | 4.70524300 | 0.12156400 |
| Ni | 1.16183100 | -0.95689700 | 0.17367700 |
| C | -4.54942000 | -0.15246300 | 2.80948800 |
| C | -3.66065800 | 0.43882100 | 1.71935300 |
| H | -5.53947900 | 0.32155100 | 2.86442300 |
| H | -4.12004300 | -0.11628700 | 3.81861900 |
| H | -2.63902600 | 0.64611700 | 2.07232700 |
| H | -4.03002900 | 1.36874100 | 1.26489300 |
| N | -4.16414100 | -1.77403900 | 1.12922500 |
| C | -3.54455200 | -0.64215900 | 0.66384400 |
| C | -4.74314600 | -1.59810800 | 2.40865000 |
| O | -5.28779100 | -2.47753800 | 3.00470000 |
| O | -2.98404200 | -0.54118000 | -0.41511600 |
| C | -4.09473200 | -3.11176700 | 0.56179300 |
| C | -2.73654400 | -3.77645700 | 0.88834100 |
| C | -1.50130000 | -2.94026700 | 0.67691400 |
| H | -4.84413800 | -3.67705400 | 1.13589600 |
| H | -2.65802600 | -4.72324700 | 0.34095100 |
| H | -2.79479800 | -4.03657600 | 1.95305900 |
| C | -1.03053100 | -2.10172900 | 1.71709900 |
| O | -0.00086400 | -1.37662700 | 1.72859700 |
| O | -1.79083900 | -2.09565900 | 2.81973200 |
| C | -0.84828000 | -2.94157900 | -0.57617700 |
| O | 0.13509000 | -2.26246900 | -0.94805400 |
| O | -1.38663300 | -3.78270600 | -1.48122400 |
| C | -1.29845200 | -1.41715600 | 3.96843300 |
| H | -1.23013700 | -0.33474900 | 3.78478600 |
| H | -2.01571200 | -1.62384500 | 4.76882300 |
| H | -0.30527300 | -1.79116300 | 4.24734800 |
| C | -0.67941600 | -3.96596900 | -2.70419700 |
| H | -0.60719900 | -3.02326800 | -3.26350100 |
| H | 0.33535400 | -4.33562700 | -2.51040800 |
| H | -1.24555700 | -4.71048000 | -3.27285300 |
| C | -6.06942500 | -2.59825700 | -0.91786400 |
| C | -6.14212500 | -1.63192200 | -1.91868500 |
| C | -7.29684600 | -0.93364400 | -2.23760600 |
| C | -8.43040600 | -1.24060100 | -1.48048300 |
| C | -8.38091700 | -2.19741600 | -0.46181700 |
| C | -7.20003300 | -2.88858600 | -0.16664800 |
| C | -4.66115100 | -3.16118100 | -0.88366600 |
| H | -7.33207400 | -0.18585300 | -3.03024500 |

| | | | |
|---|---|---|---|
| H | -9.36766600 | -0.72393200 | -1.68823400 |
| H | -9.28245900 | -2.41083200 | 0.11314500 |
| H | -7.18022700 | -3.62771200 | 0.63680500 |
| N | -4.84402400 | -1.51869300 | -2.50891300 |
| C | -3.99915300 | -2.29928100 | -1.91779400 |
| H | -2.96111900 | -2.36807300 | -2.23656400 |
| C | -4.63620300 | -4.61413700 | -1.41952300 |
| H | -3.60419100 | -4.95773400 | -1.56188200 |
| H | -5.13895600 | -5.26838700 | -0.69352200 |
| H | -5.17866400 | -4.68191300 | -2.37196100 |
| C | -4.54230800 | -0.56557300 | -3.56521500 |
| H | -3.49781800 | -0.68511600 | -3.86974100 |
| H | -5.20579500 | -0.74685600 | -4.41995900 |
| H | -4.70627700 | 0.44851300 | -3.17750900 |

**TS2**

| | | | |
|---|---|---|---|
| C | 1.30590800 | 0.92028700 | -1.70551200 |
| C | 1.89519900 | 1.57093900 | -2.78805500 |
| C | 3.25037900 | 1.34037700 | -3.02541900 |
| C | 3.98823300 | 0.49351300 | -2.19235700 |
| C | 3.31751800 | -0.14259700 | -1.15074600 |
| H | 1.31889500 | 2.26418100 | -3.40319900 |
| H | 3.74559200 | 1.84339100 | -3.85725700 |
| H | 5.05551400 | 0.33726700 | -2.35156200 |
| C | -0.12746400 | 1.21415100 | -1.23978200 |
| H | -0.81830100 | 1.08362200 | -2.08523300 |
| N | 2.01322600 | 0.07029500 | -0.96507700 |
| N | -0.24245800 | 2.56885300 | -0.74866300 |
| C | 3.97126400 | -1.08238300 | -0.12723100 |
| H | 4.09594000 | -2.08031000 | -0.56892000 |
| C | 5.02351100 | 0.20309100 | 1.50092900 |
| C | 3.49562800 | -0.52046400 | 3.36621500 |
| H | 4.11320400 | 0.11038100 | 4.01843000 |
| H | 2.45399000 | -0.48577000 | 3.72291700 |
| C | -0.18154300 | 2.62220900 | 0.69812500 |
| N | 3.13223600 | -1.18954500 | 1.08847800 |
| O | 5.81892500 | 0.88374000 | 2.08834200 |
| C | 3.57286000 | -0.03917400 | 1.92982600 |
| H | 2.97804000 | 0.85962700 | 1.71566100 |
| N | 5.22888000 | -0.53598600 | 0.35184600 |
| C | 6.44184800 | -0.60640800 | -0.39056000 |
| C | 7.33414600 | 0.47131600 | -0.39701900 |
| C | 6.72997000 | -1.75932500 | -1.13011800 |
| C | 8.50757100 | 0.38279000 | -1.14399100 |
| C | 7.90026900 | -1.82874700 | -1.88191100 |
| C | 8.79378900 | -0.75855000 | -1.89135500 |
| H | 9.20479000 | 1.22139000 | -1.13992200 |
| H | 8.11930700 | -2.73081000 | -2.45423800 |
| H | 9.71260500 | -0.81691700 | -2.47512800 |
| C | 4.00774400 | -1.95586000 | 3.23112800 |
| H | 5.10563600 | -1.95044300 | 3.17094400 |
| C | 3.39606000 | -2.42757500 | 1.90767800 |
| H | 2.43532100 | -2.93334900 | 2.07345000 |
| H | 4.05059600 | -3.11449800 | 1.35437700 |
| H | 3.72344800 | -2.60423200 | 4.06836600 |
| H | 6.05380800 | -2.61555300 | -1.11428100 |
| H | 7.11961700 | 1.35925700 | 0.19367600 |
| H | -0.79531800 | 3.46718100 | 1.04892200 |
| C | -0.92414700 | 1.29960900 | 1.03931600 |
| H | -0.57160900 | 0.88158400 | 1.99323400 |
| N | -0.52048900 | 0.38404100 | -0.07570800 |
| H | -1.32871200 | -0.20995800 | -0.29877000 |
| S | -0.43611900 | 3.84113100 | -1.83576700 |
| O | 0.83073600 | 4.53224000 | -1.98427800 |
| O | -1.03650900 | 3.18638800 | -2.99469500 |
| C | -1.57478200 | 4.90565400 | -1.02874500 |
| C | -1.07701900 | 5.95270400 | -0.25161600 |
| C | -2.94232900 | 4.68250600 | -1.18108600 |
| C | -1.98349800 | 6.77431000 | 0.40964700 |

| | | | |
|---|---|---|---|
| H | -0.00087200 | 6.12051500 | -0.18228700 |
| C | -3.82855500 | 5.51879800 | -0.51305700 |
| H | -3.29567400 | 3.87876700 | -1.82760100 |
| C | -3.36657400 | 6.57174800 | 0.29140300 |
| H | -1.61187000 | 7.59844300 | 1.02110200 |
| H | -4.90320300 | 5.36222800 | -0.62450700 |
| C | -4.33550600 | 7.49338700 | 0.97629300 |
| H | -3.91466900 | 7.89802300 | 1.90536500 |
| H | -4.57067200 | 8.34602800 | 0.32070100 |
| H | -5.27913300 | 6.98303700 | 1.20712100 |
| C | 1.20748300 | 2.76027700 | 1.30670000 |
| C | 1.35162700 | 2.64944100 | 2.69713600 |
| C | 2.31785100 | 3.12459000 | 0.53585100 |
| C | 2.58295100 | 2.89040000 | 3.30624800 |
| H | 0.48493100 | 2.41052700 | 3.31971800 |
| C | 3.55307400 | 3.35191700 | 1.14706000 |
| H | 2.20440300 | 3.27904200 | -0.53856200 |
| C | 3.69109000 | 3.23527700 | 2.53052600 |
| H | 2.67609300 | 2.81674100 | 4.39082800 |
| H | 4.41143500 | 3.63899500 | 0.53756500 |
| H | 4.65949100 | 3.40910000 | 2.99994200 |
| C | -2.41509500 | 1.55420900 | 1.13062100 |
| C | -3.23596300 | 1.57612600 | 0.00092100 |
| C | -2.96420000 | 1.88086200 | 2.37468100 |
| C | -4.58253000 | 1.91938000 | 0.11291500 |
| H | -2.83365600 | 1.32581300 | -0.98355700 |
| C | -4.30859300 | 2.23654200 | 2.48804800 |
| H | -2.33077500 | 1.87490100 | 3.26650100 |
| C | -5.12185100 | 2.25666400 | 1.35467000 |
| H | -5.21134600 | 1.93002100 | -0.77997700 |
| H | -4.72024400 | 2.50128300 | 3.46296100 |
| H | -6.17297600 | 2.53591500 | 1.44018000 |
| Ni | 1.10121500 | -0.96842900 | 0.47917200 |
| C | -5.84842100 | -2.47762400 | 2.02212700 |
| C | -4.96749500 | -1.31084200 | 1.58147400 |
| H | -6.90852300 | -2.38187900 | 1.75804400 |
| H | -5.80392700 | -2.66709800 | 3.10461100 |
| H | -4.62571300 | -0.63894200 | 2.38054200 |
| H | -5.44972600 | -0.67136200 | 0.82530500 |
| N | -4.02733100 | -3.29349100 | 0.74612400 |
| C | -3.78118900 | -1.95060700 | 0.90066100 |
| C | -5.26493200 | -3.68564800 | 1.31708200 |
| O | -5.70938900 | -4.78897500 | 1.22405500 |
| O | -2.77178400 | -1.38875600 | 0.52169500 |
| C | -3.25331100 | -4.24296200 | -0.04348300 |
| C | -1.91685300 | -4.72255700 | 0.56739900 |
| C | -0.88649400 | -3.61239500 | 0.46825800 |
| H | -3.92366600 | -5.11002500 | -0.12484200 |
| H | -1.58173800 | -5.58038100 | -0.03097400 |
| H | -2.06429200 | -5.08156800 | 1.59247300 |
| C | -0.89917600 | -2.68185400 | 1.62478900 |
| O | -0.09846100 | -1.76311500 | 1.85163600 |
| O | -1.79400400 | -2.97499100 | 2.53673900 |
| C | 0.47697900 | -3.84620200 | -0.00142200 |
| O | 1.29926800 | -2.93307000 | -0.18786400 |
| O | 0.74836200 | -5.07455200 | -0.36982900 |
| C | -1.93066500 | -2.07562300 | 3.63943200 |
| H | -2.17371600 | -1.07217100 | 3.25890400 |
| H | -2.74539000 | -2.47458600 | 4.25048300 |
| H | -1.00060400 | -2.03762500 | 4.21870400 |
| C | 2.02141600 | -5.34008100 | -0.97176700 |
| H | 2.14576400 | -4.73174200 | -1.87686800 |
| H | 2.82373700 | -5.11438100 | -0.25919100 |
| H | 2.01804000 | -6.40466400 | -1.21797900 |
| C | -3.61097400 | -2.46476400 | -1.95618800 |
| C | -2.64820400 | -1.55663800 | -2.41039600 |
| C | -2.99337300 | -0.36675000 | -3.03721400 |
| C | -4.35962800 | -0.07686000 | -3.15466000 |
| C | -5.32919200 | -0.95445700 | -2.67147900 |

| | | | |
|---|---|---|---|
| C | -4.95914100 | -2.17060600 | -2.07852400 |
| C | -2.90911600 | -3.70978400 | -1.45608100 |
| H | -2.25563000 | 0.32232200 | -3.45335300 |
| H | -4.66336800 | 0.84562200 | -3.65165600 |
| H | -6.38541300 | -0.71111200 | -2.79050300 |
| H | -5.72243200 | -2.87866600 | -1.74545100 |
| N | -1.35138700 | -2.07996200 | -2.15777600 |
| C | -1.44637500 | -3.26873500 | -1.51034100 |
| H | -0.72773100 | -4.02412900 | -1.84300000 |
| C | -3.09499300 | -4.84295900 | -2.48885500 |
| H | -2.56893300 | -5.75589200 | -2.17212600 |
| H | -4.16361600 | -5.07361800 | -2.59304800 |
| H | -2.71641200 | -4.53872000 | -3.47483500 |
| C | -0.21400100 | -1.75235000 | -3.00825000 |
| H | 0.71664300 | -2.05850800 | -2.51448700 |
| H | -0.30301000 | -2.25649500 | -3.98383500 |
| H | -0.17310600 | -0.67274500 | -3.18906300 |

**PC1**

| | | | |
|---|---|---|---|
| C | -0.65149700 | -1.85180100 | -1.78326800 |
| C | -1.01673400 | -2.90307400 | -2.62211600 |
| C | -2.37391000 | -3.10090300 | -2.87016200 |
| C | -3.32799100 | -2.27534400 | -2.27180600 |
| C | -2.88026800 | -1.23846200 | -1.45712400 |
| H | -0.25331400 | -3.55627600 | -3.04793000 |
| H | -2.69360600 | -3.91550600 | -3.52152200 |
| H | -4.39381800 | -2.43868500 | -2.43273800 |
| C | 0.79872800 | -1.63213400 | -1.34496200 |
| H | 1.43526500 | -1.59004700 | -2.24310500 |
| N | -1.57216900 | -1.03668400 | -1.26487700 |
| N | 1.24280600 | -2.70920700 | -0.48336000 |
| C | -3.82108500 | -0.29740800 | -0.69998100 |
| H | -4.30042600 | 0.39486300 | -1.40621600 |
| C | -4.39056000 | -1.26349700 | 1.34213200 |
| C | -3.28197500 | 0.50524500 | 2.73751500 |
| H | -3.68775400 | -0.05000400 | 3.59311500 |
| H | -2.30914100 | 0.93664200 | 3.01385800 |
| C | 1.31104300 | -2.29328400 | 0.90326400 |
| N | -3.08565800 | 0.46669700 | 0.33093900 |
| O | -4.91535000 | -1.93943500 | 2.18393000 |
| C | -3.12822100 | -0.41612800 | 1.54134600 |
| H | -2.24930100 | -1.07738600 | 1.59040000 |
| N | -4.81472500 | -1.04910900 | 0.04904300 |
| C | -5.96554600 | -1.63685500 | -0.55186800 |
| C | -6.35550800 | -2.93463300 | -0.20857100 |
| C | -6.69291700 | -0.90875500 | -1.49930300 |
| C | -7.47506800 | -3.49534500 | -0.82085900 |
| C | -7.80075800 | -1.48626500 | -2.11487800 |
| C | -8.19531600 | -2.78066700 | -1.77704200 |
| H | -7.78407500 | -4.50452500 | -0.54639100 |
| H | -8.36556500 | -0.91553500 | -2.85271200 |
| H | -9.06699600 | -3.22866300 | -2.25459000 |
| C | -4.23797400 | 1.56206700 | 2.18362700 |
| H | -5.26999400 | 1.18402900 | 2.21300900 |
| C | -3.77717000 | 1.73918400 | 0.73369700 |
| H | -3.05790200 | 2.56153700 | 0.63909700 |
| H | -4.61181400 | 1.95410100 | 0.05202200 |
| H | -4.21733100 | 2.50678100 | 2.73842200 |
| H | -6.41049900 | 0.11505500 | -1.75191300 |
| H | -5.79844000 | -3.48897500 | 0.54457500 |
| H | 2.12075600 | -2.84531400 | 1.40679900 |
| C | 1.74005000 | -0.82323000 | 0.70427600 |
| H | 1.44112200 | -0.19310400 | 1.55016600 |
| N | 0.94467400 | -0.42687800 | -0.48352800 |
| H | 1.45672100 | 0.31279500 | -0.97118300 |
| S | 1.77728100 | -4.17358500 | -1.11978500 |
| O | 0.82561900 | -5.19986300 | -0.74462800 |
| O | 2.00718200 | -3.85774600 | -2.52469900 |
| C | 3.29529400 | -4.47424500 | -0.28499700 |

| | | | |
|---|---|---|---|
| C | 3.28685000 | -5.25642300 | 0.86978300 |
| C | 4.46903800 | -3.91883500 | -0.79378300 |
| C | 4.48759600 | -5.45416700 | 1.54526700 |
| H | 2.35631700 | -5.70819400 | 1.21802700 |
| C | 5.65630000 | -4.13690700 | -0.10629500 |
| H | 4.44540500 | -3.34641500 | -1.72165000 |
| C | 5.68471500 | -4.89911600 | 1.07219100 |
| H | 4.49897000 | -6.06437200 | 2.45006000 |
| H | 6.58629700 | -3.71807300 | -0.49595800 |
| C | 6.98427600 | -5.14953300 | 1.78400900 |
| H | 6.82265200 | -5.41065800 | 2.83712000 |
| H | 7.51865200 | -5.98772300 | 1.31106600 |
| H | 7.64165700 | -4.27135100 | 1.73327000 |
| C | 0.02717500 | -2.48386000 | 1.69924400 |
| C | -0.10431400 | -1.87657800 | 2.95548600 |
| C | -0.96783800 | -3.36881300 | 1.26371400 |
| C | -1.20567200 | -2.15200600 | 3.76685600 |
| H | 0.68299200 | -1.21592600 | 3.32818300 |
| C | -2.07164100 | -3.63707100 | 2.07493600 |
| H | -0.84781300 | -3.88698700 | 0.31001400 |
| C | -2.19438200 | -3.03294600 | 3.32716700 |
| H | -1.28557700 | -1.68832600 | 4.75145300 |
| H | -2.83741600 | -4.33495300 | 1.73301500 |
| H | -3.06311200 | -3.24518800 | 3.95066100 |
| C | 3.23977900 | -0.72492900 | 0.49422500 |
| C | 3.82633700 | -0.58502000 | -0.76560500 |
| C | 4.07232200 | -0.83724600 | 1.61529300 |
| C | 5.21637500 | -0.55353500 | -0.90279300 |
| H | 3.21449000 | -0.49400300 | -1.66704900 |
| C | 5.45863500 | -0.80238300 | 1.48234700 |
| H | 3.62939800 | -0.96578100 | 2.60771800 |
| C | 6.03561300 | -0.65721200 | 0.21824100 |
| H | 5.65808100 | -0.44877300 | -1.89554300 |
| H | 6.09123700 | -0.89246500 | 2.36658100 |
| H | 7.12092100 | -0.63191000 | 0.11055700 |
| Ni | -0.96457400 | 0.51970900 | -0.10830900 |
| C | 5.05875200 | 4.00134800 | 0.08433900 |
| C | 4.38062800 | 2.63391600 | 0.05487000 |
| H | 5.88268800 | 4.12557900 | -0.62812900 |
| H | 5.44799900 | 4.26339500 | 1.07921600 |
| H | 4.63559200 | 1.95163000 | 0.87744400 |
| H | 4.58713800 | 2.08931100 | -0.87838600 |
| N | 2.71869100 | 4.27074300 | -0.15440200 |
| C | 2.89991200 | 2.93244500 | 0.05595800 |
| C | 3.95107800 | 4.97873100 | -0.24416600 |
| O | 4.01941100 | 6.13545200 | -0.52306300 |
| O | 1.98811000 | 2.13056000 | 0.17835500 |
| C | 1.46060300 | 4.96621400 | -0.37544900 |
| C | 0.54115700 | 5.06228400 | 0.84375500 |
| C | -0.43155200 | 3.84932200 | 0.79080200 |
| H | 1.78361500 | 5.97578800 | -0.66255800 |
| H | -0.05897500 | 5.97539300 | 0.72301800 |
| H | 1.07402400 | 5.13583300 | 1.79716000 |
| C | 0.04674800 | 2.67592300 | 1.62663600 |
| O | -0.39904100 | 1.53535100 | 1.52152000 |
| O | 0.87264000 | 2.99952300 | 2.57304900 |
| C | -1.73639500 | 4.36399000 | 1.44646500 |
| O | -2.56380100 | 4.99130500 | 0.85010300 |
| O | -1.77131200 | 4.10078500 | 2.74341700 |
| C | 1.35135600 | 1.97296600 | 3.44584500 |
| H | 2.07155600 | 1.35190400 | 2.89430200 |
| H | 1.84566000 | 2.48809400 | 4.27327100 |
| H | 0.51461600 | 1.36331300 | 3.80732300 |
| C | -2.80128400 | 4.74453400 | 3.51070300 |
| H | -2.53263100 | 5.79726300 | 3.66393000 |
| H | -3.76012300 | 4.69045100 | 2.98278000 |
| H | -2.84147700 | 4.21873700 | 4.46835300 |
| C | 1.11570700 | 3.28637300 | -2.34183900 |
| C | 0.30209500 | 2.16047800 | -2.30628000 |

| | | | |
|---|---|---|---|
| C | 0.54330400 | 1.07065900 | -3.13754600 |
| C | 1.67932800 | 1.09553300 | -3.95215700 |
| C | 2.53335000 | 2.20122200 | -3.95142000 |
| C | 2.24090200 | 3.31569900 | -3.16117500 |
| C | 0.54195100 | 4.37610400 | -1.48052000 |
| H | -0.13913400 | 0.22007700 | -3.17612900 |
| H | 1.88505000 | 0.25065900 | -4.61139300 |
| H | 3.40878600 | 2.20961800 | -4.60142800 |
| H | 2.87482400 | 4.20522400 | -3.20214300 |
| N | -0.81785200 | 2.29720400 | -1.38478800 |
| C | -0.64302800 | 3.65727200 | -0.74936800 |
| H | -1.56334600 | 4.21896400 | -0.95370400 |
| C | 0.02452100 | 5.52902800 | -2.35659400 |
| H | -0.45224200 | 6.30571900 | -1.73943300 |
| H | 0.85241100 | 5.98930400 | -2.91364000 |
| H | -0.71487200 | 5.16613400 | -3.08387800 |
| C | -2.07950500 | 2.27696600 | -2.17572000 |
| H | -2.93260800 | 2.47245500 | -1.51703000 |
| H | -2.05396400 | 3.04514700 | -2.96319000 |
| H | -2.19855800 | 1.29462300 | -2.65234500 |

**1a**

| | | | |
|---|---|---|---|
| C | -0.09340300 | -0.75286600 | -0.00000300 |
| C | -0.26111000 | 0.65578800 | -0.00002800 |
| C | -1.52757000 | 1.25622600 | -0.00001300 |
| C | -2.63230900 | 0.41692700 | 0.00000200 |
| C | -2.48771500 | -0.98582400 | 0.00000900 |
| C | -1.23242800 | -1.57553500 | 0.00000900 |
| C | 1.32412700 | -1.01116800 | 0.00000400 |
| H | -1.64346800 | 2.34122900 | -0.00000700 |
| H | -3.63337800 | 0.85093900 | 0.00001200 |
| H | -3.38023900 | -1.61300700 | 0.00002100 |
| H | -1.12891700 | -2.66263000 | 0.00001900 |
| N | 0.98577700 | 1.22878900 | -0.00005600 |
| C | 1.92712100 | 0.21946300 | -0.00001400 |
| H | 2.98815600 | 0.46478500 | -0.00002000 |
| C | 1.98381600 | -2.35359600 | 0.00000500 |
| H | 1.69741600 | -2.94042700 | 0.88629000 |
| H | 1.69752500 | -2.94038000 | -0.88634600 |
| H | 3.07771000 | -2.25651900 | 0.00007500 |
| C | 1.24376400 | 2.64578200 | 0.00005500 |
| H | 2.32724500 | 2.81293200 | -0.00041000 |
| H | 0.81548600 | 3.12529700 | -0.89342400 |
| H | 0.81626500 | 3.12507400 | 0.89403200 |

**2a**

| | | | |
|---|---|---|---|
| C | -3.50530800 | -0.91613000 | 0.12330500 |
| C | -3.08041100 | -0.12346100 | 1.35751700 |
| H | -4.03363500 | -0.31176000 | -0.62659100 |
| H | -4.13913100 | -1.78536300 | 0.34173200 |
| H | -3.42058700 | -0.56263500 | 2.30507400 |
| H | -3.40776900 | 0.92449600 | 1.35232100 |
| N | -1.15366800 | -0.93805800 | 0.27031100 |
| C | -1.56093900 | -0.14403700 | 1.34112600 |
| C | -2.21188500 | -1.40752000 | -0.50986400 |
| O | -2.09586200 | -2.07149000 | -1.50051600 |
| O | -0.81118300 | 0.42187500 | 2.08606400 |
| C | 0.21875200 | -1.11253700 | -0.07626200 |
| C | 0.64192000 | -0.86747800 | -1.47154500 |
| C | 1.07416500 | 0.11556300 | -0.39551900 |
| H | 0.74641100 | -1.87926400 | 0.49222400 |
| H | -0.11798900 | -0.51342400 | -2.16670400 |
| H | 1.41686000 | -1.51529500 | -1.88003900 |
| C | 2.52888600 | 0.05972200 | -0.01487300 |
| O | 3.28834500 | 0.98156400 | -0.01128400 |
| O | 2.90940600 | -1.20212000 | 0.25432800 |
| C | 0.44861500 | 1.48196000 | -0.33699200 |
| O | 0.92259500 | 2.44378400 | 0.18585600 |
| O | -0.75704200 | 1.50159200 | -0.94339200 |

| | | | |
|---|---|---|---|
| C | 4.28725700 | -1.37128600 | 0.55420800 |
| H | 4.55812800 | -0.79133900 | 1.44612000 |
| H | 4.43419600 | -2.44086200 | 0.73192700 |
| H | 4.90776600 | -1.02984700 | -0.28495500 |
| C | -1.44779300 | 2.73908200 | -0.85105300 |
| H | -0.87079000 | 3.54014100 | -1.33130900 |
| H | -2.40479400 | 2.59780700 | -1.36410100 |
| H | -1.60261500 | 3.00883500 | 0.20336800 |

**3a**

| | | | |
|---|---|---|---|
| C | -3.40893500 | 2.41972100 | 0.09579900 |
| C | -2.70621900 | 1.99041200 | -1.18865700 |
| H | -4.41157900 | 1.99548200 | 0.22989000 |
| H | -3.49064800 | 3.51126600 | 0.20077000 |
| H | -2.62655000 | 2.76077600 | -1.96479200 |
| H | -3.17644100 | 1.10048100 | -1.63765900 |
| N | -1.30948700 | 1.49191900 | 0.64095900 |
| C | -1.32470300 | 1.54473500 | -0.74841400 |
| C | -2.51437500 | 1.90292600 | 1.20929200 |
| O | -2.76692200 | 1.84275800 | 2.38259100 |
| O | -0.39794200 | 1.24734800 | -1.45475200 |
| C | -0.34091100 | 0.75141500 | 1.43320800 |
| C | 1.12279500 | 1.08509800 | 1.13173000 |
| C | 1.71548600 | -0.05980900 | 0.25784900 |
| H | -0.59374500 | 1.02011100 | 2.46652300 |
| H | 1.66464900 | 1.14391200 | 2.08638100 |
| H | 1.23394200 | 2.05350500 | 0.63492000 |
| C | 2.12264300 | 0.38039200 | -1.15000000 |
| O | 2.35198600 | -0.39081100 | -2.04107500 |
| O | 2.30113300 | 1.69271200 | -1.26215300 |
| C | 2.99843400 | -0.51030600 | 0.96176200 |
| O | 3.06844200 | -1.39691800 | 1.76876900 |
| O | 4.03632700 | 0.25808000 | 0.63181400 |
| C | 2.59926900 | 2.15187200 | -2.56913800 |
| H | 1.76131700 | 1.91931400 | -3.24039900 |
| H | 2.73940400 | 3.23449000 | -2.49118100 |
| H | 3.50906500 | 1.67022400 | -2.95085100 |
| C | 5.26361300 | -0.03833400 | 1.28431300 |
| H | 5.15555500 | 0.07412000 | 2.37123800 |
| H | 5.57236500 | -1.06910100 | 1.06698800 |
| H | 5.99680000 | 0.67358000 | 0.89444700 |
| C | -1.71815400 | -1.20095800 | 0.51418800 |
| C | -1.34887600 | -1.62345800 | -0.77450300 |
| C | -2.32601500 | -2.00161800 | -1.70249900 |
| C | -3.66788600 | -1.93294900 | -1.31587800 |
| C | -4.03975200 | -1.50725100 | -0.03924100 |
| C | -3.05197800 | -1.14127700 | 0.88729400 |
| C | -0.47103800 | -0.80229900 | 1.26607000 |
| H | -2.04750800 | -2.33203500 | -2.70330600 |
| H | -4.43953500 | -2.22488800 | -2.03085100 |
| H | -5.09267300 | -1.47254900 | 0.24189100 |
| H | -3.32963400 | -0.80514300 | 1.88936800 |
| N | 0.01589000 | -1.62144700 | -0.94496600 |
| C | 0.67708300 | -1.24246800 | 0.29438100 |
| H | 1.19809700 | -2.11924100 | 0.70986700 |
| C | -0.33073100 | -1.46098000 | 2.63915900 |
| H | 0.63223900 | -1.19104300 | 3.09962700 |
| H | -1.14555400 | -1.13998800 | 3.30456300 |
| H | -0.36416700 | -2.55477100 | 2.54453400 |
| C | 0.61753400 | -2.54163100 | -1.88613000 |
| H | 1.65955400 | -2.72135500 | -1.60346600 |
| H | 0.06866400 | -3.50029600 | -1.89208200 |
| H | 0.63366200 | -2.12491700 | -2.90342400 |

**c-2a**

| | | | |
|---|---|---|---|
| C | 0.72066800 | 0.98485700 | -2.01943900 |
| C | 1.10977700 | 1.57560700 | -3.21835300 |
| C | 2.38691700 | 1.28889200 | -3.70591900 |
| C | 3.24406100 | 0.44912500 | -2.99205700 |

| | | | |
|---|---|---|---|
| C | 2.78134400 | -0.09383000 | -1.79710100 |
| H | 0.44057500 | 2.26502900 | -3.73137900 |
| H | 2.72073500 | 1.73477100 | -4.64390700 |
| H | 4.25448500 | 0.23590800 | -3.34387000 |
| C | -0.59430200 | 1.26419900 | -1.29341500 |
| H | -1.36784500 | 1.54844000 | -2.02450200 |
| N | 1.55227000 | 0.17485800 | -1.36473600 |
| N | -0.41513300 | 2.30408600 | -0.28935000 |
| C | 3.63692700 | -0.93818400 | -0.86357100 |
| H | 4.27659100 | -1.59720900 | -1.48089900 |
| C | 4.52685000 | -0.59602900 | 1.28878100 |
| C | 4.00256700 | -3.03006800 | 1.80068900 |
| H | 4.94141200 | -2.93039600 | 2.36061500 |
| H | 3.28959900 | -3.58051800 | 2.43137400 |
| C | -0.72495300 | 1.83902500 | 1.05375000 |
| N | 2.81200900 | -1.76014300 | 0.07977900 |
| O | 5.28869100 | -0.25177900 | 2.14803400 |
| C | 3.43839000 | -1.64714200 | 1.43996900 |
| H | 2.71503700 | -1.28132700 | 2.18435900 |
| N | 4.45784900 | -0.11646100 | 0.00308100 |
| C | 5.37738300 | 0.85032500 | -0.51439600 |
| C | 4.93095700 | 2.14480200 | -0.78863500 |
| C | 6.70884100 | 0.49441900 | -0.73274800 |
| C | 5.82198700 | 3.08604100 | -1.29958900 |
| C | 7.59670800 | 1.44222300 | -1.23807600 |
| C | 7.15318800 | 2.73359900 | -1.52471900 |
| H | 5.47843000 | 4.09911700 | -1.51081200 |
| H | 8.64061000 | 1.17314800 | -1.40165100 |
| H | 7.85202000 | 3.47302900 | -1.91734200 |
| C | 4.13969200 | -3.71167200 | 0.43917900 |
| H | 5.04676800 | -3.36707300 | -0.08320500 |
| C | 2.88891600 | -3.20731100 | -0.26964600 |
| H | 2.00156800 | -3.70705200 | 0.14765100 |
| H | 2.86785400 | -3.33487200 | -1.36029600 |
| H | 4.18521600 | -4.80610200 | 0.49606900 |
| H | 7.04819000 | -0.51388100 | -0.48893000 |
| H | 3.88846500 | 2.40701700 | -0.59656700 |
| H | -1.24973700 | 2.63607000 | 1.60435000 |
| C | -1.68692600 | 0.67866700 | 0.73164400 |
| H | -1.68164600 | -0.07729000 | 1.53170700 |
| N | -0.99508700 | 0.11176100 | -0.44558000 |
| H | -1.60069500 | -0.54375100 | -0.94987300 |
| S | -0.43158300 | 3.93136600 | -0.72013800 |
| O | 0.60516000 | 4.56023400 | 0.06888700 |
| O | -0.39011100 | 3.88112400 | -2.17365100 |
| C | -1.99228400 | 4.55681500 | -0.19299600 |
| C | -2.08753000 | 5.19143600 | 1.04430200 |
| C | -3.09918800 | 4.40539400 | -1.03085900 |
| C | -3.33260200 | 5.65871200 | 1.45974900 |
| H | -1.19387000 | 5.33569500 | 1.65406600 |
| C | -4.32704500 | 4.88725000 | -0.59930300 |
| H | -2.98684600 | 3.94386000 | -2.01317400 |
| C | -4.46538200 | 5.51363900 | 0.65020300 |
| H | -3.42140000 | 6.16285800 | 2.42368600 |
| H | -5.20080500 | 4.78544600 | -1.24624400 |
| C | -5.80488600 | 6.03775100 | 1.08508600 |
| H | -5.81108800 | 6.29852400 | 2.15039900 |
| H | -6.06466000 | 6.94242000 | 0.51506400 |
| H | -6.59610200 | 5.29773500 | 0.89769200 |
| C | 0.50465400 | 1.42330000 | 1.85309200 |
| C | 0.35444800 | 0.74017600 | 3.07027100 |
| C | 1.77837300 | 1.88147600 | 1.48698500 |
| C | 1.45147900 | 0.52473100 | 3.90365700 |
| H | -0.63986600 | 0.42424400 | 3.39801900 |
| C | 2.87274800 | 1.67320100 | 2.32681600 |
| H | 1.88144300 | 2.47299200 | 0.57554000 |
| C | 2.71371500 | 0.99546900 | 3.53517700 |
| H | 1.31737300 | 0.01719300 | 4.86054500 |
| H | 3.85787300 | 2.04976300 | 2.04529700 |

| | | | |
|---|---|---|---|
| H | 3.57788500 | 0.83541100 | 4.18126100 |
| C | -3.11625400 | 1.14035200 | 0.49313000 |
| C | -3.80337900 | 0.91342600 | -0.70161300 |
| C | -3.78471200 | 1.78376800 | 1.54311900 |
| C | -5.13463900 | 1.31387800 | -0.84110400 |
| H | -3.32409700 | 0.39989300 | -1.53601600 |
| C | -5.11126000 | 2.18092400 | 1.40648100 |
| H | -3.26733300 | 1.97044000 | 2.48849600 |
| C | -5.79308200 | 1.94213500 | 0.21185000 |
| H | -5.65677900 | 1.13261100 | -1.78197900 |
| H | -5.61550600 | 2.67776700 | 2.23672300 |
| H | -6.83441500 | 2.24927900 | 0.10368400 |
| Ni | 0.80254100 | -0.96572500 | 0.09528600 |
| C | -5.33031500 | -4.58450100 | -1.80971400 |
| C | -4.78918400 | -3.19318600 | -2.14787200 |
| H | -5.26489200 | -5.29914200 | -2.64105800 |
| H | -6.37584300 | -4.58454600 | -1.47366900 |
| H | -5.51307700 | -2.38407800 | -1.97277100 |
| H | -4.43904800 | -3.08744800 | -3.18366800 |
| N | -3.52450900 | -4.06060900 | -0.37415300 |
| C | -3.62025900 | -2.97519200 | -1.21389800 |
| C | -4.46462200 | -5.09080400 | -0.67330700 |
| O | -4.49263200 | -6.12690900 | -0.09048400 |
| O | -2.85010400 | -2.03765100 | -1.19454400 |
| C | -2.61746100 | -4.19204300 | 0.71103100 |
| C | -1.63441600 | -5.28302600 | 0.73106500 |
| C | -1.11680500 | -3.85955200 | 0.51221700 |
| H | -3.00523100 | -3.85011500 | 1.67539800 |
| H | -1.58985800 | -5.92511700 | -0.14855000 |
| H | -1.39664900 | -5.73921000 | 1.69067000 |
| C | -0.51584700 | -3.15413600 | 1.68451700 |
| O | 0.17471300 | -2.14112100 | 1.63592400 |
| O | -0.82080400 | -3.71039000 | 2.82034100 |
| C | -0.63068500 | -3.47475200 | -0.84812000 |
| O | 0.11285800 | -2.53295500 | -1.08823700 |
| O | -1.05290200 | -4.27357300 | -1.78850600 |
| C | -0.33739700 | -3.10185600 | 4.03232200 |
| H | -0.71627900 | -2.07576100 | 4.10393800 |
| H | -0.72009600 | -3.72235300 | 4.84560500 |
| H | 0.75902000 | -3.09300700 | 4.02556800 |
| C | -0.71779700 | -3.93717300 | -3.14457500 |
| H | 0.37114400 | -3.93224000 | -3.26895200 |
| H | -1.17352800 | -4.71308000 | -3.76435200 |
| H | -1.12593600 | -2.94586100 | -3.37964000 |

2. The enantio-determining transition state in Fig. 8

**TS1**

| | | | |
|---|---|---|---|
| C | 2.71410300 | 1.03127500 | -1.86405800 |
| C | 3.62375100 | 1.59903100 | -2.75196400 |
| C | 4.73695900 | 0.84367200 | -3.12563400 |
| C | 4.92877100 | -0.43259900 | -2.59649400 |
| C | 3.97889400 | -0.92271300 | -1.70479700 |
| H | 3.47285100 | 2.61506300 | -3.11354800 |
| H | 5.46466000 | 1.25907000 | -3.82401500 |
| H | 5.80592600 | -1.02875000 | -2.85224100 |
| C | 1.48014200 | 1.75310800 | -1.32292900 |
| H | 1.11919900 | 2.46156600 | -2.08712400 |
| N | 2.90692200 | -0.19927600 | -1.38748200 |
| N | 1.72934200 | 2.44127100 | -0.06475200 |
| C | 4.13403900 | -2.24317000 | -0.96390300 |
| H | 4.57120100 | -2.98301200 | -1.66161300 |
| C | 4.51023600 | -2.81157400 | 1.29051500 |
| C | 2.93018200 | -4.79340700 | 0.90742400 |
| H | 3.68225300 | -5.29409600 | 1.53084100 |
| H | 1.93692600 | -5.07209400 | 1.28822100 |
| C | 0.87852500 | 1.95600800 | 1.01284200 |
| N | 2.83781600 | -2.74272600 | -0.42020900 |
| O | 5.09031200 | -3.01503000 | 2.32050100 |
| C | 3.09988500 | -3.26485000 | 0.96000700 |

| | | | |
|---|---|---|---|
| H | 2.42299400 | -2.80045900 | 1.69098100 |
| N | 4.97515800 | -2.09949200 | 0.20933500 |
| C | 6.29653800 | -1.55676900 | 0.14686600 |
| C | 6.47581600 | -0.17395800 | 0.22910900 |
| C | 7.39343700 | -2.40879000 | 0.01358300 |
| C | 7.76060800 | 0.36021500 | 0.16181700 |
| C | 8.67700600 | -1.86924800 | -0.04677000 |
| C | 8.85976000 | -0.48787400 | 0.02286700 |
| H | 7.90378300 | 1.43905100 | 0.22962100 |
| H | 9.53836000 | -2.53103200 | -0.14181200 |
| H | 9.86618100 | -0.07025700 | -0.02209500 |
| C | 3.02752500 | -5.11184100 | -0.58591400 |
| H | 4.07978400 | -5.15699100 | -0.90979700 |
| C | 2.32914000 | -3.90433400 | -1.20108100 |
| H | 1.24170900 | -3.96960600 | -1.04471600 |
| H | 2.50471700 | -3.74376900 | -2.27357300 |
| H | 2.55532100 | -6.06241200 | -0.86254800 |
| H | 7.23903800 | -3.48847800 | -0.02439600 |
| H | 5.60552200 | 0.47526500 | 0.34731000 |
| H | 0.55378600 | 2.80116700 | 1.63993300 |
| C | -0.30962900 | 1.39478000 | 0.19803000 |
| H | -0.84335700 | 0.60978700 | 0.75818900 |
| N | 0.44924300 | 0.77558700 | -0.90135300 |
| H | -0.13465300 | 0.49543000 | -1.68910000 |
| S | 2.47628100 | 3.94383300 | -0.01898400 |
| O | 3.35089600 | 3.91808500 | 1.13150500 |
| O | 2.96294900 | 4.11131200 | -1.37952400 |
| C | 1.19051000 | 5.11410000 | 0.28560200 |
| C | 0.93764600 | 5.52307400 | 1.59369500 |
| C | 0.46220700 | 5.61482900 | -0.79530600 |
| C | -0.09117600 | 6.43476100 | 1.82106800 |
| H | 1.55784200 | 5.15018500 | 2.41089700 |
| C | -0.55128700 | 6.53036700 | -0.54548600 |
| H | 0.71315300 | 5.31293000 | -1.81344200 |
| C | -0.84737400 | 6.94986800 | 0.76141400 |
| H | -0.29574400 | 6.76859400 | 2.83994600 |
| H | -1.12147900 | 6.94104300 | -1.38146600 |
| C | -1.93523200 | 7.95878900 | 1.00236800 |
| H | -2.82155100 | 7.73902800 | 0.39042000 |
| H | -2.23261700 | 7.98834400 | 2.05785300 |
| H | -1.58997200 | 8.96586900 | 0.72351700 |
| C | 1.56119000 | 0.91080000 | 1.88561500 |
| C | 0.84222900 | 0.26790200 | 2.90196300 |
| C | 2.93315700 | 0.65724800 | 1.75890000 |
| C | 1.48359300 | -0.61546000 | 3.76795200 |
| H | -0.22284800 | 0.48148300 | 3.03513500 |
| C | 3.57286800 | -0.23141700 | 2.62655200 |
| H | 3.50868700 | 1.22493800 | 1.02621400 |
| C | 2.84894700 | -0.87553100 | 3.62781500 |
| H | 0.92340500 | -1.08668700 | 4.57731200 |
| H | 4.64747600 | -0.40272900 | 2.54217000 |
| H | 3.35849700 | -1.56503000 | 4.30264300 |
| C | -1.30202900 | 2.46215100 | -0.24045900 |
| C | -1.59496300 | 2.71978300 | -1.58343100 |
| C | -1.97599500 | 3.19805100 | 0.74375200 |
| C | -2.52349300 | 3.70321200 | -1.93963000 |
| H | -1.09509500 | 2.17184200 | -2.38551400 |
| C | -2.90198400 | 4.17598300 | 0.39347700 |
| H | -1.78248900 | 2.99649000 | 1.80026100 |
| C | -3.17749900 | 4.43461400 | -0.95226600 |
| H | -2.72644500 | 3.89928900 | -2.99365900 |
| H | -3.41054300 | 4.74230000 | 1.17527500 |
| H | -3.89840200 | 5.20621700 | -1.22710000 |
| Ni | 1.42301100 | -1.12566400 | -0.37778500 |
| C | -4.76870800 | 1.04364900 | -0.74833500 |
| C | -5.00296100 | 0.33466200 | -2.08237200 |
| H | -5.71084700 | 1.21981300 | -0.20251800 |
| H | -4.24043500 | 2.00311600 | -0.81612800 |
| H | -4.37892800 | 0.74109800 | -2.89353200 |

| | | | |
|---|---|---|---|
| H | -6.04502600 | 0.36447800 | -2.42695900 |
| N | -3.96921100 | -1.18550500 | -0.59176900 |
| C | -4.59909700 | -1.11119600 | -1.87625600 |
| C | -3.96499100 | 0.08727100 | 0.08239400 |
| O | -3.37026200 | 0.24911100 | 1.10634600 |
| O | -4.73377000 | -2.04454300 | -2.60571400 |
| C | -3.48799100 | -2.26037800 | 0.05212400 |
| C | -2.89929600 | -3.46775800 | -0.55823000 |
| C | -1.51298100 | -2.85137800 | -0.54279900 |
| H | -3.22841600 | -2.05028700 | 1.08984200 |
| H | -3.25730300 | -3.67948200 | -1.56796400 |
| H | -2.96512500 | -4.33371500 | 0.10714200 |
| C | -0.79975700 | -2.75988100 | 0.69272100 |
| O | 0.25099200 | -2.12400000 | 0.90615600 |
| O | -1.38271700 | -3.39947400 | 1.69763600 |
| C | -0.97329800 | -2.26554900 | -1.73199800 |
| O | 0.17303200 | -1.79748000 | -1.87950000 |
| O | -1.81735500 | -2.25820000 | -2.75132900 |
| C | -0.79563800 | -3.28283000 | 2.99499700 |
| H | -0.83165200 | -2.23828900 | 3.32825200 |
| H | -1.39208900 | -3.92160800 | 3.65212100 |
| H | 0.24888100 | -3.61828500 | 2.97606100 |
| C | -1.35775600 | -1.79722200 | -4.01920200 |
| H | -0.50454600 | -2.39731200 | -4.35816800 |
| H | -2.20555700 | -1.91360000 | -4.69990400 |
| H | -1.05453700 | -0.74285400 | -3.96176700 |
| C | -6.34946200 | -1.39066400 | 1.42961900 |
| C | -7.02652100 | -1.52902400 | 0.19060200 |
| C | -7.91317900 | -0.55363500 | -0.29196700 |
| C | -8.10327200 | 0.57176400 | 0.49694500 |
| C | -7.42935400 | 0.73507700 | 1.73071900 |
| C | -6.55481100 | -0.22940000 | 2.20151700 |
| C | -5.58361700 | -2.59025900 | 1.62854400 |
| H | -8.45680600 | -0.68730100 | -1.22842500 |
| H | -8.80241300 | 1.34174600 | 0.16761600 |
| H | -7.61917500 | 1.62985300 | 2.32401800 |
| H | -6.04887600 | -0.10225700 | 3.16008600 |
| N | -6.69349000 | -2.75431600 | -0.34650900 |
| C | -5.83749700 | -3.38213900 | 0.51219800 |
| H | -5.51341900 | -4.40267900 | 0.31108000 |
| C | -4.78781300 | -2.95383500 | 2.84212500 |
| H | -4.13969100 | -2.11944900 | 3.15520500 |
| H | -5.44974700 | -3.18449900 | 3.68987500 |
| H | -4.15828200 | -3.83581900 | 2.65797300 |
| C | -7.27541200 | -3.31966800 | -1.54883900 |
| H | -6.80905600 | -4.29104100 | -1.74684200 |
| H | -8.35741500 | -3.46042400 | -1.41796300 |
| H | -7.08703800 | -2.66669100 | -2.41150700 |

***ent*-TS1**

| | | | |
|---|---|---|---|
| C | 2.17525000 | 0.51047700 | -2.05876000 |
| C | 2.85339700 | 0.78610300 | -3.24246800 |
| C | 3.85225400 | -0.10329200 | -3.64643100 |
| C | 4.16019500 | -1.21879700 | -2.86670200 |
| C | 3.44412800 | -1.41272000 | -1.68798100 |
| H | 2.62076700 | 1.68699600 | -3.80953200 |
| H | 4.39997200 | 0.08099100 | -4.57163500 |
| H | 4.95060600 | -1.91320200 | -3.15512700 |
| C | 1.12504400 | 1.42918700 | -1.43548200 |
| H | 0.63226000 | 2.00969000 | -2.23072500 |
| N | 2.48586700 | -0.56250800 | -1.33255600 |
| N | 1.73899700 | 2.31542300 | -0.45720100 |
| C | 3.74473400 | -2.52799600 | -0.69416900 |
| H | 3.98781700 | -3.44411300 | -1.26543100 |
| C | 4.63248600 | -2.52435500 | 1.48903000 |
| C | 2.95238400 | -4.32276900 | 2.11073400 |
| H | 3.79352200 | -4.64412300 | 2.73845400 |
| H | 2.03301400 | -4.39196400 | 2.70897000 |
| C | 1.24442100 | 2.08990300 | 0.89055500 |

| | | | |
|---|---|---|---|
| N | 2.60420500 | -2.79091700 | 0.23640200 |
| O | 5.44444000 | -2.53996100 | 2.37185300 |
| C | 3.16107600 | -2.87983600 | 1.62649000 |
| H | 2.69079700 | -2.14258800 | 2.29399700 |
| N | 4.84924100 | -2.17716400 | 0.17752600 |
| C | 6.14068000 | -1.83305600 | -0.33078400 |
| C | 6.41817200 | -0.50515400 | -0.66271800 |
| C | 7.11190700 | -2.82291200 | -0.48604700 |
| C | 7.67265200 | -0.16909100 | -1.16710200 |
| C | 8.36683400 | -2.47979700 | -0.98560200 |
| C | 8.64504700 | -1.15653500 | -1.32918700 |
| H | 7.89418100 | 0.86736500 | -1.42350900 |
| H | 9.13260300 | -3.24755900 | -1.10004600 |
| H | 9.62923200 | -0.89164100 | -1.71716700 |
| C | 2.79410200 | -5.10126900 | 0.80471500 |
| H | 3.77444700 | -5.28467800 | 0.33505700 |
| C | 1.97712500 | -4.11867300 | -0.02420300 |
| H | 0.94345000 | -4.08442200 | 0.35240000 |
| H | 1.94002800 | -4.31213900 | -1.10425200 |
| H | 2.29625800 | -6.07088900 | 0.92763800 |
| H | 6.88719300 | -3.85159400 | -0.19861300 |
| H | 5.64822000 | 0.25619400 | -0.52133100 |
| H | 1.14831300 | 3.05813100 | 1.40759000 |
| C | -0.15458500 | 1.52167300 | 0.57649700 |
| H | -0.50678800 | 0.86895200 | 1.38973200 |
| N | 0.16957100 | 0.66979500 | -0.58564200 |
| H | -0.67080700 | 0.40173800 | -1.10617600 |
| S | 2.52290100 | 3.72194400 | -0.94491900 |
| O | 3.78516300 | 3.76151000 | -0.23886600 |
| O | 2.44699600 | 3.65183800 | -2.39656700 |
| C | 1.51713600 | 5.04402900 | -0.35271900 |
| C | 1.82463100 | 5.63379800 | 0.87236000 |
| C | 0.43202600 | 5.46644400 | -1.12191400 |
| C | 1.00159200 | 6.65226500 | 1.34733000 |
| H | 2.70574700 | 5.30847200 | 1.42821300 |
| C | -0.36811200 | 6.49043400 | -0.63273500 |
| H | 0.23963000 | 5.01334000 | -2.09571100 |
| C | -0.10240600 | 7.09312500 | 0.60726100 |
| H | 1.23203900 | 7.12542400 | 2.30348500 |
| H | -1.21479200 | 6.83997200 | -1.22713100 |
| C | -0.97253200 | 8.21319900 | 1.10467100 |
| H | -0.83516000 | 8.38457300 | 2.17940200 |
| H | -0.72436700 | 9.14934600 | 0.58185200 |
| H | -2.03423400 | 8.00444000 | 0.91203500 |
| C | 2.12557700 | 1.18128600 | 1.73939600 |
| C | 1.64387900 | 0.67778200 | 2.95690600 |
| C | 3.48033800 | 1.00777000 | 1.42278500 |
| C | 2.50030500 | 0.03042400 | 3.84718300 |
| H | 0.59972200 | 0.83201000 | 3.23909200 |
| C | 4.33501700 | 0.36183300 | 2.31521500 |
| H | 3.86811400 | 1.44742000 | 0.50178600 |
| C | 3.85073300 | -0.12229700 | 3.53078000 |
| H | 2.11691200 | -0.33775100 | 4.80004900 |
| H | 5.39100400 | 0.23622500 | 2.06877900 |
| H | 4.52862400 | -0.63144400 | 4.21671800 |
| C | -1.17809800 | 2.61932500 | 0.33561400 |
| C | -1.80515700 | 2.83474200 | -0.89222600 |
| C | -1.52260200 | 3.43815000 | 1.42115500 |
| C | -2.77537900 | 3.83090800 | -1.02990500 |
| H | -1.57589400 | 2.21111500 | -1.75662600 |
| C | -2.48220300 | 4.43760100 | 1.28533100 |
| H | -1.03442000 | 3.29122900 | 2.38924400 |
| C | -3.11927400 | 4.63046000 | 0.05648100 |
| H | -3.26232300 | 3.97706700 | -1.99512000 |
| H | -2.73426100 | 5.06733500 | 2.13996200 |
| H | -3.87599400 | 5.40903900 | -0.05208800 |
| Ni | 1.18295400 | -1.17416000 | 0.06596900 |
| C | -4.93204400 | 0.34067500 | 2.02628100 |
| C | -4.51013200 | 1.10630800 | 0.76973100 |

| | | | |
|---|---|---|---|
| H | -5.99190400 | 0.46817600 | 2.28570500 |
| H | -4.34811300 | 0.61690700 | 2.91678200 |
| H | -3.83865900 | 1.95873400 | 0.94467400 |
| H | -5.37667200 | 1.48040100 | 0.19833600 |
| N | -4.05328700 | -1.19747800 | 0.46227000 |
| C | -3.82707300 | 0.09202300 | -0.10384800 |
| C | -4.68125800 | -1.12798400 | 1.74176100 |
| O | -4.92916200 | -2.07917200 | 2.41708700 |
| O | -3.15441800 | 0.23140500 | -1.08677100 |
| C | -3.69072800 | -2.28921800 | -0.24882000 |
| C | -3.11245000 | -3.52381500 | 0.32029500 |
| C | -1.72191800 | -2.91597300 | 0.26564700 |
| H | -3.42511700 | -2.05118500 | -1.27689700 |
| H | -3.21151200 | -4.37757900 | -0.35577300 |
| H | -3.44360500 | -3.74615800 | 1.33704400 |
| C | -1.20047800 | -2.24096700 | 1.41418600 |
| O | -0.09253800 | -1.67510200 | 1.52565300 |
| O | -2.02349200 | -2.23996300 | 2.44945400 |
| C | -1.03250600 | -2.85077600 | -0.99049400 |
| O | 0.03433800 | -2.25974400 | -1.23187400 |
| O | -1.66387900 | -3.47795600 | -1.97598300 |
| C | -1.58744800 | -1.64238600 | 3.66808300 |
| H | -1.44515900 | -0.56114800 | 3.53261400 |
| H | -2.38321500 | -1.83664300 | 4.39277800 |
| H | -0.64191900 | -2.08976100 | 3.99852600 |
| C | -1.06975500 | -3.44529800 | -3.27451700 |
| H | -0.08001400 | -3.91721100 | -3.25117700 |
| H | -1.74410400 | -4.00775300 | -3.92603400 |
| H | -0.96915000 | -2.40950700 | -3.62225200 |
| C | -6.31308300 | -1.22348300 | -1.60786800 |
| C | -7.06555000 | -1.17557300 | -0.40833600 |
| C | -7.79796800 | -0.04319400 | -0.02531100 |
| C | -7.74694800 | 1.05637700 | -0.87181500 |
| C | -6.98770500 | 1.03720600 | -2.06341400 |
| C | -6.27316200 | -0.08907600 | -2.44010800 |
| C | -5.73084700 | -2.53875600 | -1.68894100 |
| H | -8.40619200 | -0.03322300 | 0.88041700 |
| H | -8.32002500 | 1.94991600 | -0.62018700 |
| H | -6.98481500 | 1.91885300 | -2.70481400 |
| H | -5.70359100 | -0.09993700 | -3.37095700 |
| N | -6.95535400 | -2.40304100 | 0.21750600 |
| C | -6.16244600 | -3.20564400 | -0.54057900 |
| H | -5.99331700 | -4.24252000 | -0.25074600 |
| C | -5.01118700 | -3.12495500 | -2.86296600 |
| H | -5.72029400 | -3.37423000 | -3.66596300 |
| H | -4.28481700 | -2.41091700 | -3.28210400 |
| H | -4.47473500 | -4.04454300 | -2.59024100 |
| C | -7.65971000 | -2.78411700 | 1.42739800 |
| H | -7.34765000 | -3.79287700 | 1.71815600 |
| H | -7.41025900 | -2.09989800 | 2.24928100 |
| H | -8.74427000 | -2.77554200 | 1.25182300 |

**TS2**

| | | | |
|---|---|---|---|
| C | 1.30590800 | 0.92028700 | -1.70551200 |
| C | 1.89519900 | 1.57093900 | -2.78805500 |
| C | 3.25037900 | 1.34037700 | -3.02541900 |
| C | 3.98823300 | 0.49351300 | -2.19235700 |
| C | 3.31751800 | -0.14259700 | -1.15074600 |
| H | 1.31889500 | 2.26418100 | -3.40319900 |
| H | 3.74559200 | 1.84339100 | -3.85725700 |
| H | 5.05551400 | 0.33726700 | -2.35156200 |
| C | -0.12746400 | 1.21415100 | -1.23978200 |
| H | -0.81830100 | 1.08362200 | -2.08523300 |
| N | 2.01322600 | 0.07029500 | -0.96507700 |
| N | -0.24245800 | 2.56885300 | -0.74866300 |
| C | 3.97126400 | -1.08238300 | -0.12723100 |
| H | 4.09594000 | -2.08031000 | -0.56892000 |
| C | 5.02351100 | 0.20309100 | 1.50092900 |
| C | 3.49562800 | -0.52046400 | 3.36621500 |

| | | | |
|---|---|---|---|
| H | 4.11320400 | 0.11038100 | 4.01843000 |
| H | 2.45399000 | -0.48577000 | 3.72291700 |
| C | -0.18154300 | 2.62220900 | 0.69812500 |
| N | 3.13223600 | -1.18954500 | 1.08847800 |
| O | 5.81892500 | 0.88374000 | 2.08834200 |
| C | 3.57286000 | -0.03917400 | 1.92982600 |
| H | 2.97804000 | 0.85962700 | 1.71566100 |
| N | 5.22888000 | -0.53598600 | 0.35184600 |
| C | 6.44184800 | -0.60640800 | -0.39056000 |
| C | 7.33414600 | 0.47131600 | -0.39701900 |
| C | 6.72997000 | -1.75932500 | -1.13011800 |
| C | 8.50757100 | 0.38279000 | -1.14399100 |
| C | 7.90026900 | -1.82874700 | -1.88191100 |
| C | 8.79378900 | -0.75855000 | -1.89135500 |
| H | 9.20479000 | 1.22139000 | -1.13992200 |
| H | 8.11930700 | -2.73081000 | -2.45423800 |
| H | 9.71260500 | -0.81691700 | -2.47512800 |
| C | 4.00774400 | -1.95586000 | 3.23112800 |
| H | 5.10563600 | -1.95044300 | 3.17094400 |
| C | 3.39606000 | -2.42757500 | 1.90767800 |
| H | 2.43532100 | -2.93334900 | 2.07345000 |
| H | 4.05059600 | -3.11449800 | 1.35437700 |
| H | 3.72344800 | -2.60423200 | 4.06836600 |
| H | 6.05380800 | -2.61555300 | -1.11428100 |
| H | 7.11961700 | 1.35925700 | 0.19367600 |
| H | -0.79531800 | 3.46718100 | 1.04892200 |
| C | -0.92414700 | 1.29960900 | 1.03931600 |
| H | -0.57160900 | 0.88158400 | 1.99323400 |
| N | -0.52048900 | 0.38404100 | -0.07570800 |
| H | -1.32871200 | -0.20995800 | -0.29877000 |
| S | -0.43611900 | 3.84113100 | -1.83576700 |
| O | 0.83073600 | 4.53224000 | -1.98427800 |
| O | -1.03650900 | 3.18638800 | -2.99469500 |
| C | -1.57478200 | 4.90565400 | -1.02874500 |
| C | -1.07701900 | 5.95270400 | -0.25161600 |
| C | -2.94232900 | 4.68250600 | -1.18108600 |
| C | -1.98349800 | 6.77431000 | 0.40964700 |
| H | -0.00087200 | 6.12051500 | -0.18228700 |
| C | -3.82855500 | 5.51879800 | -0.51305700 |
| H | -3.29567400 | 3.87876700 | -1.82760100 |
| C | -3.36657400 | 6.57174800 | 0.29140300 |
| H | -1.61187000 | 7.59844300 | 1.02110200 |
| H | -4.90320300 | 5.36222800 | -0.62450700 |
| C | -4.33550600 | 7.49338700 | 0.97629300 |
| H | -3.91466900 | 7.89802300 | 1.90536500 |
| H | -4.57067200 | 8.34602800 | 0.32070100 |
| H | -5.27913300 | 6.98303700 | 1.20712100 |
| C | 1.20748300 | 2.76027700 | 1.30670000 |
| C | 1.35162700 | 2.64944100 | 2.69713600 |
| C | 2.31785100 | 3.12459000 | 0.53585100 |
| C | 2.58295100 | 2.89040000 | 3.30624800 |
| H | 0.48493100 | 2.41052700 | 3.31971800 |
| C | 3.55307400 | 3.35191700 | 1.14706000 |
| H | 2.20440300 | 3.27904200 | -0.53856200 |
| C | 3.69109000 | 3.23527700 | 2.53052600 |
| H | 2.67609300 | 2.81674100 | 4.39082800 |
| H | 4.41143500 | 3.63899500 | 0.53756500 |
| H | 4.65949100 | 3.40910000 | 2.99994200 |
| C | -2.41509500 | 1.55420900 | 1.13062100 |
| C | -3.23596300 | 1.57612600 | 0.00092100 |
| C | -2.96420000 | 1.88086200 | 2.37468100 |
| C | -4.58253000 | 1.91938000 | 0.11291500 |
| H | -2.83365600 | 1.32581300 | -0.98355700 |
| C | -4.30859300 | 2.23654200 | 2.48804800 |
| H | -2.33077500 | 1.87490100 | 3.26650100 |
| C | -5.12185100 | 2.25666400 | 1.35467000 |
| H | -5.21134600 | 1.93002100 | -0.77997700 |
| H | -4.72024400 | 2.50128300 | 3.46296100 |
| H | -6.17297600 | 2.53591500 | 1.44018000 |

| | | | |
|---|---|---|---|
| Ni | 1.10121500 | -0.96842900 | 0.47917200 |
| C | -5.84842100 | -2.47762400 | 2.02212700 |
| C | -4.96749500 | -1.31084200 | 1.58147400 |
| H | -6.90852300 | -2.38187900 | 1.75804400 |
| H | -5.80392700 | -2.66709800 | 3.10461100 |
| H | -4.62571300 | -0.63894200 | 2.38054200 |
| H | -5.44972600 | -0.67136200 | 0.82530500 |
| N | -4.02733100 | -3.29349100 | 0.74612400 |
| C | -3.78118900 | -1.95060700 | 0.90066100 |
| C | -5.26493200 | -3.68564800 | 1.31708200 |
| O | -5.70938900 | -4.78897500 | 1.22405500 |
| O | -2.77178400 | -1.38875600 | 0.52169500 |
| C | -3.25331100 | -4.24296200 | -0.04348300 |
| C | -1.91685300 | -4.72255700 | 0.56739900 |
| C | -0.88649400 | -3.61239500 | 0.46825800 |
| H | -3.92366600 | -5.11002500 | -0.12484200 |
| H | -1.58173800 | -5.58038100 | -0.03097400 |
| H | -2.06429200 | -5.08156800 | 1.59247300 |
| C | -0.89917600 | -2.68185400 | 1.62478900 |
| O | -0.09846100 | -1.76311500 | 1.85163600 |
| O | -1.79400400 | -2.97499100 | 2.53673900 |
| C | 0.47697900 | -3.84620200 | -0.00142200 |
| O | 1.29926800 | -2.93307000 | -0.18786400 |
| O | 0.74836200 | -5.07455200 | -0.36982900 |
| C | -1.93066500 | -2.07562300 | 3.63943200 |
| H | -2.17371600 | -1.07217100 | 3.25890400 |
| H | -2.74539000 | -2.47458600 | 4.25048300 |
| H | -1.00060400 | -2.03762500 | 4.21870400 |
| C | 2.02141600 | -5.34008100 | -0.97176700 |
| H | 2.14576400 | -4.73174200 | -1.87686800 |
| H | 2.82373700 | -5.11438100 | -0.25919100 |
| H | 2.01804000 | -6.40466400 | -1.21797900 |
| C | -3.61097400 | -2.46476400 | -1.95618800 |
| C | -2.64820400 | -1.55663800 | -2.41039600 |
| C | -2.99337300 | -0.36675000 | -3.03721400 |
| C | -4.35962800 | -0.07686000 | -3.15466000 |
| C | -5.32919200 | -0.95445700 | -2.67147900 |
| C | -4.95914100 | -2.17060600 | -2.07852400 |
| C | -2.90911600 | -3.70978400 | -1.45608100 |
| H | -2.25563000 | 0.32232200 | -3.45335300 |
| H | -4.66336800 | 0.84562200 | -3.65165600 |
| H | -6.38541300 | -0.71111200 | -2.79050300 |
| H | -5.72243200 | -2.87866600 | -1.74545100 |
| N | -1.35138700 | -2.07996200 | -2.15777600 |
| C | -1.44637500 | -3.26873500 | -1.51034100 |
| H | -0.72773100 | -4.02412900 | -1.84300000 |
| C | -3.09499300 | -4.84295900 | -2.48885500 |
| H | -2.56893300 | -5.75589200 | -2.17212600 |
| H | -4.16361600 | -5.07361800 | -2.59304800 |
| H | -2.71641200 | -4.53872000 | -3.47483500 |
| C | -0.21400100 | -1.75235000 | -3.00825000 |
| H | 0.71664300 | -2.05850800 | -2.51448700 |
| H | -0.30301000 | -2.25649500 | -3.98383500 |
| H | -0.17310600 | -0.67274500 | -3.18906300 |

***ent*-TS2**

| | | | |
|---|---|---|---|
| C | -1.79404500 | 0.94687100 | 1.86479500 |
| C | -2.39096800 | 1.31580700 | 3.06562900 |
| C | -3.48186100 | 0.57051200 | 3.51366500 |
| C | -3.94055200 | -0.51411500 | 2.76919200 |
| C | -3.28063700 | -0.82200300 | 1.57976100 |
| H | -2.02265600 | 2.18201700 | 3.61283400 |
| H | -3.97330100 | 0.83464900 | 4.45098100 |
| H | -4.78785200 | -1.11307000 | 3.10470000 |
| C | -0.63525900 | 1.71151000 | 1.23910600 |
| H | -0.01225200 | 2.13791900 | 2.04167500 |
| N | -2.24485700 | -0.09498000 | 1.16097200 |
| N | -1.10365300 | 2.76227700 | 0.34194300 |
| C | -3.70063400 | -1.98608500 | 0.68800600 |

| | | | |
|---|---|---|---|
| H | -3.71713600 | -2.90320200 | 1.29354800 |
| C | -4.90390200 | -1.29560700 | -1.20234000 |
| C | -3.30711400 | -2.38548600 | -2.81744500 |
| H | -4.10006600 | -2.17781500 | -3.54799900 |
| H | -2.33121000 | -2.21700600 | -3.29341800 |
| C | -0.62589900 | 2.56179600 | -1.02007600 |
| N | -2.78481200 | -2.16242800 | -0.46367800 |
| O | -5.77940600 | -0.87817000 | -1.90963700 |
| C | -3.44358600 | -1.46626800 | -1.61400700 |
| H | -3.01786400 | -0.46578700 | -1.78622100 |
| N | -5.00690000 | -1.74109700 | 0.09282600 |
| C | -6.20466600 | -1.72762600 | 0.86568700 |
| C | -7.04784600 | -0.61554100 | 0.83619100 |
| C | -6.51136100 | -2.83193800 | 1.66596100 |
| C | -8.20181100 | -0.61412200 | 1.61828100 |
| C | -7.65718500 | -2.81272500 | 2.45731100 |
| C | -8.50416200 | -1.70366900 | 2.43412100 |
| H | -8.86777700 | 0.24893200 | 1.59053800 |
| H | -7.89841500 | -3.67391900 | 3.08129100 |
| H | -9.40590700 | -1.69364700 | 3.04699800 |
| C | -3.40140600 | -3.76865600 | -2.17222300 |
| H | -4.45248800 | -4.00855700 | -1.95427000 |
| C | -2.61388600 | -3.59551200 | -0.87099100 |
| H | -1.54441100 | -3.79505100 | -1.02683500 |
| H | -2.95479000 | -4.26003200 | -0.06752400 |
| H | -3.00501700 | -4.58053000 | -2.79601900 |
| H | -5.86446900 | -3.71183200 | 1.66124800 |
| H | -6.80803900 | 0.23037700 | 0.19262200 |
| H | -0.36128000 | 3.53736800 | -1.45890600 |
| C | 0.64335700 | 1.73284400 | -0.74751300 |
| H | 0.91231200 | 1.11321300 | -1.60935000 |
| N | 0.14292600 | 0.84523600 | 0.31429200 |
| H | 0.90823300 | 0.37019700 | 0.80239500 |
| S | -1.52831500 | 4.27958200 | 0.94178100 |
| O | -2.67594300 | 4.70050300 | 0.16799700 |
| O | -1.58494300 | 4.06011900 | 2.37856000 |
| C | -0.20168400 | 5.38561200 | 0.58075800 |
| C | -0.25034600 | 6.14835000 | -0.58554200 |
| C | 0.83259000 | 5.52518500 | 1.50799900 |
| C | 0.78420300 | 7.04561000 | -0.84014100 |
| H | -1.10348900 | 6.06125600 | -1.26070300 |
| C | 1.84552600 | 6.43562400 | 1.23883800 |
| H | 0.81841800 | 4.95317700 | 2.43684200 |
| C | 1.84169700 | 7.20392600 | 0.06375900 |
| H | 0.75541300 | 7.65356100 | -1.74617700 |
| H | 2.65521500 | 6.56520800 | 1.96012800 |
| C | 2.94278400 | 8.19417300 | -0.19287100 |
| H | 2.91882100 | 8.56816300 | -1.22364100 |
| H | 2.84483100 | 9.05807100 | 0.48151200 |
| H | 3.92852700 | 7.74549100 | -0.00302800 |
| C | -1.65477600 | 1.89152300 | -1.92726700 |
| C | -1.26750600 | 1.15967500 | -3.06015400 |
| C | -3.02015100 | 2.14712500 | -1.72156800 |
| C | -2.22231400 | 0.71742300 | -3.98136000 |
| H | -0.21181300 | 0.97868900 | -3.27325500 |
| C | -3.96787600 | 1.70703500 | -2.64364900 |
| H | -3.32693800 | 2.73744900 | -0.85694700 |
| C | -3.57261700 | 0.99572800 | -3.77946000 |
| H | -1.90263500 | 0.17259200 | -4.87134800 |
| H | -5.02477800 | 1.91893600 | -2.47943600 |
| H | -4.32113300 | 0.66058300 | -4.49862100 |
| C | 1.83780900 | 2.58462700 | -0.34123300 |
| C | 2.51683400 | 2.41042500 | 0.86774300 |
| C | 2.30253400 | 3.55827900 | -1.23805700 |
| C | 3.62367900 | 3.20269300 | 1.18386100 |
| H | 2.22021100 | 1.64032700 | 1.58257000 |
| C | 3.41412200 | 4.33846900 | -0.93104700 |
| H | 1.79156400 | 3.71142200 | -2.19321600 |
| C | 4.07641700 | 4.16518000 | 0.28725100 |

| | | | |
|---|---|---|---|
| H | 4.13993300 | 3.04963500 | 2.13251700 |
| H | 3.75904500 | 5.09115500 | -1.64176900 |
| H | 4.94307300 | 4.78145100 | 0.53221200 |
| Ni | -1.06003600 | -0.84950400 | -0.30907000 |
| C | 5.15295600 | -3.56176600 | 3.49252700 |
| C | 4.51044600 | -2.19293800 | 3.29290100 |
| H | 6.21401100 | -3.53717300 | 3.76945200 |
| H | 4.64030000 | -4.17629100 | 4.24657900 |
| H | 3.79312700 | -1.89836400 | 4.06937900 |
| H | 5.25061600 | -1.38239200 | 3.21201800 |
| N | 4.16135600 | -3.45854300 | 1.33856600 |
| C | 3.80974100 | -2.26989700 | 1.95177800 |
| C | 5.00899000 | -4.25104300 | 2.15186400 |
| O | 5.49899300 | -5.27740100 | 1.78787200 |
| O | 3.07002500 | -1.42775700 | 1.48728500 |
| C | 3.95925200 | -3.84771500 | -0.05484200 |
| C | 2.54323200 | -4.27628700 | -0.49876100 |
| C | 1.66162500 | -3.03654400 | -0.59633200 |
| H | 4.62593900 | -4.71327600 | -0.17021600 |
| H | 2.65308800 | -4.72046900 | -1.49629000 |
| H | 2.12869400 | -5.04832400 | 0.15810200 |
| C | 1.02504700 | -2.71444200 | 0.71367600 |
| O | 0.13766700 | -1.88491800 | 0.92580700 |
| O | 1.37107200 | -3.52440800 | 1.68620300 |
| C | 0.70915500 | -2.84869800 | -1.69993900 |
| O | -0.09096500 | -1.90198700 | -1.79850300 |
| O | 0.86037100 | -3.69792300 | -2.68952200 |
| C | 0.84595300 | -3.25794400 | 2.98637700 |
| H | 1.12644700 | -2.24067400 | 3.29085900 |
| H | 1.29098700 | -4.00692200 | 3.64775900 |
| H | -0.24696400 | -3.35068400 | 2.98341000 |
| C | 0.10230600 | -3.52062000 | -3.89088500 |
| H | 0.14186000 | -2.47389100 | -4.21764600 |
| H | -0.93900800 | -3.81615200 | -3.71426300 |
| H | 0.55796500 | -4.18135400 | -4.63279100 |
| C | 5.14393000 | -1.55614500 | -0.52807100 |
| C | 4.49113400 | -0.38183900 | -0.90791700 |
| C | 5.03727700 | 0.87045900 | -0.67712500 |
| C | 6.26020800 | 0.92221300 | 0.00193500 |
| C | 6.90898600 | -0.24117300 | 0.41644800 |
| C | 6.35692100 | -1.49979800 | 0.14077500 |
| C | 4.35770000 | -2.73707400 | -1.04769800 |
| H | 4.54109500 | 1.78420600 | -1.00513000 |
| H | 6.71534600 | 1.89364700 | 0.20026400 |
| H | 7.86759600 | -0.17141600 | 0.93125100 |
| H | 6.88772100 | -2.41274000 | 0.42423800 |
| N | 3.27822100 | -0.69661600 | -1.58021500 |
| C | 3.09155300 | -2.04942600 | -1.58222500 |
| H | 2.74057800 | -2.43819800 | -2.54334000 |
| C | 5.10708900 | -3.35431500 | -2.24624200 |
| H | 4.55191000 | -4.20417000 | -2.67097400 |
| H | 6.09424400 | -3.70898900 | -1.92074500 |
| H | 5.26180300 | -2.60342600 | -3.03407800 |
| C | 2.86453800 | 0.10088800 | -2.72740900 |
| H | 1.86486300 | -0.21849800 | -3.04959600 |
| H | 3.57335500 | -0.02324800 | -3.56277300 |
| H | 2.83438600 | 1.16351200 | -2.45820600 |

3. The complex of Ni(II) with **L4** in Supplementary Fig. 131

**c-1**

| | | | |
|---|---|---|---|
| C | -0.11850100 | 0.33900700 | 1.92523900 |
| C | -0.15833800 | 1.15601800 | 3.05072300 |
| C | -1.41029900 | 1.46832200 | 3.58667500 |
| C | -2.57916200 | 0.94641300 | 3.02020500 |
| C | -2.44857600 | 0.15580900 | 1.88670100 |
| H | 0.76400400 | 1.54543900 | 3.48089400 |
| H | -1.47590100 | 2.10951700 | 4.46700400 |
| H | -3.56047700 | 1.15682900 | 3.44691600 |

| | | | |
|---|---|---|---|
| C | 1.12225500 | -0.19999100 | 1.23091500 |
| H | 1.98432200 | -0.16904100 | 1.91685100 |
| N | -1.24805800 | -0.08734200 | 1.36810600 |
| N | 1.40969600 | 0.46956600 | -0.03348600 |
| C | -3.56133200 | -0.51570100 | 1.11372500 |
| H | -4.42986200 | -0.67687700 | 1.77936800 |
| C | -4.43096800 | -0.69169000 | -1.04046800 |
| C | -5.25578300 | -2.85182900 | 0.12930700 |
| H | -6.13884100 | -2.20621900 | 0.22809800 |
| H | -5.55505100 | -3.69858900 | -0.49843100 |
| C | 1.53982600 | -0.48700700 | -1.13200000 |
| N | -3.08797800 | -1.82270100 | 0.54022800 |
| O | -5.00330600 | -0.41550300 | -2.05420100 |
| C | -4.08329800 | -2.09001300 | -0.53891800 |
| H | -3.63182300 | -2.67739500 | -1.34965700 |
| N | -3.93586600 | 0.19507000 | -0.09607300 |
| C | -4.21250400 | 1.60203700 | -0.12116000 |
| C | -3.15251100 | 2.51211700 | -0.05361800 |
| C | -5.52951200 | 2.05033400 | -0.22841500 |
| C | -3.41753200 | 3.87944000 | -0.07416800 |
| C | -5.78438200 | 3.42018500 | -0.26213600 |
| C | -4.73312000 | 4.33288900 | -0.17934900 |
| H | -2.59381900 | 4.59202300 | -0.02050100 |
| H | -6.81122500 | 3.77487500 | -0.35520300 |
| H | -4.93885500 | 5.40347400 | -0.20464200 |
| C | -4.72184400 | -3.30002200 | 1.50943800 |
| H | -5.23661000 | -2.76989100 | 2.32172900 |
| C | -3.22567000 | -2.95578000 | 1.49376000 |
| H | -2.63330400 | -3.79285500 | 1.09758200 |
| H | -2.81288300 | -2.67523000 | 2.47330400 |
| H | -4.87342400 | -4.37089200 | 1.68884600 |
| H | -6.34679600 | 1.33203300 | -0.30607400 |
| H | -2.12253100 | 2.15205200 | 0.00484300 |
| H | 2.35922300 | -0.19516700 | -1.80656400 |
| C | 1.84329500 | -1.78056200 | -0.35077700 |
| H | 1.57460700 | -2.68655900 | -0.90765900 |
| N | 0.84704100 | -1.58105700 | 0.73379600 |
| H | 0.93837400 | -2.27018300 | 1.48314900 |
| S | 2.18475800 | 1.97628800 | -0.01514900 |
| O | 1.70733300 | 2.64163200 | -1.20984600 |
| O | 1.89025200 | 2.44599500 | 1.32753500 |
| C | 3.90593300 | 1.67082200 | -0.18361100 |
| C | 4.49240300 | 1.77391600 | -1.44468800 |
| C | 4.65221700 | 1.35254300 | 0.95521900 |
| C | 5.85735700 | 1.52457600 | -1.56538300 |
| H | 3.89382700 | 2.07702000 | -2.30546100 |
| C | 6.01008200 | 1.10908300 | 0.80866600 |
| H | 4.18193000 | 1.33445600 | 1.93970600 |
| C | 6.63290200 | 1.18803500 | -0.44886100 |
| H | 6.33324700 | 1.61262200 | -2.54363400 |
| H | 6.60957100 | 0.86656400 | 1.68845800 |
| C | 8.10874100 | 0.93974900 | -0.57373600 |
| H | 8.41360100 | 0.82432500 | -1.62081600 |
| H | 8.67355400 | 1.78331000 | -0.14895800 |
| H | 8.40417300 | 0.03931600 | -0.01652700 |
| C | 0.21481600 | -0.58285100 | -1.90093700 |
| C | -0.27598400 | -1.80780000 | -2.42222000 |
| C | -0.57613500 | 0.57232800 | -2.05748100 |
| C | -1.51274200 | -1.85395900 | -3.08333200 |
| H | 0.32319500 | -2.71828200 | -2.38458800 |
| C | -1.80021900 | 0.51705700 | -2.73406200 |
| H | -0.20336900 | 1.51994400 | -1.66074700 |
| C | -2.26905200 | -0.69049400 | -3.25459900 |
| H | -1.86183300 | -2.80205500 | -3.49684900 |
| H | -2.39480600 | 1.42578500 | -2.84868100 |
| H | -3.22771700 | -0.72571700 | -3.77301000 |
| C | 3.28820400 | -1.91202100 | 0.10275200 |
| C | 3.63269600 | -2.22360900 | 1.42096300 |
| C | 4.30288900 | -1.81994300 | -0.85773100 |

| | | | |
|---|---|---|---|
| C | 4.96595100 | -2.43509000 | 1.77630300 |
| H | 2.87349900 | -2.32469500 | 2.20101000 |
| C | 5.63202800 | -2.03613400 | -0.50540400 |
| H | 4.06231300 | -1.59876000 | -1.90054800 |
| C | 5.96725100 | -2.34539100 | 0.81340900 |
| H | 5.21676100 | -2.68416300 | 2.80795900 |
| H | 6.40934900 | -1.96893400 | -1.26806800 |
| H | 7.00770400 | -2.52484200 | 1.08784000 |
| Ni | -1.15166500 | -1.31465500 | -0.19343400 |

**c-2**

| | | | |
|---|---|---|---|
| C | 0.31825800 | 0.92110500 | -1.26552800 |
| C | 0.56657100 | 1.79004500 | -2.31404400 |
| C | 1.85523700 | 1.76838400 | -2.87282300 |
| C | 2.84987800 | 0.91621400 | -2.38914000 |
| C | 2.52677600 | 0.08955000 | -1.31249300 |
| H | -0.22270300 | 2.42940200 | -2.70490400 |
| H | 2.08138300 | 2.43464200 | -3.70698900 |
| H | 3.85288300 | 0.91168100 | -2.81851000 |
| C | -0.92009700 | 0.59197800 | -0.43831000 |
| H | -1.12221400 | -0.47733500 | -0.67511700 |
| N | 1.29745800 | 0.11561700 | -0.81557400 |
| N | -2.15129000 | 1.33378700 | -0.40586300 |
| C | 3.49544300 | -0.81456700 | -0.55624200 |
| H | 4.05013900 | -1.42851700 | -1.29086400 |
| C | 4.72330400 | -0.73740700 | 1.44469700 |
| C | 4.30240400 | -3.28013900 | 1.55883000 |
| H | 5.37315500 | -3.25774800 | 1.79974300 |
| H | 3.81253300 | -3.92129500 | 2.30374300 |
| C | -2.78926700 | 1.15345000 | 0.92533300 |
| N | 2.78462900 | -1.72373300 | 0.42172800 |
| O | 5.62167300 | -0.49871300 | 2.19002400 |
| C | 3.70138100 | -1.86173100 | 1.61003300 |
| H | 3.16484500 | -1.69070600 | 2.55619300 |
| N | 4.39609900 | -0.05633900 | 0.28220400 |
| C | 5.26712200 | 0.92687600 | -0.29811700 |
| C | 4.98074800 | 2.28113600 | -0.13324500 |
| C | 6.37655800 | 0.51332700 | -1.03793100 |
| C | 5.80897600 | 3.23311600 | -0.72517800 |
| C | 7.19840700 | 1.46999500 | -1.63064200 |
| C | 6.91242600 | 2.82744300 | -1.47636200 |
| H | 5.59827100 | 4.29471400 | -0.59255900 |
| H | 8.07168200 | 1.15555700 | -2.20288500 |
| H | 7.56152700 | 3.57394500 | -1.93540100 |
| C | 3.99018200 | -3.76672000 | 0.14070300 |
| H | 4.75459500 | -3.42568600 | -0.57434700 |
| C | 2.64783800 | -3.10273400 | -0.15134800 |
| H | 1.83442500 | -3.61936300 | 0.38093600 |
| H | 2.37533900 | -3.03759400 | -1.21397100 |
| H | 3.93825000 | -4.85943600 | 0.06212600 |
| H | 6.60645900 | -0.55015000 | -1.13547200 |
| H | 4.11886000 | 2.58143100 | 0.46499700 |
| H | -3.73753100 | 0.60025700 | 0.82592100 |
| C | -1.74309400 | 0.29992100 | 1.70193200 |
| H | -1.67572400 | 0.64384900 | 2.74324500 |
| N | -0.48936600 | 0.63529000 | 0.98737500 |
| H | -0.23039400 | 1.60882400 | 1.19532000 |
| S | -3.15174000 | 1.34454300 | -1.78600300 |
| O | -4.09546200 | 2.40613300 | -1.55671600 |
| O | -2.19684300 | 1.32621500 | -2.87970500 |
| C | -4.00236000 | -0.20273300 | -1.75997000 |
| C | -5.26210900 | -0.27334800 | -1.16008500 |
| C | -3.39967600 | -1.32331800 | -2.33380500 |
| C | -5.90347600 | -1.50692800 | -1.09904600 |
| H | -5.73382000 | 0.63197500 | -0.77185200 |
| C | -4.06168500 | -2.54646000 | -2.26570400 |
| H | -2.44903800 | -1.22326900 | -2.86102400 |
| C | -5.31564700 | -2.66010700 | -1.64565500 |
| H | -6.89190100 | -1.57448400 | -0.64021200 |

| | | | |
|---|---|---|---|
| H | -3.61161500 | -3.42768000 | -2.72756000 |
| C | -6.04760000 | -3.97231500 | -1.61776900 |
| H | -6.60096300 | -4.10352800 | -0.67854400 |
| H | -6.78369200 | -4.00638500 | -2.43569600 |
| H | -5.36565500 | -4.82124900 | -1.75458900 |
| C | -3.05288300 | 2.46915500 | 1.63850100 |
| C | -3.66229300 | 2.42643400 | 2.89933700 |
| C | -2.71292700 | 3.69992600 | 1.07767300 |
| C | -3.91344900 | 3.60249700 | 3.59848600 |
| H | -3.95789400 | 1.46771500 | 3.33690700 |
| C | -2.96633300 | 4.87907900 | 1.78234500 |
| H | -2.28454600 | 3.74201500 | 0.07685300 |
| C | -3.56081900 | 4.83333700 | 3.04006700 |
| H | -4.39389400 | 3.56195600 | 4.57661300 |
| H | -2.70841900 | 5.83950800 | 1.33484600 |
| H | -3.76263300 | 5.75666000 | 3.58401500 |
| C | -1.78507600 | -1.22252000 | 1.66500500 |
| C | -0.66431400 | -1.88886300 | 2.23589600 |
| C | -2.76212200 | -1.98674100 | 1.04023700 |
| C | -0.53679500 | -3.28241400 | 2.16304000 |
| H | -0.00769100 | -1.33178000 | 2.92973700 |
| C | -2.62463600 | -3.38170000 | 0.96711400 |
| H | -3.64061000 | -1.51903100 | 0.59101100 |
| C | -1.51600900 | -4.03005700 | 1.50502200 |
| H | 0.29612300 | -3.78340500 | 2.66159400 |
| H | -3.40611400 | -3.96099000 | 0.47181400 |
| H | -1.43055100 | -5.11535600 | 1.44546600 |
| Ni | 0.98372300 | -0.81165300 | 0.91330900 |

4. The structure of complex **c-2a** in Supplementary Fig. 132

**c-2a**

| | | | |
|---|---|---|---|
| C | 0.72066800 | 0.98485700 | -2.01943900 |
| C | 1.10977700 | 1.57560700 | -3.21835300 |
| C | 2.38691700 | 1.28889200 | -3.70591900 |
| C | 3.24406100 | 0.44912500 | -2.99205700 |
| C | 2.78134400 | -0.09383000 | -1.79710100 |
| H | 0.44057500 | 2.26502900 | -3.73137900 |
| H | 2.72073500 | 1.73477100 | -4.64390700 |
| H | 4.25448500 | 0.23590800 | -3.34387000 |
| C | -0.59430200 | 1.26419900 | -1.29341500 |
| H | -1.36784500 | 1.54844000 | -2.02450200 |
| N | 1.55227000 | 0.17485800 | -1.36473600 |
| N | -0.41513300 | 2.30408600 | -0.28935000 |
| C | 3.63692700 | -0.93818400 | -0.86357100 |
| H | 4.27659100 | -1.59720900 | -1.48089900 |
| C | 4.52685000 | -0.59602900 | 1.28878100 |
| C | 4.00256700 | -3.03006800 | 1.80068900 |
| H | 4.94141200 | -2.93039600 | 2.36061500 |
| H | 3.28959900 | -3.58051800 | 2.43137400 |
| C | -0.72495300 | 1.83902500 | 1.05375000 |
| N | 2.81200900 | -1.76014300 | 0.07977900 |
| O | 5.28869100 | -0.25177900 | 2.14803400 |
| C | 3.43839000 | -1.64714200 | 1.43996900 |
| H | 2.71503700 | -1.28132700 | 2.18435900 |
| N | 4.45784900 | -0.11646100 | 0.00308100 |
| C | 5.37738300 | 0.85032500 | -0.51439600 |
| C | 4.93095700 | 2.14480200 | -0.78863500 |
| C | 6.70884100 | 0.49441900 | -0.73274800 |
| C | 5.82198700 | 3.08604100 | -1.29958900 |
| C | 7.59670800 | 1.44222300 | -1.23807600 |
| C | 7.15318800 | 2.73359900 | -1.52471900 |
| H | 5.47843000 | 4.09911700 | -1.51081200 |
| H | 8.64061000 | 1.17314800 | -1.40165100 |
| H | 7.85202000 | 3.47302900 | -1.91734200 |
| C | 4.13969200 | -3.71167200 | 0.43917900 |
| H | 5.04676800 | -3.36707300 | -0.08320500 |
| C | 2.88891600 | -3.20731100 | -0.26964600 |
| H | 2.00156800 | -3.70705200 | 0.14765100 |
| H | 2.86785400 | -3.33487200 | -1.36029600 |

| | | | |
|---|---|---|---|
| H | 4.18521600 | -4.80610200 | 0.49606900 |
| H | 7.04819000 | -0.51388100 | -0.48893000 |
| H | 3.88846500 | 2.40701700 | -0.59656700 |
| H | -1.24973700 | 2.63607000 | 1.60435000 |
| C | -1.68692600 | 0.67866700 | 0.73164400 |
| H | -1.68164600 | -0.07729000 | 1.53170700 |
| N | -0.99508700 | 0.11176100 | -0.44558000 |
| H | -1.60069500 | -0.54375100 | -0.94987300 |
| S | -0.43158300 | 3.93136600 | -0.72013800 |
| O | 0.60516000 | 4.56023400 | 0.06888700 |
| O | -0.39011100 | 3.88112400 | -2.17365100 |
| C | -1.99228400 | 4.55681500 | -0.19299600 |
| C | -2.08753000 | 5.19143600 | 1.04430200 |
| C | -3.09918800 | 4.40539400 | -1.03085900 |
| C | -3.33260200 | 5.65871200 | 1.45974900 |
| H | -1.19387000 | 5.33569500 | 1.65406600 |
| C | -4.32704500 | 4.88725000 | -0.59930300 |
| H | -2.98684600 | 3.94386000 | -2.01317400 |
| C | -4.46538200 | 5.51363900 | 0.65020300 |
| H | -3.42140000 | 6.16285800 | 2.42368600 |
| H | -5.20080500 | 4.78544600 | -1.24624400 |
| C | -5.80488600 | 6.03775100 | 1.08508600 |
| H | -5.81108800 | 6.29852400 | 2.15039900 |
| H | -6.06466000 | 6.94242000 | 0.51506400 |
| H | -6.59610200 | 5.29773500 | 0.89769200 |
| C | 0.50465400 | 1.42330000 | 1.85309200 |
| C | 0.35444800 | 0.74017600 | 3.07027100 |
| C | 1.77837300 | 1.88147600 | 1.48698500 |
| C | 1.45147900 | 0.52473100 | 3.90365700 |
| H | -0.63986600 | 0.42424400 | 3.39801900 |
| C | 2.87274800 | 1.67320100 | 2.32681600 |
| H | 1.88144300 | 2.47299200 | 0.57554000 |
| C | 2.71371500 | 0.99546900 | 3.53517700 |
| H | 1.31737300 | 0.01719300 | 4.86054500 |
| H | 3.85787300 | 2.04976300 | 2.04529700 |
| H | 3.57788500 | 0.83541100 | 4.18126100 |
| C | -3.11625400 | 1.14035200 | 0.49313000 |
| C | -3.80337900 | 0.91342600 | -0.70161300 |
| C | -3.78471200 | 1.78376800 | 1.54311900 |
| C | -5.13463900 | 1.31387800 | -0.84110400 |
| H | -3.32409700 | 0.39989300 | -1.53601600 |
| C | -5.11126000 | 2.18092400 | 1.40648100 |
| H | -3.26733300 | 1.97044000 | 2.48849600 |
| C | -5.79308200 | 1.94213500 | 0.21185000 |
| H | -5.65677900 | 1.13261100 | -1.78197900 |
| H | -5.61550600 | 2.67776700 | 2.23672300 |
| H | -6.83441500 | 2.24927900 | 0.10368400 |
| Ni | 0.80254100 | -0.96572500 | 0.09528600 |
| C | -5.33031500 | -4.58450100 | -1.80971400 |
| C | -4.78918400 | -3.19318600 | -2.14787200 |
| H | -5.26489200 | -5.29914200 | -2.64105800 |
| H | -6.37584300 | -4.58454600 | -1.47366900 |
| H | -5.51307700 | -2.38407800 | -1.97277100 |
| H | -4.43904800 | -3.08744800 | -3.18366800 |
| N | -3.52450900 | -4.06060900 | -0.37415300 |
| C | -3.62025900 | -2.97519200 | -1.21389800 |
| C | -4.46462200 | -5.09080400 | -0.67330700 |
| O | -4.49263200 | -6.12690900 | -0.09048400 |
| O | -2.85010400 | -2.03765100 | -1.19454400 |
| C | -2.61746100 | -4.19204300 | 0.71103100 |
| C | -1.63441600 | -5.28302600 | 0.73106500 |
| C | -1.11680500 | -3.85955200 | 0.51221700 |
| H | -3.00523100 | -3.85011500 | 1.67539800 |
| H | -1.58985800 | -5.92511700 | -0.14855000 |
| H | -1.39664900 | -5.73921000 | 1.69067000 |
| C | -0.51584700 | -3.15413600 | 1.68451700 |
| O | 0.17471300 | -2.14112100 | 1.63592400 |
| O | -0.82080400 | -3.71039000 | 2.82034100 |
| C | -0.63068500 | -3.47475200 | -0.84812000 |

| | | | |
|---|---|---|---|
| O | 0.11285800 | -2.53295500 | -1.08823700 |
| O | -1.05290200 | -4.27357300 | -1.78850600 |
| C | -0.33739700 | -3.10185600 | 4.03232200 |
| H | -0.71627900 | -2.07576100 | 4.10393800 |
| H | -0.72009600 | -3.72235300 | 4.84560500 |
| H | 0.75902000 | -3.09300700 | 4.02556800 |
| C | -0.71779700 | -3.93717300 | -3.14457500 |
| H | 0.37114400 | -3.93224000 | -3.26895200 |
| H | -1.17352800 | -4.71308000 | -3.76435200 |
| H | -1.12593600 | -2.94586100 | -3.37964000 |

**c-2a-f2**

| | | | |
|---|---|---|---|
| C | 1.46176200 | 1.24883100 | 1.91902900 |
| C | 2.06113100 | 2.06786400 | 2.87134500 |
| C | 1.39736000 | 3.24134200 | 3.23707900 |
| C | 0.18114900 | 3.57877000 | 2.64121600 |
| C | -0.34421300 | 2.71057400 | 1.68858500 |
| H | 3.03349600 | 1.80479500 | 3.28582100 |
| H | 1.83938500 | 3.90321400 | 3.98311300 |
| H | -0.33854100 | 4.50424700 | 2.89336200 |
| C | 2.08786100 | -0.02041800 | 1.34574400 |
| H | 2.75314500 | -0.47723400 | 2.09640000 |
| N | 0.29286000 | 1.58562300 | 1.37385600 |
| N | 2.80430600 | 0.25629600 | 0.11133900 |
| C | -1.59556800 | 3.00387000 | 0.87532000 |
| H | -2.33071400 | 3.50484000 | 1.53339900 |
| C | -1.98836000 | 3.46043200 | -1.39897700 |
| C | -4.13219800 | 2.10763600 | -1.17753200 |
| H | -4.48821700 | 2.92335100 | -1.81984500 |
| H | -4.50623600 | 1.15722800 | -1.57551000 |
| C | 2.26573300 | -0.48093700 | -1.02087600 |
| N | -2.19406100 | 1.76433800 | 0.28468500 |
| O | -2.09427300 | 4.08234500 | -2.41869300 |
| C | -2.59445100 | 2.09432800 | -1.12518800 |
| H | -2.15933900 | 1.37879700 | -1.83909300 |
| N | -1.29740000 | 3.83775600 | -0.27173700 |
| C | -0.65957200 | 5.11189800 | -0.14285300 |
| C | 0.73514000 | 5.18251900 | -0.11929200 |
| C | -1.43244400 | 6.26996900 | -0.04982600 |
| C | 1.36021500 | 6.42041900 | 0.01365200 |
| C | -0.80130900 | 7.50611100 | 0.07626800 |
| C | 0.59141600 | 7.58065200 | 0.11233500 |
| H | 2.44892600 | 6.47977900 | 0.02805700 |
| H | -1.39973200 | 8.41539500 | 0.13961900 |
| H | 1.08108100 | 8.55034200 | 0.20838500 |
| C | -4.53801900 | 2.23644600 | 0.29180500 |
| H | -4.49826600 | 3.28452200 | 0.63024100 |
| C | -3.46469800 | 1.40063000 | 0.97802200 |
| H | -3.67017800 | 0.33963200 | 0.78086000 |
| H | -3.35662400 | 1.55493600 | 2.06091900 |
| H | -5.54677000 | 1.85394200 | 0.49011700 |
| H | -2.52068200 | 6.20078300 | -0.09573200 |
| H | 1.32169900 | 4.26575800 | -0.20905300 |
| H | 3.09395200 | -0.82304200 | -1.66203700 |
| C | 1.60924400 | -1.67327500 | -0.29789800 |
| H | 0.77901700 | -2.08447800 | -0.89178100 |
| N | 1.03662200 | -0.95787000 | 0.86289900 |
| H | 0.76464100 | -1.59984600 | 1.60811400 |
| S | 4.38132900 | 0.84932900 | 0.14602400 |
| O | 4.46314600 | 1.80557000 | -0.93557100 |
| O | 4.55289600 | 1.22043300 | 1.54210400 |
| C | 5.42158100 | -0.52065800 | -0.23421900 |
| C | 5.84641300 | -0.70864500 | -1.54835000 |
| C | 5.80039700 | -1.38694100 | 0.79399200 |
| C | 6.64735500 | -1.81117600 | -1.83846200 |
| H | 5.57226500 | 0.01352300 | -2.31949900 |
| C | 6.60516800 | -2.47364400 | 0.48199800 |
| H | 5.49284500 | -1.18947600 | 1.82210800 |
| C | 7.03557200 | -2.70647800 | -0.83469100 |

| | | | |
|---|---|---|---|
| H | 6.99313900 | -1.96772100 | -2.86178300 |
| H | 6.91824700 | -3.15518300 | 1.27562900 |
| C | 7.91939800 | -3.88249300 | -1.14080800 |
| H | 7.98615200 | -4.06875800 | -2.21962200 |
| H | 8.93833700 | -3.70169200 | -0.76660100 |
| H | 7.54915600 | -4.79207900 | -0.64662500 |
| C | 1.29056100 | 0.33358200 | -1.86318800 |
| C | 0.49962900 | -0.29879400 | -2.83471500 |
| C | 1.29709100 | 1.73420100 | -1.80157700 |
| C | -0.26733300 | 0.45249300 | -3.72460300 |
| H | 0.52738600 | -1.38717500 | -2.93560800 |
| C | 0.53462900 | 2.48384600 | -2.69730800 |
| H | 1.96592400 | 2.22552500 | -1.09257700 |
| C | -0.24954100 | 1.84708100 | -3.65899500 |
| H | -0.85785200 | -0.04979000 | -4.49277800 |
| H | 0.55520900 | 3.57441400 | -2.65485600 |
| H | -0.84616200 | 2.44511600 | -4.34899100 |
| C | 2.57792200 | -2.79220800 | 0.04684500 |
| C | 2.77637400 | -3.25293000 | 1.35055200 |
| C | 3.25188700 | -3.43095600 | -1.00227600 |
| C | 3.62233700 | -4.33557800 | 1.60302900 |
| H | 2.27807700 | -2.78338800 | 2.20244100 |
| C | 4.09585900 | -4.50851700 | -0.75337000 |
| H | 3.10884900 | -3.09138900 | -2.03185900 |
| C | 4.27958300 | -4.96791700 | 0.55235700 |
| H | 3.76270000 | -4.68446800 | 2.62684900 |
| H | 4.61004100 | -4.99525600 | -1.58353100 |
| H | 4.93447600 | -5.81816200 | 0.74817300 |
| Ni | -0.73049100 | 0.19802100 | 0.35283700 |
| C | -6.92432000 | -3.88699500 | -1.39731100 |
| C | -6.70872900 | -2.39342100 | -1.65508700 |
| H | -6.71361700 | -4.52634000 | -2.26466900 |
| H | -7.94202300 | -4.13301600 | -1.06423900 |
| H | -7.60805600 | -1.78189400 | -1.49747400 |
| H | -6.34622400 | -2.16334000 | -2.66642700 |
| N | -5.33294300 | -3.04760300 | 0.13129400 |
| C | -5.65695600 | -1.95963300 | -0.65762900 |
| C | -5.96389500 | -4.25221400 | -0.28260100 |
| O | -5.72685700 | -5.31465900 | 0.20042200 |
| O | -5.12130400 | -0.88075400 | -0.55148300 |
| C | -4.41463700 | -2.99667400 | 1.20748200 |
| C | -3.30599500 | -3.95203800 | 1.27079800 |
| C | -2.96323000 | -2.48220400 | 0.97061100 |
| H | -4.82553400 | -2.63326800 | 2.15295200 |
| H | -3.18847700 | -4.63367500 | 0.42805000 |
| H | -2.99866000 | -4.31902800 | 2.24941000 |
| C | -2.40223500 | -1.68189700 | 2.09933600 |
| O | -1.60272700 | -0.75856300 | 1.99729400 |
| O | -2.84490600 | -2.07509100 | 3.26163000 |
| C | -2.53228900 | -2.12063400 | -0.40934100 |
| O | -1.78840100 | -1.18796700 | -0.70063500 |
| O | -3.00092000 | -2.91717600 | -1.32753400 |
| C | -2.39699100 | -1.38140200 | 4.43897700 |
| H | -2.66710300 | -0.32141600 | 4.36764500 |
| H | -2.90978500 | -1.86066500 | 5.27587300 |
| H | -1.31010300 | -1.48445900 | 4.53682700 |
| C | -2.71882300 | -2.59445100 | -2.69933100 |
| H | -1.63632000 | -2.61312000 | -2.86970300 |
| H | -3.21740900 | -3.36450200 | -3.29308400 |
| H | -3.11371900 | -1.59521900 | -2.92427500 |

**c2-2a**

| | | | |
|---|---|---|---|
| C | -0.63533700 | -2.54698900 | -1.11522300 |
| C | -0.92398300 | -3.90879400 | -1.14014600 |
| C | -2.21269900 | -4.29404800 | -1.51498700 |
| C | -3.17826300 | -3.33695100 | -1.84426100 |
| C | -2.81472400 | -1.99935700 | -1.77668600 |
| H | -0.14818400 | -4.63645600 | -0.90478200 |
| H | -2.46772600 | -5.35422600 | -1.55010800 |

| | | | |
|---|---|---|---|
| H | -4.19215100 | -3.62664700 | -2.12287500 |
| C | 0.70158000 | -1.88965900 | -0.78894100 |
| H | 1.26011800 | -1.76489700 | -1.73547300 |
| N | -1.57578600 | -1.64877600 | -1.43011600 |
| N | 1.53209000 | -2.51116000 | 0.22513600 |
| C | -3.76553100 | -0.82724900 | -1.95230900 |
| H | -4.54992700 | -1.09725900 | -2.68423700 |
| C | -4.43497900 | 0.96333600 | -0.58195800 |
| C | -4.92793600 | 1.79640600 | -2.98886000 |
| H | -5.90706600 | 1.38648500 | -2.70612400 |
| H | -5.06366000 | 2.87519800 | -3.12524400 |
| C | 1.84593800 | -1.55580500 | 1.29605700 |
| N | -3.03804300 | 0.40531500 | -2.37876600 |
| O | -4.83955600 | 1.59456600 | 0.35769600 |
| C | -3.87958900 | 1.52095400 | -1.88380500 |
| H | -3.26045800 | 2.40581600 | -1.68075600 |
| N | -4.34508400 | -0.40376200 | -0.68470200 |
| C | -4.77149900 | -1.30206100 | 0.34394900 |
| C | -3.82413400 | -1.86418500 | 1.20555200 |
| C | -6.12616900 | -1.60002500 | 0.48748800 |
| C | -4.23824200 | -2.73735800 | 2.20793700 |
| C | -6.53516500 | -2.46990400 | 1.49790300 |
| C | -5.59334700 | -3.04049200 | 2.35337700 |
| H | -3.49943500 | -3.17480100 | 2.88039800 |
| H | -7.59416500 | -2.70051200 | 1.61727400 |
| H | -5.91737400 | -3.72188200 | 3.14079600 |
| C | -4.37273500 | 1.09974200 | -4.25439300 |
| H | -5.02618200 | 0.27315300 | -4.56349600 |
| C | -2.98443500 | 0.57734800 | -3.84966700 |
| H | -2.20425000 | 1.32327600 | -4.06117600 |
| H | -2.69538000 | -0.36659800 | -4.33297100 |
| H | -4.30112900 | 1.77983800 | -5.11134600 |
| H | -6.85440400 | -1.14127800 | -0.18320000 |
| H | -2.76849200 | -1.60412200 | 1.09525900 |
| H | 2.89768700 | -1.67003200 | 1.60131800 |
| C | 1.59099800 | -0.19950400 | 0.58037600 |
| H | 1.29902500 | 0.57181800 | 1.30438700 |
| N | 0.39731600 | -0.56441300 | -0.20743800 |
| H | -0.28964000 | -0.76603400 | 0.53359900 |
| S | 2.60643400 | -3.74025200 | -0.19311400 |
| O | 2.73617300 | -4.55466800 | 0.99274900 |
| O | 2.04585400 | -4.23887400 | -1.44136500 |
| C | 4.15995600 | -2.97493200 | -0.52704000 |
| C | 5.12887000 | -2.94461900 | 0.47419600 |
| C | 4.39702800 | -2.44220400 | -1.79683700 |
| C | 6.35302400 | -2.33826700 | 0.20022500 |
| H | 4.93143000 | -3.41633100 | 1.43860300 |
| C | 5.62407800 | -1.84299000 | -2.04673200 |
| H | 3.64477100 | -2.53470700 | -2.58206100 |
| C | 6.61768200 | -1.77966600 | -1.05583300 |
| H | 7.12463600 | -2.31712200 | 0.97203400 |
| H | 5.82847600 | -1.42970700 | -3.03668300 |
| C | 7.94765300 | -1.14992400 | -1.36084900 |
| H | 8.51609200 | -0.94158300 | -0.44602500 |
| H | 8.55409100 | -1.82305700 | -1.98558400 |
| H | 7.82165200 | -0.21304100 | -1.92208900 |
| C | 0.91962200 | -1.72249800 | 2.49679500 |
| C | 0.80744000 | -0.69620100 | 3.44661400 |
| C | 0.18850100 | -2.90108900 | 2.67321900 |
| C | -0.03985000 | -0.84462800 | 4.54419300 |
| H | 1.39959800 | 0.21746400 | 3.34768200 |
| C | -0.65798100 | -3.04502200 | 3.77417700 |
| H | 0.31801000 | -3.71991400 | 1.96474800 |
| C | -0.78217600 | -2.01600600 | 4.70637200 |
| H | -0.10668900 | -0.04806000 | 5.28705200 |
| H | -1.20197300 | -3.98047200 | 3.91566000 |
| H | -1.43436000 | -2.13577500 | 5.57269500 |
| C | 2.73012700 | 0.34444600 | -0.25182200 |
| C | 2.55359300 | 0.76143400 | -1.57457200 |

| | | | |
|---|---|---|---|
| C | 3.98189400 | 0.52446500 | 0.35058800 |
| C | 3.60697500 | 1.33968900 | -2.28764500 |
| H | 1.58356200 | 0.64220900 | -2.06201500 |
| C | 5.03211500 | 1.10266000 | -0.35805200 |
| H | 4.13454400 | 0.22491800 | 1.38995300 |
| C | 4.84765900 | 1.51604000 | -1.67971300 |
| H | 3.45795000 | 1.64504500 | -3.32491500 |
| H | 6.00385800 | 1.22682600 | 0.12352700 |
| H | 5.67248700 | 1.96452000 | -2.23565000 |
| Ni | -1.19754200 | 0.34428700 | -1.28853500 |
| C | 3.15098700 | 5.54404400 | 1.14827100 |
| C | 3.54486900 | 4.12397800 | 1.55644600 |
| H | 3.54903000 | 5.86420300 | 0.17685200 |
| H | 3.45631200 | 6.30489500 | 1.88067500 |
| H | 4.20070500 | 4.06902200 | 2.43504800 |
| H | 4.03601400 | 3.55585000 | 0.75111900 |
| N | 1.19281200 | 4.28609000 | 1.54204700 |
| C | 2.24220700 | 3.42650600 | 1.86865600 |
| C | 1.63473700 | 5.55482600 | 1.08639500 |
| O | 0.91184900 | 6.43571500 | 0.73456200 |
| O | 2.06419100 | 2.31720900 | 2.30342900 |
| C | -0.13579600 | 3.87997400 | 1.79100900 |
| C | -1.31725100 | 4.55481400 | 1.26332700 |
| C | -0.99266400 | 3.17586400 | 0.65851400 |
| H | -0.23714600 | 3.33579700 | 2.73315200 |
| H | -1.16125900 | 5.39990500 | 0.59368200 |
| H | -2.20597500 | 4.56821200 | 1.89437100 |
| C | -1.76200100 | 1.99816700 | 1.14569500 |
| O | -1.88176000 | 0.93282600 | 0.53446700 |
| O | -2.27024600 | 2.17210400 | 2.32118500 |
| C | -0.45324700 | 3.15879300 | -0.72438300 |
| O | -0.57791700 | 2.23746900 | -1.53679200 |
| O | 0.17265600 | 4.24834200 | -1.04333000 |
| C | -3.00425000 | 1.08691900 | 2.93645500 |
| H | -2.34879300 | 0.20995800 | 3.01833600 |
| H | -3.28391800 | 1.45880100 | 3.92474400 |
| H | -3.88959100 | 0.87084700 | 2.32681700 |
| C | 0.75873500 | 4.36810700 | -2.35032500 |
| H | -0.01906500 | 4.25957100 | -3.11486700 |
| H | 1.19271700 | 5.37081200 | -2.37897700 |
| H | 1.52702600 | 3.59500100 | -2.47669300 |

**c2-2a-f2**

| | | | |
|---|---|---|---|
| C | -1.23145900 | 1.97833000 | -1.39942700 |
| C | -1.53281700 | 3.30674300 | -1.69012300 |
| C | -0.56159200 | 4.06752900 | -2.34341900 |
| C | 0.67457700 | 3.50825600 | -2.68192200 |
| C | 0.90416100 | 2.18414600 | -2.33563100 |
| H | -2.51348800 | 3.71186600 | -1.44401600 |
| H | -0.77217400 | 5.10855200 | -2.59270600 |
| H | 1.44593600 | 4.09612100 | -3.18097100 |
| C | -2.14831600 | 0.94013700 | -0.76306000 |
| H | -2.67200000 | 0.40878300 | -1.57956800 |
| N | -0.03855900 | 1.46621800 | -1.72358600 |
| N | -3.08780300 | 1.38869000 | 0.24747900 |
| C | 2.23365000 | 1.46655200 | -2.49270800 |
| H | 2.75206200 | 1.84527600 | -3.39327400 |
| C | 3.71993400 | 0.45459500 | -0.98092300 |
| C | 4.28547800 | -0.67635600 | -3.23861400 |
| H | 5.03812400 | 0.12405700 | -3.21309000 |
| H | 4.82587800 | -1.62727600 | -3.18260000 |
| C | -2.92211200 | 0.62184100 | 1.48688500 |
| N | 2.05217900 | -0.01036300 | -2.55607000 |
| O | 4.44380600 | 0.28013800 | -0.02621500 |
| C | 3.32257400 | -0.56771100 | -2.03105800 |
| H | 3.15926100 | -1.55222300 | -1.57509800 |
| N | 3.07293600 | 1.61363300 | -1.30654400 |
| C | 3.20703300 | 2.83474100 | -0.57296300 |
| C | 2.21033900 | 3.20729200 | 0.33429900 |

| | | | |
|---|---|---|---|
| C | 4.32892900 | 3.63968200 | -0.76762800 |
| C | 2.33825700 | 4.39926400 | 1.04278500 |
| C | 4.45363000 | 4.82875700 | -0.04975000 |
| C | 3.45883400 | 5.20960600 | 0.85033300 |
| H | 1.56311100 | 4.69273800 | 1.75144000 |
| H | 5.33095900 | 5.45966100 | -0.19532600 |
| H | 3.55843900 | 6.14183300 | 1.40744300 |
| C | 3.38000600 | -0.55213000 | -4.48633800 |
| H | 3.59833500 | 0.37027300 | -5.04106900 |
| C | 1.94365400 | -0.53260000 | -3.93969400 |
| H | 1.53508900 | -1.55132000 | -3.86904700 |
| H | 1.24126900 | 0.07889500 | -4.52358900 |
| H | 3.51952900 | -1.38187700 | -5.18924900 |
| H | 5.10149800 | 3.32903600 | -1.47291900 |
| H | 1.34820300 | 2.55388900 | 0.48978400 |
| H | -3.90889200 | 0.37716200 | 1.90977600 |
| C | -2.18528200 | -0.64554800 | 0.96092600 |
| H | -1.55989700 | -1.08533700 | 1.75133100 |
| N | -1.28701500 | -0.00966800 | -0.02430300 |
| H | -0.69408100 | 0.58867500 | 0.56928500 |
| S | -4.59529400 | 2.00297800 | -0.20025100 |
| O | -4.95461700 | 2.92409800 | 0.85218900 |
| O | -4.36803700 | 2.41909700 | -1.57729700 |
| C | -5.73186900 | 0.65558300 | -0.19174500 |
| C | -6.53859400 | 0.45636000 | 0.92720100 |
| C | -5.82897500 | -0.15805700 | -1.32376700 |
| C | -7.44210400 | -0.60432400 | 0.91793700 |
| H | -6.47716600 | 1.14282700 | 1.77372800 |
| C | -6.73711400 | -1.20752300 | -1.31021200 |
| H | -5.23036300 | 0.05742900 | -2.21035300 |
| C | -7.55397800 | -1.44862000 | -0.19305800 |
| H | -8.08707500 | -0.76594400 | 1.78357500 |
| H | -6.83111300 | -1.84776800 | -2.18981600 |
| C | -8.54567200 | -2.57737100 | -0.21451900 |
| H | -8.93509400 | -2.79321900 | 0.78795400 |
| H | -9.40036700 | -2.32214600 | -0.85903900 |
| H | -8.09301100 | -3.49195600 | -0.62356600 |
| C | -2.07163900 | 1.36313000 | 2.51262600 |
| C | -1.59432700 | 0.68695900 | 3.64396500 |
| C | -1.76329900 | 2.71645300 | 2.34886000 |
| C | -0.80521700 | 1.34660600 | 4.58390900 |
| H | -1.86191300 | -0.35961700 | 3.81421500 |
| C | -0.97216600 | 3.37671300 | 3.29204300 |
| H | -2.18394900 | 3.25896500 | 1.50164300 |
| C | -0.48493000 | 2.69424500 | 4.40502000 |
| H | -0.45499600 | 0.81317300 | 5.46876000 |
| H | -0.76346900 | 4.44068800 | 3.16901100 |
| H | 0.11972400 | 3.21587900 | 5.14815900 |
| C | -3.06647000 | -1.72328600 | 0.36536600 |
| C | -2.80909400 | -2.28133300 | -0.89042000 |
| C | -4.11810500 | -2.24067600 | 1.13077300 |
| C | -3.58970200 | -3.33204400 | -1.37658700 |
| H | -1.98335600 | -1.90702500 | -1.49937600 |
| C | -4.89016900 | -3.29575000 | 0.65240000 |
| H | -4.33418800 | -1.82781600 | 2.11925900 |
| C | -4.62796200 | -3.84565600 | -0.60330700 |
| H | -3.38335600 | -3.75031200 | -2.36310300 |
| H | -5.70315200 | -3.68973000 | 1.26422000 |
| H | -5.23402700 | -4.67181500 | -0.97799600 |
| Ni | 0.45685900 | -0.43781600 | -1.18947300 |
| C | 6.51351600 | -1.23945000 | 2.03584800 |
| C | 6.74189300 | -1.60950500 | 0.57091300 |
| H | 6.29146300 | -0.16844900 | 2.15506100 |
| H | 7.34038800 | -1.48666200 | 2.71216900 |
| H | 7.56453400 | -2.32741100 | 0.43538600 |
| H | 6.92866700 | -0.75390000 | -0.08756000 |
| N | 4.68770300 | -2.51832800 | 1.28379800 |
| C | 5.46496200 | -2.28621100 | 0.14096900 |
| C | 5.27084600 | -1.99472300 | 2.46376300 |

| | | | |
|---|---|---|---|
| O | 4.81879900 | -2.12988600 | 3.56083300 |
| O | 5.11319000 | -2.63217800 | -0.95695100 |
| C | 3.50364600 | -3.26302900 | 1.15554200 |
| C | 2.49197300 | -3.41518000 | 2.19538300 |
| C | 2.09932100 | -2.51279000 | 1.01573800 |
| H | 3.58729800 | -4.05043900 | 0.40172200 |
| H | 2.65643900 | -2.88997800 | 3.13562100 |
| H | 1.96444500 | -4.36758100 | 2.23440400 |
| C | 1.25064300 | -3.06986900 | -0.06348600 |
| O | 0.69916800 | -2.41944200 | -0.95732200 |
| O | 1.16170400 | -4.36559100 | -0.02826100 |
| C | 2.06860200 | -1.04847600 | 1.26317800 |
| O | 1.49050100 | -0.22293200 | 0.54690800 |
| O | 2.69271400 | -0.69745500 | 2.33523100 |
| C | 0.39326800 | -5.03183000 | -1.04762400 |
| H | 0.83723500 | -4.83304600 | -2.03000000 |
| H | 0.44568900 | -6.09524600 | -0.80424000 |
| H | -0.64172400 | -4.67017500 | -1.01691000 |
| C | 2.77802400 | 0.69440000 | 2.69565500 |
| H | 1.76607100 | 1.10603400 | 2.80978100 |
| H | 3.31344000 | 0.70387200 | 3.64862700 |
| H | 3.33580700 | 1.22848600 | 1.91706400 |

5. The side reaction pathway in Supplementary Fig. 133

**TS3**

| | | | |
|---|---|---|---|
| C | 2.69873600 | 0.92887700 | -1.89300800 |
| C | 3.60133900 | 1.44516000 | -2.81887700 |
| C | 4.68801700 | 0.65094600 | -3.19061500 |
| C | 4.86145700 | -0.61178300 | -2.62394400 |
| C | 3.92115300 | -1.04862700 | -1.69487200 |
| H | 3.46561600 | 2.45165200 | -3.21198700 |
| H | 5.40912200 | 1.02501600 | -3.91855300 |
| H | 5.71730700 | -1.23779700 | -2.88037400 |
| C | 1.49295600 | 1.69829300 | -1.35078600 |
| H | 1.13780200 | 2.39553400 | -2.12781400 |
| N | 2.87595100 | -0.28780900 | -1.37784300 |
| N | 1.79007500 | 2.41613500 | -0.11929200 |
| C | 4.05735900 | -2.35063000 | -0.91638200 |
| H | 4.46876600 | -3.12001700 | -1.59764400 |
| C | 4.44745400 | -2.85030400 | 1.35291900 |
| C | 2.80836500 | -4.79679700 | 1.07251100 |
| H | 3.55055600 | -5.28909800 | 1.71417000 |
| H | 1.80985900 | -5.03020100 | 1.46887300 |
| C | 0.96970300 | 1.97172300 | 0.99869200 |
| N | 2.75911200 | -2.80438900 | -0.34149900 |
| O | 5.03866100 | -3.03759900 | 2.38012700 |
| C | 3.01969100 | -3.27248600 | 1.05706900 |
| H | 2.36157900 | -2.75909700 | 1.77339200 |
| N | 4.91849200 | -2.19205400 | 0.24077000 |
| C | 6.25221400 | -1.68713300 | 0.14430300 |
| C | 6.46695800 | -0.30681400 | 0.15555100 |
| C | 7.32704900 | -2.57191000 | 0.04979100 |
| C | 7.76410700 | 0.19076400 | 0.05437600 |
| C | 8.62328400 | -2.06856800 | -0.04381100 |
| C | 8.84100500 | -0.69057900 | -0.04637600 |
| H | 7.93426000 | 1.26776200 | 0.06620000 |
| H | 9.46721200 | -2.75595500 | -0.10886400 |
| H | 9.85720200 | -0.30132000 | -0.11788900 |
| C | 2.88926700 | -5.18101600 | -0.40622300 |
| H | 3.93848800 | -5.26383600 | -0.73310200 |
| C | 2.21475200 | -3.98452600 | -1.06769100 |
| H | 1.12776700 | -4.01472600 | -0.89851700 |
| H | 2.38347200 | -3.87366500 | -2.14739400 |
| H | 2.39353900 | -6.13130600 | -0.63935700 |
| H | 7.14524600 | -3.64777100 | 0.06763700 |
| H | 5.61381900 | 0.36897000 | 0.24597800 |
| H | 0.68259300 | 2.83927900 | 1.61355400 |
| C | -0.25735900 | 1.41780000 | 0.23683400 |
| H | -0.78101200 | 0.65018300 | 0.82919900 |

| | | | |
|---|---|---|---|
| N | 0.44437000 | 0.76475800 | -0.87912900 |
| H | -0.17071600 | 0.47754800 | -1.64036900 |
| S | 2.58176800 | 3.89415600 | -0.13537200 |
| O | 3.50890400 | 3.86731500 | 0.97370400 |
| O | 3.01052600 | 4.02550400 | -1.51935400 |
| C | 1.34688800 | 5.10895100 | 0.20680600 |
| C | 1.16607700 | 5.54578500 | 1.51788600 |
| C | 0.58948500 | 5.61906600 | -0.84909800 |
| C | 0.18024600 | 6.49608000 | 1.77515400 |
| H | 1.80838900 | 5.16315100 | 2.31315900 |
| C | -0.37936000 | 6.57399800 | -0.57031100 |
| H | 0.78452300 | 5.29289900 | -1.87198200 |
| C | -0.60315300 | 7.02258300 | 0.74108000 |
| H | 0.03199300 | 6.85122700 | 2.79660200 |
| H | -0.97106000 | 6.99218800 | -1.38743200 |
| C | -1.64242000 | 8.07405400 | 1.01344700 |
| H | -1.90552000 | 8.11588600 | 2.07766100 |
| H | -1.26594900 | 9.06659200 | 0.72294400 |
| H | -2.55532600 | 7.88981300 | 0.42965300 |
| C | 1.66053000 | 0.93765600 | 1.87774700 |
| C | 0.94933500 | 0.30459000 | 2.90518900 |
| C | 3.03239100 | 0.68764500 | 1.74682200 |
| C | 1.59550200 | -0.57115900 | 3.77535300 |
| H | -0.11548300 | 0.51658500 | 3.04175900 |
| C | 3.67809500 | -0.18956300 | 2.62044100 |
| H | 3.60109300 | 1.24204500 | 0.99858600 |
| C | 2.96083300 | -0.82690000 | 3.63131100 |
| H | 1.03792500 | -1.04272500 | 4.58632200 |
| H | 4.75136600 | -0.36545500 | 2.52776000 |
| H | 3.47480800 | -1.51466800 | 4.30440300 |
| C | -1.24003400 | 2.50434100 | -0.17598700 |
| C | -1.57011800 | 2.76023300 | -1.51054600 |
| C | -1.84933500 | 3.27578600 | 0.82358400 |
| C | -2.47232900 | 3.77541300 | -1.84414400 |
| H | -1.11943100 | 2.18406400 | -2.32185200 |
| C | -2.74621300 | 4.28831100 | 0.49525000 |
| H | -1.62242600 | 3.08066200 | 1.87474200 |
| C | -3.05955800 | 4.54371500 | -0.84272500 |
| H | -2.70555900 | 3.96875200 | -2.89251400 |
| H | -3.20088200 | 4.88449600 | 1.28797300 |
| H | -3.75698300 | 5.34219800 | -1.10081900 |
| Ni | 1.37144900 | -1.15926900 | -0.34714000 |
| C | -4.73234100 | 1.26164600 | -0.57743300 |
| C | -4.80706100 | 0.66526800 | -1.98218300 |
| H | -5.73286100 | 1.45176800 | -0.15317300 |
| H | -4.15253800 | 2.18837800 | -0.49308500 |
| H | -4.02842400 | 1.06753700 | -2.64830600 |
| H | -5.77109300 | 0.80710700 | -2.48781600 |
| N | -4.16721900 | -1.03061000 | -0.47054700 |
| C | -4.55353200 | -0.82153600 | -1.81430400 |
| C | -4.11380500 | 0.18435400 | 0.27201900 |
| O | -3.64483100 | 0.24649400 | 1.37210000 |
| O | -4.63847300 | -1.68884300 | -2.63418100 |
| C | -3.87633100 | -2.22315000 | 0.15688700 |
| C | -3.01860600 | -3.28062600 | -0.52108200 |
| C | -1.61234400 | -2.73307800 | -0.51307900 |
| H | -3.50492000 | -2.00490100 | 1.15909800 |
| H | -3.35709900 | -3.47774700 | -1.54253600 |
| H | -3.07434700 | -4.20138800 | 0.07554700 |
| C | -0.94215500 | -2.59953700 | 0.72774400 |
| O | 0.15172700 | -2.03341300 | 0.95818200 |
| O | -1.59670100 | -3.13479100 | 1.76411000 |
| C | -0.99181900 | -2.30611200 | -1.71946300 |
| O | 0.16560200 | -1.84198700 | -1.85257700 |
| O | -1.75027400 | -2.43861800 | -2.80168400 |
| C | -1.01282500 | -2.99267100 | 3.05641400 |
| H | -0.96282900 | -1.93245400 | 3.33560600 |
| H | -1.66292900 | -3.54234100 | 3.74373700 |
| H | 0.00068400 | -3.41326300 | 3.07372100 |

| | | | |
|---|---|---|---|
| C | -1.20049100 | -2.08292300 | -4.06336800 |
| H | -0.31826400 | -2.69525500 | -4.28832300 |
| H | -1.99217100 | -2.26667900 | -4.79488200 |
| H | -0.91019400 | -1.02342100 | -4.07733300 |
| C | -6.47570300 | -1.48827000 | 1.34791700 |
| C | -7.06238500 | -1.52498700 | 0.05891600 |
| C | -7.97180000 | -0.54660300 | -0.37277200 |
| C | -8.27520600 | 0.46260500 | 0.52750500 |
| C | -7.69270600 | 0.52111700 | 1.81762100 |
| C | -6.79446100 | -0.44135300 | 2.23684700 |
| C | -5.61981400 | -2.63179700 | 1.45303400 |
| H | -8.43819800 | -0.59016100 | -1.35734000 |
| H | -8.99269300 | 1.23221300 | 0.23823000 |
| H | -7.96968600 | 1.33466400 | 2.48800000 |
| H | -6.34460100 | -0.39541500 | 3.22959300 |
| N | -6.59645300 | -2.63647500 | -0.61004500 |
| C | -5.69544700 | -3.27331800 | 0.18772200 |
| H | -5.34816200 | -4.27253800 | -0.07024300 |
| C | -4.95440800 | -3.14743000 | 2.68475400 |
| H | -4.47008700 | -2.32725500 | 3.23448900 |
| H | -5.70833900 | -3.59968900 | 3.34584400 |
| H | -4.20116200 | -3.91008100 | 2.44636400 |
| C | -7.07112000 | -3.09325000 | -1.90716400 |
| H | -6.44753700 | -3.93070800 | -2.23748700 |
| H | -8.11705800 | -3.41937400 | -1.82750300 |
| H | -6.97979400 | -2.29216700 | -2.65027800 |

**IM3**

| | | | |
|---|---|---|---|
| C | 2.99011100 | 0.48805800 | -1.93797400 |
| C | 4.01994400 | 0.72982000 | -2.84355800 |
| C | 4.84205700 | -0.34001500 | -3.20288000 |
| C | 4.64278800 | -1.60006700 | -2.63829800 |
| C | 3.60453000 | -1.75227500 | -1.72308600 |
| H | 4.18029800 | 1.73540100 | -3.23098800 |
| H | 5.65107100 | -0.18587300 | -3.91814900 |
| H | 5.29193900 | -2.44188300 | -2.88330500 |
| C | 2.04480900 | 1.57016300 | -1.41219400 |
| H | 1.88475000 | 2.31350600 | -2.20983300 |
| N | 2.80588200 | -0.72945200 | -1.42658300 |
| N | 2.54738700 | 2.21967500 | -0.21075100 |
| C | 3.36969200 | -3.03364600 | -0.93225200 |
| H | 3.51335900 | -3.89493000 | -1.61233100 |
| C | 3.66717900 | -3.57089500 | 1.34531600 |
| C | 1.48590300 | -4.89597800 | 1.22619800 |
| H | 2.03626300 | -5.54656800 | 1.91824700 |
| H | 0.46342100 | -4.77099900 | 1.61284300 |
| C | 1.68024000 | 2.01190500 | 0.93989600 |
| N | 2.01311200 | -3.08032700 | -0.31646100 |
| O | 4.20770400 | -3.90711900 | 2.36253500 |
| C | 2.16911900 | -3.52485300 | 1.10611200 |
| H | 1.74208000 | -2.78677100 | 1.80126800 |
| N | 4.27887100 | -3.13044800 | 0.19548400 |
| C | 5.69929300 | -3.08414800 | 0.04302100 |
| C | 6.35457700 | -1.85058500 | 0.05060500 |
| C | 6.42001700 | -4.26982100 | -0.10521700 |
| C | 7.73820300 | -1.80381200 | -0.10538000 |
| C | 7.80446200 | -4.21751900 | -0.25559000 |
| C | 8.46162200 | -2.98687100 | -0.25951000 |
| H | 8.25277600 | -0.84247100 | -0.09622000 |
| H | 8.37286300 | -5.14182200 | -0.36309000 |
| H | 9.54546400 | -2.95014600 | -0.37470400 |
| C | 1.44190400 | -5.39089900 | -0.22035800 |
| H | 2.42076300 | -5.79599900 | -0.52519100 |
| C | 1.14042300 | -4.09691000 | -0.96766800 |
| H | 0.09282600 | -3.80122400 | -0.80752000 |
| H | 1.32490300 | -4.11380900 | -2.05025100 |
| H | 0.68953400 | -6.16961300 | -0.39568400 |
| H | 5.89590500 | -5.22693000 | -0.08499700 |
| H | 5.77393800 | -0.93434100 | 0.17753500 |

| | | | |
|---|---|---|---|
| H | 1.63312900 | 2.93438900 | 1.54036300 |
| C | 0.32605300 | 1.77017600 | 0.23182700 |
| H | -0.34505300 | 1.15371600 | 0.85170100 |
| N | 0.79329800 | 0.96495000 | -0.90633800 |
| H | 0.09482200 | 0.86144500 | -1.64320100 |
| S | 3.66622900 | 3.46749600 | -0.29450700 |
| O | 4.64014600 | 3.21688500 | 0.74395900 |
| O | 4.01732900 | 3.50532000 | -1.70665600 |
| C | 2.76385500 | 4.92442900 | 0.12907200 |
| C | 2.78881000 | 5.38145400 | 1.44515900 |
| C | 2.02608000 | 5.57478600 | -0.86125400 |
| C | 2.02810700 | 6.50051500 | 1.77762200 |
| H | 3.40925300 | 4.87366200 | 2.18573200 |
| C | 1.28143400 | 6.69223600 | -0.50862900 |
| H | 2.05902900 | 5.21879600 | -1.89224200 |
| C | 1.26387000 | 7.16712700 | 0.81259000 |
| H | 2.04063900 | 6.87137700 | 2.80403900 |
| H | 0.70588300 | 7.21733800 | -1.27382700 |
| C | 0.46048000 | 8.38802100 | 1.16394000 |
| H | 0.34806800 | 8.49889400 | 2.24940200 |
| H | 0.95481600 | 9.29414700 | 0.78249900 |
| H | -0.53887000 | 8.34584500 | 0.70777700 |
| C | 2.12133800 | 0.85600300 | 1.82796600 |
| C | 1.31657500 | 0.46391600 | 2.90490100 |
| C | 3.36933900 | 0.24458700 | 1.65381700 |
| C | 1.74703100 | -0.52579800 | 3.78560200 |
| H | 0.35152000 | 0.95261200 | 3.06818400 |
| C | 3.79658500 | -0.75126100 | 2.53496500 |
| H | 4.02919500 | 0.60467200 | 0.86204500 |
| C | 2.98583100 | -1.14264700 | 3.59886700 |
| H | 1.12303800 | -0.80579900 | 4.63596100 |
| H | 4.77543500 | -1.21688700 | 2.40673000 |
| H | 3.32898400 | -1.92434900 | 4.27799000 |
| C | -0.39793300 | 3.04831800 | -0.15209500 |
| C | -0.60075400 | 3.44286100 | -1.47807700 |
| C | -0.93664000 | 3.83653200 | 0.87263500 |
| C | -1.33373100 | 4.59465100 | -1.77670400 |
| H | -0.20062900 | 2.85583500 | -2.30716600 |
| C | -1.66224200 | 4.98743400 | 0.57895200 |
| H | -0.80036200 | 3.53440100 | 1.91432200 |
| C | -1.86846700 | 5.36744000 | -0.74966300 |
| H | -1.48203000 | 4.88655400 | -2.81750600 |
| H | -2.07390300 | 5.58922000 | 1.39069700 |
| H | -2.44077400 | 6.26715300 | -0.98093000 |
| Ni | 1.12405500 | -1.11640200 | -0.36297500 |
| C | -3.83541200 | 1.95948100 | -0.75409200 |
| C | -4.46965300 | 1.29742800 | -1.97478100 |
| H | -4.49811100 | 2.69949200 | -0.28013200 |
| H | -2.87944000 | 2.46409700 | -0.93610700 |
| H | -3.75371000 | 1.15073800 | -2.79706800 |
| H | -5.33722700 | 1.82306700 | -2.39173200 |
| N | -4.39526400 | -0.26084300 | -0.20736000 |
| C | -4.89123200 | -0.08083100 | -1.50317600 |
| C | -3.64436200 | 0.83192400 | 0.23675100 |
| O | -2.99437000 | 0.83600100 | 1.25213100 |
| O | -5.52299300 | -0.90148900 | -2.11071600 |
| C | -4.40716900 | -1.51329200 | 0.53206500 |
| C | -3.54806700 | -2.58541400 | -0.15589800 |
| C | -2.11330300 | -2.13401300 | -0.25626800 |
| H | -3.90895800 | -1.27536700 | 1.48220200 |
| H | -3.93281200 | -2.79541200 | -1.15968100 |
| H | -3.61374000 | -3.50563700 | 0.44525400 |
| C | -1.35487600 | -1.99779500 | 0.93132300 |
| O | -0.19898600 | -1.52859700 | 1.06135300 |
| O | -1.98596900 | -2.40918500 | 2.03442400 |
| C | -1.55319100 | -1.74060200 | -1.49560100 |
| O | -0.35536300 | -1.44840400 | -1.73738200 |
| O | -2.42608700 | -1.69341000 | -2.50824300 |
| C | -1.32699100 | -2.20819000 | 3.28013300 |

| | | | |
|---|---|---|---|
| H | -1.18790900 | -1.13471600 | 3.46365400 |
| H | -1.97805700 | -2.64840700 | 4.04118800 |
| H | -0.34585800 | -2.70077000 | 3.28746800 |
| C | -1.92509100 | -1.43714200 | -3.81324400 |
| H | -1.17473600 | -2.18617600 | -4.09636700 |
| H | -2.78972900 | -1.49237900 | -4.48134200 |
| H | -1.46650200 | -0.44020600 | -3.86857100 |
| C | -7.72893200 | -0.62258300 | 0.96110900 |
| C | -7.80234900 | -1.60676300 | -0.10665800 |
| C | -8.91692600 | -1.63741000 | -0.99150500 |
| C | -9.90200800 | -0.71377000 | -0.77488800 |
| C | -9.85672500 | 0.26468400 | 0.28770800 |
| C | -8.80071100 | 0.32155600 | 1.14064100 |
| C | -6.56097500 | -0.83873100 | 1.64123000 |
| H | -8.97419600 | -2.35231400 | -1.81064600 |
| H | -10.76960800 | -0.70729700 | -1.43772600 |
| H | -10.69004900 | 0.95926200 | 0.39123000 |
| H | -8.75792300 | 1.05619400 | 1.94525500 |
| N | -6.74019100 | -2.39231700 | -0.08378700 |
| C | -5.82879400 | -1.96058700 | 0.96559900 |
| H | -5.68351900 | -2.79838800 | 1.67318500 |
| C | -6.01793700 | -0.11622100 | 2.81709500 |
| H | -5.05111500 | 0.35565000 | 2.57334700 |
| H | -6.70995100 | 0.65515800 | 3.17257600 |
| H | -5.82510200 | -0.82085300 | 3.64081000 |
| C | -6.51657100 | -3.52434100 | -0.96653400 |
| H | -5.81052300 | -4.21389000 | -0.49142200 |
| H | -7.46496300 | -4.05396300 | -1.12234000 |
| H | -6.11406500 | -3.17473800 | -1.92659000 |

**IM4**

| | | | |
|---|---|---|---|
| C | 2.88590200 | 0.66040100 | -1.92996100 |
| C | 3.81409500 | 0.99407700 | -2.91257800 |
| C | 4.75493700 | 0.02931900 | -3.27977300 |
| C | 4.76612200 | -1.21799200 | -2.65440000 |
| C | 3.81455700 | -1.46501300 | -1.66848900 |
| H | 3.80968900 | 1.99024200 | -3.35341900 |
| H | 5.49054300 | 0.25689000 | -4.05242700 |
| H | 5.50797900 | -1.97573900 | -2.91041400 |
| C | 1.83070700 | 1.62315400 | -1.38107600 |
| H | 1.52579900 | 2.31737700 | -2.18180300 |
| N | 2.90852900 | -0.54220400 | -1.35607900 |
| N | 2.30873000 | 2.35995700 | -0.22033600 |
| C | 3.78739400 | -2.73198000 | -0.82186000 |
| H | 4.03806800 | -3.59160900 | -1.47237800 |
| C | 4.21258500 | -3.14819900 | 1.45905500 |
| C | 2.26246100 | -4.79612500 | 1.41724200 |
| H | 2.93765800 | -5.34550600 | 2.08598500 |
| H | 1.25315000 | -4.82734400 | 1.85162400 |
| C | 1.52814100 | 2.08174900 | 0.97578900 |
| N | 2.46530600 | -2.94605700 | -0.16469700 |
| O | 4.82270000 | -3.37761600 | 2.46645900 |
| C | 2.71882200 | -3.33575700 | 1.25926500 |
| H | 2.19490800 | -2.66186400 | 1.95440800 |
| N | 4.72398500 | -2.64996700 | 0.28367600 |
| C | 6.11939600 | -2.40945000 | 0.08744000 |
| C | 6.58643900 | -1.09639300 | -0.00795900 |
| C | 7.00366400 | -3.48551600 | 0.00103400 |
| C | 7.94499200 | -0.86049400 | -0.20693800 |
| C | 8.36243800 | -3.24335200 | -0.19114700 |
| C | 8.83146400 | -1.93391700 | -0.29938800 |
| H | 8.31302700 | 0.16345900 | -0.27852800 |
| H | 9.05846400 | -4.08059800 | -0.25032000 |
| H | 9.89586400 | -1.74856800 | -0.44769300 |
| C | 2.23058700 | -5.31166300 | -0.02263800 |
| H | 3.24306500 | -5.58030500 | -0.36570200 |
| C | 1.71995700 | -4.08497100 | -0.76938100 |
| H | 0.64854500 | -3.93384300 | -0.56767700 |
| H | 1.86059600 | -4.09124400 | -1.85862300 |

| | | | |
|---|---|---|---|
| H | 1.58901300 | -6.19079500 | -0.15912400 |
| H | 6.62663400 | -4.50463900 | 0.10227200 |
| H | 5.88013300 | -0.26767900 | 0.07603500 |
| H | 1.39969300 | 3.00882400 | 1.55686800 |
| C | 0.18559600 | 1.64923900 | 0.34399900 |
| H | -0.35046900 | 0.96053800 | 1.01577300 |
| N | 0.69517100 | 0.87411800 | -0.79560000 |
| H | -0.01637600 | 0.66759800 | -1.49650000 |
| S | 3.22831500 | 3.75395100 | -0.39275200 |
| O | 4.22752700 | 3.70745800 | 0.65057300 |
| O | 3.56243500 | 3.75753100 | -1.80846000 |
| C | 2.12563600 | 5.08916700 | -0.04787200 |
| C | 2.09773400 | 5.63462500 | 1.23400600 |
| C | 1.29555800 | 5.56156300 | -1.06626100 |
| C | 1.19288600 | 6.65935600 | 1.50410100 |
| H | 2.79152900 | 5.27214000 | 1.99469500 |
| C | 0.40676800 | 6.58789500 | -0.77603300 |
| H | 1.37050500 | 5.14699300 | -2.07299200 |
| C | 0.33684800 | 7.14750100 | 0.50995600 |
| H | 1.16477000 | 7.09991900 | 2.50238900 |
| H | -0.24264600 | 6.97464000 | -1.56422700 |
| C | -0.61976600 | 8.27193200 | 0.79264200 |
| H | -0.76708400 | 8.41582700 | 1.87022000 |
| H | -0.23278600 | 9.21548400 | 0.37892400 |
| H | -1.59615300 | 8.08749000 | 0.32250300 |
| C | 2.14623600 | 1.01192600 | 1.86668500 |
| C | 1.42289600 | 0.51129300 | 2.95684200 |
| C | 3.47043600 | 0.59586200 | 1.68276300 |
| C | 2.00401300 | -0.39908700 | 3.83710500 |
| H | 0.40167600 | 0.85945600 | 3.13758900 |
| C | 4.05277000 | -0.31434800 | 2.56691700 |
| H | 4.05646600 | 1.04057200 | 0.87613500 |
| C | 3.32109900 | -0.81880200 | 3.64102200 |
| H | 1.43588300 | -0.76928700 | 4.69222900 |
| H | 5.08880400 | -0.62901300 | 2.42811900 |
| H | 3.78456700 | -1.53808400 | 4.31744200 |
| C | -0.73093600 | 2.80474000 | -0.02444400 |
| C | -1.17818100 | 3.02929600 | -1.32961200 |
| C | -1.19983900 | 3.64065300 | 0.99833200 |
| C | -2.08162800 | 4.05796400 | -1.60848100 |
| H | -0.83328600 | 2.40800800 | -2.15901400 |
| C | -2.09952200 | 4.66697300 | 0.72369200 |
| H | -0.86081200 | 3.48555100 | 2.02642500 |
| C | -2.54794600 | 4.87551300 | -0.58271700 |
| H | -2.41688900 | 4.21840800 | -2.63425800 |
| H | -2.44929100 | 5.31011800 | 1.53291700 |
| H | -3.25165900 | 5.68082700 | -0.79955600 |
| Ni | 1.33292500 | -1.13177200 | -0.24323000 |
| C | -7.31395200 | -1.86178000 | -2.76447800 |
| C | -7.44687500 | -3.11265400 | -1.89723200 |
| H | -8.13783800 | -1.14571600 | -2.63814600 |
| H | -7.23488500 | -2.06641700 | -3.84029700 |
| H | -7.38771700 | -4.05163100 | -2.46453300 |
| H | -8.37323100 | -3.15655600 | -1.30935200 |
| N | -5.50279000 | -1.94156400 | -1.24850900 |
| C | -6.27118000 | -3.06901500 | -0.93998400 |
| C | -6.03527400 | -1.19265400 | -2.30527700 |
| O | -5.52872000 | -0.18913300 | -2.72879100 |
| O | -6.01005800 | -3.84497700 | -0.06196200 |
| C | -4.25636600 | -1.56614300 | -0.61405600 |
| C | -3.28633200 | -2.73801800 | -0.36800500 |
| C | -1.85624800 | -2.26316400 | -0.30433800 |
| H | -3.76906500 | -0.88947400 | -1.33031100 |
| H | -3.40280300 | -3.42685800 | -1.21441200 |
| H | -3.55699100 | -3.30028800 | 0.53017200 |
| C | -1.19017100 | -2.06794500 | 0.92425000 |
| O | -0.01261400 | -1.66954900 | 1.11342000 |
| O | -1.92676500 | -2.32271300 | 2.01778100 |
| C | -1.16859700 | -2.03105100 | -1.52963800 |

| | | | |
|---|---|---|---|
| O | 0.02891700 | -1.69953800 | -1.69583800 |
| O | -1.93087400 | -2.19396500 | -2.61079600 |
| C | -1.27633900 | -2.22763200 | 3.28213700 |
| H | -0.91102800 | -1.20774000 | 3.45539400 |
| H | -2.02807200 | -2.49983100 | 4.02955100 |
| H | -0.42595800 | -2.91989300 | 3.33330700 |
| C | -1.32291300 | -2.03451300 | -3.88804600 |
| H | -0.49292100 | -2.74119500 | -4.01129800 |
| H | -2.10800700 | -2.23619100 | -4.62182300 |
| H | -0.94307000 | -1.01196100 | -4.01203200 |
| C | -6.45483400 | 0.56095500 | 0.73803700 |
| C | -6.61937100 | -0.64433400 | 1.52876100 |
| C | -7.85616400 | -0.93231500 | 2.16934400 |
| C | -8.86860100 | -0.02685400 | 1.99129900 |
| C | -8.72980000 | 1.17423700 | 1.20442500 |
| C | -7.55386900 | 1.47472800 | 0.58886200 |
| C | -5.15448700 | 0.59002500 | 0.29691900 |
| H | -7.99206300 | -1.83633300 | 2.76166700 |
| H | -9.83386600 | -0.21949200 | 2.46405200 |
| H | -9.58880600 | 1.83837500 | 1.11161600 |
| H | -7.43933500 | 2.37749600 | -0.01193500 |
| N | -5.48776000 | -1.33446300 | 1.56624300 |
| C | -4.51707900 | -0.70823700 | 0.68420500 |
| H | -3.54419600 | -0.58513600 | 1.18805000 |
| C | -4.45307200 | 1.64904500 | -0.46584000 |
| H | -4.26669300 | 1.30182600 | -1.49486200 |
| H | -5.05406600 | 2.56405800 | -0.52310600 |
| H | -3.48399200 | 1.88998400 | 0.00009900 |
| C | -5.23099400 | -2.46377500 | 2.45339700 |
| H | -5.73738800 | -3.36646200 | 2.09083500 |
| H | -4.15102200 | -2.64111800 | 2.47833900 |
| H | -5.57690800 | -2.20628200 | 3.46391600 |

**TS4**

| | | | |
|---|---|---|---|
| C | 2.62242700 | 0.25491700 | -1.99482900 |
| C | 3.56330600 | 0.40206300 | -3.01007800 |
| C | 4.32898900 | -0.71118800 | -3.36204700 |
| C | 4.15611000 | -1.92360200 | -2.69317400 |
| C | 3.19750300 | -1.98558400 | -1.68618700 |
| H | 3.71010100 | 1.37079000 | -3.48508700 |
| H | 5.07340000 | -0.62820200 | -4.15503800 |
| H | 4.76213800 | -2.79799600 | -2.93462300 |
| C | 1.76685900 | 1.39068500 | -1.43621500 |
| H | 1.60756100 | 2.15059100 | -2.21948900 |
| N | 2.46182400 | -0.91832000 | -1.38316300 |
| N | 2.36745800 | 1.98291200 | -0.24908600 |
| C | 2.97363400 | -3.21252800 | -0.81410400 |
| H | 3.10527800 | -4.11249800 | -1.44484300 |
| C | 3.29061000 | -3.68885100 | 1.46837800 |
| C | 1.12534900 | -5.03927100 | 1.37032100 |
| H | 1.70116600 | -5.68693100 | 2.04405100 |
| H | 0.11216000 | -4.93529200 | 1.78553500 |
| C | 1.50178600 | 1.89071500 | 0.91875100 |
| N | 1.62068700 | -3.21569200 | -0.18148400 |
| O | 3.83892200 | -4.01436200 | 2.48412100 |
| C | 1.78870500 | -3.65785700 | 1.24105100 |
| H | 1.35863200 | -2.92187600 | 1.93696600 |
| N | 3.88901200 | -3.25225800 | 0.30961800 |
| C | 5.30837100 | -3.20922300 | 0.13812800 |
| C | 5.95079300 | -1.97378900 | 0.02884900 |
| C | 6.03854900 | -4.39743900 | 0.09080100 |
| C | 7.33224400 | -1.92882300 | -0.14584400 |
| C | 7.42097700 | -4.34568300 | -0.07650700 |
| C | 8.06567600 | -3.11449400 | -0.19935500 |
| H | 7.83734100 | -0.96595000 | -0.22789900 |
| H | 7.99770900 | -5.27060500 | -0.10486600 |
| H | 9.14800100 | -3.07864200 | -0.32811500 |
| C | 1.05152500 | -5.52837800 | -0.07692200 |
| H | 2.02255900 | -5.93307900 | -0.40496600 |

| | | | |
|---|---|---|---|
| C | 0.73429200 | -4.23178000 | -0.81315800 |
| H | -0.31061100 | -3.94374800 | -0.62242200 |
| H | 0.88939100 | -4.24540300 | -1.90061000 |
| H | 0.29550900 | -6.30622500 | -0.23955800 |
| H | 5.52528700 | -5.35390400 | 0.20369700 |
| H | 5.36378400 | -1.05467300 | 0.08579800 |
| H | 1.57221300 | 2.82280000 | 1.50096600 |
| C | 0.12365100 | 1.77472600 | 0.23381400 |
| H | -0.60479500 | 1.25714600 | 0.87800200 |
| N | 0.49647400 | 0.87018900 | -0.87116200 |
| H | -0.22515000 | 0.79987400 | -1.58933000 |
| S | 3.60351400 | 3.11421200 | -0.37278400 |
| O | 4.48867700 | 2.85010400 | 0.73983600 |
| O | 4.01901300 | 2.99319800 | -1.76121100 |
| C | 2.85895400 | 4.69486500 | -0.12846100 |
| C | 2.89495400 | 5.27666600 | 1.13727700 |
| C | 2.27443700 | 5.34193500 | -1.21967800 |
| C | 2.30442100 | 6.52643200 | 1.31421600 |
| H | 3.40455700 | 4.76747300 | 1.95726700 |
| C | 1.69881500 | 6.58917300 | -1.02079700 |
| H | 2.30490200 | 4.88630700 | -2.21070200 |
| C | 1.70073400 | 7.19823700 | 0.24483600 |
| H | 2.33291800 | 6.99786400 | 2.29828300 |
| H | 1.24679000 | 7.11255700 | -1.86599900 |
| C | 1.08767500 | 8.55829200 | 0.42740900 |
| H | 0.95849600 | 8.80489500 | 1.48840300 |
| H | 1.73054400 | 9.33059000 | -0.02110000 |
| H | 0.11027300 | 8.62083900 | -0.07222200 |
| C | 1.83758100 | 0.71337800 | 1.82618700 |
| C | 0.97534400 | 0.36474600 | 2.87560700 |
| C | 3.07304800 | 0.06065600 | 1.71785000 |
| C | 1.34040900 | -0.61912200 | 3.79298000 |
| H | 0.02394400 | 0.88991100 | 2.99966600 |
| C | 3.43970000 | -0.91877300 | 2.64216200 |
| H | 3.77334800 | 0.38403200 | 0.94635400 |
| C | 2.57371000 | -1.26481800 | 3.67830400 |
| H | 0.67271200 | -0.86504300 | 4.62046700 |
| H | 4.41293500 | -1.40696000 | 2.56535400 |
| H | 2.86907300 | -2.03502300 | 4.39231600 |
| C | -0.44154200 | 3.11928600 | -0.20250700 |
| C | -0.84075500 | 3.37854500 | -1.51693900 |
| C | -0.60413100 | 4.12773500 | 0.75597700 |
| C | -1.38967600 | 4.61469100 | -1.86904100 |
| H | -0.72841800 | 2.62564300 | -2.30096200 |
| C | -1.14639300 | 5.36162700 | 0.40838400 |
| H | -0.31331300 | 3.94863100 | 1.79428000 |
| C | -1.54530200 | 5.60908800 | -0.90656500 |
| H | -1.69032500 | 4.79880900 | -2.90162200 |
| H | -1.25539000 | 6.13631000 | 1.16952100 |
| H | -1.96986100 | 6.57610600 | -1.18083100 |
| Ni | 0.82036800 | -1.20756600 | -0.25511700 |
| C | -8.56483500 | -1.71181600 | -1.38658400 |
| C | -8.38869600 | -2.95000600 | -0.50786900 |
| H | -9.19223400 | -0.93412100 | -0.92928000 |
| H | -8.98747800 | -1.91774700 | -2.37851600 |
| H | -8.61241200 | -3.89128600 | -1.02922700 |
| H | -8.99299600 | -2.94555300 | 0.40845500 |
| N | -6.28689900 | -1.92210600 | -0.78749600 |
| C | -6.92229200 | -2.97448300 | -0.11781500 |
| C | -7.16961700 | -1.15262100 | -1.56011500 |
| O | -6.80745600 | -0.21764400 | -2.22045100 |
| O | -6.37886100 | -3.74084600 | 0.63062200 |
| C | -4.87789100 | -1.59570300 | -0.83304800 |
| C | -3.87777000 | -2.73358200 | -0.49520600 |
| C | -2.50512800 | -2.08529700 | -0.40597600 |
| H | -4.68280800 | -1.30805200 | -1.87727600 |
| H | -3.91509200 | -3.49783200 | -1.28132500 |
| H | -4.12458900 | -3.22338200 | 0.44896100 |
| C | -1.79433600 | -2.05555500 | 0.85764400 |

| | | | |
|---|---|---|---|
| O | -0.64099700 | -1.62972700 | 1.04771300 |
| O | -2.51102700 | -2.44443700 | 1.89150900 |
| C | -1.74139500 | -1.92877300 | -1.63309700 |
| O | -0.54366900 | -1.62020400 | -1.74174500 |
| O | -2.46104900 | -2.12862700 | -2.72225600 |
| C | -1.92801500 | -2.32844600 | 3.19121100 |
| H | -1.75640900 | -1.27033000 | 3.43049600 |
| H | -2.64896900 | -2.77408300 | 3.88203200 |
| H | -0.97202100 | -2.86536700 | 3.23270700 |
| C | -1.81622400 | -2.00061500 | -3.99126600 |
| H | -0.96601600 | -2.68999100 | -4.05646600 |
| H | -2.57418600 | -2.25088100 | -4.73773600 |
| H | -1.46174700 | -0.97141000 | -4.13430100 |
| C | -4.19937800 | 1.84689000 | 0.47371100 |
| C | -4.61327700 | 1.13356100 | 1.63472700 |
| C | -4.76996800 | 1.77838500 | 2.87948900 |
| C | -4.51019500 | 3.13274500 | 2.91909200 |
| C | -4.08979500 | 3.85907900 | 1.77106800 |
| C | -3.92816100 | 3.23499500 | 0.55642000 |
| C | -4.17671700 | 0.92198300 | -0.59411600 |
| H | -5.10112300 | 1.23962000 | 3.76698700 |
| H | -4.63656900 | 3.66811700 | 3.86145400 |
| H | -3.89969700 | 4.92878700 | 1.86176100 |
| H | -3.59977700 | 3.79249100 | -0.32310600 |
| N | -4.80161100 | -0.17282400 | 1.31740500 |
| C | -4.48892400 | -0.35335700 | -0.04158100 |
| H | -3.19419400 | -0.73345700 | -0.15584100 |
| C | -3.86374900 | 1.18768700 | -2.02594000 |
| H | -4.77687600 | 1.03972900 | -2.62450600 |
| H | -3.49918500 | 2.21253300 | -2.16580500 |
| H | -3.10586700 | 0.48601100 | -2.41116400 |
| C | -5.31633800 | -1.15598600 | 2.25247100 |
| H | -6.35876900 | -0.92098600 | 2.51475900 |
| H | -5.28002900 | -2.15854600 | 1.81694700 |
| H | -4.71318500 | -1.14989600 | 3.17107900 |

**PC2**

| | | | |
|---|---|---|---|
| C | 2.50276700 | 0.00500200 | -2.07737500 |
| C | 3.38779900 | 0.02516400 | -3.15116700 |
| C | 4.00513000 | -1.17337200 | -3.51465100 |
| C | 3.74503400 | -2.34459200 | -2.80083600 |
| C | 2.85009000 | -2.27805900 | -1.73766900 |
| H | 3.61084600 | 0.96103500 | -3.66121900 |
| H | 4.70464800 | -1.19045300 | -4.35150000 |
| H | 4.23845800 | -3.28463800 | -3.05174900 |
| C | 1.81725300 | 1.23179700 | -1.48303400 |
| H | 1.72624200 | 2.02161100 | -2.24723400 |
| N | 2.25740900 | -1.12906100 | -1.42228200 |
| N | 2.53265600 | 1.70689400 | -0.30707000 |
| C | 2.54208100 | -3.44841400 | -0.81776900 |
| H | 2.56632100 | -4.37550800 | -1.42183300 |
| C | 2.88198400 | -3.92642200 | 1.45745300 |
| C | 0.60578200 | -5.07720700 | 1.43203800 |
| H | 1.14572300 | -5.77072300 | 2.08960900 |
| H | -0.38103700 | -4.88937000 | 1.87998400 |
| C | 1.68582000 | 1.74376400 | 0.87971200 |
| N | 1.21205600 | -3.30419700 | -0.14471900 |
| O | 3.42486400 | -4.30505400 | 2.45692700 |
| C | 1.38188800 | -3.75892800 | 1.27511100 |
| H | 1.04279100 | -2.98614000 | 1.98108200 |
| N | 3.48215800 | -3.54358300 | 0.28095200 |
| C | 4.89639600 | -3.61281400 | 0.07293600 |
| C | 5.62111200 | -2.43281500 | -0.11103900 |
| C | 5.53934700 | -4.85128400 | 0.07004900 |
| C | 6.99735700 | -2.49536000 | -0.31714300 |
| C | 6.91769100 | -4.90606600 | -0.12792500 |
| C | 7.64416900 | -3.73165300 | -0.32607600 |
| H | 7.56645600 | -1.57585500 | -0.45711800 |
| H | 7.42702000 | -5.87013200 | -0.12117000 |

| | | | |
|---|---|---|---|
| H | 8.72292300 | -3.77948600 | -0.47859900 |
| C | 0.44325600 | -5.56034800 | -0.00954900 |
| H | 1.36276200 | -6.05093800 | -0.36657800 |
| C | 0.21967200 | -4.24130800 | -0.73986200 |
| H | -0.79046600 | -3.86674500 | -0.51142900 |
| H | 0.33250600 | -4.26958600 | -1.83223900 |
| H | -0.38394400 | -6.26755700 | -0.14611800 |
| H | 4.96384900 | -5.76218800 | 0.24267400 |
| H | 5.10438300 | -1.47115200 | -0.08546100 |
| H | 1.91295400 | 2.65061700 | 1.46070900 |
| C | 0.29502800 | 1.83655900 | 0.22824700 |
| H | -0.50156600 | 1.45068100 | 0.88596000 |
| N | 0.50509600 | 0.87326200 | -0.87943600 |
| H | -0.23581900 | 0.92712600 | -1.57944000 |
| S | 3.90859800 | 2.66205800 | -0.47249600 |
| O | 4.76580200 | 2.29721100 | 0.63397100 |
| O | 4.27972800 | 2.45108600 | -1.86273700 |
| C | 3.39683900 | 4.33477200 | -0.25688500 |
| C | 3.55095300 | 4.93682600 | 0.99022700 |
| C | 2.87863100 | 5.03070100 | -1.35224400 |
| C | 3.14895600 | 6.26209800 | 1.14507200 |
| H | 4.00647900 | 4.38147100 | 1.81202500 |
| C | 2.48858700 | 6.35055000 | -1.17486400 |
| H | 2.81781800 | 4.55258100 | -2.33110700 |
| C | 2.61396200 | 6.98499400 | 0.07239500 |
| H | 3.27244600 | 6.74974300 | 2.11376300 |
| H | 2.08896600 | 6.91101600 | -2.02259900 |
| C | 2.19961400 | 8.42070800 | 0.23093000 |
| H | 2.16092400 | 8.71684000 | 1.28630200 |
| H | 2.91440700 | 9.08362800 | -0.27932600 |
| H | 1.21360300 | 8.59743200 | -0.22243400 |
| C | 1.86494600 | 0.52955800 | 1.78402300 |
| C | 0.95492000 | 0.27622700 | 2.82329200 |
| C | 3.03780100 | -0.23563600 | 1.70593700 |
| C | 1.22109000 | -0.71844400 | 3.76362000 |
| H | 0.05318100 | 0.88683300 | 2.92784300 |
| C | 3.30647200 | -1.22082500 | 2.65618800 |
| H | 3.77509600 | 0.01181200 | 0.94136200 |
| C | 2.39900900 | -1.46591900 | 3.68594400 |
| H | 0.52216800 | -0.88789100 | 4.58455000 |
| H | 4.23532800 | -1.79143500 | 2.60282700 |
| H | 2.61783400 | -2.23851200 | 4.42458300 |
| C | -0.06648000 | 3.24642700 | -0.21214100 |
| C | -0.56984700 | 3.52892100 | -1.48400100 |
| C | 0.01856500 | 4.28802600 | 0.72059600 |
| C | -0.99678500 | 4.81760800 | -1.81537500 |
| H | -0.65337900 | 2.75266500 | -2.24900400 |
| C | -0.40484500 | 5.57243900 | 0.39462300 |
| H | 0.38875600 | 4.09406100 | 1.72986900 |
| C | -0.92221500 | 5.84079400 | -0.87444600 |
| H | -1.39310900 | 5.01634400 | -2.81202700 |
| H | -0.33577700 | 6.36864000 | 1.13787000 |
| H | -1.26369300 | 6.84524400 | -1.12852200 |
| Ni | 0.65138200 | -1.20313500 | -0.22821300 |
| C | -8.81124000 | -0.92956000 | -1.74281400 |
| C | -8.71183100 | -2.20113700 | -0.90151900 |
| H | -9.46132300 | -0.16270600 | -1.29866500 |
| H | -9.16279500 | -1.08955500 | -2.76993000 |
| H | -8.89051500 | -3.12121600 | -1.47528000 |
| H | -9.39056200 | -2.22880100 | -0.03928800 |
| N | -6.59858100 | -1.16217700 | -0.94065600 |
| C | -7.28405800 | -2.24209000 | -0.38914300 |
| C | -7.40202800 | -0.37850800 | -1.78683200 |
| O | -6.97768000 | 0.55191500 | -2.41268800 |
| O | -6.80367400 | -3.05425500 | 0.35916400 |
| C | -5.19189500 | -0.80683500 | -0.81059600 |
| C | -4.26332900 | -2.00671300 | -0.51310500 |
| C | -2.81159100 | -1.50253400 | -0.43518000 |
| H | -4.92531400 | -0.41892700 | -1.80550500 |

| | | | |
|---|---|---|---|
| H | -4.37365600 | -2.76586000 | -1.29664800 |
| H | -4.53803600 | -2.48194600 | 0.43009400 |
| C | -2.07464800 | -1.77028300 | 0.86257800 |
| O | -0.89046500 | -1.49440700 | 1.04400200 |
| O | -2.81183200 | -2.22872000 | 1.82241600 |
| C | -1.97102500 | -1.72261500 | -1.67593000 |
| O | -0.75093300 | -1.60255900 | -1.73325600 |
| O | -2.68478900 | -1.96916300 | -2.73258500 |
| C | -2.24448700 | -2.29733200 | 3.14206900 |
| H | -2.07484600 | -1.27524000 | 3.50563200 |
| H | -2.98545400 | -2.81655400 | 3.75476500 |
| H | -1.29605900 | -2.84725700 | 3.11749000 |
| C | -2.01754000 | -2.09007900 | -3.99993600 |
| H | -1.30108100 | -2.91877900 | -3.96229300 |
| H | -2.80545000 | -2.28744600 | -4.73004300 |
| H | -1.49460900 | -1.15463900 | -4.23182600 |
| C | -3.75568600 | 2.09477700 | 0.97710100 |
| C | -4.15300200 | 1.24829800 | 2.04127400 |
| C | -3.88219900 | 1.56098400 | 3.38282900 |
| C | -3.16980900 | 2.72510400 | 3.63283100 |
| C | -2.75835800 | 3.57544400 | 2.58388100 |
| C | -3.05362600 | 3.27885600 | 1.26212700 |
| C | -4.22627700 | 1.48587500 | -0.23988100 |
| H | -4.23229500 | 0.93094700 | 4.20161800 |
| H | -2.94604800 | 3.00296600 | 4.66367300 |
| H | -2.22463400 | 4.49637200 | 2.82324700 |
| H | -2.75870600 | 3.95809600 | 0.45913000 |
| N | -4.82234100 | 0.16898800 | 1.51016300 |
| C | -4.87436400 | 0.32483000 | 0.13164200 |
| H | -2.86496700 | -0.38363400 | -0.39268700 |
| C | -4.00491900 | 1.98955500 | -1.63558200 |
| H | -4.88389200 | 1.79590000 | -2.26564600 |
| H | -3.82207600 | 3.07262800 | -1.62615200 |
| H | -3.12712500 | 1.52264300 | -2.12183600 |
| C | -5.57844700 | -0.75642200 | 2.33028000 |
| H | -6.45410100 | -0.25763300 | 2.77282200 |
| H | -5.91853400 | -1.61515100 | 1.74256300 |
| H | -4.94442700 | -1.12821400 | 3.14737000 |

**4a**

| | | | |
|---|---|---|---|
| C | 1.30716700 | 3.52601500 | -1.15926500 |
| C | 1.57543800 | 2.64659400 | -2.37440000 |
| H | 0.58605400 | 4.33334900 | -1.34907700 |
| H | 2.20876500 | 3.97141600 | -0.72168300 |
| H | 2.64756200 | 2.45350800 | -2.51900500 |
| H | 1.16585100 | 3.01936400 | -3.32133900 |
| N | 0.45656600 | 1.35519800 | -0.74769900 |
| C | 0.94323200 | 1.30770200 | -2.04581600 |
| C | 0.71094000 | 2.58766500 | -0.13302200 |
| O | 0.50336400 | 2.81626400 | 1.02846700 |
| O | 0.91168200 | 0.34060300 | -2.76605700 |
| C | 0.00364000 | 0.22581200 | 0.08317100 |
| C | 0.81452000 | -1.06318300 | -0.13230700 |
| C | 2.33411700 | -0.83254800 | -0.25406900 |
| H | 0.25108900 | 0.56942600 | 1.09541800 |
| H | 0.48860800 | -1.61800600 | -1.01907500 |
| H | 0.60202200 | -1.69834700 | 0.73882500 |
| C | 2.84136100 | 0.34589600 | 0.55761300 |
| O | 3.33965100 | 1.33546100 | 0.08641000 |
| O | 2.61368500 | 0.17858300 | 1.85557700 |
| C | 3.15502400 | -2.05901400 | 0.10407500 |
| O | 4.25739000 | -2.02111400 | 0.57436500 |
| O | 2.52035800 | -3.18986100 | -0.20669800 |
| C | 3.01895600 | 1.24930600 | 2.70584800 |
| H | 2.45304500 | 2.15674100 | 2.45655200 |
| H | 2.79809400 | 0.92480500 | 3.72671400 |
| H | 4.09294400 | 1.43867200 | 2.58422200 |
| C | 3.23963800 | -4.38991400 | 0.04627200 |
| H | 3.48448800 | -4.47013600 | 1.11346400 |

| | | | |
|---|---|---|---|
| H | 2.58530900 | -5.21046500 | -0.26200500 |
| H | 4.17452100 | -4.40353500 | -0.52932000 |
| C | -3.61308700 | -0.08913100 | 0.88375500 |
| C | -3.55455500 | -0.63181700 | -0.42171000 |
| C | -4.69147600 | -1.14385100 | -1.06516900 |
| C | -5.89251800 | -1.09637500 | -0.37390300 |
| C | -5.97145300 | -0.55572800 | 0.92693500 |
| C | -4.84509400 | -0.05425800 | 1.55965800 |
| C | -2.28083200 | 0.32686600 | 1.22446300 |
| H | -4.63669800 | -1.56898100 | -2.06804600 |
| H | -6.79556200 | -1.48770900 | -0.84496000 |
| H | -6.93459300 | -0.53675900 | 1.43860100 |
| H | -4.91143500 | 0.35882600 | 2.56814900 |
| N | -2.25908700 | -0.55315400 | -0.86590600 |
| C | -1.48715800 | 0.02365400 | 0.13710200 |
| H | 2.58123200 | -0.60453400 | -1.30100300 |
| C | -1.87615800 | 0.94103400 | 2.53065500 |
| H | -1.00241300 | 1.59604800 | 2.42118100 |
| H | -2.69685600 | 1.55622600 | 2.92646900 |
| H | -1.65036700 | 0.17443000 | 3.28891600 |
| C | -1.85820500 | -1.05086400 | -2.16207900 |
| H | -2.55222200 | -0.67657800 | -2.92862400 |
| H | -0.85377300 | -0.70222600 | -2.41752700 |
| H | -1.87805600 | -2.15250900 | -2.18496600 |
